# Warmer temperature accelerates reproductive senescence in mosquitoes

**DOI:** 10.3389/fphys.2025.1610310

**Published:** 2025-07-02

**Authors:** Lindsay E. Martin, Tania Y. Estévez-Lao, Tobias C. McCabe, Julián F. Hillyer

**Affiliations:** Department of Biological Sciences, Vanderbilt University, Nashville, TN, United States

**Keywords:** mosquito, reproduction, blood feeding, senescence, temperature, aging

## Abstract

To reproduce, most female mosquitoes must ingest blood to obtain nutrients for viable eggs. Global warming is increasing mosquito body temperature and hampering their reproduction. Moreover, because it takes several days to produce eggs and mosquitoes are short-lived, the age of blood feeding determines whether reproduction is feasible. Given that warmer temperature and aging both impair reproduction, we scrutinized whether temperature modifies the aging-based decline in fecundity and fertility. By rearing the African malaria mosquito, *Anopheles gambiae*, at three temperatures and offering them a blood meal at four ages, we demonstrate that warmer temperature reduces blood feeding propensity and blood meal mass. Warmer temperature and aging decrease survival, delay oviposition, and reduce oviposition success, fecundity, and fertility. Importantly, warmer temperature quickens the onset of the aging-dependent decline in fecundity and fertility, and at the warmest temperature of 32°C, mosquitoes are infertile. Warmer temperature accelerates reproductive senescence, which has implications for disease transmission in this warming world.

## 1 Introduction

A mosquito population can only be maintained if its members reproduce. Most mosquitoes are anautogenous, so they require a blood meal to complete oogenesis and lay eggs (Attardo et al., 2005). The blood meal provides proteins that are converted into lipids and yolk protein precursors through a process called vitellogenesis, culminating in egg production (Attardo et al., 2005). The blood meal also provides cholesterol, which is converted into the ecdysteroid, 20-hydroxyecdysone (20E), that together with other hormones, regulates vitellogenesis, oogenesis, and oviposition (Hansen et al., 2014). Ingesting blood causes major physiological changes in a female mosquito (Dana et al., 2005; Calle-Tobón et al., 2021; Pathak et al., 2025). For example, a blood meal increases body temperature (Benoit et al., 2011), alters metabolism and hormone production (Hansen et al., 2004; Hansen et al., 2014), induces structural and microbial changes in the midgut (Billingsley and Rudin, 1992; Oliveira et al., 2011), and modifies immune responses (Castillo et al., 2011; Bryant and Michel, 2013; Bottino-Rojas et al., 2015; Upton et al., 2015).

The rise in global temperatures is affecting the ability of many organisms to reproduce, but it is having a particularly profound effect on organisms that are unable to regulate their body temperature. Mosquitoes are part of that group; as ectothermic poikilotherms, mosquito body temperature is predicated by the temperature of the environment. Warmer environmental temperature increases body temperature, leading to faster metabolism (Neven, 2000; Angilletta et al., 2010), altered body composition (Barr et al., 2023), reduced survival (Olayemi et al., 2011; Agyekum et al., 2021; Agyekum et al., 2022a; Barr et al., 2024), and weakened immunity (Murdock et al., 2012; Mordecai et al., 2019; Martin and Hillyer, 2024; Barr et al., 2025). Importantly, warmer temperature reduces reproduction by decreasing egg hatching and fecundity (Costa et al., 2010; Ciota et al., 2014; Ezeakacha and Yee, 2019; Agyekum et al., 2022a). Indirectly modifying reproduction, mosquitoes that develop at warmer temperatures are smaller (Agyekum et al., 2021; Huxley et al., 2021; Agyekum et al., 2022a; Barr et al., 2023), which lowers their fecundity and survival (Briegel, 1990a; Takken et al., 1998; Agyekum et al., 2021).

In addition to temperature, aging modifies a mosquito’s ability to reproduce. With aging, mosquitoes undergo physiological deterioration through a process called senescence (Hillyer et al., 2005; Styer et al., 2007; Somé et al., 2024). This includes an aging-related decline in survival, fecundity, and fertility (McCann et al., 2009; Petersen et al., 2018; Ramírez-Sánchez et al., 2023; Barr et al., 2024). To reproduce, adult females must survive long enough to mate, take a blood meal, digest the blood meal, and complete oogenesis and oviposition. Because older mosquitoes are more likely to die and are less fecund, the age when a blood meal is ingested impacts the reproductive output.

Warmer temperature can accelerate senescence (Barr et al., 2023; Barr et al., 2024; Martin and Hillyer, 2024; Martin et al., 2024; Barr et al., 2025). For example, warmer temperature accelerates the aging-dependent decline in survival and body composition of sugar-fed mosquitoes (Barr et al., 2023; Barr et al., 2024), and warmer temperature accelerates an aging-dependent weakening of immune responses (Barr et al., 2024; Martin and Hillyer, 2024; Martin et al., 2024; Barr et al., 2025). However, whether warmer temperature modifies the aging-associated changes in blood feeding and reproduction is unknown. This is a critical question because the propensity to blood feed shapes a mosquito population and its ability to transmit disease.

Here, we tested whether warmer temperature accelerates the aging-dependent decline in blood feeding and reproduction in the African malaria mosquito, *Anopheles gambiae*. We observed that warmer temperature and aging reduce blood feeding, oviposition success, fecundity and fertility, and that mosquitoes are sterile at the warmest temperature of 32°C. Importantly, we discovered that warmer temperature accelerates the senescence of reproduction, quickening the aging-dependent decline in oviposition, fecundity, and fertility.

## 2 Materials and methods

### 2.1 Experimental design: mosquito rearing and blood feeding

A laboratory colony of *A. gambiae* (Giles *sensu stricto*, G3 strain; Diptera: Culicidae) was maintained at 27°C, with 75% relative humidity and a 12 h:12 h light:dark photoperiod. Eggs from this colony were hatched in three environmental chambers that were maintained at 75% relative humidity and at 27°C, 30°C or 32°C. Larvae were fed a mixture of koi food and baker’s yeast (2.8:1 ratio) daily. Pupae were separated daily, and upon eclosion, adults were fed 10% sucrose *ad libitum*. Rearing was done at these three temperatures because they are temperatures that mosquitoes may experience in nature and simulate rising global temperatures (Sinka et al., 2010; Pörtner et al., 2022). Female and male mosquitoes were maintained together until offered a blood meal.

At each temperature, adult mosquitoes at 3, 5, 10, and 15 days after eclosion were offered a blood meal (Supplementary Figure S1). These ages were chosen to capture the physiological changes that occur with aging, and because they encompass the timeline for parasite development within *A. gambiae* (Phillips et al., 2017). We selected 3 days as the youngest age because anautogenous mosquitoes undergo 3 days of adult maturation prior to being receptive to blood feeding (Fernandes and Briegel, 2005; Hansen et al., 2014), which we confirmed in preliminary experiments. Throughout this manuscript, age refers to the day of adulthood when the mosquito took its blood meal.

For blood feeding, mosquitoes were aspirated into a 16-ounce paper cup with a mesh top, starved for 1 hour, and offered for 30 min defibrinated sheep blood (Hemostat Laboratories, Dixon, CA, United States) heated to 37°C using a Hemotek membrane feeder (Hemotek Ltd., Blackburn, United Kingdom) with a Parafilm membrane (Amcor, Neenah, WI, United States). At the onset, the experimenter stimulated mosquitoes by breathing into the cup three times. Immediately after blood feeding, mosquitoes were anesthetized on ice, the proportion of mosquitoes that took a blood meal was calculated (see below), and the mosquitoes that blood fed were allocated into cups marked for the different experiments (Supplementary Figure S1). A subset of mosquitoes from each group were not offered a blood meal and instead were used for pre-blood meal measurements.

### 2.2 Blood feeding proportion and blood meal mass

The proportion of female mosquitoes that took a blood meal was calculated by counting the number of non-blood-fed and blood-fed females in a container. For each temperature-age combination, blood feeding success was measured across three to five independent biological trials, with an average of 159 females being offered a blood meal per trial. In total, 9,384 females were offered a blood meal.

Because rearing mosquitoes at the three temperatures has a small effect on adult size (Barr et al., 2023), relative blood meal mass was calculated by taking the mass of blood-fed mosquitoes and dividing it by the mass of non-blood-fed mosquitoes. Mass was measured by pooling five mosquitoes (either blood-fed or non-blood-fed) in a pre-weighed 1.5 mL microfuge tube, weighing the tube on an analytical balance (Denver Instrument, Inc., Denver, CO, United States), and dividing the mass of the mosquito content by five. For each temperature-age combination, blood meal mass was calculated across three to five biological trials, and each trial had approximately five biological replicates. For each biological trial, the biological replicate values of both blood-fed mosquitoes and non-blood-fed mosquitoes were averaged, and the mean blood-fed value was divided by the mean non-blood-fed value to obtain the relative blood meal size (a ratio) for that biological trial. In total, 578 mass measurements were made, derived from 2,890 female mosquitoes, and this yielded 45 relative blood meal mass values.

The proportion of females that took a blood meal was analyzed using a non-parametric beta regression with a logit link, accounting for temperature, age, and their interaction. The relative blood meal mass was found to be non-normal using the Shapiro-Wilk test, so data were log-transformed to achieve normality. The transformed data were then analyzed using a generalized linear regression with a gaussian distribution and identity link.

For both blood feeding proportion and relative blood meal mass, models were fit using the “glmmTMB” package (Brooks et al., 2017). Statistical significance of main and interactive effects of temperature and age were assessed using a type-II ANOVA with Kenward-Roger approximation of degrees of freedom using the “lmerTest” package (Kuznetsova et al., 2017). Sidak-adjusted *post hoc* pairwise comparisons were conducted using the “emmeans” and “multcomp” packages (Searle et al., 1980; Hothorn et al., 2008; Lenth, 2022).

### 2.3 Mosquito survival after a blood meal

To determine how warmer temperature and aging shape female survival after a blood meal, groups of ∼35 blood-fed females were returned to their respective temperatures and provided 10% sucrose daily. Survival was monitored daily until all individuals had died (Barr et al., 2024). For each temperature-age combination, survival was assessed in three to five independent biological trials. In total, survival was measured for 1,398 blood-fed females. No water was provided as an oviposition site in this experiment.

Survival was measured as the number of days alive after the blood meal (instead of days alive after eclosion) so that the survival of mosquitoes receiving a blood meal at an older age could be compared to the survival of mosquitoes receiving a blood meal at a younger age. Kaplan-Meier survival curves were created to visualize how temperature and age shape survival after a blood meal. Curves were fit using the “ggsurvfit” and “survminer” packages (Kassambara A et al., 2024; Sjoberg et al., 2024), and the median survival for each temperature-age group was determined.

To assess the hazard ratio, or the relative risk of dying on any given day, we scaled and centered the temperature and age variables. Then, we applied the Cox proportional hazards model using the “survival” package (Therneau and Grambsch, 2000), but residuals indicated that the proportional hazards assumption was violated. Thus, we instead used the “coxphw” package to apply a Cox non-proportional hazards model with weighted estimation (Dunkler et al., 2018; Barr et al., 2024), also accounting for experimental start date.

### 2.4 Oviposition success, fecundity, fertility, and oviposition-related survival

After a blood meal, we sequentially tracked oviposition success, fecundity, fertility, and oviposition-related survival. For each trial, blood-fed female mosquitoes from each temperature and age combination were individually placed in fly vials (Fisher Scientific, Waltham, MA, United States) with a mesh top. Cotton soaked in 10% sucrose solution was placed on top of each mesh, and the tubes were returned to their respective rearing temperature. Two days later, ∼80% of mosquitoes were provided an oviposition site by adding 5 mL of deionized water to the bottom of the tube whereas the other ∼20% were not provided an oviposition site.

For mosquitoes with an oviposition site, we quantified oviposition success (the proportion of mosquitoes that laid any eggs) and fecundity (the number of eggs laid by mosquitoes that laid eggs) by counting under a dissecting microscope (Nikon SMZ645, Nikon, Tokyo, Japan) the number of eggs laid on days 3 and 4 post blood meal. For each tube, the number of eggs laid on day 4 was calculated by counting all the eggs and subtracting the number of eggs counted on day 3.

For the mosquitoes that laid eggs on day 3 post blood meal, we quantified egg hatching success by calculating the percentage of eggs laid on day 3 that hatched on day 4 post blood meal, and fertility by counting the number of larvae on day 4 post blood meal. After counting the eggs and larvae on day 4 post blood meal, the water in the bottom of each tube was removed, leaving a dry tube except for the cotton soaked in 10% sucrose solution at the top of the tube.

For mosquitoes that had an oviposition site and for those without one, we measured survival beginning 2 days after a blood meal and ending when all mosquitoes had died. We compared the survival of females that were provided an oviposition site to females that were not provided one. We also compared survival of mosquitoes that were provided an oviposition site and laid eggs to mosquitoes that were provided an oviposition site but did not lay eggs. Survival was analyzed as described above. Oviposition, fecundity, fertility and survival outcomes were measured in three to five independent biological trials, each with ∼20 mosquitoes. In total, data were collected for 849 blood-fed females.

Data on the proportion of mosquitoes that laid eggs were analyzed by binomial regression (binomial family with logit link) with a random effect of trial number. Only mosquitoes that survived beyond 2 days post blood meal were included in the analysis, as those that died sooner were inherently unable to lay any eggs. Data on the number of eggs laid by mosquitoes that laid eggs (zeroes were excluded because those mosquitoes were captured in the proportion analysis above) were analyzed using a negative binomial regression (negative binomial family with log link). The percentage of eggs laid each day and the percentage of eggs that hatched were analyzed using a non-parametric Kruskal-Wallis chi-squared test, followed by Dunn’s *post hoc* tests. The number of larvae produced by mosquitoes that laid eggs was analyzed using a zero-inflated regression, where the count component (one or more larvae) was fit by a negative binomial regression with a log link and the zero component (zero larvae) was fit by a binomial regression with logit link using the “pscl” package (Zeileis et al., 2008). Statistical significance of main and interactive effects of warmer temperature and aging were assessed using a type-II ANOVA with Kenward-Roger approximation of degrees of freedom and Sidak-adjusted *post hoc* pairwise comparisons.

### 2.5 Statistical analysis

All statistical analyses described above were conducted using R Statistical Software, v4.4.1 (R-Core-Team, 2022). Graphs were generated in R and figures were assembled in Adobe Illustrator. Observed means and ANOVA p-values are presented in the main figures. Raw data (Supplementary Data 1), processed data including calculations, estimated marginal means and full statistical analyses (Supplementary Data 2), and R code (Supplementary Code 1) are presented in the Supplementary Material.

## 3 Results

### 3.1 Warmer temperature reduces blood feeding propensity and blood meal mass, but aging only reduces blood feeding propensity

Because *A. gambiae* are anautogenous mosquitoes that must acquire a blood meal to complete oogenesis (Attardo et al., 2005), we first asked how warmer temperature, aging, and their interaction alter the proportion of females that ingest a blood meal when one is offered. To do so, we reared mosquitoes at 27°C, 30°C or 32°C, and at each temperature, adult females at 3, 5, 10, and 15 days after eclosion were offered a blood meal (Supplementary Figure S1).

**FIGURE 1:**
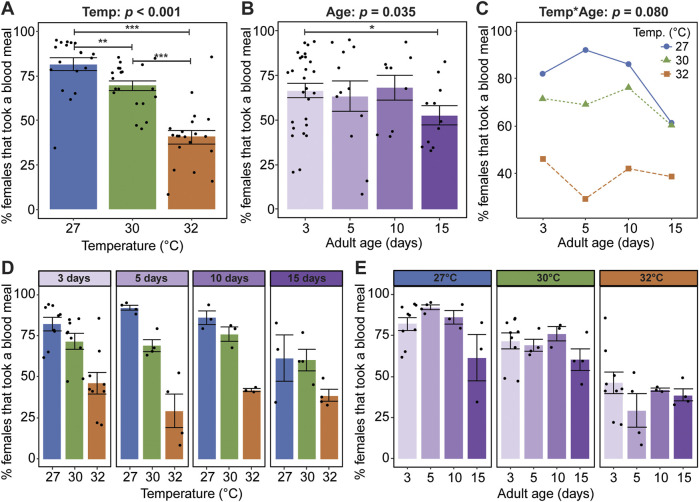
Warmer temperature and aging reduce blood feeding propensity. **(A)** Percentage of females reared at each temperature that took a blood meal, irrespective of age. **(B)** Percentage of females at each age that took a blood meal, irrespective of temperature. **(C)** Interaction plot showing the average percentage of females that took a blood meal. **(D,E)** The percentage of females that took a blood meal at each temperature within each age group **(D)** or at each age within each temperature group **(E)**. The same measurements are shown in **(A–E)** but grouped or arranged differently. Main effects of temperature (irrespective of age) and age (irrespective of temperature) are shown in **(A,B)**, respectively, and unaggregated data (values for each temperature-age combination) are shown in **(D,E)**. In **(A,B,D,E)**, bars represent means, whiskers indicate the SEM, and circles show individual data points. Statistical outcomes of main and interactive effects were determined by a non-parametric beta regression with a logit link with a type-II ANOVA with Kenward–Roger approximation of degrees of freedom and Sidak-adjusted *post hoc* multiple comparisons of means. Post-hoc comparisons in **(A,B)** are indicated by asterisks: ***p < 0.001, **p < 0.01, *p < 0.05.

Warmer temperature reduces the proportion of females that take a blood meal, regardless of age; 81% of mosquitoes took a blood meal at 27°C, whereas only 69% and 41% of mosquitoes took a blood meal at 30°C and 32°C, respectively (Figure 1A). Aging beyond 10 days slightly reduces the proportion of mosquitoes that take a blood meal, regardless of temperature, as seen in the 15% reduction between days 10 and 15 (Figure 1B). Temperature and age do not significantly interact to shape the propensity to blood feed (Figures 1C–E).

**FIGURE 2:**
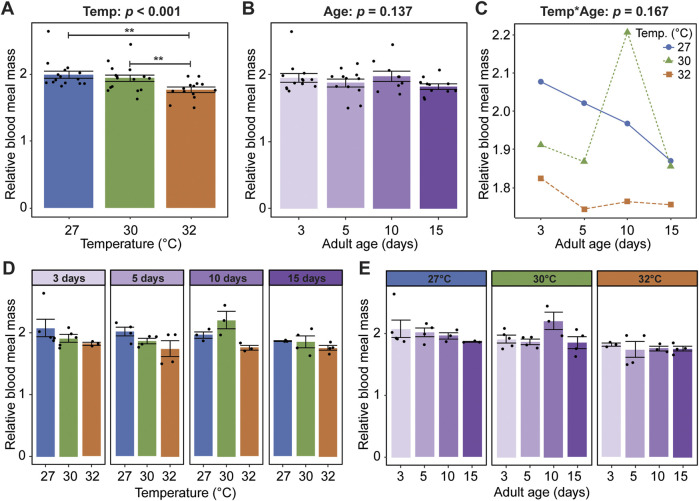
Warmer temperature, but not aging, reduces the mass of a blood meal. **(A)** Relative blood meal mass (mass blood-fed mass non-blood-fed) of females reared at each temperature, irrespective of age. **(B)** Relative blood meal mass of females blood-fed at each age, irrespective of temperature. **(C)** Interaction plot showing the average relative blood meal mass. **(D,E)** Relative blood meal mass at each temperature within each age group **(D)** or at each age within each temperature group **(E)**. The same measurements are shown in **(A–E)** but grouped or arranged differently. In **(A,B,D,E)**, bars represent means, whiskers indicate the SEM, and circles show individual data points. Statistical outcomes were determined by a generalized linear regression with a type-II ANOVA with Kenward–Roger approximation of degrees of freedom and Sidak-adjusted *post hoc* multiple comparisons of means. Post-hoc comparisons in **(A,B)** are indicated by asterisks: ***p < 0.001, **p < 0.01, *p < 0.05.

Given that warmer temperature and aging shape mosquito body size, dry weight, and body composition in sugar fed mosquitoes (Barr et al., 2023), we next asked how blood meal mass, relative to the mosquito’s body mass, is shaped by warmer temperature, aging, and their interaction. Warmer temperature reduces the blood meal mass, regardless of age. Relative to mosquitoes at 27°C, the blood meal mass of mosquitoes at 30°C and 32°C was 2.8% and 11.5% smaller, respectively (Figure 2A). Aging, regardless of temperature, does not meaningfully alter the blood meal mass (Figure 2B), and warmer temperature and aging do not interact to shape blood meal mass (Figures 2C–E). In summary, warmer temperature reduces blood feeding propensity and causes mosquitoes to take smaller blood meals, whereas aging only marginally reduces blood feeding propensity.

### 3.2 Warmer temperature accelerates the aging-dependent decline in survival after a blood meal

The survival of sugar-fed female mosquitoes is lower when the temperature is warmer and when they age (Barr et al., 2024). But for a population to be maintained, mosquitoes must take a blood meal and survive long enough to oviposit. Therefore, we measured how temperature and aging shape the survival of blood-fed mosquitoes.

**FIGURE 3:**
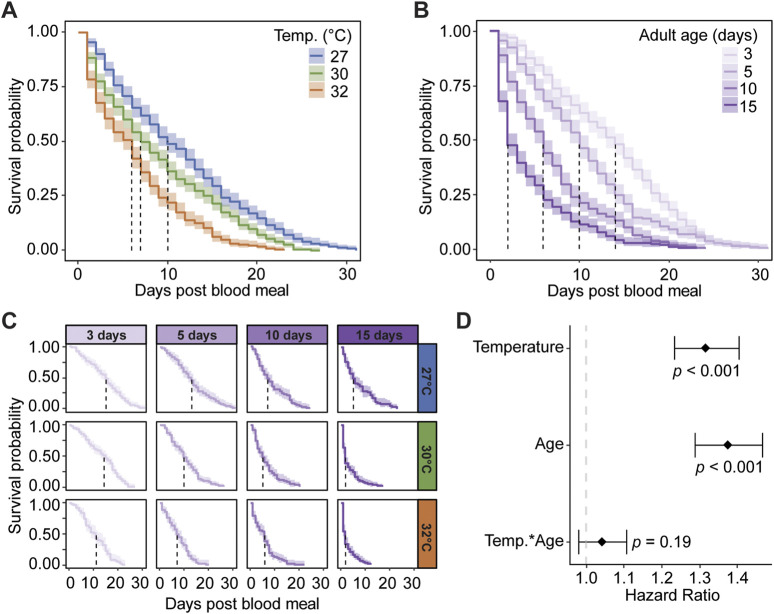
Warmer temperature and aging reduce mosquito survival after a blood meal. **(A)** Survival probability after a blood meal when mosquitoes are reared at different temperatures, irrespective of age. **(B)** Survival probability after a blood meal when feeding was done at different ages, irrespective of temperature. **(C)** Survival probability of mosquitoes reared at different temperatures after receiving a blood meal at different ages. The same measurements are shown in **(A–C)** but grouped or arranged differently, and the shading around each line represents the 95% confidence interval. **(D)** Hazard ratio, or risk of dying, associated with blood feeding and degree increase in temperature, day increase in age prior to acquiring the blood meal, and the interaction between temperature and age. Diamonds indicate the hazard ratio determined by a Cox non-proportional hazards model with weighted estimation; whiskers indicate 95% confidence intervals. A hazard greater than 1.0 indicates a greater risk of death.

Warmer temperature reduces median survival, regardless of age. Specifically, the median survival after a blood meal decreased from 10 days at 27°C to 6 days at 32°C (Figure 3A). Each 1°C increase in temperature increases the daily risk of a mosquito dying by 32% (Figure 3D).

Ingesting a blood meal at an older age lowers the median survival, regardless of temperature. When the blood meal was acquired at 3 days of age, the median survival after the blood meal was 14 days, but when acquired at 15 days, the median survival was 2 days (Figure 3B). Each day of aging prior to blood feeding increases the likelihood of a mosquito dying by 37% (Figure 3D).

Warmer temperature accelerates the aging-dependent decline in median survival (Figure 3C). For example, mosquitoes at 27°C had a median survival of 7 days when the blood meal was acquired on day 10, which was equal to the median survival at 32°C when the blood meal was acquired on day 5. Thus, the survival outcome of older mosquitoes at cooler temperatures is similar to the survival outcome of younger mosquitoes at warmer temperatures. However, the interaction of warmer temperature and aging only increased the daily risk of dying by 4% (Figure 3D). In summary, warmer temperature and aging reduce mosquito survival and increase the risk of dying after a blood meal, and at warmer temperatures, the aging-dependent decline in median survival occurs earlier in the mosquito’s life.

### 3.3 Warmer temperature accelerates the aging-dependent reduction in oviposition success

Following a blood meal, hormonal changes induce oogenesis and oviposition (Hansen et al., 2014), and oviposition success is affected by environmental factors (Impoinvil et al., 2007). We next asked how warmer temperature and aging interact to shape oviposition success, defined as the proportion of mosquitoes that lay eggs.

**FIGURE 4:**
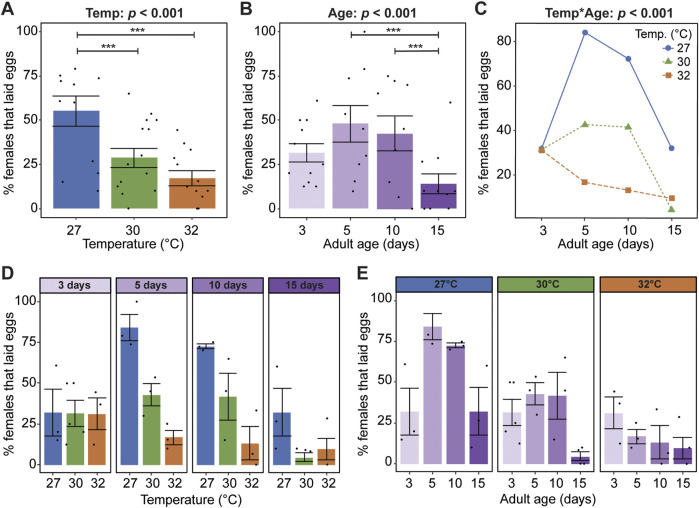
Warmer temperature accelerates the aging-dependent decline in oviposition success. **(A)** Percentage of females at each temperature that laid eggs, irrespective of age. **(B)** Percentage of females that laid eggs when the blood meal was acquired at each age, irrespective of temperature. **(C)** Interaction plot showing the average percentage of females that laid eggs at each temperature and age. **(D,E)** Percentage of females at each temperature within each age group that laid eggs **(D)** or at each age within each temperature group **(E)**. The same measurements are shown in **(A–E)** but grouped or arranged differently. In **(A,B,D,E)**, bars represent means, whiskers indicate the SEM, and circles show individual data points. Statistical outcomes were determined by a binomial regression followed by a type-II ANOVA with Kenward–Roger approximation of degrees of freedom and Sidak-adjusted *post hoc* multiple comparisons of means. Post-hoc comparisons in **(A,B)** are indicated by asterisks: ***p < 0.001.

Warmer temperature strongly reduces oviposition success, regardless of age: 55% of mosquitoes laid eggs at 27°C whereas only 29% and 17% of mosquitoes laid eggs at 30°C and 32°C, respectively (Figure 4A). Moreover, the age when a mosquito acquires a blood meal has a non-linear effect on oviposition, irrespective of temperature; oviposition success increased by 16% between 3 and 5 days of age, plateaued between 5 and 10 days of age, and decreased by 28% between 10 and 15 days of age (Figure 4B).

Warmer temperature accelerates the aging-dependent reduction in oviposition success (Figures 4C–E). For example, temperature did not affect oviposition success when the blood meal was acquired at 3 days of age but had a large effect when it was acquired at older ages (Figure 4D). Moreover, aging had different effects at different temperatures: an inverted parabola at 27°C, a plateau and sharp drop at 30°C, and a steady decline at 32°C (Figure 4E). In summary, oviposition success decreases when the temperature is warmer and as mosquitoes age beyond 10 days, and the aging-dependent decrease in oviposition success is accelerated at warmer temperatures.

### 3.4 Warmer temperature and aging reduce fecundity and delay oviposition, and the aging-dependent reduction in fecundity is accelerated at warmer temperature

**FIGURE 5:**
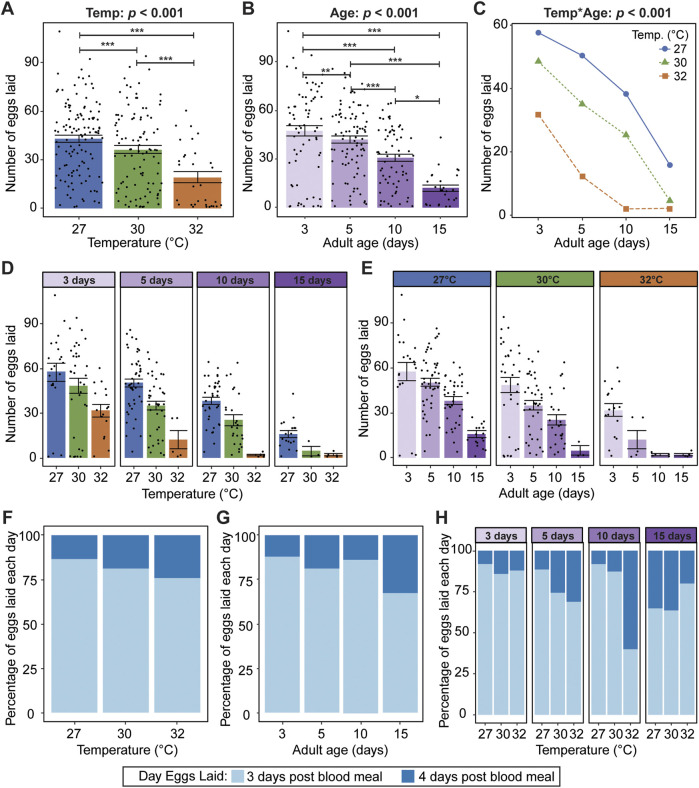
Warmer temperature and aging reduce fecundity and delay oviposition, and at warmer temperatures, the aging-dependent reduction in fecundity occurs earlier in life. **(A)** Number of eggs laid at each temperature, irrespective of age. **(B)** Number of eggs laid at each age, irrespective of temperature. **(C)** Interaction plot showing the average number of eggs at each temperature and age. **(D,E)** Number of eggs laid at each temperature within each age group **(D)** or at each age within each temperature group **(E)**. The same measurements are shown in **(A–E)** but grouped or arranged differently. In **(A,B,D,E)**, bars represent means, whiskers indicate the SEM, and circles show individual data points. **(F)** Percentage of eggs laid on days 3 and 4 post blood meal at each temperature, irrespective of age. **(G)** Percentage of eggs laid on days 3 and 4 after a blood meal acquired at different ages, irrespective of temperature. **(H)** Percentage of eggs laid on days 3 and 4 post blood meal at each temperature within each age group. The same measurements are shown in **(F–H)** but grouped or arranged differently. Data on the number of eggs laid by mosquitoes in **(A–E)** were analyzed using a negative binomial regression followed by a type-II ANOVA with Kenward–Roger approximation of degrees of freedom and Sidak-adjusted *post hoc* multiple comparisons of means. Post-hoc comparisons in **(A,B)** are indicated by asterisks: ***p < 0.001, **p < 0.01, *p < 0.05. Data on the percentage of eggs laid each day in **(F–H)** were analyzed using a non-parametric Kruskal-Wallis chi-squared test to compare all temperature-age groups (X^2^ = 26.749; d. f. = 11; *p* = 0.005).

We next analyzed the mosquitoes that laid eggs, and asked how warmer temperature and aging interactively shape fecundity, defined as the total number of eggs laid after a blood meal. Similar to oviposition success, warmer temperature reduces fecundity, regardless of age; mosquitoes laid 56% fewer eggs at 32°C than at 27°C (Figure 5A). Aging also reduces fecundity, regardless of temperature; mosquitoes laid 75% fewer eggs when the blood meal was acquired on day 15 than when it was acquired on day 3 (Figure 5B).

Warmer temperature accelerates the aging-dependent reduction in fecundity (Figures 5C–E). Fecundity declines with aging at each temperature, but egg laying was greatly reduced when the blood meal was acquired on day 15 and mosquitoes were at 30°C, a phenotype that occurred earlier—at day 10—when mosquitoes were at 32°C.

We next investigated whether temperature and aging alter the timing of oviposition by comparing the proportion of eggs laid on day 3 post blood meal versus day 4 post blood meal. Regardless of age, warmer temperature delays oviposition. At 27°C, 87% of eggs were laid by 3 days post blood meal, whereas at 32°C, 76% of eggs were laid by day 3 (Figure 5F). Regardless of temperature, aging beyond 10 days delays oviposition. Mosquitoes that acquired a blood meal at 3, 5 or 10 days of age laid ∼85% of their eggs by 3 days post blood meal, but this was reduced to 68% when the blood meal was acquired at 15 days of age (Figure 5G). Finally, temperature and aging interact to delay oviposition; the steep aging-related delay in oviposition occurs at an earlier age when the temperature is warmest (Figure 5H). Altogether, warmer temperature and aging reduce mosquito fecundity and delay oviposition, and warmer temperature accelerates the aging-dependent reduction in fecundity and delay in oviposition.

### 3.5 Mosquitoes are infertile at the warmest temperature and oldest age

Egg hatching occurs after embryogenesis is complete, within one to 2 days after oviposition (Yaro et al., 2006; Impoinvil et al., 2007; Mazigo et al., 2019). Factors such as temperature, water quality, and desiccation alter the rate of embryonic development and hatching success (Beier et al., 1990; Impoinvil et al., 2007; Kaiser et al., 2014). Thus, we next assessed how warmer temperature and aging shape egg hatching success (% of eggs that hatch) and fertility (absolute number of larvae).

**FIGURE 6:**
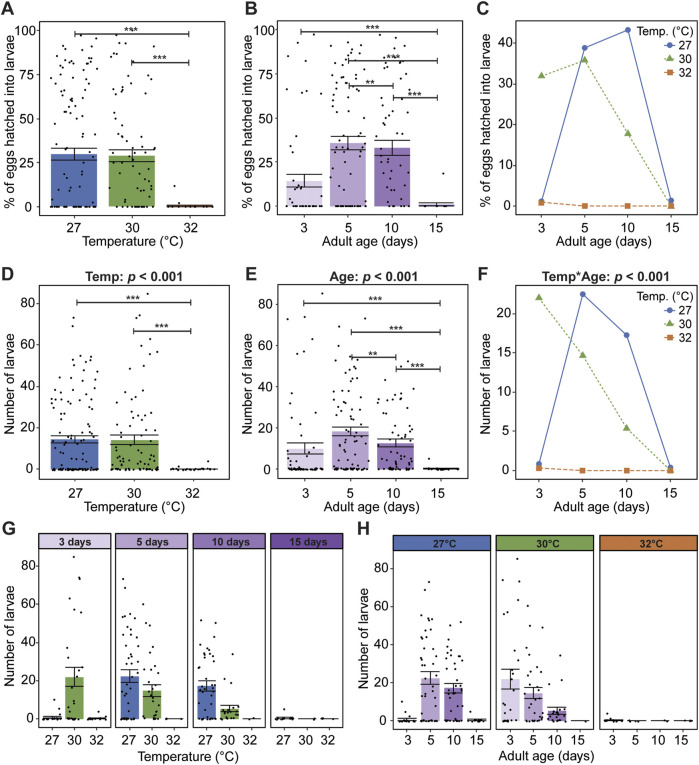
At the warmest temperature and oldest age, mosquitoes are infertile. **(A)** Percentage of eggs that hatched into larvae at each temperature, irrespective of age. **(B)** Percentage of eggs that hatched into larvae when the blood meal was acquired at each age, irrespective of temperature. **(C)** Interaction plot showing the average percentage of eggs that hatched into larvae at each temperature and age. **(D)** Number of larvae produced at each temperature, irrespective of age. **(E)** Number of larvae produced when the blood meal was acquired at each age, irrespective of temperature. **(F)** Interaction plot showing the average number of larvae produced at each temperature and age. **(G,H)** The number of larvae produced at each temperature within each age group **(G)** or at each age within each temperature group **(H)**. The same measurements are shown in **(A–C)**, and the same measurements are shown in **(D–H)**, but grouped or arranged differently. In **(A,B,D,E,G,H)**, bars represent means, whiskers indicate the SEM, and circles show individual data points. Data on the percentage of eggs hatching into larvae in **(A–C)** were analyzed using a non-parametric Kruskal-Wallis chi-squared test comparing all temperature-age groups (X^2^ = 62.585; d. f. = 11; *p* = 0.005), followed by Dunn’s *post hoc* tests. Data on the number of larvae produced in **(D–H)** were analyzed using a zero-inflated negative binomial regression followed by a type-II ANOVA with Kenward–Roger approximation of degrees of freedom and Sidak-adjusted *post hoc* multiple comparisons of means. Post-hoc comparisons are indicated by asterisks: ***p < 0.001, **p < 0.01.

Eggs laid by mosquitoes reared and maintained at 32°C do not hatch, regardless of age (Figure 6A). Specifically, ∼30% of the eggs laid at 27°C and 30°C hatched, whereas only 0.5% of the eggs laid at 32°C hatched. Moreover, eggs laid by mosquitoes that received a blood meal at 15 days of age do not hatch, regardless of temperature (Figure 6B). Egg hatching success increased from 15% to 36% between 3 and 5 days of age, plateaued between 5 and 10 days, and sharply decreased to 1% at 15 days. Warmer temperature and aging interact to decrease egg hatching success (Figure 6C). At 27°C, egg hatching was greatest when the blood meal was acquired at 10 days of age, at 30°C it was greatest at 5 days of age, and at 32°C meaningful egg hatching did not occur at any age.

We next investigated how warmer temperature and aging affect fertility, defined as the absolute number of larvae. Similar to egg hatching success, the warmest temperature of 32°C and the oldest age of 15 days render mosquitoes infertile. The number of larvae at 27°C and 30°C was similar, but the number of larvae at 32°C was 99% lower (Figure 6D). Additionally, relative to mosquitoes receiving a blood meal at 3 days of age, the number of larvae doubled when mosquitoes received a blood meal at 5 days of age (Figure 6E), decreased by about one third between 5 and 10 days of age, before sharply declining to nearly zero by 15 days of age (Figure 6E).

Warmer temperature and aging interact to decrease fertility. This interactive effect is largely driven by the warmest temperature and the youngest and oldest ages (Figure 6F). Specifically, when the blood meal was given at 3 days of age, the highest number of larvae occurred at 30°C, whereas when the blood meal was given beyond 3 days of age the highest number was at 27°C (Figure 6G). Moreover, the aging-dependent shape of the fertility curve was different for each temperature; the number of larvae at 27°C resembled an inverted parabola, at 30°C it was a steady decline, and at 32°C it was zero regardless of age (Figure 6H). In summary, warmer temperature and aging reduce egg hatching success and fertility, and warmer temperature quickens the aging-dependent decline in reproduction. At the warmest temperature of 32°C and oldest age of 15 days, mosquitoes are infertile.

### 3.6 Access to an oviposition site reduces survival, regardless of temperature or age

In one of the first experiments in this study, we measured how warmer temperature and aging interact to shape the survival of mosquitoes that ingest a blood meal (Figure 3). However, these mosquitoes did not have an opportunity to oviposit. Given that warmer temperature and aging interact to reduce both survival and oviposition, we next assessed how access to an oviposition site after a blood meal affects the survival of mosquitoes that are reared at different temperatures and ingest a blood meal at different ages.

**FIGURE 7:**
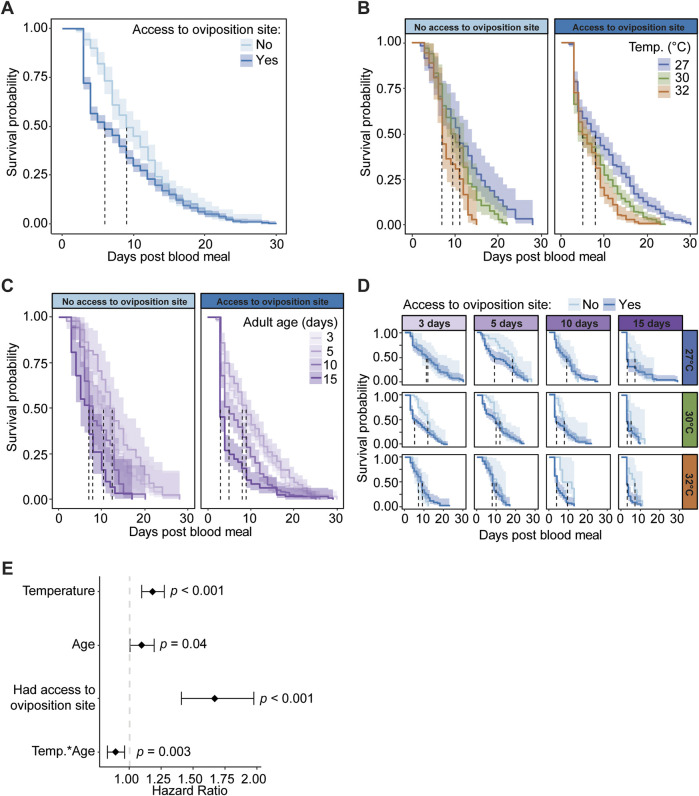
Access to an oviposition site reduces survival, regardless of temperature or age of blood feeding. **(A)** Survival probability after a blood meal with or without access to an oviposition site, irrespective of age or temperature. **(B)** Survival probability after a blood meal with or without access to an oviposition site at each temperature, irrespective of age. **(C)** Survival probability after a blood meal with or without access to an oviposition site when feeding was done at different ages, irrespective of temperature. **(D)** Survival probability after a blood meal with or without access to an oviposition site at each temperature and when feeding was done at different ages. **(A–D)**. The same measurements are shown in **(A–D)** but grouped or arranged differently, and the shading around each line represents the 95% confidence interval. **(E)** Hazard ratio, or risk of dying, associated with each degree increase in temperature, day increase in age prior to acquiring the blood meal, having access to an oviposition site, and the interaction between temperature and age. Diamonds indicate the hazard ratio determined by a Cox non-proportional hazards model with weighted estimation; whiskers indicate 95% confidence intervals. A hazard greater than 1.0 indicates a greater risk of death.

Mosquitoes that have access to an oviposition site have lower survival and an increased risk of dying than mosquitoes without access to an oviposition site, regardless of temperature or age. Specifically, access to an oviposition site decreases median survival by 3 days, and the daily risk of dying increases by 67% (Figures 7A,E). The increased mortality is most pronounced around day 3 post blood meal, which is when most oviposition takes place.

Warmer temperature reduces mosquito survival and increases the risk of dying, regardless of age or access to an oviposition site. Without an oviposition site, median survival decreased from 11 days to 7 days when the temperature warmed from 27°C to 32°C, and with an oviposition site, median survival decreased from 8 days to 5 days when the temperature warmed from 27°C to 32°C (Figure 7B). Regardless of whether an oviposition site was available, each 1°C increase in temperature increases the daily likelihood of dying by 18% (Figure 7E).

Aging beyond 5 days reduces mosquito survival and increases the risk of dying, regardless of the temperature or access to an oviposition site (Figures 7C,E). Without an oviposition site, median survival decreased 3.5 days between mosquitoes that took a blood meal at 3 versus 15 days of age, and with an oviposition site, median survival decreased 5 days between those same ages. Regardless of access to an oviposition site, each day of aging prior to blood feeding increases the daily likelihood of dying by 10% (Figure 7E).

Warmer temperature and aging interact to slightly increase survival when mosquitoes do not have access to an oviposition site, but not when an oviposition site is provided (Figures 7D,E). With an oviposition site, the median survival of 15-day-olds at 30°C was the same as 15-day-olds at 32°C (3 days), but without an oviposition site, the median survival of 15-day-olds at 30°C (5 days) was lower than 15-day-olds at 32°C (7 days). Thus, the oldest mosquitoes survive slightly longer at the warmest temperature when an oviposition site is not provided, and this interactive effect of warmer temperature and aging reduces the daily risk of dying by 11% (Figure 7E). In summary, mosquitoes that have access to an oviposition site have lower survival than mosquitoes that are deprived access to an oviposition site, regardless of temperature or age. Moreover, warmer temperature and aging reduce survival, regardless of whether the mosquitoes have access to an oviposition site.

### 3.7 When mosquitoes have access to an oviposition site, laying eggs improves survival

**FIGURE 8:**
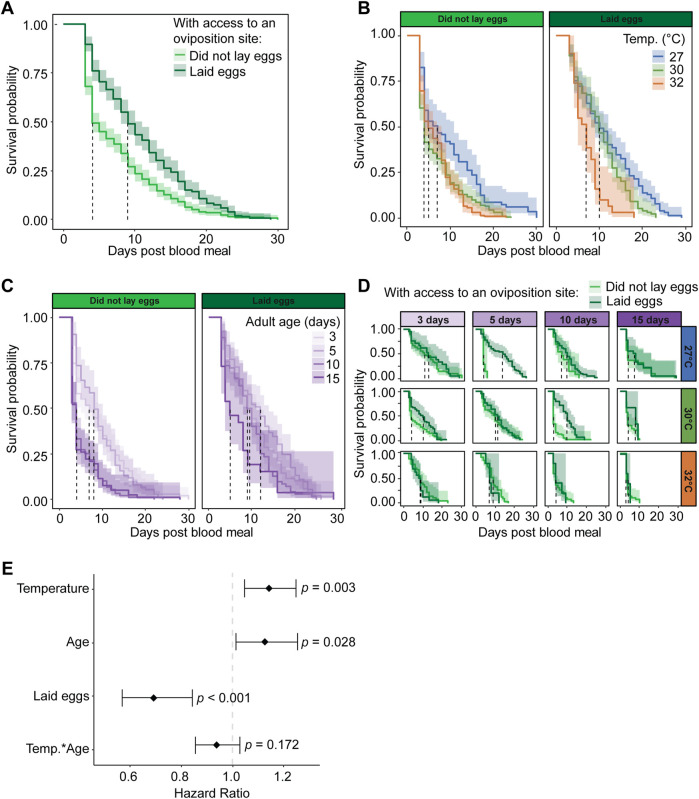
Laying eggs improves mosquito survival after a blood meal when mosquitoes have access to an oviposition site. **(A)** Survival probability after a blood meal when mosquitoes have access to an oviposition site and either lay eggs or do not lay eggs, irrespective of temperature or the age when the blood meal was acquired. **(B)** Survival probability after a blood meal when mosquitoes have access to an oviposition site and either lay eggs or do not lay eggs at each temperature, irrespective of age of blood feeding. **(C)** Survival probability when feeding was done at different ages and mosquitoes have access to an oviposition site and either lay eggs or do not lay eggs, irrespective of temperature. **(D)** Survival probability after a blood meal when mosquitoes have access to an oviposition site and either lay eggs or do not lay eggs at each temperature and each age when feeding was done. The same measurements are shown in **(A–D)** but grouped or arranged differently, and shading around each line represents the 95% confidence interval. **(E)** Hazard ratio, or risk of dying, associated with each degree increase in temperature, day increase in age prior to acquiring the blood meal, laying eggs versus not laying eggs when provided access to an oviposition site, and the interaction between temperature and age. Diamonds indicate the hazard ratio determined by a Cox non-proportional hazards model with weighted estimation; whiskers indicate 95% confidence intervals. A hazard greater than 1.0 indicates a greater risk of death. Survival data presented in this figure derives from the survival data of mosquitoes with access to an oviposition site that is presented in Figure 7.

Among those mosquitoes with access to an oviposition site, we next assessed whether the act of laying eggs alters survival. Mosquitoes that lay eggs have greater survival and a lower risk of dying than mosquitoes that do not lay eggs, regardless of temperature or age. Specifically, mosquitoes that laid eggs had a median survival that was 5 days greater than mosquitoes that did not lay eggs, and laying eggs reduced the daily risk of dying by 30% (Figures 8A,E). The greatest rate of mortality for mosquitoes that did not lay eggs occurred during the 2 days when the oviposition site was available, indicating that the risk of dying is highest when mosquitoes try to oviposit but are unable to do so.

Warmer temperature reduces survival and increases the risk of dying, regardless of age or egg laying. When mosquitoes did not lay eggs, median survival decreased by 2 days as the temperature warmed from 27°C to 32°C, and when mosquitoes laid eggs, median survival decreased by 3 days as the temperature warmed (Figure 8B). Regardless of egg laying or age, each 1°C increase in temperature increased the daily risk of dying by 14% (Figure 8E).

Aging reduces mosquito survival and increases the risk of dying, regardless of temperature or egg laying. When mosquitoes blood fed at 15 days of age, survival decreased by 44% relative to when mosquitoes blood fed at 3 days of age (Figure 8C), and each day of aging prior to blood feeding increased the daily risk of dying by 13% (Figure 8E).

When mosquitoes were given access to an oviposition site, three main interactive effects related to egg laying emerged: (i) temperature and aging, (ii) temperature and egg laying, and (iii) aging and egg laying. First, warmer temperature and aging slightly interact to improve survival when mosquitoes lay eggs. This interaction is driven by the effect of blood feeding at an older age, where 10- and 15-day-old mosquitoes have similar survival at every temperature instead of a warming-based decrease in survival (Figure 8D). The interactive effect of warmer temperature and aging marginally reduced the daily risk of dying by 7% (Figure 8E). Second, the warmest temperature causes a less pronounced decline in survival when mosquitoes do not lay eggs. Specifically, mosquitoes that laid eggs had lower survival at 32°C than at 30°C, whereas mosquitoes that did not lay eggs had similar survival at 32°C and 30°C (Figures 8B,D). Third, mosquitoes at older ages died faster when they did not lay eggs, but this effect did not occur at younger ages. Specifically, mosquitoes that ingested a blood meal at 10 or 15 days of age had a sharper decline in survival after a blood and a lower median survival when they did not lay eggs compared to when they laid eggs, whereas mosquitoes that ingested a blood meal at 3 or 5 days of age had similar survival regardless of egg laying (Figure 8C).

In summary, mosquitoes that have access to an oviposition site have higher survival when they lay eggs than when they do not lay eggs, regardless of temperature or age. Moreover, warmer temperature and aging reduce survival, regardless of whether a mosquito lays eggs.

### 3.8 Failure to lay eggs drives oviposition-related death

**FIGURE 9:**
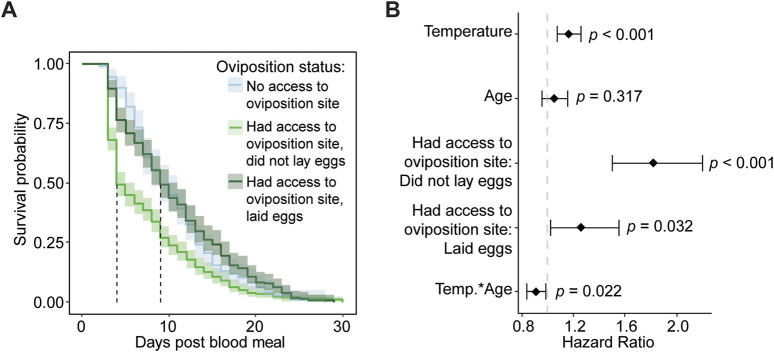
Failure to lay eggs increases risk of death. **(A)** Survival probability after a blood meal, irrespective of temperature or age when blood feeding was done, when mosquitoes have (i) no access to an oviposition site, (ii) access to an oviposition site but did not lay eggs, and (iii) access to an oviposition site and laid eggs. Shading around each line represents the 95% confidence interval. **(B)** Hazard ratio, or risk of dying, associated with each degree increase in temperature, day increase in age of acquiring the blood meal, having access to an oviposition site but not laying eggs (relative to no oviposition site access), and having access to an oviposition site and laying eggs (relative to no oviposition site access), and the interaction between temperature and age. Diamonds indicate the hazard ratio determined by a Cox non-proportional hazards model with weighted estimation; whiskers indicate 95% confidence intervals. A hazard greater than 1.0 indicates a greater risk of death. Survival data presented in this figure derives from the survival data presented in Figures 7, 8.

The findings that access to an oviposition site reduces survival but laying eggs increases survival appear contradictory, so we compared the survival of mosquitoes that did not have access to an oviposition site to the survival of mosquitoes that had access to an oviposition site and subsequently laid eggs or did not lay eggs. Mosquitoes that did not have access to an oviposition site had similar median survival as mosquitoes that were provided an oviposition site and laid eggs. This median survival was double the median survival of mosquitoes that were provided an oviposition site but did not lay eggs (Figure 9A). Moreover, although the daily risk of dying was 26% higher in mosquitoes that were provided an oviposition site and laid eggs than in mosquitoes that were not provided an oviposition site, the increased daily risk of dying jumped to 82% in mosquitoes that were provided with an oviposition site but failed to lay eggs (Figure 9B). In summary, the process of oviposition carries a risk of dying, but trying to oviposit and failing to do so carries the highest risk.

## 4 Discussion

**FIGURE 10:**
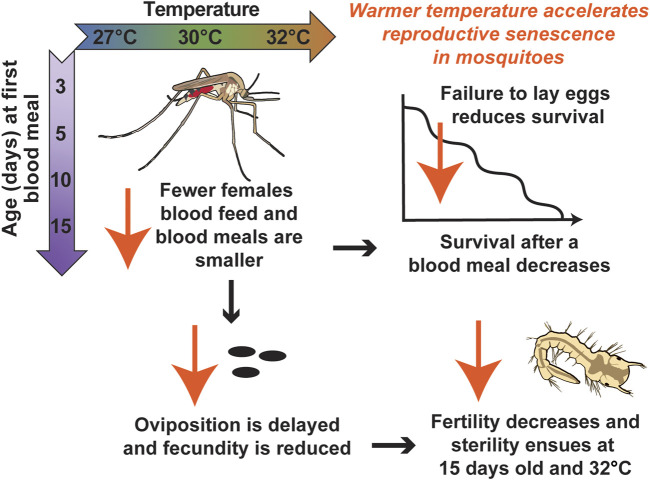
Diagrammatic summary illustrating that warmer temperature accelerates reproductive senescence in mosquitoes.

Successful reproduction is essential for the existence of a mosquito population. Here, we uncovered that warmer temperature and aging reduce mosquito blood feeding and reproductive outcomes (Figure 10). Importantly, we discovered that warmer temperature accelerates the senescence of mosquito reproduction.

Our experiments show that warmer temperature reduces blood feeding propensity and blood meal size, irrespective of aging. We also identified that warmer temperature reduces oviposition success, delays egg laying, and reduces fecundity and fertility. Importantly, at 32°C, mosquitoes are sterile. Warmer temperature has also been shown to reduce blood feeding propensity in *Aedes albopictus* (Costanzo and Occhino, 2023), and a warming-based decline in reproduction occurs in both mosquitoes and fruit flies (Impoinvil et al., 2007; Costa et al., 2010; Ciota et al., 2014; Christiansen-Jucht et al., 2015; Ezeakacha and Yee, 2019; Kirk Green et al., 2019; Klepsatel et al., 2019; Santos et al., 2021; Agyekum et al., 2022a). Although others have reported that *A. gambiae* do not lay eggs at 32°C (Agyekum et al., 2022a), we observed that mosquitoes at this temperature lay eggs, but that these eggs do not hatch. An earlier study reported that egg hatching in *A. gambiae* can occur at 31°C (Christiansen-Jucht et al., 2015), and therefore, small changes in temperature can have a large impact on fertility. Mosquitoes at warmer temperatures have a faster metabolic rate (Huestis et al., 2012; González-Tokman et al., 2020), which correlates with lower nutrient availability; these mosquitoes are slightly smaller and have lower protein content and teneral reserves (Briegel, 1990a; Briegel, 1990b; Barr et al., 2023). These reserves are needed for previtellogenic ovarian development (Caroci et al., 2004; Itoe et al., 2024), and after a blood meal, mosquitoes with low nutrient reserves are more likely to rebuild their own lipid and protein reserves rather than create egg yolk deposits (Briegel, 1990a; Briegel, 1990b; Caroci et al., 2004; Attardo et al., 2005). Therefore, we suspect that body composition at warmer temperature is insufficient for optimal reproduction, thereby shifting the blood meal usage toward resource allocation for maternal processes, including energy for survival, digestion, and excretion. Moreover, mosquitoes at warmer temperatures take smaller blood meals, likely reducing nutrient intake. Thus, we infer that mosquitoes at warmer temperatures have greater metabolic demands for nutrient stores, have fewer nutrient reserves, and acquire fewer building blocks with a blood meal, resulting in lower fertility.

After reaching reproductive maturity, decreased reproductive output with further aging is a nearly universal feature of organismal senescence (Lemaître and Gaillard, 2017). Aging decreases mosquito fecundity in both anopheline and culicine mosquitoes (DeJong et al., 2007; McCann et al., 2009; Petersen et al., 2018; Ramírez-Sánchez et al., 2023). Here, we observed that aging not only reduces mosquito fecundity, but also reduces oviposition success, delays egg laying, and reduces fertility. Moreover, at the oldest age, mosquitoes were sterile. In mosquitoes and other hematophagous dipterans, the aging-associated decline in reproduction is attributed to an increase in oxidative damage caused by reactive oxygen species (ROS) that are generated after a blood meal (DeJong et al., 2007; Diaz-Albiter et al., 2011; Michalkova et al., 2014). As flies age, there is a decrease in the transcription and activity of ROS detoxification enzymes, such as catalase and superoxide dismutase, hindering the ability to protect against oxidative stress (Klichko et al., 2004; DeJong et al., 2007; Estévez-Lao et al., 2020). Thus, aging-dependent oxidative damage is likely a major driver of the senescence of reproduction that we observed.

Prior to this study, the effects of warmer temperature and aging on mosquito reproduction—or the reproduction of any insect—had only been studied in isolation. Whether temperature and aging interactively shape reproduction remained unknown. Here, we discovered that warmer temperature accelerates reproductive senescence. In other words, when the temperature is warmer, the aging-dependent reduction in fecundity and fertility occurs earlier in life, and at the warmest temperature, mosquitoes are sterile regardless of age. Recently, warming-based acceleration of senescence has been observed in other facets of mosquito physiology: warmer temperature accelerates changes in body composition, worsens survival outcomes in sugar-fed mosquitoes, and alters the strength of the immune response (Barr et al., 2023; Barr et al., 2024; Martin and Hillyer, 2024; Martin et al., 2024; Barr et al., 2025). In another Ecdysozoan, the nematode *Caenorhabditis elegans*, warmer temperature also accelerates reproductive senescence (Klass, 1977; Hughes et al., 2007; Scharf et al., 2021). Thus, we hypothesize that warmer temperatures may accelerate reproductive senescence in Ecdysozoans in general, and this may be a feature of all poikilothermic ectotherms. A mechanistic understanding of warming-based reproductive senescence could be exploited to mitigate the spread of vector-borne diseases.

Mosquito reproduction and immunity are linked (Schwenke et al., 2016; Werling et al., 2019; Werling et al., 2024). Melanization, for example, is involved in immune defense, cuticular sclerotization, and egg chorion tanning (Tsao et al., 2015). We recently demonstrated that warmer temperature accelerates the aging-dependent weakening of the phenoloxidase-based melanization immune response (Martin and Hillyer, 2024). We suspect that the weakening of melanization transcends immunity and contributes to the decline in reproduction because warmer temperature and aging reduce the systemic level of active phenoloxidase (Martin and Hillyer, 2024). Therefore, less phenoloxidase is available for egg chorion tanning and hardening.

An unexpected finding in this study was that having access to an oviposition site increases the risk of dying. By separately examining the survival of mosquitoes that had access to an oviposition site and either laid eggs or failed to do so, we uncovered that the increased risk of death rests almost exclusively in mosquitoes that attempt to lay eggs but are unsuccessful. These mosquitoes had fully developed eggs in their abdomens, so we suspect that they died attempting to oviposit. This phenomenon occurs in humans (breeched babies) and birds (Abou-Zahr, 2022; Richmond and Ashworth, 2023). In the case of mosquitoes, failed oviposition results in eggs becoming stuck in the cloaca, which inhibits excretion through the same canal (Bradley, 2009). However, this may not be universal; a prior study demonstrated that survival is lower when mosquitoes are deprived of an oviposition site (Artis et al., 2014). We believe that the discrepancy between the two findings is driven by the stressors of warmer temperature and aging that were applied in our study.

Warmer temperature accelerates the aging-associated delay in oviposition. This may be a consequence of mosquitoes sensing sub-optimal oviposition conditions (Agyapong et al., 2014; Day, 2016; Asmare et al., 2017; Schoelitsz et al., 2020), like high water temperature. Egg hatching may also be delayed. Most eggs hatch within one to 2 days of being laid (Yaro et al., 2006; Impoinvil et al., 2007; Mazigo et al., 2019), but we were unable to measure hatching beyond the fourth day post-blood feeding out of concern that larval cannibalism would bias the readings. Regardless, eggs at 27°C and 31° have a similar time to hatching (Christiansen-Jucht et al., 2015), so it is unlikely that warmer temperature delays egg hatching.

To transmit disease, a mosquito must survive the extrinsic incubation period of the pathogen, which could be days or weeks (Bellan, 2010; Tjaden et al., 2013; Phillips et al., 2017). We observed that both warmer temperature and aging reduce mosquito survival after a blood meal. Our data match prior observations that warmer temperature reduces the survival of blood-fed mosquitoes (Aytekin et al., 2009; Olayemi et al., 2011; Faiman et al., 2017; Oliver and Brooke, 2017; Agyekum et al., 2021; Agyekum et al., 2022a). Moreover, because the mosquitoes in our study are reared from egg to adulthood at their respective temperature, we also captured carry-over effects from larval development, and larvae that develop at warmer temperatures were previously shown to have lower survival (Agyekum et al., 2021; Agyekum et al., 2022b). Similarly, our data support prior studies demonstrating that older mosquitoes survive fewer days after a blood meal than younger mosquitoes (Ryan et al., 2015; Petersen et al., 2018). We also discovered that warmer temperature accelerates the aging-dependent decline in survival after a blood meal, indicating that the effects of age are modified by temperature. In sugar-fed mosquitoes, warmer temperature accelerates the aging-dependent decline in survival (Barr et al., 2024). Taking a blood meal decreases survival when sugar is plentiful (Faiman et al., 2017), and our data support this: sugar-fed mosquitoes had median survival of 21 versus 8 days when at 27°C versus 32°C (Barr et al., 2024), whereas blood-fed mosquitoes had a median survival of 10 days versus 6 days when at 27°C versus 32°C. Thus, the warming-based acceleration of senescence is exacerbated in blood-fed mosquitoes.

The mosquitoes used in this study were reared at their respective experimental temperature. Therefore, the phenotypes observed could be due to the quality of the females, the quality of the males, or both. Our prior research has demonstrated that the quality of the females is compromised at warmer temperature (Barr et al., 2023). One intriguing possibility is that male-associated traits contribute to warmer temperature accelerating reproductive senescence. Prior to blood feeding, mating between a female and a male mosquito permanently alters the female’s transcriptional profile (Rogers et al., 2008). During mating, the male transfers the ecdysteroid, 20E, into the female (Pondeville et al., 2008), and although the female also produces 20E to regulate oogenesis, the male-transferred 20E signals the female to protect the sperm and increase egg development (Baldini et al., 2013; Gabrieli et al., 2014; Shaw et al., 2014). In our experiments, the mosquitoes had mated because the cages were roughly 50% female and 50% male for 3 days, and (i) the majority of adult *A. gambiae* mate after 24 h post emergence (Takken et al., 2024), and (ii) a single male can mate with about 8 females (Okanda et al., 2002). It is unknown how temperature affects the reproductive physiology of male mosquitoes, but warmer temperature negatively impacts sperm abundance in male fruit flies (Gandara and Drummond-Barbosa, 2023). It is possible that temperature-related changes in male physiology can be exploited to curtail mosquito populations. Conversely, if the males are unable to copulate in warm environments, then warmer temperatures may prevent the success of control strategies such as the sterile insect technique.

In summary, warmer temperature accelerates reproductive senescence in the mosquito, and at the warmest temperature of 32°C, mosquitoes are sterile. Therefore, climate change resulting in warmer global temperatures will have consequential effects on mosquito population dynamics and disease transmission outcomes, including the potential for significantly reduced mosquito populations in geographical areas that become too warm.

## Data Availability

The original contributions presented in the study are included in the article/Supplementary Material, further inquiries can be directed to the corresponding author.
