# Supplementary material for: Warmer temperature accelerates reproductive senescence in mosquitoes
Source: Front Physiol. 2025 Jul 2;16:1610310. doi: 10.3389/fphys.2025.1610310 (PMC12265498; doi:10.3389/fphys.2025.1610310)
Supplement: Supplementary file 1 [file DataSheet2.pdf]

# *Warmer temperature accelerates reproductive senescence in mosquitoes*

*Lindsay E. Martin et al.*

# *Supplementary Material*

## 1 Supplementary Data

**Supplementary Data 1. Raw data.** (xlsx file)

**Supplementary Data 2. Processed data.** (xlsx file)

**Supplementary Code 1. R code.** (PDF file)

## 2 Supplementary Figures

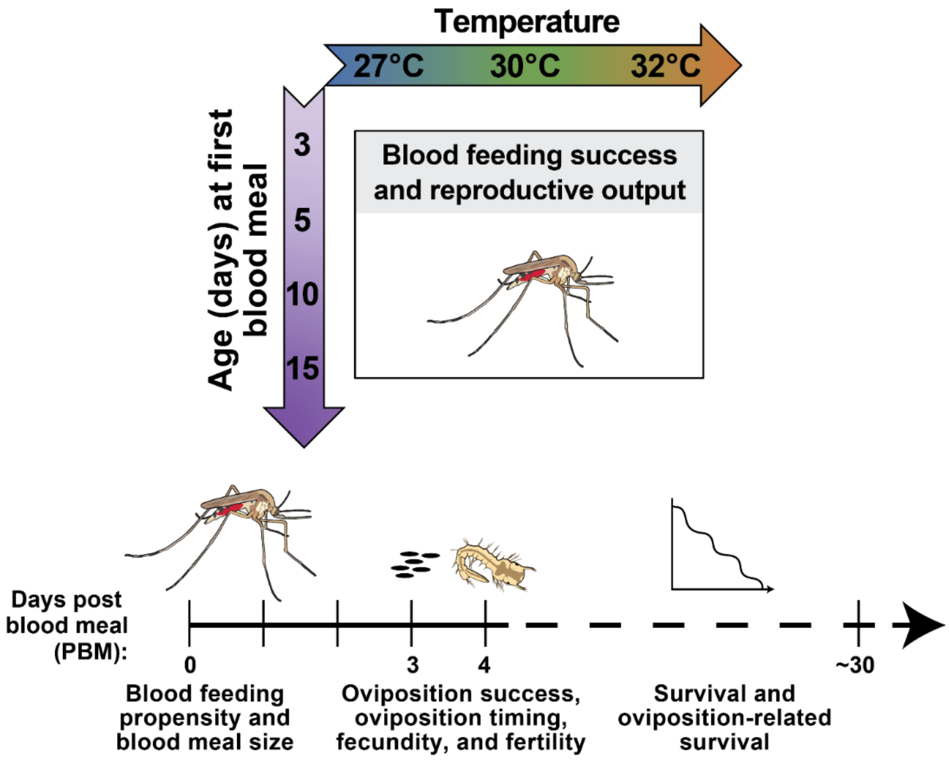


**Supplementary Figure 1. Experimental overview to determine the effects of warmer temperature, aging, and their interaction on mosquito blood feeding, reproduction, and survival.**
