# Supplementary material for: Warmer temperature accelerates reproductive senescence in mosquitoes
Source: Front Physiol. 2025 Jul 2;16:1610310. doi: 10.3389/fphys.2025.1610310 (PMC12265498; doi:10.3389/fphys.2025.1610310)
Supplement: Supplementary file 3 [file DataSheet1.pdf]

Supplementary Code 1

Lindsay E. Martin

2025-03-14

Supplementary Code 1

Manuscript: Warmer temperature accelerates reproductive senescence in mosquitoes

Authors: Lindsay E. Martin, Tania Y. Estévez-Lao, Tobias McCabe, & Julián F. Hillyer

# Corresponding author email: julian.hillyer@vanderbilt.edu (mailto:julian.hillyer@vanderbilt.edu)

Code written by Lindsay E. Martin

Code written in R version 4.4.1 (2024-06-14 ucrt)

See Supplementary Data 1 for Raw Data. See Supplementary Data 2 for Processed Data output.

Code for Figure 1: blood feeding propensity

Import data and clean it up.

```
# clear existing workspace
rm(list = ls(all = TRUE))
graphics.off()
shell("cls")

#set wd to your project folder
getwd() #check working directory
```

```
## [1] "C:/Users/linzm/OneDrive - Vanderbilt/Hillyer_Lab/Blood_feeding_project/Bloodfeeding"
```

```
#load libraries needed:
library(readxl)
library(writexl)
library(ggplot2)
library(dplyr)
```

```
##
## Attaching package: 'dplyr'
```

```
## The following objects are masked from 'package:stats':
##
##     filter, lag
```

```
## The following objects are masked from 'package:base':
##
##     intersect, setdiff, setequal, union
```

```r
library(tidyverse)
```

```
## ── Attaching core tidyverse packages ──────────────────────── tidyverse 2.0.0 ──
## ✔ forcats   1.0.0     ✔ stringr   1.5.1
## ✔ lubridate 1.9.3     ✔ tibble    3.2.1
## ✔ purrr     1.0.2     ✔ tidyr     1.3.1
## ✔ readr     2.1.5
```

```
## ── Conflicts ────────────────────────────────────────── tidyverse_conflicts() ──
## ✖ dplyr::filter() masks stats::filter()
## ✖ dplyr::lag()    masks stats::lag()
## ℹ Use the conflicted package (<http://conflicted.r-lib.org/>) to force all conflicts to become errors
```

```r
library(rstatix)
```

```
##
## Attaching package: 'rstatix'
##
## The following object is masked from 'package:stats':
##
##     filter
```

```r
library(car)
```

```
## Loading required package: carData
##
## Attaching package: 'car'
##
## The following object is masked from 'package:purrr':
##
##     some
##
## The following object is masked from 'package:dplyr':
##
##     recode
```

```r
library(ggpubr)
library(emmeans)
```

```
## Welcome to emmeans.
## Caution: You lose important information if you filter this package's results.
## See '? untidy'
```

```
library(lmtest)
```

```
## Loading required package: zoo
##
## Attaching package: 'zoo'
##
## The following objects are masked from 'package:base':
##
##     as.Date, as.Date.numeric
```

```
library(glmmTMB)
library(DHARMa)
```

```
## This is DHARMa 0.4.6. For overview type '?DHARMa'. For recent changes, type news(package = 'DHARMa')
```

```
#######################################################
#import the data and clean it up:
#import the data:

Proportion_data <- read_xlsx("SupplementaryData1_RawData.xlsx",
                             sheet = "Fig1")
Proportion_data <- as.data.frame(Proportion_data)
str(Proportion_data)
```

```
## 'data.frame':    59 obs. of  13 variables:
##  $ Overall_ID   : num  1 2 3 4 5 6 7 8 9 10 ...
##  $ Temperature  : num  27 27 27 32 32 27 27 32 30 32 ...
##  $ Age          : num  3 3 3 3 3 3 3 3 3 3 ...
##  $ BM1_Date     : POSIXct, format: "2024-02-06" "2024-02-07" ...
##  $ Age_of_BM    : num  3 3 3 3 3 3 3 3 3 3 ...
##  $ BM_number    : num  1 1 1 1 1 1 1 1 1 1 ...
##  $ Trial_number : num  1 2 2 1 1 3 3 2 1 3 ...
##  $ BF           : num  170 79 56 28 44 110 127 62 106 23 ...
##  $ NCF          : num  25 5 4 40 43 59 35 219 19 88 ...
##  $ Total        : num  195 84 60 68 87 169 162 281 125 111 ...
##  $ Percentage_BF: num  87.2 94 93.3 41.2 50.6 ...
##  $ BF_ID        : num  1 2 3 7 8 33 34 40 41 42 ...
##  $ Blood_date   : POSIXct, format: "2024-02-02" "2024-02-02" ...
```

```
head(Proportion_data)
```

```
##   Overall_ID Temperature Age   BM1_Date Age_of_BM BM_number Trial_number  BF
## 1          1          27   3 2024-02-06         3         1            1 170
## 2          2          27   3 2024-02-07         3         1            2  79
## 3          3          27   3 2024-02-07         3         1            2  56
## 4          4          32   3 2024-02-13         3         1            1  28
## 5          5          32   3 2024-02-13         3         1            1  44
## 6          6          27   3 2024-04-16         3         1            3 110
##   NCF Total Percentage_BF BF_ID Blood_date
## 1  25   195      87.17949     1 2024-02-02
## 2   5    84      94.04762     2 2024-02-02
## 3   4    60      93.33333     3 2024-02-02
## 4  40    68      41.17647     7 2024-02-02
## 5  43    87      50.57471     8 2024-02-02
## 6  59   169      65.08876    33 2024-03-18
```

```
#variables of interest:
Proportion_data$Temperature <- as.factor(Proportion_data$Temperature)
Proportion_data$Age <- as.factor(Proportion_data$Age)
Proportion_data$Age_of_BM <- as.factor(Proportion_data$Age_of_BM)

hist(Proportion_data$Percentage_BF)

library(rstatix)
identify_outliers(
  data = Proportion_data,
  variable = "Percentage_BF"
)
```

```
##  [1] Overall_ID    Temperature   Age           BM1_Date      Age_of_BM    
##  [6] BM_number     Trial_number  BF            NCF           Total        
## [11] Percentage_BF BF_ID         Blood_date    is.outlier    is.extreme   
## <0 rows> (or 0-length row.names)
```

```
#no outliers detected.
```

Calculate summary stats

```
#summary stats:
Proportion_summary1 <- Proportion_data %>%
  group_by(Temperature,Age,BM_number) %>%
  summarise(mean_percentage = mean(Percentage_BF),
            median_percentage = median(Percentage_BF),
            sd_percentage = sd(Percentage_BF),
            n_percentage = n(),
            SE_percentage = sd(Percentage_BF)/sqrt(n()))
```

```
## `summarise()` has grouped output by 'Temperature', 'Age'. You can override
## using the `.groups` argument.
```

```
write_xlsx(Proportion_summary1, "./Proportion/Proportion_bloodfed_summary1.xlsx")

Proportion_summary1_firstBM <- subset(Proportion_summary1, BM_number == 1)
Proportion_data_firstBM <- subset(Proportion_data, BM_number == 1)
```

Plot all data

```
#labels:
Proportion_data_firstBM$Age <- factor(Proportion_data_firstBM$Age,
                                      labels = c("3","5","10","15"))

Proportion_data_firstBM$Temperature <- factor(Proportion_data_firstBM$Temperature,
                                              labels = c("27˚C","30˚C","32˚C"))

Proportion_summary1_firstBM$Age <- factor(Proportion_summary1_firstBM$Age,
                                          labels = c("3","5","10","15"))

Proportion_summary1_firstBM$Temperature <- factor(Proportion_summary1_firstBM$Temperature,
                                                  labels = c("27˚C","30˚C","32˚C"))




#Plot:
firstBM_proportionbloodfed_agewithintemp <- Proportion_summary1_firstBM %>%
  ggplot(aes(x=Age,y=mean_percentage,group=Age))+
  geom_bar(aes(fill=Age),
           stat = "identity",
           position = position_dodge(1),
           width = 0.8) +
  scale_shape_identity(guide="legend")+
  facet_grid(~Temperature)+
  geom_errorbar(aes(ymin=mean_percentage - SE_percentage,
                    ymax=mean_percentage + SE_percentage),
                width=0.8,position=position_dodge(0.9),
                color="black")+
  ylab(expression("% females that took a bloodmeal"))+
  xlab("Adult age (days)") +
  theme_pubr()+
  theme(legend.position = "none")+
  geom_jitter(data=Proportion_data_firstBM, aes(x=Age,y=Percentage_BF),#color=ZOI_italic$Technical_Rep,
              position = "jitter",size=1)+
  scale_y_continuous(limits=c(0,100))+
  scale_fill_manual(values= c("#DCD1E9","#BAA4D3","#9776BE","#7549A8"))+
  theme(panel.background = element_rect(fill = NA, color = "black"))+
  theme(panel.spacing = unit(0.5, "lines"))
firstBM_proportionbloodfed_agewithintemp
```

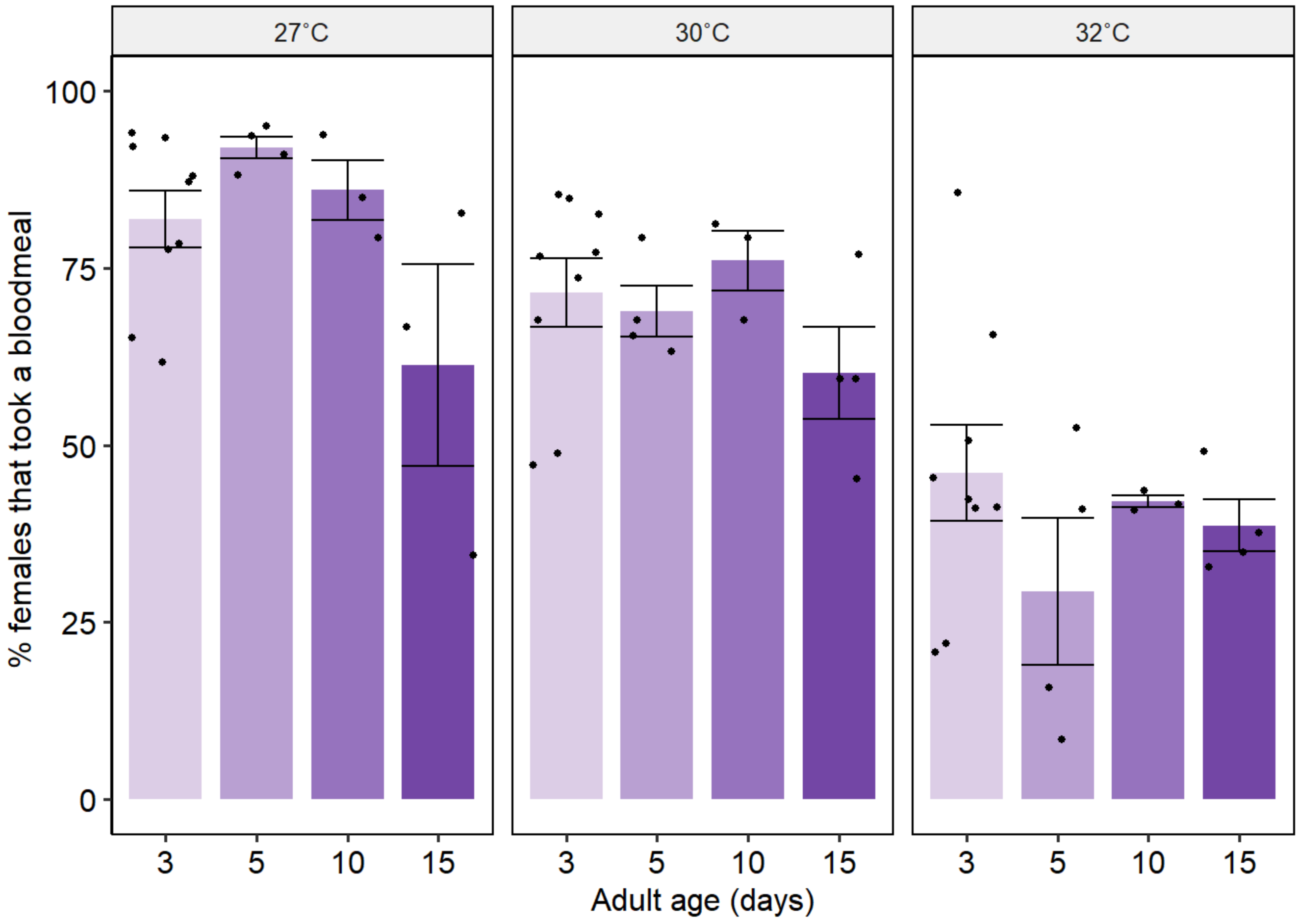
27°C
30°C
32°C
% females that took a bloodmeal
100
75
50
25
0
3
5
10
15
Adult age (days)

```
ggsave("Proportion/firstBM_proportionbloodfed_agewithintemp.png",plot=firstBM_proportionbloodfed_agewithint
emp,width = 5, height = 4, units = "in", dpi = 600)
ggsave("Proportion/firstBM_proportionbloodfed_agewithintemp.pdf",plot=firstBM_proportionbloodfed_agewithint
emp,width = 5, height = 4, units = "in", dpi = 600)


Proportion_data_firstBM$Age <- factor(Proportion_data_firstBM$Age,
                                      labels = c("3 days","5 days","10 days","15 days"))

Proportion_data_firstBM$Temperature <- factor(Proportion_data_firstBM$Temperature,
                                              labels = c("27","30","32"))

Proportion_summary1_firstBM$Age <- factor(Proportion_summary1_firstBM$Age,
                                          labels = c("3 days","5 days","10 days","15 days"))

Proportion_summary1_firstBM$Temperature <- factor(Proportion_summary1_firstBM$Temperature,
                                                  labels = c("27","30","32"))

firstBM_proportionbloodfed_tempwithinage <- Proportion_summary1_firstBM %>%
  ggplot(aes(x=Temperature,y=mean_percentage,group=Temperature))+
  geom_bar(aes(fill=Temperature),
           stat = "identity",
           position = position_dodge(1),
           width = 0.8) +
  scale_shape_identity(guide="legend")+
  facet_grid(~Age)+
  geom_errorbar(aes(ymin=mean_percentage - SE_percentage,
                    ymax=mean_percentage + SE_percentage),
                width=0.8,position=position_dodge(0.9),
                color="black")+
  ylab(expression("% females that took a bloodmeal"))+
  xlab("Temperature (˚C)") +
  theme_pubr()+
  theme(legend.position = "none")+
  scale_y_continuous(limits=c(0,100))+
  geom_jitter(data=Proportion_data_firstBM, aes(x=Temperature,y=Percentage_BF),#color=ZOI_italic$Technical_
Rep,
              position = "jitter",size=1)+
  scale_fill_manual(values= c("#4D6FAE","#6F9F51", "#CC763B"))+
  theme(panel.background = element_rect(fill = NA, color = "black"))+
  theme(panel.spacing = unit(0.5, "lines"))
firstBM_proportionbloodfed_tempwithinage
```

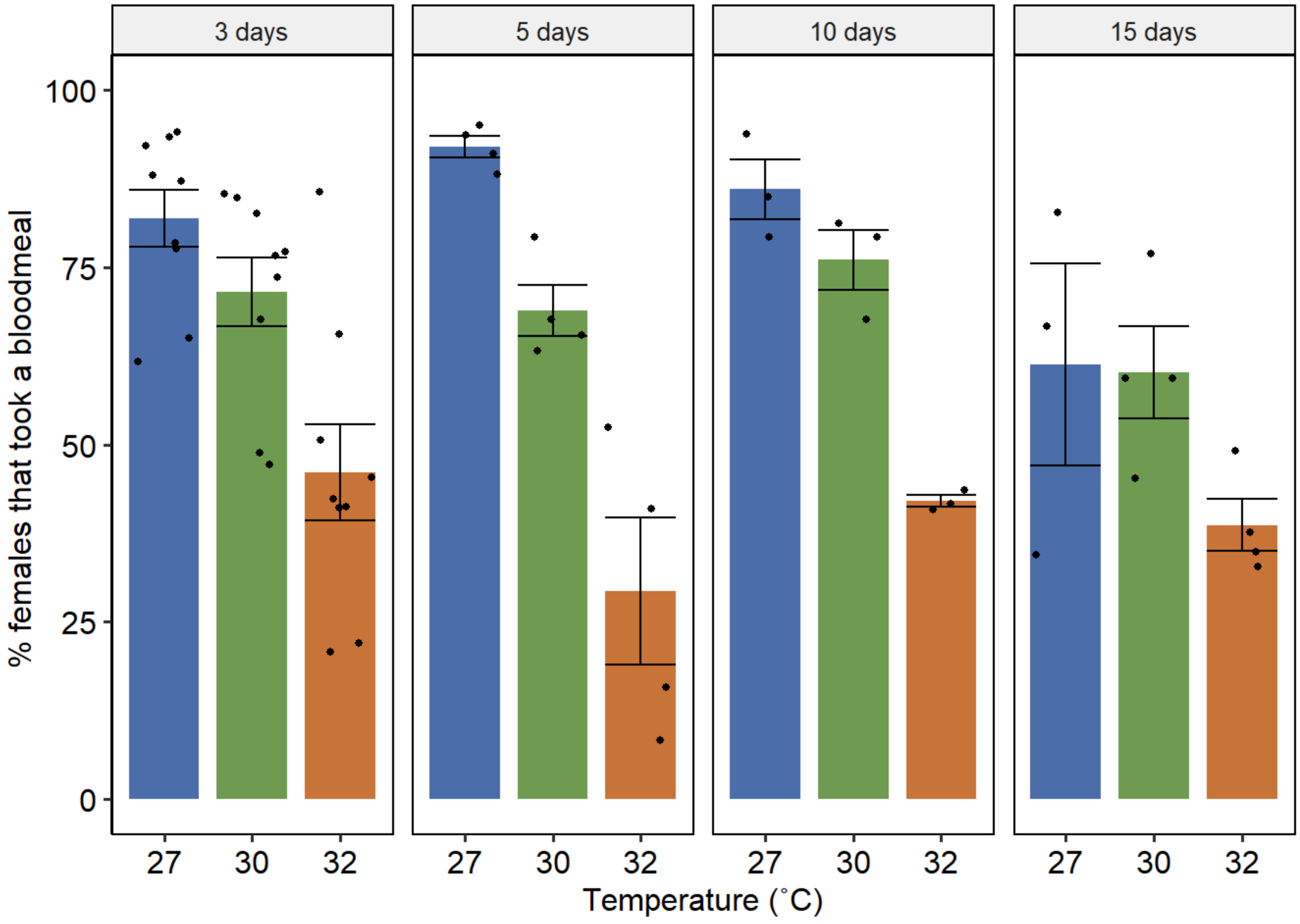
3 days
5 days
10 days
15 days
% females that took a bloodmeal
100
75
50
25
0
27
30
32
Temperature (°C)

```
ggsave("Proportion/firstBM_proportionbloodfed_tempwithinage.png",plot=firstBM_proportionbloodfed_tempwithin
age,width = 5, height = 4, units = "in", dpi = 600)
ggsave("Proportion/firstBM_proportionbloodfed_tempwithinage.pdf",plot=firstBM_proportionbloodfed_tempwithin
age,width = 5, height = 4, units = "in", dpi = 600)


proportion_raw_interaction <- Proportion_summary1_firstBM %>%
  ggplot()+
  aes(x = Age, group = Temperature, y = mean_percentage) +
  #stat_summary(fun.y = mean, geom = "point",shape="Temperature") +
  geom_point(aes(shape=Temperature),size=3)+
  # scale_size_manual(values=c(2,5,10)) +
  #stat_summary(fun.y = mean, geom = "line")+
  geom_line(aes(linetype = Temperature),linewidth=0.6)+
  theme_pubr()+
  scale_shape(labels=c(27,30,32))+
  scale_linetype(labels=c(27,30,32))+
  guides(shape = guide_legend(title = "Temperature (˚C)"),
         linetype = guide_legend(title = "Temperature (˚C)"))+
  xlab("Adult Age (days)") +
  theme(legend.position = "right")+
  ylab(expression("% of mosquitoes taking 1st BM"))+
  scale_y_continuous(limits=c(25,100)) +
  #scale_y_continuous(labels = function(x) paste0(x*100)) +
  scale_x_discrete(labels=c(3,5,10,15))+
  theme(panel.background = element_rect(fill = NA, color = "black"))+
  theme(panel.spacing = unit(0.6, "lines"))


ggsave("Proportion/BM1_proportion_raw_interaction.png",plot=proportion_raw_interaction,width = 5.5, height
= 4, units = "in", dpi = 600)
ggsave("Proportion/BM1_proportion_raw_interaction.pdf",plot=proportion_raw_interaction,width = 5.5, height
= 4, units = "in", dpi = 600)
```

Plot main effects

```
#main effects:
Proportion_summary1_firstBM_TEMP <- Proportion_data_firstBM %>%
  group_by(Temperature) %>%
  summarise(mean_percentage = mean(Percentage_BF),
            median_percentage = median(Percentage_BF),
            sd_percentage = sd(Percentage_BF),
            n_percentage = n(),
            SE_percentage = sd(Percentage_BF)/sqrt(n()))

write_xlsx(Proportion_summary1_firstBM_TEMP, "Proportion/Proportion_bloodfed_summary1_TEMP.xlsx")



Proportion_summary1_firstBM_AGE <- Proportion_data_firstBM %>%
  group_by(Age) %>%
  summarise(mean_percentage = mean(Percentage_BF),
            median_percentage = median(Percentage_BF),
            sd_percentage = sd(Percentage_BF),
            n_percentage = n(),
            SE_percentage = sd(Percentage_BF)/sqrt(n()))

write_xlsx(Proportion_summary1_firstBM_AGE, "Proportion/Proportion_bloodfed_summary1_AGE.xlsx")



#Plot:
Proportion_data_firstBM$Age <- factor(Proportion_data_firstBM$Age,
                                      labels = c("3","5","10","15"))

Proportion_summary1_firstBM_AGE$Age <- factor(Proportion_summary1_firstBM_AGE$Age,
                                              labels = c("3","5","10","15"))

firstBM_proportionbloodfed_AGEonly <- Proportion_summary1_firstBM_AGE %>%
  ggplot(aes(x=Age,y=mean_percentage,group=Age))+
  geom_bar(aes(fill=Age),
           stat = "identity",
           position = position_dodge(1),
           width = 0.8) +
  scale_shape_identity(guide="legend")+
  #facet_grid(~Temperature)+
  geom_errorbar(aes(ymin=mean_percentage - SE_percentage,
                    ymax=mean_percentage + SE_percentage),
                width=0.8,position=position_dodge(0.9),
                color="black")+
  ylab(expression("% females that took a bloodmeal"))+
  xlab("Adult age (days)") +
  theme_pubr()+
  theme(legend.position = "none")+
  geom_jitter(data=Proportion_data_firstBM, aes(x=Age,y=Percentage_BF),#color=ZOI_italic$Technical_Rep,
             position = "jitter",size=1)+
  scale_y_continuous(limits=c(0,100))+
  scale_fill_manual(values= c("#DCD1E9","#BAA4D3","#9776BE","#7549A8"))+
  theme(panel.background = element_rect(fill = NA, color = "black"))+
  theme(panel.spacing = unit(0.5, "lines"))
firstBM_proportionbloodfed_AGEonly
```

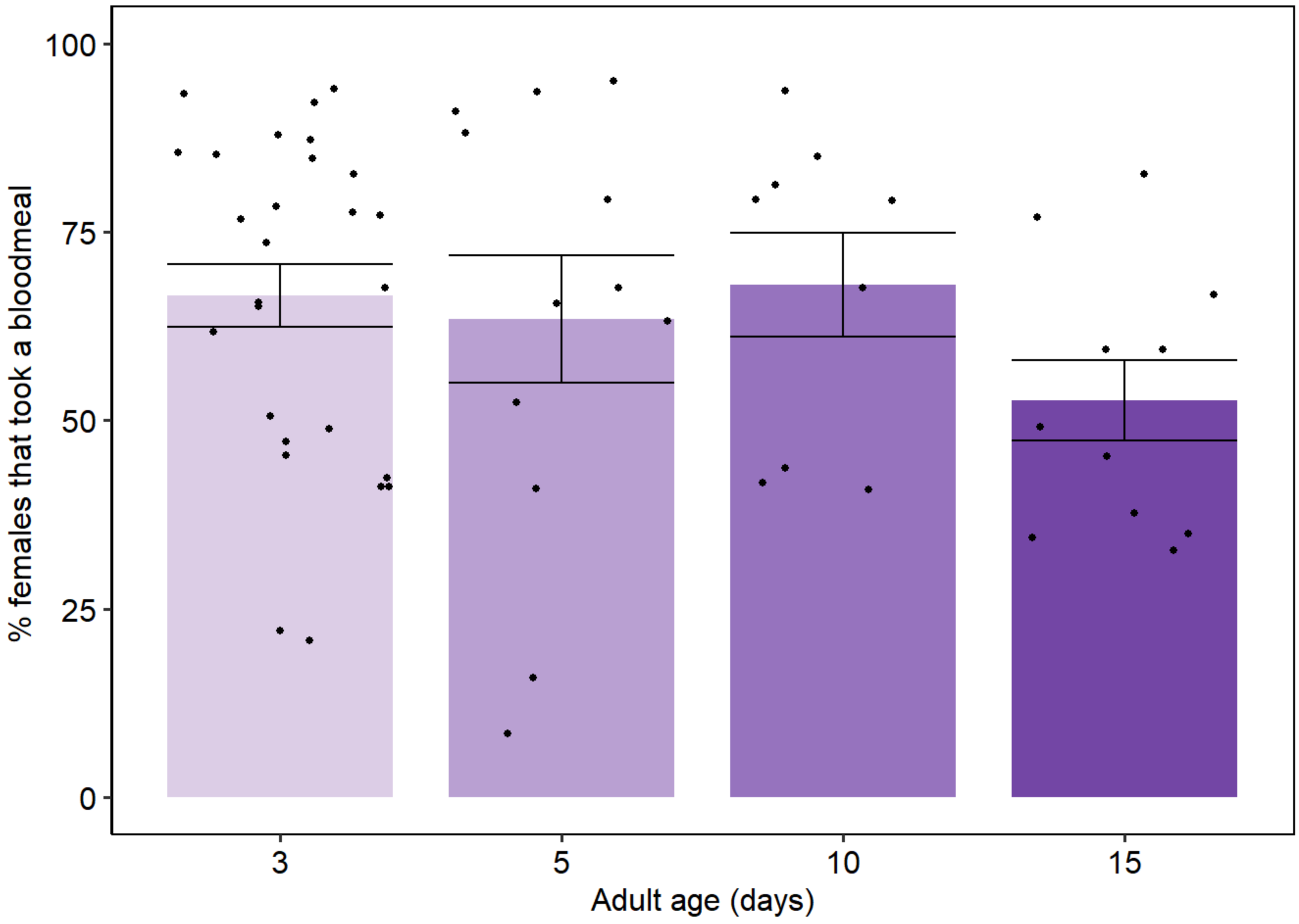


```
ggsave("Proportion/firstBM_proportionbloodfed_AGEonly.png",plot=firstBM_proportionbloodfed_AGEonly,width =
4, height = 4, units = "in", dpi = 600)
ggsave("Proportion/firstBM_proportionbloodfed_AGEonly.pdf",plot=firstBM_proportionbloodfed_AGEonly,width =
4, height = 4, units = "in", dpi = 600)


firstBM_proportionbloodfed_TEMPonly <- Proportion_summary1_firstBM_TEMP %>%
  ggplot(aes(x=Temperature,y=mean_percentage,group=Temperature))+
  geom_bar(aes(fill=Temperature),
           stat = "identity",
           position = position_dodge(1),
           width = 0.8) +
  scale_shape_identity(guide="legend")+
  #facet_grid(~Age)+
  geom_errorbar(aes(ymin=mean_percentage - SE_percentage,
                    ymax=mean_percentage + SE_percentage),
                width=0.8,position=position_dodge(0.9),
                color="black")+
  ylab(expression("% females that took a bloodmeal"))+
  xlab("Temperature (˚C)") +
  theme_pubr()+
  theme(legend.position = "none")+
  scale_y_continuous(limits=c(0,100))+
  geom_jitter(data=Proportion_data, aes(x=Temperature,y=Percentage_BF),#color=ZOI_italic$Technical_Rep,
              position = "jitter",size=1)+
  scale_fill_manual(values= c("#4D6FAE","#6F9F51", "#CC763B"))+
  theme(panel.background = element_rect(fill = NA, color = "black"))+
  theme(panel.spacing = unit(0.5, "lines"))
firstBM_proportionbloodfed_TEMPonly
```

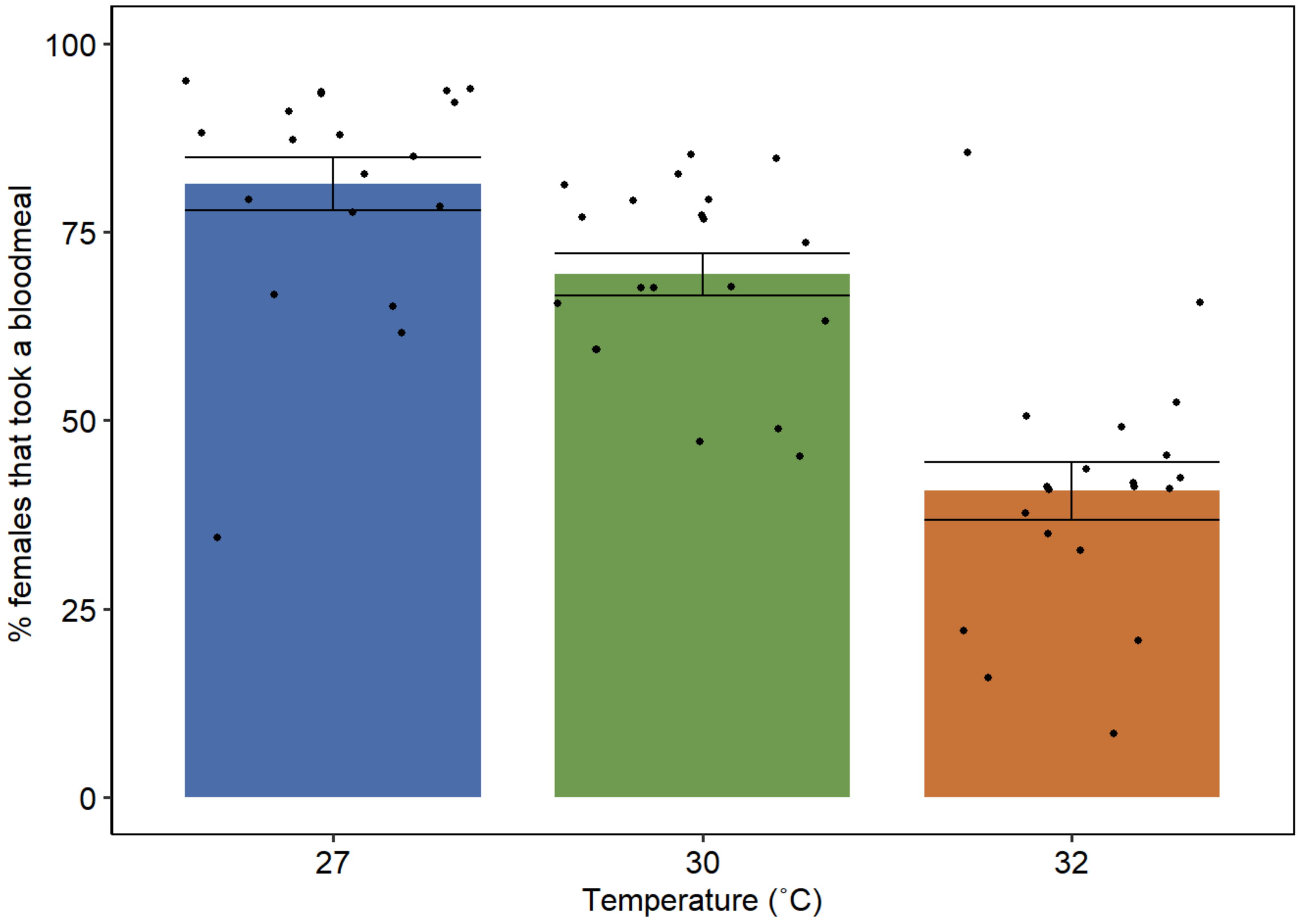


```
ggsave("Proportion/firstBM_proportionbloodfed_TEMPonly.png",plot=firstBM_proportionbloodfed_TEMPonly,width
= 4, height = 4, units = "in", dpi = 600)
ggsave("Proportion/firstBM_proportionbloodfed_TEMPonly.pdf",plot=firstBM_proportionbloodfed_TEMPonly,width
= 4, height = 4, units = "in", dpi = 600)
```

Analyze Fig 1 data.

```
#go back to numeric numbers w/o labels:
Proportion_data_firstBM$Age <- factor(Proportion_data_firstBM$Age,
                                      labels = c("3","5","10","15"))

Proportion_data_firstBM$Temperature <- factor(Proportion_data_firstBM$Temperature,
                                              labels = c("27","30","32"))
Proportion_data_firstBM$Age <- as.factor(Proportion_data_firstBM$Age)
Proportion_data_firstBM$Temperature <- as.factor(Proportion_data_firstBM$Temperature)

ggplot(data = Proportion_data_firstBM, aes(x = Percentage_BF))+
  geom_density(color = "#FF6885", alpha = 0.6, bw = 1.5)+
  ylab("Density")+
  xlab("Percentage bf")+
  theme_pubr()
```

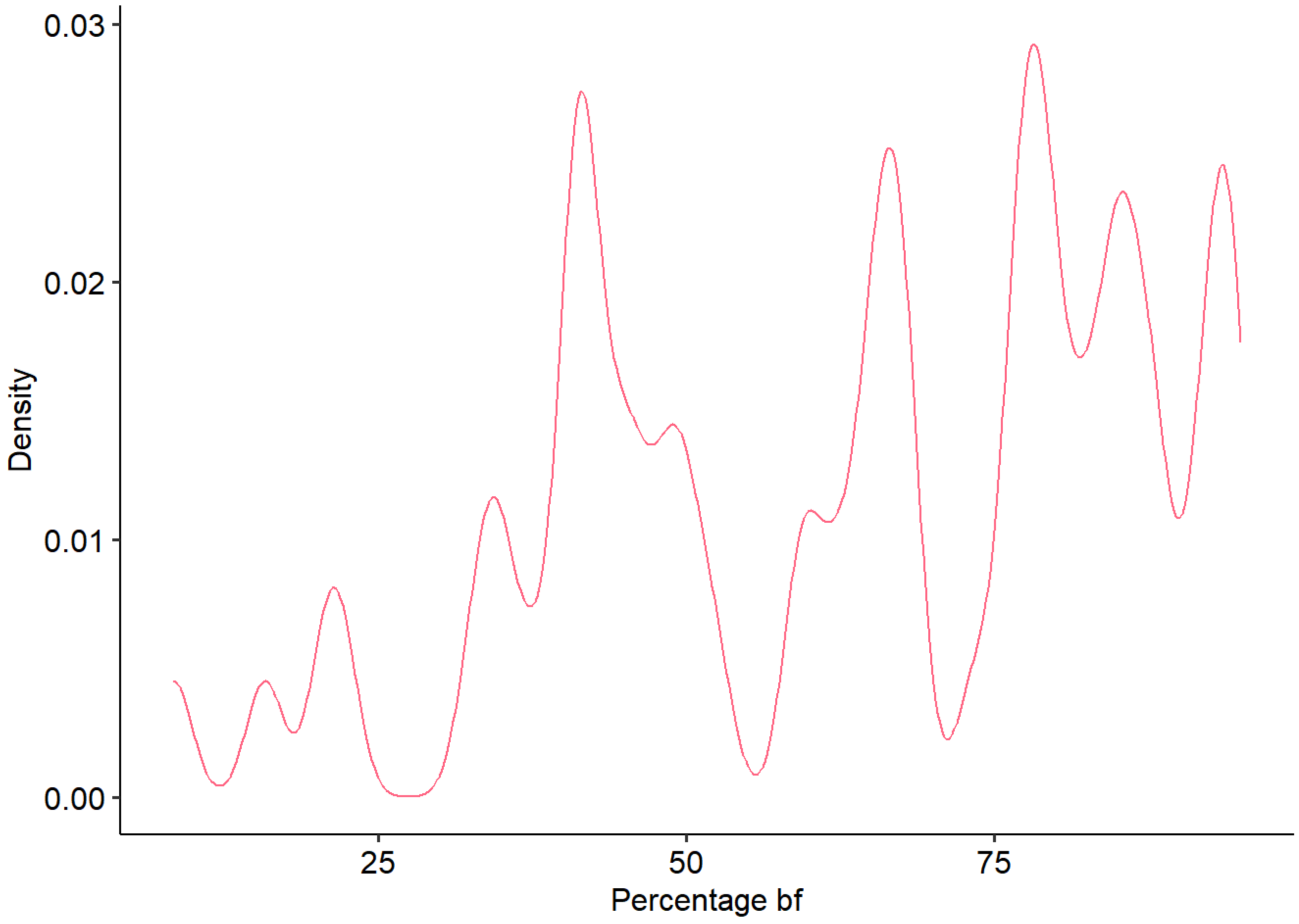


```
shapiro.test(Proportion_data_firstBM$Percentage_BF)
```

```
##
##  Shapiro-Wilk normality test
##
## data:  Proportion_data_firstBM$Percentage_BF
## W = 0.94155, p-value = 0.006939
```

```
str(Proportion_data_firstBM$Percentage_BF)
```

```
##  num [1:59] 87.2 94 93.3 41.2 50.6 ...
```

```
Proportion_data_firstBM$proportion <- Proportion_data_firstBM$Percentage_BF / 100

#model selection:
library(glmmTMB)
#check different models
model.Proportion_firstBM_1 = glmmTMB(proportion ~ Temperature+Age, data= Proportion_data_firstBM,family=bet
a_family(link="logit")) #equals model 1
model.Proportion_firstBM_2 = glmmTMB(proportion ~ Temperature+Age+(1|Trial_number), data= Proportion_data_f
irstBM,family=beta_family(link="logit")) #equals model 1
model.Proportion_firstBM_3 = glmmTMB(proportion ~ Temperature*Age, data= Proportion_data_firstBM,family=bet
a_family(link="logit")) #equals model 1
model.Proportion_firstBM_4 = glmmTMB(proportion ~ Temperature*Age+(1|Trial_number), data= Proportion_data_f
irstBM,family=beta_family(link="logit")) #equals model 1

library(lmtest)
summary(model.Proportion_firstBM_1)
```

```
##  Family: beta  ( logit )
## Formula:          proportion ~ Temperature + Age
## Data: Proportion_data_firstBM
##
##      AIC      BIC   logLik deviance df.resid
##    -61.3    -46.8     37.7    -75.3       52
##
##
## Dispersion parameter for beta family ():  9.8
##
## Conditional model:
##               Estimate Std. Error z value Pr(>|z|)
## (Intercept)     1.5952     0.1992   8.008 1.16e-15 ***
## Temperature30  -0.6674     0.2220  -3.006  0.00265 **
## Temperature32  -1.8400     0.2211  -8.322  < 2e-16 ***
## Age5           -0.2007     0.2189  -0.917  0.35923
## Age10           0.0334     0.2555   0.131  0.89600
## Age15          -0.5453     0.2435  -2.240  0.02510 *
## ---
## Signif. codes:  0 '***' 0.001 '**' 0.01 '*' 0.05 '.' 0.1 ' ' 1
```

```
summary(model.Proportion_firstBM_2)
```

```
##  Family: beta  ( logit )
## Formula:          proportion ~ Temperature + Age + (1 | Trial_number)
## Data: Proportion_data_firstBM
##
##      AIC      BIC   logLik deviance df.resid
##    -59.3    -42.7     37.7    -75.3       51
##
## Random effects:
##
## Conditional model:
##  Groups       Name        Variance  Std.Dev.
##  Trial_number (Intercept) 1.825e-10 1.351e-05
## Number of obs: 59, groups:  Trial_number, 5
##
## Dispersion parameter for beta family ():  9.8
##
## Conditional model:
##               Estimate Std. Error z value Pr(>|z|)
## (Intercept)     1.5952     0.1992   8.008 1.16e-15 ***
## Temperature30  -0.6674     0.2220  -3.006  0.00265 **
## Temperature32  -1.8400     0.2211  -8.322  < 2e-16 ***
## Age5           -0.2007     0.2189  -0.917  0.35923
## Age10           0.0334     0.2555   0.131  0.89599
## Age15          -0.5453     0.2435  -2.240  0.02510 *
## ---
## Signif. codes:  0 '***' 0.001 '**' 0.01 '*' 0.05 '.' 0.1 ' ' 1
```

```
summary(model.Proportion_firstBM_3)
```

```
##  Family: beta  ( logit )
## Formula:          proportion ~ Temperature * Age
## Data: Proportion_data_firstBM
##
##      AIC      BIC   logLik deviance df.resid
##    -60.5    -33.5     43.3    -86.5       46
##
##
## Dispersion parameter for beta family (): 11.9
##
## Conditional model:
##                      Estimate Std. Error z value Pr(>|z|)
## (Intercept)           1.51271    0.22877   6.612 3.78e-11 ***
## Temperature30        -0.60081    0.30319  -1.982   0.0475 *
## Temperature32        -1.65145    0.29480  -5.602 2.12e-08 ***
## Age5                  0.59001    0.45089   1.309   0.1907
## Age10                 0.17370    0.46711   0.372   0.7100
## Age15                -1.01942    0.40000  -2.549   0.0108 *
## Temperature30:Age5   -0.76035    0.57434  -1.324   0.1855
## Temperature32:Age5   -1.43263    0.57583  -2.488   0.0128 *
## Temperature30:Age10 -0.02072    0.62148  -0.033   0.9734
## Temperature32:Age10 -0.33085    0.59784  -0.553   0.5800
## Temperature30:Age15  0.51284    0.52894   0.970   0.3323
## Temperature32:Age15  0.72808    0.52381   1.390   0.1645
## ---
## Signif. codes:  0 '***' 0.001 '**' 0.01 '*' 0.05 '.' 0.1 ' ' 1
```

```
summary(model.Proportion_firstBM_4)
```

```
##  Family: beta  ( logit )
## Formula:          proportion ~ Temperature * Age + (1 | Trial_number)
## Data: Proportion_data_firstBM
##
##      AIC      BIC   logLik deviance df.resid
##    -58.5    -29.5     43.3    -86.5       45
##
## Random effects:
##
## Conditional model:
##  Groups       Name        Variance  Std.Dev.
##  Trial_number (Intercept) 1.298e-10 1.139e-05
## Number of obs: 59, groups:  Trial_number, 5
##
## Dispersion parameter for beta family (): 11.9
##
## Conditional model:
##                       Estimate Std. Error z value Pr(>|z|)
## (Intercept)            1.51271    0.22877   6.612 3.78e-11 ***
## Temperature30         -0.60081    0.30319  -1.982   0.0475 *
## Temperature32         -1.65145    0.29480  -5.602 2.12e-08 ***
## Age5                   0.59001    0.45089   1.309   0.1907
## Age10                  0.17369    0.46711   0.372   0.7100
## Age15                 -1.01942    0.40000  -2.549   0.0108 *
## Temperature30:Age5  -0.76036    0.57434  -1.324   0.1855
## Temperature32:Age5  -1.43263    0.57583  -2.488   0.0128 *
## Temperature30:Age10 -0.02072    0.62148  -0.033   0.9734
## Temperature32:Age10 -0.33085    0.59784  -0.553   0.5800
## Temperature30:Age15  0.51284    0.52894   0.970   0.3323
## Temperature32:Age15  0.72808    0.52381   1.390   0.1645
## ---
## Signif. codes:  0 '***' 0.001 '**' 0.01 '*' 0.05 '.' 0.1 ' ' 1
```

```
lrtest(model.Proportion_firstBM_1,model.Proportion_firstBM_2) #bio rep does not matter
```

```
## Likelihood ratio test
##
## Model 1: proportion ~ Temperature + Age
## Model 2: proportion ~ Temperature + Age + (1 | Trial_number)
##   #Df LogLik Df Chisq Pr(>Chisq)
## 1   7 37.655
## 2   8 37.655  1     0     0.9999
```

```
lrtest(model.Proportion_firstBM_1,model.Proportion_firstBM_3)#interaction matters
```

```
## Likelihood ratio test
##
## Model 1: proportion ~ Temperature + Age
## Model 2: proportion ~ Temperature * Age
##   #Df LogLik Df Chisq Pr(>Chisq)
## 1   7 37.655
## 2  13 43.270  6 11.23    0.08152 .
## ---
## Signif. codes:  0 '***' 0.001 '**' 0.01 '*' 0.05 '.' 0.1 ' ' 1
```

```
lrtest(model.Proportion_firstBM_3,model.Proportion_firstBM_4) #bio rep does not matter
```

```
## Likelihood ratio test
##
## Model 1: proportion ~ Temperature * Age
## Model 2: proportion ~ Temperature * Age + (1 | Trial_number)
##   #Df LogLik Df Chisq Pr(>Chisq)
## 1  13  43.27
## 2  14  43.27  1     0     0.9999
```

```
AIC(model.Proportion_firstBM_1,model.Proportion_firstBM_2,model.Proportion_firstBM_3,model.Proportion_first
BM_4)
```

```
##                            df       AIC
## model.Proportion_firstBM_1  7 -61.31090
## model.Proportion_firstBM_2  8 -59.31090
## model.Proportion_firstBM_3 13 -60.54098
## model.Proportion_firstBM_4 14 -58.54098
```

```
BIC(model.Proportion_firstBM_1,model.Proportion_firstBM_2,model.Proportion_firstBM_3,model.Proportion_first
BM_4)
```

```
##                            df       BIC
## model.Proportion_firstBM_1  7 -46.76814
## model.Proportion_firstBM_2  8 -42.69060
## model.Proportion_firstBM_3 13 -33.53300
## model.Proportion_firstBM_4 14 -29.45546
```

```
#interaction needed
#go with 3
sink("Proportion/BM1proportionbloodfed_lrtests_significance.txt")
lrtest(model.Proportion_firstBM_1,model.Proportion_firstBM_2) #bio rep does not matter
lrtest(model.Proportion_firstBM_1,model.Proportion_firstBM_3)#interaction matters
lrtest(model.Proportion_firstBM_3,model.Proportion_firstBM_4) #bio rep does not matter
sink()

#check residuals
library(DHARMa)
plot(simulateResiduals(model.Proportion_firstBM_1))
```

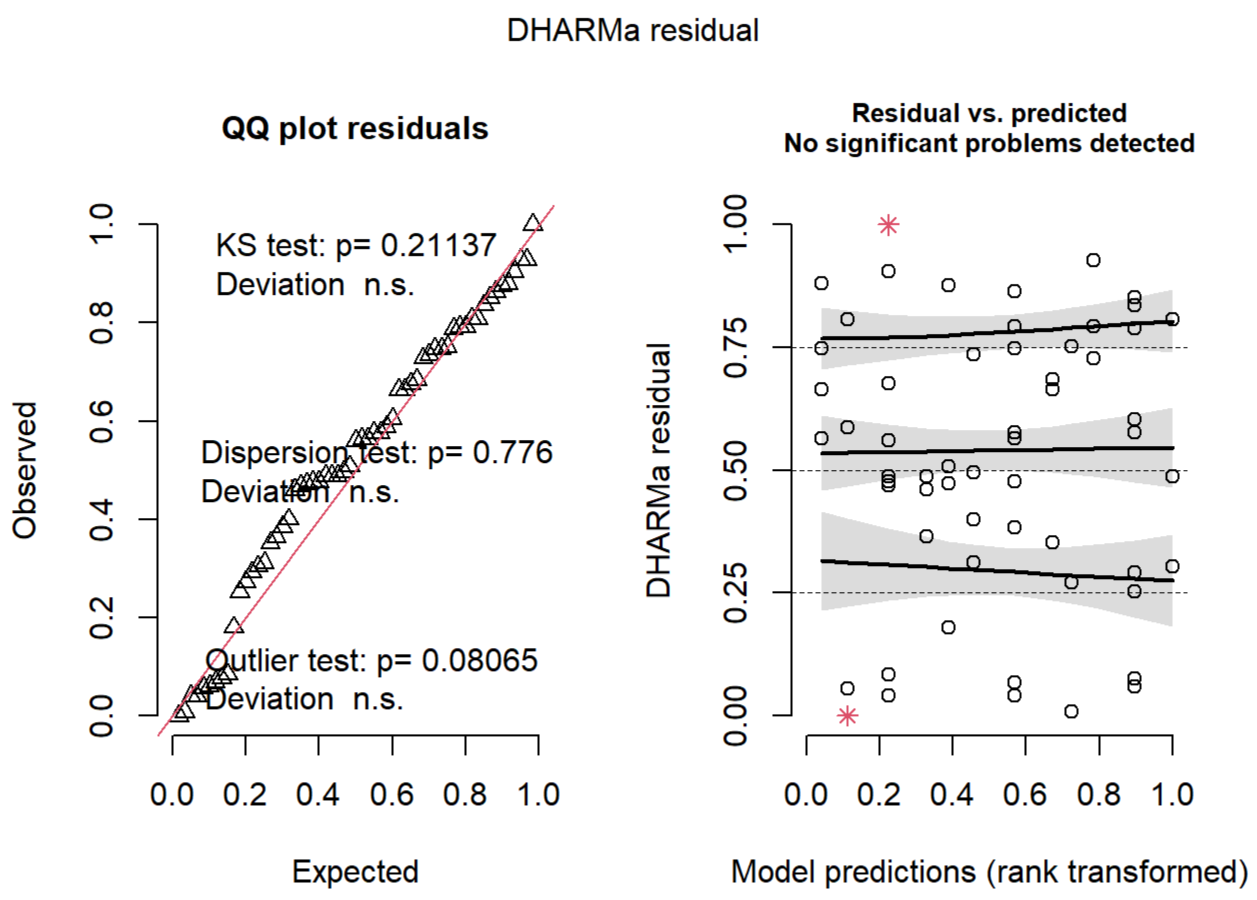


```
plot(simulateResiduals(model.Proportion_firstBM_2))
```

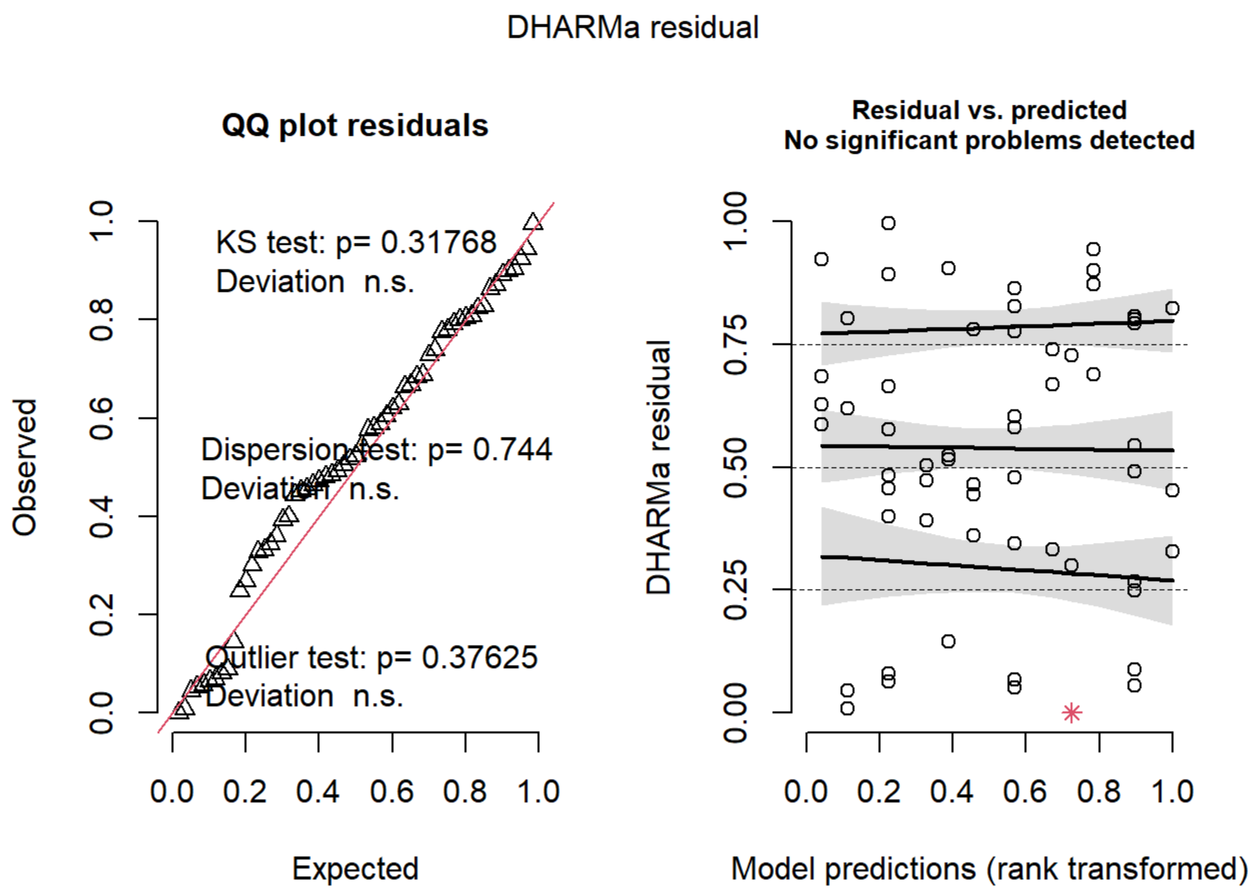

```
plot(simulateResiduals(model.Proportion_firstBM_3))
```

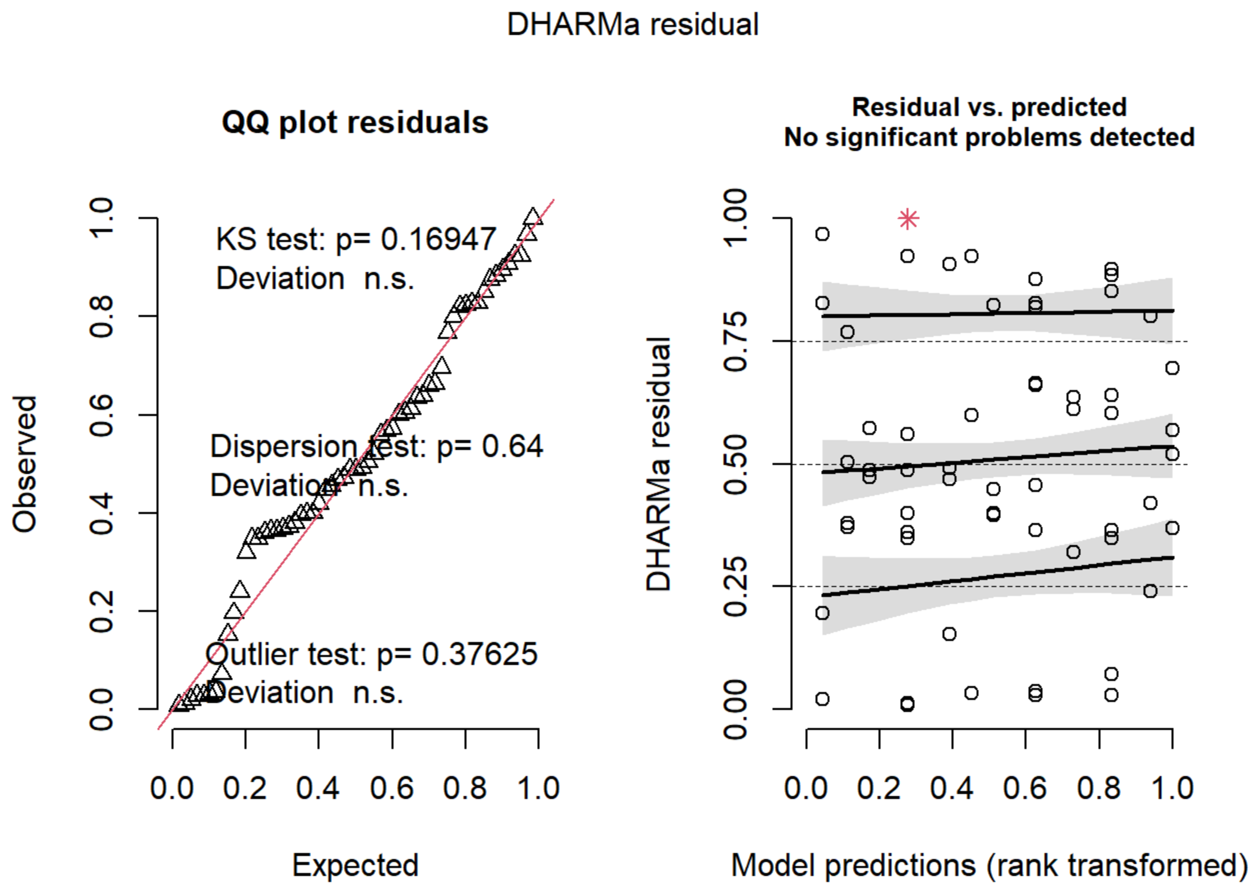


```
plot(simulateResiduals(model.Proportion_firstBM_4))
```

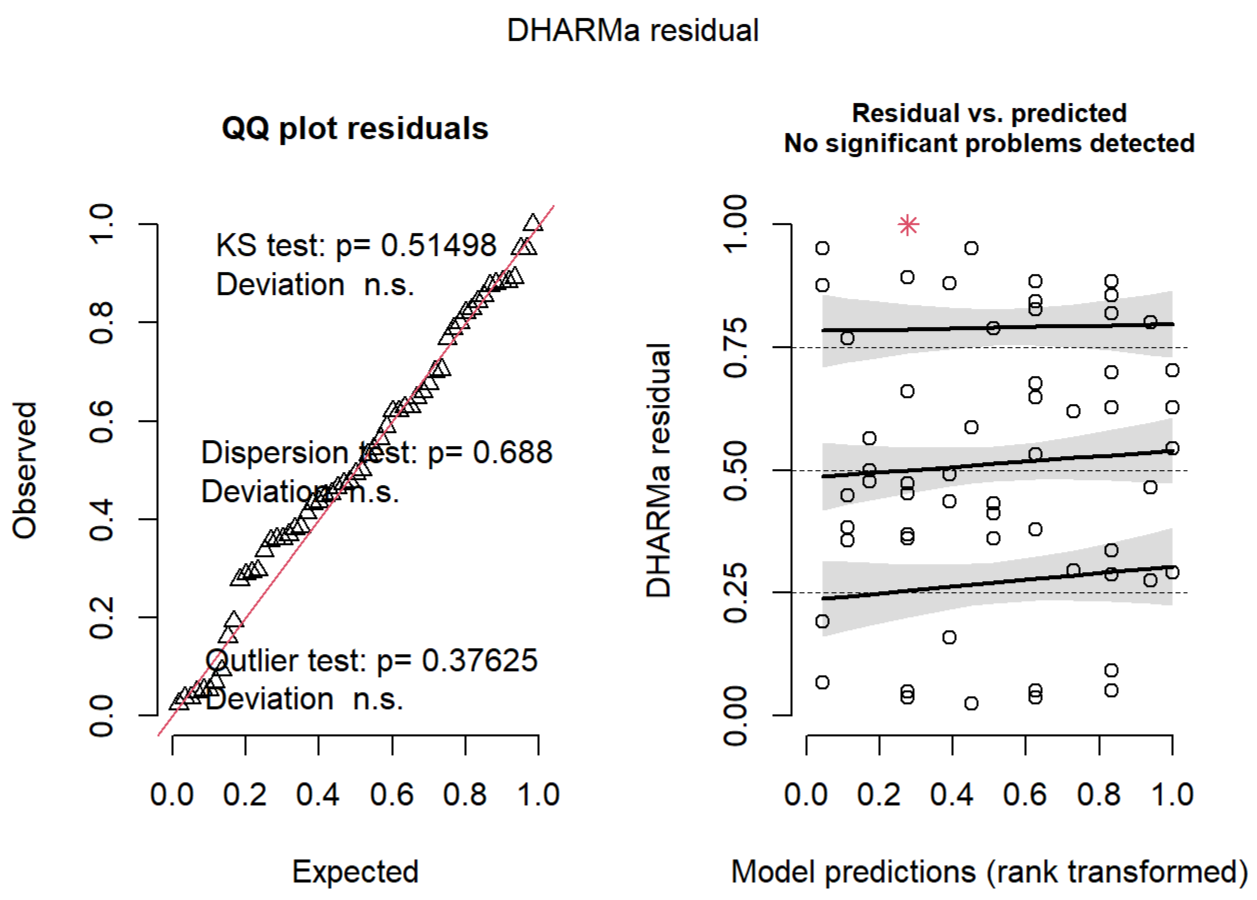


```
plot(fitted(model.Proportion_firstBM_3),
     residuals(model.Proportion_firstBM_3))
```

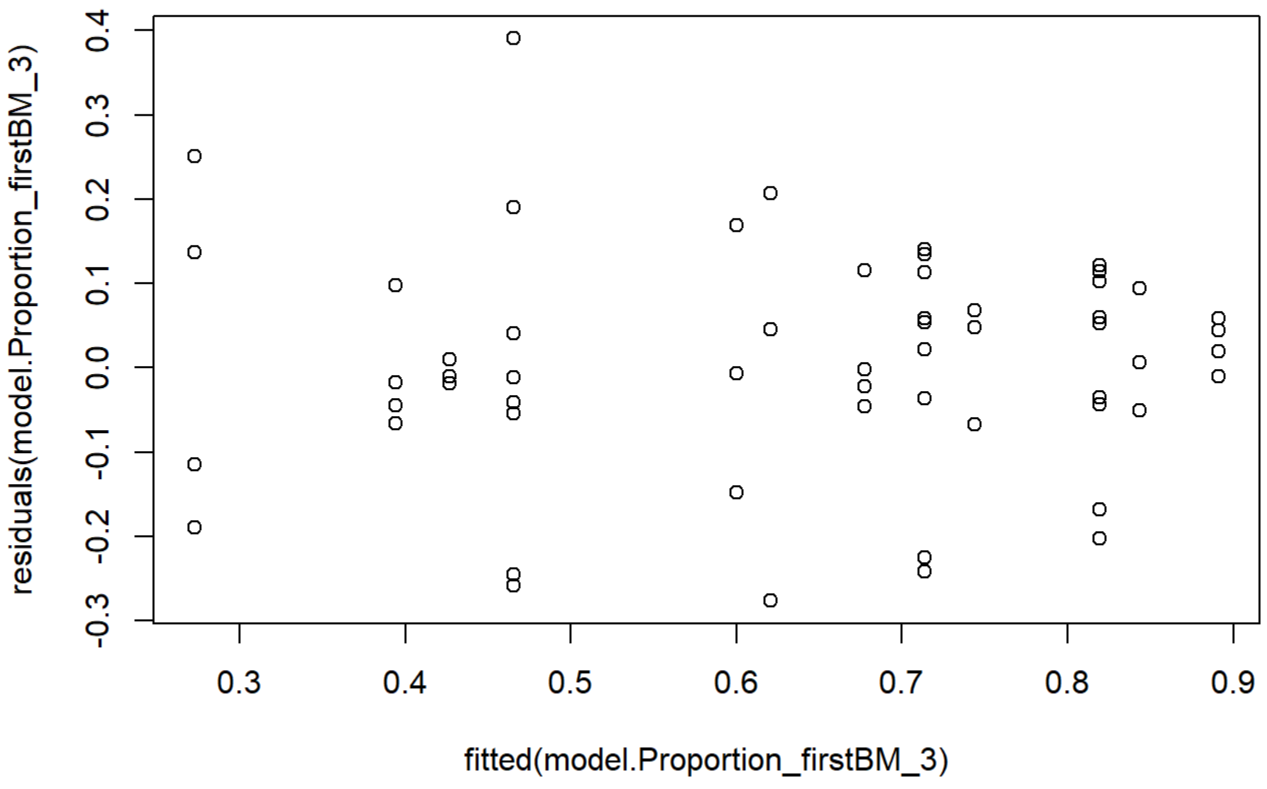

```r
#now check significance of factors in model
library(car)
anova_BM1_proportion <- Anova(model.Proportion_firstBM_3,type=2)
anova_BM1_proportion <- as.data.frame(anova_BM1_proportion)
anova_BM1_proportion
```

```
##                     Chisq Df   Pr(>Chisq)
## Temperature     82.557569  2 1.182638e-18
## Age              8.616196  3 3.485393e-02
## Temperature:Age 11.286412  6 7.991772e-02
```

```r
write_xlsx(anova_BM1_proportion,"Proportion/BM1_proportionBF_ANOVA.xlsx")

sink("Proportion/BM1_proportionBF_ANOVA.txt")
Anova(model.Proportion_firstBM_3,type=2)
sink()

sink("Proportion/BM1_proportionBF_modelsummary.txt")
summary(model.Proportion_firstBM_3)
sink()

#effects and post hoc
library(effects)
```

```
## lattice theme set by effectsTheme()
## See ?effectsTheme for details.
```

```r
ae <- allEffects(model.Proportion_firstBM_3)
plot(ae)
```

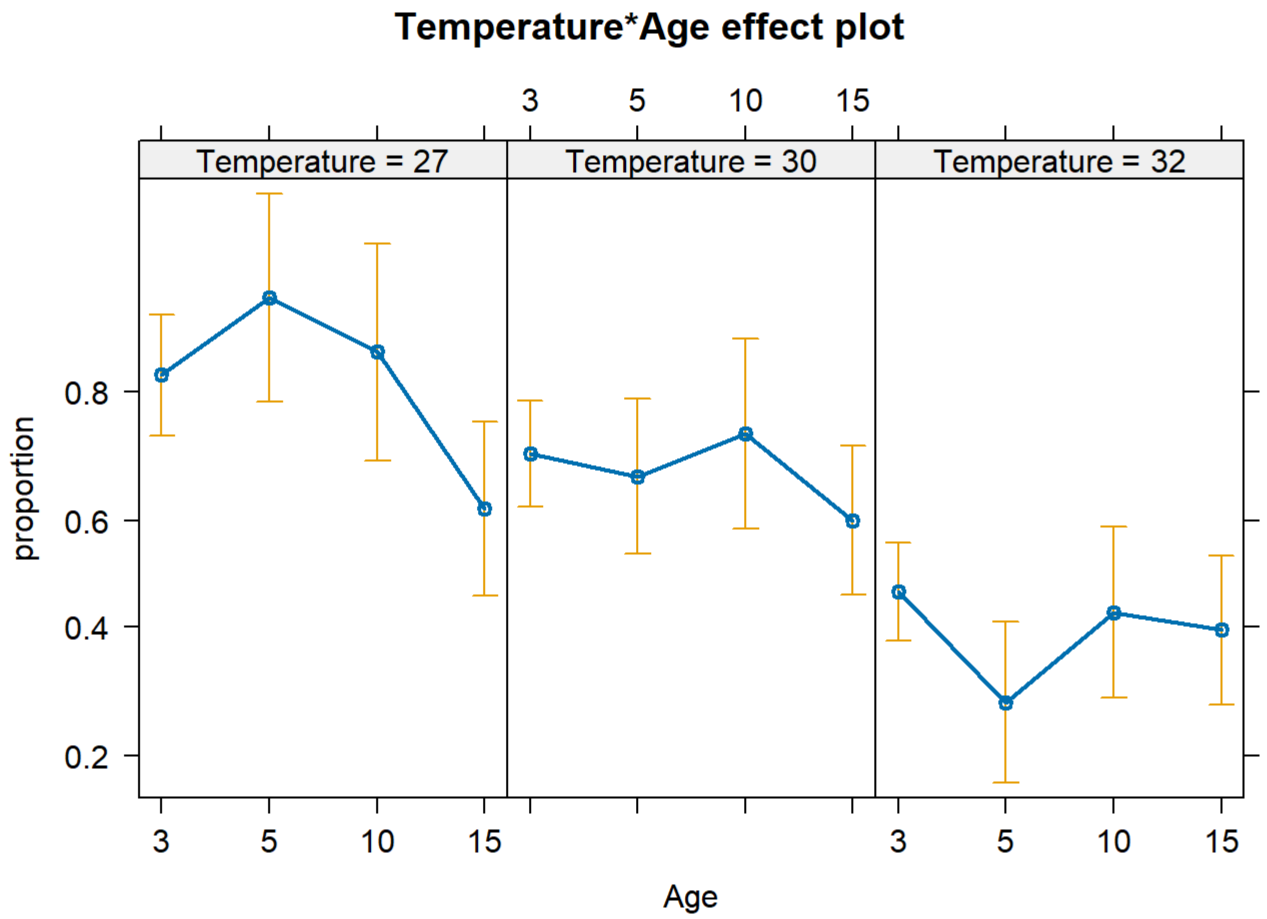


```
#post hoc comparisons:
library(multcomp)
```

```
## Loading required package: mvtnorm
```

```
## Loading required package: survival
```

```
## Loading required package: TH.data
```

```
## Loading required package: MASS
```

```
##
## Attaching package: 'MASS'
```

```
## The following object is masked from 'package:rstatix':
##
##     select
```

```
## The following object is masked from 'package:dplyr':
##
##     select
```

```
##
## Attaching package: 'TH.data'
```

```
## The following object is masked from 'package:MASS':
##
##     geyser
```

```
library(emmeans)
BM1_proportion_emmeans <- emmeans(model.Proportion_firstBM_3,specs=c("Temperature","Age"),type="response")
BM1_proportion_emmeans <- as.data.frame(BM1_proportion_emmeans)
head(BM1_proportion_emmeans)
```

```
##  Temperature Age  response         SE  df asymp.LCL asymp.UCL
##  27          3   0.8194628 0.03384463 Inf 0.7435184 0.8766507
##  30          3   0.7133891 0.04118624 Inf 0.6264689 0.7869613
##  32          3   0.4653705 0.04617198 Inf 0.3769612 0.5560105
##  27          5   0.8911674 0.03810183 Inf 0.7912949 0.9464800
##  30          5   0.6773364 0.06417548 Inf 0.5414136 0.7886956
##  32          5   0.2726222 0.06054874 Inf 0.1708217 0.4054268
##
## Confidence level used: 0.95
## Intervals are back-transformed from the logit scale
```

```
#save and re-run to make emmeans object again:
write_xlsx(BM1_proportion_emmeans, "Proportion/BM1_proportion_emmeans_all.xlsx")
BM1_proportion_emmeans <- emmeans(model.Proportion_firstBM_3,specs=c("Temperature","Age"),type="response")



BM1_proportion_emmeans_pwc <- pairs(BM1_proportion_emmeans,adjust="sidak")
BM1_proportion_emmeans_pwc <- as.data.frame(BM1_proportion_emmeans_pwc)
write_xlsx(BM1_proportion_emmeans_pwc, "Proportion/BM1_proportion_emmeans_pairwisecontrasts.xlsx")
BM1_proportion_emmeans_pwc
```

```
##  contrast                                    odds.ratio        SE  df null
##  Temperature27 Age3 / Temperature30 Age3      1.823597  0.552894 Inf    1
##  Temperature27 Age3 / Temperature32 Age3      5.214549  1.537255 Inf    1
##  Temperature27 Age3 / Temperature27 Age5      0.554322  0.249941 Inf    1
##  Temperature27 Age3 / Temperature30 Age5      2.162261  0.802623 Inf    1
##  Temperature27 Age3 / Temperature32 Age5     12.110483  4.637661 Inf    1
##  Temperature27 Age3 / Temperature27 Age10     0.840551  0.392627 Inf    1
##  Temperature27 Age3 / Temperature30 Age10     1.564923  0.662551 Inf    1
##  Temperature27 Age3 / Temperature32 Age10     6.101952  2.420884 Inf    1
##  Temperature27 Age3 / Temperature27 Age15     2.771581  1.108640 Inf    1
##  Temperature27 Age3 / Temperature30 Age15     3.026444  1.098622 Inf    1
##  Temperature27 Age3 / Temperature32 Age15     6.978189  2.544291 Inf    1
##  Temperature30 Age3 / Temperature32 Age3      2.859486  0.783532 Inf    1
##  Temperature30 Age3 / Temperature27 Age5      0.303972  0.133615 Inf    1
##  Temperature30 Age3 / Temperature30 Age5      1.185712  0.421572 Inf    1
##  Temperature30 Age3 / Temperature32 Age5      6.640987  2.434194 Inf    1
##  Temperature30 Age3 / Temperature27 Age10     0.460930  0.210005 Inf    1
##  Temperature30 Age3 / Temperature30 Age10     0.858152  0.351825 Inf    1
##  Temperature30 Age3 / Temperature32 Age10     3.346108  1.276355 Inf    1
##  Temperature30 Age3 / Temperature27 Age15     1.519843  0.585657 Inf    1
##  Temperature30 Age3 / Temperature30 Age15     1.659601  0.575416 Inf    1
##  Temperature30 Age3 / Temperature32 Age15     3.826607  1.330910 Inf    1
##  Temperature32 Age3 / Temperature27 Age5      0.106303  0.046214 Inf    1
##  Temperature32 Age3 / Temperature30 Age5      0.414659  0.144070 Inf    1
##  Temperature32 Age3 / Temperature32 Age5      2.322441  0.829596 Inf    1
##  Temperature32 Age3 / Temperature27 Age10     0.161193  0.072586 Inf    1
##  Temperature32 Age3 / Temperature30 Age10     0.300107  0.121027 Inf    1
##  Temperature32 Age3 / Temperature32 Age10     1.170178  0.436586 Inf    1
##  Temperature32 Age3 / Temperature27 Age15     0.531509  0.200734 Inf    1
##  Temperature32 Age3 / Temperature30 Age15     0.580385  0.196223 Inf    1
##  Temperature32 Age3 / Temperature32 Age15     1.338215  0.452997 Inf    1
##  Temperature27 Age5 / Temperature30 Age5      3.900731  1.907900 Inf    1
##  Temperature27 Age5 / Temperature32 Age5     21.847377 10.910955 Inf    1
##  Temperature27 Age5 / Temperature27 Age10     1.516358  0.855805 Inf    1
##  Temperature27 Age5 / Temperature30 Age10     2.823129  1.494892 Inf    1
##  Temperature27 Age5 / Temperature32 Age10    11.007955  5.608719 Inf    1
##  Temperature27 Age5 / Temperature27 Age15     4.999948  2.557548 Inf    1
##  Temperature27 Age5 / Temperature30 Age15     5.459721  2.638159 Inf    1
##  Temperature27 Age5 / Temperature32 Age15    12.588691  6.105698 Inf    1
##  Temperature30 Age5 / Temperature32 Age5      5.600841  2.375461 Inf    1
##  Temperature30 Age5 / Temperature27 Age10     0.388737  0.195719 Inf    1
##  Temperature30 Age5 / Temperature30 Age10     0.723744  0.334698 Inf    1
##  Temperature30 Age5 / Temperature32 Age10     2.822023  1.233702 Inf    1
##  Temperature30 Age5 / Temperature27 Age15     1.281798  0.564850 Inf    1
##  Temperature30 Age5 / Temperature30 Age15     1.399666  0.570107 Inf    1
##  Temperature30 Age5 / Temperature32 Age15     3.227264  1.317142 Inf    1
##  Temperature32 Age5 / Temperature27 Age10     0.069407  0.035573 Inf    1
##  Temperature32 Age5 / Temperature30 Age10     0.129221  0.060885 Inf    1
##  Temperature32 Age5 / Temperature32 Age10     0.503857  0.224119 Inf    1
##  Temperature32 Age5 / Temperature27 Age15     0.228858  0.102773 Inf    1
##  Temperature32 Age5 / Temperature30 Age15     0.249903  0.104031 Inf    1
##  Temperature32 Age5 / Temperature32 Age15     0.576211  0.239787 Inf    1
##  Temperature27 Age10 / Temperature30 Age10    1.861782  1.010978 Inf    1
##  Temperature27 Age10 / Temperature32 Age10    7.259467  3.795086 Inf    1
##  Temperature27 Age10 / Temperature27 Age15    3.297339  1.731502 Inf    1
##  Temperature27 Age10 / Temperature30 Age15    3.600547  1.791401 Inf    1
```

```
##  Temperature27 Age10 / Temperature32 Age15   8.301923  4.141464 Inf    1
##  Temperature30 Age10 / Temperature32 Age10   3.899203  1.882420 Inf    1
##  Temperature30 Age10 / Temperature27 Age15   1.771066  0.860326 Inf    1
##  Temperature30 Age10 / Temperature30 Age15   1.933925  0.881384 Inf    1
##  Temperature30 Age10 / Temperature32 Age15   4.459127  2.036361 Inf    1
##  Temperature32 Age10 / Temperature27 Age15   0.454212  0.209633 Inf    1
##  Temperature32 Age10 / Temperature30 Age15   0.495980  0.213143 Inf    1
##  Temperature32 Age10 / Temperature32 Age15   1.143599  0.491740 Inf    1
##  Temperature27 Age15 / Temperature30 Age15   1.091956  0.473333 Inf    1
##  Temperature27 Age15 / Temperature32 Age15   2.517765  1.092966 Inf    1
##  Temperature30 Age15 / Temperature32 Age15   2.305739  0.922597 Inf    1
##  z.ratio p.value
##    1.982  0.9598
##    5.602  <.0001
##   -1.309  1.0000
##    2.077  0.9212
##    6.513  <.0001
##   -0.372  1.0000
##    1.058  1.0000
##    4.559  0.0003
##    2.549  0.5122
##    3.051  0.1401
##    5.328  <.0001
##    3.834  0.0083
##   -2.709  0.3603
##    0.479  1.0000
##    5.165  <.0001
##   -1.700  0.9979
##   -0.373  1.0000
##    3.166  0.0969
##    1.086  1.0000
##    1.461  1.0000
##    3.858  0.0075
##   -5.156  <.0001
##   -2.534  0.5273
##    2.359  0.7051
##   -4.053  0.0033
##   -2.985  0.1711
##    0.421  1.0000
##   -1.674  0.9985
##   -1.609  0.9995
##    0.861  1.0000
##    2.783  0.2999
##    6.175  <.0001
##    0.738  1.0000
##    1.960  0.9661
##    4.708  0.0002
##    3.146  0.1034
##    3.513  0.0288
##    5.222  <.0001
##    4.062  0.0032
##   -1.877  0.9838
##   -0.699  1.0000
##    2.373  0.6910
##    0.563  1.0000
##    0.825  1.0000
```

```
##    2.871  0.2373
##   -5.205  <.0001
##   -4.343  0.0009
##   -1.541  0.9998
##   -3.284  0.0654
##   -3.331  0.0555
##   -1.325  1.0000
##    1.145  1.0000
##    3.792  0.0098
##    2.272  0.7859
##    2.575  0.4858
##    4.243  0.0015
##    2.819  0.2732
##    1.177  1.0000
##    1.447  1.0000
##    3.274  0.0677
##   -1.710  0.9976
##   -1.632  0.9992
##    0.312  1.0000
##    0.203  1.0000
##    2.127  0.8939
##    2.088  0.9159
##
## P value adjustment: sidak method for 66 tests
## Tests are performed on the log odds ratio scale
```

```
BM1_proportion_emmeans_TEMP <- emmeans(model.Proportion_firstBM_3,specs=c("Temperature"),
                                       type="response")
```

```
## NOTE: Results may be misleading due to involvement in interactions
```

```
BM1_proportion_emmeans_TEMP
```

```
##  Temperature response     SE  df asymp.LCL asymp.UCL
##  27             0.810 0.0271 Inf     0.751     0.857
##  30             0.686 0.0313 Inf     0.622     0.744
##  32             0.387 0.0332 Inf     0.324     0.453
##
## Results are averaged over the levels of: Age
## Confidence level used: 0.95
## Intervals are back-transformed from the logit scale
```

```
BM1_proportion_emmeans_TEMP_pairs <- pairs(BM1_proportion_emmeans_TEMP, adjust="sidak")
BM1_proportion_emmeans_TEMP_pairs
```

```
##  contrast                      odds.ratio    SE  df null z.ratio p.value
##  Temperature27 / Temperature30       1.95 0.441 Inf    1   2.955  0.0094
##  Temperature27 / Temperature32       6.76 1.525 Inf    1   8.460  <.0001
##  Temperature30 / Temperature32       3.46 0.700 Inf    1   6.148  <.0001
##
## Results are averaged over the levels of: Age
## P value adjustment: sidak method for 3 tests
## Tests are performed on the log odds ratio scale
```

```
BM1_proportion_emmeans_TEMP_pairs <- as.data.frame(BM1_proportion_emmeans_TEMP_pairs)
write_xlsx(BM1_proportion_emmeans_TEMP_pairs, "Proportion/BM1_proprtion_emmeans_TEMP_pairs.xlsx")



BM1_proportion_emmeans_AGE <- emmeans(model.Proportion_firstBM_3,specs=c("Age"),
                                      type="response")
```

```
## NOTE: Results may be misleading due to involvement in interactions
```

```
BM1_proportion_emmeans_AGE
```

```
##  Age response     SE  df asymp.LCL asymp.UCL
##  3      0.682 0.0259 Inf     0.629     0.730
##  5      0.650 0.0437 Inf     0.561     0.731
##  10     0.694 0.0449 Inf     0.600     0.774
##  15     0.539 0.0429 Inf     0.455     0.621
##
## Results are averaged over the levels of: Temperature
## Confidence level used: 0.95
## Intervals are back-transformed from the logit scale
```

```
BM1_proportion_emmeans_AGE_pairs <- pairs(BM1_proportion_emmeans_AGE, adjust="sidak")
BM1_proportion_emmeans_AGE_pairs <- as.data.frame(BM1_proportion_emmeans_AGE_pairs)
BM1_proportion_emmeans_AGE_pairs
```

```
##  contrast      odds.ratio        SE  df null z.ratio p.value
##  Age3 / Age5    1.1514067 0.2593402 Inf    1   0.626  0.9894
##  Age3 / Age10   0.9450615 0.2283551 Inf    1  -0.234  1.0000
##  Age3 / Age15   1.8326764 0.3842067 Inf    1   2.890  0.0229
##  Age5 / Age10   0.8207886 0.2337236 Inf    1  -0.694  0.9820
##  Age5 / Age15   1.5916847 0.4110368 Inf    1   1.800  0.3608
##  Age10 / Age15  1.9392139 0.5288220 Inf    1   2.429  0.0876
##
## Results are averaged over the levels of: Temperature
## P value adjustment: sidak method for 6 tests
## Tests are performed on the log odds ratio scale
```

```
write_xlsx(BM1_proportion_emmeans_AGE_pairs, "Proportion/BM1_proprtion_emmeans_AGE_pairs.xlsx")

sink("Proportion/modelparameters_BM1_proportionBF.txt")
parameters::model_parameters(
  model.Proportion_firstBM_3, exponentiate = TRUE, ci_method = "wald",
  effects = "all",
  component = "conditional",
  group_level = TRUE,
  verbose = FALSE
)
sink()
```

Code for Figure 2: relative blood meal size

First clear workspace and import data.

```
################
# clear existing workspace
rm(list = ls(all = TRUE))
graphics.off()
shell("cls")

#set wd to your project folder
getwd() #check working directory
```

```
## [1] "C:/Users/linzm/OneDrive - Vanderbilt/Hillyer_Lab/Blood_feeding_project/Bloodfeeding"
```

```
#sessionInfo()
###########################

#####################
#load libraries needed:
library(readxl)
library(writexl)
library(ggplot2)
library(dplyr)
library(tidyverse)
library(rstatix)
library(car)
library(ggpubr)
library(emmeans)
library(lmtest)
library(glmmTMB)
library(DHARMa)
#######################################################

#######################################################
#import the data and clean it up:
BMsize_data <- read_xlsx("SupplementaryData1_RawData.xlsx",
                             sheet = "Fig2")
BMsize_data <- as.data.frame(BMsize_data)
str(BMsize_data)
```

```
## 'data.frame':    578 obs. of  10 variables:
##  $ ID_Number            : num  1 2 3 4 5 6 7 8 9 10 ...
##  $ Temperature          : num  27 27 27 27 27 27 27 27 27 27 ...
##  $ Age                  : num  3 3 3 3 3 3 3 3 3 3 ...
##  $ Age_of_BM            : num  3 3 3 3 3 3 3 3 3 3 ...
##  $ BM1_Date             : POSIXct, format: "2024-02-06" "2024-02-06" ...
##  $ Pre_or_Post_BM       : chr  "Post" "Post" "Post" "Post" ...
##  $ Mass_per_mosquito(mg): num  2.88 1.96 2.7 3.64 3.58 2.12 1.1 1.1 1.32 0.88 ...
##  $ Technical_Replicate  : num  1 2 3 4 5 6 1 2 3 4 ...
##  $ Biological_Replicate : num  1 1 1 1 1 1 1 1 1 1 ...
##  $ BM_Number            : num  1 1 1 1 1 1 1 1 1 1 ...
```

```
head(BMsize_data)
```

```
##   ID_Number Temperature Age Age_of_BM   BM1_Date Pre_or_Post_BM
## 1         1          27   3         3 2024-02-06           Post
## 2         2          27   3         3 2024-02-06           Post
## 3         3          27   3         3 2024-02-06           Post
## 4         4          27   3         3 2024-02-06           Post
## 5         5          27   3         3 2024-02-06           Post
## 6         6          27   3         3 2024-02-06           Post
##   Mass_per_mosquito(mg) Technical_Replicate Biological_Replicate BM_Number
## 1                  2.88                   1                    1         1
## 2                  1.96                   2                    1         1
## 3                  2.70                   3                    1         1
## 4                  3.64                   4                    1         1
## 5                  3.58                   5                    1         1
## 6                  2.12                   6                    1         1
```

```
#variables of interest:
BMsize_data$Temperature <- as.factor(BMsize_data$Temperature)
BMsize_data$Age <- as.factor(BMsize_data$Age)
BMsize_data$Pre_or_Post_BM <- as.factor(BMsize_data$Pre_or_Post_BM)

BMsize_data$Age_of_BM <- as.factor(BMsize_data$Age_of_BM)
BMsize_data$BM_Number <- as.factor(BMsize_data$BM_Number)

str(BMsize_data)
```

```
## 'data.frame':    578 obs. of  10 variables:
##  $ ID_Number            : num  1 2 3 4 5 6 7 8 9 10 ...
##  $ Temperature          : Factor w/ 3 levels "27","30","32": 1 1 1 1 1 1 1 1 1 1 ...
##  $ Age                  : Factor w/ 4 levels "3","5","10","15": 1 1 1 1 1 1 1 1 1 1 ...
##  $ Age_of_BM            : Factor w/ 4 levels "3","5","10","15": 1 1 1 1 1 1 1 1 1 1 ...
##  $ BM1_Date             : POSIXct, format: "2024-02-06" "2024-02-06" ...
##  $ Pre_or_Post_BM       : Factor w/ 2 levels "Post","Pre": 1 1 1 1 1 1 2 2 2 2 ...
##  $ Mass_per_mosquito(mg): num  2.88 1.96 2.7 3.64 3.58 2.12 1.1 1.1 1.32 0.88 ...
##  $ Technical_Replicate  : num  1 2 3 4 5 6 1 2 3 4 ...
##  $ Biological_Replicate : num  1 1 1 1 1 1 1 1 1 1 ...
##  $ BM_Number            : Factor w/ 1 level "1": 1 1 1 1 1 1 1 1 1 1 ...
```

```
#summary stats:
BMsize_summary1 <- BMsize_data %>%
  group_by(Temperature,Age,Age_of_BM,Biological_Replicate,BM_Number,Pre_or_Post_BM) %>%
  summarise(mean_mass = mean(`Mass_per_mosquito(mg)`),
            median_mass = median(`Mass_per_mosquito(mg)`),
            sd_mass = sd(`Mass_per_mosquito(mg)`),
            n_mass = n(),
            SE_mass = sd(`Mass_per_mosquito(mg)`)/sqrt(n()))
```

```
## `summarise()` has grouped output by 'Temperature', 'Age', 'Age_of_BM',
## 'Biological_Replicate', 'BM_Number'. You can override using the `.groups`
## argument.
```

```
BMsize_summary1
```

```
## # A tibble: 90 × 11
## # Groups:   Temperature, Age, Age_of_BM, Biological_Replicate, BM_Number [45]
##    Temperature Age   Age_of_BM Biological_Replicate BM_Number Pre_or_Post_BM
##    <fct>       <fct> <fct>                    <dbl> <fct>     <fct>
##  1 27          3     3                            1 1         Post
##  2 27          3     3                            1 1         Pre
##  3 27          3     3                            2 1         Post
##  4 27          3     3                            2 1         Pre
##  5 27          3     3                            3 1         Post
##  6 27          3     3                            3 1         Pre
##  7 27          3     3                            4 1         Post
##  8 27          3     3                            4 1         Pre
##  9 27          3     3                            5 1         Post
## 10 27          3     3                            5 1         Pre
## # ℹ 80 more rows
## # ℹ 5 more variables: mean_mass <dbl>, median_mass <dbl>, sd_mass <dbl>,
## #   n_mass <int>, SE_mass <dbl>
```

```
write_xlsx(BMsize_summary1, "BM_size/BMsize_summary1.xlsx")

BMsize_summary2 <- BMsize_data %>%
  group_by(Temperature,Age,Age_of_BM,BM_Number,Pre_or_Post_BM) %>% #ignores biological replicate
  summarise(mean_mass = mean(`Mass_per_mosquito(mg)`),
            median_mass = median(`Mass_per_mosquito(mg)`),
            sd_mass = sd(`Mass_per_mosquito(mg)`),
            n_mass = n(),
            SE_mass = sd(`Mass_per_mosquito(mg)`)/sqrt(n()))
```

```
## `summarise()` has grouped output by 'Temperature', 'Age', 'Age_of_BM',
## 'BM_Number'. You can override using the `.groups` argument.
```

```
write_xlsx(BMsize_summary2, "BM_size/BMsize_summary2.xlsx")

BMsize_summary1_firstBM <- BMsize_summary1

##########
```

Calculate the total blood meal size values:

```
percentdiff_values <- data.frame()

for(i in unique(BMsize_summary1_firstBM$Temperature)){
  temp_sub <- subset(BMsize_summary1_firstBM,Temperature==i)
  #tempmass <- data.frame()

  for (j in unique(temp_sub$Age)){
    age_sub <- subset(temp_sub, Age==j)
    #agemass <- data.frame()

    for (k in unique(age_sub$Biological_Replicate)){
      Rep_sub <- subset(age_sub, Biological_Replicate==k)
      #Repmass <- data.frame()

      pre_mass_df <- subset(Rep_sub, Rep_sub$Temperature==i & Rep_sub$Age==j & Rep_sub$Biological_Replicate
==k & Rep_sub$Pre_or_Post_BM=="Pre")
      pre_mass <- pre_mass_df[,7]
      pre_mass_value <- as.numeric(pre_mass$mean_mass[1])

      post_mass_df <- subset(Rep_sub, Rep_sub$Temperature==i & Rep_sub$Age==j & Rep_sub$Biological_Replicat
e==k & Rep_sub$Pre_or_Post_BM=="Post")
      post_mass <- post_mass_df[,7]
      post_mass_value <- as.numeric(post_mass$mean_mass[1])

      post_minus_pre <- (post_mass_value - pre_mass_value)
      percent_diff <- (((post_minus_pre)/pre_mass)*100)

      post_over_pre <- (post_mass_value/pre_mass_value)

      names(percent_diff)[1]<-"percent_diff"
      #names(post_over_pre)[1]<-"post_over_pre"

      percent_df <-cbind(Temperature=i,Age=j, Biological_Replicate=k,
                         pre_mass_value,post_mass_value,
                         post_minus_pre,percent_diff,
                         post_over_pre)
      percentdiff_values <- rbind(percentdiff_values,percent_df)
    }
  }
}

#view(percentdiff_values)
str(percentdiff_values)
```

```
## 'data.frame':    45 obs. of  8 variables:
##  $ Temperature         : chr  "27" "27" "27" "27" ...
##  $ Age                 : chr  "3" "3" "3" "3" ...
##  $ Biological_Replicate: num  1 2 3 4 5 1 2 3 4 1 ...
##  $ pre_mass_value      : num  1.06 1.02 1.14 1.19 1.04 ...
##  $ post_mass_value     : num  2.81 1.96 2.31 2.29 1.94 ...
##  $ post_minus_pre      : num  1.749 0.938 1.169 1.105 0.907 ...
##  $ percent_diff        : num  164.4 91.5 102.2 92.9 87.5 ...
##  $ post_over_pre       : num  2.64 1.92 2.02 1.93 1.87 ...
```

```
percentdiff_values<-as.data.frame(percentdiff_values)

percentdiff_values$Temperature <- as.factor(percentdiff_values$Temperature)
percentdiff_values$Age <- as.factor(percentdiff_values$Age)

write_xlsx(percentdiff_values, "BM_size/percentdiffvalues_all.xlsx")

#summary stats on % diff:
BM1_percentdiff_summary <- percentdiff_values %>%
  group_by(Temperature,Age) %>%
  summarise(mean_pre = mean(pre_mass_value),
            mean_post = mean(post_mass_value),
            mean_postminuspre = mean(post_minus_pre),
            mean_percentdiff = mean(percent_diff),
            mean_postoverpre = mean(post_over_pre),
            median_pre = median(pre_mass_value),
            median_post = median(post_mass_value),
            median_postminuspre = median(post_minus_pre),
            median_percentdiff = median(percent_diff),
            median_postoverpre = median(post_over_pre),
            n_mass = n(),
            sd_pre = sd(pre_mass_value),
            sd_post = sd(post_mass_value),
            sd_postminuspre = sd(post_minus_pre),
            sd_percentdiff = sd(percent_diff),
            sd_postoverpre = sd(post_over_pre),
            SE_pre = sd(pre_mass_value)/sqrt(n()),
            SE_post = sd(post_mass_value)/sqrt(n()),
            SE_postminuspre = sd(post_minus_pre)/sqrt(n()),
            SE_percentdiff = sd(percent_diff)/sqrt(n()),
            SE_postoverpre = sd(post_over_pre)/sqrt(n()))
```

```
## `summarise()` has grouped output by 'Temperature'. You can override using the
## `.groups` argument.
```

```
BM1_percentdiff_summary <- as.data.frame(BM1_percentdiff_summary)
BM1_percentdiff_summary$Temperature <- as.factor(BM1_percentdiff_summary$Temperature)
BM1_percentdiff_summary$Age <- as.factor(BM1_percentdiff_summary$Age)
str(BM1_percentdiff_summary)
```

```
## 'data.frame':    12 obs. of  23 variables:
##  $ Temperature        : Factor w/ 3 levels "27","30","32": 1 1 1 1 2 2 2 2 3 3 ...
##  $ Age                : Factor w/ 4 levels "10","15","3",..: 1 2 3 4 1 2 3 4 1 2 ...
##  $ mean_pre           : num  0.993 1.078 1.092 1.041 0.959 ...
##  $ mean_post          : num  1.95 2.02 2.27 2.1 2.09 ...
##  $ mean_postminuspre  : num  0.96 0.938 1.174 1.063 1.134 ...
##  $ mean_percentdiff   : num  96.8 87 107.7 102.1 120.6 ...
##  $ mean_postoverpre   : num  1.97 1.87 2.08 2.02 2.21 ...
##  $ median_pre         : num  1 1.068 1.064 1.042 0.907 ...
##  $ median_post        : num  1.9 2.01 2.29 2 2.07 ...
##  $ median_postminuspre: num  0.933 0.943 1.105 1.021 1.09 ...
##  $ median_percentdiff : num  96.7 86.3 92.9 104.7 120.2 ...
##  $ median_postoverpre : num  1.97 1.86 1.93 2.05 2.2 ...
##  $ n_mass             : int  3 3 5 4 3 4 5 4 3 4 ...
##  $ sd_pre             : num  0.0262 0.0189 0.0718 0.0791 0.1485 ...
##  $ sd_post            : num  0.0953 0.0295 0.353 0.2265 0.1124 ...
##  $ sd_postminuspre    : num  0.0923 0.0139 0.3401 0.1802 0.0766 ...
##  $ sd_percentdiff     : num  9.57 1.26 32.16 14.9 24.06 ...
##  $ sd_postoverpre     : num  0.0957 0.0126 0.3216 0.149 0.2406 ...
##  $ SE_pre             : num  0.0151 0.0109 0.0321 0.0396 0.0857 ...
##  $ SE_post            : num  0.055 0.017 0.1578 0.1132 0.0649 ...
##  $ SE_postminuspre    : num  0.0533 0.008 0.1521 0.0901 0.0442 ...
##  $ SE_percentdiff     : num  5.526 0.729 14.382 7.45 13.892 ...
##  $ SE_postoverpre     : num  0.05526 0.00729 0.14382 0.0745 0.13892 ...
```

```
write_xlsx(BM1_percentdiff_summary, "BM_size/BM1_BMsize_summary.xlsx")

#relevel
percentdiff_values <- percentdiff_values %>%
  mutate(Age = fct_relevel(Age, "3","5","10","15"))
BM1_percentdiff_summary <- BM1_percentdiff_summary %>%
  mutate(Age = fct_relevel(Age, "3","5","10","15"))



#############################################
```

Plot blood meal size data.

```
#plot ratio of post / pre BM size:
BM1_percentdiff_summary$Age <- factor(BM1_percentdiff_summary$Age,
                                      labels = c("3","5","10","15"))
BM1_percentdiff_summary$Temperature <- factor(BM1_percentdiff_summary$Temperature,
                                              labels = c("27˚C","30˚C","32˚C"))
percentdiff_values$Age <- factor(percentdiff_values$Age,
                                 labels = c("3","5","10","15"))
percentdiff_values$Temperature <- factor(percentdiff_values$Temperature,
                                         labels = c("27˚C","30˚C","32˚C"))



postpreratio_mass_BM1_byage <- BM1_percentdiff_summary %>%
  ggplot(aes(x=Age,y=mean_postoverpre,group=Age))+
  geom_bar(aes(fill=Age),
           stat = "identity",
           position = position_dodge(1),
           width = 0.8) +
  scale_shape_identity(guide="legend")+
  facet_grid(~Temperature)+
  geom_errorbar(aes(ymin=mean_postoverpre - SE_postoverpre,
                    ymax=mean_postoverpre + SE_postoverpre),
                width=0.8,position=position_dodge(0.9),
                color="black")+
  ylab(expression("Ratio of post/pre-BM mass"))+
  xlab("Adult age (days)") +
  theme_pubr()+
  theme(legend.position = "none")+
  geom_jitter(data=percentdiff_values, aes(x=Age,y=post_over_pre),#color=ZOI_italic$Technical_Rep,
              position = "jitter",size=1)+
  scale_fill_manual(values= c("#DCD1E9","#BAA4D3","#9776BE","#7549A8"))+
  theme(panel.background = element_rect(fill = NA, color = "black"))+
  theme(panel.spacing = unit(0.5, "lines"))
postpreratio_mass_BM1_byage
```

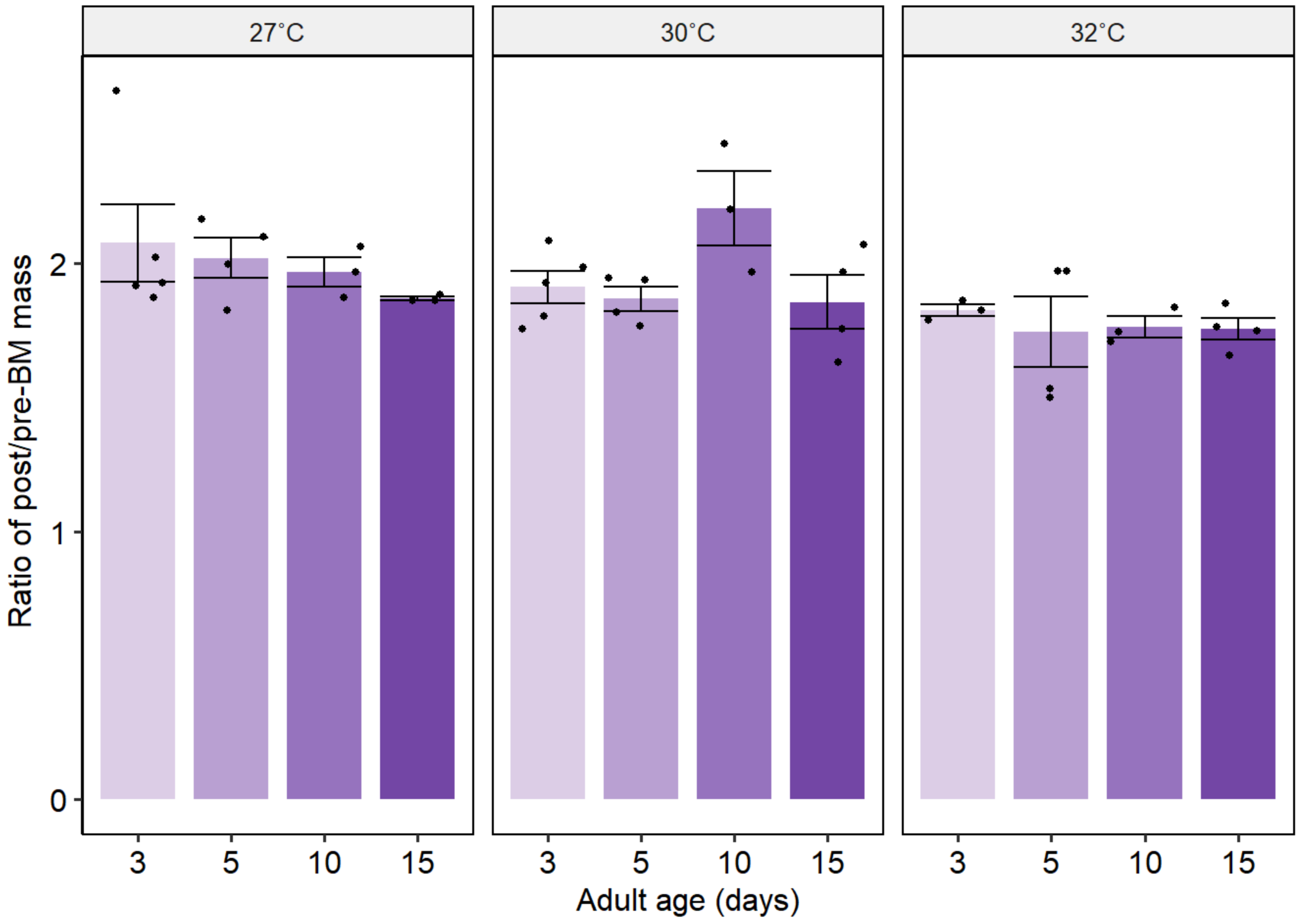


```
ggsave("BM_size/postpreratio_mass_BM1_byage.png", plot=postpreratio_mass_BM1_byage,width = 6, height = 4, u
nits = "in", dpi = 600)
ggsave("BM_size/postpreratio_mass_BM1_byage.pdf", plot=postpreratio_mass_BM1_byage,width = 6, height = 4, u
nits = "in", dpi = 600)


#interaction plot with raw data:
BM1size_interactionplot_rawdata <- BM1_percentdiff_summary %>%
  ggplot() +
  aes(x = Age, group = Temperature, y = mean_postoverpre) +
  #stat_summary(fun.y = mean, geom = "point",shape="Temperature") +
  geom_point(aes(shape=Temperature),size=3)+
  # scale_size_manual(values=c(2,5,10)) +
  #stat_summary(fun.y = mean, geom = "line")+
  geom_line(aes(linetype = Temperature),linewidth=0.6)+
  theme_pubr()+
  scale_shape(labels=c(27,30,32))+
  scale_linetype(labels=c(27,30,32))+
  guides(shape = guide_legend(title = "Temperature (˚C)"),
         linetype = guide_legend(title = "Temperature (˚C)"))+
  xlab("Adult Age (days)") +
  theme(legend.position = "right")+
  scale_x_discrete(labels=c(3,5,10,15))+
  ylab(expression("Ratio of post/pre-BM body mass"))+
  theme(panel.background = element_rect(fill = NA, color = "black"))+
  theme(panel.spacing = unit(0.6, "lines"))
BM1size_interactionplot_rawdata
```

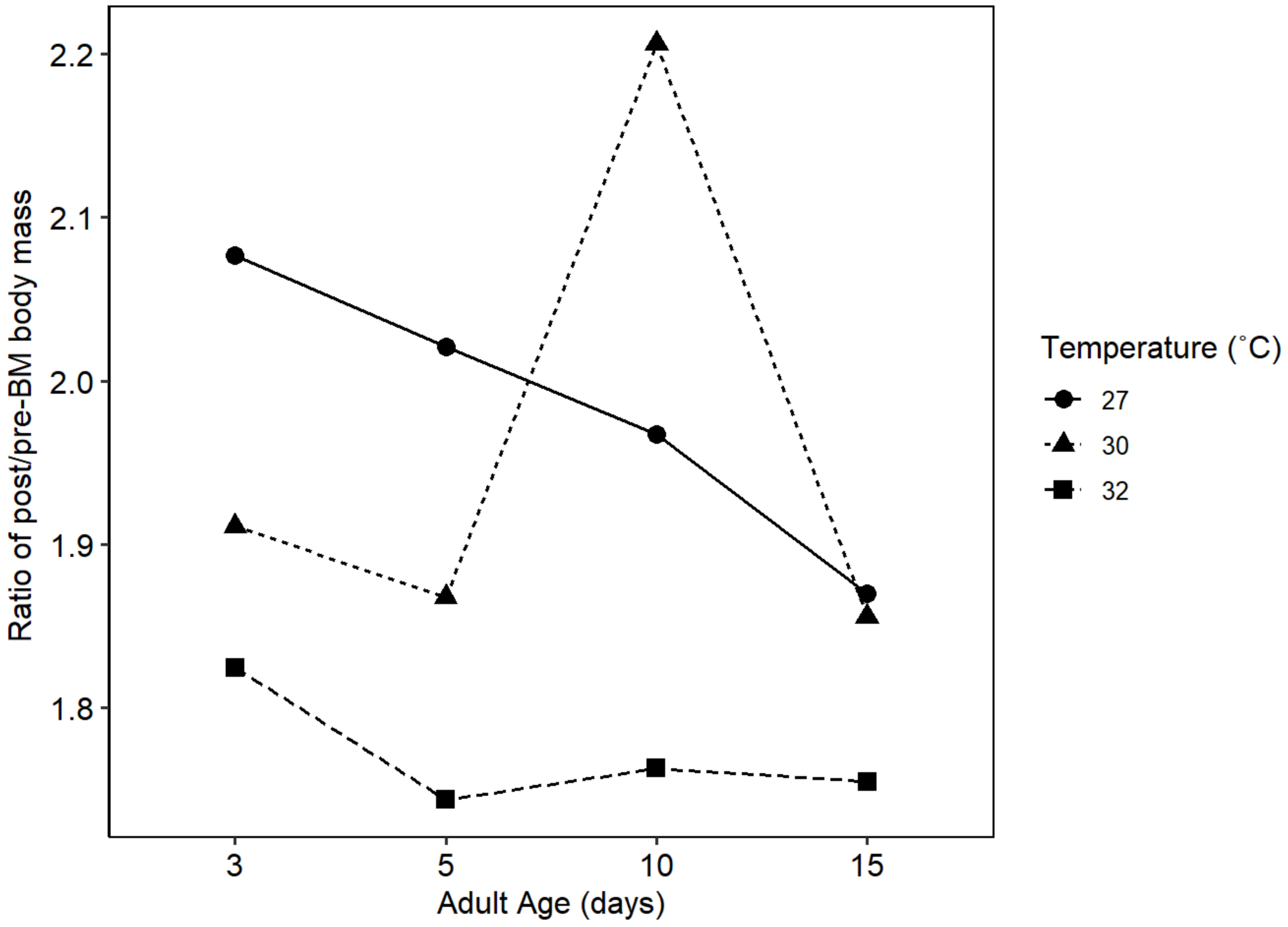

Ratio of post/pre-BM body mass
2.2
2.1
2.0
1.9
1.8
3
5
10
15
Adult Age (days)
Temperature (˚C)
27
30
32

```
ggsave("BM_Size/BM1size_interactionplot_rawdata.png",plot=BM1size_interactionplot_rawdata,width = 5.5, height = 4, units = "in", dpi = 600)
ggsave("BM_Size/BM1size_interactionplot_rawdata.pdf",plot=BM1size_interactionplot_rawdata,width = 5.5, height = 4, units = "in", dpi = 600)





BM1_percentdiff_summary$Age <- factor(BM1_percentdiff_summary$Age,
                                      labels = c("3 days","5 days","10 days","15 days"))
BM1_percentdiff_summary$Temperature <- factor(BM1_percentdiff_summary$Temperature,
                                              labels = c("27","30","32"))
percentdiff_values$Age <- factor(percentdiff_values$Age,
                                 labels = c("3 days","5 days","10 days","15 days"))
percentdiff_values$Temperature <- factor(percentdiff_values$Temperature,
                                         labels = c("27","30","32"))

postpreratio_mass_BM1_bytemp <- BM1_percentdiff_summary %>%
  ggplot(aes(x=Temperature,y=mean_postoverpre,group=Temperature))+
  geom_bar(aes(fill=Temperature),
           stat = "identity",
           position = position_dodge(1),
           width = 0.8) +
  scale_shape_identity(guide="legend")+
  facet_grid(~Age)+
  geom_errorbar(aes(ymin=mean_postoverpre - SE_postoverpre,
                    ymax=mean_postoverpre + SE_postoverpre),
                width=0.8,position=position_dodge(0.9),
                color="black")+
  ylab(expression("Ratio of post/pre-BM mass"))+
  xlab("Temperature (˚C)") +
  theme_pubr()+
  theme(legend.position = "none")+
  geom_jitter(data=percentdiff_values, aes(x=Temperature,y=post_over_pre),#color=ZOI_italic$Technical_Rep,
              position = "jitter",size=1)+
  scale_fill_manual(values= c("#4D6FAE","#6F9F51", "#CC763B"))+
  theme(panel.background = element_rect(fill = NA, color = "black"))+
  theme(panel.spacing = unit(0.5, "lines"))
postpreratio_mass_BM1_bytemp
```

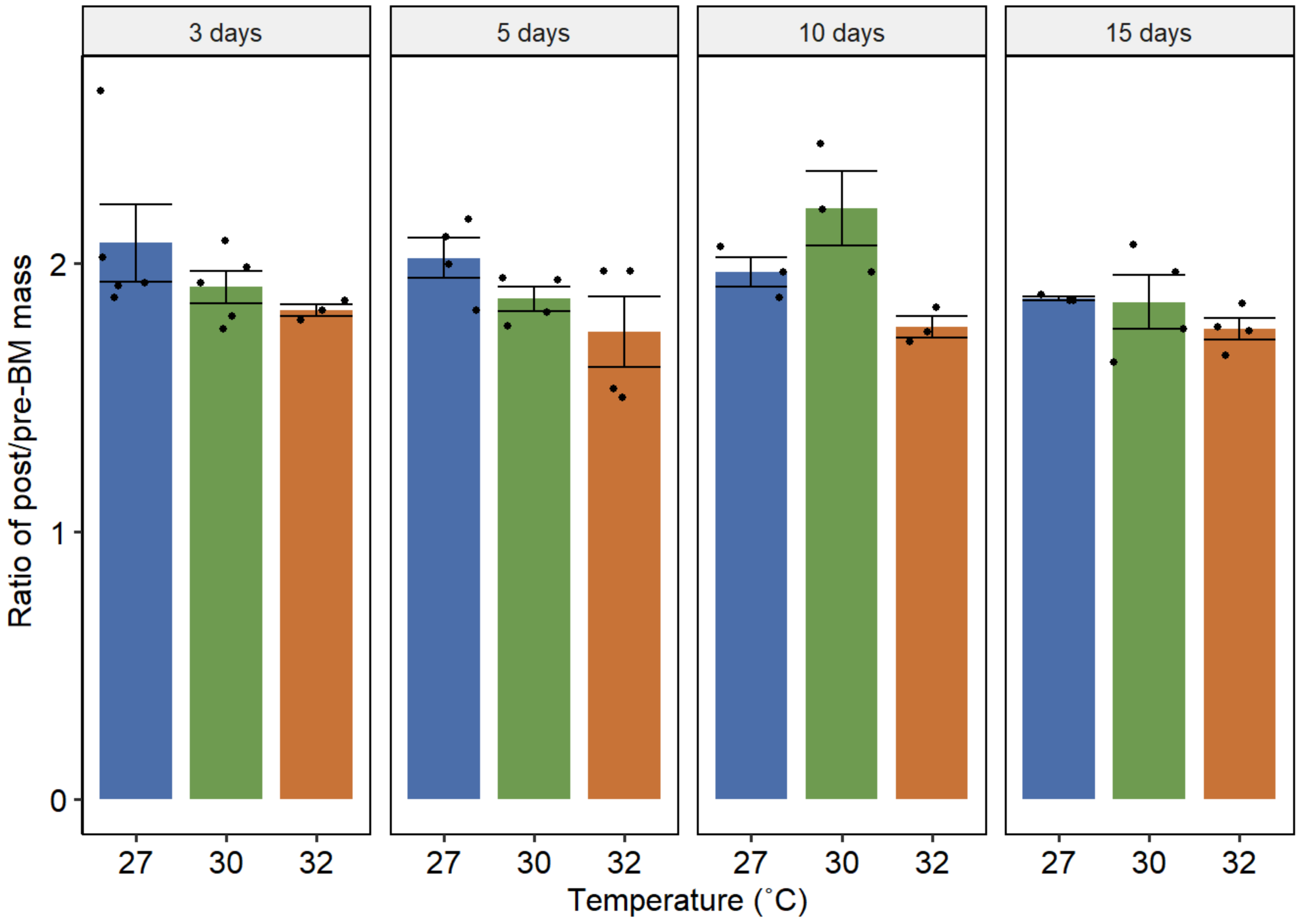

3 days
5 days
10 days
15 days
Ratio of post/pre-BM mass
0
1
2
27
30
32
Temperature (˚C)

```
ggsave("BM_size/postpreratio_mass_BM1_bytemp.png", plot=postpreratio_mass_BM1_bytemp,width = 6, height = 4,
units = "in", dpi = 600)
ggsave("BM_size/postpreratio_mass_BM1_bytemp.pdf", plot=postpreratio_mass_BM1_bytemp,width = 6, height = 4,
units = "in", dpi = 600)

################################################################################
################################################################################

#main effects plots:
BM1_percentdiff_summary_TEMP <- percentdiff_values %>%
  group_by(Temperature) %>%
  summarise(mean_pre = mean(pre_mass_value),
            mean_post = mean(post_mass_value),
            mean_postminuspre = mean(post_minus_pre),
            mean_percentdiff = mean(percent_diff),
            mean_postoverpre = mean(post_over_pre),
            median_pre = median(pre_mass_value),
            median_post = median(post_mass_value),
            median_postminuspre = median(post_minus_pre),
            median_percentdiff = median(percent_diff),
            median_postoverpre = median(post_over_pre),
            n_mass = n(),
            sd_pre = sd(pre_mass_value),
            sd_post = sd(post_mass_value),
            sd_postminuspre = sd(post_minus_pre),
            sd_percentdiff = sd(percent_diff),
            sd_postoverpre = sd(post_over_pre),
            SE_pre = sd(pre_mass_value)/sqrt(n()),
            SE_post = sd(post_mass_value)/sqrt(n()),
            SE_postminuspre = sd(post_minus_pre)/sqrt(n()),
            SE_percentdiff = sd(percent_diff)/sqrt(n()),
            SE_postoverpre = sd(post_over_pre)/sqrt(n()))

BM1_percentdiff_summary_TEMP <- as.data.frame(BM1_percentdiff_summary_TEMP)
BM1_percentdiff_summary_TEMP$Temperature <- as.factor(BM1_percentdiff_summary_TEMP$Temperature)
write_xlsx(BM1_percentdiff_summary_TEMP, "BM_size/BM1_BMsize_summary_TEMP.xlsx")

BM1_percentdiff_summary_AGE <- percentdiff_values %>%
  group_by(Age) %>%
  summarise(mean_pre = mean(pre_mass_value),
            mean_post = mean(post_mass_value),
            mean_postminuspre = mean(post_minus_pre),
            mean_percentdiff = mean(percent_diff),
            mean_postoverpre = mean(post_over_pre),
            median_pre = median(pre_mass_value),
            median_post = median(post_mass_value),
            median_postminuspre = median(post_minus_pre),
            median_percentdiff = median(percent_diff),
            median_postoverpre = median(post_over_pre),
            n_mass = n(),
            sd_pre = sd(pre_mass_value),
            sd_post = sd(post_mass_value),
            sd_postminuspre = sd(post_minus_pre),
            sd_percentdiff = sd(percent_diff),
            sd_postoverpre = sd(post_over_pre),
            SE_pre = sd(pre_mass_value)/sqrt(n()),
```

```
            SE_post = sd(post_mass_value)/sqrt(n()),
            SE_postminuspre = sd(post_minus_pre)/sqrt(n()),
            SE_percentdiff = sd(percent_diff)/sqrt(n()),
            SE_postoverpre = sd(post_over_pre)/sqrt(n()))

BM1_percentdiff_summary_AGE <- as.data.frame(BM1_percentdiff_summary_AGE)
BM1_percentdiff_summary_AGE$Age <- as.factor(BM1_percentdiff_summary_AGE$Age)
write_xlsx(BM1_percentdiff_summary_AGE, "BM_size/BM1_BMsize_summary_AGE.xlsx")

###########
#plot ratio post/pre BM size:
BM1_percentdiff_summary_AGE$Age <- factor(BM1_percentdiff_summary_AGE$Age,
                                          labels = c("3","5","10","15"))
percentdiff_values$Age <- factor(percentdiff_values$Age,
                                 labels = c("3","5","10","15"))

postpreratio_mass_BM1_AGEonly <- BM1_percentdiff_summary_AGE %>%
  ggplot(aes(x=Age,y=mean_postoverpre,group=Age))+
  geom_bar(aes(fill=Age),
           stat = "identity",
           position = position_dodge(1),
           width = 0.8) +
  scale_shape_identity(guide="legend")+
  #facet_grid(~Temperature)+
  geom_errorbar(aes(ymin=mean_postoverpre - SE_postoverpre,
                    ymax=mean_postoverpre + SE_postoverpre),
                width=0.8,position=position_dodge(0.9),
                color="black")+
  ylab(expression("Ratio of post/pre-BM body mass"))+
  xlab("Adult age (days)") +
  theme_pubr()+
  theme(legend.position = "none")+
  geom_jitter(data=percentdiff_values, aes(x=Age,y=post_over_pre),#color=ZOI_italic$Technical_Rep,
              position = "jitter",size=1)+
  scale_fill_manual(values= c("#DCD1E9","#BAA4D3","#9776BE","#7549A8"))+
  theme(panel.background = element_rect(fill = NA, color = "black"))+
  theme(panel.spacing = unit(0.5, "lines"))
postpreratio_mass_BM1_AGEonly
```

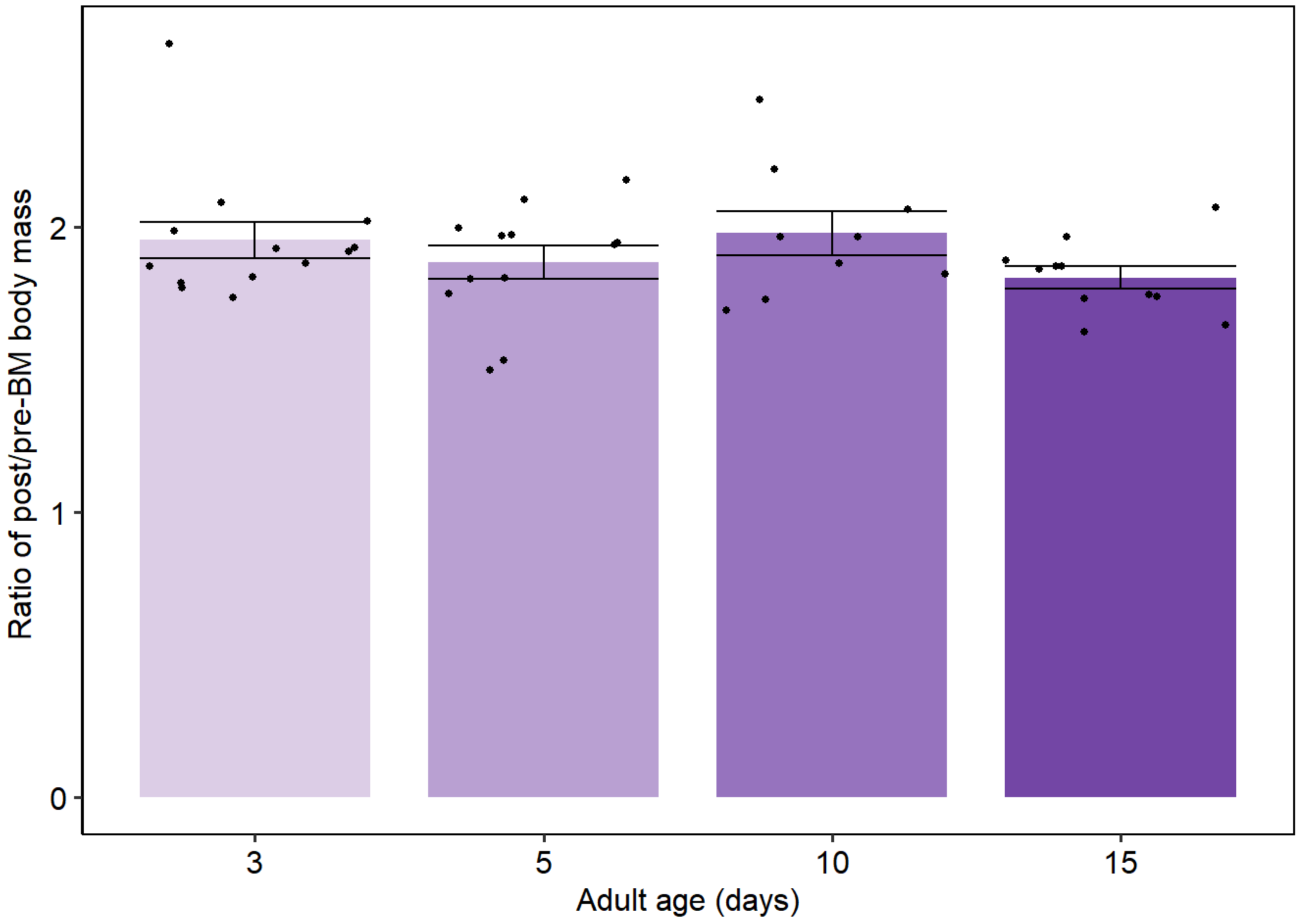

Ratio of post/pre-BM body mass
0
1
2
3
5
10
15
Adult age (days)

```
ggsave("BM_size/postpreratio_mass_BM1_AGEonly.png", plot=postpreratio_mass_BM1_AGEonly,width = 4, height =
4, units = "in", dpi = 600)
ggsave("BM_size/postpreratio_mass_BM1_AGEonly.pdf", plot=postpreratio_mass_BM1_AGEonly,width = 4, height =
4, units = "in", dpi = 600)




BM1_percentdiff_summary_TEMP$Temperature <- factor(BM1_percentdiff_summary_TEMP$Temperature,
                                                   labels = c("27","30","32"))
percentdiff_values$Temperature <- factor(percentdiff_values$Temperature,
                                         labels = c("27","30","32"))

postpreratio_mass_BM1_TEMPonly <- BM1_percentdiff_summary_TEMP%>%
  ggplot(aes(x=Temperature,y=mean_postoverpre,group=Temperature))+
  geom_bar(aes(fill=Temperature),
           stat = "identity",
           position = position_dodge(1),
           width = 0.8) +
  scale_shape_identity(guide="legend")+
  geom_errorbar(aes(ymin=mean_postoverpre - SE_postoverpre,
                    ymax=mean_postoverpre + SE_postoverpre),
                width=0.8,position=position_dodge(0.9),
                color="black")+
  ylab(expression("Ratio of post/pre-BM body mass"))+
  xlab("Temperature (˚C)") +
  theme_pubr()+
  theme(legend.position = "none")+
  geom_jitter(data=percentdiff_values, aes(x=Temperature,y=post_over_pre),#color=ZOI_italic$Technical_Rep,
              position = "jitter",size=1)+
  scale_fill_manual(values= c("#4D6FAE","#6F9F51", "#CC763B"))+
  theme(panel.background = element_rect(fill = NA, color = "black"))+
  theme(panel.spacing = unit(0.5, "lines"))
postpreratio_mass_BM1_TEMPonly
```

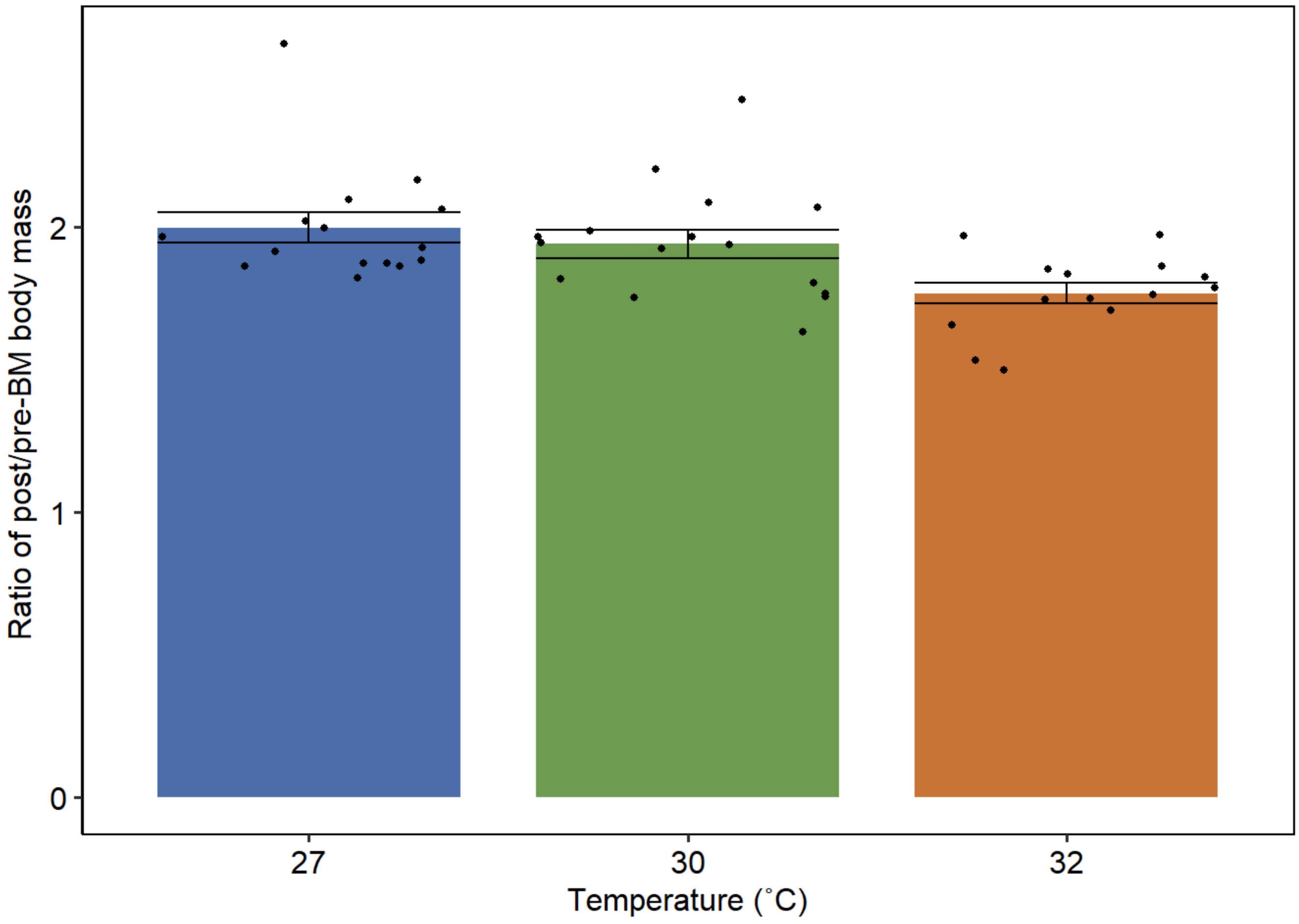


```
ggsave("BM_size/postpreratio_mass_BM1_TEMPonly.png", plot=postpreratio_mass_BM1_TEMPonly,width = 4, height
= 4, units = "in", dpi = 600)
ggsave("BM_size/postpreratio_mass_BM1_TEMPonly.pdf", plot=postpreratio_mass_BM1_TEMPonly,width = 4, height
= 4, units = "in", dpi = 600)
```

Analysis for Fig 2 data

```
#ANALYSIS
#ratio of post/pre BM mass
ggplot(data = percentdiff_values, aes(x = post_over_pre))+
  geom_density(color = "#FF6885", alpha = 0.6, bw = 1.5)+
  ylab("Density")+
  xlab("ratio post/pre")+
  theme_pubr()
```

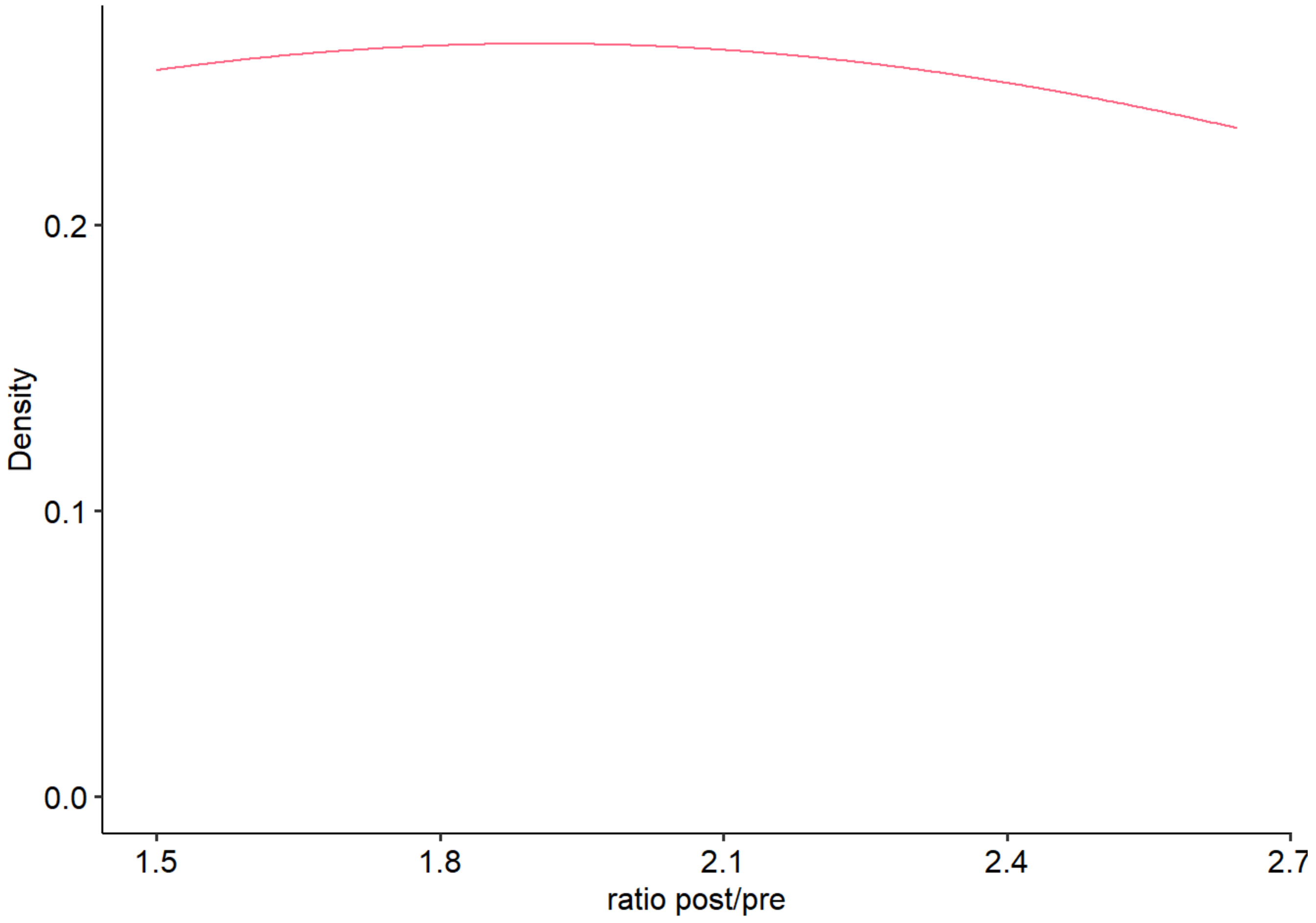


```
hist(percentdiff_values$post_over_pre)
```

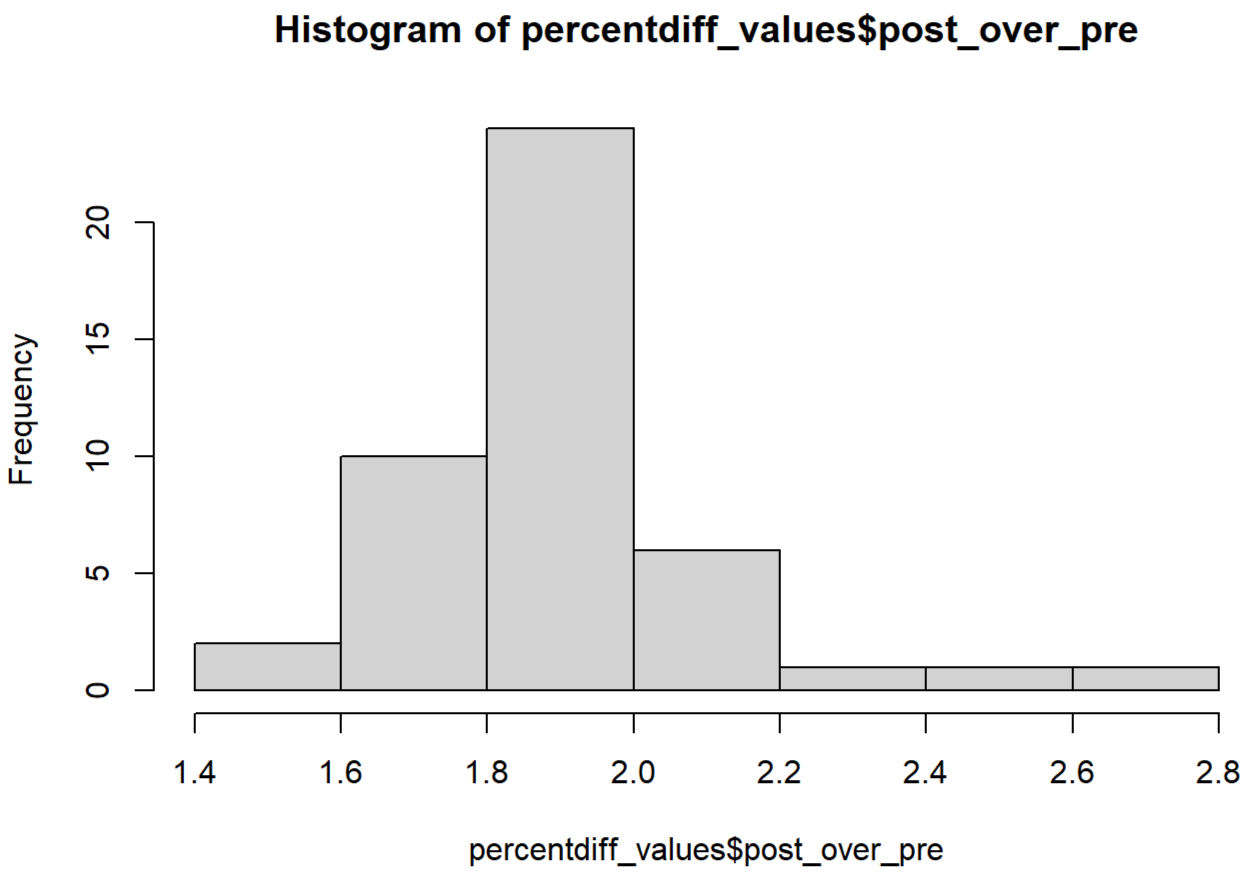

```
shapiro.test(percentdiff_values$post_over_pre)
```

```
##
##  Shapiro-Wilk normality test
##
## data:  percentdiff_values$post_over_pre
## W = 0.91555, p-value = 0.002997
```

```
percentdiff_values$log_post_over_pre <- log(percentdiff_values$post_over_pre)
shapiro.test(percentdiff_values$log_post_over_pre) #better
```

```
##
##  Shapiro-Wilk normality test
##
## data:  percentdiff_values$log_post_over_pre
## W = 0.95091, p-value = 0.05498
```

```
#ratio of post/pre BM mass
str(percentdiff_values) #temp and age are factors
```

```
## 'data.frame':    45 obs. of  9 variables:
##  $ Temperature        : Factor w/ 3 levels "27","30","32": 1 1 1 1 1 1 1 1 1 1 ...
##  $ Age                : Factor w/ 4 levels "3","5","10","15": 1 1 1 1 1 2 2 2 2 3 ...
##  $ Biological_Replicate: num  1 2 3 4 5 1 2 3 4 1 ...
##  $ pre_mass_value     : num  1.06 1.02 1.14 1.19 1.04 ...
##  $ post_mass_value    : num  2.81 1.96 2.31 2.29 1.94 ...
##  $ post_minus_pre     : num  1.749 0.938 1.169 1.105 0.907 ...
##  $ percent_diff       : num  164.4 91.5 102.2 92.9 87.5 ...
##  $ post_over_pre      : num  2.64 1.92 2.02 1.93 1.87 ...
##  $ log_post_over_pre  : num  0.972 0.65 0.704 0.657 0.628 ...
```

```
#model selection
library(glmmTMB)
model_postoverpre_1 = glmmTMB(log(post_over_pre) ~ Temperature+Age, data= percentdiff_values,family=gaussia
n(link="identity")) #equals model 1
model_postoverpre_2 = glmmTMB(log(post_over_pre) ~ Temperature*Age, data= percentdiff_values,family=gaussia
n(link="identity")) #equals model 1
model_postoverpre_3 = glmmTMB(log(post_over_pre) ~ Temperature+Age+(1|Biological_Replicate), data= percentd
iff_values,family=gaussian(link="identity")) #equals model 1
model_postoverpre_4 = glmmTMB(log(post_over_pre) ~ Temperature*Age+(1|Biological_Replicate), data= percentd
iff_values,family=gaussian(link="identity")) #equals model 1

summary(model_postoverpre_1)
```

```
##  Family: gaussian  ( identity )
## Formula:          log(post_over_pre) ~ Temperature + Age
## Data: percentdiff_values
##
##      AIC      BIC   logLik deviance df.resid
##    -80.6    -68.0     47.3    -94.6       38
##
##
## Dispersion estimate for gaussian family (sigma^2): 0.00715
##
## Conditional model:
##               Estimate Std. Error z value Pr(>|z|)
## (Intercept)    0.70214    0.02873  24.443  < 2e-16 ***
## Temperature30 -0.02691    0.03042  -0.885 0.376350
## Temperature32 -0.11630    0.03162  -3.678 0.000235 ***
## Age5          -0.03015    0.03396  -0.888 0.374576
## Age10          0.02222    0.03677   0.604 0.545621
## Age15         -0.05179    0.03485  -1.486 0.137281
## ---
## Signif. codes:  0 '***' 0.001 '**' 0.01 '*' 0.05 '.' 0.1 ' ' 1
```

```
summary(model_postoverpre_2)
```

```
##  Family: gaussian  ( identity )
## Formula:          log(post_over_pre) ~ Temperature * Age
## Data: percentdiff_values
##
##      AIC      BIC   logLik deviance df.resid
##    -77.0    -53.5     51.5   -103.0       32
##
##
## Dispersion estimate for gaussian family (sigma^2): 0.00594
##
## Conditional model:
##                      Estimate Std. Error z value Pr(>|z|)
## (Intercept)          0.722299   0.034474  20.952   <2e-16 ***
## Temperature30       -0.076486   0.048754  -1.569   0.1167
## Temperature32       -0.121016   0.056296  -2.150   0.0316 *
## Age5                -0.020860   0.051711  -0.403   0.6867
## Age10               -0.046282   0.056296  -0.822   0.4110
## Age15               -0.096467   0.056296  -1.714   0.0866 .
## Temperature30:Age5  -0.001127   0.073131  -0.015   0.9877
## Temperature32:Age5  -0.032927   0.078361  -0.420   0.6743
## Temperature30:Age10  0.187716   0.079615   2.358   0.0184 *
## Temperature32:Age10  0.011610   0.084444   0.137   0.8906
## Temperature30:Age15  0.064777   0.076441   0.847   0.3968
## Temperature32:Age15  0.056939   0.081459   0.699   0.4846
## ---
## Signif. codes:  0 '***' 0.001 '**' 0.01 '*' 0.05 '.' 0.1 ' ' 1
```

```
summary(model_postoverpre_3)
```

```
##  Family: gaussian  ( identity )
## Formula:
## log(post_over_pre) ~ Temperature + Age + (1 | Biological_Replicate)
## Data: percentdiff_values
##
##      AIC      BIC   logLik deviance df.resid
##    -78.6    -64.2     47.3    -94.6       37
##
## Random effects:
##
## Conditional model:
##  Groups               Name        Variance  Std.Dev.
##  Biological_Replicate (Intercept) 8.661e-12 2.943e-06
##  Residual                         7.147e-03 8.454e-02
## Number of obs: 45, groups:  Biological_Replicate, 5
##
## Dispersion estimate for gaussian family (sigma^2): 0.00715
##
## Conditional model:
##                Estimate Std. Error z value Pr(>|z|)
## (Intercept)    0.70214    0.02873  24.443  < 2e-16 ***
## Temperature30 -0.02691    0.03042  -0.885 0.376347
## Temperature32 -0.11630    0.03162  -3.678 0.000235 ***
## Age5          -0.03015    0.03396  -0.888 0.374574
## Age10          0.02222    0.03677   0.604 0.545630
## Age15         -0.05179    0.03485  -1.486 0.137278
## ---
## Signif. codes:  0 '***' 0.001 '**' 0.01 '*' 0.05 '.' 0.1 ' ' 1
```

```
summary(model_postoverpre_4)
```

```
##  Family: gaussian  ( identity )
## Formula:
## log(post_over_pre) ~ Temperature * Age + (1 | Biological_Replicate)
## Data: percentdiff_values
##
##      AIC      BIC   logLik deviance df.resid
##    -75.0    -49.7     51.5   -103.0       31
##
## Random effects:
##
## Conditional model:
##  Groups               Name        Variance  Std.Dev.
##  Biological_Replicate (Intercept) 2.238e-11 4.730e-06
##  Residual                         5.942e-03 7.709e-02
## Number of obs: 45, groups:  Biological_Replicate, 5
##
## Dispersion estimate for gaussian family (sigma^2): 0.00594
##
## Conditional model:
##                       Estimate Std. Error z value Pr(>|z|)
## (Intercept)           0.722299   0.034474  20.952   <2e-16 ***
## Temperature30        -0.076487   0.048754  -1.569   0.1167
## Temperature32        -0.121016   0.056296  -2.150   0.0316 *
## Age5                 -0.020861   0.051711  -0.403   0.6866
## Age10                -0.046283   0.056296  -0.822   0.4110
## Age15                -0.096467   0.056296  -1.714   0.0866 .
## Temperature30:Age5  -0.001127   0.073131  -0.015   0.9877
## Temperature32:Age5  -0.032927   0.078361  -0.420   0.6743
## Temperature30:Age10  0.187717   0.079615   2.358   0.0184 *
## Temperature32:Age10  0.011610   0.084444   0.137   0.8906
## Temperature30:Age15  0.064776   0.076441   0.847   0.3968
## Temperature32:Age15  0.056939   0.081459   0.699   0.4846
## ---
## Signif. codes:  0 '***' 0.001 '**' 0.01 '*' 0.05 '.' 0.1 ' ' 1
```

```
library(lmtest)
lrtest(model_postoverpre_1,model_postoverpre_2)
```

```
## Likelihood ratio test
##
## Model 1: log(post_over_pre) ~ Temperature + Age
## Model 2: log(post_over_pre) ~ Temperature * Age
##   #Df LogLik Df  Chisq Pr(>Chisq)
## 1   7 47.323
## 2  13 51.475  6 8.3046     0.2166
```

```
lrtest(model_postoverpre_1,model_postoverpre_3)#bio rep does not matter
```

```
## Likelihood ratio test
##
## Model 1: log(post_over_pre) ~ Temperature + Age
## Model 2: log(post_over_pre) ~ Temperature + Age + (1 | Biological_Replicate)
##   #Df LogLik Df Chisq Pr(>Chisq)
## 1   7 47.323
## 2   8 47.323  1     0     0.9999
```

```
lrtest(model_postoverpre_2,model_postoverpre_4) #bio rep does not matter
```

```
## Likelihood ratio test
##
## Model 1: log(post_over_pre) ~ Temperature * Age
## Model 2: log(post_over_pre) ~ Temperature * Age + (1 | Biological_Replicate)
##   #Df LogLik Df Chisq Pr(>Chisq)
## 1  13 51.475
## 2  14 51.475  1     0     0.9999
```

```
#same thing as lrtest:
anova(model_postoverpre_1,model_postoverpre_3)
```

```
## Data: percentdiff_values
## Models:
## model_postoverpre_1: log(post_over_pre) ~ Temperature + Age, zi=~0, disp=~1
## model_postoverpre_3: log(post_over_pre) ~ Temperature + Age + (1 | Biological_Replicate), zi=~0, disp=~1
##                     Df     AIC     BIC logLik deviance Chisq Chi Df Pr(>Chisq)
## model_postoverpre_1  7 -80.645 -67.999 47.323  -94.645
## model_postoverpre_3  8 -78.645 -64.192 47.323  -94.645     0      1          1
```

```
anova(model_postoverpre_1,model_postoverpre_2)
```

```
## Data: percentdiff_values
## Models:
## model_postoverpre_1: log(post_over_pre) ~ Temperature + Age, zi=~0, disp=~1
## model_postoverpre_2: log(post_over_pre) ~ Temperature * Age, zi=~0, disp=~1
##                     Df     AIC     BIC logLik deviance  Chisq Chi Df Pr(>Chisq)
## model_postoverpre_1  7 -80.645 -67.999 47.323  -94.645
## model_postoverpre_2 13 -76.950 -53.463 51.475 -102.950 8.3046      6     0.2166
```

```
anova(model_postoverpre_2,model_postoverpre_4)
```

```
## Data: percentdiff_values
## Models:
## model_postoverpre_2: log(post_over_pre) ~ Temperature * Age, zi=~0, disp=~1
## model_postoverpre_4: log(post_over_pre) ~ Temperature * Age + (1 | Biological_Replicate), zi=~0, disp=~1
##                     Df    AIC     BIC logLik deviance Chisq Chi Df Pr(>Chisq)
## model_postoverpre_2 13 -76.95 -53.463 51.475  -102.95
## model_postoverpre_4 14 -74.95 -49.657 51.475  -102.95     0      1          1
```

```
AIC(model_postoverpre_1,model_postoverpre_2,model_postoverpre_3,model_postoverpre_4)
```

```
##                     df       AIC
## model_postoverpre_1  7 -80.64545
## model_postoverpre_2 13 -76.95002
## model_postoverpre_3  8 -78.64545
## model_postoverpre_4 14 -74.95002
```

```
BIC(model_postoverpre_1,model_postoverpre_2,model_postoverpre_3,model_postoverpre_4)
```

```
##                     df       BIC
## model_postoverpre_1  7 -67.99881
## model_postoverpre_2 13 -53.46341
## model_postoverpre_3  8 -64.19215
## model_postoverpre_4 14 -49.65675
```

```
sink("BM_size/BM1size_lrtests_significance.txt")
lrtest(model_postoverpre_1,model_postoverpre_2)
lrtest(model_postoverpre_1,model_postoverpre_3)#bio rep does not matter
lrtest(model_postoverpre_2,model_postoverpre_4) #bio rep does not matter
sink()

#check residuals
library(DHARMa)
plot(simulateResiduals(model_postoverpre_1))
```

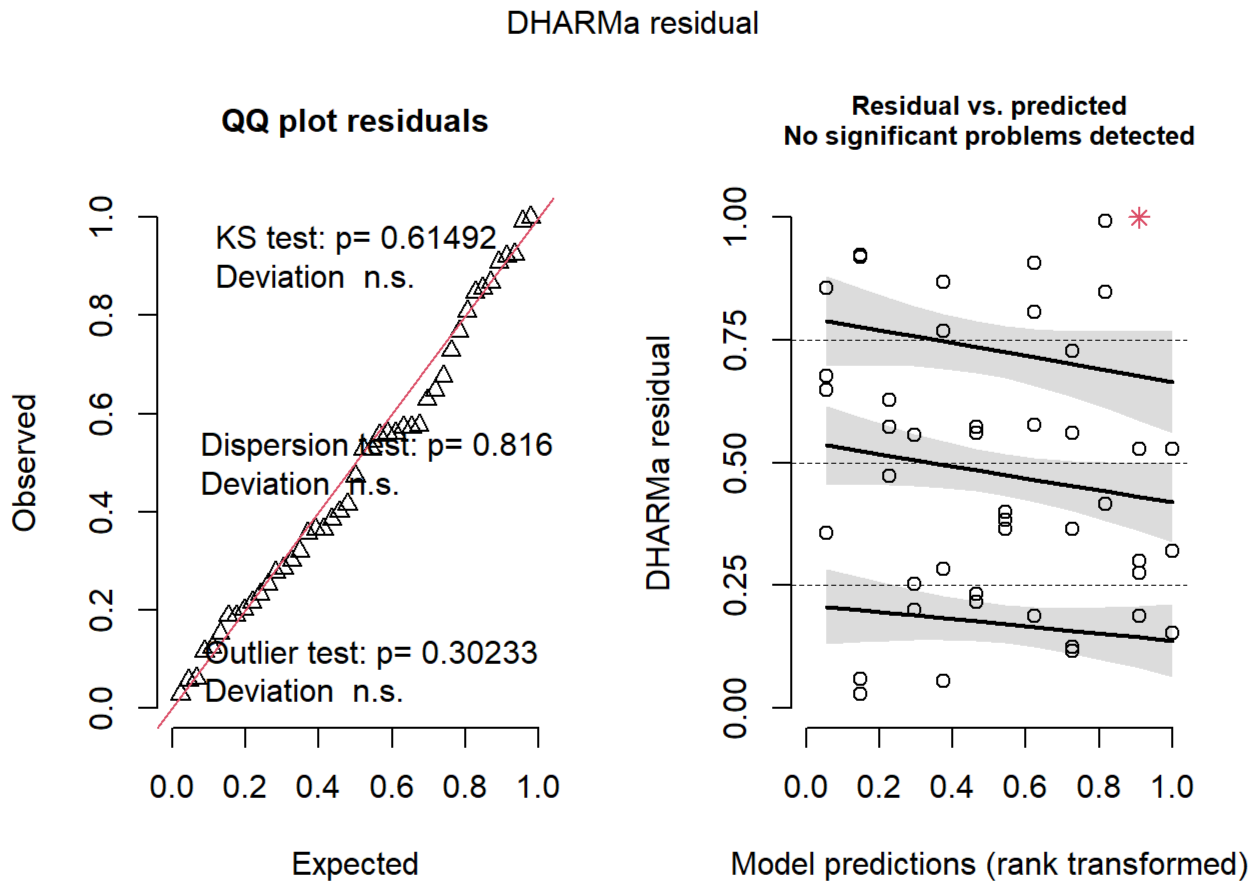

```
plot(simulateResiduals(model_postoverpre_2))
```

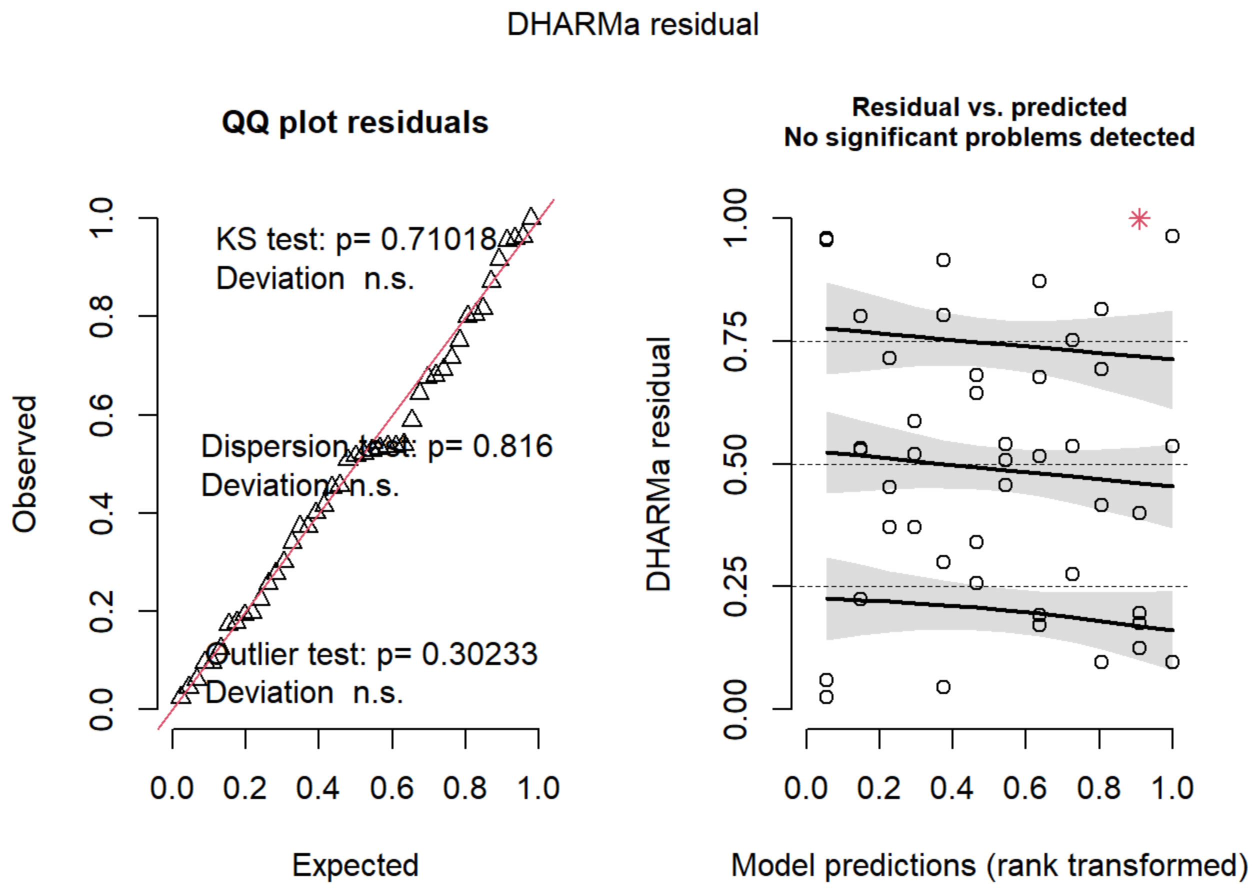


```
plot(simulateResiduals(model_postoverpre_3))
```

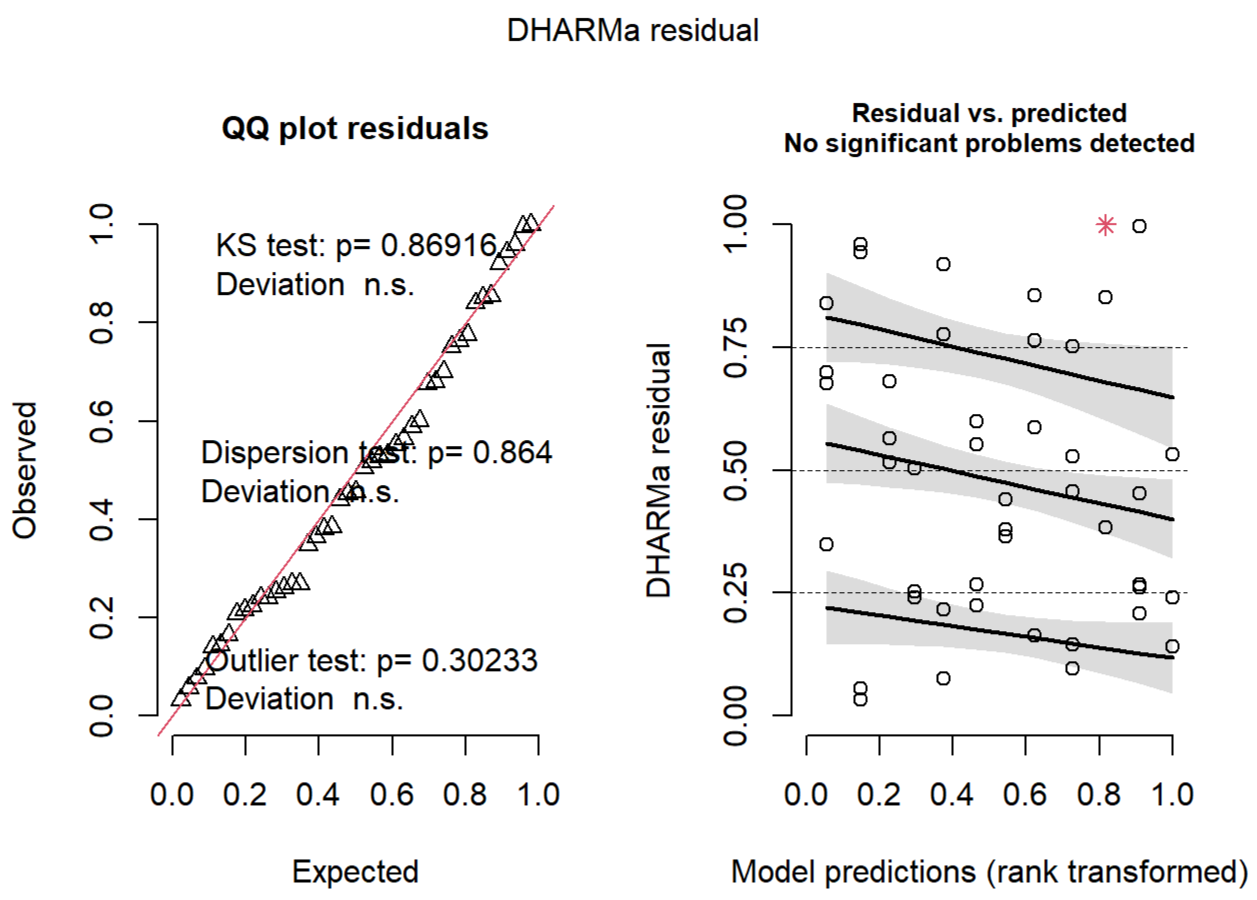


```
plot(simulateResiduals(model_postoverpre_4))
```

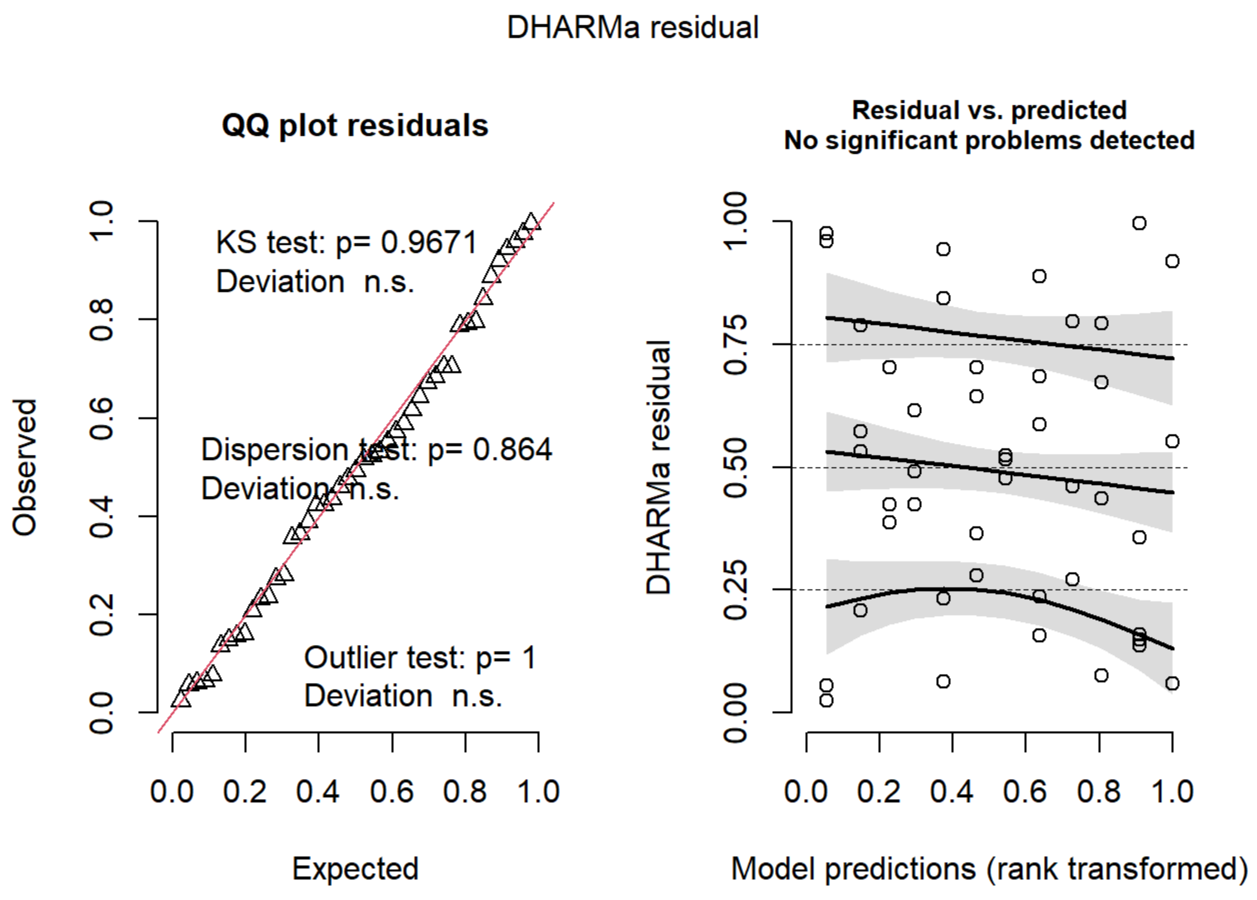

```
plot(fitted(model_postoverpre_2),
     residuals(model_postoverpre_2))
```

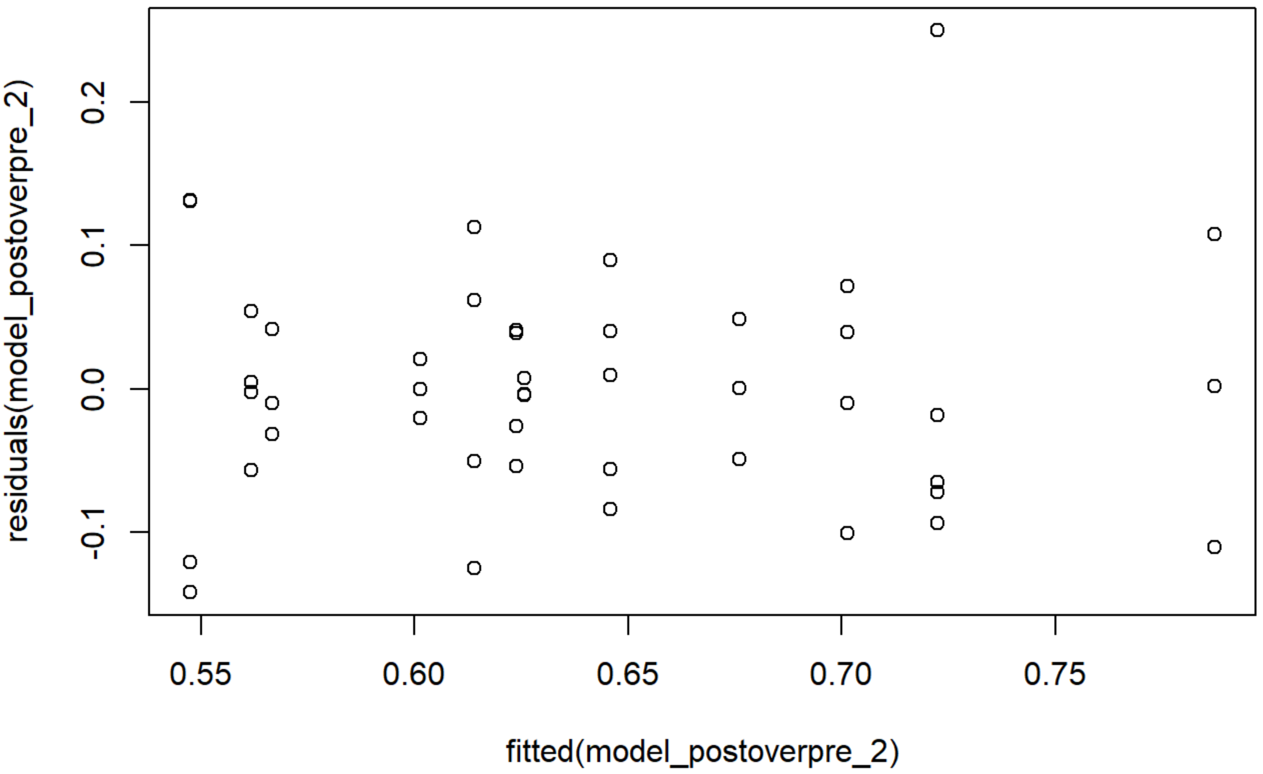


```
#2 is best for residuals
#go with 2 b/c this also matches our biological question of interest and is a good fit

#now check significance of factors in model
library(car)
anova_BM1size <- Anova(model_postoverpre_2,type=2)
anova_BM1size <- as.data.frame(anova_BM1size)
anova_BM1size
```

```
##                      Chisq Df   Pr(>Chisq)
## Temperature      17.716751  2 0.0001421859
## Age               5.520970  3 0.1373897748
## Temperature:Age   9.120143  6 0.1669331974
```

```
write_xlsx(anova_BM1size,"BM_Size/BM1Size_postoverpre_ANOVA.xlsx")

sink("BM_Size/BM1Size_postoverpre_ANOVA.txt")
Anova(model_postoverpre_2,type=2)
sink()

sink("BM_Size/BM1size_postoverpre_modelsummary.txt")
summary(model_postoverpre_2)
sink()

#effects and post hoc
library(effects)
ae <- allEffects(model_postoverpre_2)
plot(ae)
```

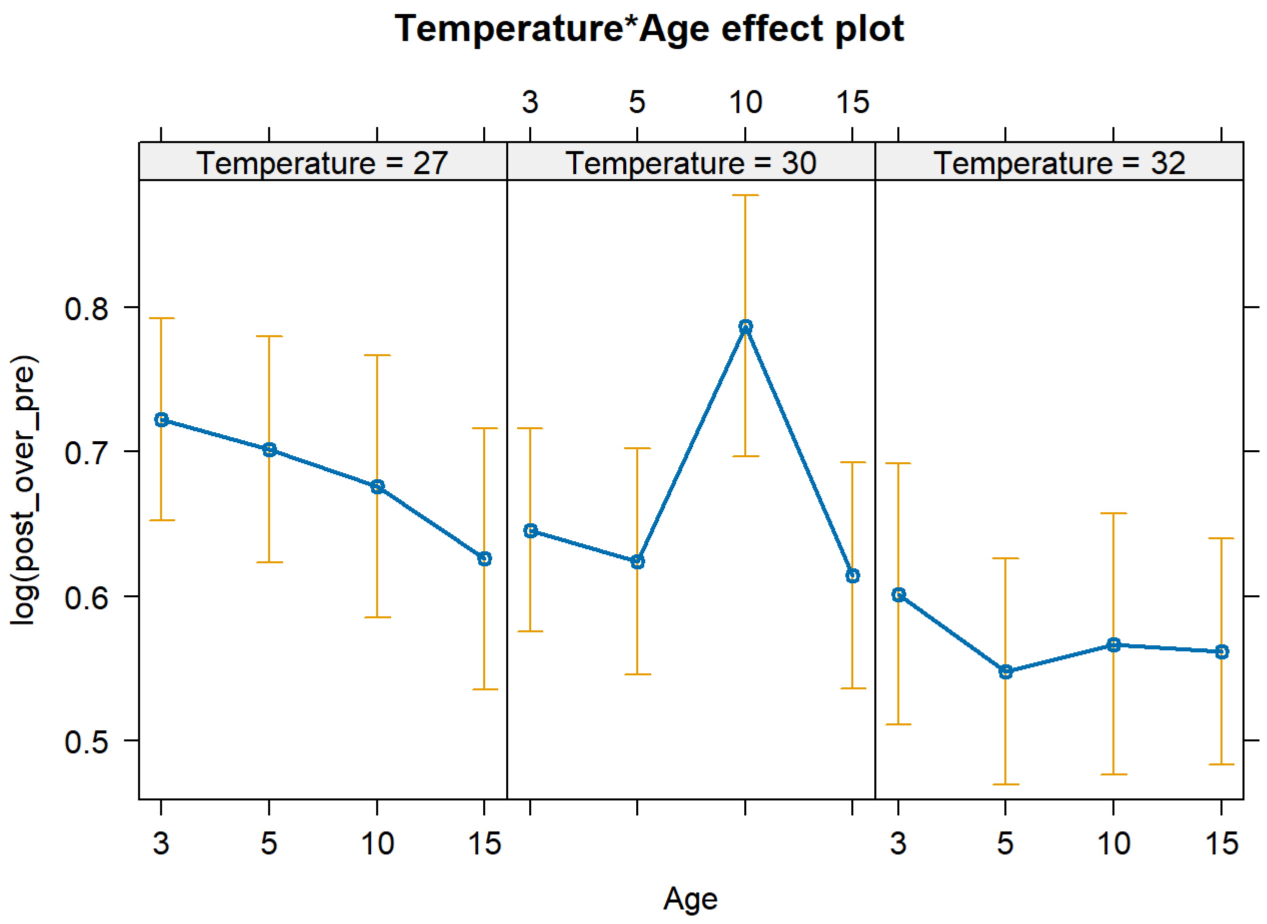


```
ref_grid(model_postoverpre_2)
```

```
## 'emmGrid' object with variables:
##     Temperature = 27, 30, 32
##     Age = 3, 5, 10, 15
## Transformation: "log"
```

```r
#post hoc comparisons:
#emmeans:
library(emmeans)
BM1postoverpre_emmeans <- emmeans(model_postoverpre_2,specs=c("Temperature","Age"),type="response")
BM1postoverpre_emmeans <- as.data.frame(BM1postoverpre_emmeans)
head(BM1postoverpre_emmeans)
```

```
##  Temperature Age response         SE df lower.CL upper.CL
##  27          3   2.059161 0.07098775 32 1.919524 2.208957
##  30          3   1.907536 0.06576062 32 1.778181 2.046302
##  32          3   1.824458 0.08119910 32 1.666336 1.997584
##  27          5   2.016652 0.07772827 32 1.864380 2.181360
##  30          5   1.866053 0.07192372 32 1.725153 2.018462
##  32          5   1.728918 0.06663807 32 1.598372 1.870126
##
## Confidence level used: 0.95
## Intervals are back-transformed from the log scale
```

```r
#save and re-run to make emmeans object again:
write_xlsx(BM1postoverpre_emmeans, "BM_size/BM1postoverpre_emmeans_all.xlsx")
BM1postoverpre_emmeans <- emmeans(model_postoverpre_2,specs=c("Temperature","Age"),type="response")

BM1postoverpre_emmeans_pwc <- pairs(BM1postoverpre_emmeans,adjust="sidak")
BM1postoverpre_emmeans_pwc <- as.data.frame(BM1postoverpre_emmeans_pwc)
write_xlsx(BM1postoverpre_emmeans_pwc, "BM_size/BM1postoverpre_emmeans_pairwisecontrasts.xlsx")
BM1postoverpre_emmeans_pwc
```

```
##  contrast                                      ratio         SE df null t.ratio
##  Temperature27 Age3 / Temperature30 Age3   1.0794873 0.05262906 32    1   1.569
##  Temperature27 Age3 / Temperature32 Age3   1.1286430 0.06353807 32    1   2.150
##  Temperature27 Age3 / Temperature27 Age5   1.0210792 0.05280120 32    1   0.403
##  Temperature27 Age3 / Temperature30 Age5   1.1034847 0.05706248 32    1   1.904
##  Temperature27 Age3 / Temperature32 Age5   1.1910117 0.06158860 32    1   3.380
##  Temperature27 Age3 / Temperature27 Age10  1.0473695 0.05896270 32    1   0.822
##  Temperature27 Age3 / Temperature30 Age10  0.9371165 0.05275590 32    1  -1.154
##  Temperature27 Age3 / Temperature32 Age10  1.1684611 0.06577967 32    1   2.766
##  Temperature27 Age3 / Temperature27 Age15  1.1012727 0.06199723 32    1   1.714
##  Temperature27 Age3 / Temperature30 Age15  1.1142433 0.05761882 32    1   2.092
##  Temperature27 Age3 / Temperature32 Age15  1.1741494 0.06071664 32    1   3.105
##  Temperature30 Age3 / Temperature32 Age3   1.0455361 0.05885949 32    1   0.791
##  Temperature30 Age3 / Temperature27 Age5   0.9458928 0.04891322 32    1  -1.076
##  Temperature30 Age3 / Temperature30 Age5   1.0222303 0.05286072 32    1   0.425
##  Temperature30 Age3 / Temperature32 Age5   1.1033124 0.05705357 32    1   1.901
##  Temperature30 Age3 / Temperature27 Age10  0.9702472 0.05462102 32    1  -0.537
##  Temperature30 Age3 / Temperature30 Age10  0.8681126 0.04887125 32    1  -2.512
##  Temperature30 Age3 / Temperature32 Age10  1.0824222 0.06093603 32    1   1.407
##  Temperature30 Age3 / Temperature27 Age15  1.0201812 0.05743211 32    1   0.355
##  Temperature30 Age3 / Temperature30 Age15  1.0321967 0.05337609 32    1   0.613
##  Temperature30 Age3 / Temperature32 Age15  1.0876917 0.05624581 32    1   1.626
##  Temperature32 Age3 / Temperature27 Age5   0.9046964 0.05326468 32    1  -1.701
##  Temperature32 Age3 / Temperature30 Age5   0.9777092 0.05756337 32    1  -0.383
##  Temperature32 Age3 / Temperature32 Age5   1.0552599 0.06212922 32    1   0.914
##  Temperature32 Age3 / Temperature27 Age10  0.9279901 0.05840846 32    1  -1.187
##  Temperature32 Age3 / Temperature30 Age10  0.8303038 0.05226001 32    1  -2.955
##  Temperature32 Age3 / Temperature32 Age10  1.0352796 0.06516135 32    1   0.551
##  Temperature32 Age3 / Temperature27 Age15  0.9757494 0.06141447 32    1  -0.390
##  Temperature32 Age3 / Temperature30 Age15  0.9872415 0.05812459 32    1  -0.218
##  Temperature32 Age3 / Temperature32 Age15  1.0403196 0.06124960 32    1   0.671
##  Temperature27 Age5 / Temperature30 Age5   1.0807043 0.05890741 32    1   1.424
##  Temperature27 Age5 / Temperature32 Age5   1.1664244 0.06357987 32    1   2.824
##  Temperature27 Age5 / Temperature27 Age10  1.0257475 0.06039166 32    1   0.432
##  Temperature27 Age5 / Temperature30 Age10  0.9177706 0.05403444 32    1  -1.457
##  Temperature27 Age5 / Temperature32 Age10  1.1443393 0.06737383 32    1   2.290
##  Temperature27 Age5 / Temperature27 Age15  1.0785380 0.06349973 32    1   1.284
##  Temperature27 Age5 / Temperature30 Age15  1.0912407 0.05948174 32    1   1.602
##  Temperature27 Age5 / Temperature32 Age15  1.1499102 0.06267971 32    1   2.563
##  Temperature30 Age5 / Temperature32 Age5   1.0793188 0.05883189 32    1   1.400
##  Temperature30 Age5 / Temperature27 Age10  0.9491473 0.05588176 32    1  -0.886
##  Temperature30 Age5 / Temperature30 Age10  0.8492338 0.04999928 32    1  -2.776
##  Temperature30 Age5 / Temperature32 Age10  1.0588829 0.06234253 32    1   0.972
##  Temperature30 Age5 / Temperature27 Age15  0.9979955 0.05875773 32    1  -0.034
##  Temperature30 Age5 / Temperature30 Age15  1.0097496 0.05503979 32    1   0.178
##  Temperature30 Age5 / Temperature32 Age15  1.0640378 0.05799895 32    1   1.139
##  Temperature32 Age5 / Temperature27 Age10  0.8793948 0.05177503 32    1  -2.183
##  Temperature32 Age5 / Temperature30 Age10  0.7868239 0.04632485 32    1  -4.072
##  Temperature32 Age5 / Temperature32 Age10  0.9810659 0.05776099 32    1  -0.325
##  Temperature32 Age5 / Temperature27 Age15  0.9246531 0.05443965 32    1  -1.331
##  Temperature32 Age5 / Temperature30 Age15  0.9355435 0.05099493 32    1  -1.222
##  Temperature32 Age5 / Temperature32 Age15  0.9858420 0.05373663 32    1  -0.262
##  Temperature27 Age10 / Temperature30 Age10 0.8947334 0.05631526 32    1  -1.767
##  Temperature27 Age10 / Temperature32 Age10 1.1156149 0.07021772 32    1   1.738
##  Temperature27 Age10 / Temperature27 Age15 1.0514653 0.06618009 32    1   0.797
##  Temperature27 Age10 / Temperature30 Age15 1.0638492 0.06263492 32    1   1.051
```

```
##  Temperature27 Age10 / Temperature32 Age15 1.1210460 0.06600243 32    1   1.941
##  Temperature30 Age10 / Temperature32 Age10 1.2468685 0.07847893 32    1   3.505
##  Temperature30 Age10 / Temperature27 Age15 1.1751716 0.07396627 32    1   2.565
##  Temperature30 Age10 / Temperature30 Age15 1.1890125 0.07000400 32    1   2.940
##  Temperature30 Age10 / Temperature32 Age15 1.2529385 0.07376770 32    1   3.830
##  Temperature32 Age10 / Temperature27 Age15 0.9424984 0.05932163 32    1  -0.941
##  Temperature32 Age10 / Temperature30 Age15 0.9535990 0.05614386 32    1  -0.807
##  Temperature32 Age10 / Temperature32 Age15 1.0048683 0.05916237 32    1   0.082
##  Temperature27 Age15 / Temperature30 Age15 1.0117778 0.05956918 32    1   0.199
##  Temperature27 Age15 / Temperature32 Age15 1.0661750 0.06277185 32    1   1.088
##  Temperature30 Age15 / Temperature32 Age15 1.0537640 0.05743894 32    1   0.961
##  p.value
##   0.9999
##   0.9288
##   1.0000
##   0.9889
##   0.1192
##   1.0000
##   1.0000
##   0.4623
##   0.9987
##   0.9503
##   0.2309
##   1.0000
##   1.0000
##   1.0000
##   0.9892
##   1.0000
##   0.6825
##   1.0000
##   1.0000
##   1.0000
##   0.9997
##   0.9989
##   1.0000
##   1.0000
##   1.0000
##   0.3202
##   1.0000
##   1.0000
##   1.0000
##   1.0000
##   1.0000
##   0.4150
##   1.0000
##   1.0000
##   0.8542
##   1.0000
##   0.9998
##   0.6385
##   1.0000
##   1.0000
##   0.4539
##   1.0000
##   1.0000
##   1.0000
```

```
##   1.0000
##   0.9140
##   0.0187
##   1.0000
##   1.0000
##   1.0000
##   1.0000
##   0.9975
##   0.9983
##   1.0000
##   1.0000
##   0.9845
##   0.0867
##   0.6368
##   0.3297
##   0.0365
##   1.0000
##   1.0000
##   1.0000
##   1.0000
##   1.0000
##   1.0000
##
## P value adjustment: sidak method for 66 tests
## Tests are performed on the log scale
```

```
BM1postoverpre_emmeans_TEMP <- emmeans(model_postoverpre_2,specs=c("Temperature"),
                                       type="response")
```

```
## NOTE: Results may be misleading due to involvement in interactions
```

```
BM1postoverpre_emmeans_TEMP
```

```
##  Temperature response     SE df lower.CL upper.CL
##  27              1.98 0.0403 32     1.90     2.06
##  30              1.95 0.0382 32     1.87     2.03
##  32              1.77 0.0368 32     1.69     1.84
##
## Results are averaged over the levels of: Age
## Confidence level used: 0.95
## Intervals are back-transformed from the log scale
```

```
BM1postoverpre_emmeans_TEMP_pairs <- pairs(BM1postoverpre_emmeans_TEMP, adjust="sidak")
BM1postoverpre_emmeans_TEMP_pairs
```

```
##  contrast                      ratio     SE df null t.ratio p.value
##  Temperature27 / Temperature30  1.01 0.0286 32    1   0.483  0.9504
##  Temperature27 / Temperature32  1.12 0.0326 32    1   3.850  0.0016
##  Temperature30 / Temperature32  1.10 0.0315 32    1   3.445  0.0048
## 
## Results are averaged over the levels of: Age 
## P value adjustment: sidak method for 3 tests 
## Tests are performed on the log scale
```

```
BM1postoverpre_emmeans_TEMP_pairs <- as.data.frame(BM1postoverpre_emmeans_TEMP_pairs)
write_xlsx(BM1postoverpre_emmeans_TEMP_pairs, "BM_size/BM1postoverpre_emmeans_TEMP_pairs.xlsx")

BM1postoverpre_emmeans_AGE <- emmeans(model_postoverpre_2,specs=c("Age"),
                                      type="response")
```

```
## NOTE: Results may be misleading due to involvement in interactions
```

```
BM1postoverpre_emmeans_AGE
```

```
##  Age response     SE df lower.CL upper.CL
##  3       1.93 0.0424 32     1.84     2.02
##  5       1.87 0.0415 32     1.78     1.95
##  10      1.97 0.0505 32     1.87     2.07
##  15      1.82 0.0428 32     1.74     1.91
## 
## Results are averaged over the levels of: Temperature 
## Confidence level used: 0.95 
## Intervals are back-transformed from the log scale
```

```
BM1postoverpre_emmeans_AGE_pairs <- pairs(BM1postoverpre_emmeans_AGE, adjust="sidak")
BM1postoverpre_emmeans_AGE_pairs <- as.data.frame(BM1postoverpre_emmeans_AGE_pairs)
write_xlsx(BM1postoverpre_emmeans_AGE_pairs, "BM_size/BM1postoverpre_emmeans_AGE_pairs.xlsx")

sink("BM_Size/modelparameters_BM1size_postoverpre.txt")
parameters::model_parameters(
  model_postoverpre_2, exponentiate = TRUE, ci_method = "wald",
  effects = "all",
  component = "conditional",
  group_level = TRUE,
  verbose = FALSE
)
sink()
```

Code for Figure 3 - survival

Import data and calculate survival time.

```
################

# clear existing workspace
rm(list = ls(all = TRUE))
graphics.off()
shell("cls")

#set wd to your project folder
getwd() #check working directory
```

```
## [1] "C:/Users/linzm/OneDrive - Vanderbilt/Hillyer_Lab/Blood_feeding_project/Bloodfeeding"
```

```
###########################
#####################
#load libraries needed:
library(readxl)
library(writexl)
library(tidyverse)
library(rstatix)
library(car)
library(ggpubr)
library(survminer)
```

```
##
## Attaching package: 'survminer'
```

```
## The following object is masked from 'package:survival':
##
##     myeloma
```

```
library(survival)
library(emmeans)

library(lubridate)
library(ggsurvfit)
library(gtsummary)
```

```
##
## Attaching package: 'gtsummary'
```

```
## The following object is masked from 'package:MASS':
##
##     select
```

```
library(tidycmprsk)
```

```
##
## Attaching package: 'tidycmprsk'
```

```
## The following object is masked from 'package:gtsummary':
##
##     trial
```

```
#install.packages("remotes")
#library(remotes)
#remotes::install_github("zabore/condsurv")
library(condsurv)

library(knitr)
```

```
## Warning: package 'knitr' was built under R version 4.4.2
```

```
library(dplyr)
library(survival)
library(ggplot2)
library(tibble)
library(condsurv)
library(lubridate)
library(ggsurvfit)
library(survival)
library(survminer)
library(coxphw)
library(survminer)
library(forestplot)
```

```
## Loading required package: grid
```

```
## Loading required package: checkmate
```

```
## Loading required package: abind
```

```
library(gridExtra)
```

```
##
## Attaching package: 'gridExtra'
```

```
## The following object is masked from 'package:dplyr':
##
##     combine
```

```
#######################################################
#import the data and clean it up:
#import the data:
bucket_survival_data <- read_xlsx("SupplementaryData1_RawData.xlsx",
                             sheet = "Fig3")
bucket_survival_data <- as.data.frame(bucket_survival_data)
str(bucket_survival_data) #make sure dates are dates
```

```
## 'data.frame':    1398 obs. of  13 variables:
##  $ ID_overall      : num  1 2 3 4 5 6 7 8 9 10 ...
##  $ Temperature     : num  27 27 27 27 27 27 27 27 27 27 ...
##  $ Age             : num  3 3 3 3 3 3 3 3 3 3 ...
##  $ Age_of_BM       : num  3 3 3 3 3 3 3 3 3 3 ...
##  $ ID_per_group    : num  1 2 3 4 5 6 7 8 9 10 ...
##  $ Trial_start_date: POSIXct, format: "2024-02-07" "2024-02-07" ...
##  $ Date_Group      : chr  "A" "A" "A" "A" ...
##  $ Trial_number    : num  1 1 1 1 1 1 1 1 1 1 ...
##  $ Initial_n       : num  35 35 35 35 35 35 35 35 35 35 ...
##  $ BM1_Date        : POSIXct, format: "2024-02-07" "2024-02-07" ...
##  $ Bloodmeal_number: num  1 1 1 1 1 1 1 1 1 1 ...
##  $ Date_of_death   : POSIXct, format: "2024-02-15" "2024-02-16" ...
##  $ Censor          : num  1 1 1 1 1 1 1 1 1 1 ...
```

```
head(bucket_survival_data)
```

```
##   ID_overall Temperature Age Age_of_BM ID_per_group Trial_start_date Date_Group
## 1          1          27   3         3            1       2024-02-07          A
## 2          2          27   3         3            2       2024-02-07          A
## 3          3          27   3         3            3       2024-02-07          A
## 4          4          27   3         3            4       2024-02-07          A
## 5          5          27   3         3            5       2024-02-07          A
## 6          6          27   3         3            6       2024-02-07          A
##   Trial_number Initial_n   BM1_Date Bloodmeal_number Date_of_death Censor
## 1            1        35 2024-02-07                1    2024-02-15      1
## 2            1        35 2024-02-07                1    2024-02-16      1
## 3            1        35 2024-02-07                1    2024-02-16      1
## 4            1        35 2024-02-07                1    2024-02-16      1
## 5            1        35 2024-02-07                1    2024-02-17      1
## 6            1        35 2024-02-07                1    2024-02-18      1
```

```
#calculate days to death and age of death:
bucket_survival_data$Days_to_death_post_BM <- bucket_survival_data$Date_of_death - bucket_survival_data$Tri
al_start_date
bucket_survival_data$Age_of_death <- bucket_survival_data$Age_of_BM + bucket_survival_data$Days_to_death_po
st_BM

bucket_survival_data <-
  bucket_survival_data %>%
  mutate(
    Age_of_BM_days = as.difftime(Age_of_BM, unit="days")
  )
bucket_survival_data$Date_of_eclosion <- bucket_survival_data$Trial_start_date - (bucket_survival_data$Age_
of_BM_days)

#days alive post BM and post eclosion:
bucket_survival_data <-
  bucket_survival_data %>%
  mutate(
    days_alive_post_BM = as.duration(Trial_start_date %--% Date_of_death) / ddays(1),
    days_alive_post_eclosion = as.duration(Date_of_eclosion %--% Date_of_death) / ddays(1),
  )

str(bucket_survival_data)
```

```
## 'data.frame':    1398 obs. of  19 variables:
##  $ ID_overall              : num  1 2 3 4 5 6 7 8 9 10 ...
##  $ Temperature             : num  27 27 27 27 27 27 27 27 27 27 ...
##  $ Age                     : num  3 3 3 3 3 3 3 3 3 3 ...
##  $ Age_of_BM               : num  3 3 3 3 3 3 3 3 3 3 ...
##  $ ID_per_group            : num  1 2 3 4 5 6 7 8 9 10 ...
##  $ Trial_start_date        : POSIXct, format: "2024-02-07" "2024-02-07" ...
##  $ Date_Group              : chr  "A" "A" "A" "A" ...
##  $ Trial_number            : num  1 1 1 1 1 1 1 1 1 1 ...
##  $ Initial_n               : num  35 35 35 35 35 35 35 35 35 35 ...
##  $ BM1_Date                : POSIXct, format: "2024-02-07" "2024-02-07" ...
##  $ Bloodmeal_number        : num  1 1 1 1 1 1 1 1 1 1 ...
##  $ Date_of_death           : POSIXct, format: "2024-02-15" "2024-02-16" ...
##  $ Censor                  : num  1 1 1 1 1 1 1 1 1 1 ...
##  $ Days_to_death_post_BM   : 'difftime' num  8 9 9 9 ...
##   ..- attr(*, "units")= chr "days"
##  $ Age_of_death            : 'difftime' num  11 12 12 12 ...
##   ..- attr(*, "units")= chr "days"
##  $ Age_of_BM_days          : 'difftime' num  3 3 3 3 ...
##   ..- attr(*, "units")= chr "days"
##  $ Date_of_eclosion        : POSIXct, format: "2024-02-04" "2024-02-04" ...
##  $ days_alive_post_BM      : num  8 9 9 9 10 11 11 11 12 12 ...
##  $ days_alive_post_eclosion: num  11 12 12 12 13 14 14 14 15 15 ...
```

```
#start and stop time:
bucket_survival_data$start_time <- (bucket_survival_data$Age_of_BM +1) #start monitoring the day after BM
bucket_survival_data$stop_time <- (bucket_survival_data$days_alive_post_eclosion+1) #age of death + 1 #aod can't be sooner than start
bucket_survival_data$stopminusstart <- bucket_survival_data$stop_time - bucket_survival_data$start_time

#format as numeric or factors:
bucket_survival_data_numeric<-bucket_survival_data
bucket_survival_data$Temperature <- as.factor(bucket_survival_data$Temperature)
bucket_survival_data$Age <- as.factor(bucket_survival_data$Age)
str(bucket_survival_data$Temperature)
```

```
##  Factor w/ 3 levels "27","30","32": 1 1 1 1 1 1 1 1 1 1 ...
```

```
str(bucket_survival_data$Age)
```

```
##  Factor w/ 4 levels "3","5","10","15": 1 1 1 1 1 1 1 1 1 1 ...
```

Plot survival curves

```
#make labels:
bucket_survival_data$Age <- factor(bucket_survival_data$Age,
                                   labels = c("3 days","5 days","10 days","15 days"))
bucket_survival_data$Temperature <- factor(bucket_survival_data$Temperature,
                                           labels = c("27˚C","30˚C","32˚C"))



#generate survival curves
library(ggsurvfit)

s1 <- survfit2(Surv(stopminusstart, Censor) ~ Temperature+Age,
               data = bucket_survival_data)

s2 <- survfit2(Surv(stopminusstart, Censor) ~ Temperature,
               data = bucket_survival_data)

s3 <- survfit2(Surv(stopminusstart, Censor) ~ Age,
               data = bucket_survival_data)

s1
```

```
## Call: survfit(formula = Surv(stopminusstart, Censor) ~ Temperature +
##     Age, data = bucket_survival_data)
##
##                                  n events median 0.95LCL 0.95UCL
## Temperature=27˚C, Age=3 days  140    140   15.0      13      16
## Temperature=27˚C, Age=5 days  105    105   13.0      11      14
## Temperature=27˚C, Age=10 days 105    105    7.0       6       9
## Temperature=27˚C, Age=15 days 105    105    5.0       4       7
## Temperature=30˚C, Age=3 days  172    172   14.5      13      16
## Temperature=30˚C, Age=5 days  140    140   10.0       9      11
## Temperature=30˚C, Age=10 days 105    105    5.0       4       6
## Temperature=30˚C, Age=15 days 130    130    2.0       2       2
## Temperature=32˚C, Age=3 days   80     80   11.0       9      14
## Temperature=32˚C, Age=5 days   91     91    7.0       6       9
## Temperature=32˚C, Age=10 days 105    105    6.0       4       6
## Temperature=32˚C, Age=15 days 120    120    2.0       1       2
```

```
str(s1) #inspect
```

```
## List of 19
##  $ n          : int [1:12] 140 105 105 105 172 140 105 130 80 91 ...
##  $ time       : num [1:252] 1 3 4 5 6 7 8 9 10 11 ...
##  $ n.risk     : num [1:252] 140 138 131 124 119 113 107 106 102 99 ...
##  $ n.event    : num [1:252] 2 7 7 5 6 6 1 4 3 7 ...
##  $ n.censor   : num [1:252] 0 0 0 0 0 0 0 0 0 0 ...
##  $ surv       : num [1:252] 0.986 0.936 0.886 0.85 0.807 ...
##  $ std.err    : num [1:252] 0.0102 0.0222 0.0304 0.0355 0.0413 ...
##  $ cumhaz     : num [1:252] 0.0143 0.065 0.1184 0.1588 0.2092 ...
##  $ std.chaz   : num [1:252] 0.0101 0.0217 0.0296 0.0347 0.0403 ...
##  $ strata     : Named int [1:12] 29 30 22 20 25 25 17 15 22 18 ...
##   ..- attr(*, "names")= chr [1:12] "Temperature=27˚C, Age=3 days " "Temperature=27˚C, Age=5 days " "Temp
erature=27˚C, Age=10 days" "Temperature=27˚C, Age=15 days" ...
##  $ type       : chr "right"
##  $ logse      : logi TRUE
##  $ conf.int   : num 0.95
##  $ conf.type  : chr "log"
##  $ lower      : num [1:252] 0.966 0.896 0.835 0.793 0.744 ...
##  $ upper      : num [1:252] 1 0.977 0.94 0.911 0.875 ...
##  $ t0         : num 0
##  $ call       : language survfit(formula = Surv(stopminusstart, Censor) ~ Temperature + Age, data = buc
ket_survival_data)
##  $ .Environment:<environment: R_GlobalEnv>
##  - attr(*, "class")= chr [1:2] "survfit2" "survfit"
```

```
summary(s1)
```

```
## Call: survfit(formula = Surv(stopminusstart, Censor) ~ Temperature +
##     Age, data = bucket_survival_data)
##
##                 Temperature=27°C, Age=3 days
##  time n.risk n.event survival std.err lower 95% CI upper 95% CI
##     1    140       2   0.9857  0.0100      0.96625       1.0000
##     3    138       7   0.9357  0.0207      0.89596       0.9772
##     4    131       7   0.8857  0.0269      0.83455       0.9400
##     5    124       5   0.8500  0.0302      0.79286       0.9113
##     6    119       6   0.8071  0.0333      0.74436       0.8752
##     7    113       6   0.7643  0.0359      0.69711       0.8379
##     8    107       1   0.7571  0.0362      0.68934       0.8316
##     9    106       4   0.7286  0.0376      0.65851       0.8061
##    10    102       3   0.7071  0.0385      0.63564       0.7867
##    11     99       7   0.6571  0.0401      0.58304       0.7407
##    12     92       6   0.6143  0.0411      0.53872       0.7004
##    13     86       7   0.5643  0.0419      0.48785       0.6527
##    14     79       3   0.5429  0.0421      0.46630       0.6320
##    15     76       9   0.4786  0.0422      0.40258       0.5689
##    16     67      10   0.4071  0.0415      0.33338       0.4972
##    17     57       4   0.3786  0.0410      0.30618       0.4681
##    18     53       7   0.3286  0.0397      0.25929       0.4164
##    19     46       6   0.2857  0.0382      0.21988       0.3713
##    20     40       3   0.2643  0.0373      0.20047       0.3484
##    21     37       8   0.2071  0.0343      0.14980       0.2864
##    22     29       5   0.1714  0.0319      0.11910       0.2467
##    23     24       7   0.1214  0.0276      0.07777       0.1896
##    24     17       3   0.1000  0.0254      0.06084       0.1644
##    25     14       5   0.0643  0.0207      0.03417       0.1209
##    26      9       1   0.0571  0.0196      0.02916       0.1120
##    27      8       1   0.0500  0.0184      0.02429       0.1029
##    28      7       3   0.0286  0.0141      0.01088       0.0751
##    29      4       2   0.0143  0.0100      0.00361       0.0566
##    31      2       2   0.0000     NaN           NA           NA
##
##                 Temperature=27°C, Age=5 days
##  time n.risk n.event survival std.err lower 95% CI upper 95% CI
##     1    105       2  0.98095 0.01334      0.95515       1.0000
##     2    103       3  0.95238 0.02078      0.91251       0.9940
##     3    100       6  0.89524 0.02989      0.83854       0.9558
##     4     94       4  0.85714 0.03415      0.79276       0.9268
##     5     90       3  0.82857 0.03678      0.75953       0.9039
##     6     87       4  0.79048 0.03972      0.71634       0.8723
##     7     83       1  0.78095 0.04036      0.70572       0.8642
##     8     82       8  0.70476 0.04452      0.62270       0.7976
##     9     74       5  0.65714 0.04632      0.57235       0.7545
##    10     69       7  0.59048 0.04799      0.50353       0.6924
##    11     62       3  0.56190 0.04842      0.47459       0.6653
##    12     59       6  0.50476 0.04879      0.41764       0.6101
##    13     53       5  0.45714 0.04862      0.37113       0.5631
##    14     48       9  0.37143 0.04715      0.28961       0.4764
##    15     39       4  0.33333 0.04600      0.25433       0.4369
##    16     35       6  0.27619 0.04363      0.20264       0.3764
##    18     29       3  0.24762 0.04212      0.17741       0.3456
##    19     26       3  0.21905 0.04036      0.15265       0.3143
##    20     23       3  0.19048 0.03832      0.12841       0.2825
```

```
##    21      20        2  0.17143 0.03678      0.11258        0.2610
##    22      18        2  0.15238 0.03507      0.09705        0.2392
##    23      16        3  0.12381 0.03214      0.07443        0.2059
##    24      13        3  0.09524 0.02865      0.05282        0.1717
##    25      10        2  0.07619 0.02589      0.03914        0.1483
##    26       8        1  0.06667 0.02434      0.03259        0.1364
##    27       7        2  0.04762 0.02078      0.02024        0.1120
##    28       5        1  0.03810 0.01868      0.01457        0.0996
##    29       4        1  0.02857 0.01626      0.00937        0.0872
##    30       3        2  0.00952 0.00948      0.00135        0.0670
##    31       1        1  0.00000     NaN           NA            NA
##
##                  Temperature=27˚C, Age=10 days
##  time n.risk n.event survival std.err lower 95% CI upper 95% CI
##     1    105       5   0.9524  0.0208      0.91251        0.9940
##     2    100       8   0.8762  0.0321      0.81540        0.9415
##     3     92       9   0.7905  0.0397      0.71634        0.8723
##     4     83      11   0.6857  0.0453      0.60243        0.7805
##     5     72       8   0.6095  0.0476      0.52300        0.7104
##     6     64       6   0.5524  0.0485      0.46501        0.6562
##     7     58       7   0.4857  0.0488      0.39894        0.5914
##     8     51       5   0.4381  0.0484      0.35277        0.5441
##     9     46       9   0.3524  0.0466      0.27189        0.4567
##    10     37       4   0.3143  0.0453      0.23693        0.4169
##    12     33       4   0.2762  0.0436      0.20264        0.3764
##    13     29       4   0.2381  0.0416      0.16910        0.3352
##    14     25       1   0.2286  0.0410      0.16085        0.3248
##    15     24       2   0.2095  0.0397      0.14451        0.3038
##    16     22       8   0.1333  0.0332      0.08188        0.2171
##    17     14       4   0.0952  0.0286      0.05282        0.1717
##    18     10       3   0.0667  0.0243      0.03259        0.1364
##    19      7       1   0.0571  0.0227      0.02627        0.1243
##    20      6       1   0.0476  0.0208      0.02024        0.1120
##    21      5       2   0.0286  0.0163      0.00937        0.0872
##    23      3       1   0.0190  0.0133      0.00483        0.0752
##    24      2       2   0.0000     NaN           NA            NA
##
##                  Temperature=27˚C, Age=15 days
##  time n.risk n.event survival std.err lower 95% CI upper 95% CI
##     1    105      12   0.8857  0.0310      0.82690        0.9487
##     2     93      14   0.7524  0.0421      0.67419        0.8396
##     3     79      10   0.6571  0.0463      0.57235        0.7545
##     4     69      10   0.5619  0.0484      0.47459        0.6653
##     5     59       7   0.4952  0.0488      0.40827        0.6007
##     6     52       9   0.4095  0.0480      0.32549        0.5153
##     7     43       2   0.3905  0.0476      0.30747        0.4959
##     8     41       4   0.3524  0.0466      0.27189        0.4567
##     9     37       8   0.2762  0.0436      0.20264        0.3764
##    11     29       1   0.2667  0.0432      0.19418        0.3662
##    12     28       6   0.2095  0.0397      0.14451        0.3038
##    13     22       5   0.1619  0.0359      0.10478        0.2502
##    14     17       6   0.1048  0.0299      0.05989        0.1832
##    15     11       1   0.0952  0.0286      0.05282        0.1717
##    16     10       1   0.0857  0.0273      0.04589        0.1601
##    18      9       1   0.0762  0.0259      0.03914        0.1483
##    19      8       1   0.0667  0.0243      0.03259        0.1364
```

```
##    20      7        3   0.0381  0.0187      0.01457        0.0996
##    21      4        2   0.0190  0.0133      0.00483        0.0752
##    23      2        2   0.0000     NaN           NA            NA
## 
##                 Temperature=30˚C, Age=3 days 
##  time n.risk n.event survival std.err lower 95% CI upper 95% CI
##     1    172       3  0.98256 0.00998     0.963188       1.0000
##     2    169       5  0.95349 0.01606     0.922530       0.9855
##     3    164       3  0.93605 0.01866     0.900187       0.9733
##     4    161      10  0.87791 0.02496     0.830318       0.9282
##     5    151       5  0.84884 0.02731     0.796958       0.9041
##     6    146       6  0.81395 0.02967     0.757826       0.8742
##     7    140      10  0.75581 0.03276     0.694263       0.8228
##     8    130      10  0.69767 0.03502     0.632307       0.7698
##     9    120       5  0.66860 0.03589     0.601833       0.7428
##    10    115      10  0.61047 0.03718     0.541771       0.6879
##    11    105       3  0.59302 0.03746     0.523968       0.6712
##    12    102       2  0.58140 0.03762     0.512152       0.6600
##    13    100       9  0.52907 0.03806     0.459493       0.6092
##    14     91       5  0.50000 0.03812     0.430593       0.5806
##    15     86       7  0.45930 0.03800     0.390552       0.5402
##    16     79       7  0.41860 0.03762     0.351006       0.4992
##    17     72       8  0.37209 0.03686     0.306436       0.4518
##    18     64      17  0.27326 0.03398     0.214153       0.3487
##    19     47       9  0.22093 0.03163     0.166869       0.2925
##    20     38      10  0.16279 0.02815     0.115996       0.2285
##    21     28       6  0.12791 0.02547     0.086581       0.1890
##    22     22       7  0.08721 0.02151     0.053776       0.1414
##    23     15       7  0.04651 0.01606     0.023643       0.0915
##    24      8       7  0.00581 0.00580     0.000824       0.0410
##    27      1       1  0.00000     NaN           NA           NA
## 
##                 Temperature=30˚C, Age=5 days 
##  time n.risk n.event survival std.err lower 95% CI upper 95% CI
##     1    140       2  0.98571 0.01003      0.96625       1.0000
##     2    138       6  0.94286 0.01962      0.90518       0.9821
##     3    132      10  0.87143 0.02829      0.81771       0.9287
##     4    122       6  0.82857 0.03185      0.76844       0.8934
##     5    116      15  0.72143 0.03789      0.65086       0.7996
##     6    101       8  0.66429 0.03991      0.59049       0.7473
##     7     93       3  0.64286 0.04050      0.56819       0.7273
##     8     90       6  0.60000 0.04140      0.52410       0.6869
##     9     84       5  0.56429 0.04191      0.48785       0.6527
##    10     79      14  0.46429 0.04215      0.38861       0.5547
##    11     65       8  0.40714 0.04152      0.33338       0.4972
##    12     57       7  0.35714 0.04050      0.28597       0.4460
##    13     50       7  0.30714 0.03899      0.23949       0.3939
##    14     43       6  0.26429 0.03727      0.20047       0.3484
##    15     37       7  0.21429 0.03468      0.15604       0.2943
##    16     30      12  0.12857 0.02829      0.08353       0.1979
##    17     18       2  0.11429 0.02689      0.07206       0.1812
##    18     16       2  0.10000 0.02535      0.06084       0.1644
##    19     14       2  0.08571 0.02366      0.04990       0.1472
##    20     12       1  0.07857 0.02274      0.04456       0.1386
##    21     11       5  0.04286 0.01712      0.01959       0.0938
##    23      6       1  0.03571 0.01568      0.01510       0.0845
```

```
##    24      5         3  0.01429 0.01003      0.00361        0.0566
##    25      2         1  0.00714 0.00712      0.00101        0.0504
##    26      1         1  0.00000     NaN           NA            NA
## 
##                  Temperature=30°C, Age=10 days 
##  time n.risk n.event survival std.err lower 95% CI upper 95% CI
##     1    105       9   0.9143  0.0273      0.86228        0.9694
##     2     96      17   0.7524  0.0421      0.67419        0.8396
##     3     79      14   0.6190  0.0474      0.53279        0.7193
##     4     65       7   0.5524  0.0485      0.46501        0.6562
##     5     58       8   0.4762  0.0487      0.38963        0.5820
##     6     50       9   0.3905  0.0476      0.30747        0.4959
##     7     41       5   0.3429  0.0463      0.26309        0.4468
##     8     36       9   0.2571  0.0427      0.18577        0.3559
##     9     27       4   0.2190  0.0404      0.15265        0.3143
##    10     23       3   0.1905  0.0383      0.12841        0.2825
##    11     20       6   0.1333  0.0332      0.08188        0.2171
##    12     14       2   0.1143  0.0310      0.06710        0.1946
##    14     12       2   0.0952  0.0286      0.05282        0.1717
##    15     10       2   0.0762  0.0259      0.03914        0.1483
##    16      8       3   0.0476  0.0208      0.02024        0.1120
##    18      5       2   0.0286  0.0163      0.00937        0.0872
##    20      3       3   0.0000     NaN           NA            NA
## 
##                  Temperature=30°C, Age=15 days 
##  time n.risk n.event survival std.err lower 95% CI upper 95% CI
##     1    130      49  0.62308 0.04250      0.54510        0.7122
##     2     81      32  0.37692 0.04250      0.30218        0.4702
##     3     49       9  0.30769 0.04048      0.23776        0.3982
##     4     40       6  0.26154 0.03854      0.19592        0.3491
##     5     34       3  0.23846 0.03738      0.17539        0.3242
##     6     31       9  0.16923 0.03289      0.11563        0.2477
##     7     22       7  0.11538 0.02802      0.07169        0.1857
##     8     15       2  0.10000 0.02631      0.05971        0.1675
##     9     13       3  0.07692 0.02337      0.04241        0.1395
##    10     10       2  0.06154 0.02108      0.03145        0.1204
##    11      8       2  0.04615 0.01840      0.02113        0.1008
##    12      6       1  0.03846 0.01687      0.01628        0.0908
##    13      5       1  0.03077 0.01515      0.01173        0.0807
##    15      4       3  0.00769 0.00766      0.00109        0.0542
##    17      1       1  0.00000     NaN           NA            NA
## 
##                  Temperature=32°C, Age=3 days 
##  time n.risk n.event survival std.err lower 95% CI upper 95% CI
##     1     80       1   0.9875  0.0124      0.96345        1.0000
##     2     79       1   0.9750  0.0175      0.94138        1.0000
##     3     78       4   0.9250  0.0294      0.86905        0.9846
##     4     74       5   0.8625  0.0385      0.79024        0.9414
##     5     69       2   0.8375  0.0412      0.76044        0.9224
##     6     67       7   0.7500  0.0484      0.66087        0.8511
##     7     60       9   0.6375  0.0537      0.54040        0.7520
##     8     51       3   0.6000  0.0548      0.50170        0.7176
##     9     48       6   0.5250  0.0558      0.42622        0.6467
##    10     42       1   0.5125  0.0559      0.41388        0.6346
##    11     41       6   0.4375  0.0555      0.34125        0.5609
##    12     35       3   0.4000  0.0548      0.30585        0.5231
```

```
##    13     32        1   0.3875  0.0545       0.29419        0.5104
##    14     31        5   0.3250  0.0524       0.23699        0.4457
##    15     26        8   0.2250  0.0467       0.14982        0.3379
##    16     18        4   0.1750  0.0425       0.10874        0.2816
##    17     14        7   0.0875  0.0316       0.04312        0.1776
##    19      7        1   0.0750  0.0294       0.03474        0.1619
##    20      6        1   0.0625  0.0271       0.02675        0.1460
##    21      5        3   0.0250  0.0175       0.00636        0.0982
##    22      2        1   0.0125  0.0124       0.00178        0.0877
##    23      1        1   0.0000     NaN            NA            NA
## 
##                 Temperature=32˚C, Age=5 days 
##  time n.risk n.event survival std.err lower 95% CI upper 95% CI
##     1     91      10   0.8901  0.0328       0.82812        0.9567
##     2     81       3   0.8571  0.0367       0.78818        0.9321
##     3     78       9   0.7582  0.0449       0.67519        0.8515
##     4     69       7   0.6813  0.0488       0.59200        0.7841
##     5     62       4   0.6374  0.0504       0.54586        0.7442
##     6     58       6   0.5714  0.0519       0.47828        0.6827
##     7     52       7   0.4945  0.0524       0.40175        0.6087
##     8     45       5   0.4396  0.0520       0.34855        0.5543
##     9     40       8   0.3516  0.0501       0.26604        0.4648
##    10     32       3   0.3187  0.0488       0.23599        0.4304
##    11     29       9   0.2198  0.0434       0.14923        0.3237
##    12     20       7   0.1429  0.0367       0.08636        0.2363
##    13     13       4   0.0989  0.0313       0.05319        0.1839
##    14      9       2   0.0769  0.0279       0.03775        0.1567
##    15      7       3   0.0440  0.0215       0.01686        0.1146
##    16      4       2   0.0220  0.0154       0.00558        0.0865
##    19      2       1   0.0110  0.0109       0.00156        0.0772
##    20      1       1   0.0000     NaN            NA            NA
## 
##                 Temperature=32˚C, Age=10 days 
##  time n.risk n.event survival std.err lower 95% CI upper 95% CI
##     1    105      21  0.80000 0.03904       0.72704        0.8803
##     2     84      13  0.67619 0.04567       0.59236        0.7719
##     3     71       6  0.61905 0.04739       0.53279        0.7193
##     4     65       6  0.56190 0.04842       0.47459        0.6653
##     5     59       3  0.53333 0.04869       0.44596        0.6378
##     6     56      16  0.38095 0.04739       0.29852        0.4861
##     7     40       7  0.31429 0.04530       0.23693        0.4169
##     8     33      13  0.19048 0.03832       0.12841        0.2825
##     9     20       5  0.14286 0.03415       0.08942        0.2282
##    10     15       1  0.13333 0.03317       0.08188        0.2171
##    11     14       2  0.11429 0.03105       0.06710        0.1946
##    12     12       2  0.09524 0.02865       0.05282        0.1717
##    13     10       1  0.08571 0.02732       0.04589        0.1601
##    14      9       2  0.06667 0.02434       0.03259        0.1364
##    15      7       5  0.01905 0.01334       0.00483        0.0752
##    18      2       1  0.00952 0.00948       0.00135        0.0670
##    22      1       1  0.00000     NaN            NA            NA
## 
##                 Temperature=32˚C, Age=15 days 
##  time n.risk n.event survival std.err lower 95% CI upper 95% CI
##     1    120      54   0.5500  0.0454       0.46782        0.6466
##     2     66      25   0.3417  0.0433       0.26653        0.4380
```

```
##     3     41      10   0.2583  0.0400      0.19077       0.3498
##     4     31       6   0.2083  0.0371      0.14699       0.2953
##     5     25       5   0.1667  0.0340      0.11171       0.2487
##     6     20       5   0.1250  0.0302      0.07786       0.2007
##     7     15       2   0.1083  0.0284      0.06484       0.1810
##     8     13       4   0.0750  0.0240      0.04001       0.1406
##     9      9       3   0.0500  0.0199      0.02292       0.1091
##    10      6       3   0.0250  0.0143      0.00818       0.0764
##    11      3       1   0.0167  0.0117      0.00422       0.0659
##    12      2       2   0.0000     NaN           NA           NA
```

```
surv_summary(s3, data=bucket_survival_data)
```

```
##     time n.risk n.event n.censor        surv     std.err      upper       lower
## 1      1    392       6        0 0.984693878 0.006297076 0.99692232 0.972615434
## 2      2    386       6        0 0.969387755 0.008975441 0.98659166 0.952483850
## 3      3    380      14        0 0.933673469 0.013461799 0.95863597 0.909360979
## 4      4    366      22        0 0.877551020 0.018866805 0.91060877 0.845693362
## 5      5    344      12        0 0.846938776 0.021471558 0.88334151 0.812036207
## 6      6    332      19        0 0.798469388 0.025374550 0.83918387 0.759730243
## 7      7    313      25        0 0.734693878 0.030351307 0.77972507 0.692263353
## 8      8    288      14        0 0.698979592 0.033145356 0.74589531 0.655014807
## 9      9    274      15        0 0.660714286 0.036193694 0.70928671 0.615468130
## 10    10    259      14        0 0.625000000 0.039123040 0.67481015 0.578866511
## 11    11    245      16        0 0.584183673 0.042612109 0.63506899 0.537375575
## 12    12    229      11        0 0.556122449 0.045123559 0.60754670 0.509050870
## 13    13    218      17        0 0.512755102 0.049235190 0.56470165 0.465587080
## 14    14    201      13        0 0.479591837 0.052613007 0.53168702 0.432600990
## 15    15    188      24        0 0.418367347 0.059552838 0.47016387 0.372277092
## 16    16    164      21        0 0.364795918 0.066648230 0.41570098 0.320124486
## 17    17    143      19        0 0.316326531 0.074252917 0.36588097 0.273483683
## 18    18    124      24        0 0.255102041 0.086307471 0.30211966 0.215401578
## 19    19    100      16        0 0.214285714 0.096714743 0.25901025 0.177283975
## 20    20     84      14        0 0.178571429 0.108326792 0.22081059 0.144412252
## 21    21     70      17        0 0.135204082 0.127737638 0.17366819 0.105259021
## 22    22     53      13        0 0.102040816 0.149829835 0.13687033 0.076074400
## 23    23     40      15        0 0.063775510 0.193517388 0.09319149 0.043644713
## 24    24     25      10        0 0.038265306 0.253210676 0.06285481 0.023295491
## 25    25     15       5        0 0.025510204 0.312168191 0.04703617 0.013835533
## 26    26     10       1        0 0.022959184 0.329484583 0.04379396 0.012036457
## 27    27      9       2        0 0.017857143 0.374574589 0.03720921 0.008569856
## 28    28      7       3        0 0.010204082 0.497442438 0.02705190 0.003849020
## 29    29      4       2        0 0.005102041 0.705300631 0.02032811 0.001280533
## 30    31      2       2        0 0.000000000         Inf         NA          NA
## 31     1    336      14        0 0.958333333 0.011375394 0.97993969 0.937203370
## 32     2    322      12        0 0.922619048 0.015799240 0.95163576 0.894487095
## 33     3    310      25        0 0.848214286 0.023077726 0.88746121 0.810703012
## 34     4    285      17        0 0.797619048 0.027480044 0.84175668 0.755795777
## 35     5    268      22        0 0.732142857 0.032997730 0.78105847 0.686290696
## 36     6    246      18        0 0.678571429 0.037546963 0.73039132 0.630428062
## 37     7    228      11        0 0.645833333 0.040399313 0.69905028 0.596667660
## 38     8    217      19        0 0.589285714 0.045544644 0.64430809 0.538962119
## 39     9    198      18        0 0.535714286 0.050787450 0.59178450 0.484956597
## 40    10    180      24        0 0.464285714 0.058600904 0.52079463 0.413908306
## 41    11    156      20        0 0.404761905 0.066157016 0.46080012 0.355538534
## 42    12    136      20        0 0.345238095 0.075129882 0.40000865 0.297966912
## 43    13    116      16        0 0.297619048 0.083808171 0.35075056 0.252535873
## 44    14    100      17        0 0.247023810 0.095247059 0.29772360 0.204957762
## 45    15     83      14        0 0.205357143 0.107315251 0.25342924 0.166403672
## 46    16     69      20        0 0.145833333 0.132030197 0.18890398 0.112582917
## 47    17     49       2        0 0.139880952 0.135278990 0.18235105 0.107302266
## 48    18     47       5        0 0.125000000 0.144337567 0.16587097 0.094199729
## 49    19     42       6        0 0.107142857 0.157485197 0.14588642 0.078688557
## 50    20     36       5        0 0.092261905 0.171119473 0.12902670 0.065972852
## 51    21     31       7        0 0.071428571 0.196698948 0.10502735 0.048578211
## 52    22     24       2        0 0.065476190 0.206102778 0.09806599 0.043716804
## 53    23     22       4        0 0.053571429 0.229301908 0.08396830 0.034178350
## 54    24     18       6        0 0.035714286 0.283473355 0.06224937 0.020490333
## 55    25     12       3        0 0.026785714 0.328838746 0.05102832 0.014060319
```

```
## 56    26       9        2         0 0.020833333 0.374006621 0.04336245 0.010009301
## 57    27       7        2         0 0.014880952 0.443873641 0.03551867 0.006234545
## 58    28       5        1         0 0.011904762 0.497014899 0.03153411 0.004494288
## 59    29       4        1         0 0.008928571 0.574767034 0.02754384 0.002894272
## 60    30       3        2         0 0.002976190 0.998510796 0.02106661 0.000420462
## 61    31       1        1         0 0.000000000         Inf         NA          NA
## 62     1     315       35         0 0.888888889 0.019920477 0.92428056 0.854852403
## 63     2     280       38         0 0.768253968 0.030945569 0.81629235 0.723042618
## 64     3     242       29         0 0.676190476 0.038990159 0.72989020 0.626441565
## 65     4     213       24         0 0.600000000 0.046004371 0.65661416 0.548267192
## 66     5     189       19         0 0.539682540 0.052036043 0.59762881 0.487354755
## 67     6     170       31         0 0.441269841 0.063400642 0.49965584 0.389706386
## 68     7     139       19         0 0.380952381 0.071824301 0.43853842 0.330928169
## 69     8     120       27         0 0.295238095 0.087052197 0.35016389 0.248927817
## 70     9      93       18         0 0.238095238 0.100790526 0.29009735 0.195414893
## 71    10      75        8         0 0.212698413 0.108400968 0.26304819 0.171986032
## 72    11      67        8         0 0.187301587 0.117365026 0.23574514 0.148812758
## 73    12      59        8         0 0.161904762 0.128192199 0.20815029 0.125933776
## 74    13      51        5         0 0.146031746 0.136251706 0.19073260 0.111807160
## 75    14      46        5         0 0.130158730 0.145655898 0.17316329 0.097834218
## 76    15      41        9         0 0.101587302 0.167557145 0.14107963 0.073150033
## 77    16      32       11         0 0.066666667 0.210818511 0.10077615 0.044102144
## 78    17      21        4         0 0.053968254 0.235900246 0.08569136 0.033989104
## 79    18      17        6         0 0.034920635 0.296200081 0.06240338 0.019541422
## 80    19      11        1         0 0.031746032 0.311167795 0.05841925 0.017251344
## 81    20      10        4         0 0.019047619 0.404341518 0.04207429 0.008623123
## 82    21       6        2         0 0.012698413 0.496815254 0.03362322 0.004795783
## 83    22       4        1         0 0.009523810 0.574594405 0.02937016 0.003088269
## 84    23       3        1         0 0.006349206 0.704858423 0.02527528 0.001594935
## 85    24       2        2         0 0.000000000         Inf         NA          NA
## 86     1     355      115         0 0.676056338 0.036739152 0.72653294 0.629086646
## 87     2     240       71         0 0.476056338 0.055679964 0.53094951 0.426838397
## 88     3     169       29         0 0.394366197 0.065771998 0.44862649 0.346668560
## 89     4     140       22         0 0.332394366 0.075217517 0.38519347 0.286832519
## 90     5     118       15         0 0.290140845 0.083017085 0.34140757 0.246572478
## 91     6     103       23         0 0.225352113 0.098402737 0.27328903 0.185823687
## 92     7      80       11         0 0.194366197 0.108054857 0.24021338 0.157269417
## 93     8      69       10         0 0.166197183 0.118879145 0.20980400 0.131653845
## 94     9      59       14         0 0.126760563 0.139302982 0.16655554 0.096473769
## 95    10      45        5         0 0.112676056 0.148939916 0.15087230 0.084149929
## 96    11      40        4         0 0.101408451 0.157990115 0.13821513 0.074403389
## 97    12      36        9         0 0.076056338 0.184986853 0.10929405 0.052926639
## 98    13      27        6         0 0.059154930 0.211665175 0.08956961 0.039068004
## 99    14      21        6         0 0.042253521 0.252685111 0.06933442 0.025749983
## 100   15      15        4         0 0.030985915 0.296803284 0.05543751 0.017319084
## 101   16      11        1         0 0.028169014 0.311742039 0.05189517 0.015290312
## 102   17      10        1         0 0.025352113 0.329080856 0.04832015 0.013301482
## 103   18       9        1         0 0.022535211 0.349546991 0.04470917 0.011358649
## 104   19       8        1         0 0.019718310 0.374219510 0.04105877 0.009469640
## 105   20       7        3         0 0.011267606 0.497175119 0.02985574 0.004252413
## 106   21       4        2         0 0.005633803 0.705112118 0.02243852 0.001414520
## 107   23       2        2         0 0.000000000         Inf         NA          NA
##           strata     Age
## 1    Age=3 days  3 days
## 2    Age=3 days  3 days
## 3    Age=3 days  3 days
```

```
## 4    Age=3 days  3 days
## 5    Age=3 days  3 days
## 6    Age=3 days  3 days
## 7    Age=3 days  3 days
## 8    Age=3 days  3 days
## 9    Age=3 days  3 days
## 10   Age=3 days  3 days
## 11   Age=3 days  3 days
## 12   Age=3 days  3 days
## 13   Age=3 days  3 days
## 14   Age=3 days  3 days
## 15   Age=3 days  3 days
## 16   Age=3 days  3 days
## 17   Age=3 days  3 days
## 18   Age=3 days  3 days
## 19   Age=3 days  3 days
## 20   Age=3 days  3 days
## 21   Age=3 days  3 days
## 22   Age=3 days  3 days
## 23   Age=3 days  3 days
## 24   Age=3 days  3 days
## 25   Age=3 days  3 days
## 26   Age=3 days  3 days
## 27   Age=3 days  3 days
## 28   Age=3 days  3 days
## 29   Age=3 days  3 days
## 30   Age=3 days  3 days
## 31   Age=5 days  5 days
## 32   Age=5 days  5 days
## 33   Age=5 days  5 days
## 34   Age=5 days  5 days
## 35   Age=5 days  5 days
## 36   Age=5 days  5 days
## 37   Age=5 days  5 days
## 38   Age=5 days  5 days
## 39   Age=5 days  5 days
## 40   Age=5 days  5 days
## 41   Age=5 days  5 days
## 42   Age=5 days  5 days
## 43   Age=5 days  5 days
## 44   Age=5 days  5 days
## 45   Age=5 days  5 days
## 46   Age=5 days  5 days
## 47   Age=5 days  5 days
## 48   Age=5 days  5 days
## 49   Age=5 days  5 days
## 50   Age=5 days  5 days
## 51   Age=5 days  5 days
## 52   Age=5 days  5 days
## 53   Age=5 days  5 days
## 54   Age=5 days  5 days
## 55   Age=5 days  5 days
## 56   Age=5 days  5 days
## 57   Age=5 days  5 days
## 58   Age=5 days  5 days
## 59   Age=5 days  5 days
```

```
## 60   Age=5 days  5 days
## 61   Age=5 days  5 days
## 62  Age=10 days 10 days
## 63  Age=10 days 10 days
## 64  Age=10 days 10 days
## 65  Age=10 days 10 days
## 66  Age=10 days 10 days
## 67  Age=10 days 10 days
## 68  Age=10 days 10 days
## 69  Age=10 days 10 days
## 70  Age=10 days 10 days
## 71  Age=10 days 10 days
## 72  Age=10 days 10 days
## 73  Age=10 days 10 days
## 74  Age=10 days 10 days
## 75  Age=10 days 10 days
## 76  Age=10 days 10 days
## 77  Age=10 days 10 days
## 78  Age=10 days 10 days
## 79  Age=10 days 10 days
## 80  Age=10 days 10 days
## 81  Age=10 days 10 days
## 82  Age=10 days 10 days
## 83  Age=10 days 10 days
## 84  Age=10 days 10 days
## 85  Age=10 days 10 days
## 86  Age=15 days 15 days
## 87  Age=15 days 15 days
## 88  Age=15 days 15 days
## 89  Age=15 days 15 days
## 90  Age=15 days 15 days
## 91  Age=15 days 15 days
## 92  Age=15 days 15 days
## 93  Age=15 days 15 days
## 94  Age=15 days 15 days
## 95  Age=15 days 15 days
## 96  Age=15 days 15 days
## 97  Age=15 days 15 days
## 98  Age=15 days 15 days
## 99  Age=15 days 15 days
## 100 Age=15 days 15 days
## 101 Age=15 days 15 days
## 102 Age=15 days 15 days
## 103 Age=15 days 15 days
## 104 Age=15 days 15 days
## 105 Age=15 days 15 days
## 106 Age=15 days 15 days
## 107 Age=15 days 15 days
```

```
summary(s3)
```

```
## Call: survfit(formula = Surv(stopminusstart, Censor) ~ Age, data = bucket_survival_data)
##
##                 Age=3 days
##  time n.risk n.event survival std.err lower 95% CI upper 95% CI
##     1    392       6   0.9847 0.00620      0.97262       0.9969
##     2    386       6   0.9694 0.00870      0.95248       0.9866
##     3    380      14   0.9337 0.01257      0.90936       0.9586
##     4    366      22   0.8776 0.01656      0.84569       0.9106
##     5    344      12   0.8469 0.01819      0.81204       0.8833
##     6    332      19   0.7985 0.02026      0.75973       0.8392
##     7    313      25   0.7347 0.02230      0.69226       0.7797
##     8    288      14   0.6990 0.02317      0.65501       0.7459
##     9    274      15   0.6607 0.02391      0.61547       0.7093
##    10    259      14   0.6250 0.02445      0.57887       0.6748
##    11    245      16   0.5842 0.02489      0.53738       0.6351
##    12    229      11   0.5561 0.02509      0.50905       0.6075
##    13    218      17   0.5128 0.02525      0.46559       0.5647
##    14    201      13   0.4796 0.02523      0.43260       0.5317
##    15    188      24   0.4184 0.02491      0.37228       0.4702
##    16    164      21   0.3648 0.02431      0.32012       0.4157
##    17    143      19   0.3163 0.02349      0.27348       0.3659
##    18    124      24   0.2551 0.02202      0.21540       0.3021
##    19    100      16   0.2143 0.02072      0.17728       0.2590
##    20     84      14   0.1786 0.01934      0.14441       0.2208
##    21     70      17   0.1352 0.01727      0.10526       0.1737
##    22     53      13   0.1020 0.01529      0.07607       0.1369
##    23     40      15   0.0638 0.01234      0.04364       0.0932
##    24     25      10   0.0383 0.00969      0.02330       0.0629
##    25     15       5   0.0255 0.00796      0.01384       0.0470
##    26     10       1   0.0230 0.00756      0.01204       0.0438
##    27      9       2   0.0179 0.00669      0.00857       0.0372
##    28      7       3   0.0102 0.00508      0.00385       0.0271
##    29      4       2   0.0051 0.00360      0.00128       0.0203
##    31      2       2   0.0000     NaN           NA           NA
##
##                 Age=5 days
##  time n.risk n.event survival std.err lower 95% CI upper 95% CI
##     1    336      14  0.95833 0.01090      0.93720       0.9799
##     2    322      12  0.92262 0.01458      0.89449       0.9516
##     3    310      25  0.84821 0.01957      0.81070       0.8875
##     4    285      17  0.79762 0.02192      0.75580       0.8418
##     5    268      22  0.73214 0.02416      0.68629       0.7811
##     6    246      18  0.67857 0.02548      0.63043       0.7304
##     7    228      11  0.64583 0.02609      0.59667       0.6991
##     8    217      19  0.58929 0.02684      0.53896       0.6443
##     9    198      18  0.53571 0.02721      0.48496       0.5918
##    10    180      24  0.46429 0.02721      0.41391       0.5208
##    11    156      20  0.40476 0.02678      0.35554       0.4608
##    12    136      20  0.34524 0.02594      0.29797       0.4000
##    13    116      16  0.29762 0.02494      0.25254       0.3508
##    14    100      17  0.24702 0.02353      0.20496       0.2977
##    15     83      14  0.20536 0.02204      0.16640       0.2534
##    16     69      20  0.14583 0.01925      0.11258       0.1889
##    17     49       2  0.13988 0.01892      0.10730       0.1824
##    18     47       5  0.12500 0.01804      0.09420       0.1659
##    19     42       6  0.10714 0.01687      0.07869       0.1459
```

```
##    20     36        5  0.09226 0.01579      0.06597       0.1290
##    21     31        7  0.07143 0.01405      0.04858       0.1050
##    22     24        2  0.06548 0.01349      0.04372       0.0981
##    23     22        4  0.05357 0.01228      0.03418       0.0840
##    24     18        6  0.03571 0.01012      0.02049       0.0622
##    25     12        3  0.02679 0.00881      0.01406       0.0510
##    26      9        2  0.02083 0.00779      0.01001       0.0434
##    27      7        2  0.01488 0.00661      0.00623       0.0355
##    28      5        1  0.01190 0.00592      0.00449       0.0315
##    29      4        1  0.00893 0.00513      0.00289       0.0275
##    30      3        2  0.00298 0.00297      0.00042       0.0211
##    31      1        1  0.00000     NaN           NA           NA
## 
##                 Age=10 days 
##  time n.risk n.event survival std.err lower 95% CI upper 95% CI
##     1    315       35  0.88889 0.01771      0.85485       0.9243
##     2    280       38  0.76825 0.02377      0.72304       0.8163
##     3    242       29  0.67619 0.02636      0.62644       0.7299
##     4    213       24  0.60000 0.02760      0.54827       0.6566
##     5    189       19  0.53968 0.02808      0.48735       0.5976
##     6    170       31  0.44127 0.02798      0.38971       0.4997
##     7    139       19  0.38095 0.02736      0.33093       0.4385
##     8    120       27  0.29524 0.02570      0.24893       0.3502
##     9     93       18  0.23810 0.02400      0.19541       0.2901
##    10     75        8  0.21270 0.02306      0.17199       0.2630
##    11     67        8  0.18730 0.02198      0.14881       0.2357
##    12     59        8  0.16190 0.02075      0.12593       0.2082
##    13     51        5  0.14603 0.01990      0.11181       0.1907
##    14     46        5  0.13016 0.01896      0.09783       0.1732
##    15     41        9  0.10159 0.01702      0.07315       0.1411
##    16     32       11  0.06667 0.01405      0.04410       0.1008
##    17     21        4  0.05397 0.01273      0.03399       0.0857
##    18     17        6  0.03492 0.01034      0.01954       0.0624
##    19     11        1  0.03175 0.00988      0.01725       0.0584
##    20     10        4  0.01905 0.00770      0.00862       0.0421
##    21      6        2  0.01270 0.00631      0.00480       0.0336
##    22      4        1  0.00952 0.00547      0.00309       0.0294
##    23      3        1  0.00635 0.00448      0.00159       0.0253
##    24      2        2  0.00000     NaN           NA           NA
## 
##                 Age=15 days 
##  time n.risk n.event survival std.err lower 95% CI upper 95% CI
##     1    355      115  0.67606 0.02484      0.62909       0.7265
##     2    240       71  0.47606 0.02651      0.42684       0.5309
##     3    169       29  0.39437 0.02594      0.34667       0.4486
##     4    140       22  0.33239 0.02500      0.28683       0.3852
##     5    118       15  0.29014 0.02409      0.24657       0.3414
##     6    103       23  0.22535 0.02218      0.18582       0.2733
##     7     80       11  0.19437 0.02100      0.15727       0.2402
##     8     69       10  0.16620 0.01976      0.13165       0.2098
##     9     59       14  0.12676 0.01766      0.09647       0.1666
##    10     45        5  0.11268 0.01678      0.08415       0.1509
##    11     40        4  0.10141 0.01602      0.07440       0.1382
##    12     36        9  0.07606 0.01407      0.05293       0.1093
##    13     27        6  0.05915 0.01252      0.03907       0.0896
##    14     21        6  0.04225 0.01068      0.02575       0.0693
```

```
##    15     15        4  0.03099 0.00920       0.01732        0.0554
##    16     11        1  0.02817 0.00878       0.01529        0.0519
##    17     10        1  0.02535 0.00834       0.01330        0.0483
##    18      9        1  0.02254 0.00788       0.01136        0.0447
##    19      8        1  0.01972 0.00738       0.00947        0.0411
##    20      7        3  0.01127 0.00560       0.00425        0.0299
##    21      4        2  0.00563 0.00397       0.00141        0.0224
##    23      2        2  0.00000     NaN            NA            NA
```

```
# Plot survival curves
ggsurv <- ggsurvplot(s1, conf.int = TRUE, color="Age",palette = c("#DCD1E9","#BAA4D3","#9776BE","#7549A8"),
                     ggtheme = theme_pubr(),surv.median.line = "v",confint=TRUE)

s1table <- as.data.frame(ggsurv$data.survplot)

firstBM_kaplanmeiersurvivalcurves_stopminusstart <- ggsurv$plot +
  theme_pubr() +
  theme(legend.position = "none")+
  facet_grid(Temperature~Age)+
  ylab(expression("Survival probability"))+
  xlab("Time (Days post blood feeding)") +
  theme(panel.background = element_rect(fill = NA, color = "black"))+
  theme(panel.spacing = unit(0.6, "lines"))
firstBM_kaplanmeiersurvivalcurves_stopminusstart
```

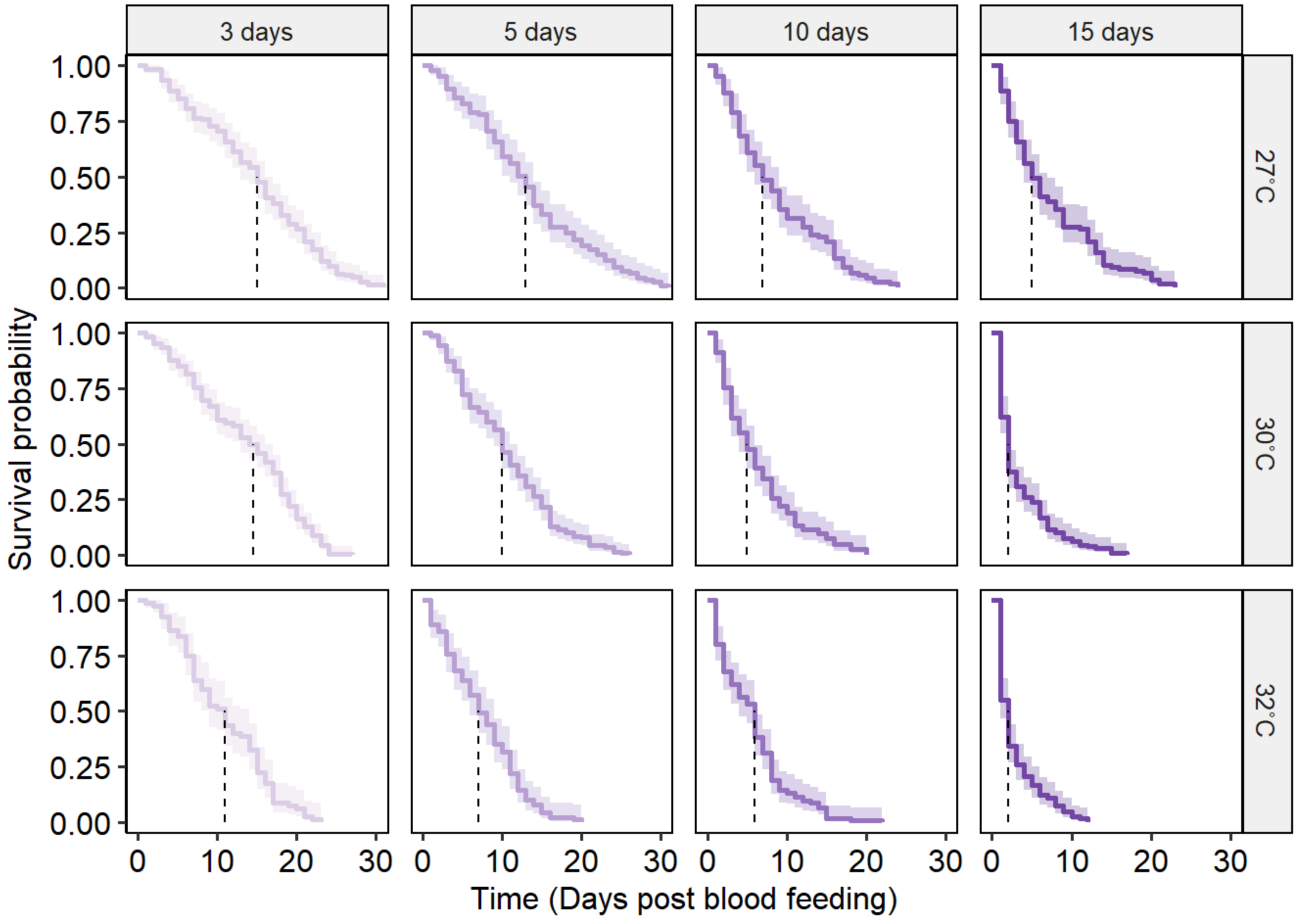

```r
ggsave("Bucket_survival/firstBM_kaplanmeiersurvivalcurves_stopminusstart.png", plot=firstBM_kaplanmeiersurv
ivalcurves_stopminusstart, width = 6, height = 4, units = "in",dpi=600)
ggsave("Bucket_survival/firstBM_kaplanmeiersurvivalcurves_stopminusstart.pdf", plot=firstBM_kaplanmeiersurv
ivalcurves_stopminusstart, width = 6, height = 4, units = "in",dpi=600)

###
#save median survival time:
s1
```

```
## Call: survfit(formula = Surv(stopminusstart, Censor) ~ Temperature +
##     Age, data = bucket_survival_data)
##
##                                  n events median 0.95LCL 0.95UCL
## Temperature=27˚C, Age=3 days  140    140   15.0      13      16
## Temperature=27˚C, Age=5 days  105    105   13.0      11      14
## Temperature=27˚C, Age=10 days 105    105    7.0       6       9
## Temperature=27˚C, Age=15 days 105    105    5.0       4       7
## Temperature=30˚C, Age=3 days  172    172   14.5      13      16
## Temperature=30˚C, Age=5 days  140    140   10.0       9      11
## Temperature=30˚C, Age=10 days 105    105    5.0       4       6
## Temperature=30˚C, Age=15 days 130    130    2.0       2       2
## Temperature=32˚C, Age=3 days   80     80   11.0       9      14
## Temperature=32˚C, Age=5 days   91     91    7.0       6       9
## Temperature=32˚C, Age=10 days 105    105    6.0       4       6
## Temperature=32˚C, Age=15 days 120    120    2.0       1       2
```

```r
sink("Bucket_survival/firstBM_bucketsurvival_mediansurvivaltime.txt")
print(s1,print.rmean = TRUE)
sink()

s1data <- print(s1,print.rmean = TRUE)
```

```
## Call: survfit(formula = Surv(stopminusstart, Censor) ~ Temperature +
##     Age, data = bucket_survival_data)
##
##                                   n events rmean* se(rmean) median 0.95LCL
## Temperature=27˚C, Age=3 days  140    140  14.76     0.628   15.0      13
## Temperature=27˚C, Age=5 days  105    105  13.29     0.734   13.0      11
## Temperature=27˚C, Age=10 days 105    105   8.80     0.585    7.0       6
## Temperature=27˚C, Age=15 days 105    105   7.29     0.568    5.0       4
## Temperature=30˚C, Age=3 days  172    172  13.53     0.509   14.5      13
## Temperature=30˚C, Age=5 days  140    140  10.49     0.501   10.0       9
## Temperature=30˚C, Age=10 days 105    105   6.40     0.472    5.0       4
## Temperature=30˚C, Age=15 days 130    130   3.49     0.308    2.0       2
## Temperature=32˚C, Age=3 days   80     80  10.91     0.608   11.0       9
## Temperature=32˚C, Age=5 days   91     91   7.66     0.481    7.0       6
## Temperature=32˚C, Age=10 days 105    105   5.81     0.430    6.0       4
## Temperature=32˚C, Age=15 days 120    120   2.93     0.253    2.0       1
##                               0.95UCL
## Temperature=27˚C, Age=3 days       16
## Temperature=27˚C, Age=5 days       14
## Temperature=27˚C, Age=10 days       9
## Temperature=27˚C, Age=15 days       7
## Temperature=30˚C, Age=3 days       16
## Temperature=30˚C, Age=5 days       11
## Temperature=30˚C, Age=10 days       6
## Temperature=30˚C, Age=15 days       2
## Temperature=32˚C, Age=3 days       14
## Temperature=32˚C, Age=5 days        9
## Temperature=32˚C, Age=10 days       6
## Temperature=32˚C, Age=15 days       2
##     * restricted mean with upper limit =  31
```

```
#s2
ggsurv <- ggsurvplot(s2, conf.int = TRUE,
                     ggtheme = theme_pubr(),surv.median.line = "v",confint=TRUE,
                     palette = c("#4D6FAE","#6F9F51", "#CC763B"),risk.table = TRUE,
)

ggsurv
```

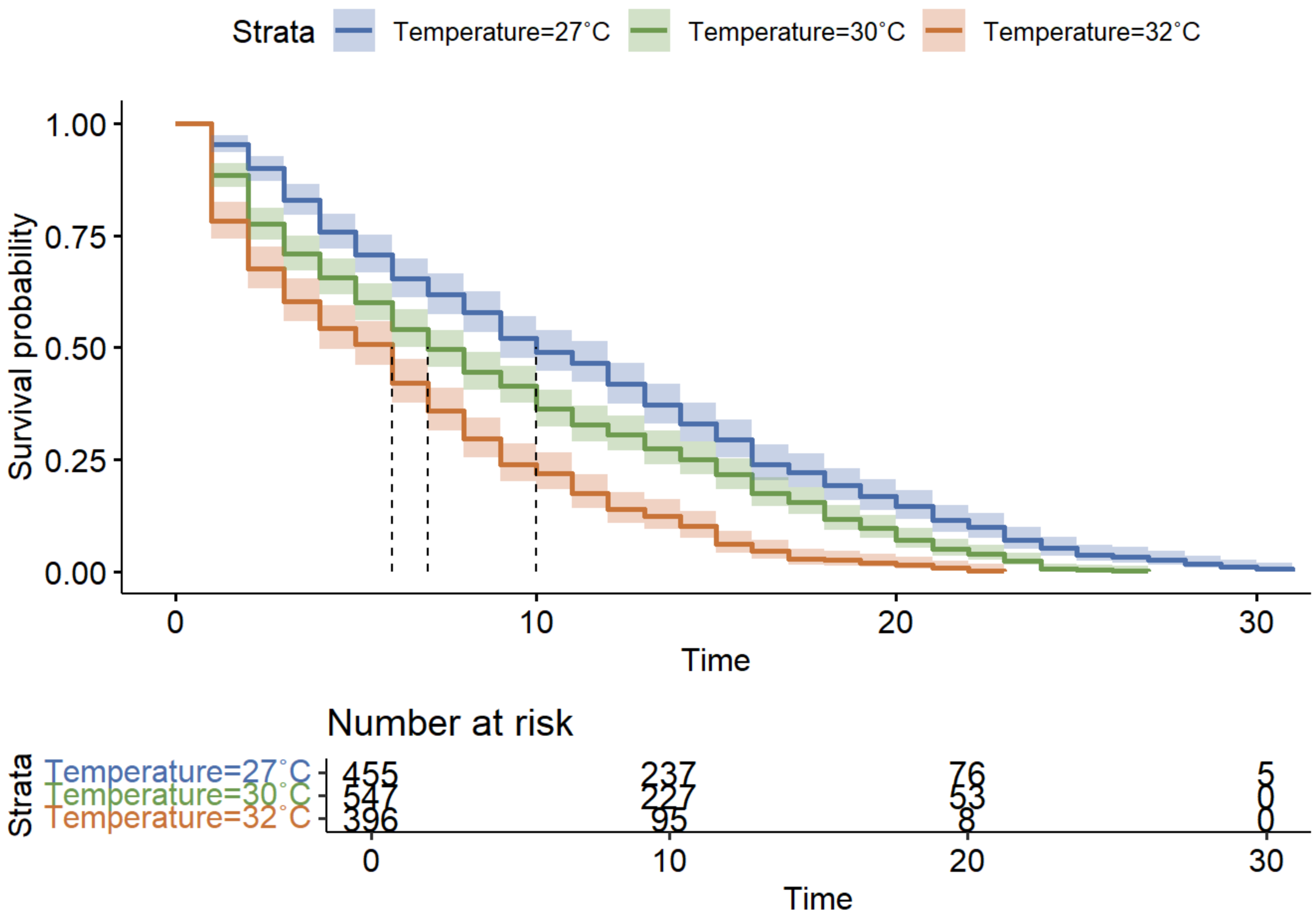


```
firstBM_TEMPonegraph_kaplanmeiersurvivalcurves_stopminusstart <- ggsurv$plot +
  theme_pubr() +
  theme(legend.position = "right")+
  ylab(expression("Survival probability"))+
  #scale_y_continuous(labels = function(x) paste0(x*100),limits=c(0,1)) +
  xlab("Time (Days post blood feeding)") +
  theme(legend.position = "none")+
  #scale_fill_discrete(name="Temperature")
  #scale_color_manual(values= c("#4D6FAE","#6F9F51", "#CC763B"))+
  theme(panel.background = element_rect(fill = NA, color = "black"))+
  theme(panel.spacing = unit(0.6, "lines"))
firstBM_TEMPonegraph_kaplanmeiersurvivalcurves_stopminusstart
```

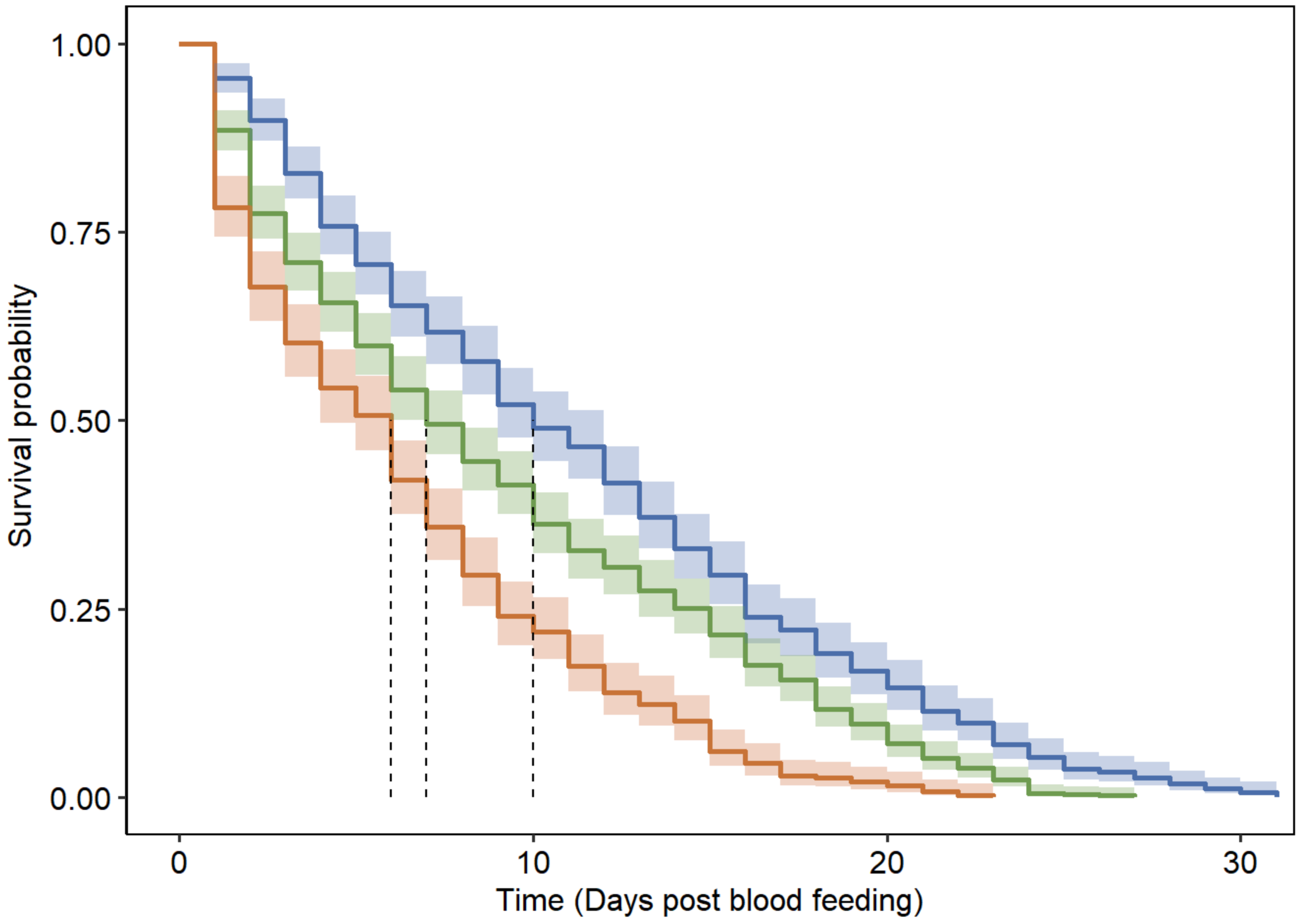


```
ggsave("Bucket_survival/firstBM_TEMPonegraph_kaplanmeiersurvivalcurves_stopminusstart.pdf", plot=firstBM_TE
MPonegraph_kaplanmeiersurvivalcurves_stopminusstart, width = 5, height = 4, units = "in",dpi=600)
ggsave("Bucket_survival/firstBM_TEMPonegraph_kaplanmeiersurvivalcurves_stopminusstart.png", plot=firstBM_TE
MPonegraph_kaplanmeiersurvivalcurves_stopminusstart, width = 5, height = 4, units = "in",dpi=600)

###
#save median survival time:
s2
```

```
## Call: survfit(formula = Surv(stopminusstart, Censor) ~ Temperature,
##     data = bucket_survival_data)
##
##                      n events median 0.95LCL 0.95UCL
## Temperature=27˚C 455    455     10       9      12
## Temperature=30˚C 547    547      7       7       8
## Temperature=32˚C 396    396      6       4       6
```

```
sink("Bucket_survival/firstBM_TEMP_bucketsurvival_mediansurvivaltime_stopminusstart.txt")
print(s2,print.rmean = TRUE)
sink()

tables2<- s2 %>%
  tbl_survfit(
    probs = 0.5,
    label_header = "**Median survival (95% CI)**"
  )
tables2
```

Characteristic	Median survival (95% CI)
Temperature	
27˚C	10 (9.0, 12)
30˚C	7.0 (7.0, 8.0)
32˚C	6.0 (4.0, 6.0)

```
#s3
ggsurv <- ggsurvplot(s3, conf.int = TRUE,
                     ggtheme = theme_pubr(),surv.median.line = "v",confint=TRUE,
                     palette = c("#DCD1E9","#BAA4D3","#9776BE","#7549A8"),
                     axes.offset = TRUE,
                     risk.table = TRUE)



firstBM_AGEonegraph_kaplanmeiersurvivalcurves_stopminusstart <- ggsurv$plot +
  theme_pubr() +
  theme(legend.position = "right")+
  ylab(expression("Survival probability"))+
  xlab("Time (Days post blood feeding)") +
  theme(legend.position = "none")+
  #scale_color_manual(values= c("#DCD1E9","#BAA4D3","#9776BE","#7549A8"))+
  #geom_smooth(fill= c("#DCD1E9","#BAA4D3","#9776BE","#7549A8"))+
  theme(panel.background = element_rect(fill = NA, color = "black"))+
  theme(panel.spacing = unit(0.6, "lines"))
firstBM_AGEonegraph_kaplanmeiersurvivalcurves_stopminusstart
```

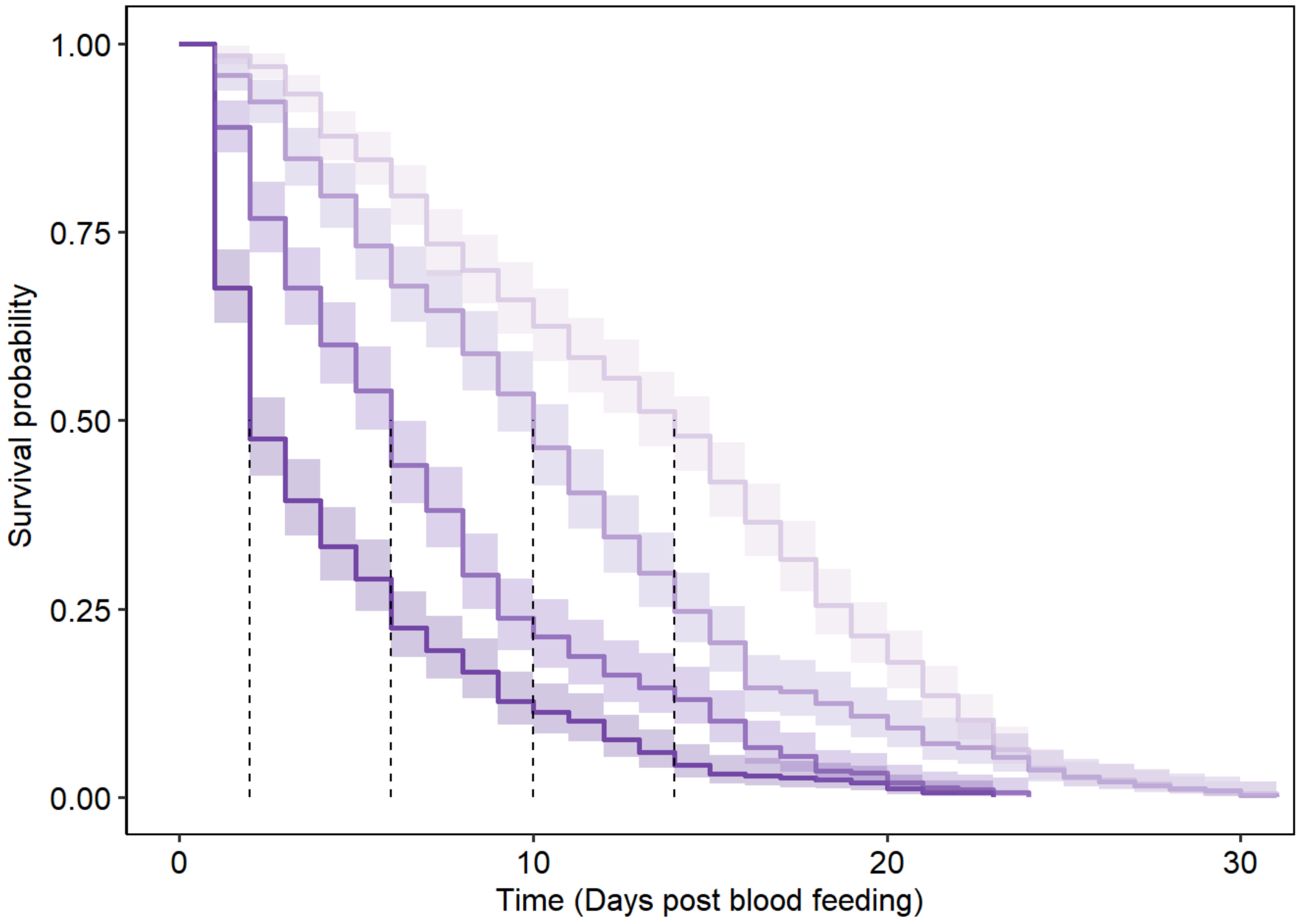


```
ggsave("Bucket_survival/firstBM_AGEonegraph_kaplanmeiersurvivalcurves_stopminusstart.pdf", plot=firstBM_AGE
onegraph_kaplanmeiersurvivalcurves_stopminusstart, width = 5, height = 4, units = "in",dpi=600)
ggsave("Bucket_survival/firstBM_AGEonegraph_kaplanmeiersurvivalcurves_stopminusstart.png", plot=firstBM_AGE
onegraph_kaplanmeiersurvivalcurves_stopminusstart, width = 5, height = 4, units = "in",dpi=600)

###
#save median survival time:
s3
```

```
## Call: survfit(formula = Surv(stopminusstart, Censor) ~ Age, data = bucket_survival_data)
##
##                  n events median 0.95LCL 0.95UCL
## Age=3 days  392    392     14      13      15
## Age=5 days  336    336     10       9      11
## Age=10 days 315    315      6       5       6
## Age=15 days 355    355      2       2       3
```

```
sink("Bucket_survival/firstBM_AGE_bucketsurvival_mediansurvivaltime_stopminusstart.txt")
print(s3,print.rmean = TRUE)
sink()

s3 %>%
  tbl_survfit(
    probs = 0.5,
    label_header = "**Median survival (95% CI)**"
  )
```

Characteristic	Median survival (95% CI)
Age	
3 days	14 (13, 15)
5 days	10 (9.0, 11)
10 days	6.0 (5.0, 6.0)
15 days	2.0 (2.0, 3.0)

Analysis for survival data Fig 3

```
#ANALYSIS - HAZARD RATIOS
#switch back to numbers:
bucket_survival_data_numeric<-bucket_survival_data
str(bucket_survival_data_numeric)
```

```
## 'data.frame':    1398 obs. of  22 variables:
##  $ ID_overall              : num  1 2 3 4 5 6 7 8 9 10 ...
##  $ Temperature             : Factor w/ 3 levels "27˚C","30˚C",..: 1 1 1 1 1 1 1 1 1 1 ...
##  $ Age                     : Factor w/ 4 levels "3 days","5 days",..: 1 1 1 1 1 1 1 1 1 1 ...
##  $ Age_of_BM               : num  3 3 3 3 3 3 3 3 3 3 ...
##  $ ID_per_group            : num  1 2 3 4 5 6 7 8 9 10 ...
##  $ Trial_start_date        : POSIXct, format: "2024-02-07" "2024-02-07" ...
##  $ Date_Group              : chr  "A" "A" "A" "A" ...
##  $ Trial_number            : num  1 1 1 1 1 1 1 1 1 1 ...
##  $ Initial_n               : num  35 35 35 35 35 35 35 35 35 35 ...
##  $ BM1_Date                : POSIXct, format: "2024-02-07" "2024-02-07" ...
##  $ Bloodmeal_number        : num  1 1 1 1 1 1 1 1 1 1 ...
##  $ Date_of_death           : POSIXct, format: "2024-02-15" "2024-02-16" ...
##  $ Censor                  : num  1 1 1 1 1 1 1 1 1 1 ...
##  $ Days_to_death_post_BM   : 'difftime' num  8 9 9 9 ...
##   ..- attr(*, "units")= chr "days"
##  $ Age_of_death            : 'difftime' num  11 12 12 12 ...
##   ..- attr(*, "units")= chr "days"
##  $ Age_of_BM_days          : 'difftime' num  3 3 3 3 ...
##   ..- attr(*, "units")= chr "days"
##  $ Date_of_eclosion        : POSIXct, format: "2024-02-04" "2024-02-04" ...
##  $ days_alive_post_BM      : num  8 9 9 9 10 11 11 11 12 12 ...
##  $ days_alive_post_eclosion: num  11 12 12 12 13 14 14 14 15 15 ...
##  $ start_time              : num  4 4 4 4 4 4 4 4 4 4 ...
##  $ stop_time               : num  12 13 13 13 14 15 15 15 16 16 ...
##  $ stopminusstart          : num  8 9 9 9 10 11 11 11 12 12 ...
```

```
bucket_survival_data_numeric$Age <- factor(bucket_survival_data_numeric$Age,
                                           labels = c("3","5","10","15"))
bucket_survival_data_numeric$Temperature <- factor(bucket_survival_data_numeric$Temperature,
                                                   labels = c("27","30","32"))
str(bucket_survival_data_numeric)
```

```
## 'data.frame':    1398 obs. of  22 variables:
##  $ ID_overall             : num  1 2 3 4 5 6 7 8 9 10 ...
##  $ Temperature            : Factor w/ 3 levels "27","30","32": 1 1 1 1 1 1 1 1 1 1 ...
##  $ Age                    : Factor w/ 4 levels "3","5","10","15": 1 1 1 1 1 1 1 1 1 1 ...
##  $ Age_of_BM              : num  3 3 3 3 3 3 3 3 3 3 ...
##  $ ID_per_group           : num  1 2 3 4 5 6 7 8 9 10 ...
##  $ Trial_start_date       : POSIXct, format: "2024-02-07" "2024-02-07" ...
##  $ Date_Group             : chr  "A" "A" "A" "A" ...
##  $ Trial_number           : num  1 1 1 1 1 1 1 1 1 1 ...
##  $ Initial_n              : num  35 35 35 35 35 35 35 35 35 35 ...
##  $ BM1_Date               : POSIXct, format: "2024-02-07" "2024-02-07" ...
##  $ Bloodmeal_number       : num  1 1 1 1 1 1 1 1 1 1 ...
##  $ Date_of_death          : POSIXct, format: "2024-02-15" "2024-02-16" ...
##  $ Censor                 : num  1 1 1 1 1 1 1 1 1 1 ...
##  $ Days_to_death_post_BM   : 'difftime' num  8 9 9 9 ...
##   ..- attr(*, "units")= chr "days"
##  $ Age_of_death           : 'difftime' num  11 12 12 12 ...
##   ..- attr(*, "units")= chr "days"
##  $ Age_of_BM_days         : 'difftime' num  3 3 3 3 ...
##   ..- attr(*, "units")= chr "days"
##  $ Date_of_eclosion       : POSIXct, format: "2024-02-04" "2024-02-04" ...
##  $ days_alive_post_BM     : num  8 9 9 9 10 11 11 11 12 12 ...
##  $ days_alive_post_eclosion: num  11 12 12 12 13 14 14 14 15 15 ...
##  $ start_time             : num  4 4 4 4 4 4 4 4 4 4 ...
##  $ stop_time              : num  12 13 13 13 14 15 15 15 16 16 ...
##  $ stopminusstart         : num  8 9 9 9 10 11 11 11 12 12 ...
```

```
bucket_survival_data_numeric$Age <- as.character(bucket_survival_data_numeric$Age)
bucket_survival_data_numeric$Age <- as.numeric(bucket_survival_data_numeric$Age)
str(bucket_survival_data_numeric)
```

```
## 'data.frame':	1398 obs. of  22 variables:
##  $ ID_overall             : num  1 2 3 4 5 6 7 8 9 10 ...
##  $ Temperature            : Factor w/ 3 levels "27","30","32": 1 1 1 1 1 1 1 1 1 1 ...
##  $ Age                    : num  3 3 3 3 3 3 3 3 3 3 ...
##  $ Age_of_BM              : num  3 3 3 3 3 3 3 3 3 3 ...
##  $ ID_per_group           : num  1 2 3 4 5 6 7 8 9 10 ...
##  $ Trial_start_date       : POSIXct, format: "2024-02-07" "2024-02-07" ...
##  $ Date_Group             : chr  "A" "A" "A" "A" ...
##  $ Trial_number           : num  1 1 1 1 1 1 1 1 1 1 ...
##  $ Initial_n              : num  35 35 35 35 35 35 35 35 35 35 ...
##  $ BM1_Date               : POSIXct, format: "2024-02-07" "2024-02-07" ...
##  $ Bloodmeal_number       : num  1 1 1 1 1 1 1 1 1 1 ...
##  $ Date_of_death          : POSIXct, format: "2024-02-15" "2024-02-16" ...
##  $ Censor                 : num  1 1 1 1 1 1 1 1 1 1 ...
##  $ Days_to_death_post_BM  : 'difftime' num  8 9 9 9 ...
##   ..- attr(*, "units")= chr "days"
##  $ Age_of_death           : 'difftime' num  11 12 12 12 ...
##   ..- attr(*, "units")= chr "days"
##  $ Age_of_BM_days         : 'difftime' num  3 3 3 3 ...
##   ..- attr(*, "units")= chr "days"
##  $ Date_of_eclosion       : POSIXct, format: "2024-02-04" "2024-02-04" ...
##  $ days_alive_post_BM     : num  8 9 9 9 10 11 11 11 12 12 ...
##  $ days_alive_post_eclosion: num  11 12 12 12 13 14 14 14 15 15 ...
##  $ start_time             : num  4 4 4 4 4 4 4 4 4 4 ...
##  $ stop_time              : num  12 13 13 13 14 15 15 15 16 16 ...
##  $ stopminusstart         : num  8 9 9 9 10 11 11 11 12 12 ...
```

```
range(bucket_survival_data_numeric$Age)
```

```
## [1]  3 15
```

```
str(bucket_survival_data_numeric)
```

```
## 'data.frame':    1398 obs. of  22 variables:
##  $ ID_overall              : num  1 2 3 4 5 6 7 8 9 10 ...
##  $ Temperature             : Factor w/ 3 levels "27","30","32": 1 1 1 1 1 1 1 1 1 1 ...
##  $ Age                     : num  3 3 3 3 3 3 3 3 3 3 ...
##  $ Age_of_BM               : num  3 3 3 3 3 3 3 3 3 3 ...
##  $ ID_per_group            : num  1 2 3 4 5 6 7 8 9 10 ...
##  $ Trial_start_date        : POSIXct, format: "2024-02-07" "2024-02-07" ...
##  $ Date_Group              : chr  "A" "A" "A" "A" ...
##  $ Trial_number            : num  1 1 1 1 1 1 1 1 1 1 ...
##  $ Initial_n               : num  35 35 35 35 35 35 35 35 35 35 ...
##  $ BM1_Date                : POSIXct, format: "2024-02-07" "2024-02-07" ...
##  $ Bloodmeal_number        : num  1 1 1 1 1 1 1 1 1 1 ...
##  $ Date_of_death           : POSIXct, format: "2024-02-15" "2024-02-16" ...
##  $ Censor                  : num  1 1 1 1 1 1 1 1 1 1 ...
##  $ Days_to_death_post_BM   : 'difftime' num  8 9 9 9 ...
##   ..- attr(*, "units")= chr "days"
##  $ Age_of_death            : 'difftime' num  11 12 12 12 ...
##   ..- attr(*, "units")= chr "days"
##  $ Age_of_BM_days          : 'difftime' num  3 3 3 3 ...
##   ..- attr(*, "units")= chr "days"
##  $ Date_of_eclosion        : POSIXct, format: "2024-02-04" "2024-02-04" ...
##  $ days_alive_post_BM      : num  8 9 9 9 10 11 11 11 12 12 ...
##  $ days_alive_post_eclosion: num  11 12 12 12 13 14 14 14 15 15 ...
##  $ start_time              : num  4 4 4 4 4 4 4 4 4 4 ...
##  $ stop_time               : num  12 13 13 13 14 15 15 15 16 16 ...
##  $ stopminusstart          : num  8 9 9 9 10 11 11 11 12 12 ...
```

```
bucket_survival_data_numeric$Temperature <- as.character(bucket_survival_data_numeric$Temperature)
bucket_survival_data_numeric$Temperature <- as.numeric(bucket_survival_data_numeric$Temperature)
str(bucket_survival_data_numeric)
```

```
## 'data.frame':    1398 obs. of  22 variables:
##  $ ID_overall             : num  1 2 3 4 5 6 7 8 9 10 ...
##  $ Temperature            : num  27 27 27 27 27 27 27 27 27 27 ...
##  $ Age                    : num  3 3 3 3 3 3 3 3 3 3 ...
##  $ Age_of_BM              : num  3 3 3 3 3 3 3 3 3 3 ...
##  $ ID_per_group           : num  1 2 3 4 5 6 7 8 9 10 ...
##  $ Trial_start_date       : POSIXct, format: "2024-02-07" "2024-02-07" ...
##  $ Date_Group             : chr  "A" "A" "A" "A" ...
##  $ Trial_number           : num  1 1 1 1 1 1 1 1 1 1 ...
##  $ Initial_n              : num  35 35 35 35 35 35 35 35 35 35 ...
##  $ BM1_Date               : POSIXct, format: "2024-02-07" "2024-02-07" ...
##  $ Bloodmeal_number       : num  1 1 1 1 1 1 1 1 1 1 ...
##  $ Date_of_death          : POSIXct, format: "2024-02-15" "2024-02-16" ...
##  $ Censor                 : num  1 1 1 1 1 1 1 1 1 1 ...
##  $ Days_to_death_post_BM  : 'difftime' num  8 9 9 9 ...
##   ..- attr(*, "units")= chr "days"
##  $ Age_of_death           : 'difftime' num  11 12 12 12 ...
##   ..- attr(*, "units")= chr "days"
##  $ Age_of_BM_days         : 'difftime' num  3 3 3 3 ...
##   ..- attr(*, "units")= chr "days"
##  $ Date_of_eclosion       : POSIXct, format: "2024-02-04" "2024-02-04" ...
##  $ days_alive_post_BM     : num  8 9 9 9 10 11 11 11 12 12 ...
##  $ days_alive_post_eclosion: num  11 12 12 12 13 14 14 14 15 15 ...
##  $ start_time             : num  4 4 4 4 4 4 4 4 4 4 ...
##  $ stop_time              : num  12 13 13 13 14 15 15 15 16 16 ...
##  $ stopminusstart         : num  8 9 9 9 10 11 11 11 12 12 ...
```

```
range(bucket_survival_data_numeric$Temperature)
```

```
## [1] 27 32
```

```
#Compare hazard ratios:
#treat variables as numeric so hazard shows with every increase in age(days) or temp (degrees)
bucket_survival_data_numeric$Trial_number <- as.factor(bucket_survival_data_numeric$Trial_number)
bucket_survival_data_numeric$Trial_start_date_factor <- as.factor(bucket_survival_data_numeric$Trial_start_
date)
str(bucket_survival_data_numeric$Trial_start_date_factor)
```

```
##  Factor w/ 33 levels "2024-02-07","2024-02-13",..: 1 1 1 1 1 1 1 1 1 1 ...
```

```
str(bucket_survival_data_numeric)
```

```
## 'data.frame':    1398 obs. of  23 variables:
##  $ ID_overall             : num  1 2 3 4 5 6 7 8 9 10 ...
##  $ Temperature            : num  27 27 27 27 27 27 27 27 27 27 ...
##  $ Age                    : num  3 3 3 3 3 3 3 3 3 3 ...
##  $ Age_of_BM              : num  3 3 3 3 3 3 3 3 3 3 ...
##  $ ID_per_group           : num  1 2 3 4 5 6 7 8 9 10 ...
##  $ Trial_start_date       : POSIXct, format: "2024-02-07" "2024-02-07" ...
##  $ Date_Group             : chr  "A" "A" "A" "A" ...
##  $ Trial_number           : Factor w/ 5 levels "1","2","3","4",..: 1 1 1 1 1 1 1 1 1 1 ...
##  $ Initial_n              : num  35 35 35 35 35 35 35 35 35 35 ...
##  $ BM1_Date               : POSIXct, format: "2024-02-07" "2024-02-07" ...
##  $ Bloodmeal_number       : num  1 1 1 1 1 1 1 1 1 1 ...
##  $ Date_of_death          : POSIXct, format: "2024-02-15" "2024-02-16" ...
##  $ Censor                 : num  1 1 1 1 1 1 1 1 1 1 ...
##  $ Days_to_death_post_BM   : 'difftime' num  8 9 9 9 ...
##   ..- attr(*, "units")= chr "days"
##  $ Age_of_death           : 'difftime' num  11 12 12 12 ...
##   ..- attr(*, "units")= chr "days"
##  $ Age_of_BM_days         : 'difftime' num  3 3 3 3 ...
##   ..- attr(*, "units")= chr "days"
##  $ Date_of_eclosion       : POSIXct, format: "2024-02-04" "2024-02-04" ...
##  $ days_alive_post_BM     : num  8 9 9 9 10 11 11 11 12 12 ...
##  $ days_alive_post_eclosion: num  11 12 12 12 13 14 14 14 15 15 ...
##  $ start_time             : num  4 4 4 4 4 4 4 4 4 4 ...
##  $ stop_time              : num  12 13 13 13 14 15 15 15 16 16 ...
##  $ stopminusstart         : num  8 9 9 9 10 11 11 11 12 12 ...
##  $ Trial_start_date_factor : Factor w/ 33 levels "2024-02-07","2024-02-13",..: 1 1 1 1 1 1 1 1 1 1 ...
```

```
bucket_survival_data_numeric$Trial_start_date_number <- as.numeric(bucket_survival_data_numeric$Trial_start
_date_factor)
str(bucket_survival_data_numeric)
```

```
## 'data.frame':    1398 obs. of  24 variables:
##  $ ID_overall              : num  1 2 3 4 5 6 7 8 9 10 ...
##  $ Temperature             : num  27 27 27 27 27 27 27 27 27 27 ...
##  $ Age                     : num  3 3 3 3 3 3 3 3 3 3 ...
##  $ Age_of_BM               : num  3 3 3 3 3 3 3 3 3 3 ...
##  $ ID_per_group            : num  1 2 3 4 5 6 7 8 9 10 ...
##  $ Trial_start_date        : POSIXct, format: "2024-02-07" "2024-02-07" ...
##  $ Date_Group              : chr  "A" "A" "A" "A" ...
##  $ Trial_number            : Factor w/ 5 levels "1","2","3","4",..: 1 1 1 1 1 1 1 1 1 1 ...
##  $ Initial_n               : num  35 35 35 35 35 35 35 35 35 35 ...
##  $ BM1_Date                : POSIXct, format: "2024-02-07" "2024-02-07" ...
##  $ Bloodmeal_number        : num  1 1 1 1 1 1 1 1 1 1 ...
##  $ Date_of_death           : POSIXct, format: "2024-02-15" "2024-02-16" ...
##  $ Censor                  : num  1 1 1 1 1 1 1 1 1 1 ...
##  $ Days_to_death_post_BM   : 'difftime' num  8 9 9 9 ...
##   ..- attr(*, "units")= chr "days"
##  $ Age_of_death            : 'difftime' num  11 12 12 12 ...
##   ..- attr(*, "units")= chr "days"
##  $ Age_of_BM_days          : 'difftime' num  3 3 3 3 ...
##   ..- attr(*, "units")= chr "days"
##  $ Date_of_eclosion        : POSIXct, format: "2024-02-04" "2024-02-04" ...
##  $ days_alive_post_BM      : num  8 9 9 9 10 11 11 11 12 12 ...
##  $ days_alive_post_eclosion: num  11 12 12 12 13 14 14 14 15 15 ...
##  $ start_time              : num  4 4 4 4 4 4 4 4 4 4 ...
##  $ stop_time               : num  12 13 13 13 14 15 15 15 16 16 ...
##  $ stopminusstart          : num  8 9 9 9 10 11 11 11 12 12 ...
##  $ Trial_start_date_factor : Factor w/ 33 levels "2024-02-07","2024-02-13",..: 1 1 1 1 1 1 1 1 1 1 ...
##  $ Trial_start_date_number : num  1 1 1 1 1 1 1 1 1 1 ...
```

```
#center and scale variables:
bucket_survival_data_numeric$Tempscaled <- scale(bucket_survival_data_numeric$Temperature, center=TRUE, sca
le=TRUE)
bucket_survival_data_numeric$Agescaled <- scale(bucket_survival_data_numeric$Age, center=TRUE, scale=TRUE)

str(bucket_survival_data_numeric$Tempscaled)
```

```
##  num [1:1398, 1] -1.31 -1.31 -1.31 -1.31 -1.31 ...
##  - attr(*, "scaled:center")= num 29.6
##  - attr(*, "scaled:scale")= num 1.97
```

```
str(bucket_survival_data_numeric$Agescaled)
```

```
##  num [1:1398, 1] -1.08 -1.08 -1.08 -1.08 -1.08 ...
##  - attr(*, "scaled:center")= num 8.11
##  - attr(*, "scaled:scale")= num 4.75
```

```
#check proportional hazards model
mv_fit <- coxph(Surv(stop_time, Censor) ~ Temperature*Age, data = bucket_survival_data_numeric)
#or
mv_fit <- coxph(Surv(stop_time, Censor) ~ Tempscaled*Agescaled, data = bucket_survival_data_numeric)
#or
mv_fit <- coxph(Surv(start_time,stop_time, Censor) ~ Temperature*Age+cluster(Trial_start_date_factor), robu
st=TRUE,
                data = bucket_survival_data_numeric,method="breslow")
cz <- cox.zph(mv_fit)
print(cz) # significant p-value indicates that the proportional hazards assumption is violated
```

```
##                  chisq df       p
## Temperature      0.104  1    0.75
## Age             49.781  1 1.7e-12
## Temperature:Age 47.479  1 5.6e-12
## GLOBAL          51.038  3 4.8e-11
```

```
plot(cz) #violated for age, temp*age, global - non-proportional
```

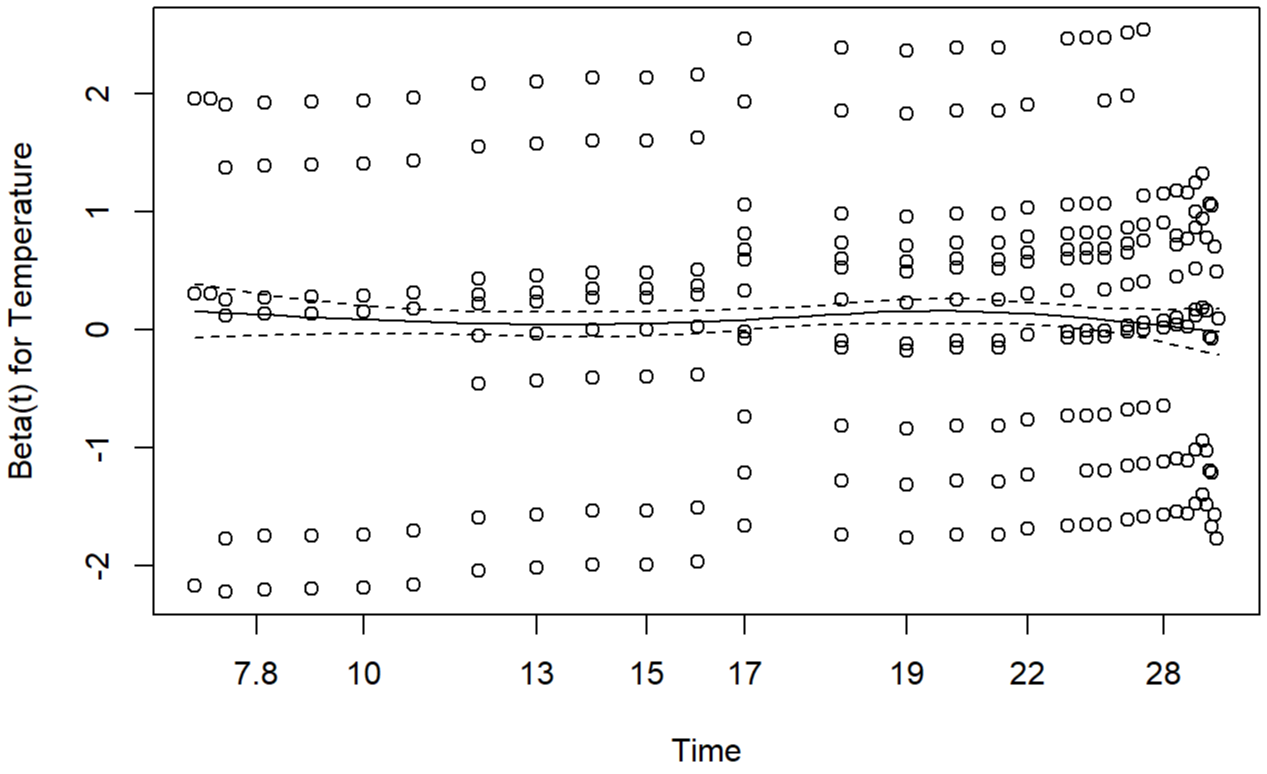

Beta(t) for Temperature
2
1
0
-1
-2
7.8
10
13
15
17
19
22
28
Time

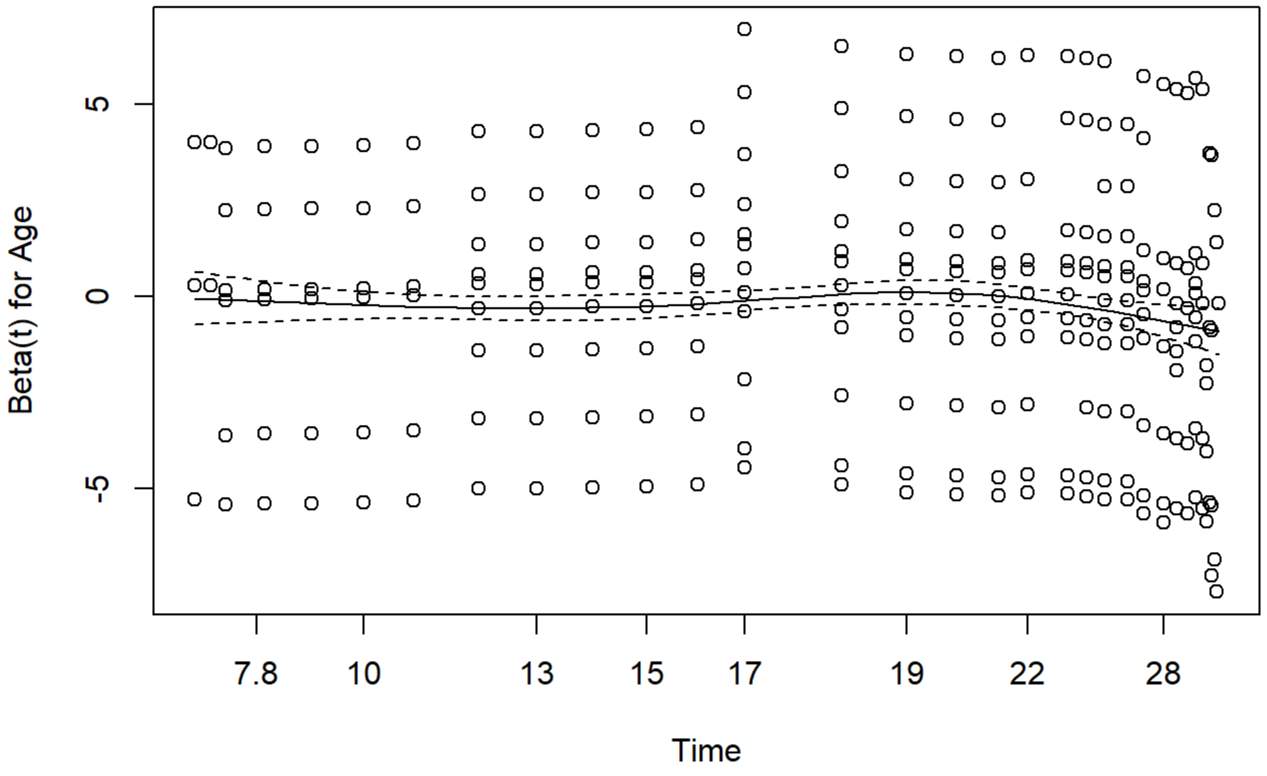

Beta(t) for Age
5
0
-5
7.8
10
13
15
17
19
22
28
Time

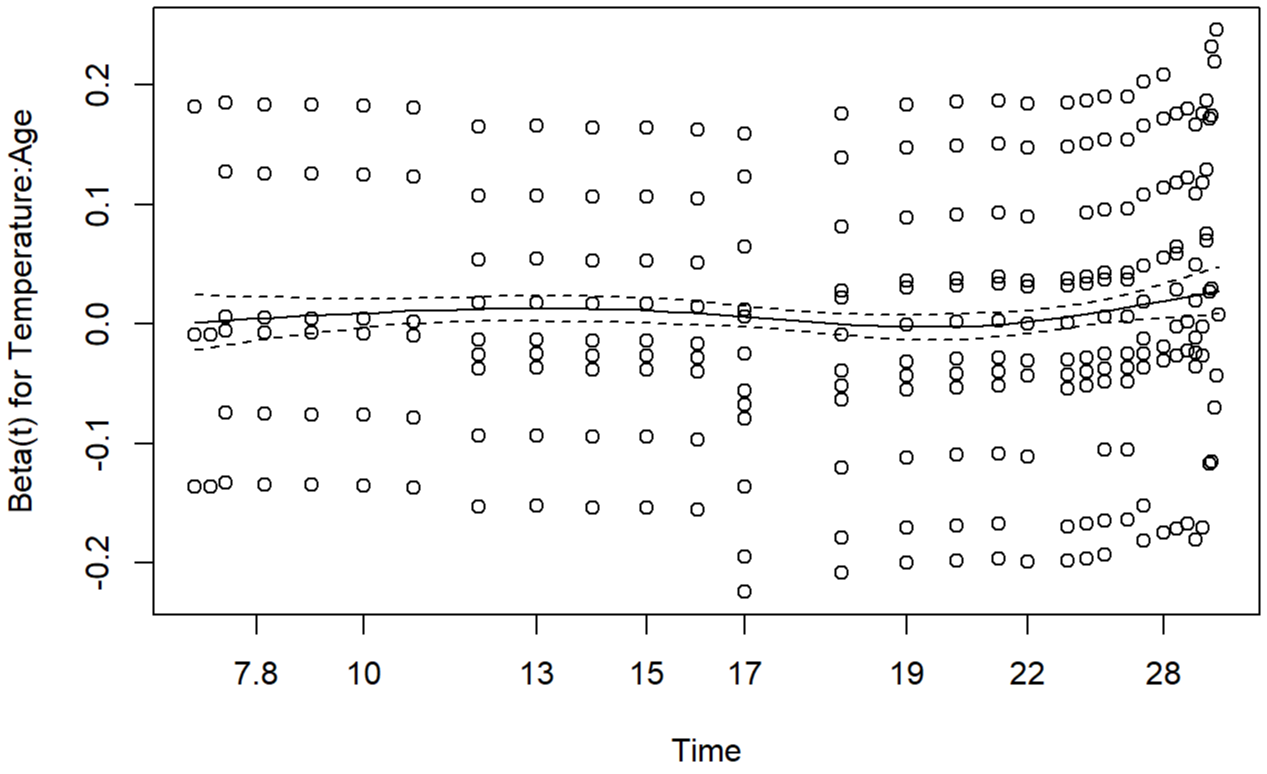


```
#assumptions violated.
###
#non-proportional hazards:
#coxph regression with weighted estimation, accounting for experimental block

library(condsurv)
library(coxphw)
fit1 <- coxphw(Surv(start_time,stop_time, Censor) ~ Temperature*Age +
                 frailty(Trial_start_date_factor,distribution = "gaussian"),
               data = bucket_survival_data_numeric,
               template = "AHR")

summary(fit1)
```

```
## coxphw(formula = Surv(start_time, stop_time, Censor) ~ Temperature *
##     Age + frailty(Trial_start_date_factor, distribution = "gaussian"),
##     data = bucket_survival_data_numeric, template = "AHR")
##
## Model fitted by weighted estimation (AHR template)
##
##                                                                      coef
## Temperature                                                   0.103830695
## Age                                                          -0.060568231
## frailty(Trial_start_date_factor, distribution = "gaussian")  0.005922331
## Temperature:Age                                               0.004311410
##                                                                 se(coef)
## Temperature                                                  0.032231490
## Age                                                          0.098119713
## frailty(Trial_start_date_factor, distribution = "gaussian") 0.003409579
## Temperature:Age                                              0.003286059
##                                                              exp(coef)
## Temperature                                                  1.1094126
## Age                                                          0.9412295
## frailty(Trial_start_date_factor, distribution = "gaussian") 1.0059399
## Temperature:Age                                              1.0043207
##                                                              lower 0.95
## Temperature                                                   1.0414960
## Age                                                           0.7765616
## frailty(Trial_start_date_factor, distribution = "gaussian")  0.9992400
## Temperature:Age                                               0.9978731
##                                                              upper 0.95
## Temperature                                                    1.181758
## Age                                                            1.140815
## frailty(Trial_start_date_factor, distribution = "gaussian")   1.012685
## Temperature:Age                                                1.010810
##                                                                       z
## Temperature                                                   3.2214054
## Age                                                          -0.6172891
## frailty(Trial_start_date_factor, distribution = "gaussian")  1.7369685
## Temperature:Age                                               1.3120307
##                                                                        p
## Temperature                                                  0.001275636
## Age                                                          0.537044043
## frailty(Trial_start_date_factor, distribution = "gaussian") 0.082392724
## Temperature:Age                                              0.189509782
##
## Wald Chi-square = 206.1528 on 4  df  p = 0  n = 1398
##
## Covariance-Matrix:
##                                                                Temperature
## Temperature                                                   1.038869e-03
## Age                                                           2.682925e-03
## frailty(Trial_start_date_factor, distribution = "gaussian") -2.970047e-06
## Temperature:Age                                              -9.066049e-05
##                                                                        Age
## Temperature                                                   2.682925e-03
## Age                                                           9.627478e-03
## frailty(Trial_start_date_factor, distribution = "gaussian")  6.601079e-05
## Temperature:Age                                              -3.216012e-04
##                                                              frailty(Trial_start_date_factor, distributio
```

```
n = "gaussian")
## Temperature
-2.970047e-06
## Age
6.601079e-05
## frailty(Trial_start_date_factor, distribution = "gaussian")
1.162523e-05
## Temperature:Age
-2.045844e-06
##                                                               Temperature:Age
## Temperature                                                     -9.066049e-05
## Age                                                             -3.216012e-04
## frailty(Trial_start_date_factor, distribution = "gaussian")   -2.045844e-06
## Temperature:Age                                                  1.079818e-05
##
## Generalized concordance probability:
##                                                               concordance prob.
## Temperature                                                              0.5259
## Age                                                                      0.4849
## frailty(Trial_start_date_factor, distribution = "gaussian")           0.5015
## Temperature:Age                                                          0.5011
##                                                               lower 0.95
## Temperature                                                       0.5102
## Age                                                               0.4371
## frailty(Trial_start_date_factor, distribution = "gaussian")     0.4998
## Temperature:Age                                                   0.4995
##                                                               upper 0.95
## Temperature                                                       0.5417
## Age                                                               0.5329
## frailty(Trial_start_date_factor, distribution = "gaussian")     0.5032
## Temperature:Age                                                   0.5027
```

```
plot(fit1$dfbeta.resid) # residuals are bad
```

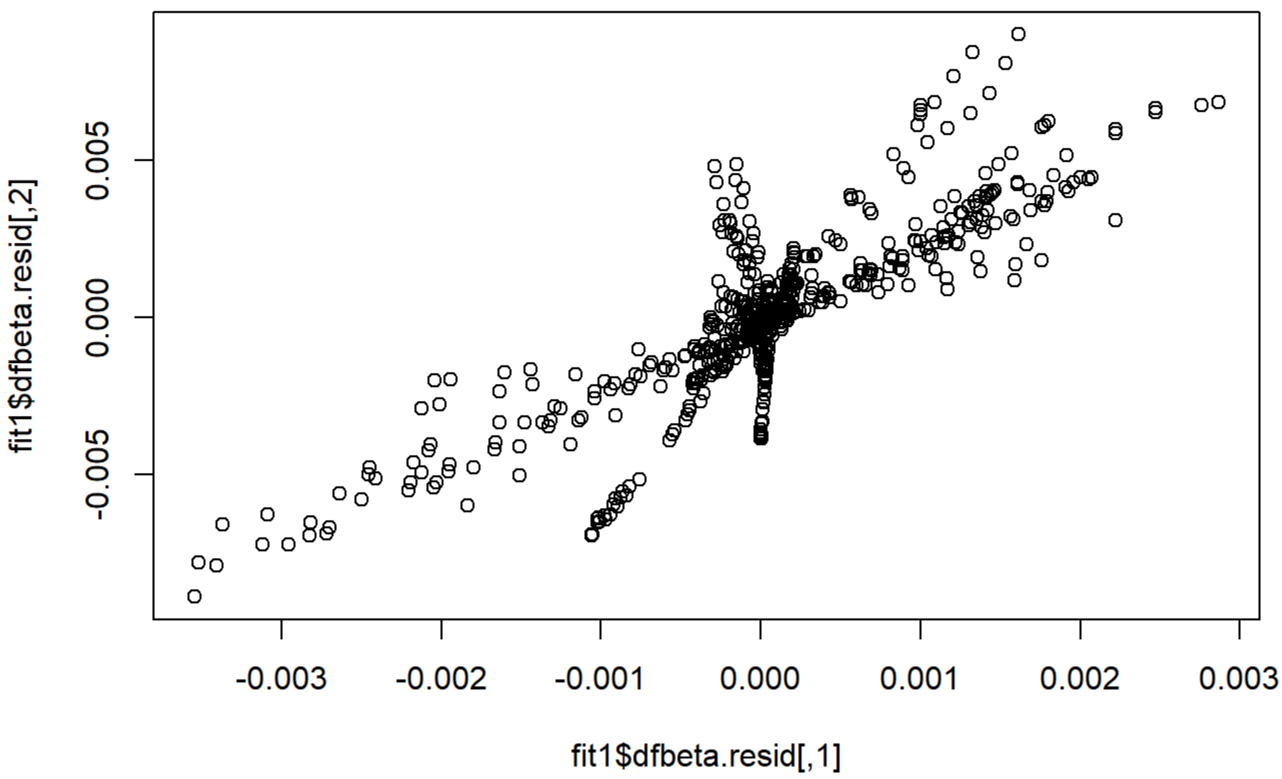


```
#residuals are bad - try centering and scaling temperature and age numbers

#scale and center independent numeric variables:
fit1 <- coxphw(Surv(start_time,stop_time, Censor) ~ Tempscaled*Agescaled +
                 frailty(Trial_start_date_factor,distribution = "gaussian"),
               data = bucket_survival_data_numeric,
               template = "AHR")
summary(fit1)
```

```
## coxphw(formula = Surv(start_time, stop_time, Censor) ~ Tempscaled *
##     Agescaled + frailty(Trial_start_date_factor, distribution = "gaussian"),
##     data = bucket_survival_data_numeric, template = "AHR")
##
## Model fitted by weighted estimation (AHR template)
##
##                                                                    coef
## Tempscaled                                                  0.273954753
## Agescaled                                                   0.317998819
## frailty(Trial_start_date_factor, distribution = "gaussian") 0.005922331
## Tempscaled:Agescaled                                        0.040391647
##                                                                se(coef)
## Tempscaled                                                  0.032950444
## Agescaled                                                   0.033444845
## frailty(Trial_start_date_factor, distribution = "gaussian") 0.003409579
## Tempscaled:Agescaled                                        0.030785596
##                                                             exp(coef)
## Tempscaled                                                   1.315155
## Agescaled                                                    1.374375
## frailty(Trial_start_date_factor, distribution = "gaussian")  1.005940
## Tempscaled:Agescaled                                         1.041218
##                                                             lower 0.95
## Tempscaled                                                   1.2329049
## Agescaled                                                    1.2871727
## frailty(Trial_start_date_factor, distribution = "gaussian")  0.9992400
## Tempscaled:Agescaled                                         0.9802506
##                                                             upper 0.95        z
## Tempscaled                                                    1.402893 8.314144
## Agescaled                                                     1.467484 9.508157
## frailty(Trial_start_date_factor, distribution = "gaussian")   1.012685 1.736969
## Tempscaled:Agescaled                                          1.105978 1.312031
##                                                                        p
## Tempscaled                                                  1.110223e-16
## Agescaled                                                   0.000000e+00
## frailty(Trial_start_date_factor, distribution = "gaussian") 8.239272e-02
## Tempscaled:Agescaled                                        1.895098e-01
##
## Wald Chi-square = 206.1528 on 4  df  p = 0  n = 1398
##
## Covariance-Matrix:
##                                                                Tempscaled
## Tempscaled                                                   1.085732e-03
## Agescaled                                                   -1.555282e-04
## frailty(Trial_start_date_factor, distribution = "gaussian") -3.859723e-05
## Tempscaled:Agescaled                                        -5.806446e-05
##                                                                 Agescaled
## Tempscaled                                                  -1.555282e-04
## Agescaled                                                    1.118558e-03
## frailty(Trial_start_date_factor, distribution = "gaussian")  2.597822e-05
## Tempscaled:Agescaled                                        -9.254643e-05
##                                                             frailty(Trial_start_date_factor, distributio
n = "gaussian")
## Tempscaled
-3.859723e-05
## Agescaled
2.597822e-05
```

```
## frailty(Trial_start_date_factor, distribution = "gaussian")
1.162523e-05
## Tempscaled:Agescaled
-1.916659e-05
##                                                             Tempscaled:Agescaled
## Tempscaled                                                         -5.806446e-05
## Agescaled                                                          -9.254643e-05
## frailty(Trial_start_date_factor, distribution = "gaussian")        -1.916659e-05
## Tempscaled:Agescaled                                                9.477529e-04
##
## Generalized concordance probability:
##                                                             concordance prob.
## Tempscaled                                                             0.5681
## Agescaled                                                              0.5788
## frailty(Trial_start_date_factor, distribution = "gaussian")            0.5015
## Tempscaled:Agescaled                                                   0.5101
##                                                             lower 0.95
## Tempscaled                                                      0.5522
## Agescaled                                                       0.5628
## frailty(Trial_start_date_factor, distribution = "gaussian")     0.4998
## Tempscaled:Agescaled                                            0.4950
##                                                             upper 0.95
## Tempscaled                                                      0.5838
## Agescaled                                                       0.5947
## frailty(Trial_start_date_factor, distribution = "gaussian")     0.5032
## Tempscaled:Agescaled                                            0.5252
```

```
plot(fit1$dfbeta.resid) # looks good!
```

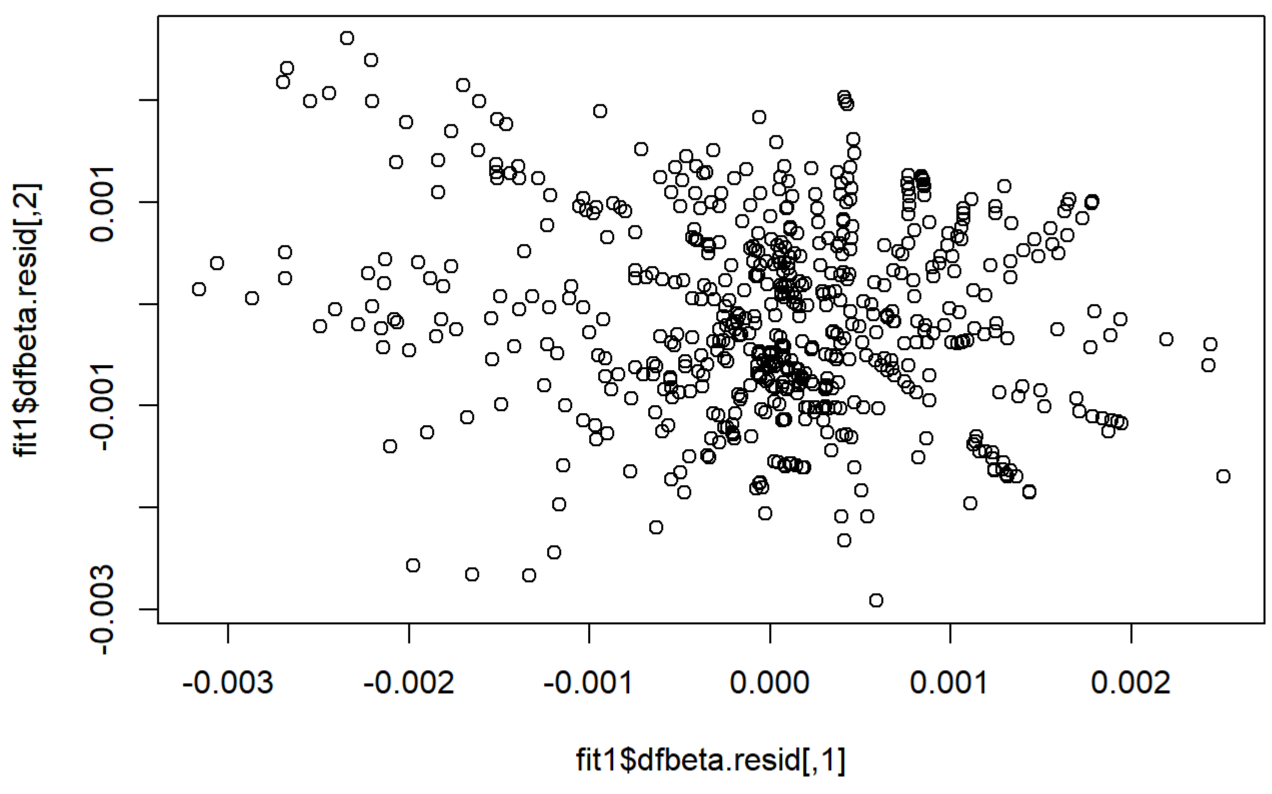

```r
fit1
```

```
## coxphw(formula = Surv(start_time, stop_time, Censor) ~ Tempscaled *
##     Agescaled + frailty(Trial_start_date_factor, distribution = "gaussian"),
##     data = bucket_survival_data_numeric, template = "AHR")
##
## Model fitted by weighted estimation (AHR template)
##
##                                                                     coef
## Tempscaled                                                   0.273954753
## Agescaled                                                    0.317998819
## frailty(Trial_start_date_factor, distribution = "gaussian") 0.005922331
## Tempscaled:Agescaled                                         0.040391647
##                                                                 se(coef)
## Tempscaled                                                   0.032950444
## Agescaled                                                    0.033444845
## frailty(Trial_start_date_factor, distribution = "gaussian") 0.003409579
## Tempscaled:Agescaled                                         0.030785596
##                                                              exp(coef)
## Tempscaled                                                    1.315155
## Agescaled                                                     1.374375
## frailty(Trial_start_date_factor, distribution = "gaussian")  1.005940
## Tempscaled:Agescaled                                          1.041218
##                                                              lower 0.95
## Tempscaled                                                    1.2329049
## Agescaled                                                     1.2871727
## frailty(Trial_start_date_factor, distribution = "gaussian")  0.9992400
## Tempscaled:Agescaled                                          0.9802506
##                                                              upper 0.95        z
## Tempscaled                                                     1.402893 8.314144
## Agescaled                                                      1.467484 9.508157
## frailty(Trial_start_date_factor, distribution = "gaussian")   1.012685 1.736969
## Tempscaled:Agescaled                                           1.105978 1.312031
##                                                                         p
## Tempscaled                                                   1.110223e-16
## Agescaled                                                    0.000000e+00
## frailty(Trial_start_date_factor, distribution = "gaussian") 8.239272e-02
## Tempscaled:Agescaled                                         1.895098e-01
##
## Wald Chi-square=206.1528 on 4df, p=0, n=1398
```

```r
plot(fit1)
```

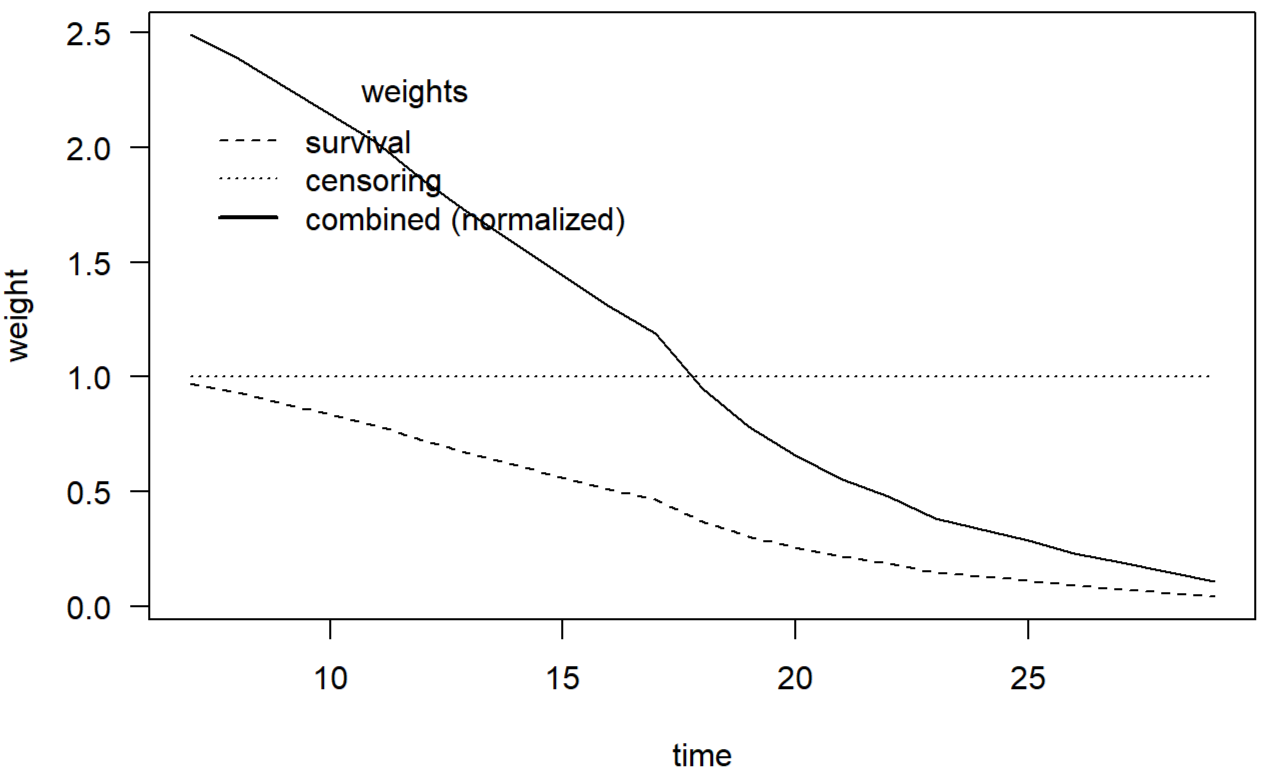


```
#go with this model.

sink("Bucket_survival/firstBM_coxmodel_bucketsurvival.txt")
fit1
summary(fit1)
sink()

#extract coefficients to plot hazard ratios:
#inspect at each point to make sure code is correctly extracting.
coef <- as.numeric(fit1$coefficients[1:4])
expcoef <- as.numeric(exp(fit1$coefficients[1:4]))
names <- names(fit1$coefficients[1:4])
lowerCI <- as.numeric(fit1$ci.lower[1:4])
upperCI <- as.numeric(fit1$ci.upper[1:4])
pval <- as.numeric(fit1$prob[1:4])
Index <- c(1:3)
Label <- names(fit1$coefficients[1:4])

hazardratiotable <- as.data.frame(cbind(Label,coef,expcoef,lowerCI,upperCI,pval))

hazardratiotable <- hazardratiotable[-3,]

hazardratiotable <- as.data.frame(cbind(Index,hazardratiotable))

print(hazardratiotable)
```

```
##   Index                Label                coef          expcoef
## 1     1           Tempscaled  0.273954752857555 1.31515529387352
## 2     2            Agescaled  0.317998819053278 1.37437463816338
## 4     3 Tempscaled:Agescaled 0.0403916471187162 1.04121848456731
##             lowerCI          upperCI                 pval
## 1  1.23290487171645 1.40289286439072 1.11022302462516e-16
## 2  1.28717272591382  1.4674842062752                    0
## 4 0.980250612439383 1.10597832722262    0.189509781291393
```

```
str(hazardratiotable)
```

```
## 'data.frame':    3 obs. of  7 variables:
##  $ Index  : int  1 2 3
##  $ Label  : chr  "Tempscaled" "Agescaled" "Tempscaled:Agescaled"
##  $ coef   : chr  "0.273954752857555" "0.317998819053278" "0.0403916471187162"
##  $ expcoef: chr  "1.31515529387352" "1.37437463816338" "1.04121848456731"
##  $ lowerCI: chr  "1.23290487171645" "1.28717272591382" "0.980250612439383"
##  $ upperCI: chr  "1.40289286439072" "1.4674842062752" "1.10597832722262"
##  $ pval   : chr  "1.11022302462516e-16" "0" "0.189509781291393"
```

```
#round numbers in table:
hazardratiotable$coef <- round(as.numeric(hazardratiotable$coef),digits = 3)
hazardratiotable$expcoef <- round(as.numeric(hazardratiotable$expcoef),digits = 3)
hazardratiotable$lowerCI <- round(as.numeric(hazardratiotable$lowerCI),digits = 3)
hazardratiotable$upperCI <- round(as.numeric(hazardratiotable$upperCI),digits = 3)
hazardratiotable$pval <- round(as.numeric(hazardratiotable$pval),digits = 3)
print(hazardratiotable)
```

```
##   Index                Label  coef expcoef lowerCI upperCI pval
## 1     1           Tempscaled 0.274   1.315   1.233   1.403 0.00
## 2     2            Agescaled 0.318   1.374   1.287   1.467 0.00
## 4     3 Tempscaled:Agescaled 0.040   1.041   0.980   1.106 0.19
```

```
print(hazardratiotable)
```

```
##   Index                Label  coef expcoef lowerCI upperCI pval
## 1     1           Tempscaled 0.274   1.315   1.233   1.403 0.00
## 2     2            Agescaled 0.318   1.374   1.287   1.467 0.00
## 4     3 Tempscaled:Agescaled 0.040   1.041   0.980   1.106 0.19
```

```
hazardratiotable$Label <- c("Temperature","Age","Temperature:Age")
hazardratiotable$pval[1]<-"< 0.001" #make 0 value less than 0.001
hazardratiotable$pval[2]<-"< 0.001" #make 0 value less than 0.001



hazardratiotable
```

```
##   Index           Label  coef expcoef lowerCI upperCI    pval
## 1     1     Temperature 0.274   1.315   1.233   1.403 < 0.001
## 2     2             Age 0.318   1.374   1.287   1.467 < 0.001
## 4     3 Temperature:Age 0.040   1.041   0.980   1.106    0.19
```

```
write_xlsx(hazardratiotable,"Bucket_survival/firstBM_bucketsurvival_hazardratiotable.xlsx")
```

Plot hazard ratio forest plot.

```
## Plot forest plot
#https://rpubs.com/mbounthavong/forest_plots_r
plot1 <- ggplot(hazardratiotable, aes(y = Index, x = expcoef)) +
  geom_point(shape = 18, size =4) +
  geom_errorbarh(aes(xmin = lowerCI, xmax = upperCI), height = 0.25) +
  geom_vline(xintercept = 1, color = "gray", linetype = "dashed", cex = 1, alpha = 0.5) +
  scale_y_continuous(name = "",breaks = 1:3, labels = hazardratiotable$Label,  trans = "reverse") +
  xlab("Hazard Ratio (95% CI)") +
  ylab(" ") +
  theme_pubr() +
  theme(panel.border = element_blank(),
        panel.background = element_blank(),
        panel.grid.major = element_blank(),
        panel.grid.minor = element_blank(),
        axis.line = element_line(colour = "black"),
        axis.text.y = element_text(size = 12, colour = "black"),
        axis.text.x.bottom = element_text(size = 12, colour = "black"),
        axis.title.x = element_text(size = 12, colour = "black"))
```

```
## Warning: Using `size` aesthetic for lines was deprecated in ggplot2 3.4.0.
## ℹ Please use `linewidth` instead.
## This warning is displayed once every 8 hours.
## Call `lifecycle::last_lifecycle_warnings()` to see where this warning was
## generated.
```

```
plot1
```

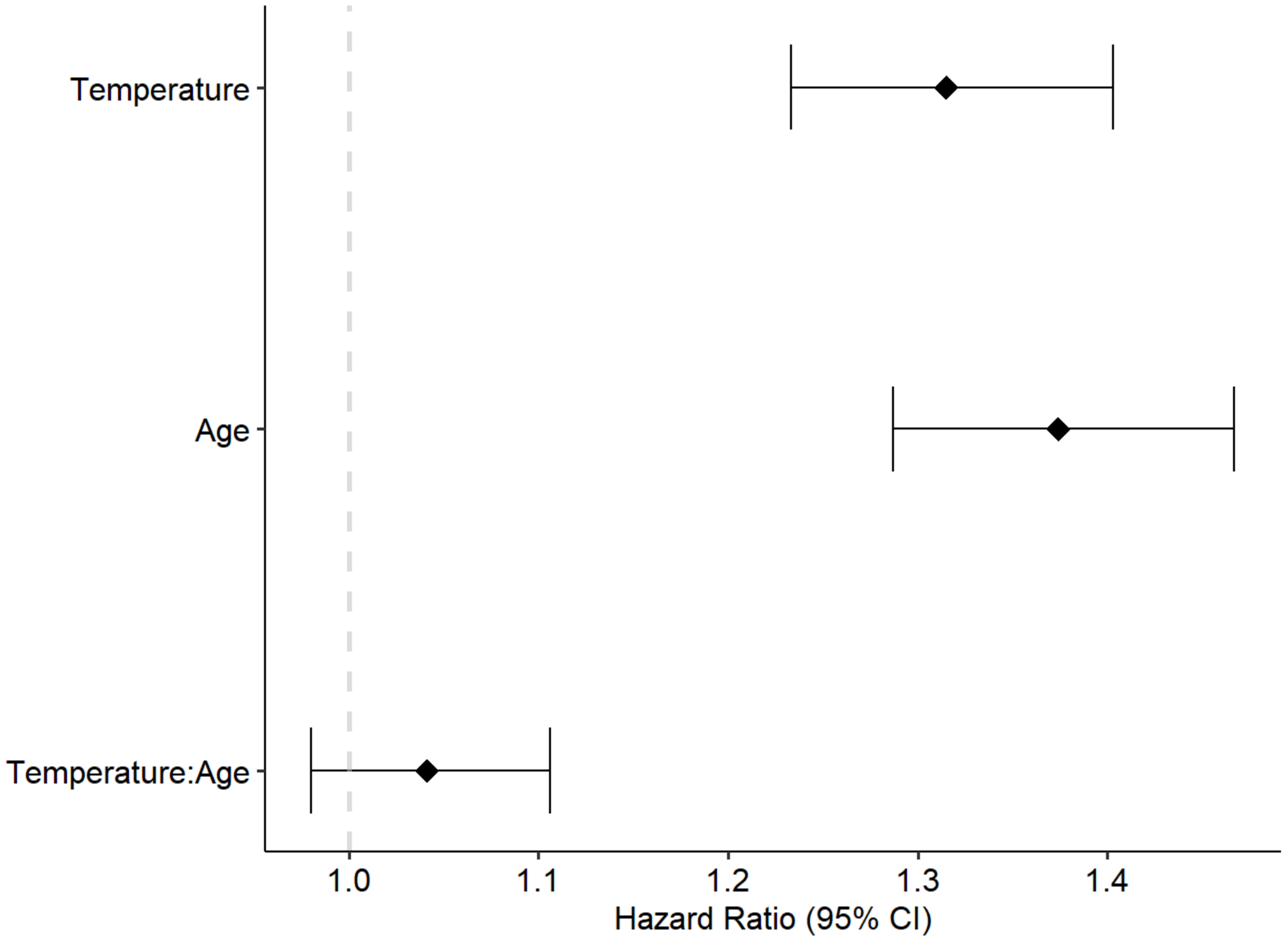


```
#add CIs to plot:

## Create the table-base pallete
table_base <- ggplot(hazardratiotable, aes(y=Label)) +
  ylab(NULL) + xlab("  ") +
  theme(plot.title = element_text(hjust = 0.5, size=12),
        axis.text.x = element_text(color="white", hjust = -3, size = 25), ## This is used to help with alig
nment
        axis.line = element_blank(),
        axis.text.y = element_blank(),
        axis.ticks = element_blank(),
        axis.title.y = element_blank(),
        legend.position = "none",
        panel.background = element_blank(),
        panel.border = element_blank(),
        panel.grid.major = element_blank(),
        panel.grid.minor = element_blank(),
        plot.background = element_blank())

## HR point estimate table
tab1 <- table_base +
  labs(title = "space") +
  geom_text(aes(y = rev(Index), x = 1, label = sprintf("%0.1f", round(expcoef, digits = 1))), size = 4) + #
# decimal places
  ggtitle("HR")
tab1
```

HR

1.3

1.4

1.0

```
#pval
tab3 <- table_base +
  geom_text(aes(y = rev(Index), x = 1, label = pval), size = 4) +
  ggtitle("P value")
tab3
```

P value
< 0.001
< 0.001
0.19

```
## Merge tables with plot
lay <-  matrix(c(1,1,1,1,1,1,1,1,1,1,2,2,2), nrow = 1)
bucketsurvival_forest <- grid.arrange(plot1, tab3,layout_matrix = lay)
```

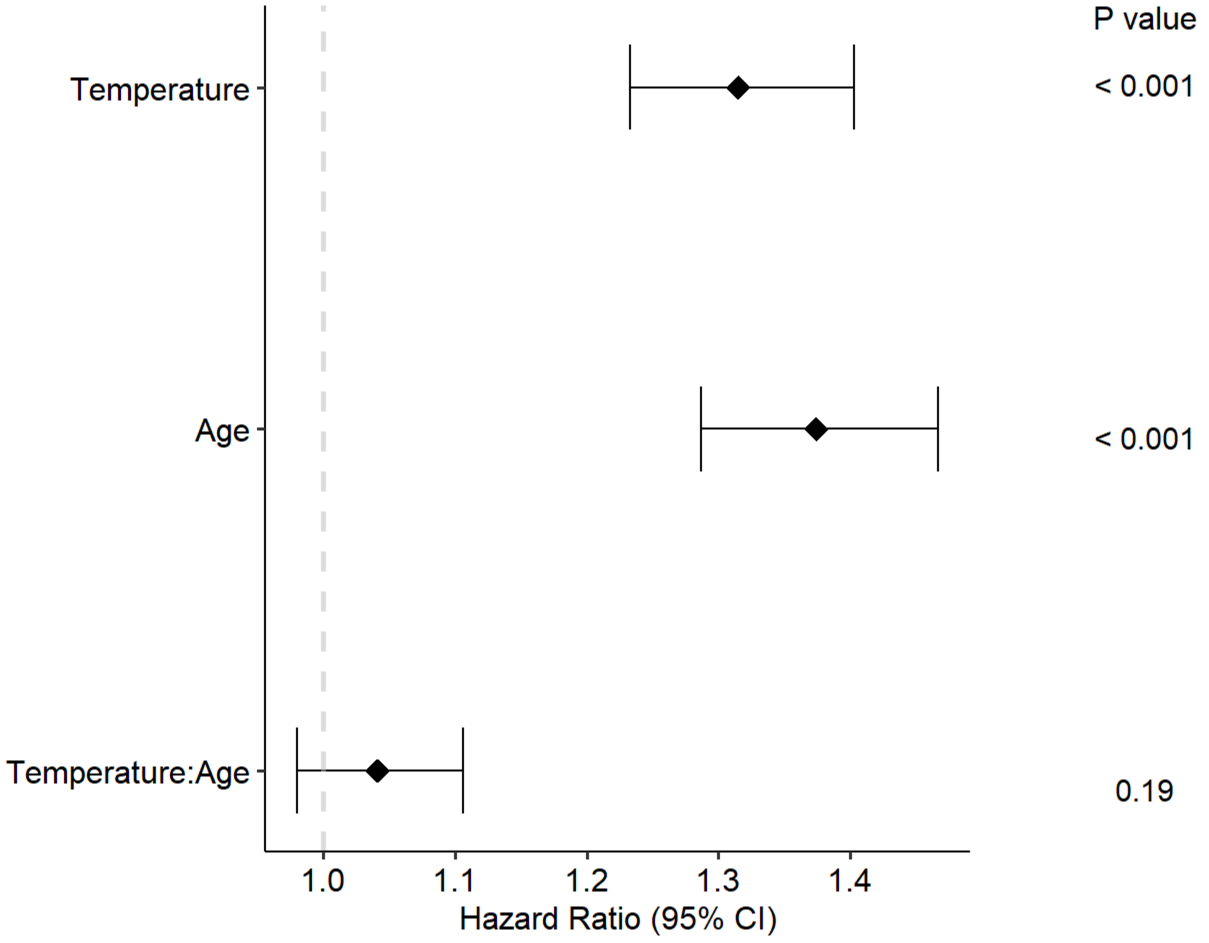


```
ggsave("Bucket_survival/firstBM_bucketsurvival_forest_plot.png",bucketsurvival_forest,dpi=600,width = 6,hei
ght=4,units="in")
ggsave("Bucket_survival/firstBM_bucketsurvival_forest_plot.pdf",bucketsurvival_forest,dpi=600,width = 6,hei
ght=4,units="in")
```

Code for Figure 4: Oviposition success- binary proportions of mosquitoes laying eggs (did or did not lay eggs)

Import data, clean it up, calculate oviposition success, survival, etc.

```
#do not filter to only show eggs >1! only remove NA (dead).
################
# clear existing workspace
rm(list = ls(all = TRUE))
graphics.off()
shell("cls")

#set wd to your project folder
getwd() #check working directory
```

```
## [1] "C:/Users/linzm/OneDrive - Vanderbilt/Hillyer_Lab/Blood_feeding_project/Bloodfeeding"
```

```r
#sessionInfo()
###########################

#####################
#load libraries needed:
library(readxl)
library(writexl)
library(ggplot2)
library(dplyr)
library(tidyverse)
library(rstatix)
library(car)
library(ggpubr)
#library(lme4)
#library(Rcpp)
#library(lmerTest)
#library(effects)
#library(broom)
#library(fitdistrplus)
library(emmeans)


#######################################################
#import the data and clean it up:



#import the data:
Fecundity_data <- read_xlsx("SupplementaryData1_RawData.xlsx",
                             sheet = "Figs4-9")
Fecundity_data <- as.data.frame(Fecundity_data)
str(Fecundity_data)
```

```
## 'data.frame':    849 obs. of  18 variables:
##  $ ID_overall               : num  1 2 3 4 5 6 7 8 9 10 ...
##  $ Temperature              : num  32 32 32 32 32 32 32 32 32 32 ...
##  $ Age                      : num  3 3 3 3 3 3 3 3 3 3 ...
##  $ ID_per_group             : num  1 2 3 4 5 6 7 8 9 10 ...
##  $ Trial_start_date         : POSIXct, format: "2024-02-13" "2024-02-13" ...
##  $ Trial_number             : num  1 1 1 1 1 1 1 1 1 1 ...
##  $ BM1_Date                 : POSIXct, format: "2024-02-13" "2024-02-13" ...
##  $ Age_of_BM                : num  3 3 3 3 3 3 3 3 3 3 ...
##  $ Bloodmeal_number         : num  1 1 1 1 1 1 1 1 1 1 ...
##  $ Oviposition_positive(y/n): chr  "Y" "Y" "Y" "Y" ...
##  $ Eggs_day3                : chr  "0" "0" "0" "0" ...
##  $ Eggs_day4                : chr  "0" "0" "0" "0" ...
##  $ Larvae_day4              : chr  "0" "0" "0" "0" ...
##  $ Surv_to_eggs(y/n)        : chr  "Y" "Y" "Y" "Y" ...
##  $ Surv_to_larvae(y/n)      : chr  "Y" "Y" "Y" "Y" ...
##  $ Date_of_death            : POSIXct, format: "2024-03-01" "2024-03-07" ...
##  $ Censor                   : num  1 1 1 1 1 1 1 1 1 1 ...
##  $ Notes                    : chr  NA NA NA NA ...
```

```r
head(Fecundity_data)
```

```
##   ID_overall Temperature Age ID_per_group Trial_start_date Trial_number
## 1          1          32   3            1       2024-02-13            1
## 2          2          32   3            2       2024-02-13            1
## 3          3          32   3            3       2024-02-13            1
## 4          4          32   3            4       2024-02-13            1
## 5          5          32   3            5       2024-02-13            1
## 6          6          32   3            6       2024-02-13            1
##     BM1_Date Age_of_BM Bloodmeal_number Oviposition_positive(y/n) Eggs_day3
## 1 2024-02-13         3                1                         Y         0
## 2 2024-02-13         3                1                         Y         0
## 3 2024-02-13         3                1                         Y         0
## 4 2024-02-13         3                1                         Y         0
## 5 2024-02-13         3                1                         Y         0
## 6 2024-02-13         3                1                         Y        14
##   Eggs_day4 Larvae_day4 Surv_to_eggs(y/n) Surv_to_larvae(y/n) Date_of_death
## 1         0           0                 Y                   Y    2024-03-01
## 2         0           0                 Y                   Y    2024-03-07
## 3         0           0                 Y                   Y    2024-03-02
## 4         0           0                 Y                   Y    2024-02-26
## 5         0           0                 Y                   Y    2024-02-28
## 6        14           0                 Y                   Y    2024-03-02
##   Censor Notes
## 1      1  <NA>
## 2      1  <NA>
## 3      1  <NA>
## 4      1  <NA>
## 5      1  <NA>
## 6      1  <NA>
```

```
Fecundity_data_numeric <- Fecundity_data

#variables of interest:
Fecundity_data$Temperature <- as.factor(Fecundity_data$Temperature)
Fecundity_data$Age <- as.factor(Fecundity_data$Age)
Fecundity_data$Age_of_BM <- as.numeric(Fecundity_data$Age_of_BM)
Fecundity_data$Bloodmeal_number <- as.factor(Fecundity_data$Bloodmeal_number)

Fecundity_data$Eggs_day3 <- as.numeric(Fecundity_data$Eggs_day3)
```

```
## Warning: NAs introduced by coercion
```

```
Fecundity_data$Eggs_day4 <- as.numeric(Fecundity_data$Eggs_day4)
```

```
## Warning: NAs introduced by coercion
```

```
Fecundity_data$Larvae_day4 <- as.numeric(Fecundity_data$Larvae_day4)
```

```
## Warning: NAs introduced by coercion
```

```r
Fecundity_data$Oviposition_positive <- as.factor(Fecundity_data$Oviposition_positive)
Fecundity_data$`Surv_to_eggs(y/n)` <- as.factor(Fecundity_data$`Surv_to_eggs(y/n)`)
Fecundity_data$`Surv_to_larvae(y/n)` <- as.factor(Fecundity_data$`Surv_to_larvae(y/n)`)

str(Fecundity_data)
```

```
## 'data.frame':	849 obs. of  19 variables:
##  $ ID_overall              : num  1 2 3 4 5 6 7 8 9 10 ...
##  $ Temperature             : Factor w/ 3 levels "27","30","32": 3 3 3 3 3 3 3 3 3 3 ...
##  $ Age                     : Factor w/ 4 levels "3","5","10","15": 1 1 1 1 1 1 1 1 1 1 ...
##  $ ID_per_group            : num  1 2 3 4 5 6 7 8 9 10 ...
##  $ Trial_start_date        : POSIXct, format: "2024-02-13" "2024-02-13" ...
##  $ Trial_number            : num  1 1 1 1 1 1 1 1 1 1 ...
##  $ BM1_Date                : POSIXct, format: "2024-02-13" "2024-02-13" ...
##  $ Age_of_BM               : num  3 3 3 3 3 3 3 3 3 3 ...
##  $ Bloodmeal_number        : Factor w/ 1 level "1": 1 1 1 1 1 1 1 1 1 1 ...
##  $ Oviposition_positive(y/n): chr  "Y" "Y" "Y" "Y" ...
##  $ Eggs_day3               : num  0 0 0 0 0 14 NA NA NA NA ...
##  $ Eggs_day4               : num  0 0 0 0 0 14 NA NA NA NA ...
##  $ Larvae_day4             : num  0 0 0 0 0 0 0 0 NA NA ...
##  $ Surv_to_eggs(y/n)       : Factor w/ 3 levels "N","NA","Y": 3 3 3 3 3 3 1 1 3 3 ...
##  $ Surv_to_larvae(y/n)     : Factor w/ 3 levels "N","NA","Y": 3 3 3 3 3 3 2 2 3 3 ...
##  $ Date_of_death           : POSIXct, format: "2024-03-01" "2024-03-07" ...
##  $ Censor                  : num  1 1 1 1 1 1 1 1 1 1 ...
##  $ Notes                   : chr  NA NA NA NA ...
##  $ Oviposition_positive    : Factor w/ 2 levels "N","Y": 2 2 2 2 2 2 2 2 1 1 ...
```

```r
Fecundity_data <- subset(Fecundity_data, Censor == 1) #get rid of mosquitoes censored out by experimental e
rror (get rid of 0 values; 1 = died naturally)

#calculate the total eggs laid per mosquito:

#need to subtract to find ones only laid on day 4 (exclude day 3 eggs)
Fecundity_data$Eggs_day4 <- (Fecundity_data$Eggs_day4)-(Fecundity_data$Eggs_day3)

#replace negative eggs day 4 values with zero (assume miscounted/eggs degraded and no new eggs laid)
for (row in 1:nrow(Fecundity_data)){
  if (is.na(Fecundity_data$Eggs_day4[row])){
    Fecundity_data$Eggs_day4[row] <- NA #keep NA values
  } else if ((Fecundity_data$Eggs_day4[row] <= 0)){
    Fecundity_data$Eggs_day4[row] <- 0
  }
}

#total eggs addition:
Fecundity_data$total_eggs <- (Fecundity_data$Eggs_day3)+(Fecundity_data$Eggs_day4)

#percents:
Fecundity_data$Percent_eggs_day3 <- (Fecundity_data$Eggs_day3) / (Fecundity_data$total_eggs)
Fecundity_data$Percent_eggs_day4 <- (Fecundity_data$Eggs_day4) / (Fecundity_data$total_eggs)

# decide if each mosquito laid eggs and on what day
#day 3
for (row in 1:nrow(Fecundity_data)){
  if(!is.na(Fecundity_data$Eggs_day3[row])){
    if (Fecundity_data$Eggs_day3[row] > 0){
      Fecundity_data$egg_binary[row] = 1
      Fecundity_data$egg_binary_day3[row] = 1
    }
    else {
      Fecundity_data$egg_binary[row] = 0
      Fecundity_data$egg_binary_day3[row] = 0
    }
  }
  else if (is.na(Fecundity_data$Eggs_day3[row])){
    Fecundity_data$egg_binary[row] = NA
    Fecundity_data$egg_binary_day3[row] = NA
  }
}

#day 4
for (row in 1:nrow(Fecundity_data)){
  if(!is.na(Fecundity_data$Eggs_day4[row])){
    if (Fecundity_data$Eggs_day4[row] >0){
      Fecundity_data$egg_binary[row] = 1
      Fecundity_data$egg_binary_day4[row] = 1
    }
    else{
      Fecundity_data$egg_binary_day4[row] = 0
    }
  }
  else if (is.na(Fecundity_data$Eggs_day4[row])){
    Fecundity_data$egg_binary_day4[row] = NA
```

```
  }
}

# decide if each mosquito had larvae
for (row in 1:nrow(Fecundity_data)){
  if(!is.na(Fecundity_data$Larvae_day4[row])){
    if (Fecundity_data$Larvae_day4[row] >0){
      Fecundity_data$larvae_binary[row] = 1
    }
    else{
      Fecundity_data$larvae_binary[row] = 0
    }
  }
  else{
    Fecundity_data$larvae_binary[row] = NA
  }
}

#percents:
Fecundity_data$Percent_eggshatchedtolarv <- (Fecundity_data$Larvae_day4) / (Fecundity_data$Eggs_day3)


#survival:
#calculate:
Fecundity_data$Days_to_death_post_BM <- Fecundity_data$Date_of_death - Fecundity_data$Trial_start_date
Fecundity_data$Age_of_death <- Fecundity_data$Age_of_BM + Fecundity_data$Days_to_death_post_BM

Fecundity_data <-
  Fecundity_data %>%
  mutate(
    Age_of_BM_days = as.difftime(Age_of_BM, unit="days")
  )
Fecundity_data$Date_of_eclosion <- Fecundity_data$Trial_start_date - (Fecundity_data$Age_of_BM_days)

library(lubridate)
Fecundity_data <-
  Fecundity_data %>%
  mutate(
    days_alive_post_BM = as.duration(Trial_start_date %--% Date_of_death) / ddays(1),
    days_alive_post_eclosion = as.duration(Date_of_eclosion %--% Date_of_death) / ddays(1),
  )

str(Fecundity_data)
```

```
## 'data.frame':	842 obs. of  33 variables:
##  $ ID_overall              : num  1 2 3 4 5 6 7 8 9 10 ...
##  $ Temperature             : Factor w/ 3 levels "27","30","32": 3 3 3 3 3 3 3 3 3 3 ...
##  $ Age                     : Factor w/ 4 levels "3","5","10","15": 1 1 1 1 1 1 1 1 1 1 ...
##  $ ID_per_group            : num  1 2 3 4 5 6 7 8 9 10 ...
##  $ Trial_start_date        : POSIXct, format: "2024-02-13" "2024-02-13" ...
##  $ Trial_number            : num  1 1 1 1 1 1 1 1 1 1 ...
##  $ BM1_Date                : POSIXct, format: "2024-02-13" "2024-02-13" ...
##  $ Age_of_BM               : num  3 3 3 3 3 3 3 3 3 3 ...
##  $ Bloodmeal_number        : Factor w/ 1 level "1": 1 1 1 1 1 1 1 1 1 1 ...
##  $ Oviposition_positive(y/n): chr  "Y" "Y" "Y" "Y" ...
##  $ Eggs_day3               : num  0 0 0 0 0 14 NA NA NA NA ...
##  $ Eggs_day4               : num  0 0 0 0 0 0 NA NA NA NA ...
##  $ Larvae_day4             : num  0 0 0 0 0 0 0 0 NA NA ...
##  $ Surv_to_eggs(y/n)       : Factor w/ 3 levels "N","NA","Y": 3 3 3 3 3 3 1 1 3 3 ...
##  $ Surv_to_larvae(y/n)     : Factor w/ 3 levels "N","NA","Y": 3 3 3 3 3 3 2 2 3 3 ...
##  $ Date_of_death           : POSIXct, format: "2024-03-01" "2024-03-07" ...
##  $ Censor                  : num  1 1 1 1 1 1 1 1 1 1 ...
##  $ Notes                   : chr  NA NA NA NA ...
##  $ Oviposition_positive    : Factor w/ 2 levels "N","Y": 2 2 2 2 2 2 2 2 1 1 ...
##  $ total_eggs              : num  0 0 0 0 0 14 NA NA NA NA ...
##  $ Percent_eggs_day3       : num  NaN NaN NaN NaN NaN 1 NA NA NA NA ...
##  $ Percent_eggs_day4       : num  NaN NaN NaN NaN NaN 0 NA NA NA NA ...
##  $ egg_binary              : num  0 0 0 0 0 1 NA NA NA NA ...
##  $ egg_binary_day3         : num  0 0 0 0 0 1 NA NA NA NA ...
##  $ egg_binary_day4         : num  0 0 0 0 0 0 NA NA NA NA ...
##  $ larvae_binary           : num  0 0 0 0 0 0 0 0 NA NA ...
##  $ Percent_eggshatchedtolarv: num  NaN NaN NaN NaN NaN 0 NA NA NA NA ...
##  $ Days_to_death_post_BM   : 'difftime' num  17 23 18 13 ...
##   ..- attr(*, "units")= chr "days"
##  $ Age_of_death            : 'difftime' num  20 26 21 16 ...
##   ..- attr(*, "units")= chr "days"
##  $ Age_of_BM_days          : 'difftime' num  3 3 3 3 ...
##   ..- attr(*, "units")= chr "days"
##  $ Date_of_eclosion        : POSIXct, format: "2024-02-10" "2024-02-10" ...
##  $ days_alive_post_BM      : num  17 23 18 13 15 18 3 3 12 11 ...
##  $ days_alive_post_eclosion : num  20 26 21 16 18 21 6 6 15 14 ...
```

```
Fecundity_data_firstBM <- subset(Fecundity_data, Bloodmeal_number == 1)

#subset by oviposition positive and negative:
Fecundity_data_ovipos <- subset(Fecundity_data, Oviposition_positive== "Y")
Fecundity_data_ovineg <- subset(Fecundity_data, Oviposition_positive== "N")
```

Calculate summary statistics:

```
####
#calculate summary stats:
Summary_Fecundity_data_ovipos <- Fecundity_data_ovipos %>%
  group_by(Temperature,Age,Oviposition_positive) %>%
  summarise(mean_eggsday3 = mean(Eggs_day3, na.rm = TRUE),
            mean_eggsday4 = mean(Eggs_day4, na.rm = TRUE),
            mean_totaleggs= mean(total_eggs, na.rm = TRUE),
            mean_percenteggs_day3 = mean(Percent_eggs_day3, na.rm = TRUE), #% of total eggs laid on day 3
            mean_percenteggs_day4 = mean(Percent_eggs_day4, na.rm = TRUE),#% of total eggs laid on day 4
            mean_larvae_day4 = mean(Larvae_day4, na.rm = TRUE),
            mean_Percent_eggshatchedtolarv = mean(Percent_eggshatchedtolarv, na.rm = TRUE), #of the day3 eg
gs, what percent hatched?
            #binary proportions:
            sum_egg_binary = sum(egg_binary, na.rm = TRUE), #gives total number of mosquitoes per group tha
t laid eggs
            sum_surv_to_day3 = sum(`Surv_to_eggs(y/n)`=="Y"), #gives total number of mosquitoes per group t
hat survived to day 3 post BM
            sum_surv_to_day4 = sum(`Surv_to_larvae(y/n)`=="Y"), #gives total number of mosquitoes per group
that survived to day 4 post BM
            sum_egg_binary_day3 = sum(egg_binary_day3, na.rm = TRUE), #number of eggs laid on day 3
            sum_egg_binary_day4 = sum(egg_binary_day4, na.rm = TRUE), #number of eggs laid on day 4
            sum_larvae_binary = sum(larvae_binary, na.rm = TRUE), #number of larvae counted on day 4
            proportion_binaryegg = (sum_egg_binary / n()), #what proportion laid any number of eggs? #remov
e NAs, but keep all mosquitoes bc some laid eggs even if dead on day 3 count
            proportion_binaryegg_day3 = (sum_egg_binary_day3 / sum_egg_binary), #what proportion laid eggs
on day 3, out of ones that laid eggs?
            proportion_binaryegg_day4 = (sum_egg_binary_day4 / sum_egg_binary), #what proportion laid eggs
on day 4, out of ones that laid eggs?
            proportion_binarylarvae = (sum_larvae_binary / sum_egg_binary_day3), #of ones that had eggs on
day 3, what proportion had any # of larvae hatch?
            #sample sizes
            n_mosquitoes = n(), #total n
            #standard errors
            SE_eggsday3 = sd(Eggs_day3,na.rm = TRUE)/sqrt(n()),
            SE_eggsday4 = sd(Eggs_day4,na.rm = TRUE)/sqrt(n()),
            SE_totaleggs = sd(total_eggs,na.rm = TRUE)/sqrt(n()),
            SE_percenteggs_day3 = sd(Percent_eggs_day3,na.rm = TRUE)/sqrt(n()),
            SE_percenteggs_day4 = sd(Percent_eggs_day4,na.rm = TRUE)/sqrt(n()),
            SE_larvae_day4 = sd(Larvae_day4,na.rm = TRUE)/sqrt(n()),
            SE_percenteggshatched = sd(Percent_eggshatchedtolarv,na.rm = TRUE)/sqrt(n()))
```

```
## `summarise()` has grouped output by 'Temperature', 'Age'. You can override
## using the `.groups` argument.
```

```
Summary_Fecundity_data_ovipos <- as.data.frame(Summary_Fecundity_data_ovipos)

str(Summary_Fecundity_data_ovipos)
```

```
## 'data.frame':    12 obs. of  28 variables:
##  $ Temperature                    : Factor w/ 3 levels "27","30","32": 1 1 1 1 2 2 2 2 3 3 ...
##  $ Age                            : Factor w/ 4 levels "3","5","10","15": 1 2 3 4 1 2 3 4 1 2 ...
##  $ Oviposition_positive           : Factor w/ 2 levels "N","Y": 2 2 2 2 2 2 2 2 2 2 ...
##  $ mean_eggsday3                  : num  17.21 42.09 26.64 5.43 13.56 ...
##  $ mean_eggsday4                  : num  2.05 2.13 2.22 3.15 2.62 ...
##  $ mean_totaleggs                 : num  19.8 43.8 30.6 10.6 20.8 ...
##  $ mean_percenteggs_day3          : num  0.919 0.886 0.918 0.649 0.859 ...
##  $ mean_percenteggs_day4          : num  0.0812 0.1143 0.0817 0.3514 0.1413 ...
##  $ mean_larvae_day4               : num  0.85 19.038 16.07 0.227 22 ...
##  $ mean_Percent_eggshatchedtolarv: num  0.0123 0.3888 0.4327 0.0142 0.3193 ...
##  $ sum_egg_binary                 : num  21 49 42 18 33 38 25 3 16 5 ...
##  $ sum_surv_to_day3               : int  58 52 50 25 71 68 33 19 40 34 ...
##  $ sum_surv_to_day4               : int  48 43 41 17 52 58 26 11 35 32 ...
##  $ sum_egg_binary_day3            : num  20 44 40 13 30 31 22 2 15 4 ...
##  $ sum_egg_binary_day4            : num  9 9 8 8 14 24 5 2 8 2 ...
##  $ sum_larvae_binary              : num  3 29 28 1 16 22 15 0 2 0 ...
##  $ proportion_binaryegg           : num  0.333 0.845 0.724 0.327 0.32 ...
##  $ proportion_binaryegg_day3      : num  0.952 0.898 0.952 0.722 0.909 ...
##  $ proportion_binaryegg_day4      : num  0.429 0.184 0.19 0.444 0.424 ...
##  $ proportion_binarylarvae        : num  0.15 0.6591 0.7 0.0769 0.5333 ...
##  $ n_mosquitoes                   : int  63 58 58 55 103 83 60 42 45 37 ...
##  $ SE_eggsday3                    : num  3.72 3.47 2.69 1.35 2.58 ...
##  $ SE_eggsday4                    : num  1.274 0.847 0.83 0.828 0.966 ...
##  $ SE_totaleggs                   : num  4.02 3.25 2.68 1.48 3.03 ...
##  $ SE_percenteggs_day3            : num  0.0276 0.0408 0.0309 0.0625 0.0292 ...
##  $ SE_percenteggs_day4            : num  0.0276 0.0408 0.0309 0.0625 0.0292 ...
##  $ SE_larvae_day4                 : num  0.309 2.859 2.113 0.144 2.733 ...
##  $ SE_percenteggshatched          : num  0.00406 0.04467 0.04737 0.00693 0.03642 ...
```

```
write_xlsx(Summary_Fecundity_data_ovipos, "Oviposition_binary/Summary_Fecundity_data_ovipos.xlsx")



Summary_Fecundity_data_ovipos_proportions <- Fecundity_data_ovipos %>% #Fecundity_data_firstBM_ovipos %>%
  group_by(Temperature,Age,Oviposition_positive,Trial_number) %>%
  summarise(#binary proportions:
    sum_egg_binary = sum(egg_binary, na.rm = TRUE), #gives total number of mosquitoes per group that laid e
ggs
    sum_surv_to_day3 = sum(`Surv_to_eggs(y/n)`=="Y"), #gives total number of mosquitoes per group that surv
ived to day 3 post BM
    sum_surv_to_day4 = sum(`Surv_to_larvae(y/n)`=="Y"), #gives total number of mosquitoes per group that su
rvived to day 4 post BM
    sum_egg_binary_day3 = sum(egg_binary_day3, na.rm = TRUE), #number of eggs laid on day 3
    sum_egg_binary_day4 = sum(egg_binary_day4, na.rm = TRUE), #number of eggs laid on day 4
    sum_larvae_binary = sum(larvae_binary, na.rm = TRUE), #number of larvae counted on day 4
    proportion_binaryegg = (sum_egg_binary / n()), #what proportion laid any number of eggs? #remove NAs, b
ut keep all mosquitoes bc some laid eggs even if dead on day 3 count
    proportion_binaryegg_day3 = (sum_egg_binary_day3 / sum_egg_binary), #what proportion laid eggs on day
3, out of ones that laid eggs?
    proportion_binaryegg_day4 = (sum_egg_binary_day4 / sum_egg_binary), #what proportion laid eggs on day
4, out of ones that laid eggs?
    proportion_binarylarvae = (sum_larvae_binary / sum_egg_binary_day3), #of ones that had eggs on day 3, w
hat proportion had any # of larvae hatch?
    proportion_surv_to_day3 = (sum_surv_to_day3 /n()),
    proportion_surv_to_day4 = (sum_surv_to_day4 /n()),
    #sample sizes
    n_mosquitoes = n()) #total n
```

```
## `summarise()` has grouped output by 'Temperature', 'Age',
## 'Oviposition_positive'. You can override using the `.groups` argument.
```

```
#standard errors

Summary_Fecundity_data_ovipos_proportions <- as.data.frame(Summary_Fecundity_data_ovipos_proportions)
write_xlsx(Summary_Fecundity_data_ovipos_proportions, "Oviposition_binary/Summary_Fecundity_data_ovipos_pro
portions.xlsx")

Summary_Fecundity_data_ovipos_proportions_means <- Summary_Fecundity_data_ovipos_proportions %>% #Fecundity
_data_firstBM_ovipos %>%
  group_by(Temperature,Age,Oviposition_positive) %>%
  summarise(#binary proportions:
    mean_proportion_binaryegg = mean(proportion_binaryegg,na.rm = TRUE), #what proportion laid any number o
f eggs? #remove NAs, but keep all mosquitoes bc some laid eggs even if dead on day 3 count
    mean_proportion_binaryegg_day3 = mean(proportion_binaryegg_day3,na.rm = TRUE), #what proportion laid eg
gs on day 3, out of ones that laid eggs?
    mean_proportion_binaryegg_day4 = mean(proportion_binaryegg_day4,na.rm = TRUE), #what proportion laid eg
gs on day 4, out of ones that laid eggs?
    mean_proportion_binarylarvae = mean(proportion_binarylarvae,na.rm = TRUE), #of ones that had eggs on da
y 3, what proportion had any # of larvae hatch?
    mean_proportion_surv_to_day3 = mean(proportion_surv_to_day3,na.rm = TRUE),
    mean_proportion_surv_to_day4 = mean(proportion_surv_to_day4,na.rm = TRUE),
    #sample sizes
    n_mosquitoes = sum(n_mosquitoes),
    n_trials = n(),
    SE_proportion_binaryegg = sd(proportion_binaryegg,na.rm = TRUE)/sqrt(n()),
    SE_proportion_binaryegg_day3 = sd(proportion_binaryegg_day3,na.rm = TRUE)/sqrt(n()),
    SE_proportion_binaryegg_day4 = sd(proportion_binaryegg_day4,na.rm = TRUE)/sqrt(n()),
    SE_proportion_binarylarvae = sd(proportion_binarylarvae,na.rm = TRUE)/sqrt(n()),
    SE_proportion_surv_to_day3 = sd(proportion_surv_to_day3,na.rm = TRUE)/sqrt(n()),
    SE_proportion_surv_to_day4 = sd(proportion_surv_to_day4,na.rm = TRUE)/sqrt(n()))
```

```
## `summarise()` has grouped output by 'Temperature', 'Age'. You can override
## using the `.groups` argument.
```

```
Summary_Fecundity_data_ovipos_proportions_means <- as.data.frame(Summary_Fecundity_data_ovipos_proportions_
means)
write_xlsx(Summary_Fecundity_data_ovipos_proportions_means, "Oviposition_binary/Summary_Fecundity_data_ovip
os_proportions_means.xlsx")


#TEMP only:
Summary_Fecundity_data_ovipos_TEMP <- Fecundity_data_ovipos %>%
  group_by(Temperature) %>%
  summarise(mean_eggsday3 = mean(Eggs_day3, na.rm = TRUE),
            mean_eggsday4 = mean(Eggs_day4, na.rm = TRUE),
            mean_totaleggs= mean(total_eggs, na.rm = TRUE),
            mean_percenteggs_day3 = mean(Percent_eggs_day3, na.rm = TRUE), #% of total eggs laid on day 3
            mean_percenteggs_day4 = mean(Percent_eggs_day4, na.rm = TRUE),#% of total eggs laid on day 4
            mean_larvae_day4 = mean(Larvae_day4, na.rm = TRUE),
            mean_Percent_eggshatchedtolarv = mean(Percent_eggshatchedtolarv, na.rm = TRUE), #of the day3 eg
gs, what percent hatched?
            #binary proportions:
            sum_egg_binary = sum(egg_binary, na.rm = TRUE), #gives total number of mosquitoes per group tha
t laid eggs
            sum_surv_to_day3 = sum(`Surv_to_eggs(y/n)`=="Y"), #gives total number of mosquitoes per group t
hat survived to day 3 post BM
            sum_surv_to_day4 = sum(`Surv_to_larvae(y/n)`=="Y"), #gives total number of mosquitoes per group
that survived to day 4 post BM
            sum_egg_binary_day3 = sum(egg_binary_day3, na.rm = TRUE), #number of eggs laid on day 3
            sum_egg_binary_day4 = sum(egg_binary_day4, na.rm = TRUE), #number of eggs laid on day 4
            sum_larvae_binary = sum(larvae_binary, na.rm = TRUE), #number of larvae counted on day 4
            proportion_binaryegg = (sum_egg_binary / n()), #what proportion laid any number of eggs? #remov
e NAs, but keep all mosquitoes bc some laid eggs even if dead on day 3 count
            proportion_binaryegg_day3 = (sum_egg_binary_day3 / sum_egg_binary), #what proportion laid eggs
on day 3, out of ones that laid eggs?
            proportion_binaryegg_day4 = (sum_egg_binary_day4 / sum_egg_binary), #what proportion laid eggs
on day 4, out of ones that laid eggs?
            proportion_binarylarvae = (sum_larvae_binary / sum_egg_binary_day3), #of ones that had eggs on
day 3, what proportion had any # of larvae hatch?
            #sample sizes
            n_mosquitoes = n(), #total n
            #standard errors
            SE_eggsday3 = sd(Eggs_day3,na.rm = TRUE)/sqrt(n()),
            SE_eggsday4 = sd(Eggs_day4,na.rm = TRUE)/sqrt(n()),
            SE_totaleggs = sd(total_eggs,na.rm = TRUE)/sqrt(n()),
            SE_percenteggs_day3 = sd(Percent_eggs_day3,na.rm = TRUE)/sqrt(n()),
            SE_percenteggs_day4 = sd(Percent_eggs_day4,na.rm = TRUE)/sqrt(n()),
            SE_larvae_day4 = sd(Larvae_day4,na.rm = TRUE)/sqrt(n()),
            SE_percenteggshatched = sd(Percent_eggshatchedtolarv,na.rm = TRUE)/sqrt(n()))

Summary_Fecundity_data_ovipos_TEMP <- as.data.frame(Summary_Fecundity_data_ovipos_TEMP)
write_xlsx(Summary_Fecundity_data_ovipos_TEMP, "Oviposition_binary/Summary_Fecundity_data_ovipos_TEMP.xls
x")

Summary_Fecundity_data_ovipos_proportions_means_TEMP <- Summary_Fecundity_data_ovipos_proportions %>% #Fecu
ndity_data_firstBM_ovipos %>%
  group_by(Temperature) %>%
  summarise(#binary proportions:
    mean_proportion_binaryegg = mean(proportion_binaryegg,na.rm = TRUE), #what proportion laid any number o
f eggs? #remove NAs, but keep all mosquitoes bc some laid eggs even if dead on day 3 count
```

```
    mean_proportion_binaryegg_day3 = mean(proportion_binaryegg_day3,na.rm = TRUE), #what proportion laid eg
gs on day 3, out of ones that laid eggs?
    mean_proportion_binaryegg_day4 = mean(proportion_binaryegg_day4,na.rm = TRUE), #what proportion laid eg
gs on day 4, out of ones that laid eggs?
    mean_proportion_binarylarvae = mean(proportion_binarylarvae,na.rm = TRUE), #of ones that had eggs on da
y 3, what proportion had any # of larvae hatch?
    mean_proportion_surv_to_day3 = mean(proportion_surv_to_day3,na.rm = TRUE),
    mean_proportion_surv_to_day4 = mean(proportion_surv_to_day4,na.rm = TRUE),
    #sample sizes
    n_mosquitoes = sum(n_mosquitoes),
    n_trials = n(),
    SE_proportion_binaryegg = sd(proportion_binaryegg,na.rm = TRUE)/sqrt(n()),
    SE_proportion_binaryegg_day3 = sd(proportion_binaryegg_day3,na.rm = TRUE)/sqrt(n()),
    SE_proportion_binaryegg_day4 = sd(proportion_binaryegg_day4,na.rm = TRUE)/sqrt(n()),
    SE_proportion_binarylarvae = sd(proportion_binarylarvae,na.rm = TRUE)/sqrt(n()),
    SE_proportion_surv_to_day3 = sd(proportion_surv_to_day3,na.rm = TRUE)/sqrt(n()),
    SE_proportion_surv_to_day4 = sd(proportion_surv_to_day4,na.rm = TRUE)/sqrt(n()))


Summary_Fecundity_data_ovipos_proportions_means_TEMP <- as.data.frame(Summary_Fecundity_data_ovipos_proport
ions_means_TEMP)
write_xlsx(Summary_Fecundity_data_ovipos_proportions_means_TEMP, "Oviposition_binary/Summary_Fecundity_data
_ovipos_proportions_means_TEMP.xlsx")


#AGE only:
Summary_Fecundity_data_ovipos_AGE <- Fecundity_data_ovipos %>%
  group_by(Age) %>%
  summarise(mean_eggsday3 = mean(Eggs_day3, na.rm = TRUE),
            mean_eggsday4 = mean(Eggs_day4, na.rm = TRUE),
            mean_totaleggs= mean(total_eggs, na.rm = TRUE),
            mean_percenteggs_day3 = mean(Percent_eggs_day3, na.rm = TRUE), #% of total eggs laid on day 3
            mean_percenteggs_day4 = mean(Percent_eggs_day4, na.rm = TRUE),#% of total eggs laid on day 4
            mean_larvae_day4 = mean(Larvae_day4, na.rm = TRUE),
            mean_Percent_eggshatchedtolarv = mean(Percent_eggshatchedtolarv, na.rm = TRUE), #of the day3 eg
gs, what percent hatched?
            #binary proportions:
            sum_egg_binary = sum(egg_binary, na.rm = TRUE), #gives total number of mosquitoes per group tha
t laid eggs
            sum_surv_to_day3 = sum(`Surv_to_eggs(y/n)`=="Y"), #gives total number of mosquitoes per group t
hat survived to day 3 post BM
            sum_surv_to_day4 = sum(`Surv_to_larvae(y/n)`=="Y"), #gives total number of mosquitoes per group
that survived to day 4 post BM
            sum_egg_binary_day3 = sum(egg_binary_day3, na.rm = TRUE), #number of eggs laid on day 3
            sum_egg_binary_day4 = sum(egg_binary_day4, na.rm = TRUE), #number of eggs laid on day 4
            sum_larvae_binary = sum(larvae_binary, na.rm = TRUE), #number of larvae counted on day 4
            proportion_binaryegg = (sum_egg_binary / n()), #what proportion laid any number of eggs? #remov
e NAs, but keep all mosquitoes bc some laid eggs even if dead on day 3 count
            proportion_binaryegg_day3 = (sum_egg_binary_day3 / sum_egg_binary), #what proportion laid eggs
on day 3, out of ones that laid eggs?
            proportion_binaryegg_day4 = (sum_egg_binary_day4 / sum_egg_binary), #what proportion laid eggs
on day 4, out of ones that laid eggs?
            proportion_binarylarvae = (sum_larvae_binary / sum_egg_binary_day3), #of ones that had eggs on
day 3, what proportion had any # of larvae hatch?
            #sample sizes
            n_mosquitoes = n(), #total n
```

```
            #standard errors
            SE_eggsday3 = sd(Eggs_day3,na.rm = TRUE)/sqrt(n()),
            SE_eggsday4 = sd(Eggs_day4,na.rm = TRUE)/sqrt(n()),
            SE_totaleggs = sd(total_eggs,na.rm = TRUE)/sqrt(n()),
            SE_percenteggs_day3 = sd(Percent_eggs_day3,na.rm = TRUE)/sqrt(n()),
            SE_percenteggs_day4 = sd(Percent_eggs_day4,na.rm = TRUE)/sqrt(n()),
            SE_larvae_day4 = sd(Larvae_day4,na.rm = TRUE)/sqrt(n()),
            SE_percenteggshatched = sd(Percent_eggshatchedtolarv,na.rm = TRUE)/sqrt(n()))

Summary_Fecundity_data_ovipos_AGE <- as.data.frame(Summary_Fecundity_data_ovipos_AGE)
write_xlsx(Summary_Fecundity_data_ovipos_AGE, "Oviposition_binary/Summary_Fecundity_data_ovipos_AGE.xlsx")


Summary_Fecundity_data_ovipos_proportions_means_AGE <- Summary_Fecundity_data_ovipos_proportions %>% #Fecundity_data_firstBM_ovipos %>%
  group_by(Age) %>%
  summarise(#binary proportions:
    mean_proportion_binaryegg = mean(proportion_binaryegg,na.rm = TRUE), #what proportion laid any number of eggs? #remove NAs, but keep all mosquitoes bc some laid eggs even if dead on day 3 count
    mean_proportion_binaryegg_day3 = mean(proportion_binaryegg_day3,na.rm = TRUE), #what proportion laid eggs on day 3, out of ones that laid eggs?
    mean_proportion_binaryegg_day4 = mean(proportion_binaryegg_day4,na.rm = TRUE), #what proportion laid eggs on day 4, out of ones that laid eggs?
    mean_proportion_binarylarvae = mean(proportion_binarylarvae,na.rm = TRUE), #of ones that had eggs on day 3, what proportion had any # of larvae hatch?
    mean_proportion_surv_to_day3 = mean(proportion_surv_to_day3,na.rm = TRUE),
    mean_proportion_surv_to_day4 = mean(proportion_surv_to_day4,na.rm = TRUE),
    #sample sizes
    n_mosquitoes = sum(n_mosquitoes),
    n_trials = n(),
    SE_proportion_binaryegg = sd(proportion_binaryegg,na.rm = TRUE)/sqrt(n()),
    SE_proportion_binaryegg_day3 = sd(proportion_binaryegg_day3,na.rm = TRUE)/sqrt(n()),
    SE_proportion_binaryegg_day4 = sd(proportion_binaryegg_day4,na.rm = TRUE)/sqrt(n()),
    SE_proportion_binarylarvae = sd(proportion_binarylarvae,na.rm = TRUE)/sqrt(n()),
    SE_proportion_surv_to_day3 = sd(proportion_surv_to_day3,na.rm = TRUE)/sqrt(n()),
    SE_proportion_surv_to_day4 = sd(proportion_surv_to_day4,na.rm = TRUE)/sqrt(n()))


Summary_Fecundity_data_ovipos_proportions_means_AGE <- as.data.frame(Summary_Fecundity_data_ovipos_proportions_means_AGE)
write_xlsx(Summary_Fecundity_data_ovipos_proportions_means_AGE, "Oviposition_binary/Summary_Fecundity_data_ovipos_proportions_means_AGE.xlsx")



#################################
```

Plot oviposition success data

```
#basic plots:
Fecundity_data_firstBM_ovipos <- Fecundity_data_ovipos
Summary_Fecundity_data_firstBM_ovipos <- Summary_Fecundity_data_ovipos
Summary_Fecundity_data_firstBM_ovipos_proportions <- Summary_Fecundity_data_ovipos_proportions
Summary_Fecundity_data_firstBM_ovipos_proportions_means <- Summary_Fecundity_data_ovipos_proportions_means
Summary_Fecundity_data_firstBM_ovipos_AGE <- Summary_Fecundity_data_ovipos_AGE
Summary_Fecundity_data_firstBM_ovipos_proportions_means_AGE <- Summary_Fecundity_data_ovipos_proportions_me
ans_AGE
Summary_Fecundity_data_firstBM_ovipos_TEMP <- Summary_Fecundity_data_ovipos_TEMP
Summary_Fecundity_data_firstBM_ovipos_proportions_means_TEMP <- Summary_Fecundity_data_ovipos_proportions_m
eans_TEMP

# what proportion laid any number of eggs (binary)?
Summary_Fecundity_data_firstBM_ovipos_proportions$Age <- factor(Summary_Fecundity_data_firstBM_ovipos_propo
rtions$Age,
                                                                labels = c("3 days","5 days","10 days","15
days"))

Summary_Fecundity_data_firstBM_ovipos_proportions$Temperature <- factor(Summary_Fecundity_data_firstBM_ovip
os_proportions$Temperature,
                                                                        labels = c("27","30","32"))

Summary_Fecundity_data_firstBM_ovipos_proportions_means$Age <- factor(Summary_Fecundity_data_firstBM_ovipos
_proportions_means$Age,
                                                                      labels = c("3 days","5 days","10 day
s","15 days"))

Summary_Fecundity_data_firstBM_ovipos_proportions_means$Temperature <- factor(Summary_Fecundity_data_firstB
M_ovipos_proportions_means$Temperature,
                                                                              labels = c("27","30","32"))



BM1_ovipos_proportionbinaryegg_tempwithinage <- Summary_Fecundity_data_firstBM_ovipos_proportions_means %>%
  ggplot(aes(x=Temperature,y=mean_proportion_binaryegg,group=Temperature))+
  geom_bar(aes(fill=Temperature),
           stat = "identity",
           position = position_dodge(1),
           width = 0.8) +
  scale_shape_identity(guide="legend")+
  facet_grid(~Age)+
  geom_errorbar(aes(ymin=(mean_proportion_binaryegg - SE_proportion_binaryegg),
                    ymax=(mean_proportion_binaryegg + SE_proportion_binaryegg)),
                width=0.8,position=position_dodge(0.9),
                color="black")+
  ylab(expression("% of mosquitoes that laid any eggs"))+
  xlab("Temperature (˚C)") +
  theme_pubr()+
  theme(legend.position = "none")+
  scale_y_continuous(labels = function(x) paste0(x*100),limits=c(0,1)) +
  geom_jitter(data=Summary_Fecundity_data_firstBM_ovipos_proportions, aes(x=Temperature,y=proportion_binary
egg),#color=ZOI_italic$Technical_Rep,
              position = "jitter", na.rm = TRUE,size=0.5)+
  scale_fill_manual(values= c("#4D6FAE","#6F9F51", "#CC763B"))+
  theme(panel.background = element_rect(fill = NA, color = "black"))+
```

```
  theme(panel.spacing = unit(0.5, "lines"))
BM1_ovipos_proportionbinaryegg_tempwithinage
```

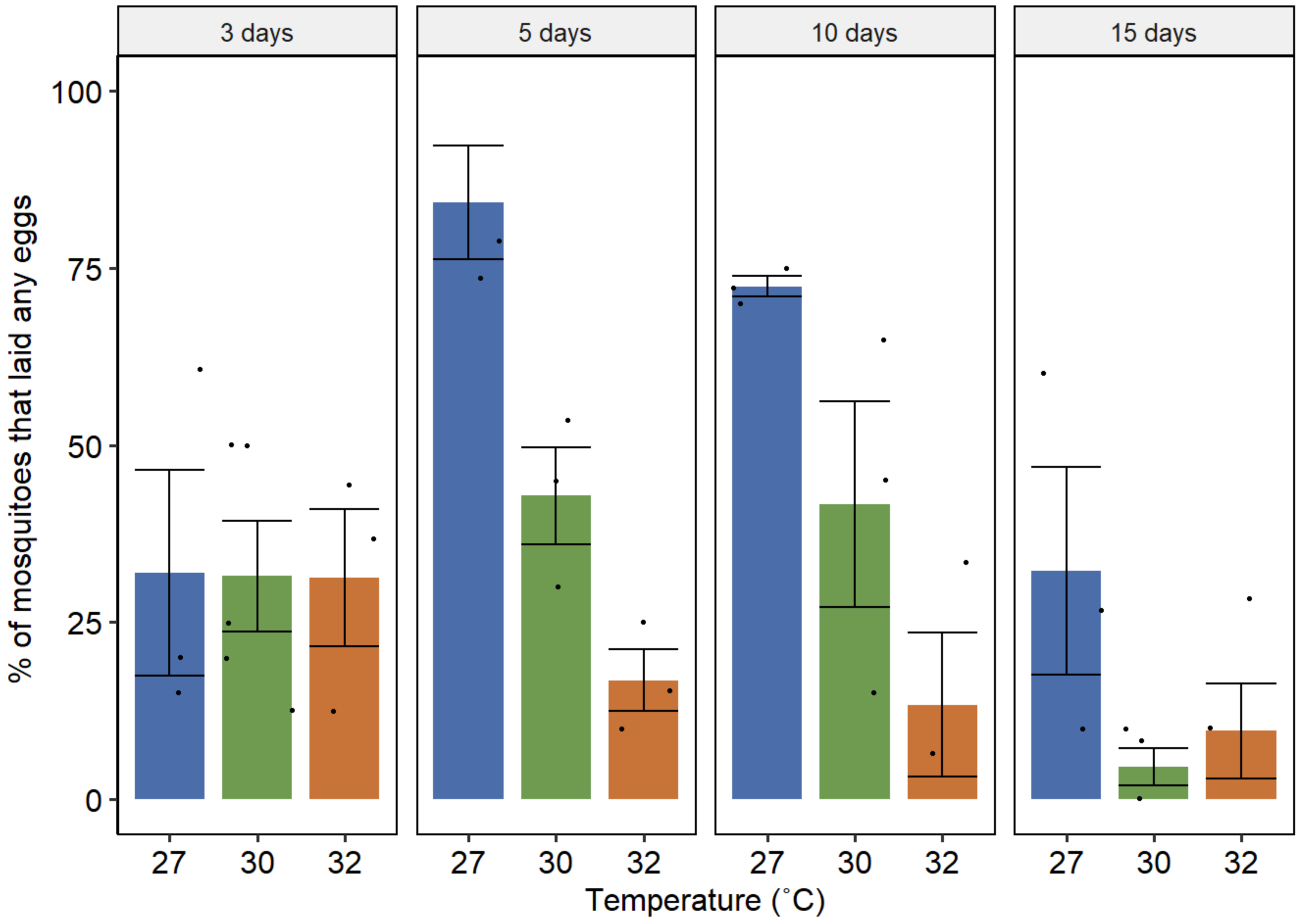

```
ggsave("Oviposition_binary/BM1_ovipos_proportionbinaryegg_tempwithinage.pdf", plot=BM1_ovipos_proportionbin
aryegg_tempwithinage, width = 6, height = 4,
       units = "in",dpi=600)
ggsave("Oviposition_binary/BM1_ovipos_proportionbinaryegg_tempwithinage.png", plot=BM1_ovipos_proportionbin
aryegg_tempwithinage, width = 6, height = 4,
       units = "in",dpi=600)



Summary_Fecundity_data_firstBM_ovipos_proportions_means$Age <- factor(Summary_Fecundity_data_firstBM_ovipos
_proportions_means$Age,
                                                                      labels = c("3","5","10","15"))
Summary_Fecundity_data_firstBM_ovipos_proportions_means$Temperature <- factor(Summary_Fecundity_data_firstB
M_ovipos_proportions_means$Temperature,
                                                                              labels = c("27˚C","30˚C","32˚
C"))

Summary_Fecundity_data_firstBM_ovipos_proportions$Age <- factor(Summary_Fecundity_data_firstBM_ovipos_propo
rtions$Age,
                                                                labels = c("3","5","10","15"))
Summary_Fecundity_data_firstBM_ovipos_proportions$Temperature <- factor(Summary_Fecundity_data_firstBM_ovip
os_proportions$Temperature,
                                                                        labels = c("27˚C","30˚C","32˚C"))

BM1_ovipos_proportionbinaryegg_agewithintemp <- Summary_Fecundity_data_firstBM_ovipos_proportions_means%>%
  ggplot(aes(x=Age,y=mean_proportion_binaryegg,group=Age))+
  geom_bar(aes(fill=Age),
           stat = "identity",
           position = position_dodge(1),
           width = 0.8) +
  scale_shape_identity(guide="legend")+
  facet_grid(~Temperature)+
  geom_errorbar(aes(ymin=(mean_proportion_binaryegg - SE_proportion_binaryegg),
                    ymax=(mean_proportion_binaryegg + SE_proportion_binaryegg)),
                width=0.8,position=position_dodge(0.9),
                color="black")+
  ylab(expression("% of mosquitoes that laid any eggs"))+
  scale_y_continuous(labels = function(x) paste0(x*100),limits=c(0,1)) +
  xlab("Age of first BM (days old)") +
  theme_pubr()+
  theme(legend.position = "none")+
  geom_jitter(data=Summary_Fecundity_data_firstBM_ovipos_proportions, aes(x=Age,y=proportion_binaryegg),#co
lor=ZOI_italic$Technical_Rep,
              position = "jitter", na.rm = TRUE,size=0.5)+
  scale_fill_manual(values= c("#DCD1E9","#BAA4D3","#9776BE","#7549A8"))+
  theme(panel.background = element_rect(fill = NA, color = "black"))+
  theme(panel.spacing = unit(0.5, "lines"))
BM1_ovipos_proportionbinaryegg_agewithintemp
```

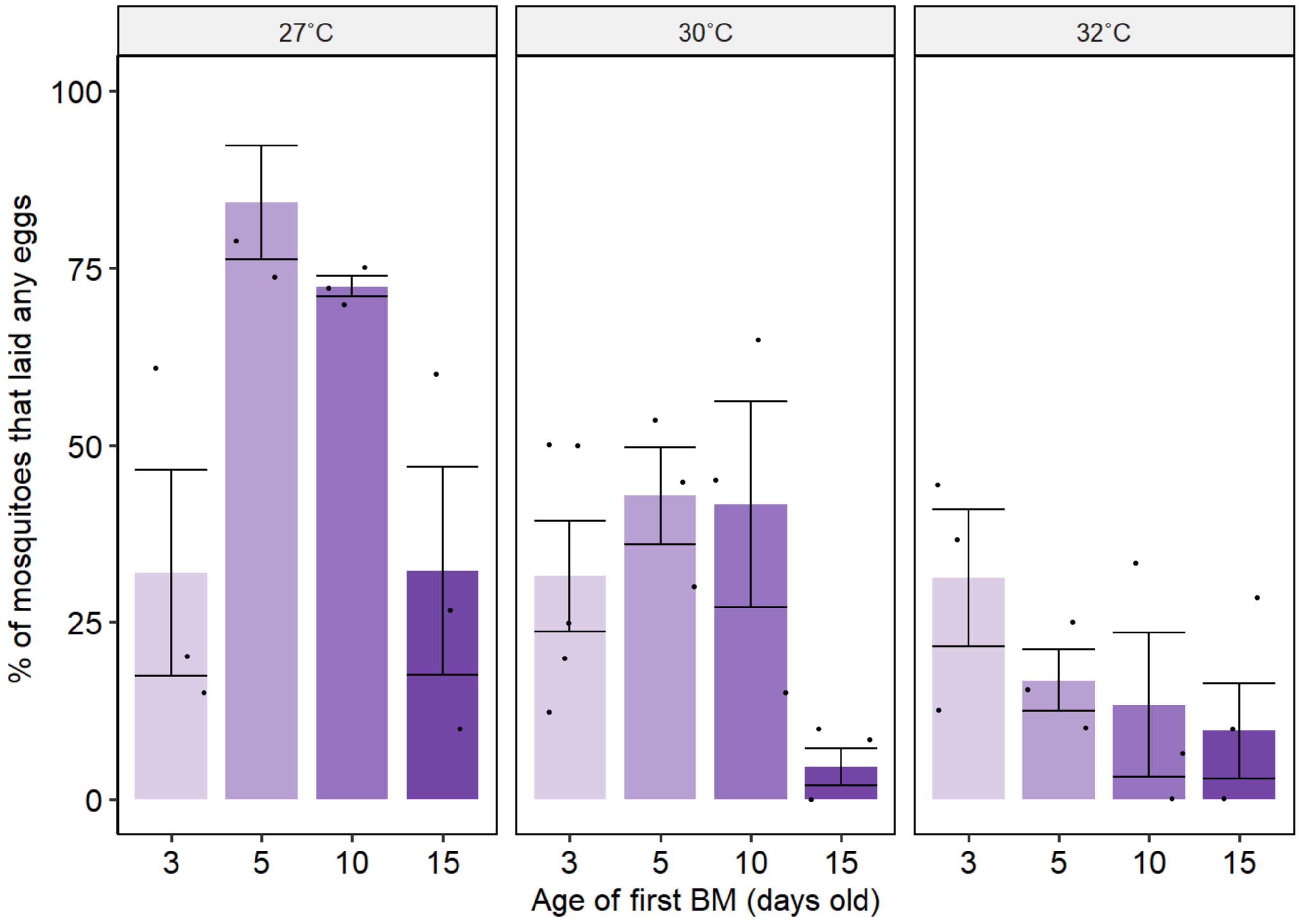


```
ggsave("Oviposition_binary/BM1_ovipos_proportionbinaryegg_agewithintemp.pdf", plot=BM1_ovipos_proportionbin
aryegg_agewithintemp, width = 6, height = 4,
       units = "in",dpi=600)
ggsave("Oviposition_binary/BM1_ovipos_proportionbinaryegg_agewithintemp.png", plot=BM1_ovipos_proportionbin
aryegg_agewithintemp, width = 6, height = 4,
       units = "in",dpi=600)

#raw data interaction plot:
BM1_ovipos_proportionbinaryegg_rawdata_interaction <- Summary_Fecundity_data_firstBM_ovipos_proportions_mea
ns%>%
  ggplot()+
  aes(x=Age,y=mean_proportion_binaryegg,group=Temperature)+
  geom_point(aes(shape=Temperature),size=3)+
  geom_line(aes(linetype = Temperature),linewidth=0.6)+
  theme_pubr()+
  scale_shape(labels=c(27,30,32))+
  scale_linetype(labels=c(27,30,32))+
  guides(shape = guide_legend(title = "Temperature (˚C)"),
         linetype = guide_legend(title = "Temperature (˚C)"))+
  xlab("Adult Age (days)") +
  theme(legend.position = "right")+
  scale_x_discrete(labels=c(3,5,10,15))+
  ylab(expression("% of mosquitoes that laid any eggs"))+
  theme(panel.background = element_rect(fill = NA, color = "black"))+
  theme(panel.spacing = unit(0.6, "lines"))
BM1_ovipos_proportionbinaryegg_rawdata_interaction
```

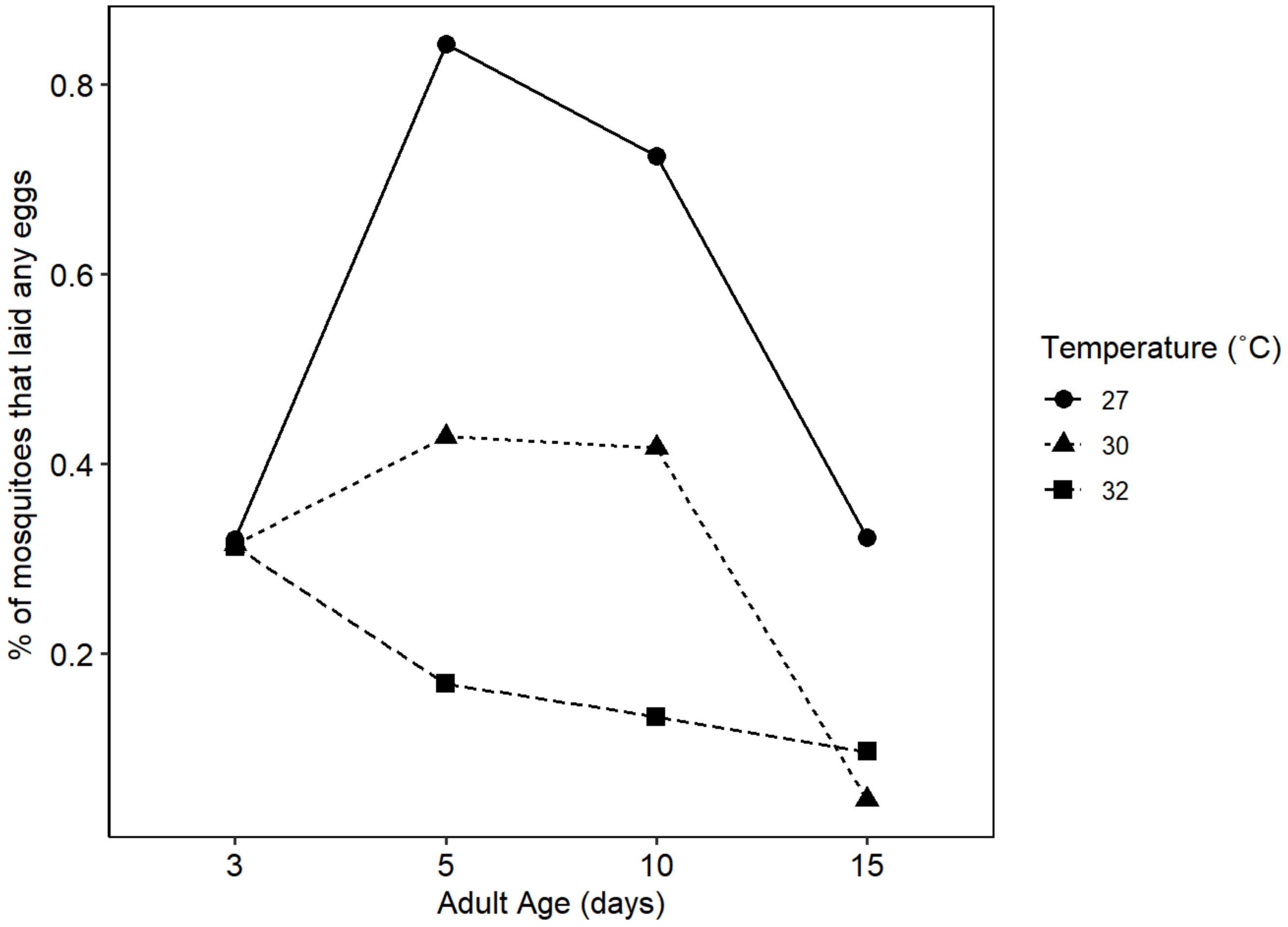

0.8
0.6
0.4
0.2
% of mosquitoes that laid any eggs
3
5
10
15
Adult Age (days)
Temperature (˚C)
27
30
32

```
ggsave("Oviposition_binary/BM1_ovipos_proportionbinaryegg_rawdata_interaction.png",plot=BM1_ovipos_proporti
onbinaryegg_rawdata_interaction,width = 5.5, height = 4, units = "in", dpi = 600)
ggsave("Oviposition_binary/BM1_ovipos_proportionbinaryegg_rawdata_interaction.pdf",plot=BM1_ovipos_proporti
onbinaryegg_rawdata_interaction,width = 5.5, height = 4, units = "in", dpi = 600)



#TEMP ONLY
Summary_Fecundity_data_firstBM_ovipos_proportions_means_TEMP$Temperature <- factor(Summary_Fecundity_data_f
irstBM_ovipos_proportions_means_TEMP$Temperature,
                                                                                  labels = c("27","30","3
2"))
Summary_Fecundity_data_firstBM_ovipos_proportions$Temperature <- factor(Summary_Fecundity_data_firstBM_ovip
os_proportions$Temperature,
                                                                       labels = c("27","30","32"))

BM1_ovipos_proportionbinaryegg_TEMP <- Summary_Fecundity_data_firstBM_ovipos_proportions_means_TEMP %>%
  ggplot(aes(x=Temperature,y=mean_proportion_binaryegg,group=Temperature))+
  geom_bar(aes(fill=Temperature),
           stat = "identity",
           position = position_dodge(1),
           width = 0.8) +
  scale_shape_identity(guide="legend")+
  geom_errorbar(aes(ymin=(mean_proportion_binaryegg - SE_proportion_binaryegg),
                    ymax=(mean_proportion_binaryegg + SE_proportion_binaryegg)),
                width=0.8,position=position_dodge(0.9),
                color="black")+
  ylab(expression("% of mosquitoes that laid any eggs"))+
  scale_y_continuous(labels = function(x) paste0(x*100),limits=c(0,1)) +
  xlab("Temperature (˚C)") +
  theme_pubr()+
  theme(legend.position = "none")+
  geom_jitter(data=Summary_Fecundity_data_firstBM_ovipos_proportions, aes(x=Temperature,y=proportion_binary
egg),#color=ZOI_italic$Technical_Rep,
              position = "jitter", na.rm = TRUE,size=0.5)+
  scale_fill_manual(values= c("#4D6FAE","#6F9F51", "#CC763B"))+
  theme(panel.background = element_rect(fill = NA, color = "black"))+
  theme(panel.spacing = unit(0.5, "lines"))
BM1_ovipos_proportionbinaryegg_TEMP
```

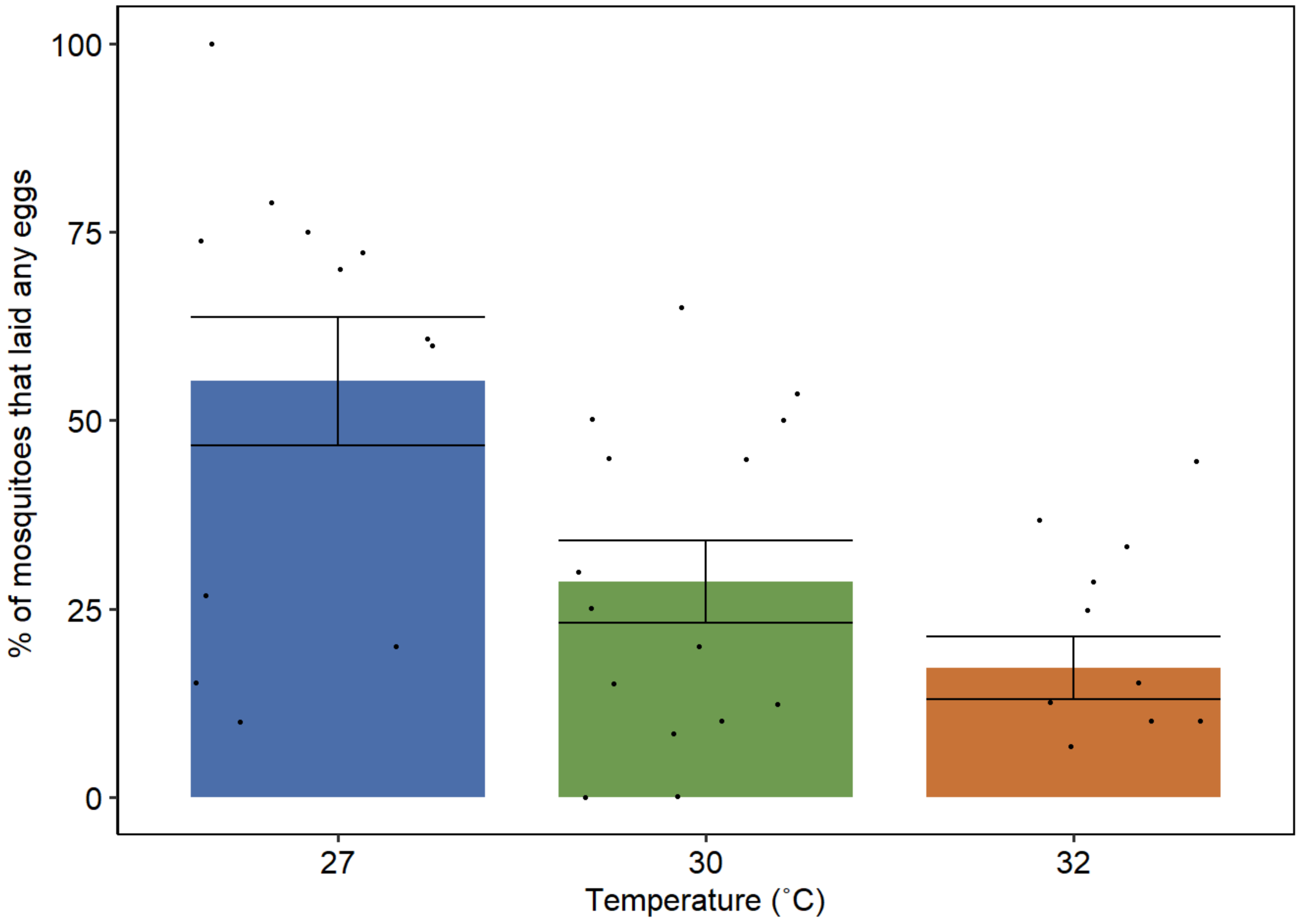

% of mosquitoes that laid any eggs
100
75
50
25
0
27
30
32
Temperature (˚C)

```
ggsave("Oviposition_binary/BM1_ovipos_proportionbinaryegg_TEMP.pdf", plot=BM1_ovipos_proportionbinaryegg_TE
MP, width = 4, height = 4,
       units = "in",dpi=600)
ggsave("Oviposition_binary/BM1_ovipos_proportionbinaryegg_TEMP.png", plot=BM1_ovipos_proportionbinaryegg_TE
MP, width =4, height = 4,
       units = "in",dpi=600)



#AGE ONLY
Summary_Fecundity_data_firstBM_ovipos_proportions_means_AGE$Age <- factor(Summary_Fecundity_data_firstBM_ov
ipos_proportions_means_AGE$Age,
                                                                          labels = c("3","5","10","15"))
Summary_Fecundity_data_firstBM_ovipos_proportions$Age <- factor(Summary_Fecundity_data_firstBM_ovipos_propo
rtions$Age,
                                                                labels = c("3","5","10","15"))

BM1_ovipos_proportionbinaryegg_AGE <- Summary_Fecundity_data_firstBM_ovipos_proportions_means_AGE %>%
  ggplot(aes(x=Age,y=mean_proportion_binaryegg,group=Age))+
  geom_bar(aes(fill=Age),
           stat = "identity",
           position = position_dodge(1),
           width = 0.8) +
  scale_shape_identity(guide="legend")+
  geom_errorbar(aes(ymin=(mean_proportion_binaryegg - SE_proportion_binaryegg),
                    ymax=(mean_proportion_binaryegg + SE_proportion_binaryegg)),
                width=0.8,position=position_dodge(0.9),
                color="black")+
  ylab(expression("% of mosquitoes that laid any eggs"))+
  scale_y_continuous(labels = function(x) paste0(x*100),limits=c(0,1)) +
  xlab("Age of first BM (days old)") +
  theme_pubr()+
  theme(legend.position = "none")+
  geom_jitter(data=Summary_Fecundity_data_firstBM_ovipos_proportions, aes(x=Age,y=proportion_binaryegg),#co
lor=ZOI_italic$Technical_Rep,
              position = "jitter", na.rm = TRUE,size=0.5)+
  scale_fill_manual(values= c("#DCD1E9","#BAA4D3","#9776BE","#7549A8"))+
  theme(panel.background = element_rect(fill = NA, color = "black"))+
  theme(panel.spacing = unit(0.5, "lines"))
BM1_ovipos_proportionbinaryegg_AGE
```

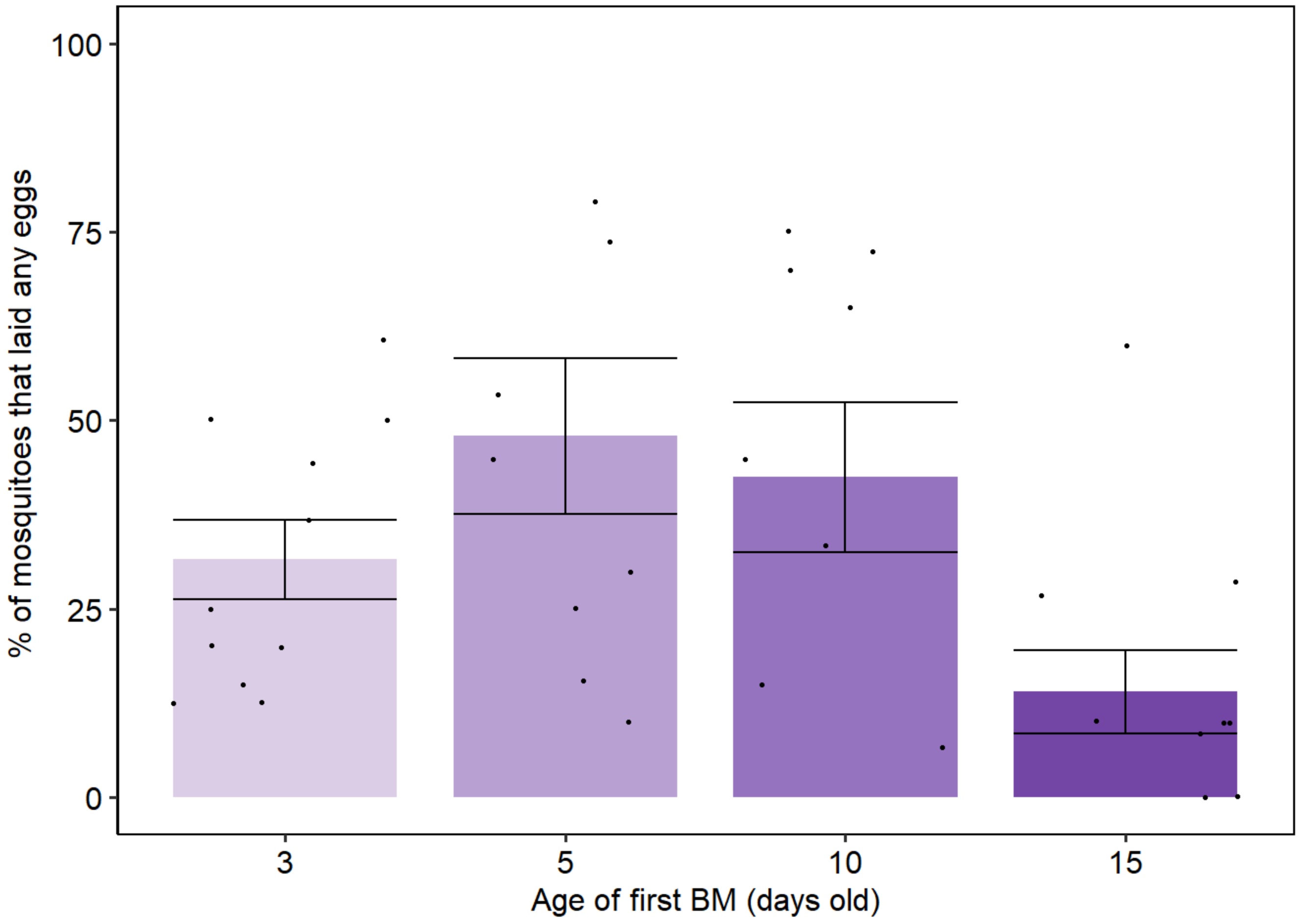


```
ggsave("Oviposition_binary/BM1_ovipos_proportionbinaryegg_AGE.pdf", plot=BM1_ovipos_proportionbinaryegg_AG
E, width = 4, height = 4,
       units = "in",dpi=600)
ggsave("Oviposition_binary/BM1_ovipos_proportionbinaryegg_AGE.png", plot=BM1_ovipos_proportionbinaryegg_AG
E, width = 4, height = 4,
       units = "in",dpi=600)
```

Analysis for oviposition success data

```
###############################
#Analysis
str(Summary_Fecundity_data_firstBM_ovipos_proportions)
```

```
## 'data.frame':    40 obs. of  17 variables:
##  $ Temperature               : Factor w/ 3 levels "27","30","32": 1 1 1 1 1 1 1 1 1 1 ...
##  $ Age                       : Factor w/ 4 levels "3","5","10","15": 1 1 1 2 2 2 3 3 3 4 ...
##  $ Oviposition_positive      : Factor w/ 2 levels "N","Y": 2 2 2 2 2 2 2 2 2 2 ...
##  $ Trial_number              : num  1 2 5 1 2 4 1 2 3 1 ...
##  $ sum_egg_binary            : num  3 4 14 14 15 20 13 15 14 12 ...
##  $ sum_surv_to_day3          : int  17 18 23 17 17 18 15 17 18 14 ...
##  $ sum_surv_to_day4          : int  15 13 20 14 12 17 8 15 18 9 ...
##  $ sum_egg_binary_day3       : num  3 4 13 12 14 18 12 15 13 8 ...
##  $ sum_egg_binary_day4       : num  2 0 7 3 4 2 5 1 2 6 ...
##  $ sum_larvae_binary         : num  0 0 3 9 9 11 4 12 12 0 ...
##  $ proportion_binaryegg      : num  0.15 0.2 0.609 0.737 0.789 ...
##  $ proportion_binaryegg_day3: num  1 1 0.929 0.857 0.933 ...
##  $ proportion_binaryegg_day4: num  0.667 0 0.5 0.214 0.267 ...
##  $ proportion_binarylarvae   : num  0 0 0.231 0.75 0.643 ...
##  $ proportion_surv_to_day3   : num  0.85 0.9 1 0.895 0.895 ...
##  $ proportion_surv_to_day4   : num  0.75 0.65 0.87 0.737 0.632 ...
##  $ n_mosquitoes              : int  20 20 23 19 19 20 18 20 20 20 ...
```

```
hist(Summary_Fecundity_data_firstBM_ovipos_proportions$proportion_binaryegg)
```

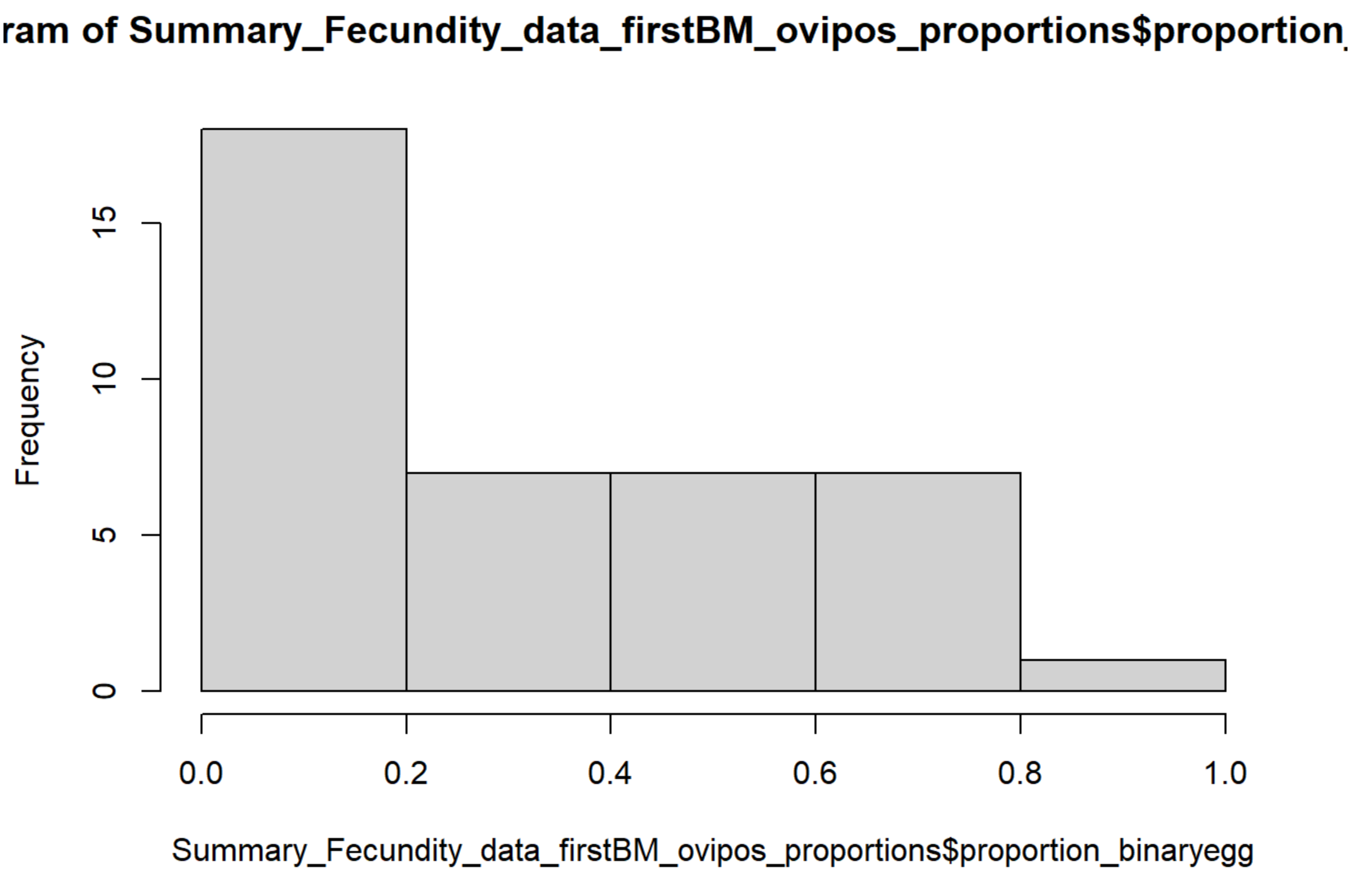


```
shapiro.test(Summary_Fecundity_data_firstBM_ovipos_proportions$proportion_binaryegg)
```

```
##
##  Shapiro-Wilk normality test
##
## data:  Summary_Fecundity_data_firstBM_ovipos_proportions$proportion_binaryegg
## W = 0.92188, p-value = 0.008826
```

```
#non-normal
unique(Fecundity_data_firstBM_ovipos$egg_binary) #NA did not survive. remove.
```

```
## [1]  0  1 NA
```

```
Fecundity_data_firstBM_ovipos_narm <- subset(Fecundity_data_firstBM_ovipos, egg_binary == 0 | egg_binary==
1)

#model fitting:
library(glmmTMB)
model.Proportion_firstBM_1 = glmmTMB(egg_binary ~ Temperature+Age, data= Fecundity_data_firstBM_ovipos_nar
m,family=binomial(link="logit")) #equals model 1
model.Proportion_firstBM_2 = glmmTMB(egg_binary ~ Temperature*Age, data= Fecundity_data_firstBM_ovipos_nar
m,family=binomial(link="logit")) #equals model 1
model.Proportion_firstBM_3 = glmmTMB(egg_binary ~ Temperature+Age+(1|Trial_number), data= Fecundity_data_fi
rstBM_ovipos_narm,family=binomial(link="logit")) #equals model 1
model.Proportion_firstBM_4 = glmmTMB(egg_binary ~ Temperature*Age+(1|Trial_number), data= Fecundity_data_fi
rstBM_ovipos_narm,family=binomial(link="logit")) #equals model 1

summary(model.Proportion_firstBM_1)
```

```
##  Family: binomial  ( logit )
## Formula:          egg_binary ~ Temperature + Age
## Data: Fecundity_data_firstBM_ovipos_narm
##
##      AIC      BIC   logLik deviance df.resid
##    771.5    798.3   -379.8    759.5      632
##
##
## Conditional model:
##                Estimate Std. Error z value Pr(>|z|)
## (Intercept)     0.1777     0.1934   0.919   0.3581
## Temperature30  -1.0949     0.1975  -5.544 2.96e-08 ***
## Temperature32  -1.9403     0.2530  -7.668 1.74e-14 ***
## Age5            0.9641     0.2270   4.248 2.16e-05 ***
## Age10           0.5579     0.2311   2.414   0.0158 *
## Age15          -0.4413     0.2877  -1.534   0.1251
## ---
## Signif. codes:  0 '***' 0.001 '**' 0.01 '*' 0.05 '.' 0.1 ' ' 1
```

```
summary(model.Proportion_firstBM_2)
```

```
##  Family: binomial  ( logit )
## Formula:          egg_binary ~ Temperature * Age
## Data: Fecundity_data_firstBM_ovipos_narm
##
##      AIC      BIC   logLik deviance df.resid
##    737.7    791.2   -356.9    713.7      626
##
##
## Conditional model:
##                       Estimate Std. Error z value Pr(>|z|)
## (Intercept)           -0.69315    0.26726  -2.594  0.00950 **
## Temperature30         -0.05885    0.34061  -0.173  0.86284
## Temperature32          0.16990    0.41348   0.411  0.68114
## Age5                   2.63904    0.48445   5.447 5.11e-08 ***
## Age10                  1.86588    0.41492   4.497 6.89e-06 ***
## Age15                  0.63908    0.42381   1.508  0.13157
## Temperature30:Age5    -1.86038    0.57674  -3.226  0.00126 **
## Temperature32:Age5    -3.94034    0.75265  -5.235 1.65e-07 ***
## Temperature30:Age10 -1.45036    0.53416  -2.715  0.00662 **
## Temperature32:Age10 -3.39678    0.70523  -4.817 1.46e-06 ***
## Temperature30:Age15 -2.15576    0.76943  -2.802  0.00508 **
## Temperature32:Age15 -1.87369    0.71668  -2.614  0.00894 **
## ---
## Signif. codes:  0 '***' 0.001 '**' 0.01 '*' 0.05 '.' 0.1 ' ' 1
```

```
summary(model.Proportion_firstBM_3)
```

```
##  Family: binomial  ( logit )
## Formula:          egg_binary ~ Temperature + Age + (1 | Trial_number)
## Data: Fecundity_data_firstBM_ovipos_narm
##
##      AIC      BIC   logLik deviance df.resid
##    769.0    800.2   -377.5    755.0      631
##
## Random effects:
##
## Conditional model:
##  Groups       Name        Variance Std.Dev.
##  Trial_number (Intercept) 0.06024  0.2454
## Number of obs: 638, groups:  Trial_number, 5
##
## Conditional model:
##               Estimate Std. Error z value Pr(>|z|)
## (Intercept)     0.1663     0.2284   0.728 0.466588
## Temperature30  -1.1320     0.2026  -5.587 2.31e-08 ***
## Temperature32  -1.9069     0.2557  -7.457 8.85e-14 ***
## Age5            0.9333     0.2408   3.876 0.000106 ***
## Age10           0.5639     0.2419   2.331 0.019756 *
## Age15          -0.5208     0.3001  -1.735 0.082708 .
## ---
## Signif. codes:  0 '***' 0.001 '**' 0.01 '*' 0.05 '.' 0.1 ' ' 1
```

```
summary(model.Proportion_firstBM_4)
```

```
##  Family: binomial  ( logit )
## Formula:          egg_binary ~ Temperature * Age + (1 | Trial_number)
## Data: Fecundity_data_firstBM_ovipos_narm
##
##      AIC      BIC   logLik deviance df.resid
##    729.9    787.9   -352.0    703.9      625
##
## Random effects:
##
## Conditional model:
##  Groups       Name        Variance Std.Dev.
##  Trial_number (Intercept) 0.1305   0.3613
## Number of obs: 638, groups:  Trial_number, 5
##
## Conditional model:
##                      Estimate Std. Error z value Pr(>|z|)
## (Intercept)          -0.86490    0.32860  -2.632 0.008487 **
## Temperature30         0.06421    0.35186   0.182 0.855195
## Temperature32         0.52226    0.44861   1.164 0.244357
## Age5                  2.84970    0.51275   5.558 2.73e-08 ***
## Age10                 2.16400    0.44830   4.827 1.38e-06 ***
## Age15                 0.80035    0.45332   1.766 0.077472 .
## Temperature30:Age5  -2.12675    0.59582  -3.569 0.000358 ***
## Temperature32:Age5  -4.25942    0.77868  -5.470 4.50e-08 ***
## Temperature30:Age10 -1.66937    0.54989  -3.036 0.002399 **
## Temperature32:Age10 -3.84452    0.74036  -5.193 2.07e-07 ***
## Temperature30:Age15 -2.39827    0.78548  -3.053 0.002264 **
## Temperature32:Age15 -2.34096    0.75954  -3.082 0.002056 **
## ---
## Signif. codes:  0 '***' 0.001 '**' 0.01 '*' 0.05 '.' 0.1 ' ' 1
```

```
library(lmtest)
lrtest(model.Proportion_firstBM_1,model.Proportion_firstBM_2) #bio rep does not matter
```

```
## Likelihood ratio test
##
## Model 1: egg_binary ~ Temperature + Age
## Model 2: egg_binary ~ Temperature * Age
##   #Df  LogLik Df Chisq Pr(>Chisq)
## 1   6 -379.77
## 2  12 -356.86  6 45.83  3.201e-08 ***
## ---
## Signif. codes:  0 '***' 0.001 '**' 0.01 '*' 0.05 '.' 0.1 ' ' 1
```

```
lrtest(model.Proportion_firstBM_1,model.Proportion_firstBM_3)#interaction matters
```

```
## Likelihood ratio test
##
## Model 1: egg_binary ~ Temperature + Age
## Model 2: egg_binary ~ Temperature + Age + (1 | Trial_number)
##   #Df  LogLik Df  Chisq Pr(>Chisq)
## 1   6 -379.77
## 2   7 -377.48  1 4.5816    0.03232 *
## ---
## Signif. codes:  0 '***' 0.001 '**' 0.01 '*' 0.05 '.' 0.1 ' ' 1
```

```
lrtest(model.Proportion_firstBM_3,model.Proportion_firstBM_4) #bio rep does not matter
```

```
## Likelihood ratio test
##
## Model 1: egg_binary ~ Temperature + Age + (1 | Trial_number)
## Model 2: egg_binary ~ Temperature * Age + (1 | Trial_number)
##   #Df  LogLik Df  Chisq Pr(>Chisq)
## 1   7 -377.48
## 2  13 -351.97  6 51.021  2.934e-09 ***
## ---
## Signif. codes:  0 '***' 0.001 '**' 0.01 '*' 0.05 '.' 0.1 ' ' 1
```

```
AIC(model.Proportion_firstBM_1,model.Proportion_firstBM_2,model.Proportion_firstBM_3,model.Proportion_first
BM_4)
```

```
##                            df      AIC
## model.Proportion_firstBM_1  6 771.5463
## model.Proportion_firstBM_2 12 737.7166
## model.Proportion_firstBM_3  7 768.9647
## model.Proportion_firstBM_4 13 729.9438
```

```
BIC(model.Proportion_firstBM_1,model.Proportion_firstBM_2,model.Proportion_firstBM_3,model.Proportion_first
BM_4)
```

```
##                            df      BIC
## model.Proportion_firstBM_1  6 798.2963
## model.Proportion_firstBM_2 12 791.2167
## model.Proportion_firstBM_3  7 800.1730
## model.Proportion_firstBM_4 13 787.9022
```

```
#interaction and trial number are both needed
#go with 4

sink("Oviposition_binary/BM1eggbinary_lrtests_significance.txt")
lrtest(model.Proportion_firstBM_1,model.Proportion_firstBM_2) #bio rep does not matter
lrtest(model.Proportion_firstBM_1,model.Proportion_firstBM_3)#interaction matters
lrtest(model.Proportion_firstBM_3,model.Proportion_firstBM_4) #bio rep does not matter
sink()

#check residuals
library(DHARMa)

plot(simulateResiduals(model.Proportion_firstBM_4))
```

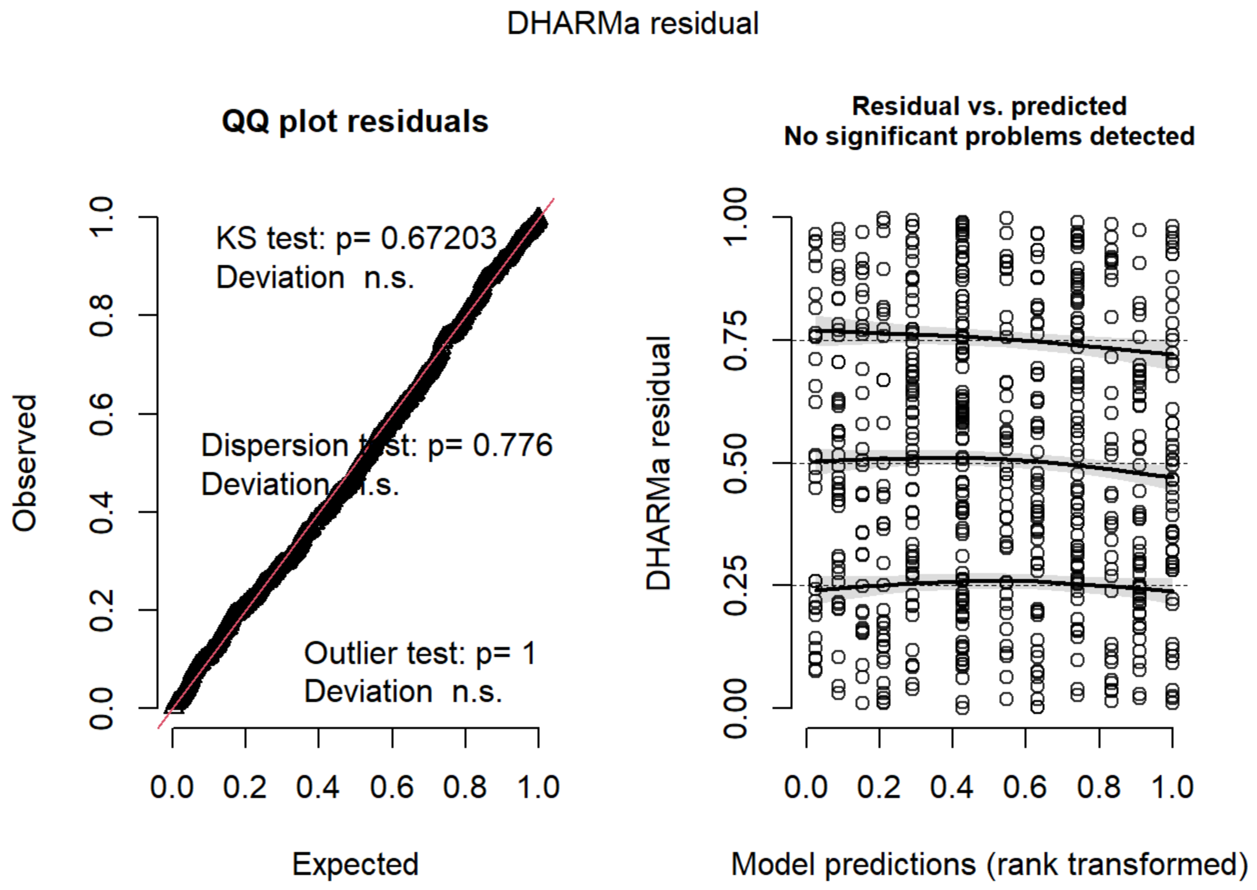


```
plot(fitted(model.Proportion_firstBM_4),
     residuals(model.Proportion_firstBM_4))
```

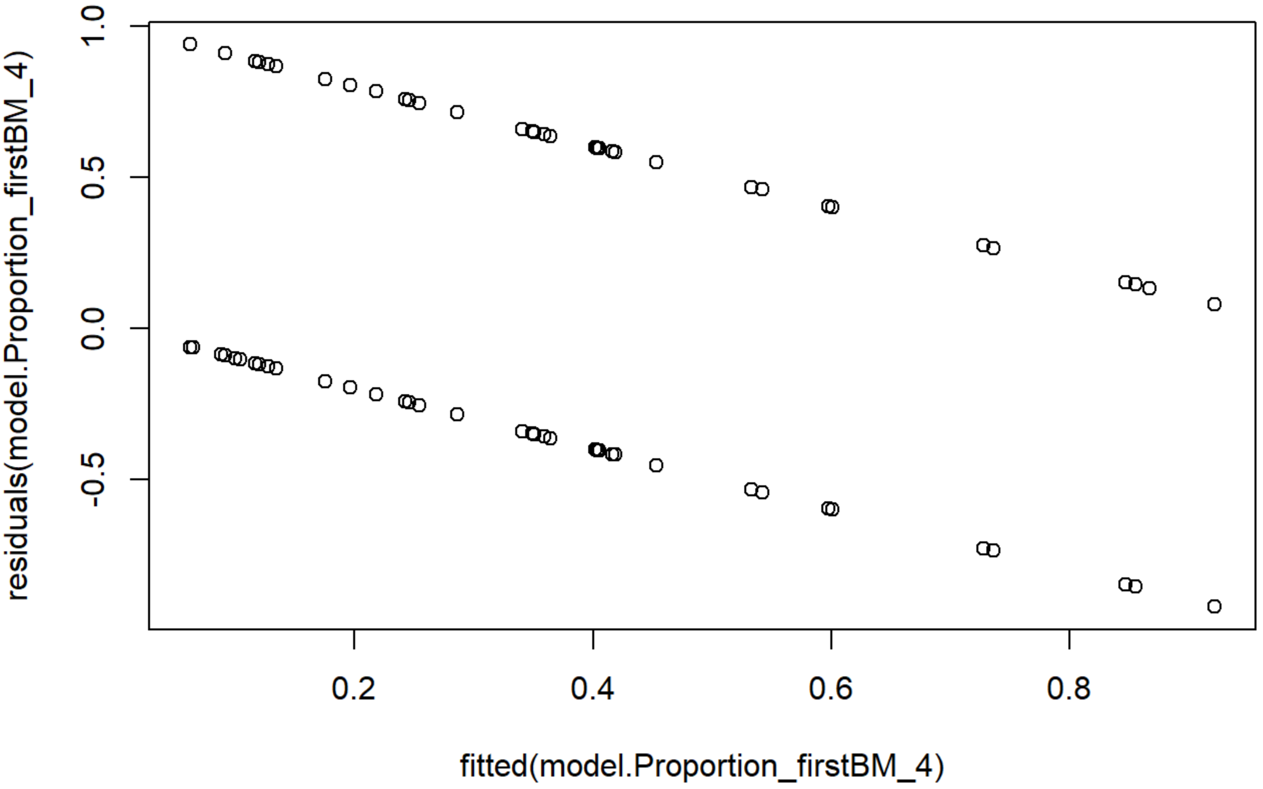


```r
#now check significance of factors in model
library(car)
anova_BM1_proportion <- Anova(model.Proportion_firstBM_4,type=2)
anova_BM1_proportion <- as.data.frame(anova_BM1_proportion)
anova_BM1_proportion
```

```
##                    Chisq Df   Pr(>Chisq)
## Temperature     41.58288  2 9.340999e-10
## Age             20.96128  3 1.072437e-04
## Temperature:Age 45.69940  6 3.397816e-08
```

```r
write_xlsx(anova_BM1_proportion,"Oviposition_binary/BM1_proportioneggs_ANOVA.xlsx")

sink("Oviposition_binary/BM1_proportioneggs_ANOVA.txt")
Anova(model.Proportion_firstBM_4,type=2)
sink()

sink("Oviposition_binary/BM1_proportioneggs_modelsummary.txt")
summary(model.Proportion_firstBM_4)
sink()

#effects and post hoc
library(effects)
ae <- allEffects(model.Proportion_firstBM_4)
plot(ae)
```

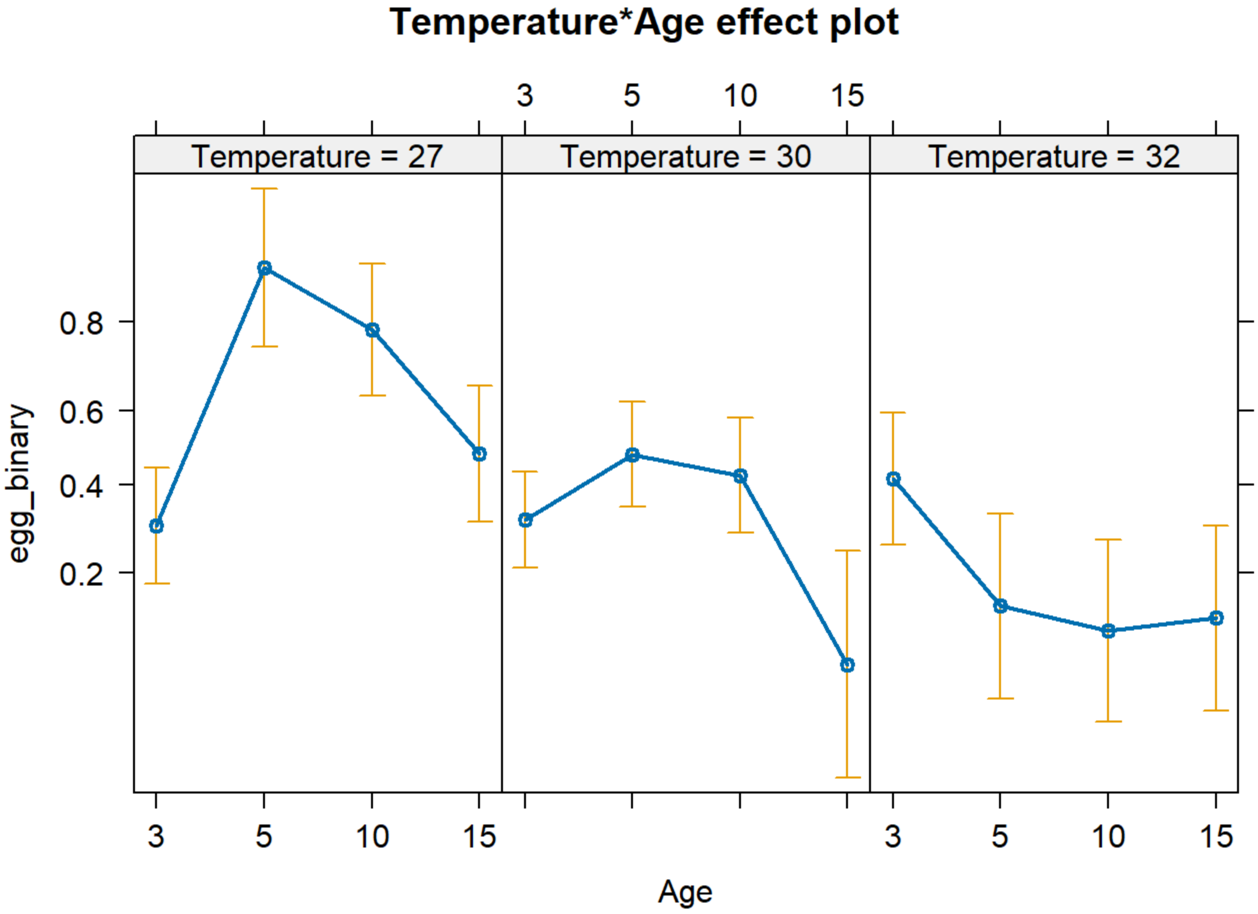


```
#post hoc comparisons:
###
library(emmeans)
BM1_proportion_emmeans <- emmeans(model.Proportion_firstBM_4,specs=c("Temperature","Age"),type="response")
BM1_proportion_emmeans <- as.data.frame(BM1_proportion_emmeans)
head(BM1_proportion_emmeans)
```

```
##   Temperature Age      prob         SE  df asymp.LCL asymp.UCL
##   27          3   0.2963176 0.06851749 Inf 0.1810961 0.4450105
##   30          3   0.3098792 0.05770060 Inf 0.2092408 0.4324505
##   32          3   0.4151685 0.09041255 Inf 0.2549338 0.5956062
##   27          5   0.8791924 0.04740281 Inf 0.7521430 0.9458094
##   30          5   0.4805758 0.07386306 Inf 0.3412594 0.6229798
##   32          5   0.1477493 0.06572030 Inf 0.0586728 0.3253236
##
## Confidence level used: 0.95
## Intervals are back-transformed from the logit scale
```

```
#save and re-run to make emmeans object again (Can't use if data frame)
write_xlsx(BM1_proportion_emmeans, "Oviposition_binary/BM1_proportioneggs_emmeans_all.xlsx")
BM1_proportion_emmeans <- emmeans(model.Proportion_firstBM_4,specs=c("Temperature","Age"),type="response")


BM1_proportion_emmeans_pwc <- pairs(BM1_proportion_emmeans,adjust="sidak")
BM1_proportion_emmeans_pwc <- as.data.frame(BM1_proportion_emmeans_pwc)
write_xlsx(BM1_proportion_emmeans_pwc, "Oviposition_binary/BM1_proportioneggs_emmeans_pairwisecontrasts.xls
x")
BM1_proportion_emmeans_pwc
```

```
##  contrast                                  odds.ratio       SE  df null z.ratio
##  Temperature27 Age3 / Temperature30 Age3      0.93781  0.32997 Inf    1  -0.182
##  Temperature27 Age3 / Temperature32 Age3      0.59318  0.26611 Inf    1  -1.164
##  Temperature27 Age3 / Temperature27 Age5      0.05786  0.02967 Inf    1  -5.558
##  Temperature27 Age3 / Temperature30 Age5      0.45514  0.17583 Inf    1  -2.038
##  Temperature27 Age3 / Temperature32 Age5      2.42897  1.41595 Inf    1   1.522
##  Temperature27 Age3 / Temperature27 Age10     0.11486  0.05149 Inf    1  -4.827
##  Temperature27 Age3 / Temperature30 Age10     0.57187  0.23046 Inf    1  -1.387
##  Temperature27 Age3 / Temperature32 Age10     3.18438  1.80019 Inf    1   2.049
##  Temperature27 Age3 / Temperature27 Age15     0.44917  0.20362 Inf    1  -1.766
##  Temperature27 Age3 / Temperature30 Age15     4.63531  3.15103 Inf    1   2.256
##  Temperature27 Age3 / Temperature32 Age15     2.76862  1.58615 Inf    1   1.778
##  Temperature30 Age3 / Temperature32 Age3      0.63252  0.25475 Inf    1  -1.137
##  Temperature30 Age3 / Temperature27 Age5      0.06170  0.02916 Inf    1  -5.895
##  Temperature30 Age3 / Temperature30 Age5      0.48532  0.16047 Inf    1  -2.186
##  Temperature30 Age3 / Temperature32 Age5      2.59006  1.41150 Inf    1   1.746
##  Temperature30 Age3 / Temperature27 Age10     0.12248  0.04932 Inf    1  -5.215
##  Temperature30 Age3 / Temperature30 Age10     0.60980  0.21664 Inf    1  -1.392
##  Temperature30 Age3 / Temperature32 Age10     3.39556  1.80937 Inf    1   2.294
##  Temperature30 Age3 / Temperature27 Age15     0.47896  0.19644 Inf    1  -1.795
##  Temperature30 Age3 / Temperature30 Age15     4.94271  3.23695 Inf    1   2.440
##  Temperature30 Age3 / Temperature32 Age15     2.95223  1.59508 Inf    1   2.004
##  Temperature32 Age3 / Temperature27 Age5      0.09754  0.05179 Inf    1  -4.384
##  Temperature32 Age3 / Temperature30 Age5      0.76728  0.31932 Inf    1  -0.637
##  Temperature32 Age3 / Temperature32 Age5      4.09483  2.46015 Inf    1   2.346
##  Temperature32 Age3 / Temperature27 Age10     0.19364  0.08771 Inf    1  -3.625
##  Temperature32 Age3 / Temperature30 Age10     0.96408  0.40399 Inf    1  -0.087
##  Temperature32 Age3 / Temperature32 Age10     5.36831  3.10827 Inf    1   2.902
##  Temperature32 Age3 / Temperature27 Age15     0.75722  0.35465 Inf    1  -0.594
##  Temperature32 Age3 / Temperature30 Age15     7.81432  5.46262 Inf    1   2.941
##  Temperature32 Age3 / Temperature32 Age15     4.66741  2.79289 Inf    1   2.575
##  Temperature27 Age5 / Temperature30 Age5      7.86593  3.78472 Inf    1   4.287
##  Temperature27 Age5 / Temperature32 Age5     41.97895 26.64648 Inf    1   5.888
##  Temperature27 Age5 / Temperature27 Age10     1.98515  1.05515 Inf    1   1.290
##  Temperature27 Age5 / Temperature30 Age10     9.88341  4.93496 Inf    1   4.588
##  Temperature27 Age5 / Temperature32 Age10    55.03427 35.16792 Inf    1   6.272
##  Temperature27 Age5 / Temperature27 Age15     7.76282  4.21317 Inf    1   3.776
##  Temperature27 Age5 / Temperature30 Age15    80.11010 59.85452 Inf    1   5.867
##  Temperature27 Age5 / Temperature32 Age15    47.84889 30.74580 Inf    1   6.020
##  Temperature30 Age5 / Temperature32 Age5      5.33681  2.93505 Inf    1   3.045
##  Temperature30 Age5 / Temperature27 Age10     0.25237  0.10476 Inf    1  -3.317
##  Temperature30 Age5 / Temperature30 Age10     1.25648  0.46190 Inf    1   0.621
##  Temperature30 Age5 / Temperature32 Age10     6.99654  3.78216 Inf    1   3.599
##  Temperature30 Age5 / Temperature27 Age15     0.98689  0.41243 Inf    1  -0.032
##  Temperature30 Age5 / Temperature30 Age15    10.18444  6.70927 Inf    1   3.523
##  Temperature30 Age5 / Temperature32 Age15     6.08306  3.30755 Inf    1   3.321
##  Temperature32 Age5 / Temperature27 Age10     0.04729  0.02843 Inf    1  -5.075
##  Temperature32 Age5 / Temperature30 Age10     0.23544  0.13476 Inf    1  -2.527
##  Temperature32 Age5 / Temperature32 Age10     1.31100  0.91421 Inf    1   0.388
##  Temperature32 Age5 / Temperature27 Age15     0.18492  0.11278 Inf    1  -2.767
##  Temperature32 Age5 / Temperature30 Age15     1.90834  1.52068 Inf    1   0.811
##  Temperature32 Age5 / Temperature32 Age15     1.13983  0.79346 Inf    1   0.188
##  Temperature27 Age10 / Temperature30 Age10    4.97867  2.08813 Inf    1   3.827
##  Temperature27 Age10 / Temperature32 Age10   27.72295 16.05589 Inf    1   5.736
##  Temperature27 Age10 / Temperature27 Age15    3.91044  1.83077 Inf    1   2.913
##  Temperature27 Age10 / Temperature30 Age15   40.35465 28.18387 Inf    1   5.295
```

```
##  Temperature27 Age10 / Temperature32 Age15   24.10339 14.40708 Inf    1   5.324
##  Temperature30 Age10 / Temperature32 Age10    5.56835  3.05533 Inf    1   3.129
##  Temperature30 Age10 / Temperature27 Age15    0.78544  0.33785 Inf    1  -0.561
##  Temperature30 Age10 / Temperature30 Age15    8.10551  5.43273 Inf    1   3.122
##  Temperature30 Age10 / Temperature32 Age15    4.84133  2.73316 Inf    1   2.794
##  Temperature32 Age10 / Temperature27 Age15    0.14105  0.08262 Inf    1  -3.344
##  Temperature32 Age10 / Temperature30 Age15    1.45564  1.13309 Inf    1   0.482
##  Temperature32 Age10 / Temperature32 Age15    0.86944  0.59960 Inf    1  -0.203
##  Temperature27 Age15 / Temperature30 Age15   10.31971  7.21738 Inf    1   3.337
##  Temperature27 Age15 / Temperature32 Age15    6.16385  3.69656 Inf    1   3.033
##  Temperature30 Age15 / Temperature32 Age15    0.59729  0.46904 Inf    1  -0.656
##  p.value
##   1.0000
##   1.0000
##   <.0001
##   0.9394
##   0.9999
##   0.0001
##   1.0000
##   0.9346
##   0.9951
##   0.7996
##   0.9944
##   1.0000
##   <.0001
##   0.8545
##   0.9961
##   <.0001
##   1.0000
##   0.7663
##   0.9931
##   0.6234
##   0.9525
##   0.0008
##   1.0000
##   0.7172
##   0.0189
##   1.0000
##   0.2172
##   1.0000
##   0.1945
##   0.4861
##   0.0012
##   <.0001
##   1.0000
##   0.0003
##   <.0001
##   0.0105
##   <.0001
##   <.0001
##   0.1425
##   0.0583
##   1.0000
##   0.0209
##   1.0000
##   0.0278
```

```
##   0.0576
##   <.0001
##   0.5343
##   1.0000
##   0.3121
##   1.0000
##   1.0000
##   0.0085
##   <.0001
##   0.2109
##   <.0001
##   <.0001
##   0.1093
##   1.0000
##   0.1119
##   0.2916
##   0.0531
##   1.0000
##   1.0000
##   0.0543
##   0.1480
##   1.0000
##
## P value adjustment: sidak method for 66 tests
## Tests are performed on the log odds ratio scale
```

```
BM1_proportion_emmeans_TEMP <- emmeans(model.Proportion_firstBM_4,specs=c("Temperature"),
                                       type="response")
```

```
## NOTE: Results may be misleading due to involvement in interactions
```

```
BM1_proportion_emmeans_TEMP
```

```
##  Temperature  prob     SE  df asymp.LCL asymp.UCL
##  27          0.643 0.0546 Inf     0.531     0.742
##  30          0.290 0.0518 Inf     0.200     0.401
##  32          0.182 0.0421 Inf     0.114     0.280
##
## Results are averaged over the levels of: Age
## Confidence level used: 0.95
## Intervals are back-transformed from the logit scale
```

```
BM1_proportion_emmeans_TEMP_pairs <- pairs(BM1_proportion_emmeans_TEMP, adjust="sidak")
BM1_proportion_emmeans_TEMP_pairs
```

```
##  contrast                      odds.ratio    SE  df null z.ratio p.value
##  Temperature27 / Temperature30       4.41 1.118 Inf    1   5.860  <.0001
##  Temperature27 / Temperature32       8.08 2.286 Inf    1   7.380  <.0001
##  Temperature30 / Temperature32       1.83 0.538 Inf    1   2.055  0.1148
##
## Results are averaged over the levels of: Age
## P value adjustment: sidak method for 3 tests
## Tests are performed on the log odds ratio scale
```

```
BM1_proportion_emmeans_TEMP_pairs <- as.data.frame(BM1_proportion_emmeans_TEMP_pairs)
write_xlsx(BM1_proportion_emmeans_TEMP_pairs, "Oviposition_binary/BM1_proprtioneggs_emmeans_TEMP_pairs.xls
x")

BM1_proportion_emmeans_AGE <- emmeans(model.Proportion_firstBM_4,specs=c("Age"),
                                      type="response")
```

```
## NOTE: Results may be misleading due to involvement in interactions
```

```
BM1_proportion_emmeans_AGE
```

```
##  Age  prob     SE  df asymp.LCL asymp.UCL
##  3   0.339 0.0512 Inf     0.246     0.445
##  5   0.513 0.0717 Inf     0.375     0.649
##  10  0.415 0.0679 Inf     0.291     0.551
##  15  0.190 0.0517 Inf     0.108     0.312
##
## Results are averaged over the levels of: Temperature
## Confidence level used: 0.95
## Intervals are back-transformed from the logit scale
```

```
BM1_proportion_emmeans_AGE_pairs <- pairs(BM1_proportion_emmeans_AGE, adjust="sidak")
BM1_proportion_emmeans_AGE_pairs <- as.data.frame(BM1_proportion_emmeans_AGE_pairs)
write_xlsx(BM1_proportion_emmeans_AGE_pairs, "Oviposition_binary/BM1_proprtioneggs_emmeans_AGE_pairs.xlsx")

sink("Oviposition_binary/modelparameters_BM1_proportioneggs.txt")
parameters::model_parameters(
  model.Proportion_firstBM_4, exponentiate = TRUE, ci_method = "wald",
  effects = "all",
  component = "conditional",
  group_level = TRUE,
  verbose = FALSE
)
sink()
```

Code for Figure 5 - total eggs laid (if laid any eggs) and oviposition timing (percent laid day 3 vs. day 4)

Import data and clean it up

```
################
# clear existing workspace
rm(list = ls(all = TRUE))
graphics.off()
shell("cls")

#set wd to your project folder
getwd() #check working directory
```

```
## [1] "C:/Users/linzm/OneDrive - Vanderbilt/Hillyer_Lab/Blood_feeding_project/Bloodfeeding"
```

```
###########################
#####################
#load libraries needed:
library(readxl)
library(writexl)
library(ggplot2)
library(dplyr)
library(tidyverse)
library(rstatix)
library(car)
library(ggpubr)
library(emmeans)

######################################################
#import the data and clean it up:

Fecundity_data <- read_xlsx("SupplementaryData1_RawData.xlsx",
                            sheet = "Figs4-9")
Fecundity_data <- as.data.frame(Fecundity_data)
str(Fecundity_data)
```

```
## 'data.frame':    849 obs. of  18 variables:
##  $ ID_overall               : num  1 2 3 4 5 6 7 8 9 10 ...
##  $ Temperature              : num  32 32 32 32 32 32 32 32 32 32 ...
##  $ Age                      : num  3 3 3 3 3 3 3 3 3 3 ...
##  $ ID_per_group             : num  1 2 3 4 5 6 7 8 9 10 ...
##  $ Trial_start_date         : POSIXct, format: "2024-02-13" "2024-02-13" ...
##  $ Trial_number             : num  1 1 1 1 1 1 1 1 1 1 ...
##  $ BM1_Date                 : POSIXct, format: "2024-02-13" "2024-02-13" ...
##  $ Age_of_BM                : num  3 3 3 3 3 3 3 3 3 3 ...
##  $ Bloodmeal_number         : num  1 1 1 1 1 1 1 1 1 1 ...
##  $ Oviposition_positive(y/n): chr  "Y" "Y" "Y" "Y" ...
##  $ Eggs_day3                : chr  "0" "0" "0" "0" ...
##  $ Eggs_day4                : chr  "0" "0" "0" "0" ...
##  $ Larvae_day4              : chr  "0" "0" "0" "0" ...
##  $ Surv_to_eggs(y/n)        : chr  "Y" "Y" "Y" "Y" ...
##  $ Surv_to_larvae(y/n)      : chr  "Y" "Y" "Y" "Y" ...
##  $ Date_of_death            : POSIXct, format: "2024-03-01" "2024-03-07" ...
##  $ Censor                   : num  1 1 1 1 1 1 1 1 1 1 ...
##  $ Notes                    : chr  NA NA NA NA ...
```

```
head(Fecundity_data)
```

```
##   ID_overall Temperature Age ID_per_group Trial_start_date Trial_number
## 1          1          32   3            1       2024-02-13            1
## 2          2          32   3            2       2024-02-13            1
## 3          3          32   3            3       2024-02-13            1
## 4          4          32   3            4       2024-02-13            1
## 5          5          32   3            5       2024-02-13            1
## 6          6          32   3            6       2024-02-13            1
##     BM1_Date Age_of_BM Bloodmeal_number Oviposition_positive(y/n) Eggs_day3
## 1 2024-02-13         3                1                         Y         0
## 2 2024-02-13         3                1                         Y         0
## 3 2024-02-13         3                1                         Y         0
## 4 2024-02-13         3                1                         Y         0
## 5 2024-02-13         3                1                         Y         0
## 6 2024-02-13         3                1                         Y        14
##   Eggs_day4 Larvae_day4 Surv_to_eggs(y/n) Surv_to_larvae(y/n) Date_of_death
## 1         0           0                 Y                   Y    2024-03-01
## 2         0           0                 Y                   Y    2024-03-07
## 3         0           0                 Y                   Y    2024-03-02
## 4         0           0                 Y                   Y    2024-02-26
## 5         0           0                 Y                   Y    2024-02-28
## 6        14           0                 Y                   Y    2024-03-02
##   Censor Notes
## 1      1  <NA>
## 2      1  <NA>
## 3      1  <NA>
## 4      1  <NA>
## 5      1  <NA>
## 6      1  <NA>
```

```
Fecundity_data_numeric <- Fecundity_data

#variables of interest:
Fecundity_data$Temperature <- as.factor(Fecundity_data$Temperature)
Fecundity_data$Age <- as.factor(Fecundity_data$Age)
Fecundity_data$Age_of_BM <- as.numeric(Fecundity_data$Age_of_BM)
Fecundity_data$Bloodmeal_number <- as.factor(Fecundity_data$Bloodmeal_number)

Fecundity_data$Eggs_day3 <- as.numeric(Fecundity_data$Eggs_day3)
```

```
## Warning: NAs introduced by coercion
```

```
Fecundity_data$Eggs_day4 <- as.numeric(Fecundity_data$Eggs_day4)
```

```
## Warning: NAs introduced by coercion
```

```
Fecundity_data$Larvae_day4 <- as.numeric(Fecundity_data$Larvae_day4)
```

```
## Warning: NAs introduced by coercion
```

```
Fecundity_data$Oviposition_positive <- as.factor(Fecundity_data$Oviposition_positive)
Fecundity_data$`Surv_to_eggs(y/n)` <- as.factor(Fecundity_data$`Surv_to_eggs(y/n)`)
Fecundity_data$`Surv_to_larvae(y/n)` <- as.factor(Fecundity_data$`Surv_to_larvae(y/n)`)
str(Fecundity_data)
```

```
## 'data.frame':    849 obs. of  19 variables:
##  $ ID_overall              : num  1 2 3 4 5 6 7 8 9 10 ...
##  $ Temperature             : Factor w/ 3 levels "27","30","32": 3 3 3 3 3 3 3 3 3 3 ...
##  $ Age                     : Factor w/ 4 levels "3","5","10","15": 1 1 1 1 1 1 1 1 1 1 ...
##  $ ID_per_group            : num  1 2 3 4 5 6 7 8 9 10 ...
##  $ Trial_start_date        : POSIXct, format: "2024-02-13" "2024-02-13" ...
##  $ Trial_number            : num  1 1 1 1 1 1 1 1 1 1 ...
##  $ BM1_Date                : POSIXct, format: "2024-02-13" "2024-02-13" ...
##  $ Age_of_BM               : num  3 3 3 3 3 3 3 3 3 3 ...
##  $ Bloodmeal_number        : Factor w/ 1 level "1": 1 1 1 1 1 1 1 1 1 1 ...
##  $ Oviposition_positive(y/n): chr  "Y" "Y" "Y" "Y" ...
##  $ Eggs_day3               : num  0 0 0 0 0 14 NA NA NA NA ...
##  $ Eggs_day4               : num  0 0 0 0 0 14 NA NA NA NA ...
##  $ Larvae_day4             : num  0 0 0 0 0 0 0 0 NA NA ...
##  $ Surv_to_eggs(y/n)       : Factor w/ 3 levels "N","NA","Y": 3 3 3 3 3 3 1 1 3 3 ...
##  $ Surv_to_larvae(y/n)     : Factor w/ 3 levels "N","NA","Y": 3 3 3 3 3 3 2 2 3 3 ...
##  $ Date_of_death           : POSIXct, format: "2024-03-01" "2024-03-07" ...
##  $ Censor                  : num  1 1 1 1 1 1 1 1 1 1 ...
##  $ Notes                   : chr  NA NA NA NA ...
##  $ Oviposition_positive    : Factor w/ 2 levels "N","Y": 2 2 2 2 2 2 2 2 1 1 ...
```

```
Fecundity_data <- subset(Fecundity_data, Censor == 1) #get rid of mosquitoes censored out by experimental e
rror (get rid of 0 values; 1 = died naturally)

#calculate the total eggs laid per mosquito:

#need to subtract to find ones only laid on day 4 (exclude day 3 eggs)
Fecundity_data$Eggs_day4 <- (Fecundity_data$Eggs_day4)-(Fecundity_data$Eggs_day3)

#replace negative eggs day 4 values with zero (assume miscounted/eggs degraded and no new eggs laid)
for (row in 1:nrow(Fecundity_data)){
  if (is.na(Fecundity_data$Eggs_day4[row])){
    Fecundity_data$Eggs_day4[row] <- NA #keep NA values
  } else if ((Fecundity_data$Eggs_day4[row] <= 0)){
    Fecundity_data$Eggs_day4[row] <- 0
  }
}

#total eggs addition:
Fecundity_data$total_eggs <- (Fecundity_data$Eggs_day3)+(Fecundity_data$Eggs_day4)

#percents:
Fecundity_data$Percent_eggs_day3 <- (Fecundity_data$Eggs_day3) / (Fecundity_data$total_eggs)
Fecundity_data$Percent_eggs_day4 <- (Fecundity_data$Eggs_day4) / (Fecundity_data$total_eggs)

# decide if each mosquito laid eggs and on what day
#day 3
for (row in 1:nrow(Fecundity_data)){
  if(!is.na(Fecundity_data$Eggs_day3[row])){
    if (Fecundity_data$Eggs_day3[row] > 0){
      Fecundity_data$egg_binary[row] = 1
      Fecundity_data$egg_binary_day3[row] = 1
    }
    else {
      Fecundity_data$egg_binary[row] = 0
      Fecundity_data$egg_binary_day3[row] = 0
    }
  }
  else if (is.na(Fecundity_data$Eggs_day3[row])){
    Fecundity_data$egg_binary[row] = NA
    Fecundity_data$egg_binary_day3[row] = NA
  }
}

#day 4
for (row in 1:nrow(Fecundity_data)){
  if(!is.na(Fecundity_data$Eggs_day4[row])){
    if (Fecundity_data$Eggs_day4[row] >0){
      Fecundity_data$egg_binary[row] = 1
      Fecundity_data$egg_binary_day4[row] = 1
    }
    else{
      Fecundity_data$egg_binary_day4[row] = 0
    }
  }
  else if (is.na(Fecundity_data$Eggs_day4[row])){
    Fecundity_data$egg_binary_day4[row] = NA
```

```
  }
}

# decide if each mosquito had larvae
for (row in 1:nrow(Fecundity_data)){
  if(!is.na(Fecundity_data$Larvae_day4[row])){
    if (Fecundity_data$Larvae_day4[row] >0){
      Fecundity_data$larvae_binary[row] = 1
    }
    else{
      Fecundity_data$larvae_binary[row] = 0
    }
  }
  else{
    Fecundity_data$larvae_binary[row] = NA
  }
}

#percents:
Fecundity_data$Percent_eggshatchedtolarv <- (Fecundity_data$Larvae_day4) / (Fecundity_data$Eggs_day3)

#survival:
#calculate:
Fecundity_data$Days_to_death_post_BM <- Fecundity_data$Date_of_death - Fecundity_data$Trial_start_date
Fecundity_data$Age_of_death <- Fecundity_data$Age_of_BM + Fecundity_data$Days_to_death_post_BM

Fecundity_data <-
  Fecundity_data %>%
  mutate(
    Age_of_BM_days = as.difftime(Age_of_BM, unit="days")
  )
Fecundity_data$Date_of_eclosion <- Fecundity_data$Trial_start_date - (Fecundity_data$Age_of_BM_days)

library(lubridate)
Fecundity_data <-
  Fecundity_data %>%
  mutate(
    days_alive_post_BM = as.duration(Trial_start_date %--% Date_of_death) / ddays(1),
    days_alive_post_eclosion = as.duration(Date_of_eclosion %--% Date_of_death) / ddays(1),
  )

str(Fecundity_data)
```

```
## 'data.frame':    842 obs. of  33 variables:
##  $ ID_overall               : num  1 2 3 4 5 6 7 8 9 10 ...
##  $ Temperature              : Factor w/ 3 levels "27","30","32": 3 3 3 3 3 3 3 3 3 3 ...
##  $ Age                      : Factor w/ 4 levels "3","5","10","15": 1 1 1 1 1 1 1 1 1 1 ...
##  $ ID_per_group             : num  1 2 3 4 5 6 7 8 9 10 ...
##  $ Trial_start_date         : POSIXct, format: "2024-02-13" "2024-02-13" ...
##  $ Trial_number             : num  1 1 1 1 1 1 1 1 1 1 ...
##  $ BM1_Date                 : POSIXct, format: "2024-02-13" "2024-02-13" ...
##  $ Age_of_BM                : num  3 3 3 3 3 3 3 3 3 3 ...
##  $ Bloodmeal_number         : Factor w/ 1 level "1": 1 1 1 1 1 1 1 1 1 1 ...
##  $ Oviposition_positive(y/n): chr  "Y" "Y" "Y" "Y" ...
##  $ Eggs_day3                : num  0 0 0 0 0 14 NA NA NA NA ...
##  $ Eggs_day4                : num  0 0 0 0 0 0 NA NA NA NA ...
##  $ Larvae_day4              : num  0 0 0 0 0 0 0 0 NA NA ...
##  $ Surv_to_eggs(y/n)        : Factor w/ 3 levels "N","NA","Y": 3 3 3 3 3 3 1 1 3 3 ...
##  $ Surv_to_larvae(y/n)      : Factor w/ 3 levels "N","NA","Y": 3 3 3 3 3 3 2 2 3 3 ...
##  $ Date_of_death            : POSIXct, format: "2024-03-01" "2024-03-07" ...
##  $ Censor                   : num  1 1 1 1 1 1 1 1 1 1 ...
##  $ Notes                    : chr  NA NA NA NA ...
##  $ Oviposition_positive     : Factor w/ 2 levels "N","Y": 2 2 2 2 2 2 2 2 1 1 ...
##  $ total_eggs               : num  0 0 0 0 0 14 NA NA NA NA ...
##  $ Percent_eggs_day3        : num  NaN NaN NaN NaN NaN 1 NA NA NA NA ...
##  $ Percent_eggs_day4        : num  NaN NaN NaN NaN NaN 0 NA NA NA NA ...
##  $ egg_binary               : num  0 0 0 0 0 1 NA NA NA NA ...
##  $ egg_binary_day3          : num  0 0 0 0 0 1 NA NA NA NA ...
##  $ egg_binary_day4          : num  0 0 0 0 0 0 NA NA NA NA ...
##  $ larvae_binary            : num  0 0 0 0 0 0 0 0 NA NA ...
##  $ Percent_eggshatchedtolarv: num  NaN NaN NaN NaN NaN 0 NA NA NA NA ...
##  $ Days_to_death_post_BM    : 'difftime' num  17 23 18 13 ...
##   ..- attr(*, "units")= chr "days"
##  $ Age_of_death             : 'difftime' num  20 26 21 16 ...
##   ..- attr(*, "units")= chr "days"
##  $ Age_of_BM_days           : 'difftime' num  3 3 3 3 ...
##   ..- attr(*, "units")= chr "days"
##  $ Date_of_eclosion         : POSIXct, format: "2024-02-10" "2024-02-10" ...
##  $ days_alive_post_BM       : num  17 23 18 13 15 18 3 3 12 11 ...
##  $ days_alive_post_eclosion : num  20 26 21 16 18 21 6 6 15 14 ...
```

```
#subset by oviposition positive and negative:
Fecundity_data_ovipos <- subset(Fecundity_data, Oviposition_positive== "Y")
Fecundity_data_ovineg <- subset(Fecundity_data, Oviposition_positive== "N")

####
#Filter to only look at mosquitoes that laid eggs (and also survived to day 3)
#run this for total eggs, percent eggs day 3 v. day 4
Fecundity_data_ovipos_eggsgreaterthanzero <- subset(Fecundity_data_ovipos,total_eggs > 0)
Fecundity_data_ovipos <- Fecundity_data_ovipos_eggsgreaterthanzero
#################
```

Calculate summary stats:

```
library(dplyr)
#calculate summary stats:
Summary_Fecundity_data_ovipos <- Fecundity_data_ovipos %>%
  group_by(Temperature,Age,Oviposition_positive) %>%
  dplyr::summarise(mean_eggsday3 = mean(Eggs_day3, na.rm = TRUE),
                   mean_eggsday4 = mean(Eggs_day4, na.rm = TRUE),
                   mean_totaleggs= mean(total_eggs, na.rm = TRUE),
                   mean_percenteggs_day3 = mean(Percent_eggs_day3, na.rm = TRUE), #% of total eggs laid on 
day 3
                   mean_percenteggs_day4 = mean(Percent_eggs_day4, na.rm = TRUE),#% of total eggs laid on d
ay 4
                   mean_larvae_day4 = mean(Larvae_day4, na.rm = TRUE),
                   mean_Percent_eggshatchedtolarv = mean(Percent_eggshatchedtolarv, na.rm = TRUE), #of the 
day3 eggs, what percent hatched?
                   #binary proportions:
                   sum_egg_binary = sum(egg_binary, na.rm = TRUE), #gives total number of mosquitoes per gr
oup that laid eggs
                   sum_surv_to_day3 = sum(`Surv_to_eggs(y/n)`=="Y"), #gives total number of mosquitoes per 
group that survived to day 3 post BM
                   sum_surv_to_day4 = sum(`Surv_to_larvae(y/n)`=="Y"), #gives total number of mosquitoes pe
r group that survived to day 4 post BM
                   sum_egg_binary_day3 = sum(egg_binary_day3, na.rm = TRUE), #number of eggs laid on day 3
                   sum_egg_binary_day4 = sum(egg_binary_day4, na.rm = TRUE), #number of eggs laid on day 4
                   sum_larvae_binary = sum(larvae_binary, na.rm = TRUE), #number of larvae counted on day 4
                   proportion_binaryegg = (sum_egg_binary / n()), #what proportion laid any number of eggs? 
#remove NAs, but keep all mosquitoes bc some laid eggs even if dead on day 3 count
                   proportion_binaryegg_day3 = (sum_egg_binary_day3 / sum_egg_binary), #what proportion lai
d eggs on day 3, out of ones that laid eggs?
                   proportion_binaryegg_day4 = (sum_egg_binary_day4 / sum_egg_binary), #what proportion lai
d eggs on day 4, out of ones that laid eggs?
                   proportion_binarylarvae = (sum_larvae_binary / sum_egg_binary_day3), #of ones that had e
ggs on day 3, what proportion had any # of larvae hatch?
                   #sample sizes
                   n_mosquitoes = n(), #total n
                   #standard errors
                   SE_eggsday3 = sd(Eggs_day3,na.rm = TRUE)/sqrt(n()),
                   SE_eggsday4 = sd(Eggs_day4,na.rm = TRUE)/sqrt(n()),
                   SE_totaleggs = sd(total_eggs,na.rm = TRUE)/sqrt(n()),
                   SE_percenteggs_day3 = sd(Percent_eggs_day3,na.rm = TRUE)/sqrt(n()),
                   SE_percenteggs_day4 = sd(Percent_eggs_day4,na.rm = TRUE)/sqrt(n()),
                   SE_larvae_day4 = sd(Larvae_day4,na.rm = TRUE)/sqrt(n()),
                   SE_percenteggshatched = sd(Percent_eggshatchedtolarv,na.rm = TRUE)/sqrt(n()))
```

```
## `summarise()` has grouped output by 'Temperature', 'Age'. You can override
## using the `.groups` argument.
```

```
#Summary_Fecundity_data_firstBM_ovipos <- as.data.frame(Summary_Fecundity_data_firstBM_ovipos)
Summary_Fecundity_data_ovipos <- as.data.frame(Summary_Fecundity_data_ovipos)

str(Summary_Fecundity_data_ovipos)
```

```
## 'data.frame':	12 obs. of  28 variables:
##  $ Temperature                 : Factor w/ 3 levels "27","30","32": 1 1 1 1 2 2 2 2 3 3 ...
##  $ Age                         : Factor w/ 4 levels "3","5","10","15": 1 2 3 4 1 2 3 4 1 2 ...
##  $ Oviposition_positive        : Factor w/ 2 levels "N","Y": 2 2 2 2 2 2 2 2 2 2 ...
##  $ mean_eggsday3               : num  51.6 47.9 35.5 11.2 42.3 ...
##  $ mean_eggsday4               : num  5.95 2.45 2.78 4.72 6.12 ...
##  $ mean_totaleggs              : num  57.6 50.4 38.3 15.9 48.5 ...
##  $ mean_percenteggs_day3       : num  0.919 0.886 0.918 0.649 0.859 ...
##  $ mean_percenteggs_day4       : num  0.0812 0.1143 0.0817 0.3514 0.1413 ...
##  $ mean_larvae_day4            : num  0.85 22 19.6 0.278 22 ...
##  $ mean_Percent_eggshatchedtolarv: num  0.0123 0.3988 0.5064 0.0142 0.3193 ...
##  $ sum_egg_binary              : num  21 47 36 18 33 38 25 3 16 5 ...
##  $ sum_surv_to_day3            : int  20 45 33 14 29 36 21 2 16 5 ...
##  $ sum_surv_to_day4            : int  17 41 31 10 23 32 20 2 14 5 ...
##  $ sum_egg_binary_day3         : num  20 42 34 13 30 31 22 2 15 4 ...
##  $ sum_egg_binary_day4         : num  9 9 8 8 14 24 5 2 8 2 ...
##  $ sum_larvae_binary           : num  3 28 27 1 16 22 15 0 2 0 ...
##  $ proportion_binaryegg        : num  1 1 1 1 1 1 1 1 1 1 ...
##  $ proportion_binaryegg_day3   : num  0.952 0.894 0.944 0.722 0.909 ...
##  $ proportion_binaryegg_day4   : num  0.429 0.191 0.222 0.444 0.424 ...
##  $ proportion_binarylarvae     : num  0.15 0.6667 0.7941 0.0769 0.5333 ...
##  $ n_mosquitoes                : int  21 47 36 18 33 38 25 3 16 5 ...
##  $ SE_eggsday3                 : num  6.34 3.34 2.75 2.85 5.32 ...
##  $ SE_eggsday4                 : num  3.67 1 1.16 1.66 2.5 ...
##  $ SE_totaleggs                : num  6.04 2.81 2.48 2.31 5.12 ...
##  $ SE_percenteggs_day3         : num  0.0478 0.0453 0.0392 0.1093 0.0517 ...
##  $ SE_percenteggs_day4         : num  0.0478 0.0453 0.0392 0.1093 0.0517 ...
##  $ SE_larvae_day4              : num  0.536 3.242 2.637 0.278 4.828 ...
##  $ SE_percenteggshatched       : num  0.00703 0.05001 0.05684 0.01211 0.06435 ...
```

```
write_xlsx(Summary_Fecundity_data_ovipos, "Oviposition_eggsandlarvae/Summary_Fecundity_data_ovipos.xlsx")



Summary_Fecundity_data_ovipos_proportions <- Fecundity_data_ovipos %>% #Fecundity_data_firstBM_ovipos %>%
  group_by(Temperature,Age,Oviposition_positive,Trial_number) %>%
  dplyr::summarise(#binary proportions:
    sum_egg_binary = sum(egg_binary, na.rm = TRUE), #gives total number of mosquitoes per group that laid e
ggs
    sum_surv_to_day3 = sum(`Surv_to_eggs(y/n)`=="Y"), #gives total number of mosquitoes per group that surv
ived to day 3 post BM
    sum_surv_to_day4 = sum(`Surv_to_larvae(y/n)`=="Y"), #gives total number of mosquitoes per group that su
rvived to day 4 post BM
    sum_egg_binary_day3 = sum(egg_binary_day3, na.rm = TRUE), #number of eggs laid on day 3
    sum_egg_binary_day4 = sum(egg_binary_day4, na.rm = TRUE), #number of eggs laid on day 4
    sum_larvae_binary = sum(larvae_binary, na.rm = TRUE), #number of larvae counted on day 4
    proportion_binaryegg = (sum_egg_binary / n()), #what proportion laid any number of eggs? #remove NAs, b
ut keep all mosquitoes bc some laid eggs even if dead on day 3 count
    proportion_binaryegg_day3 = (sum_egg_binary_day3 / sum_egg_binary), #what proportion laid eggs on day
3, out of ones that laid eggs?
    proportion_binaryegg_day4 = (sum_egg_binary_day4 / sum_egg_binary), #what proportion laid eggs on day
4, out of ones that laid eggs?
    proportion_binarylarvae = (sum_larvae_binary / sum_egg_binary_day3), #of ones that had eggs on day 3, w
hat proportion had any # of larvae hatch?
    proportion_surv_to_day3 = (sum_surv_to_day3 /n()),
    proportion_surv_to_day4 = (sum_surv_to_day4 /n()),
    #sample sizes
    n_mosquitoes = n()) #total n
```

```
## `summarise()` has grouped output by 'Temperature', 'Age',
## 'Oviposition_positive'. You can override using the `.groups` argument.
```

```
#standard errors

Summary_Fecundity_data_ovipos_proportions <- as.data.frame(Summary_Fecundity_data_ovipos_proportions)
write_xlsx(Summary_Fecundity_data_ovipos_proportions, "Oviposition_eggsandlarvae/Summary_Fecundity_data_ovi
pos_proportions.xlsx")

Summary_Fecundity_data_ovipos_proportions_means <- Summary_Fecundity_data_ovipos_proportions %>% #Fecundity
_data_firstBM_ovipos %>%
  group_by(Temperature,Age,Oviposition_positive) %>%
  dplyr::summarise(#binary proportions:
    mean_proportion_binaryegg = mean(proportion_binaryegg,na.rm = TRUE), #what proportion laid any number o
f eggs? #remove NAs, but keep all mosquitoes bc some laid eggs even if dead on day 3 count
    mean_proportion_binaryegg_day3 = mean(proportion_binaryegg_day3,na.rm = TRUE), #what proportion laid eg
gs on day 3, out of ones that laid eggs?
    mean_proportion_binaryegg_day4 = mean(proportion_binaryegg_day4,na.rm = TRUE), #what proportion laid eg
gs on day 4, out of ones that laid eggs?
    mean_proportion_binarylarvae = mean(proportion_binarylarvae,na.rm = TRUE), #of ones that had eggs on da
y 3, what proportion had any # of larvae hatch?
    mean_proportion_surv_to_day3 = mean(proportion_surv_to_day3,na.rm = TRUE),
    mean_proportion_surv_to_day4 = mean(proportion_surv_to_day4,na.rm = TRUE),
    #sample sizes
    n_mosquitoes = sum(n_mosquitoes),
    n_trials = n(),
    SE_proportion_binaryegg = sd(proportion_binaryegg,na.rm = TRUE)/sqrt(n()),
    SE_proportion_binaryegg_day3 = sd(proportion_binaryegg_day3,na.rm = TRUE)/sqrt(n()),
    SE_proportion_binaryegg_day4 = sd(proportion_binaryegg_day4,na.rm = TRUE)/sqrt(n()),
    SE_proportion_binarylarvae = sd(proportion_binarylarvae,na.rm = TRUE)/sqrt(n()),
    SE_proportion_surv_to_day3 = sd(proportion_surv_to_day3,na.rm = TRUE)/sqrt(n()),
    SE_proportion_surv_to_day4 = sd(proportion_surv_to_day4,na.rm = TRUE)/sqrt(n()))
```

```
## `summarise()` has grouped output by 'Temperature', 'Age'. You can override
## using the `.groups` argument.
```

```
Summary_Fecundity_data_ovipos_proportions_means <- as.data.frame(Summary_Fecundity_data_ovipos_proportions_
means)
write_xlsx(Summary_Fecundity_data_ovipos_proportions_means, "Oviposition_eggsandlarvae/Summary_Fecundity_da
ta_ovipos_proportions_means.xlsx")


#TEMP only:
Summary_Fecundity_data_ovipos_TEMP <- Fecundity_data_ovipos %>%
  group_by(Temperature) %>%
  dplyr::summarise(mean_eggsday3 = mean(Eggs_day3, na.rm = TRUE),
                   mean_eggsday4 = mean(Eggs_day4, na.rm = TRUE),
                   mean_totaleggs= mean(total_eggs, na.rm = TRUE),
                   mean_percenteggs_day3 = mean(Percent_eggs_day3, na.rm = TRUE), #% of total eggs laid on
day 3
                   mean_percenteggs_day4 = mean(Percent_eggs_day4, na.rm = TRUE),#% of total eggs laid on d
ay 4
                   mean_larvae_day4 = mean(Larvae_day4, na.rm = TRUE),
                   mean_Percent_eggshatchedtolarv = mean(Percent_eggshatchedtolarv, na.rm = TRUE), #of the
day3 eggs, what percent hatched?
                   #binary proportions:
                   sum_egg_binary = sum(egg_binary, na.rm = TRUE), #gives total number of mosquitoes per gr
oup that laid eggs
                   sum_surv_to_day3 = sum(`Surv_to_eggs(y/n)`=="Y"), #gives total number of mosquitoes per
group that survived to day 3 post BM
                   sum_surv_to_day4 = sum(`Surv_to_larvae(y/n)`=="Y"), #gives total number of mosquitoes pe
r group that survived to day 4 post BM
                   sum_egg_binary_day3 = sum(egg_binary_day3, na.rm = TRUE), #number of eggs laid on day 3
                   sum_egg_binary_day4 = sum(egg_binary_day4, na.rm = TRUE), #number of eggs laid on day 4
                   sum_larvae_binary = sum(larvae_binary, na.rm = TRUE), #number of larvae counted on day 4
                   proportion_binaryegg = (sum_egg_binary / n()), #what proportion laid any number of eggs?
#remove NAs, but keep all mosquitoes bc some laid eggs even if dead on day 3 count
                   proportion_binaryegg_day3 = (sum_egg_binary_day3 / sum_egg_binary), #what proportion lai
d eggs on day 3, out of ones that laid eggs?
                   proportion_binaryegg_day4 = (sum_egg_binary_day4 / sum_egg_binary), #what proportion lai
d eggs on day 4, out of ones that laid eggs?
                   proportion_binarylarvae = (sum_larvae_binary / sum_egg_binary_day3), #of ones that had e
ggs on day 3, what proportion had any # of larvae hatch?
                   #sample sizes
                   n_mosquitoes = n(), #total n
                   #standard errors
                   SE_eggsday3 = sd(Eggs_day3,na.rm = TRUE)/sqrt(n()),
                   SE_eggsday4 = sd(Eggs_day4,na.rm = TRUE)/sqrt(n()),
                   SE_totaleggs = sd(total_eggs,na.rm = TRUE)/sqrt(n()),
                   SE_percenteggs_day3 = sd(Percent_eggs_day3,na.rm = TRUE)/sqrt(n()),
                   SE_percenteggs_day4 = sd(Percent_eggs_day4,na.rm = TRUE)/sqrt(n()),
                   SE_larvae_day4 = sd(Larvae_day4,na.rm = TRUE)/sqrt(n()),
                   SE_percenteggshatched = sd(Percent_eggshatchedtolarv,na.rm = TRUE)/sqrt(n()))

Summary_Fecundity_data_ovipos_TEMP <- as.data.frame(Summary_Fecundity_data_ovipos_TEMP)
write_xlsx(Summary_Fecundity_data_ovipos_TEMP, "Oviposition_eggsandlarvae/Summary_Fecundity_data_ovipos_TEM
P.xlsx")

Summary_Fecundity_data_ovipos_proportions_means_TEMP <- Summary_Fecundity_data_ovipos_proportions %>% #Fecu
ndity_data_firstBM_ovipos %>%
  group_by(Temperature) %>%
  dplyr::summarise(#binary proportions:
```

```
    mean_proportion_binaryegg = mean(proportion_binaryegg,na.rm = TRUE), #what proportion laid any number o
f eggs? #remove NAs, but keep all mosquitoes bc some laid eggs even if dead on day 3 count
    mean_proportion_binaryegg_day3 = mean(proportion_binaryegg_day3,na.rm = TRUE), #what proportion laid eg
gs on day 3, out of ones that laid eggs?
    mean_proportion_binaryegg_day4 = mean(proportion_binaryegg_day4,na.rm = TRUE), #what proportion laid eg
gs on day 4, out of ones that laid eggs?
    mean_proportion_binarylarvae = mean(proportion_binarylarvae,na.rm = TRUE), #of ones that had eggs on da
y 3, what proportion had any # of larvae hatch?
    mean_proportion_surv_to_day3 = mean(proportion_surv_to_day3,na.rm = TRUE),
    mean_proportion_surv_to_day4 = mean(proportion_surv_to_day4,na.rm = TRUE),
    #sample sizes
    n_mosquitoes = sum(n_mosquitoes),
    n_trials = n(),
    SE_proportion_binaryegg = sd(proportion_binaryegg,na.rm = TRUE)/sqrt(n()),
    SE_proportion_binaryegg_day3 = sd(proportion_binaryegg_day3,na.rm = TRUE)/sqrt(n()),
    SE_proportion_binaryegg_day4 = sd(proportion_binaryegg_day4,na.rm = TRUE)/sqrt(n()),
    SE_proportion_binarylarvae = sd(proportion_binarylarvae,na.rm = TRUE)/sqrt(n()),
    SE_proportion_surv_to_day3 = sd(proportion_surv_to_day3,na.rm = TRUE)/sqrt(n()),
    SE_proportion_surv_to_day4 = sd(proportion_surv_to_day4,na.rm = TRUE)/sqrt(n()))


Summary_Fecundity_data_ovipos_proportions_means_TEMP <- as.data.frame(Summary_Fecundity_data_ovipos_proport
ions_means_TEMP)
write_xlsx(Summary_Fecundity_data_ovipos_proportions_means_TEMP, "Oviposition_eggsandlarvae/Summary_Fecundi
ty_data_ovipos_proportions_means_TEMP.xlsx")


#AGE only:
Summary_Fecundity_data_ovipos_AGE <- Fecundity_data_ovipos %>%
  group_by(Age) %>%
  dplyr::summarise(mean_eggsday3 = mean(Eggs_day3, na.rm = TRUE),
                   mean_eggsday4 = mean(Eggs_day4, na.rm = TRUE),
                   mean_totaleggs= mean(total_eggs, na.rm = TRUE),
                   mean_percenteggs_day3 = mean(Percent_eggs_day3, na.rm = TRUE), #% of total eggs laid on
day 3
                   mean_percenteggs_day4 = mean(Percent_eggs_day4, na.rm = TRUE),#% of total eggs laid on d
ay 4
                   mean_larvae_day4 = mean(Larvae_day4, na.rm = TRUE),
                   mean_Percent_eggshatchedtolarv = mean(Percent_eggshatchedtolarv, na.rm = TRUE), #of the
day3 eggs, what percent hatched?
                   #binary proportions:
                   sum_egg_binary = sum(egg_binary, na.rm = TRUE), #gives total number of mosquitoes per gr
oup that laid eggs
                   sum_surv_to_day3 = sum(`Surv_to_eggs(y/n)`=="Y"), #gives total number of mosquitoes per
group that survived to day 3 post BM
                   sum_surv_to_day4 = sum(`Surv_to_larvae(y/n)`=="Y"), #gives total number of mosquitoes pe
r group that survived to day 4 post BM
                   sum_egg_binary_day3 = sum(egg_binary_day3, na.rm = TRUE), #number of eggs laid on day 3
                   sum_egg_binary_day4 = sum(egg_binary_day4, na.rm = TRUE), #number of eggs laid on day 4
                   sum_larvae_binary = sum(larvae_binary, na.rm = TRUE), #number of larvae counted on day 4
                   proportion_binaryegg = (sum_egg_binary / n()), #what proportion laid any number of eggs?
#remove NAs, but keep all mosquitoes bc some laid eggs even if dead on day 3 count
                   proportion_binaryegg_day3 = (sum_egg_binary_day3 / sum_egg_binary), #what proportion lai
d eggs on day 3, out of ones that laid eggs?
                   proportion_binaryegg_day4 = (sum_egg_binary_day4 / sum_egg_binary), #what proportion lai
d eggs on day 4, out of ones that laid eggs?
```

```
                   proportion_binarylarvae = (sum_larvae_binary / sum_egg_binary_day3), #of ones that had e
ggs on day 3, what proportion had any # of larvae hatch?
                   #sample sizes
                   n_mosquitoes = n(), #total n
                   #standard errors
                   SE_eggsday3 = sd(Eggs_day3,na.rm = TRUE)/sqrt(n()),
                   SE_eggsday4 = sd(Eggs_day4,na.rm = TRUE)/sqrt(n()),
                   SE_totaleggs = sd(total_eggs,na.rm = TRUE)/sqrt(n()),
                   SE_percenteggs_day3 = sd(Percent_eggs_day3,na.rm = TRUE)/sqrt(n()),
                   SE_percenteggs_day4 = sd(Percent_eggs_day4,na.rm = TRUE)/sqrt(n()),
                   SE_larvae_day4 = sd(Larvae_day4,na.rm = TRUE)/sqrt(n()),
                   SE_percenteggshatched = sd(Percent_eggshatchedtolarv,na.rm = TRUE)/sqrt(n()))

Summary_Fecundity_data_ovipos_AGE <- as.data.frame(Summary_Fecundity_data_ovipos_AGE)
write_xlsx(Summary_Fecundity_data_ovipos_AGE, "Oviposition_eggsandlarvae/Summary_Fecundity_data_ovipos_AGE.
xlsx")


Summary_Fecundity_data_ovipos_proportions_means_AGE <- Summary_Fecundity_data_ovipos_proportions %>% #Fecun
dity_data_firstBM_ovipos %>%
  group_by(Age) %>%
  dplyr::summarise(#binary proportions:
    mean_proportion_binaryegg = mean(proportion_binaryegg,na.rm = TRUE), #what proportion laid any number o
f eggs? #remove NAs, but keep all mosquitoes bc some laid eggs even if dead on day 3 count
    mean_proportion_binaryegg_day3 = mean(proportion_binaryegg_day3,na.rm = TRUE), #what proportion laid eg
gs on day 3, out of ones that laid eggs?
    mean_proportion_binaryegg_day4 = mean(proportion_binaryegg_day4,na.rm = TRUE), #what proportion laid eg
gs on day 4, out of ones that laid eggs?
    mean_proportion_binarylarvae = mean(proportion_binarylarvae,na.rm = TRUE), #of ones that had eggs on da
y 3, what proportion had any # of larvae hatch?
    mean_proportion_surv_to_day3 = mean(proportion_surv_to_day3,na.rm = TRUE),
    mean_proportion_surv_to_day4 = mean(proportion_surv_to_day4,na.rm = TRUE),
    #sample sizes
    n_mosquitoes = sum(n_mosquitoes),
    n_trials = n(),
    SE_proportion_binaryegg = sd(proportion_binaryegg,na.rm = TRUE)/sqrt(n()),
    SE_proportion_binaryegg_day3 = sd(proportion_binaryegg_day3,na.rm = TRUE)/sqrt(n()),
    SE_proportion_binaryegg_day4 = sd(proportion_binaryegg_day4,na.rm = TRUE)/sqrt(n()),
    SE_proportion_binarylarvae = sd(proportion_binarylarvae,na.rm = TRUE)/sqrt(n()),
    SE_proportion_surv_to_day3 = sd(proportion_surv_to_day3,na.rm = TRUE)/sqrt(n()),
    SE_proportion_surv_to_day4 = sd(proportion_surv_to_day4,na.rm = TRUE)/sqrt(n()))


Summary_Fecundity_data_ovipos_proportions_means_AGE <- as.data.frame(Summary_Fecundity_data_ovipos_proporti
ons_means_AGE)
write_xlsx(Summary_Fecundity_data_ovipos_proportions_means_AGE, "Oviposition_eggsandlarvae/Summary_Fecundit
y_data_ovipos_proportions_means_AGE.xlsx")
```

Plot total eggs laid

```
#separate for plotting:
Fecundity_data_firstBM_ovipos <- Fecundity_data_ovipos
Summary_Fecundity_data_firstBM_ovipos <- Summary_Fecundity_data_ovipos
Summary_Fecundity_data_firstBM_ovipos_proportions <- Summary_Fecundity_data_ovipos_proportions
Summary_Fecundity_data_firstBM_ovipos_proportions_means <- Summary_Fecundity_data_ovipos_proportions_means
Summary_Fecundity_data_firstBM_ovipos_AGE <- Summary_Fecundity_data_ovipos_AGE
Summary_Fecundity_data_firstBM_ovipos_proportions_means_AGE <- Summary_Fecundity_data_ovipos_proportions_me
ans_AGE
Summary_Fecundity_data_firstBM_ovipos_TEMP <- Summary_Fecundity_data_ovipos_TEMP
Summary_Fecundity_data_firstBM_ovipos_proportions_means_TEMP <-Summary_Fecundity_data_ovipos_proportions_me
ans_TEMP



#total eggs
Summary_Fecundity_data_firstBM_ovipos$Age <- factor(Summary_Fecundity_data_firstBM_ovipos$Age,
                                                    labels = c("3 days","5 days","10 days","15 days"))

Summary_Fecundity_data_firstBM_ovipos$Temperature <- factor(Summary_Fecundity_data_firstBM_ovipos$Temperatu
re,
                                                            labels = c("27","30","32"))

Fecundity_data_firstBM_ovipos$Age <- factor(Fecundity_data_firstBM_ovipos$Age,
                                            labels = c("3 days","5 days","10 days","15 days"))

Fecundity_data_firstBM_ovipos$Temperature <- factor(Fecundity_data_firstBM_ovipos$Temperature,
                                                    labels = c("27","30","32"))

firstBM_ovipos_totaleggs_tempwithinage <- Summary_Fecundity_data_firstBM_ovipos %>%
  ggplot(aes(x=Temperature,y=mean_totaleggs,group=Temperature))+
  geom_bar(aes(fill=Temperature),
           stat = "identity",
           position = position_dodge(1),
           width = 0.8) +
  scale_shape_identity(guide="legend")+
  facet_grid(~Age)+
  geom_errorbar(aes(ymin=(mean_totaleggs - SE_totaleggs),
                    ymax=(mean_totaleggs + SE_totaleggs)),
                width=0.8,position=position_dodge(0.9),
                color="black")+
  ylab(expression("Total number of eggs laid"))+
  xlab("Temperature (˚C)") +
  theme_pubr()+
  theme(legend.position = "none")+
  geom_jitter(data=Fecundity_data_firstBM_ovipos, aes(x=Temperature,y=total_eggs),#color=ZOI_italic$Technic
al_Rep,
              position = "jitter", na.rm = TRUE,size=0.5)+
  scale_fill_manual(values= c("#4D6FAE","#6F9F51", "#CC763B"))+
  theme(panel.background = element_rect(fill = NA, color = "black"))+
  theme(panel.spacing = unit(0.5, "lines"))
firstBM_ovipos_totaleggs_tempwithinage
```

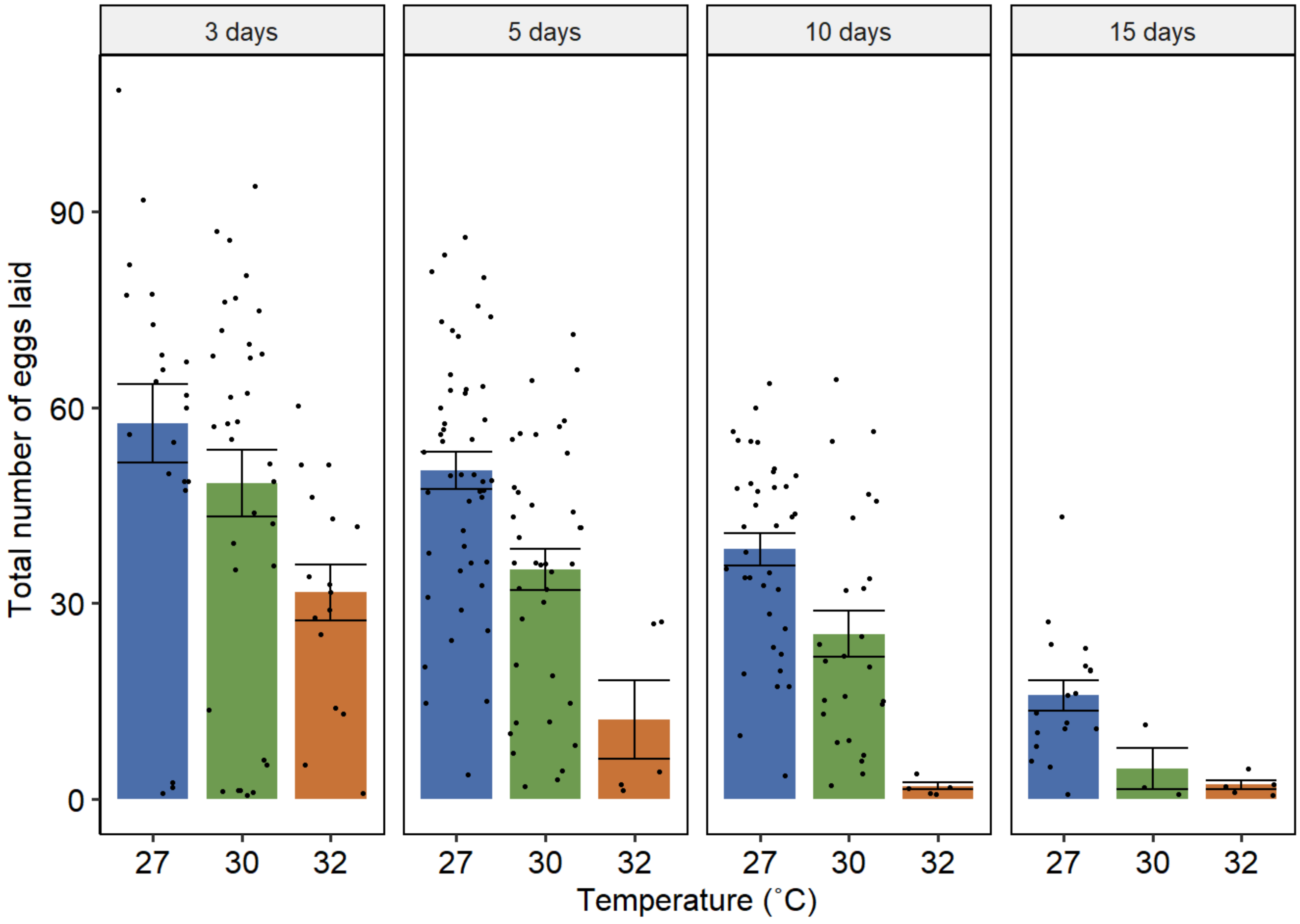

3 days
5 days
10 days
15 days
Total number of eggs laid
0
30
60
90
27
30
32
Temperature (°C)

```
ggsave("Oviposition_eggsandlarvae/firstBM_ovipos_totaleggs_tempwithinage.pdf", plot=firstBM_ovipos_totalegg
s_tempwithinage, width = 6, height = 4, units = "in",dpi=600)
ggsave("Oviposition_eggsandlarvae/firstBM_ovipos_totaleggs_tempwithinage.png", plot=firstBM_ovipos_totalegg
s_tempwithinage, width = 6, height = 4, units = "in",dpi=600)

Summary_Fecundity_data_firstBM_ovipos$Age <- factor(Summary_Fecundity_data_firstBM_ovipos$Age,
                                                    labels = c("3","5","10","15"))
Summary_Fecundity_data_firstBM_ovipos$Temperature <- factor(Summary_Fecundity_data_firstBM_ovipos$Temperatu
re,
                                                            labels = c("27˚C","30˚C","32˚C"))

Fecundity_data_firstBM_ovipos$Age <- factor(Fecundity_data_firstBM_ovipos$Age,
                                            labels = c("3","5","10","15"))
Fecundity_data_firstBM_ovipos$Temperature <- factor(Fecundity_data_firstBM_ovipos$Temperature,
                                                    labels = c("27˚C","30˚C","32˚C"))

firstBM_ovipos_totaleggs_agewithintemp <- Summary_Fecundity_data_firstBM_ovipos %>%
  ggplot(aes(x=Age,y=mean_totaleggs,group=Age))+
  geom_bar(aes(fill=Age),
           stat = "identity",
           position = position_dodge(1),
           width = 0.8) +
  scale_shape_identity(guide="legend")+
  facet_grid(~Temperature)+
  geom_errorbar(aes(ymin=(mean_totaleggs - SE_totaleggs),
                    ymax=(mean_totaleggs + SE_totaleggs)),
                width=0.8,position=position_dodge(0.9),
                color="black")+
  ylab(expression("Total number of eggs laid"))+
  xlab("Age of first BM (days old)") +
  theme_pubr()+
  theme(legend.position = "none")+
  geom_jitter(data=Fecundity_data_firstBM_ovipos, aes(x=Age,y=total_eggs),#color=ZOI_italic$Technical_Rep,
              position = "jitter", na.rm = TRUE,size=0.5)+
  scale_fill_manual(values= c("#DCD1E9","#BAA4D3","#9776BE","#7549A8"))+
  theme(panel.background = element_rect(fill = NA, color = "black"))+
  theme(panel.spacing = unit(0.5, "lines"))

firstBM_ovipos_totaleggs_agewithintemp
```

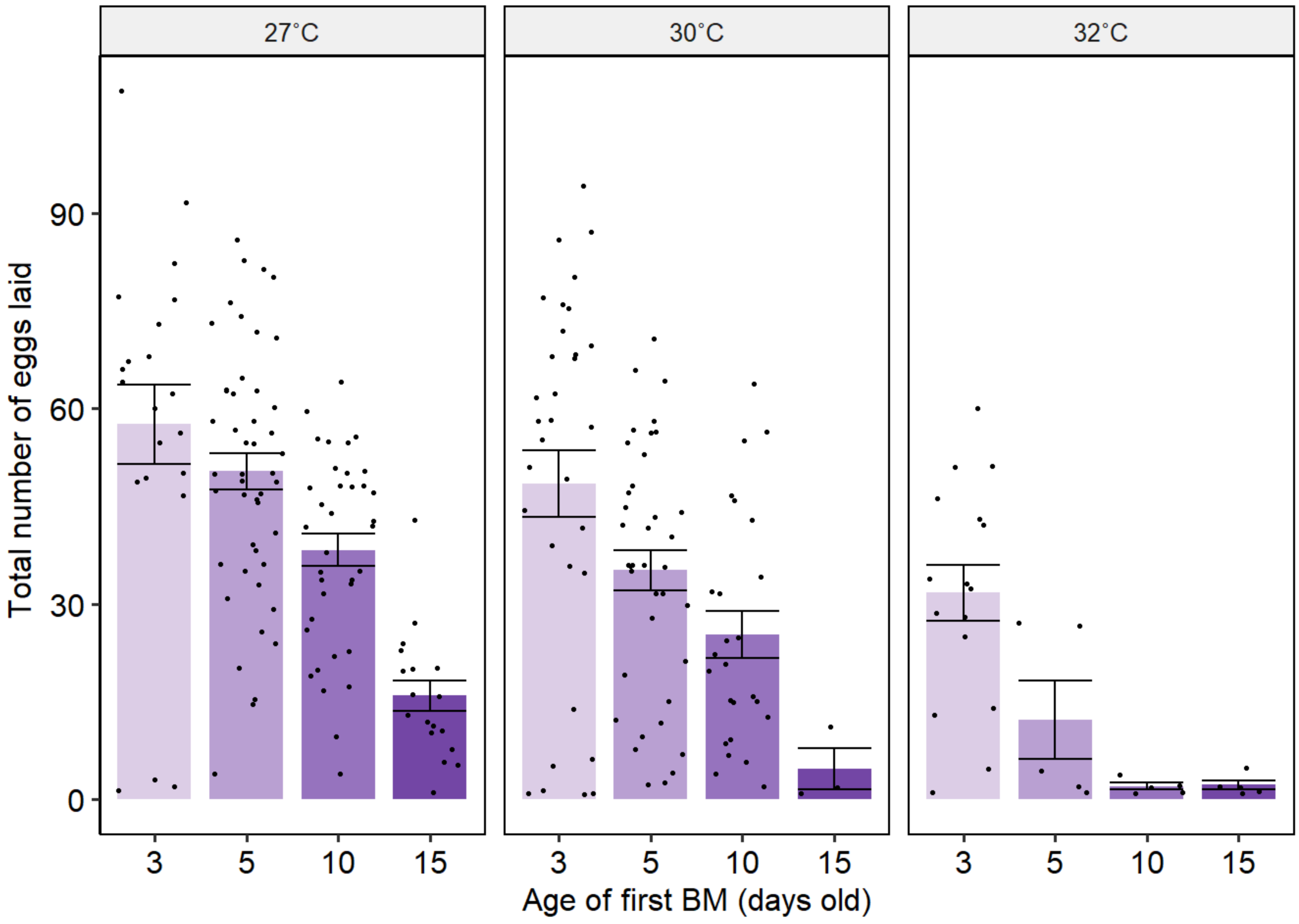


```
ggsave("Oviposition_eggsandlarvae/firstBM_ovipos_totaleggs_agewithintemp.pdf", plot=firstBM_ovipos_totalegg
s_agewithintemp, width = 6, height = 4, units = "in",dpi=600)
ggsave("Oviposition_eggsandlarvae/firstBM_ovipos_totaleggs_agewithintemp.png", plot=firstBM_ovipos_totalegg
s_agewithintemp, width = 6, height = 4, units = "in",dpi=600)


#raw data interaction plot:
BM1_ovipos_proportion_totaleggs_rawdata_interaction <- Summary_Fecundity_data_firstBM_ovipos%>%
  ggplot()+
  aes(x=Age,y=mean_totaleggs,group=Temperature)+
  geom_point(aes(shape=Temperature),size=3)+
  geom_line(aes(linetype = Temperature),linewidth=0.6)+
  theme_pubr()+
  scale_shape(labels=c(27,30,32))+
  scale_linetype(labels=c(27,30,32))+
  guides(shape = guide_legend(title = "Temperature (˚C)"),
         linetype = guide_legend(title = "Temperature (˚C)"))+
  xlab("Adult Age (days)") +
  theme(legend.position = "right")+
  scale_x_discrete(labels=c(3,5,10,15))+
  ylab(expression("Total number of eggs laid"))+
  theme(panel.background = element_rect(fill = NA, color = "black"))+
  theme(panel.spacing = unit(0.6, "lines"))
BM1_ovipos_proportion_totaleggs_rawdata_interaction
```

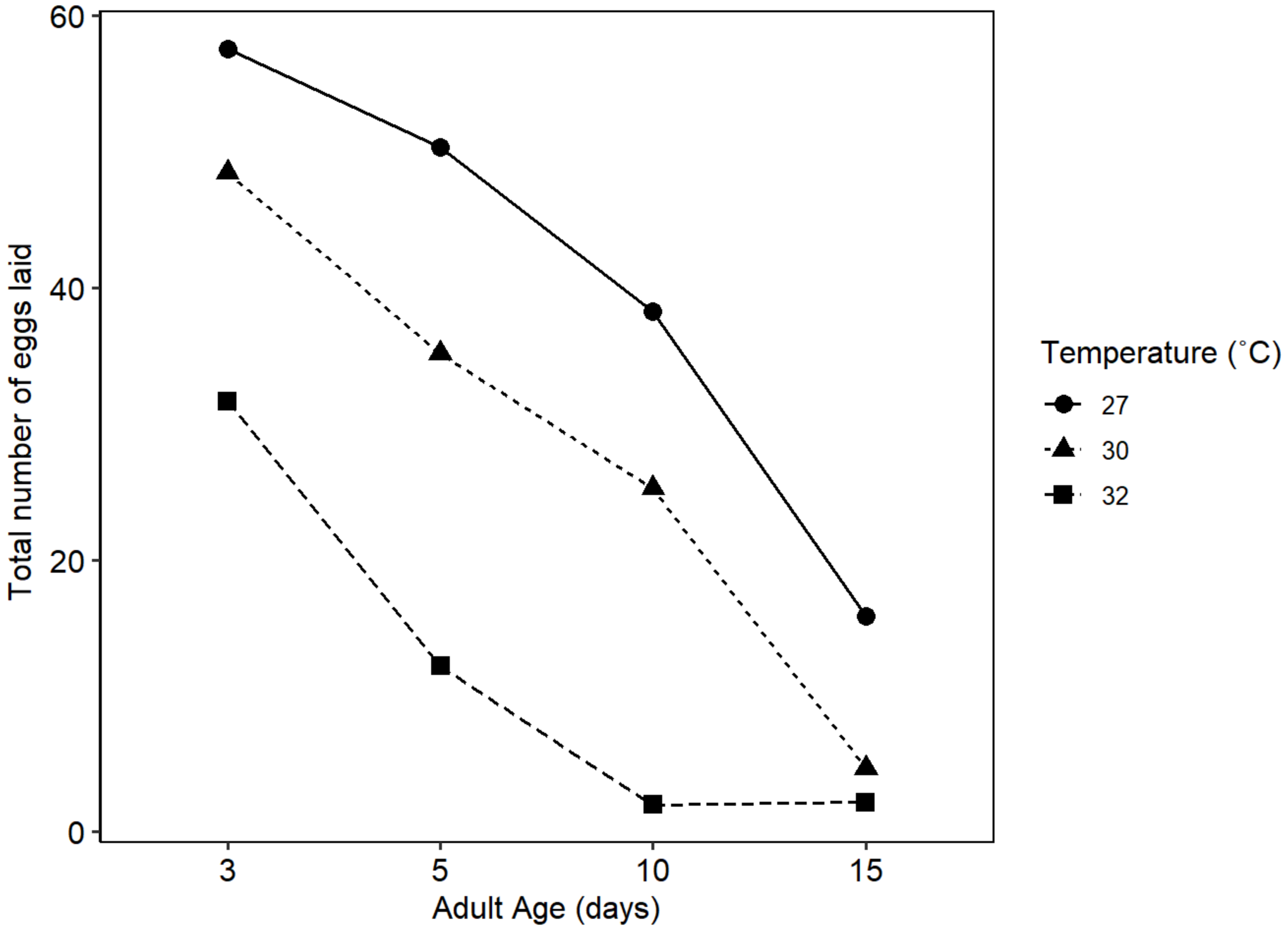

Total number of eggs laid
60
40
20
0
3
5
10
15
Adult Age (days)
Temperature (˚C)
27
30
32

```
ggsave("Oviposition_eggsandlarvae/BM1_ovipos_proportion_totaleggs_rawdata_interaction.png",plot=BM1_ovipos_
proportion_totaleggs_rawdata_interaction,width = 5.5, height = 4, units = "in", dpi = 600)
ggsave("Oviposition_eggsandlarvae/BM1_ovipos_proportion_totaleggs_rawdata_interaction.pdf",plot=BM1_ovipos_
proportion_totaleggs_rawdata_interaction,width = 5.5, height = 4, units = "in", dpi = 600)




#TEMP ONLY
Summary_Fecundity_data_firstBM_ovipos_TEMP$Temperature <- factor(Summary_Fecundity_data_firstBM_ovipos_TEMP
$Temperature,
                                                                 labels = c("27","30","32"))
Fecundity_data_firstBM_ovipos$Temperature <- factor(Fecundity_data_firstBM_ovipos$Temperature,
                                                    labels = c("27","30","32"))

firstBM_ovipos_totaleggs_TEMPonly <- Summary_Fecundity_data_firstBM_ovipos_TEMP %>%
  ggplot(aes(x=Temperature,y=mean_totaleggs,group=Temperature))+
  geom_bar(aes(fill=Temperature),
           stat = "identity",
           position = position_dodge(1),
           width = 0.8) +
  scale_shape_identity(guide="legend")+
  #facet_grid(~Age)+
  geom_errorbar(aes(ymin=(mean_totaleggs - SE_totaleggs),
                    ymax=(mean_totaleggs + SE_totaleggs)),
                width=0.8,position=position_dodge(0.9),
                color="black")+
  ylab(expression("Total number of eggs laid"))+
  xlab("Temperature (˚C)") +
  theme_pubr()+
  theme(legend.position = "none")+
  geom_jitter(data=Fecundity_data_firstBM_ovipos, aes(x=Temperature,y=total_eggs),#color=ZOI_italic$Technic
al_Rep,
              position = "jitter", na.rm = TRUE,size=0.5)+
  scale_fill_manual(values= c("#4D6FAE","#6F9F51", "#CC763B"))+
  theme(panel.background = element_rect(fill = NA, color = "black"))+
  theme(panel.spacing = unit(0.5, "lines"))
firstBM_ovipos_totaleggs_TEMPonly
```

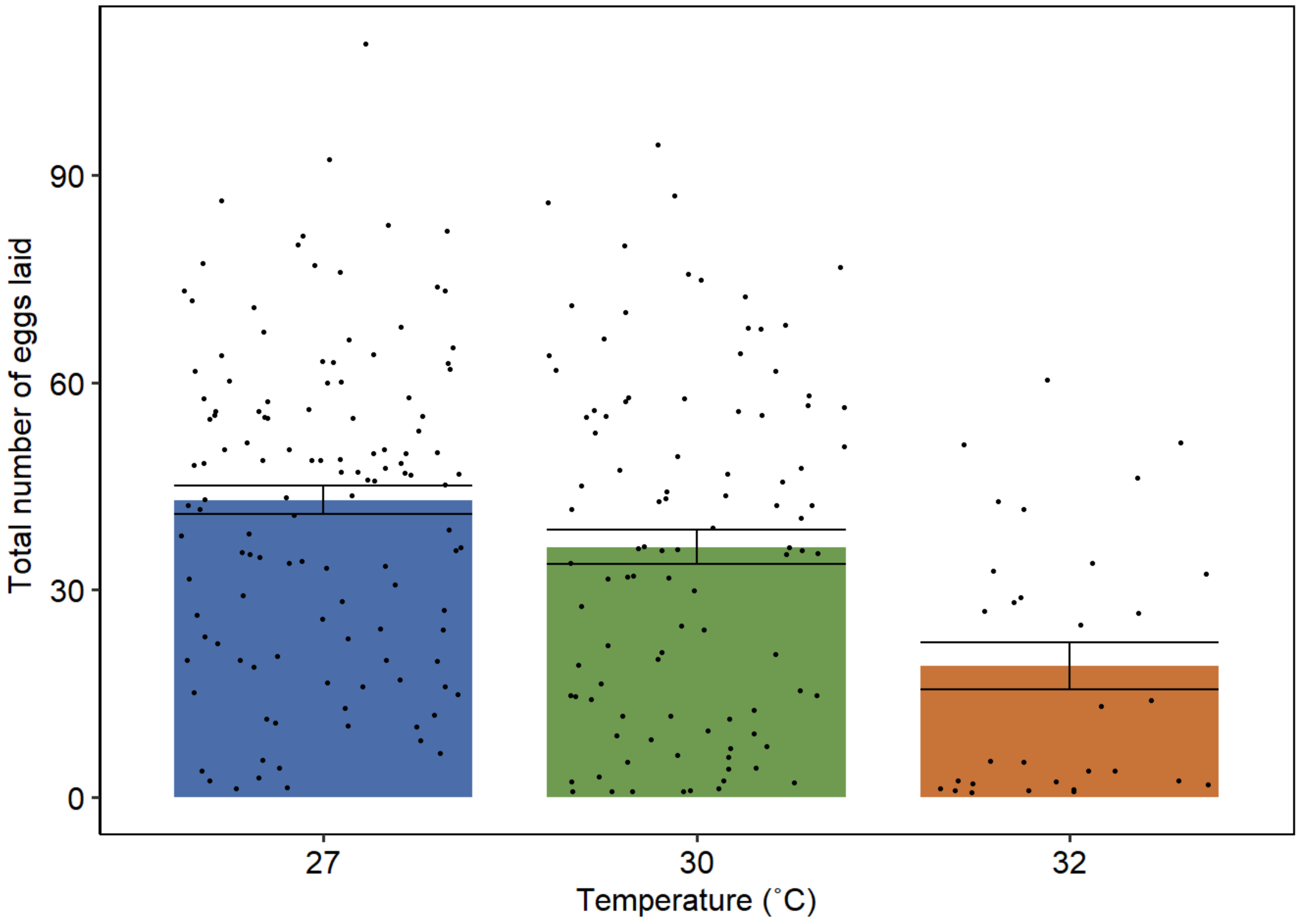

Total number of eggs laid
90
60
30
0
27
30
32
Temperature (˚C)

```
ggsave("Oviposition_eggsandlarvae/firstBM_ovipos_totaleggs_TEMPonly.pdf", plot=firstBM_ovipos_totaleggs_TEMPonly, width = 4, height = 4, units = "in",dpi=600)
ggsave("Oviposition_eggsandlarvae/firstBM_ovipos_totaleggs_TEMPonly.png", plot=firstBM_ovipos_totaleggs_TEMPonly, width = 4, height = 4, units = "in",dpi=600)

#AGE ONLY
Summary_Fecundity_data_firstBM_ovipos_AGE$Age <- factor(Summary_Fecundity_data_firstBM_ovipos_AGE$Age,
                                                        labels = c("3","5","10","15"))
Fecundity_data_firstBM_ovipos$Age <- factor(Fecundity_data_firstBM_ovipos$Age,
                                            labels = c("3","5","10","15"))

firstBM_ovipos_totaleggs_AGEonly <- Summary_Fecundity_data_firstBM_ovipos_AGE %>%
  ggplot(aes(x=Age,y=mean_totaleggs,group=Age))+
  geom_bar(aes(fill=Age),
           stat = "identity",
           position = position_dodge(1),
           width = 0.8) +
  scale_shape_identity(guide="legend")+
  #facet_grid(~Temperature)+
  geom_errorbar(aes(ymin=(mean_totaleggs - SE_totaleggs),
                    ymax=(mean_totaleggs + SE_totaleggs)),
                width=0.8,position=position_dodge(0.9),
                color="black")+
  ylab(expression("Total number of eggs laid"))+
  xlab("Age of first BM (days old)") +
  theme_pubr()+
  theme(legend.position = "none")+
  geom_jitter(data=Fecundity_data_firstBM_ovipos, aes(x=Age,y=total_eggs),#color=ZOI_italic$Technical_Rep,
              position = "jitter", na.rm = TRUE,size=0.5)+
  scale_fill_manual(values= c("#DCD1E9","#BAA4D3","#9776BE","#7549A8"))+
  theme(panel.background = element_rect(fill = NA, color = "black"))+
  theme(panel.spacing = unit(0.5, "lines"))

firstBM_ovipos_totaleggs_AGEonly
```

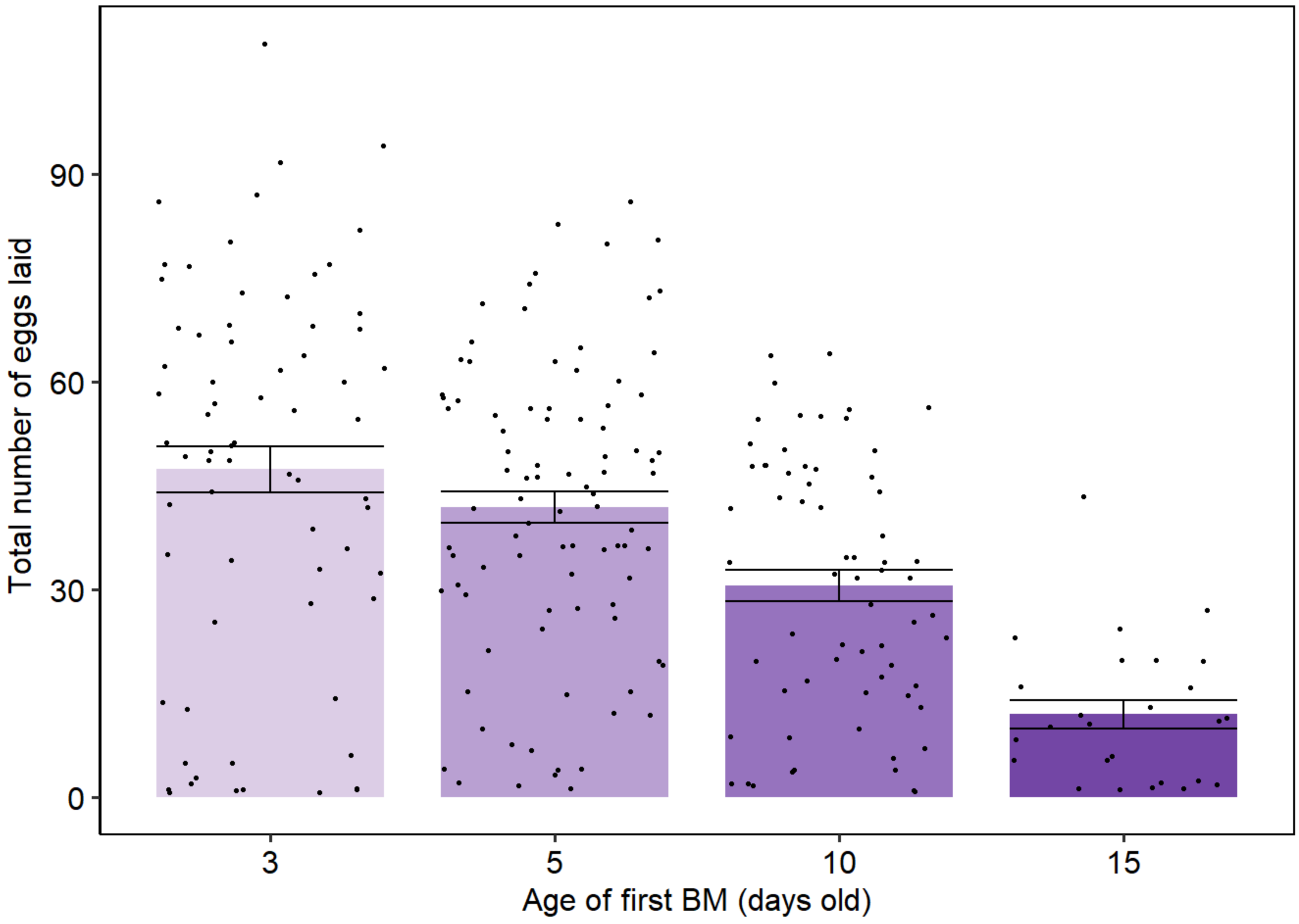


```
ggsave("Oviposition_eggsandlarvae/firstBM_ovipos_totaleggs_AGEonly.pdf", plot=firstBM_ovipos_totaleggs_AGEonly, width = 4, height = 4, units = "in",dpi=600)
ggsave("Oviposition_eggsandlarvae/firstBM_ovipos_totaleggs_AGEonly.png", plot=firstBM_ovipos_totaleggs_AGEonly, width = 4, height = 4, units = "in",dpi=600)
```

Plot distribution of eggs laid days 3 vs 4

```
#distribution plot to show number eggs day 3 and day 4 out of total
#on same graph
eggdaysummary <- Fecundity_data_firstBM_ovipos %>%
  group_by(Temperature,Age) %>%
  reframe(n_day3 = mean(Eggs_day3),
          P_day3 = mean(Percent_eggs_day3),
          n_day4 = mean(Eggs_day4),
          P_day4 = mean(Percent_eggs_day4),
          n_3and4total = mean(total_eggs),
          n_mosquitoes = n())

write_xlsx(eggdaysummary, "Oviposition_eggsandlarvae/egg_timing_proportion_summary.xlsx")


eggdaysummary <- eggdaysummary %>%
  pivot_longer(cols=c("P_day3","P_day4"),names_to="day")

######
eggdaysummary$Age <- factor(eggdaysummary$Age,
                            labels = c("3","5","10","15"))

eggdaysummary$Temperature <- factor(eggdaysummary$Temperature,
                                    labels = c("27˚C","30˚C","32˚C"))



proportioneggslaidgraph1 <- eggdaysummary %>%
  ggplot(aes(fill=day,y=value, x= Age))+
  geom_bar(position=position_fill(reverse = TRUE),stat="identity")+ #stacked + percentages
  facet_grid(~Temperature)+
  theme_pubr()+
  theme(legend.position="right")+
  ylab(expression("Percentage of eggs laid each day"))+
  xlab("Age of first BM (days old)") +
  scale_y_continuous(labels = function(x) paste0(x*100)) +
  theme(panel.background = element_rect(fill = NA, color = "black"))+
  theme(panel.spacing = unit(0.5, "lines"))+
  theme(text = element_text(size=12),
        axis.text.x = element_text(size=(10)),
        axis.text.y=element_text(size=10))+
  scale_fill_brewer(palette="Paired",
                    name = "Day Eggs Laid", labels = c("3d PBM", "4d PBM"))
proportioneggslaidgraph1
```

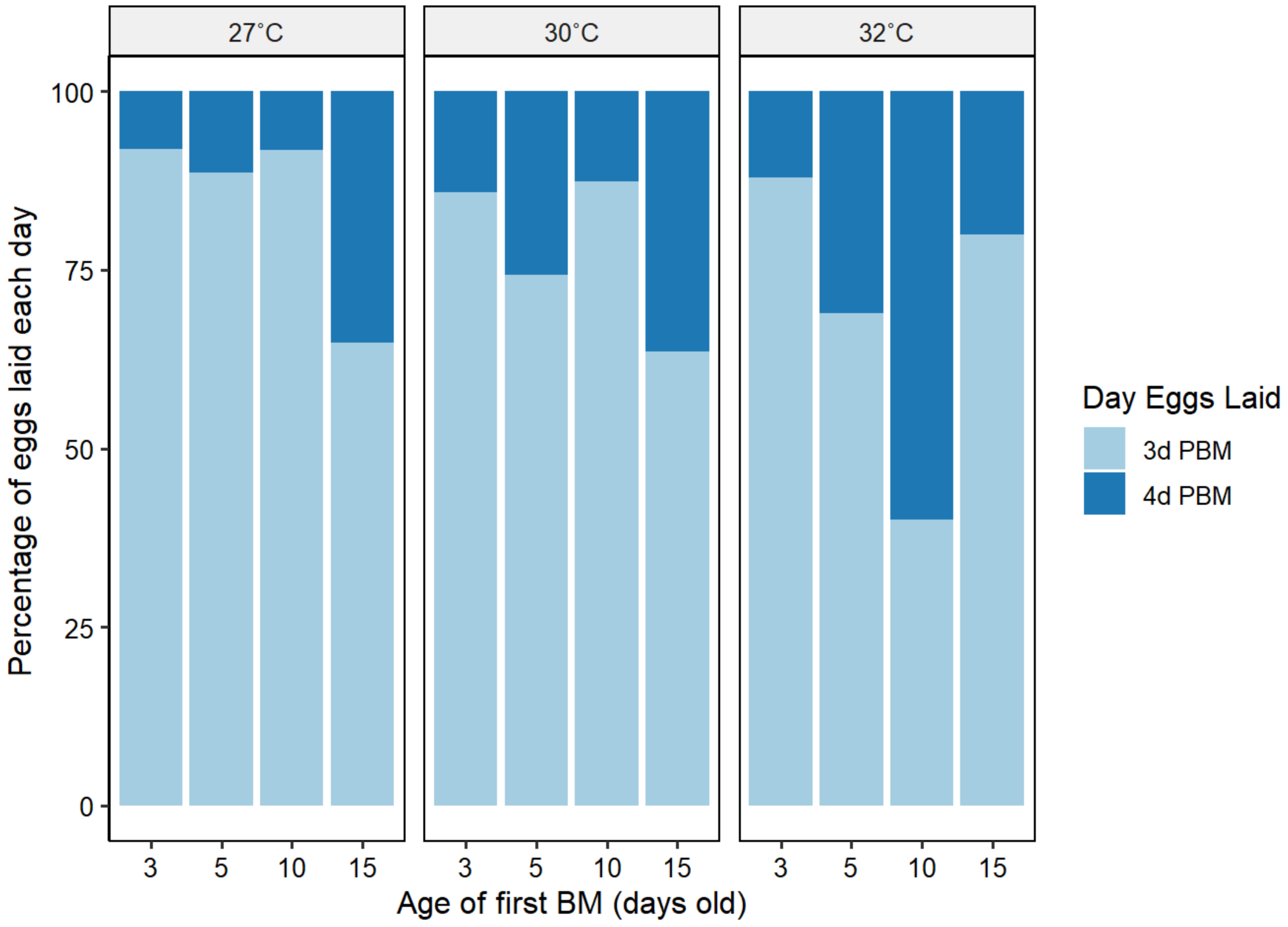

27°C
30°C
32°C
Percentage of eggs laid each day
100
75
50
25
0
3
5
10
15
Age of first BM (days old)
Day Eggs Laid
3d PBM
4d PBM

```
ggsave("Oviposition_eggsandlarvae/firstBM_proportioneggslaidgraph1.pdf", plot=proportioneggslaidgraph1, wid
th = 6, height = 4,
       units = "in",dpi=600)
ggsave("Oviposition_eggsandlarvae/firstBM_proportioneggslaidgraph1.png", plot=proportioneggslaidgraph1, wid
th = 6, height = 4,
       units = "in",dpi=600)



eggdaysummary$Age <- factor(eggdaysummary$Age,
                            labels = c("3 days","5 days","10 days","15 days"))

eggdaysummary$Temperature <- factor(eggdaysummary$Temperature,
                                    labels = c("27","30","32"))

proportioneggslaidgraph2 <- eggdaysummary %>%
  ggplot(aes(fill=day,y=value, x= Temperature))+
  geom_bar(position=position_fill(reverse = TRUE),stat="identity")+ #stacked + percentages
  facet_grid(~Age)+
  theme_pubr()+
  theme(legend.position="right")+
  ylab(expression("Percentage of eggs laid each day"))+
  xlab("Temperature (˚C)") +
  scale_y_continuous(labels = function(x) paste0(x*100)) +
  theme(panel.background = element_rect(fill = NA, color = "black"))+
  theme(panel.spacing = unit(0.5, "lines"))+
  theme(text = element_text(size=12),
        axis.text.x = element_text(size=(10)),
        axis.text.y=element_text(size=10))+
  scale_fill_brewer(palette="Paired",
                    name = "Day Eggs Laid", labels = c("3d PBM", "4d PBM"))
proportioneggslaidgraph2
```

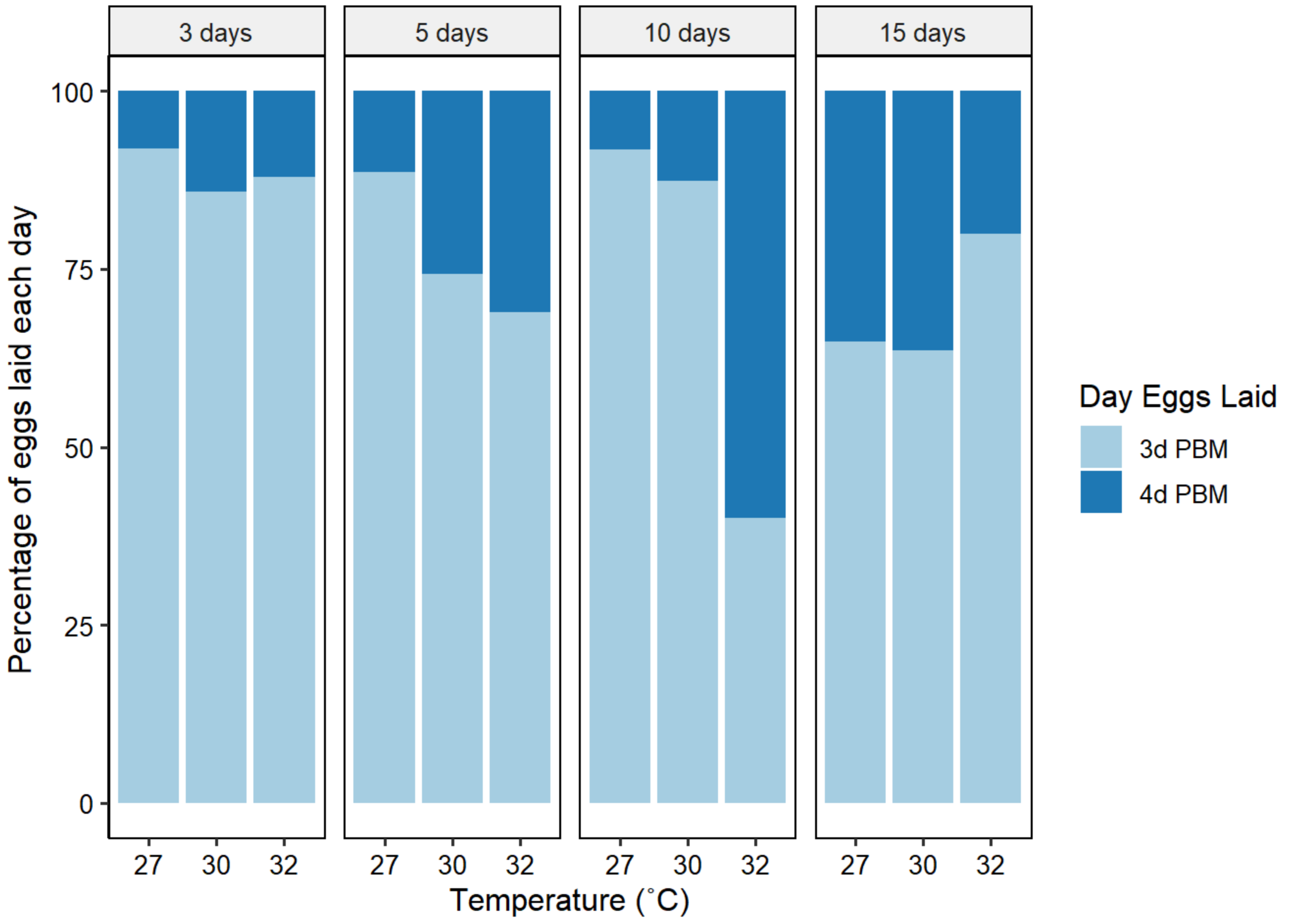
3 days
5 days
10 days
15 days
100
75
50
25
0
Percentage of eggs laid each day
27
30
32
Temperature (˚C)
Day Eggs Laid
3d PBM
4d PBM

```
ggsave("Oviposition_eggsandlarvae/firstBM_proportioneggslaidgraph2.pdf", plot=proportioneggslaidgraph2, wid
th = 6, height = 4,
       units = "in",dpi=600)
ggsave("Oviposition_eggsandlarvae/firstBM_proportioneggslaidgraph2.png", plot=proportioneggslaidgraph2, wid
th = 6, height = 4,
       units = "in",dpi=600)

#plot by main effects:
eggdaysummary_Temp <- Fecundity_data_firstBM_ovipos %>%
  group_by(Temperature) %>%
  reframe(n_day3 = mean(Eggs_day3),
          P_day3 = mean(Percent_eggs_day3),
          n_day4 = mean(Eggs_day4),
          P_day4 = mean(Percent_eggs_day4),
          n_3and4total = mean(total_eggs),
          n_mosquitoes = n())

write_xlsx(eggdaysummary_Temp, "Oviposition_eggsandlarvae/egg_timing_proportion_summary_TEMP.xlsx")

eggdaysummary_Temp <- eggdaysummary_Temp %>%
  pivot_longer(cols=c("P_day3","P_day4"),names_to="day")


eggdaysummary_Age <- Fecundity_data_firstBM_ovipos %>%
  group_by(Age) %>%
  reframe(n_day3 = mean(Eggs_day3),
          P_day3 = mean(Percent_eggs_day3),
          n_day4 = mean(Eggs_day4),
          P_day4 = mean(Percent_eggs_day4),
          n_3and4total = mean(total_eggs),
          n_mosquitoes = n())

write_xlsx(eggdaysummary_Age, "Oviposition_eggsandlarvae/egg_timing_proportion_summary_AGE.xlsx")

eggdaysummary_Age <- eggdaysummary_Age %>%
  pivot_longer(cols=c("P_day3","P_day4"),names_to="day")
###
eggdaysummary_Temp$Temperature <- factor(eggdaysummary_Temp$Temperature,
                                         labels = c("27","30","32"))

proportioneggslaidgraph_temp <- eggdaysummary_Temp %>%
  ggplot(aes(fill=day,y=value, x= Temperature))+
  geom_bar(position=position_fill(reverse = TRUE),stat="identity")+ #stacked + percentages
  #facet_grid(~Age)+
  theme_pubr()+
  theme(legend.position="right")+
  ylab(expression("Percentage of eggs laid each day"))+
  xlab("Temperature (˚C)") +
  scale_y_continuous(labels = function(x) paste0(x*100)) +
  theme(panel.background = element_rect(fill = NA, color = "black"))+
  theme(panel.spacing = unit(0.5, "lines"))+
  theme(text = element_text(size=12),
        axis.text.x = element_text(size=(10)),
        axis.text.y=element_text(size=10))+
  scale_fill_brewer(palette="Paired",
```

```
                    name = "Day Eggs Laid", labels = c("3d PBM", "4d PBM"))
proportioneggslaidgraph_temp
```

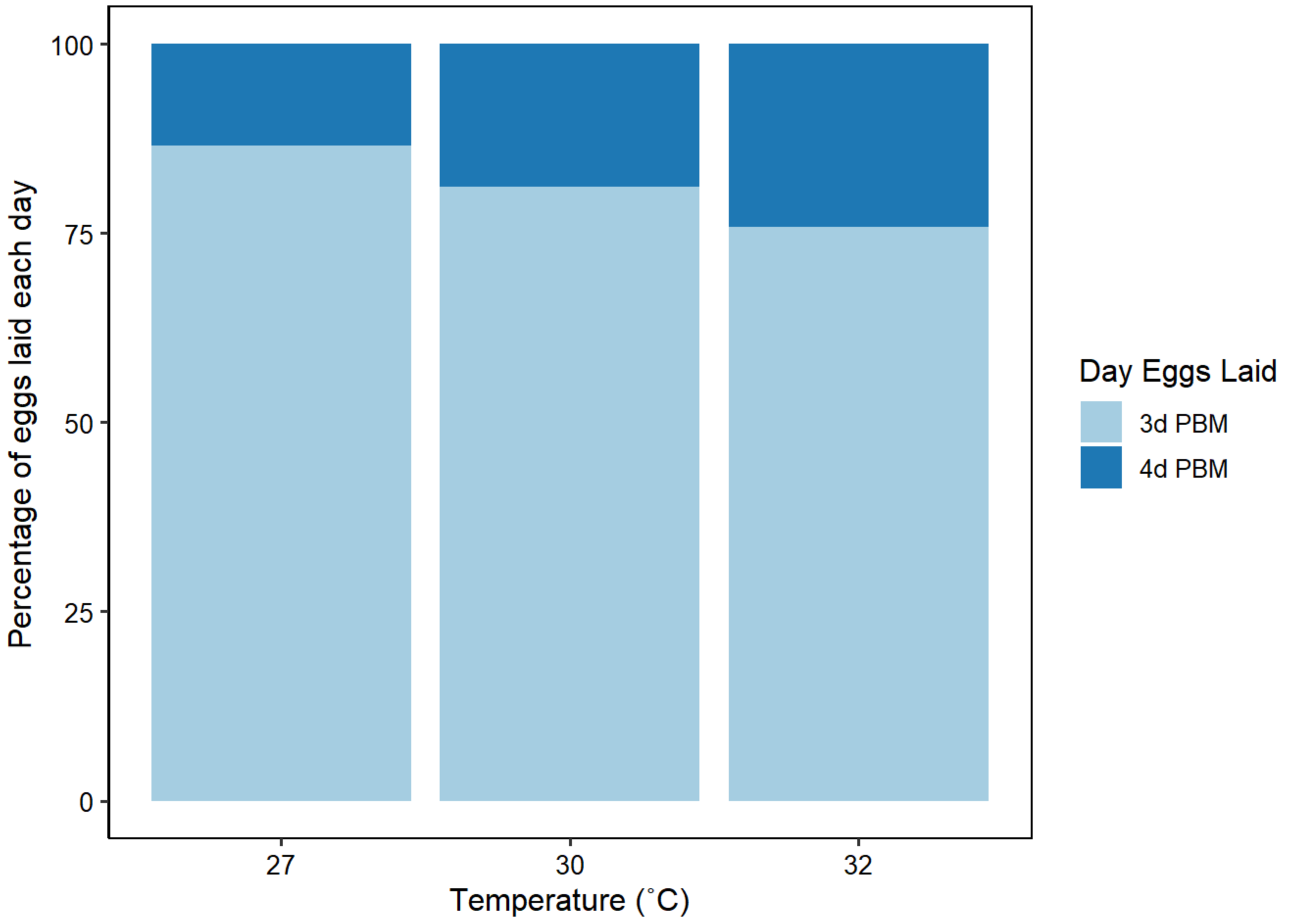

```
ggsave("Oviposition_eggsandlarvae/proportioneggslaidgraph_temp.pdf", plot=proportioneggslaidgraph_temp, wid
th = 5, height = 4,
       units = "in",dpi=600)
ggsave("Oviposition_eggsandlarvae/proportioneggslaidgraph_temp.png", plot=proportioneggslaidgraph_temp, wid
th = 5, height = 4,
       units = "in",dpi=600)

eggdaysummary_Age$Age <- factor(eggdaysummary_Age$Age,
                                labels = c("3","5","10","15"))

proportioneggslaidgraph_age <- eggdaysummary_Age %>%
  ggplot(aes(fill=day,y=value, x= Age))+
  geom_bar(position=position_fill(reverse = TRUE),stat="identity")+ #stacked + percentages
  #facet_grid(~Temperature)+
  theme_pubr()+
  theme(legend.position="right")+
  ylab(expression("Percentage of eggs laid each day"))+
  xlab("Age of first BM (days old)") +
  scale_y_continuous(labels = function(x) paste0(x*100)) +
  theme(panel.background = element_rect(fill = NA, color = "black"))+
  theme(panel.spacing = unit(0.5, "lines"))+
  theme(text = element_text(size=12),
        axis.text.x = element_text(size=(10)),
        axis.text.y=element_text(size=10))+
  scale_fill_brewer(palette="Paired",
                    name = "Day Eggs Laid", labels = c("3d PBM", "4d PBM"))
proportioneggslaidgraph_age
```

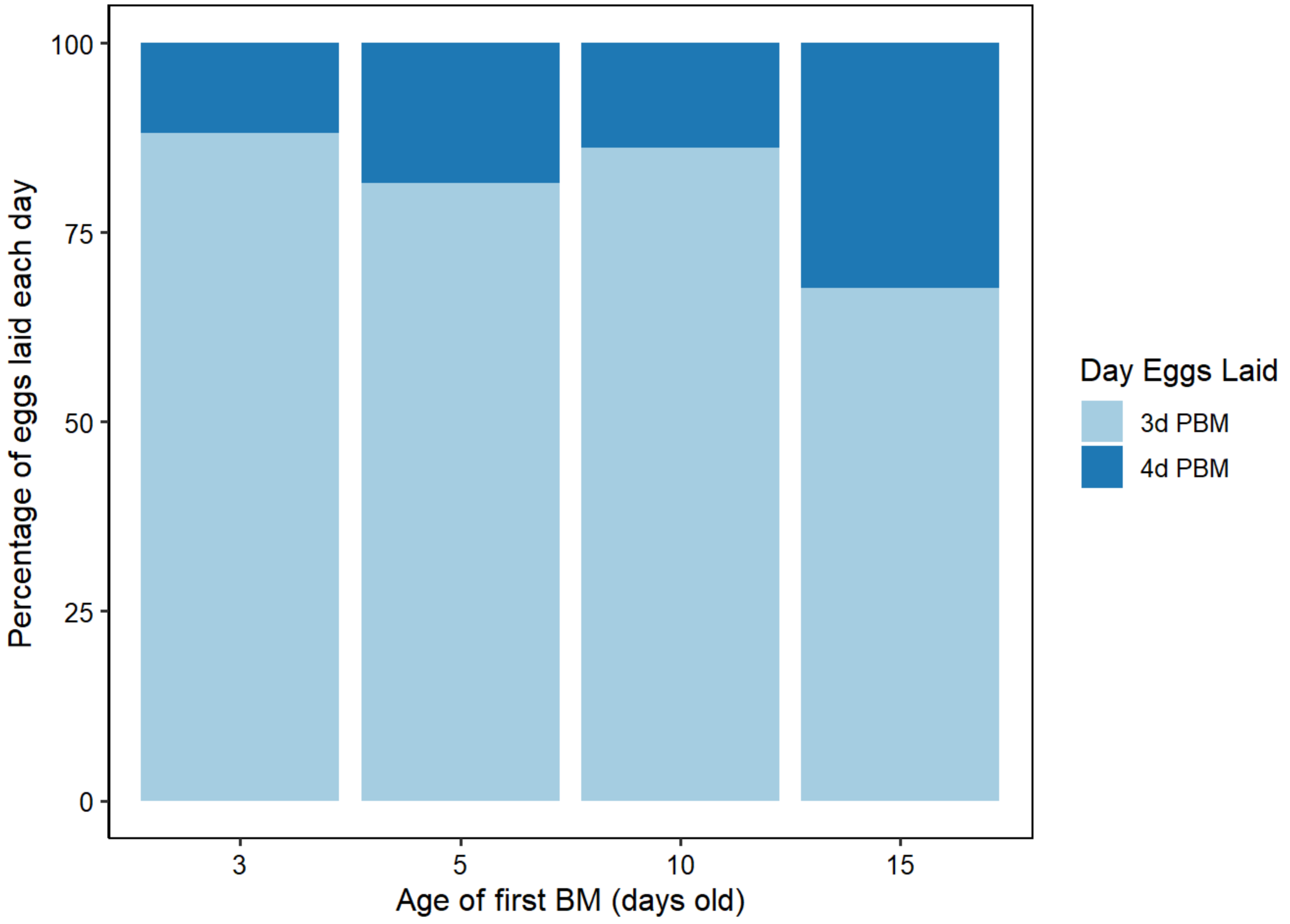

```
ggsave("Oviposition_eggsandlarvae/proportioneggslaidgraph_age.pdf", plot=proportioneggslaidgraph_age, width
= 5, height = 4,
       units = "in",dpi=600)
ggsave("Oviposition_eggsandlarvae/proportioneggslaidgraph_age.png", plot=proportioneggslaidgraph_age, width
= 5, height = 4,
       units = "in",dpi=600)
```

Analysis for total eggs

```
#analysis for total eggs

Fecundity_data_firstBM_ovipos_narm <- Fecundity_data_firstBM_ovipos %>%
  drop_na(total_eggs)
str(Fecundity_data_firstBM_ovipos_narm)
```

```
## 'data.frame':    252 obs. of  33 variables:
##  $ ID_overall               : num  6 12 15 18 38 41 42 43 46 47 ...
##  $ Temperature              : Factor w/ 3 levels "27","30","32": 3 1 1 1 2 2 2 2 2 2 ...
##  $ Age                      : Factor w/ 4 levels "3","5","10","15": 1 1 1 1 1 1 1 1 1 1 ...
##  $ ID_per_group             : num  6 2 5 8 3 6 7 8 11 12 ...
##  $ Trial_start_date         : POSIXct, format: "2024-02-13" "2024-04-16" ...
##  $ Trial_number             : num  1 1 1 1 1 1 1 1 1 1 ...
##  $ BM1_Date                 : POSIXct, format: "2024-02-13" "2024-04-16" ...
##  $ Age_of_BM                : num  3 3 3 3 3 3 3 3 3 3 ...
##  $ Bloodmeal_number         : Factor w/ 1 level "1": 1 1 1 1 1 1 1 1 1 1 ...
##  $ Oviposition_positive(y/n): chr  "Y" "Y" "Y" "Y" ...
##  $ Eggs_day3                : num  14 72 64 61 53 0 62 72 6 1 ...
##  $ Eggs_day4                : num  0 10 0 1 4 72 0 5 0 0 ...
##  $ Larvae_day4              : num  0 0 0 0 0 NA 9 22 0 0 ...
##  $ Surv_to_eggs(y/n)        : Factor w/ 3 levels "N","NA","Y": 3 3 3 3 3 3 3 1 1 3 ...
##  $ Surv_to_larvae(y/n)      : Factor w/ 3 levels "N","NA","Y": 3 3 3 3 3 3 3 2 2 3 ...
##  $ Date_of_death            : POSIXct, format: "2024-03-02" "2024-04-28" ...
##  $ Censor                   : num  1 1 1 1 1 1 1 1 1 1 ...
##  $ Notes                    : chr  NA NA NA NA ...
##  $ Oviposition_positive     : Factor w/ 2 levels "N","Y": 2 2 2 2 2 2 2 2 2 2 ...
##  $ total_eggs               : num  14 82 64 62 57 72 62 77 6 1 ...
##  $ Percent_eggs_day3        : num  1 0.878 1 0.984 0.93 ...
##  $ Percent_eggs_day4        : num  0 0.122 0 0.0161 0.0702 ...
##  $ egg_binary               : num  1 1 1 1 1 1 1 1 1 1 ...
##  $ egg_binary_day3          : num  1 1 1 1 1 0 1 1 1 1 ...
##  $ egg_binary_day4          : num  0 1 0 1 1 1 0 1 0 0 ...
##  $ larvae_binary            : num  0 0 0 0 0 NA 1 1 0 0 ...
##  $ Percent_eggshatchedtolarv: num  0 0 0 0 0 ...
##  $ Days_to_death_post_BM    : 'difftime' num  18 12 8 17 ...
##   ..- attr(*, "units")= chr "days"
##  $ Age_of_death             : 'difftime' num  21 15 11 20 ...
##   ..- attr(*, "units")= chr "days"
##  $ Age_of_BM_days           : 'difftime' num  3 3 3 3 ...
##   ..- attr(*, "units")= chr "days"
##  $ Date_of_eclosion         : POSIXct, format: "2024-02-10" "2024-04-13" ...
##  $ days_alive_post_BM       : num  18 12 8 17 8 17 10 3 3 11 ...
##  $ days_alive_post_eclosion : num  21 15 11 20 11 20 13 6 6 14 ...
```

```
Fecundity_data_firstBM_ovipos_narm$Temperature <- as.character(Fecundity_data_firstBM_ovipos_narm$Temperatu
re)
Fecundity_data_firstBM_ovipos_narm$Temperature <- as.numeric(Fecundity_data_firstBM_ovipos_narm$Temperatur
e)

Fecundity_data_firstBM_ovipos_narm$Temperature_centered <- scale(Fecundity_data_firstBM_ovipos_narm$Tempera
ture, scale= TRUE, center=TRUE)
Fecundity_data_firstBM_ovipos_narm$Temperature <- as.factor(Fecundity_data_firstBM_ovipos_narm$Temperature)


Fecundity_data_firstBM_ovipos_narm$Age <- as.character(Fecundity_data_firstBM_ovipos_narm$Age)
Fecundity_data_firstBM_ovipos_narm$Age <- as.numeric(Fecundity_data_firstBM_ovipos_narm$Age)

Fecundity_data_firstBM_ovipos_narm$Age_centered <- scale(Fecundity_data_firstBM_ovipos_narm$Age, scale= TRU
E, center=TRUE)
Fecundity_data_firstBM_ovipos_narm$Age <- as.factor(Fecundity_data_firstBM_ovipos_narm$Age)

Fecundity_data_firstBM_ovipos_narm$Age_centered <-round(Fecundity_data_firstBM_ovipos_narm$Age_centered, 2)
Fecundity_data_firstBM_ovipos_narm$Temperature_centered <-round(Fecundity_data_firstBM_ovipos_narm$Temperat
ure_centered, 2)

#analysis for total egg counts, egg number day 3 and day 4:
# count data

str(Fecundity_data_firstBM_ovipos)
```

```
## 'data.frame':    252 obs. of  33 variables:
##  $ ID_overall               : num  6 12 15 18 38 41 42 43 46 47 ...
##  $ Temperature              : Factor w/ 3 levels "27","30","32": 3 1 1 1 2 2 2 2 2 2 ...
##  $ Age                      : Factor w/ 4 levels "3","5","10","15": 1 1 1 1 1 1 1 1 1 1 ...
##  $ ID_per_group             : num  6 2 5 8 3 6 7 8 11 12 ...
##  $ Trial_start_date         : POSIXct, format: "2024-02-13" "2024-04-16" ...
##  $ Trial_number             : num  1 1 1 1 1 1 1 1 1 1 ...
##  $ BM1_Date                 : POSIXct, format: "2024-02-13" "2024-04-16" ...
##  $ Age_of_BM                : num  3 3 3 3 3 3 3 3 3 3 ...
##  $ Bloodmeal_number         : Factor w/ 1 level "1": 1 1 1 1 1 1 1 1 1 1 ...
##  $ Oviposition_positive(y/n): chr  "Y" "Y" "Y" "Y" ...
##  $ Eggs_day3                : num  14 72 64 61 53 0 62 72 6 1 ...
##  $ Eggs_day4                : num  0 10 0 1 4 72 0 5 0 0 ...
##  $ Larvae_day4              : num  0 0 0 0 0 NA 9 22 0 0 ...
##  $ Surv_to_eggs(y/n)        : Factor w/ 3 levels "N","NA","Y": 3 3 3 3 3 3 3 1 1 3 ...
##  $ Surv_to_larvae(y/n)      : Factor w/ 3 levels "N","NA","Y": 3 3 3 3 3 3 3 2 2 3 ...
##  $ Date_of_death            : POSIXct, format: "2024-03-02" "2024-04-28" ...
##  $ Censor                   : num  1 1 1 1 1 1 1 1 1 1 ...
##  $ Notes                    : chr  NA NA NA NA ...
##  $ Oviposition_positive     : Factor w/ 2 levels "N","Y": 2 2 2 2 2 2 2 2 2 2 ...
##  $ total_eggs               : num  14 82 64 62 57 72 62 77 6 1 ...
##  $ Percent_eggs_day3        : num  1 0.878 1 0.984 0.93 ...
##  $ Percent_eggs_day4        : num  0 0.122 0 0.0161 0.0702 ...
##  $ egg_binary               : num  1 1 1 1 1 1 1 1 1 1 ...
##  $ egg_binary_day3          : num  1 1 1 1 1 0 1 1 1 1 ...
##  $ egg_binary_day4          : num  0 1 0 1 1 1 0 1 0 0 ...
##  $ larvae_binary            : num  0 0 0 0 0 NA 1 1 0 0 ...
##  $ Percent_eggshatchedtolarv: num  0 0 0 0 0 ...
##  $ Days_to_death_post_BM    : 'difftime' num  18 12 8 17 ...
##   ..- attr(*, "units")= chr "days"
##  $ Age_of_death             : 'difftime' num  21 15 11 20 ...
##   ..- attr(*, "units")= chr "days"
##  $ Age_of_BM_days           : 'difftime' num  3 3 3 3 ...
##   ..- attr(*, "units")= chr "days"
##  $ Date_of_eclosion         : POSIXct, format: "2024-02-10" "2024-04-13" ...
##  $ days_alive_post_BM       : num  18 12 8 17 8 17 10 3 3 11 ...
##  $ days_alive_post_eclosion : num  21 15 11 20 11 20 13 6 6 14 ...
```

```
#this includes mosquitoes that survived to lay eggs day 3, laid >0 eggs day 3,
#NA values in total_eggs if did not survive to day 4 eggs even if laid eggs day 3
#total eggs only mosquitoes that survived >0 day 3.

#inspect
Fecundity_data_firstBM_ovipos$log_total_eggs <- log(Fecundity_data_firstBM_ovipos$total_eggs +0.1)

hist(Fecundity_data_firstBM_ovipos$total_eggs)
```

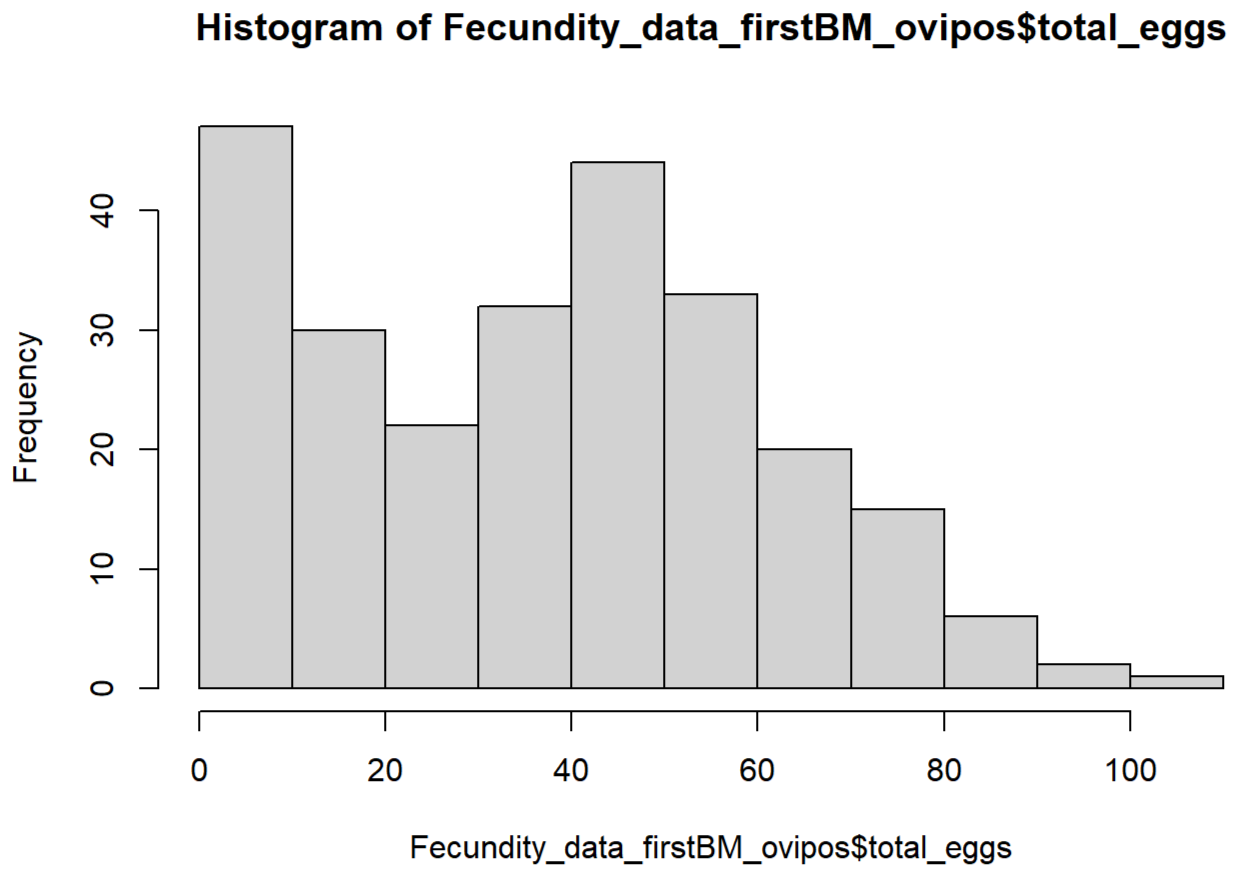


```
hist(Fecundity_data_firstBM_ovipos$log_total_eggs)
```

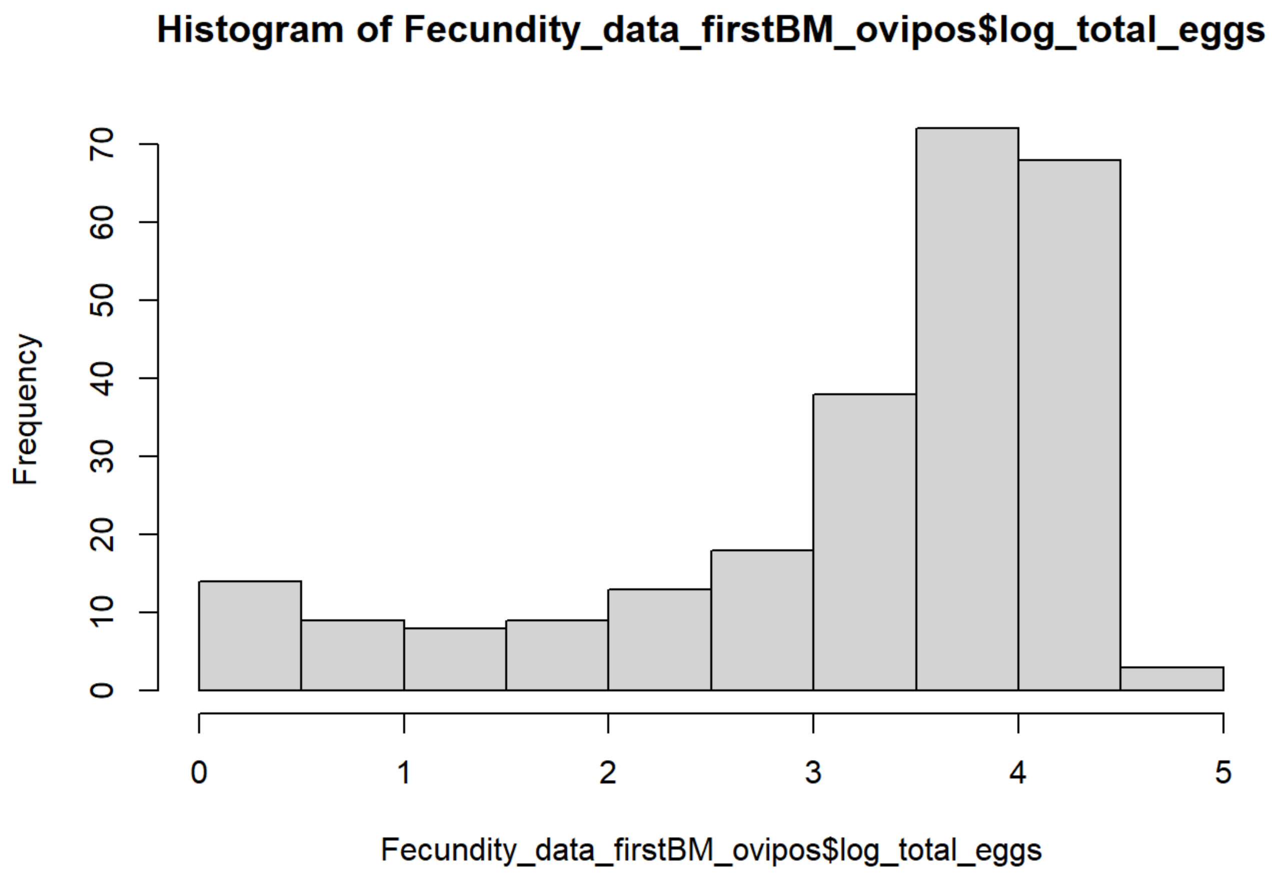

```
hist(Fecundity_data_firstBM_ovipos$Eggs_day3)
```

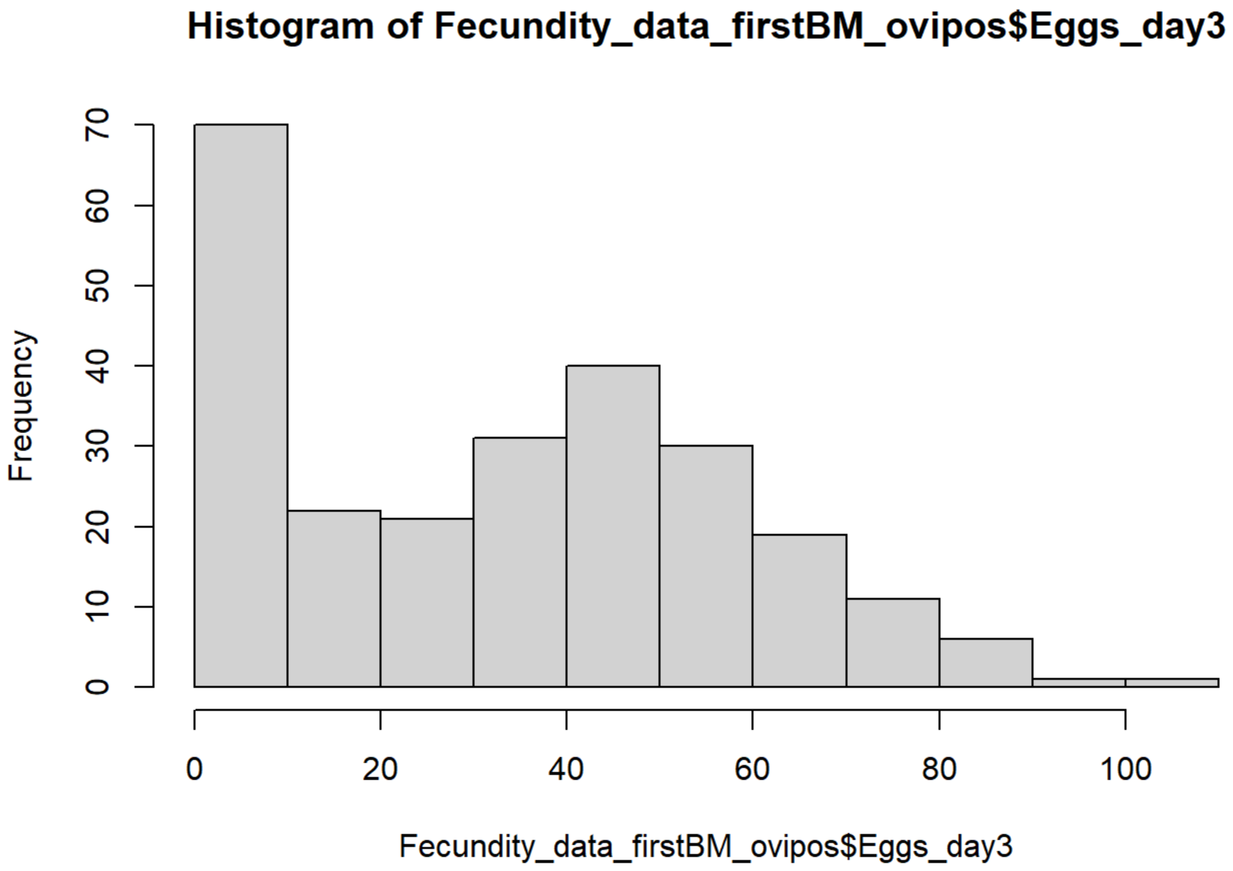


```
hist(Fecundity_data_firstBM_ovipos$Eggs_day4)
```

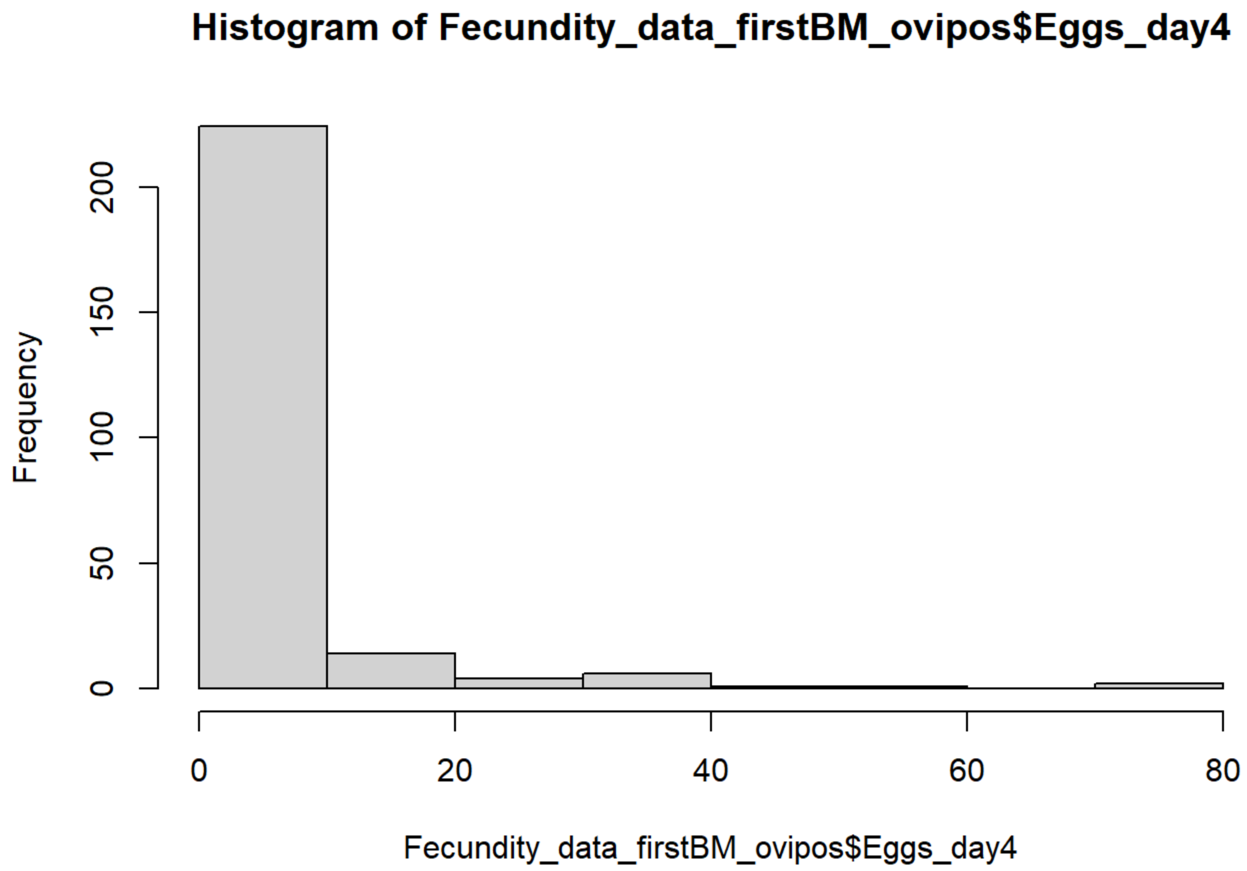


```
shapiro.test(Fecundity_data_firstBM_ovipos$total_eggs)
```

```
##
##  Shapiro-Wilk normality test
##
## data:  Fecundity_data_firstBM_ovipos$total_eggs
## W = 0.96431, p-value = 6.404e-06
```

```
shapiro.test(Fecundity_data_firstBM_ovipos$log_total_eggs)
```

```
##
##  Shapiro-Wilk normality test
##
## data:  Fecundity_data_firstBM_ovipos$log_total_eggs
## W = 0.82369, p-value = 3.207e-16
```

```
shapiro.test(Fecundity_data_firstBM_ovipos$Eggs_day3)
```

```
##
##  Shapiro-Wilk normality test
##
## data:  Fecundity_data_firstBM_ovipos$Eggs_day3
## W = 0.93932, p-value = 1.081e-08
```

```
shapiro.test(Fecundity_data_firstBM_ovipos$Eggs_day4)
```

```
##
##  Shapiro-Wilk normality test
##
## data:  Fecundity_data_firstBM_ovipos$Eggs_day4
## W = 0.44271, p-value < 2.2e-16
```

```
#non-normal. count data.

library(glmmTMB)
#model fitting:
model.totaleggs.1 = glmmTMB(total_eggs ~ Temperature+Age+(1|Trial_number), data= Fecundity_data_firstBM_ovi
pos_narm,family=poisson(link="log")) #equals model 1
model.totaleggs.2 = glmmTMB(total_eggs ~ Temperature*Age+(1|Trial_number), data= Fecundity_data_firstBM_ovi
pos_narm,family=poisson(link="log")) #equals model 1

#overdispersion?
pr <- residuals(model.totaleggs.2,"pearson")
phi <- sum(pr^2)/df.residual(model.totaleggs.2)
round(c(phi,sqrt(phi)),4) #variance is higher
```

```
## [1] 9.9075 3.1476
```

```
library(DHARMa)

plot(simulateResiduals(model.totaleggs.1))
```

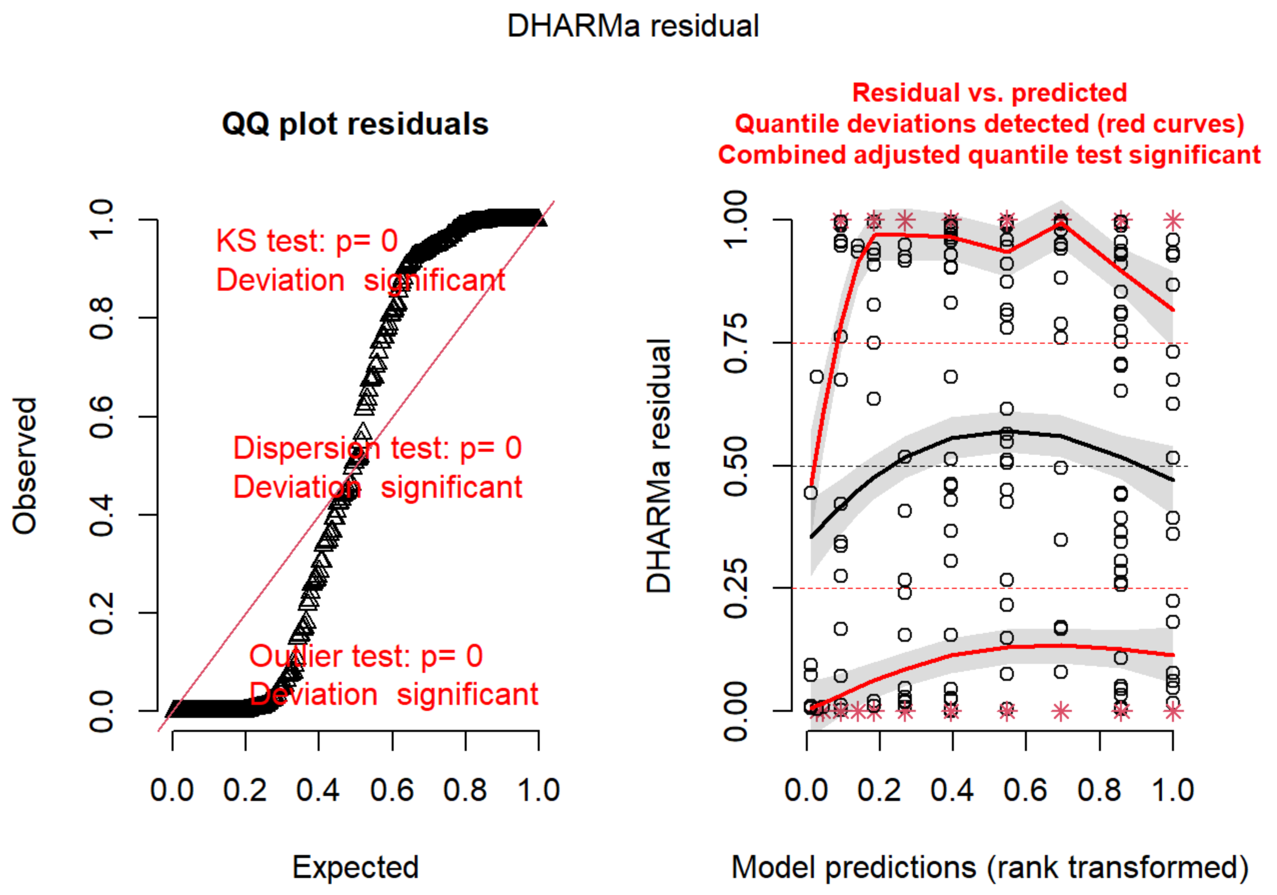

```
plot(fitted(model.totaleggs.1),
     residuals(model.totaleggs.1))
```

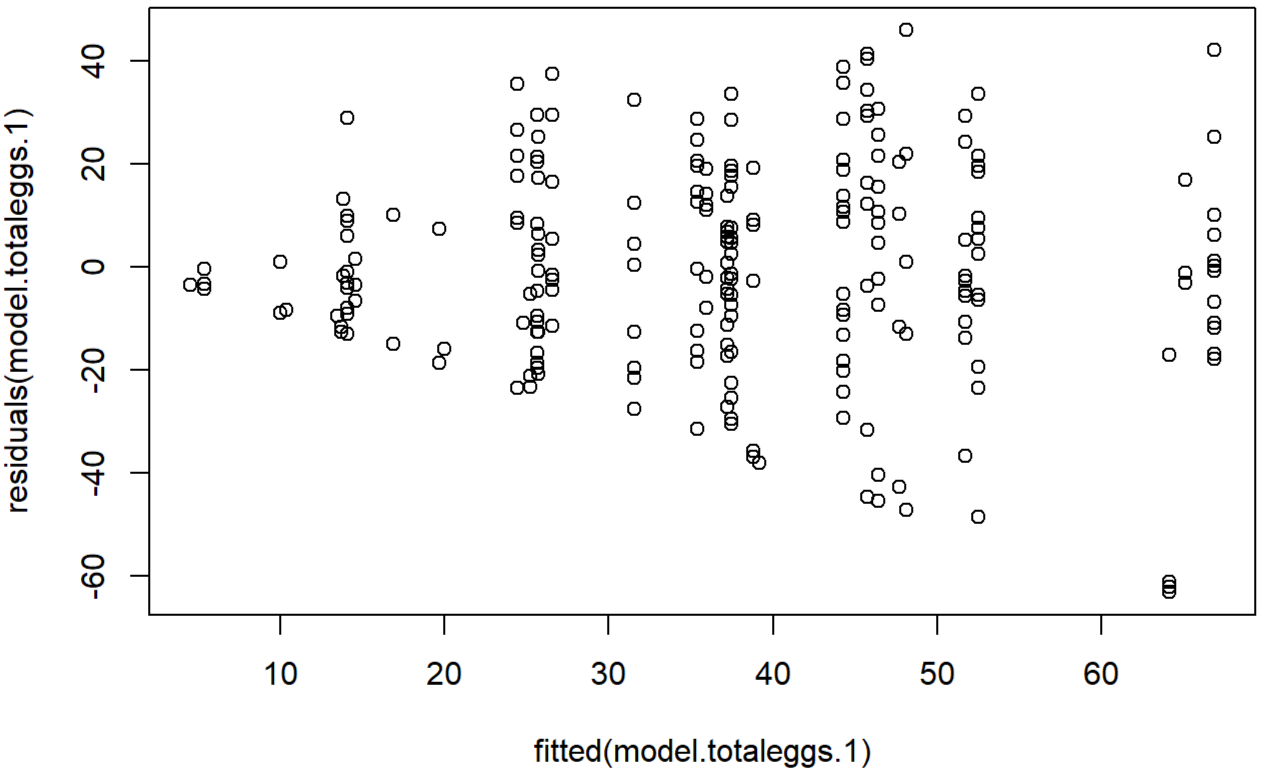


```
plot(simulateResiduals(model.totaleggs.2))
```

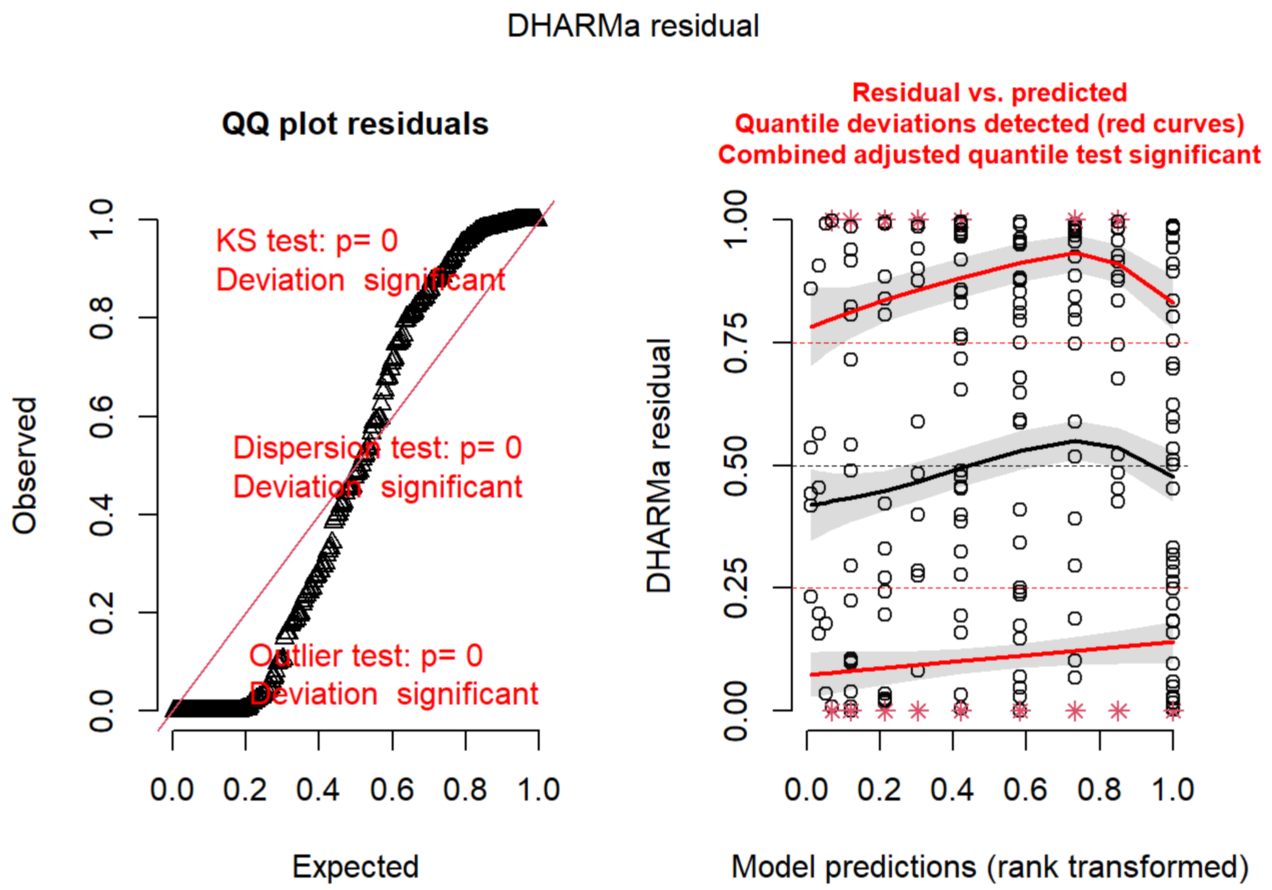


```
plot(fitted(model.totaleggs.2),
     residuals(model.totaleggs.2))
```

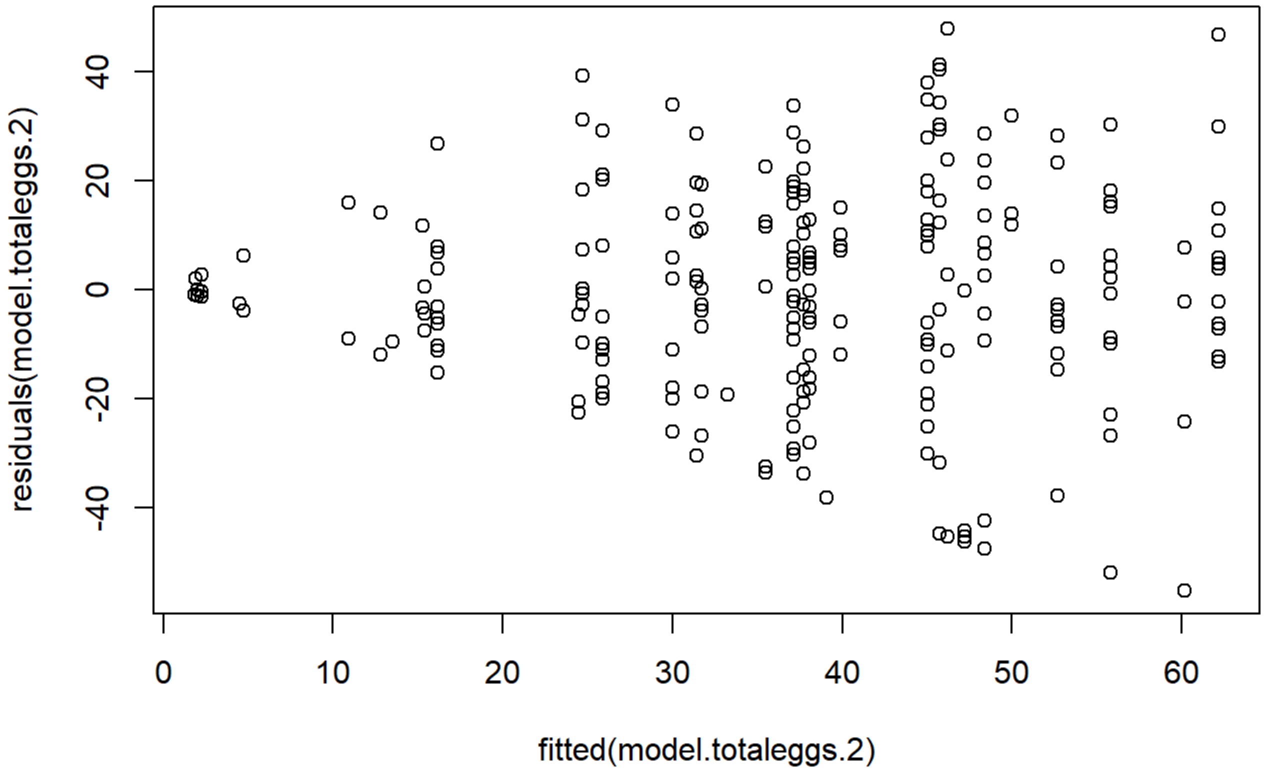

```
model.totaleggs.3 = glmmTMB(total_eggs ~ Temperature*Age, data= Fecundity_data_firstBM_ovipos,family=nbinom
2)
plot(simulateResiduals(model.totaleggs.3))
```

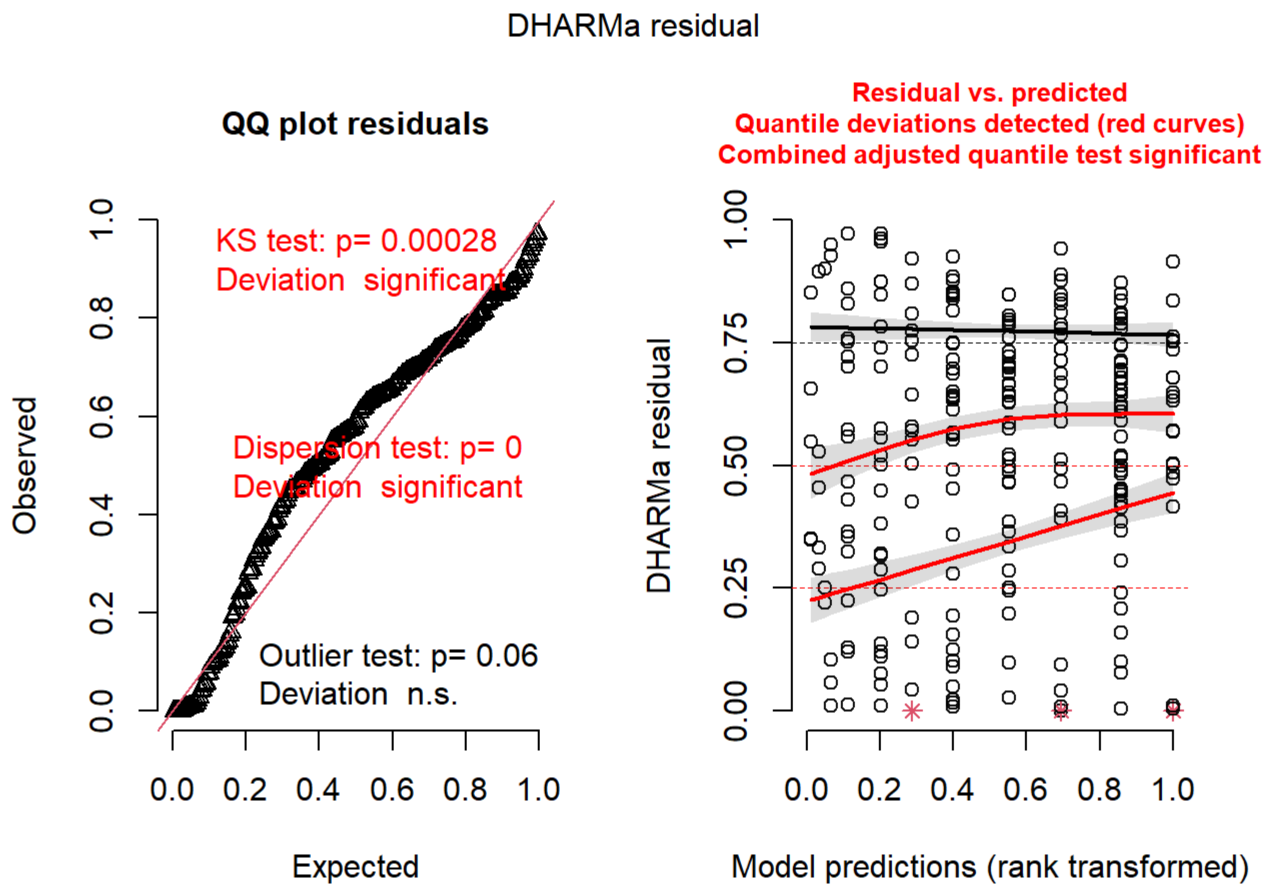


```
plot(fitted(model.totaleggs.3),
     residuals(model.totaleggs.3))
```

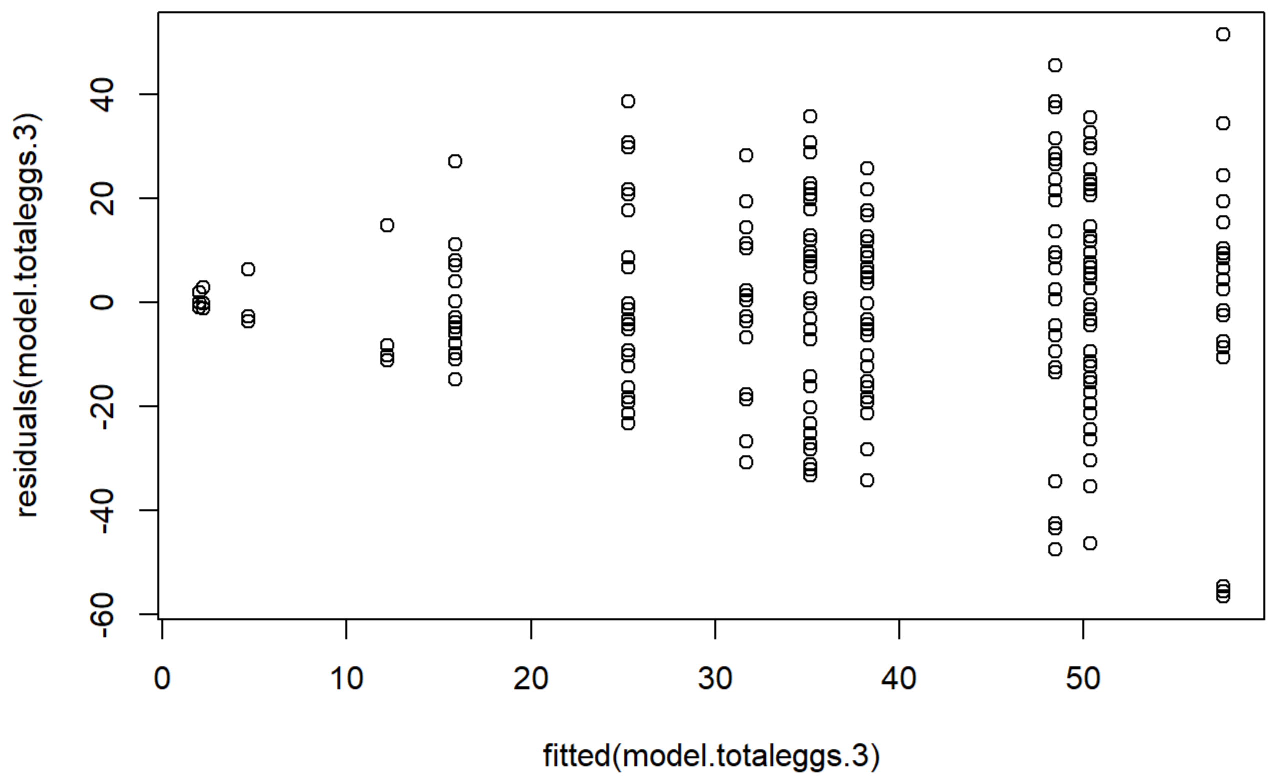


```
model.totaleggs.4 = glmmTMB(total_eggs ~ Temperature*Age + (1|Trial_number),
                            data= Fecundity_data_firstBM_ovipos_narm,
                            family=nbinom2(link = "log"))
plot(simulateResiduals(model.totaleggs.4))
```

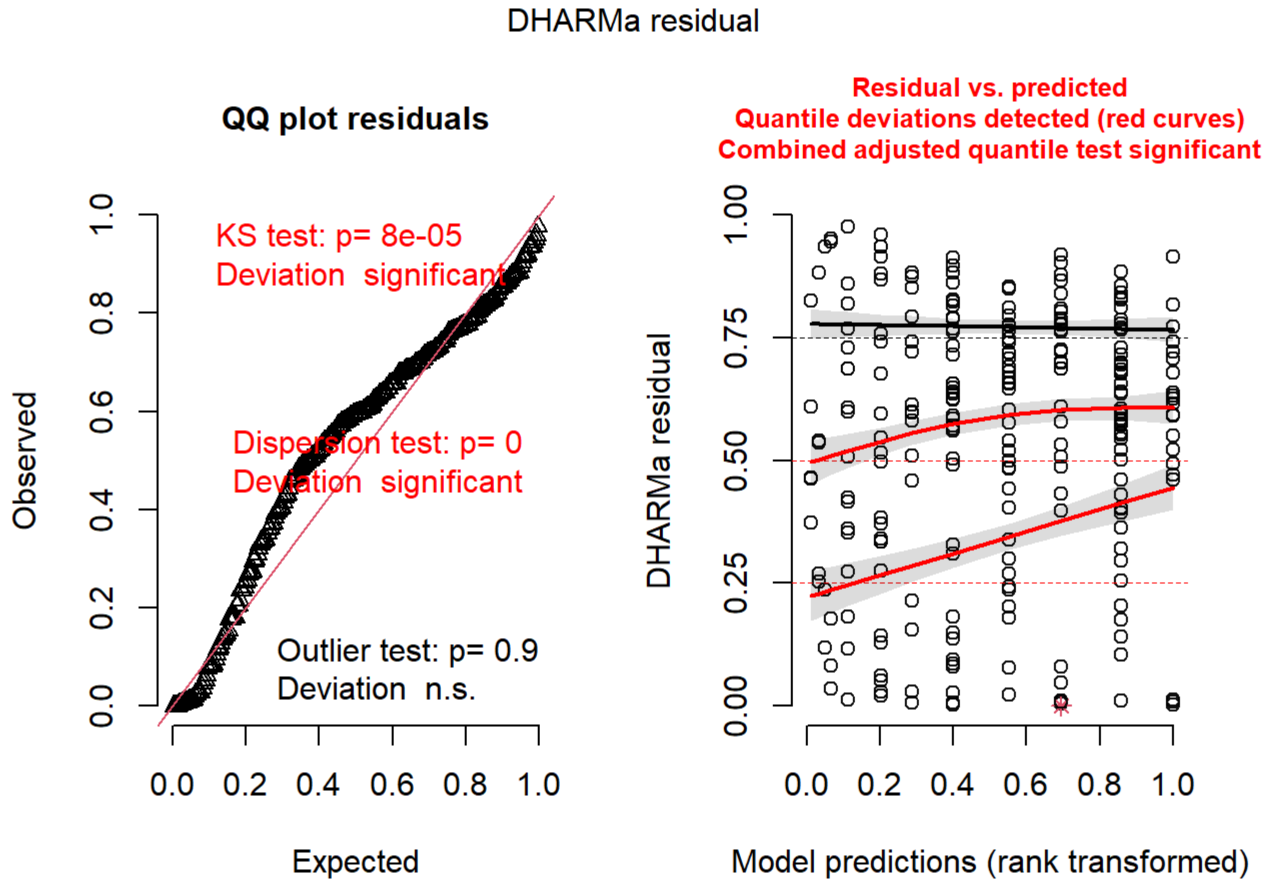


```
plot(fitted(model.totaleggs.4),
     residuals(model.totaleggs.4))
```

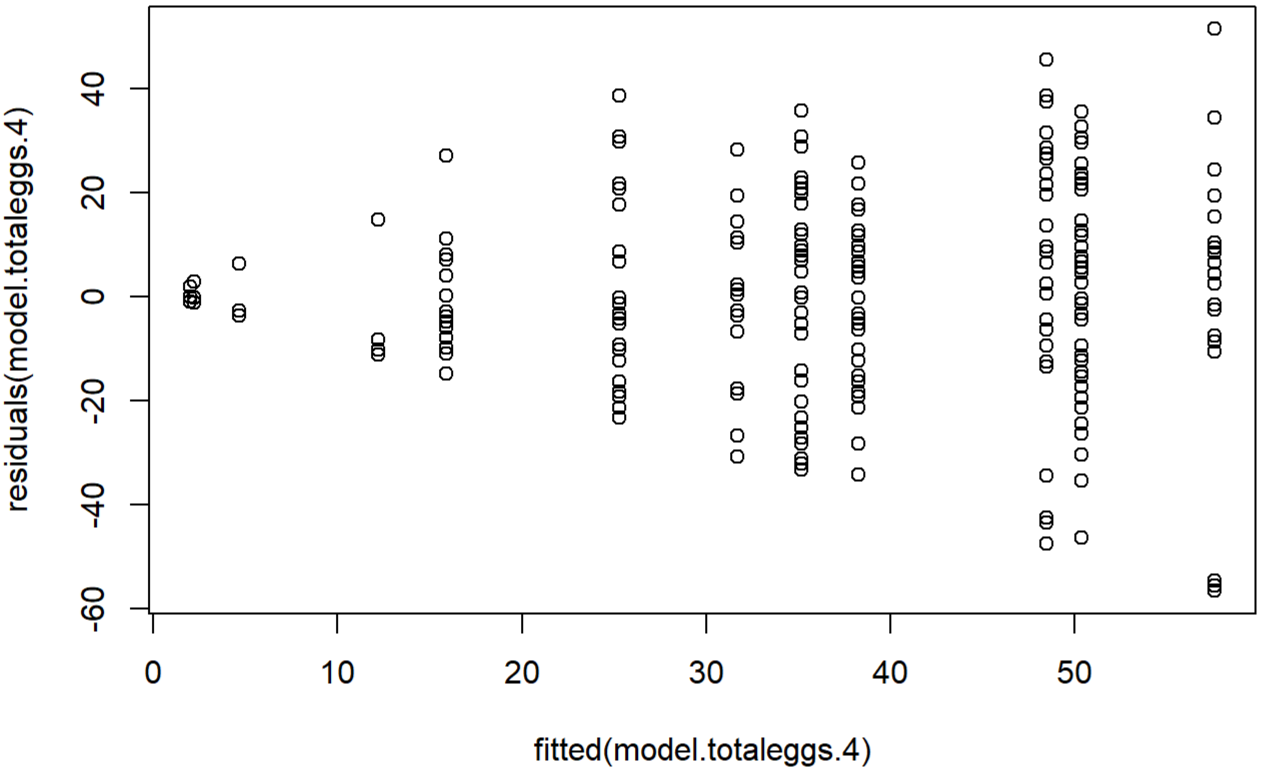

```
summary(model.totaleggs.4)
```

```
##  Family: nbinom2  ( log )
## Formula:          total_eggs ~ Temperature * Age + (1 | Trial_number)
## Data: Fecundity_data_firstBM_ovipos_narm
##
##      AIC      BIC   logLik deviance df.resid
##   2221.2   2270.6  -1096.6   2193.2      238
##
## Random effects:
##
## Conditional model:
##  Groups       Name        Variance Std.Dev.
##  Trial_number (Intercept) 5.19e-10 2.278e-05
## Number of obs: 252, groups:  Trial_number, 5
##
## Dispersion parameter for nbinom2 family (): 2.39
##
## Conditional model:
##                       Estimate Std. Error z value Pr(>|z|)
## (Intercept)             4.0530     0.1439  28.162  < 2e-16 ***
## Temperature30          -0.1724     0.1844  -0.935  0.34975
## Temperature32          -0.5971     0.2209  -2.703  0.00686 **
## Age5                   -0.1338     0.1733  -0.772  0.43999
## Age10                  -0.4082     0.1818  -2.246  0.02473 *
## Age15                  -1.2874     0.2177  -5.913 3.36e-09 ***
## Temperature30:Age5     -0.1862     0.2346  -0.794  0.42729
## Temperature32:Age5     -0.8207     0.3975  -2.065  0.03896 *
## Temperature30:Age10    -0.2425     0.2542  -0.954  0.34013
## Temperature32:Age10    -2.3546     0.4946  -4.761 1.93e-06 ***
## Temperature30:Age15    -1.0528     0.5209  -2.021  0.04326 *
## Temperature32:Age15    -1.3801     0.4999  -2.761  0.00577 **
## ---
## Signif. codes:  0 '***' 0.001 '**' 0.01 '*' 0.05 '.' 0.1 ' ' 1
```

```
Anova(model.totaleggs.4,type=2)
```

```
## Analysis of Deviance Table (Type II Wald chisquare tests)
##
## Response: total_eggs
##                    Chisq Df Pr(>Chisq)
## Temperature       69.208  2  9.370e-16 ***
## Age              111.408  3  < 2.2e-16 ***
## Temperature:Age   29.208  6  5.556e-05 ***
## ---
## Signif. codes:  0 '***' 0.001 '**' 0.01 '*' 0.05 '.' 0.1 ' ' 1
```

```
model.totaleggs.5 = glmmTMB(total_eggs ~ Temperature_centered*Age_centered + (1|Trial_number), data= Fecund
ity_data_firstBM_ovipos_narm,
                            family=nbinom2(link = "log"))
plot(simulateResiduals(model.totaleggs.5))
```

```
## qu = 0.75, log(sigma) = -2.85947 : outer Newton did not converge fully.
```

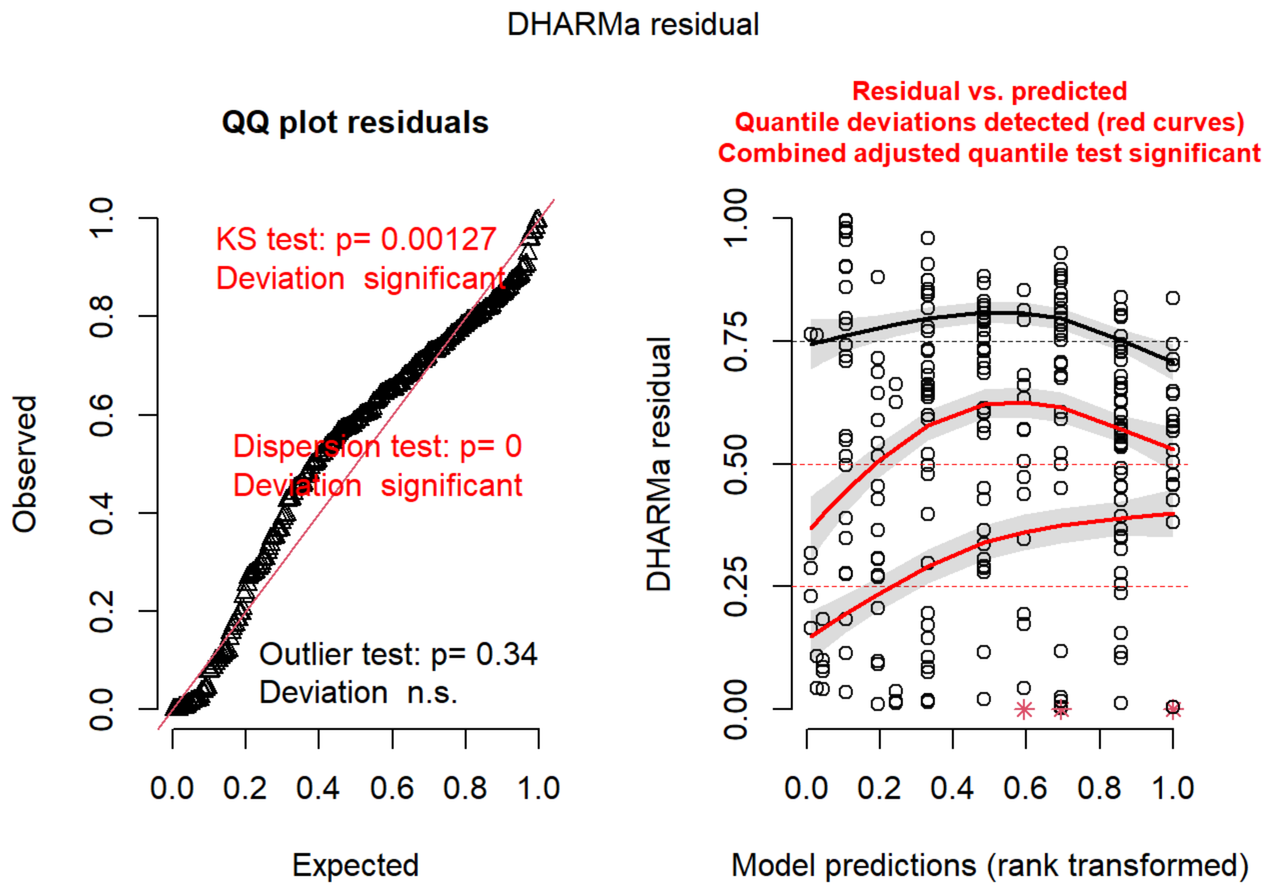


```
plot(fitted(model.totaleggs.5),
     residuals(model.totaleggs.5))
```

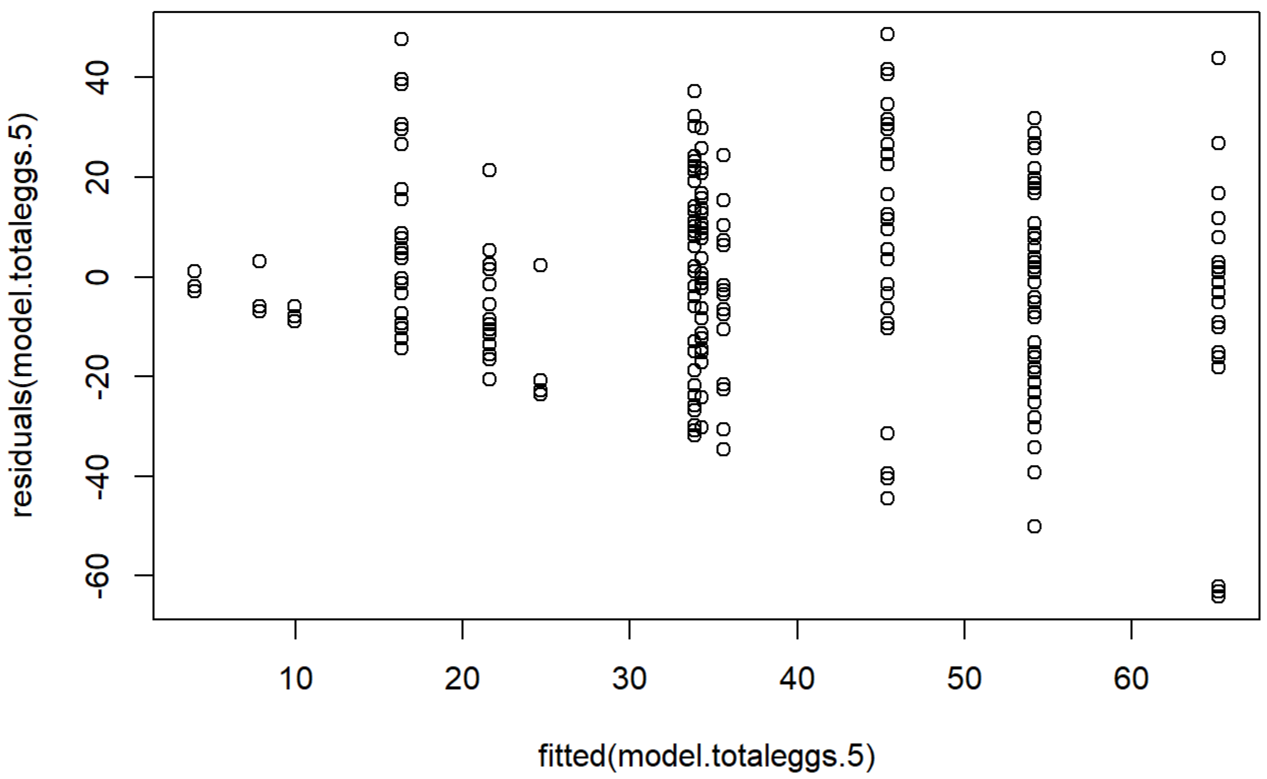


```
summary(model.totaleggs.5)
```

```
##  Family: nbinom2  ( log )
## Formula:
## total_eggs ~ Temperature_centered * Age_centered + (1 | Trial_number)
## Data: Fecundity_data_firstBM_ovipos_narm
##
##      AIC      BIC   logLik deviance df.resid
##   2241.9   2263.1  -1114.9   2229.9      246
##
## Random effects:
##
## Conditional model:
##  Groups       Name        Variance  Std.Dev.
##  Trial_number (Intercept) 1.201e-09 3.466e-05
## Number of obs: 252, groups:  Trial_number, 5
##
## Dispersion parameter for nbinom2 family (): 2.08
##
## Conditional model:
##                                   Estimate Std. Error z value Pr(>|z|)
## (Intercept)                        3.48978    0.04637   75.26  < 2e-16 ***
## Temperature_centered              -0.34941    0.04963   -7.04 1.91e-12 ***
## Age_centered                      -0.48047    0.05021   -9.57  < 2e-16 ***
## Temperature_centered:Age_centered -0.12901    0.04966   -2.60  0.00939 **
## ---
## Signif. codes:  0 '***' 0.001 '**' 0.01 '*' 0.05 '.' 0.1 ' ' 1
```

```
Anova(model.totaleggs.5,type=2)
```

```
## Analysis of Deviance Table (Type II Wald chisquare tests)
##
## Response: total_eggs
##                                    Chisq Df Pr(>Chisq)
## Temperature_centered             46.5716  1  8.833e-12 ***
## Age_centered                     87.2755  1  < 2.2e-16 ***
## Temperature_centered:Age_centered  6.7473  1   0.009389 **
## ---
## Signif. codes:  0 '***' 0.001 '**' 0.01 '*' 0.05 '.' 0.1 ' ' 1
```

```
model.totaleggs.6 = glmmTMB(total_eggs ~ Temperature_centered*Age_centered+(1|Trial_number), data= Fecundit
y_data_firstBM_ovipos_narm,family=poisson(link="log")) #equals model 1

require(fitdistrplus)
```

```
## Loading required package: fitdistrplus
```

```
fit_negbinom_CFUs <- fitdist((Fecundity_data_firstBM_ovipos_narm$total_eggs), "pois")
summary(fit_negbinom_CFUs)
```

```
## Fitting of the distribution ' pois ' by maximum likelihood
## Parameters :
##        estimate Std. Error
## lambda 37.34524  0.3849615
## Loglikelihood:  -2963.447   AIC:  5928.893   BIC:  5932.423
```

```
plot(fit_negbinom_CFUs)
```

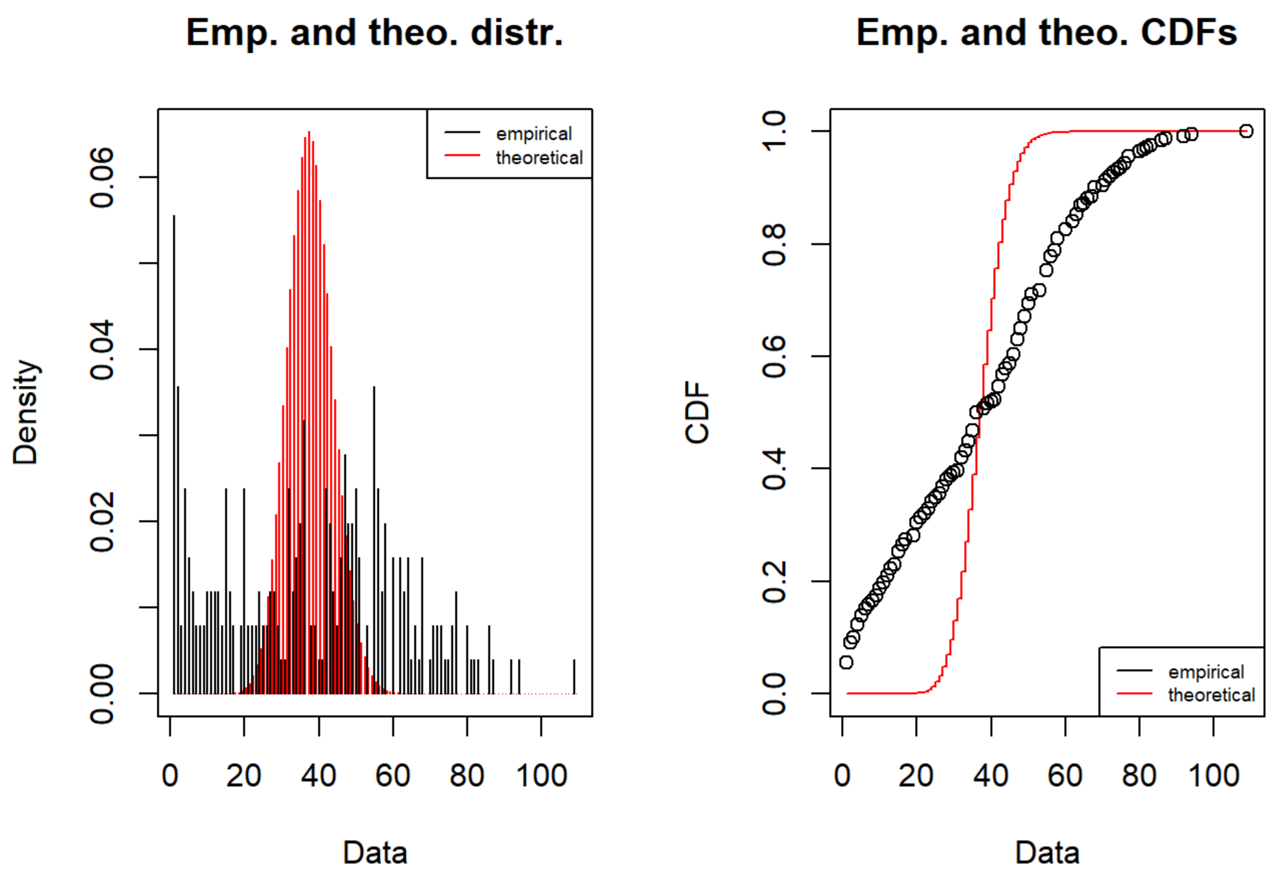


```
library(lmtest)
lrtest(model.totaleggs.2,model.totaleggs.4)
```

```
## Likelihood ratio test
##
## Model 1: total_eggs ~ Temperature * Age + (1 | Trial_number)
## Model 2: total_eggs ~ Temperature * Age + (1 | Trial_number)
##   #Df  LogLik Df  Chisq Pr(>Chisq)
## 1  13 -2066.4
## 2  14 -1096.6  1 1939.7  < 2.2e-16 ***
## ---
## Signif. codes:  0 '***' 0.001 '**' 0.01 '*' 0.05 '.' 0.1 ' ' 1
```

```
AIC(model.totaleggs.2,model.totaleggs.3,model.totaleggs.4,model.totaleggs.5,model.totaleggs.6)
```

```
##                   df      AIC
## model.totaleggs.2 13 4158.866
## model.totaleggs.3 13 2219.163
## model.totaleggs.4 14 2221.163
## model.totaleggs.5  6 2241.888
## model.totaleggs.6  5 4434.417
```

```
#4 has lowest aic and is clear to interpret. matches other analyses.
#negative binomial is better

BM1_ovipos_anova <- Anova(model.totaleggs.4,type=2)
BM1_ovipos_anova <- as.data.frame(BM1_ovipos_anova)
write_xlsx(BM1_ovipos_anova, "Oviposition_eggsandlarvae/BM1_ovipos_anova.xlsx")

sink("Oviposition_eggsandlarvae/BM1_ovipos_anova.txt")
Anova(model.totaleggs.4,type=2)
sink()

sink("Oviposition_eggsandlarvae/BM1_ovipostotaleggs_modelsummary.txt")
summary(model.totaleggs.4)
sink()

BM1_ovipos_emmeans <- emmeans(model.totaleggs.4,~Temperature*Age,
                              type="response")
BM1_ovipos_emmeans_PAIRS <- pairs(BM1_ovipos_emmeans, adjust="sidak")
BM1_ovipos_emmeans_PAIRS
```

```
##  contrast                                   ratio      SE  df null z.ratio
##  Temperature27 Age3 / Temperature30 Age3    1.188  0.2191 Inf    1   0.935
##  Temperature27 Age3 / Temperature32 Age3    1.817  0.4013 Inf    1   2.703
##  Temperature27 Age3 / Temperature27 Age5    1.143  0.1981 Inf    1   0.772
##  Temperature27 Age3 / Temperature30 Age5    1.636  0.2948 Inf    1   2.734
##  Temperature27 Age3 / Temperature32 Age5    4.719  1.6389 Inf    1   4.467
##  Temperature27 Age3 / Temperature27 Age10   1.504  0.2734 Inf    1   2.246
##  Temperature27 Age3 / Temperature30 Age10   2.277  0.4497 Inf    1   4.168
##  Temperature27 Age3 / Temperature32 Age10  28.786 13.0088 Inf    1   7.435
##  Temperature27 Age3 / Temperature27 Age15   3.623  0.7889 Inf    1   5.913
##  Temperature27 Age3 / Temperature30 Age15  12.337  5.9337 Inf    1   5.224
##  Temperature27 Age3 / Temperature32 Age15  26.169 11.5600 Inf    1   7.390
##  Temperature30 Age3 / Temperature32 Age3    1.529  0.3110 Inf    1   2.089
##  Temperature30 Age3 / Temperature27 Age5    0.962  0.1446 Inf    1  -0.257
##  Temperature30 Age3 / Temperature30 Age5    1.377  0.2178 Inf    1   2.023
##  Temperature30 Age3 / Temperature32 Age5    3.972  1.3362 Inf    1   4.099
##  Temperature30 Age3 / Temperature27 Age10   1.266  0.2026 Inf    1   1.473
##  Temperature30 Age3 / Temperature30 Age10   1.917  0.3405 Inf    1   3.662
##  Temperature30 Age3 / Temperature32 Age10  24.227 10.7477 Inf    1   7.185
##  Temperature30 Age3 / Temperature27 Age15   3.050  0.6097 Inf    1   5.577
##  Temperature30 Age3 / Temperature30 Age15  10.383  4.9131 Inf    1   4.946
##  Temperature30 Age3 / Temperature32 Age15  22.025  9.5423 Inf    1   7.137
##  Temperature32 Age3 / Temperature27 Age5    0.629  0.1216 Inf    1  -2.396
##  Temperature32 Age3 / Temperature30 Age5    0.901  0.1797 Inf    1  -0.525
##  Temperature32 Age3 / Temperature32 Age5    2.597  0.9292 Inf    1   2.668
##  Temperature32 Age3 / Temperature27 Age10   0.828  0.1664 Inf    1  -0.940
##  Temperature32 Age3 / Temperature30 Age10   1.253  0.2699 Inf    1   1.049
##  Temperature32 Age3 / Temperature32 Age10  15.844  7.2879 Inf    1   6.006
##  Temperature32 Age3 / Temperature27 Age15   1.994  0.4667 Inf    1   2.950
##  Temperature32 Age3 / Temperature30 Age15   6.790  3.3174 Inf    1   3.921
##  Temperature32 Age3 / Temperature32 Age15  14.403  6.4815 Inf    1   5.928
##  Temperature27 Age5 / Temperature30 Age5    1.431  0.2076 Inf    1   2.472
##  Temperature27 Age5 / Temperature32 Age5    4.128  1.3642 Inf    1   4.290
##  Temperature27 Age5 / Temperature27 Age10   1.316  0.1935 Inf    1   1.865
##  Temperature27 Age5 / Temperature30 Age10   1.992  0.3309 Inf    1   4.149
##  Temperature27 Age5 / Temperature32 Age10  25.181 11.0574 Inf    1   7.347
##  Temperature27 Age5 / Temperature27 Age15   3.170  0.6014 Inf    1   6.080
##  Temperature27 Age5 / Temperature30 Age15  10.792  5.0610 Inf    1   5.072
##  Temperature27 Age5 / Temperature32 Age15  22.892  9.8123 Inf    1   7.304
##  Temperature30 Age5 / Temperature32 Age5    2.884  0.9636 Inf    1   3.170
##  Temperature30 Age5 / Temperature27 Age10   0.919  0.1426 Inf    1  -0.543
##  Temperature30 Age5 / Temperature30 Age10   1.392  0.2412 Inf    1   1.908
##  Temperature30 Age5 / Temperature32 Age10  17.592  7.7735 Inf    1   6.489
##  Temperature30 Age5 / Temperature27 Age15   2.214  0.4341 Inf    1   4.055
##  Temperature30 Age5 / Temperature30 Age15   7.539  3.5553 Inf    1   4.284
##  Temperature30 Age5 / Temperature32 Age15  15.993  6.9004 Inf    1   6.425
##  Temperature32 Age5 / Temperature27 Age10   0.319  0.1068 Inf    1  -3.413
##  Temperature32 Age5 / Temperature30 Age10   0.483  0.1659 Inf    1  -2.119
##  Temperature32 Age5 / Temperature32 Age10   6.100  3.2475 Inf    1   3.397
##  Temperature32 Age5 / Temperature27 Age15   0.768  0.2732 Inf    1  -0.742
##  Temperature32 Age5 / Temperature30 Age15   2.614  1.4568 Inf    1   1.725
##  Temperature32 Age5 / Temperature32 Age15   5.545  2.9046 Inf    1   3.270
##  Temperature27 Age10 / Temperature30 Age10  1.514  0.2649 Inf    1   2.371
##  Temperature27 Age10 / Temperature32 Age10 19.139  8.4697 Inf    1   6.670
##  Temperature27 Age10 / Temperature27 Age15  2.409  0.4759 Inf    1   4.451
##  Temperature27 Age10 / Temperature30 Age15  8.202  3.8730 Inf    1   4.457
```

```
##  Temperature27 Age10 / Temperature32 Age15 17.399  7.5189 Inf    1   6.610
##  Temperature30 Age10 / Temperature32 Age10 12.640  5.6782 Inf    1   5.647
##  Temperature30 Age10 / Temperature27 Age15  1.591  0.3374 Inf    1   2.190
##  Temperature30 Age10 / Temperature30 Age15  5.417  2.5918 Inf    1   3.531
##  Temperature30 Age10 / Temperature32 Age15 11.491  5.0444 Inf    1   5.562
##  Temperature32 Age10 / Temperature27 Age15  0.126  0.0577 Inf    1  -4.520
##  Temperature32 Age10 / Temperature30 Age15  0.429  0.2691 Inf    1  -1.350
##  Temperature32 Age10 / Temperature32 Age15  0.909  0.5439 Inf    1  -0.159
##  Temperature27 Age15 / Temperature30 Age15  3.405  1.6586 Inf    1   2.515
##  Temperature27 Age15 / Temperature32 Age15  7.222  3.2389 Inf    1   4.409
##  Temperature30 Age15 / Temperature32 Age15  2.121  1.3163 Inf    1   1.212
##  p.value
##   1.0000
##   0.3653
##   1.0000
##   0.3395
##   0.0005
##   0.8085
##   0.0020
##   <.0001
##   <.0001
##   <.0001
##   <.0001
##   0.9155
##   1.0000
##   0.9452
##   0.0027
##   1.0000
##   0.0164
##   <.0001
##   <.0001
##   0.0001
##   <.0001
##   0.6678
##   1.0000
##   0.3968
##   1.0000
##   1.0000
##   <.0001
##   0.1896
##   0.0058
##   <.0001
##   0.5903
##   0.0012
##   0.9855
##   0.0022
##   <.0001
##   <.0001
##   <.0001
##   <.0001
##   0.0958
##   1.0000
##   0.9783
##   <.0001
##   0.0033
##   0.0012
```

```
##   <.0001
##   0.0415
##   0.8985
##   0.0440
##   1.0000
##   0.9971
##   0.0684
##   0.6930
##   <.0001
##   0.0006
##   0.0005
##   <.0001
##   <.0001
##   0.8522
##   0.0269
##   <.0001
##   0.0004
##   1.0000
##   1.0000
##   0.5463
##   0.0007
##   1.0000
##
## P value adjustment: sidak method for 66 tests
## Tests are performed on the log scale
```

```
BM1_ovipos_emmeans_PAIRS <- as.data.frame(BM1_ovipos_emmeans_PAIRS)
write_xlsx(BM1_ovipos_emmeans_PAIRS, "Oviposition_eggsandlarvae/BM1_ovipos_emmeans_PAIRS.xlsx")

BM1_ovipos_emmeans_TEMP <- emmeans(model.totaleggs.4,~Temperature,
                                   type="response")
```

```
## NOTE: Results may be misleading due to involvement in interactions
```

```
BM1_ovipos_emmeans_TEMP_PAIRS <- pairs(BM1_ovipos_emmeans_TEMP, adjust="sidak")
BM1_ovipos_emmeans_TEMP_PAIRS
```

```
##  contrast                        ratio    SE  df null z.ratio p.value
##  Temperature27 / Temperature30  1.72 0.244 Inf    1   3.820  0.0004
##  Temperature27 / Temperature32  5.67 1.057 Inf    1   9.321  <.0001
##  Temperature30 / Temperature32  3.30 0.709 Inf    1   5.549  <.0001
##
## Results are averaged over the levels of: Age
## P value adjustment: sidak method for 3 tests
## Tests are performed on the log scale
```

```
BM1_ovipos_emmeans_TEMP_PAIRS <- as.data.frame(BM1_ovipos_emmeans_TEMP_PAIRS)
write_xlsx(BM1_ovipos_emmeans_TEMP_PAIRS, "Oviposition_eggsandlarvae/BM1_ovipos_emmeans_TEMP_PAIRS.xlsx")

BM1_ovipos_emmeans_AGE <- emmeans(model.totaleggs.4,~Age,
                                  type="response")
```

```
## NOTE: Results may be misleading due to involvement in interactions
```

```
BM1_ovipos_emmeans_AGE_PAIRS <- pairs(BM1_ovipos_emmeans_AGE, adjust="sidak")
BM1_ovipos_emmeans_AGE_PAIRS
```

```
##  contrast      ratio    SE  df null z.ratio p.value
##  Age3 / Age5    1.60 0.228 Inf    1   3.292  0.0060
##  Age3 / Age10   3.57 0.626 Inf    1   7.272  <.0001
##  Age3 / Age15   8.15 1.871 Inf    1   9.145  <.0001
##  Age5 / Age10   2.24 0.431 Inf    1   4.169  0.0002
##  Age5 / Age15   5.10 1.240 Inf    1   6.695  <.0001
##  Age10 / Age15  2.28 0.601 Inf    1   3.127  0.0106
##
## Results are averaged over the levels of: Temperature
## P value adjustment: sidak method for 6 tests
## Tests are performed on the log scale
```

```
BM1_ovipos_emmeans_AGE_PAIRS <- as.data.frame(BM1_ovipos_emmeans_AGE_PAIRS)
write_xlsx(BM1_ovipos_emmeans_AGE_PAIRS, "Oviposition_eggsandlarvae/BM1_ovipos_emmeans_AGE_PAIRS.xlsx")



sink("Oviposition_eggsandlarvae/BM1_ovipos_totaleggsmodelparam.txt")
parameters::model_parameters(
  model.totaleggs.4, exponentiate = TRUE, ci_method = "wald",
  effects = "all",
  component = "conditional",
  group_level = TRUE,
  verbose = FALSE
)
sink()
```

Analysis for % of eggs laid on day 3 (vs day 4)

```
###############
Fecundity_data_firstBM_ovipos_narm <- Fecundity_data_firstBM_ovipos %>%
  drop_na(total_eggs)
str(Fecundity_data_firstBM_ovipos_narm)
```

```
## 'data.frame':    252 obs. of  34 variables:
##  $ ID_overall               : num  6 12 15 18 38 41 42 43 46 47 ...
##  $ Temperature              : Factor w/ 3 levels "27","30","32": 3 1 1 1 2 2 2 2 2 2 ...
##  $ Age                      : Factor w/ 4 levels "3","5","10","15": 1 1 1 1 1 1 1 1 1 1 ...
##  $ ID_per_group             : num  6 2 5 8 3 6 7 8 11 12 ...
##  $ Trial_start_date         : POSIXct, format: "2024-02-13" "2024-04-16" ...
##  $ Trial_number             : num  1 1 1 1 1 1 1 1 1 1 ...
##  $ BM1_Date                 : POSIXct, format: "2024-02-13" "2024-04-16" ...
##  $ Age_of_BM                : num  3 3 3 3 3 3 3 3 3 3 ...
##  $ Bloodmeal_number         : Factor w/ 1 level "1": 1 1 1 1 1 1 1 1 1 1 ...
##  $ Oviposition_positive(y/n): chr  "Y" "Y" "Y" "Y" ...
##  $ Eggs_day3                : num  14 72 64 61 53 0 62 72 6 1 ...
##  $ Eggs_day4                : num  0 10 0 1 4 72 0 5 0 0 ...
##  $ Larvae_day4              : num  0 0 0 0 0 NA 9 22 0 0 ...
##  $ Surv_to_eggs(y/n)        : Factor w/ 3 levels "N","NA","Y": 3 3 3 3 3 3 3 1 1 3 ...
##  $ Surv_to_larvae(y/n)      : Factor w/ 3 levels "N","NA","Y": 3 3 3 3 3 3 3 2 2 3 ...
##  $ Date_of_death            : POSIXct, format: "2024-03-02" "2024-04-28" ...
##  $ Censor                   : num  1 1 1 1 1 1 1 1 1 1 ...
##  $ Notes                    : chr  NA NA NA NA ...
##  $ Oviposition_positive     : Factor w/ 2 levels "N","Y": 2 2 2 2 2 2 2 2 2 2 ...
##  $ total_eggs               : num  14 82 64 62 57 72 62 77 6 1 ...
##  $ Percent_eggs_day3        : num  1 0.878 1 0.984 0.93 ...
##  $ Percent_eggs_day4        : num  0 0.122 0 0.0161 0.0702 ...
##  $ egg_binary               : num  1 1 1 1 1 1 1 1 1 1 ...
##  $ egg_binary_day3          : num  1 1 1 1 1 0 1 1 1 1 ...
##  $ egg_binary_day4          : num  0 1 0 1 1 1 0 1 0 0 ...
##  $ larvae_binary            : num  0 0 0 0 0 NA 1 1 0 0 ...
##  $ Percent_eggshatchedtolarv: num  0 0 0 0 0 ...
##  $ Days_to_death_post_BM    : 'difftime' num  18 12 8 17 ...
##   ..- attr(*, "units")= chr "days"
##  $ Age_of_death             : 'difftime' num  21 15 11 20 ...
##   ..- attr(*, "units")= chr "days"
##  $ Age_of_BM_days           : 'difftime' num  3 3 3 3 ...
##   ..- attr(*, "units")= chr "days"
##  $ Date_of_eclosion         : POSIXct, format: "2024-02-10" "2024-04-13" ...
##  $ days_alive_post_BM       : num  18 12 8 17 8 17 10 3 3 11 ...
##  $ days_alive_post_eclosion : num  21 15 11 20 11 20 13 6 6 14 ...
##  $ log_total_eggs           : num  2.65 4.41 4.16 4.13 4.04 ...
```

```
#use complete cases
############################################
#proportion day 3 vs. day 4

#inspect
hist(Fecundity_data_firstBM_ovipos_narm$Percent_eggs_day3)
```

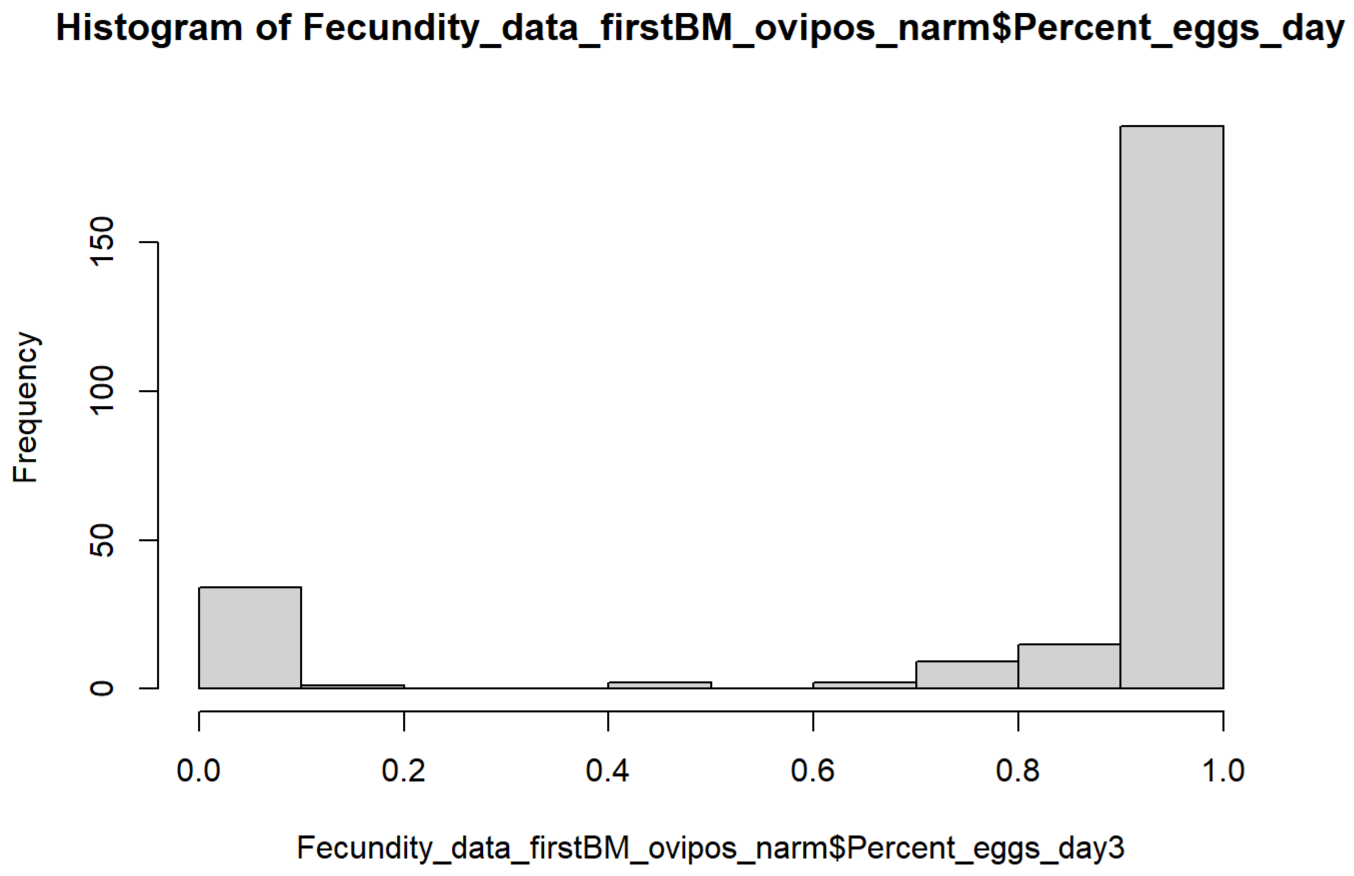


```
hist(Fecundity_data_firstBM_ovipos_narm$Percent_eggs_day4)
```

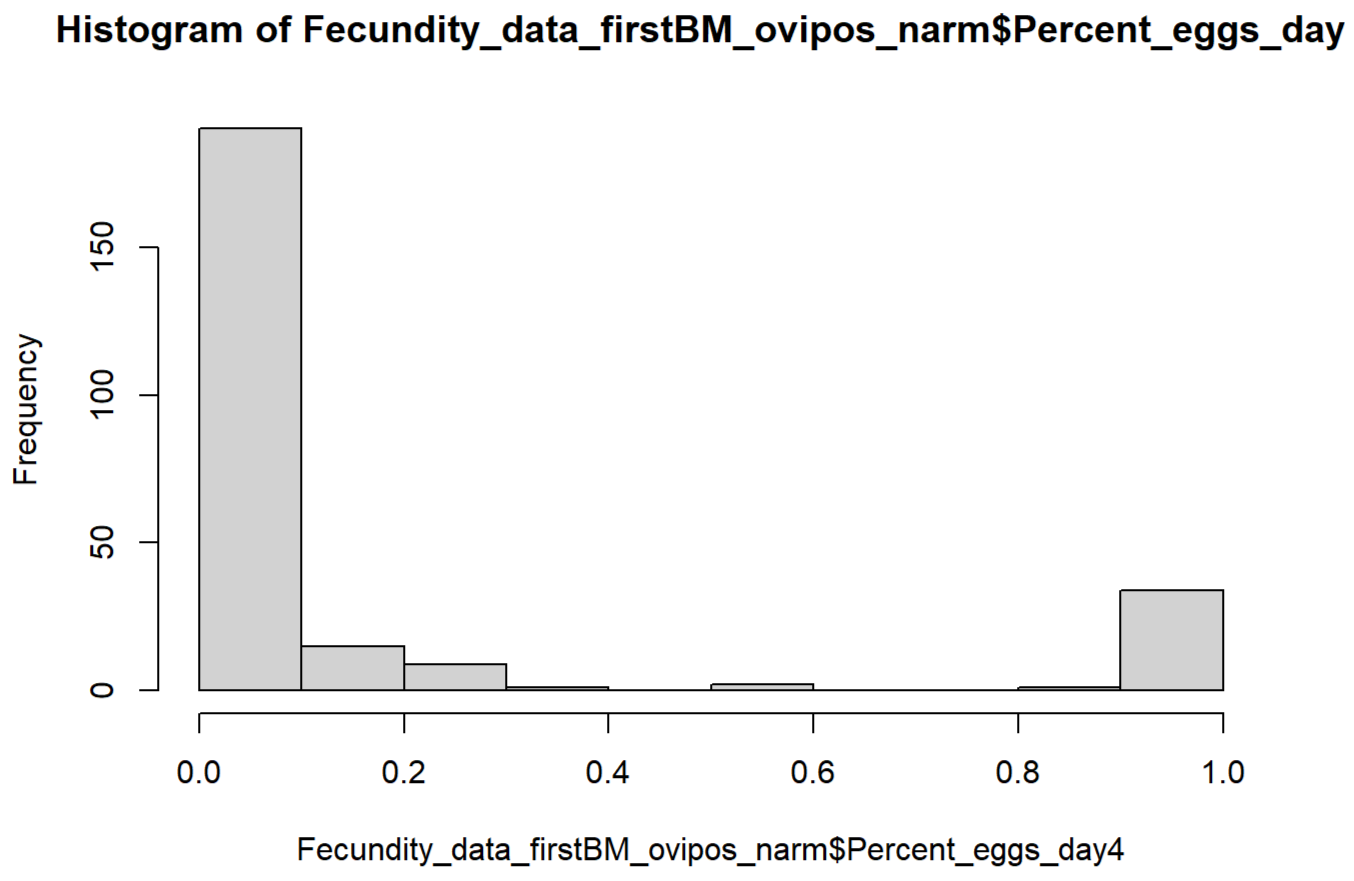

```
str(Fecundity_data_firstBM_ovipos_narm$Percent_eggs_day3)
```

```
##  num [1:252] 1 0.878 1 0.984 0.93 ...
```

```
Fecundity_data_firstBM_ovipos_narm$Percent_eggs_day3transformed <- (((Fecundity_data_firstBM_ovipos_narm$Pe
rcent_eggs_day3)*(218))+0.5)/219
hist(Fecundity_data_firstBM_ovipos_narm$Percent_eggs_day3transformed)
```

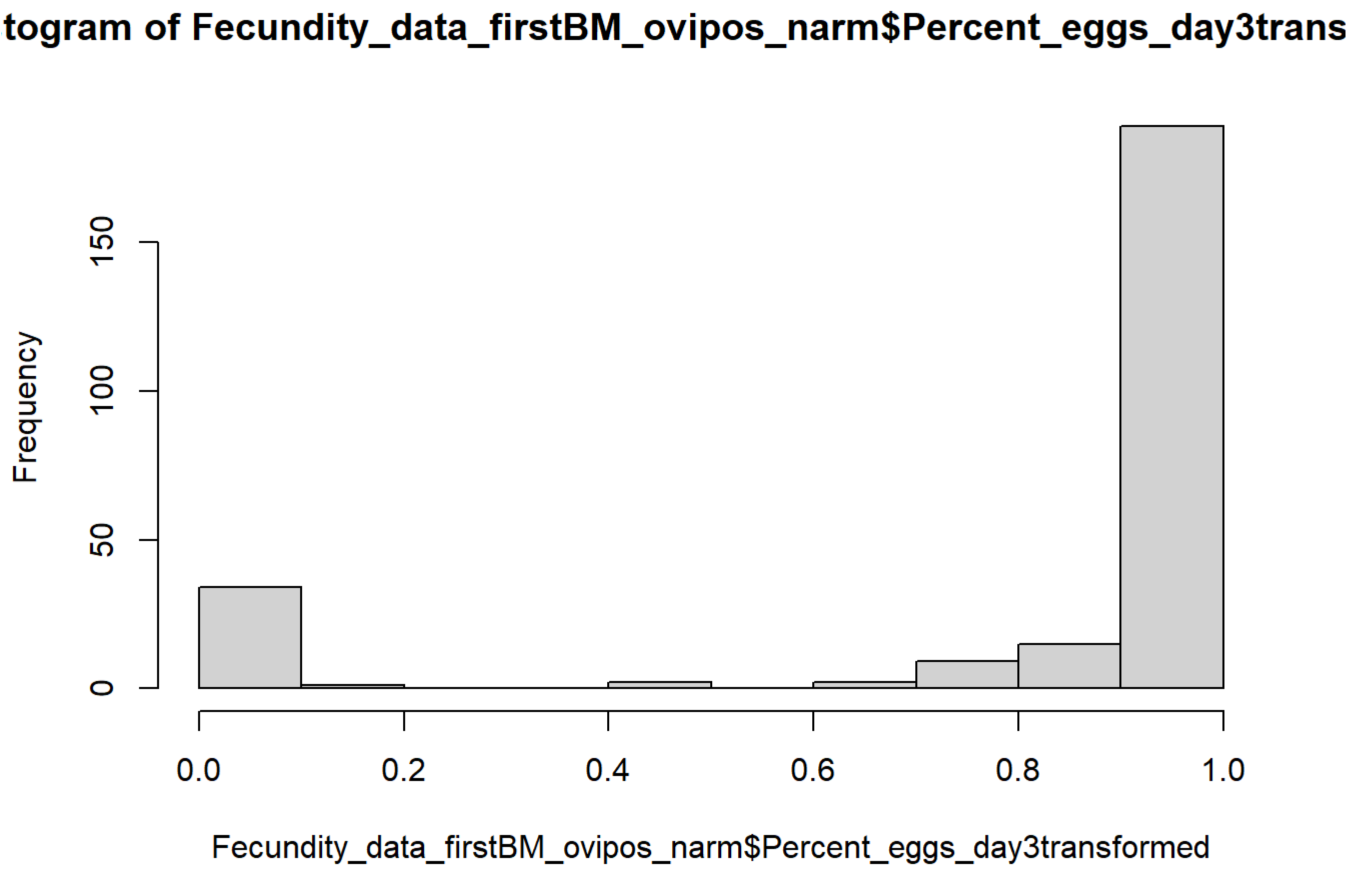


```
model.Proportion_day3.1 = glmmTMB(Percent_eggs_day3transformed ~ Temperature*Age+(1|Trial_number),
                                  data= Fecundity_data_firstBM_ovipos_narm,
                                  family=beta_family(link="logit"))



plot(simulateResiduals(model.Proportion_day3.1))
```

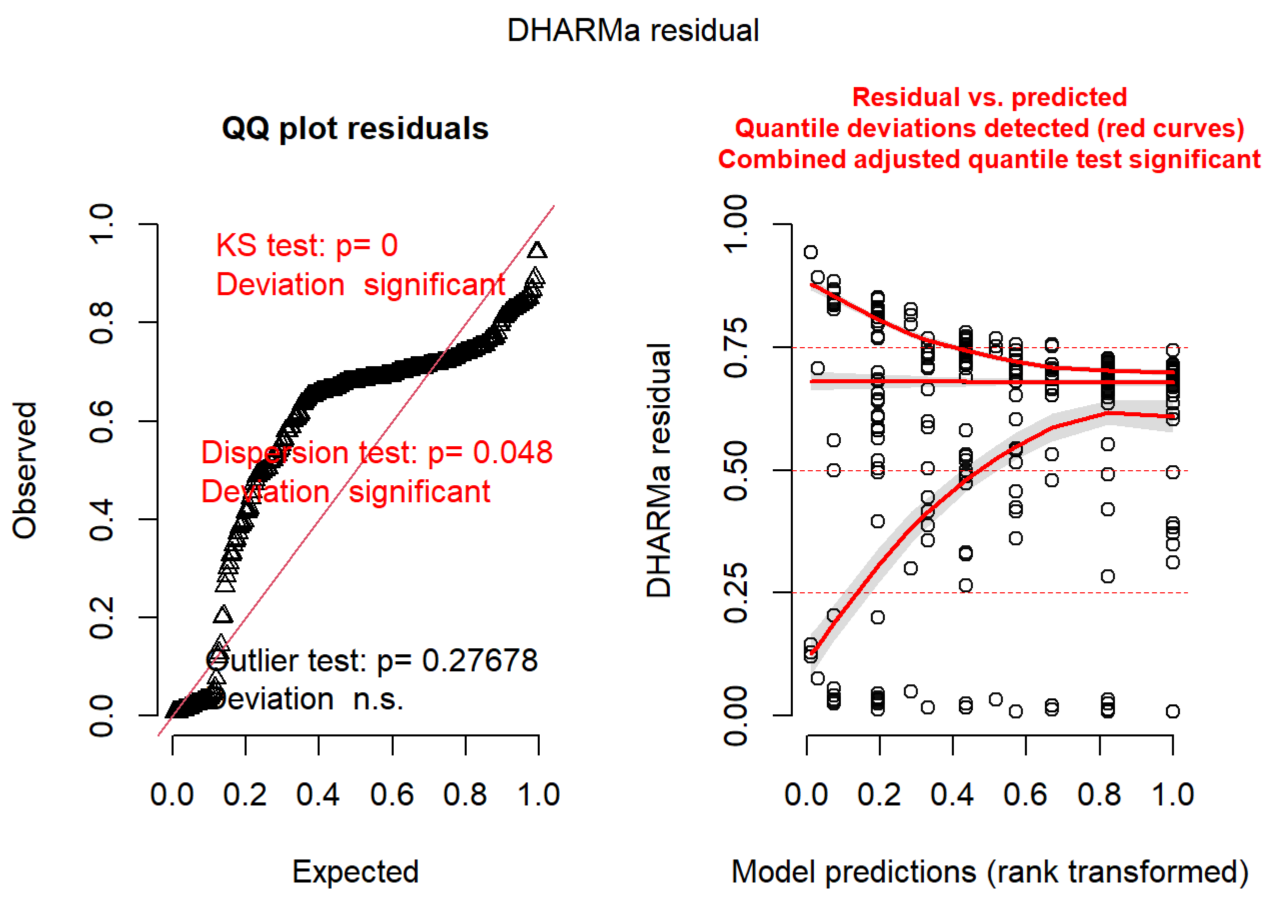


```
plot(fitted(model.Proportion_day3.1),
     residuals(model.Proportion_day3.1))
```

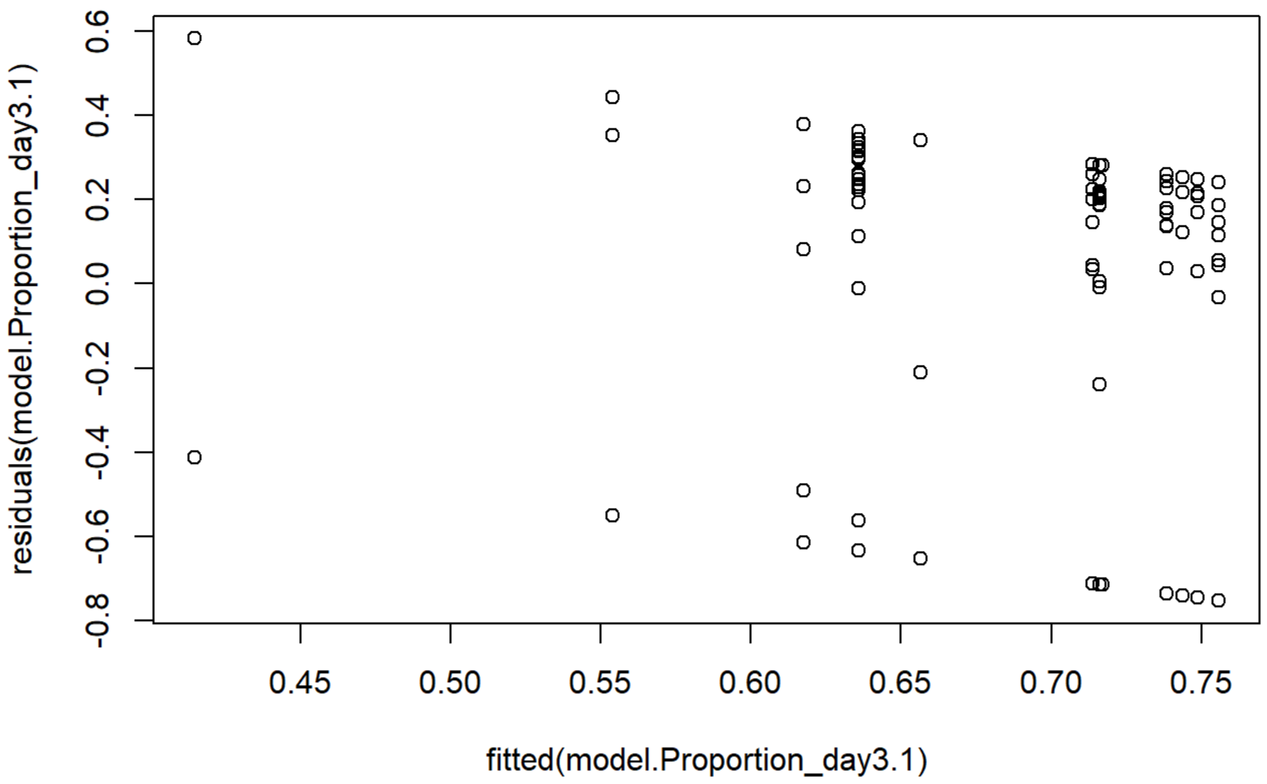

```
#residuals are bad.
#change strategy:
#make temp and age a single group and then do non-parametric kruskal wallis test
Fecundity_data_firstBM_ovipos_narm$group <- paste(Fecundity_data_firstBM_ovipos_narm$Temperature, Fecundity
_data_firstBM_ovipos_narm$Age, sep="_")

#PERCENT EGGS DAY 3
#check to see if there are differences among groups using rank-based kruskal wallis test:
kruskal.test(Fecundity_data_firstBM_ovipos_narm$Percent_eggs_day3,
             Fecundity_data_firstBM_ovipos_narm$group)
```

```
##
##  Kruskal-Wallis rank sum test
##
## data:  Fecundity_data_firstBM_ovipos_narm$Percent_eggs_day3 and Fecundity_data_firstBM_ovipos_narm$group
## Kruskal-Wallis chi-squared = 26.749, df = 11, p-value = 0.005014
```

```
#save the chi squared test
sink("Oviposition_eggsandlarvae/Kruskaltest_BM1percentday3.txt")
kruskal.test(Fecundity_data_firstBM_ovipos_narm$Percent_eggs_day3,
             Fecundity_data_firstBM_ovipos_narm$group)
sink()

#Dunn's post hoc tests for groups:
Dunns_Pday3_temp_groupedbyage <- Fecundity_data_firstBM_ovipos_narm %>%
  group_by(Age)%>%
  dunn_test(Percent_eggs_day3 ~ Temperature)
Dunns_Pday3_temp_groupedbyage <- as.data.frame(Dunns_Pday3_temp_groupedbyage)
write_xlsx(Dunns_Pday3_temp_groupedbyage,"Dunns_Pday3_temp_groupedbyage.xlsx")

Dunns_Pday3_age_groupedbytemp <- Fecundity_data_firstBM_ovipos_narm %>%
  group_by(Temperature)%>%
  dunn_test(Percent_eggs_day3 ~ Age)
Dunns_Pday3_age_groupedbytemp <- as.data.frame(Dunns_Pday3_age_groupedbytemp)
write_xlsx(Dunns_Pday3_age_groupedbytemp,"Dunns_Pday3_age_groupedbytemp.xlsx")



#main effects: no differences
Dunns_Pday3_main_temp <- Fecundity_data_firstBM_ovipos_narm %>%
  dunn_test(Percent_eggs_day3 ~ Temperature)
Dunns_Pday3_main_temp <- as.data.frame(Dunns_Pday3_main_temp)

Dunns_Pday3_main_age <- Fecundity_data_firstBM_ovipos_narm %>%
  dunn_test(Percent_eggs_day3 ~ Age)
Dunns_Pday3_main_age <-as.data.frame(Dunns_Pday3_main_age)

write_xlsx(Dunns_Pday3_main_temp, "Dunns_Pday3_main_temp.xlsx")
write_xlsx(Dunns_Pday3_main_age, "Dunns_Pday3_main_age.xlsx")
```

Code for Fig 6 - egg hatching success: percent eggs laid on day 3 that hatch on day 4; &

Fertility (number of larvae on day 4)

Import data and clean it up

```
# clear existing workspace
rm(list = ls(all = TRUE))
graphics.off()
shell("cls")

#set wd to your project folder
getwd() #check working directory
```

```
## [1] "C:/Users/linzm/OneDrive - Vanderbilt/Hillyer_Lab/Blood_feeding_project/Bloodfeeding"
```

```
###########################
#####################
#load libraries needed:
library(readxl)
library(writexl)
library(ggplot2)
library(dplyr)
library(tidyverse)
library(rstatix)
library(car)
library(ggpubr)
library(emmeans)
#######################################################
#import the data and clean it up:
Fecundity_data <- read_xlsx("SupplementaryData1_RawData.xlsx",
                             sheet = "Figs4-9")
Fecundity_data <- as.data.frame(Fecundity_data)
str(Fecundity_data)
```

```
## 'data.frame':    849 obs. of  18 variables:
##  $ ID_overall               : num  1 2 3 4 5 6 7 8 9 10 ...
##  $ Temperature              : num  32 32 32 32 32 32 32 32 32 32 ...
##  $ Age                      : num  3 3 3 3 3 3 3 3 3 3 ...
##  $ ID_per_group             : num  1 2 3 4 5 6 7 8 9 10 ...
##  $ Trial_start_date         : POSIXct, format: "2024-02-13" "2024-02-13" ...
##  $ Trial_number             : num  1 1 1 1 1 1 1 1 1 1 ...
##  $ BM1_Date                 : POSIXct, format: "2024-02-13" "2024-02-13" ...
##  $ Age_of_BM                : num  3 3 3 3 3 3 3 3 3 3 ...
##  $ Bloodmeal_number         : num  1 1 1 1 1 1 1 1 1 1 ...
##  $ Oviposition_positive(y/n): chr  "Y" "Y" "Y" "Y" ...
##  $ Eggs_day3                : chr  "0" "0" "0" "0" ...
##  $ Eggs_day4                : chr  "0" "0" "0" "0" ...
##  $ Larvae_day4              : chr  "0" "0" "0" "0" ...
##  $ Surv_to_eggs(y/n)        : chr  "Y" "Y" "Y" "Y" ...
##  $ Surv_to_larvae(y/n)      : chr  "Y" "Y" "Y" "Y" ...
##  $ Date_of_death            : POSIXct, format: "2024-03-01" "2024-03-07" ...
##  $ Censor                   : num  1 1 1 1 1 1 1 1 1 1 ...
##  $ Notes                    : chr  NA NA NA NA ...
```

```
head(Fecundity_data)
```

```
##   ID_overall Temperature Age ID_per_group Trial_start_date Trial_number
## 1          1          32   3            1       2024-02-13            1
## 2          2          32   3            2       2024-02-13            1
## 3          3          32   3            3       2024-02-13            1
## 4          4          32   3            4       2024-02-13            1
## 5          5          32   3            5       2024-02-13            1
## 6          6          32   3            6       2024-02-13            1
##     BM1_Date Age_of_BM Bloodmeal_number Oviposition_positive(y/n) Eggs_day3
## 1 2024-02-13         3                1                         Y         0
## 2 2024-02-13         3                1                         Y         0
## 3 2024-02-13         3                1                         Y         0
## 4 2024-02-13         3                1                         Y         0
## 5 2024-02-13         3                1                         Y         0
## 6 2024-02-13         3                1                         Y        14
##   Eggs_day4 Larvae_day4 Surv_to_eggs(y/n) Surv_to_larvae(y/n) Date_of_death
## 1         0           0                 Y                   Y    2024-03-01
## 2         0           0                 Y                   Y    2024-03-07
## 3         0           0                 Y                   Y    2024-03-02
## 4         0           0                 Y                   Y    2024-02-26
## 5         0           0                 Y                   Y    2024-02-28
## 6        14           0                 Y                   Y    2024-03-02
##   Censor Notes
## 1      1  <NA>
## 2      1  <NA>
## 3      1  <NA>
## 4      1  <NA>
## 5      1  <NA>
## 6      1  <NA>
```

```
Fecundity_data_numeric <- Fecundity_data

#variables of interest:
Fecundity_data$Temperature <- as.factor(Fecundity_data$Temperature)
Fecundity_data$Age <- as.factor(Fecundity_data$Age)
Fecundity_data$Age_of_BM <- as.numeric(Fecundity_data$Age_of_BM)
Fecundity_data$Bloodmeal_number <- as.factor(Fecundity_data$Bloodmeal_number)

Fecundity_data$Eggs_day3 <- as.numeric(Fecundity_data$Eggs_day3)
```

```
## Warning: NAs introduced by coercion
```

```
Fecundity_data$Eggs_day4 <- as.numeric(Fecundity_data$Eggs_day4)
```

```
## Warning: NAs introduced by coercion
```

```
Fecundity_data$Larvae_day4 <- as.numeric(Fecundity_data$Larvae_day4)
```

```
## Warning: NAs introduced by coercion
```

```r
Fecundity_data$Oviposition_positive <- as.factor(Fecundity_data$Oviposition_positive)
Fecundity_data$`Surv_to_eggs(y/n)` <- as.factor(Fecundity_data$`Surv_to_eggs(y/n)`)
Fecundity_data$`Surv_to_larvae(y/n)` <- as.factor(Fecundity_data$`Surv_to_larvae(y/n)`)

str(Fecundity_data)
```

```
## 'data.frame':    849 obs. of  19 variables:
##  $ ID_overall               : num  1 2 3 4 5 6 7 8 9 10 ...
##  $ Temperature              : Factor w/ 3 levels "27","30","32": 3 3 3 3 3 3 3 3 3 3 ...
##  $ Age                      : Factor w/ 4 levels "3","5","10","15": 1 1 1 1 1 1 1 1 1 1 ...
##  $ ID_per_group             : num  1 2 3 4 5 6 7 8 9 10 ...
##  $ Trial_start_date         : POSIXct, format: "2024-02-13" "2024-02-13" ...
##  $ Trial_number             : num  1 1 1 1 1 1 1 1 1 1 ...
##  $ BM1_Date                 : POSIXct, format: "2024-02-13" "2024-02-13" ...
##  $ Age_of_BM                : num  3 3 3 3 3 3 3 3 3 3 ...
##  $ Bloodmeal_number         : Factor w/ 1 level "1": 1 1 1 1 1 1 1 1 1 1 ...
##  $ Oviposition_positive(y/n): chr  "Y" "Y" "Y" "Y" ...
##  $ Eggs_day3                : num  0 0 0 0 0 14 NA NA NA NA ...
##  $ Eggs_day4                : num  0 0 0 0 0 14 NA NA NA NA ...
##  $ Larvae_day4              : num  0 0 0 0 0 0 0 0 NA NA ...
##  $ Surv_to_eggs(y/n)        : Factor w/ 3 levels "N","NA","Y": 3 3 3 3 3 3 1 1 3 3 ...
##  $ Surv_to_larvae(y/n)      : Factor w/ 3 levels "N","NA","Y": 3 3 3 3 3 3 2 2 3 3 ...
##  $ Date_of_death            : POSIXct, format: "2024-03-01" "2024-03-07" ...
##  $ Censor                   : num  1 1 1 1 1 1 1 1 1 1 ...
##  $ Notes                    : chr  NA NA NA NA ...
##  $ Oviposition_positive     : Factor w/ 2 levels "N","Y": 2 2 2 2 2 2 2 2 1 1 ...
```

```
Fecundity_data <- subset(Fecundity_data, Censor == 1) #get rid of mosquitoes censored out by experimental e
rror (get rid of 0 values; 1 = died naturally)

#calculate the total eggs laid per mosquito:

#need to subtract to find ones only laid on day 4 (exclude day 3 eggs)
Fecundity_data$Eggs_day4 <- (Fecundity_data$Eggs_day4)-(Fecundity_data$Eggs_day3)

#replace negative eggs day 4 values with zero (assume miscounted/eggs degraded and no new eggs laid)
for (row in 1:nrow(Fecundity_data)){
  if (is.na(Fecundity_data$Eggs_day4[row])){
    Fecundity_data$Eggs_day4[row] <- NA #keep NA values
  } else if ((Fecundity_data$Eggs_day4[row] <= 0)){
    Fecundity_data$Eggs_day4[row] <- 0
  }
}

#total eggs addition:
Fecundity_data$total_eggs <- (Fecundity_data$Eggs_day3)+(Fecundity_data$Eggs_day4)

#percents:
Fecundity_data$Percent_eggs_day3 <- (Fecundity_data$Eggs_day3) / (Fecundity_data$total_eggs)
Fecundity_data$Percent_eggs_day4 <- (Fecundity_data$Eggs_day4) / (Fecundity_data$total_eggs)

# decide if each mosquito laid eggs and on what day
#day 3
for (row in 1:nrow(Fecundity_data)){
  if(!is.na(Fecundity_data$Eggs_day3[row])){
    if (Fecundity_data$Eggs_day3[row] > 0){
      Fecundity_data$egg_binary[row] = 1
      Fecundity_data$egg_binary_day3[row] = 1
    }
    else {
      Fecundity_data$egg_binary[row] = 0
      Fecundity_data$egg_binary_day3[row] = 0
    }
  }
  else if (is.na(Fecundity_data$Eggs_day3[row])){
    Fecundity_data$egg_binary[row] = NA
    Fecundity_data$egg_binary_day3[row] = NA
  }
}

#day 4
for (row in 1:nrow(Fecundity_data)){
  if(!is.na(Fecundity_data$Eggs_day4[row])){
    if (Fecundity_data$Eggs_day4[row] >0){
      Fecundity_data$egg_binary[row] = 1
      Fecundity_data$egg_binary_day4[row] = 1
    }
    else{
      Fecundity_data$egg_binary_day4[row] = 0
    }
  }
  else if (is.na(Fecundity_data$Eggs_day4[row])){
    Fecundity_data$egg_binary_day4[row] = NA
```

```
  }
}

# decide if each mosquito had larvae
for (row in 1:nrow(Fecundity_data)){
  if(!is.na(Fecundity_data$Larvae_day4[row])){
    if (Fecundity_data$Larvae_day4[row] >0){
      Fecundity_data$larvae_binary[row] = 1
    }
    else{
      Fecundity_data$larvae_binary[row] = 0
    }
  }
  else{
    Fecundity_data$larvae_binary[row] = NA
  }
}

#percents:
Fecundity_data$Percent_eggshatchedtolarv <- (Fecundity_data$Larvae_day4) / (Fecundity_data$Eggs_day3)


#survival:
#calculate:
Fecundity_data$Days_to_death_post_BM <- Fecundity_data$Date_of_death - Fecundity_data$Trial_start_date
Fecundity_data$Age_of_death <- Fecundity_data$Age_of_BM + Fecundity_data$Days_to_death_post_BM

Fecundity_data <-
  Fecundity_data %>%
  mutate(
    Age_of_BM_days = as.difftime(Age_of_BM, unit="days")
  )
Fecundity_data$Date_of_eclosion <- Fecundity_data$Trial_start_date - (Fecundity_data$Age_of_BM_days)

library(lubridate)
Fecundity_data <-
  Fecundity_data %>%
  mutate(
    days_alive_post_BM = as.duration(Trial_start_date %--% Date_of_death) / ddays(1),
    days_alive_post_eclosion = as.duration(Date_of_eclosion %--% Date_of_death) / ddays(1),
  )

str(Fecundity_data)
```

```
## 'data.frame':    842 obs. of  33 variables:
##  $ ID_overall               : num  1 2 3 4 5 6 7 8 9 10 ...
##  $ Temperature              : Factor w/ 3 levels "27","30","32": 3 3 3 3 3 3 3 3 3 3 ...
##  $ Age                      : Factor w/ 4 levels "3","5","10","15": 1 1 1 1 1 1 1 1 1 1 ...
##  $ ID_per_group             : num  1 2 3 4 5 6 7 8 9 10 ...
##  $ Trial_start_date         : POSIXct, format: "2024-02-13" "2024-02-13" ...
##  $ Trial_number             : num  1 1 1 1 1 1 1 1 1 1 ...
##  $ BM1_Date                 : POSIXct, format: "2024-02-13" "2024-02-13" ...
##  $ Age_of_BM                : num  3 3 3 3 3 3 3 3 3 3 ...
##  $ Bloodmeal_number         : Factor w/ 1 level "1": 1 1 1 1 1 1 1 1 1 1 ...
##  $ Oviposition_positive(y/n): chr  "Y" "Y" "Y" "Y" ...
##  $ Eggs_day3                : num  0 0 0 0 0 14 NA NA NA NA ...
##  $ Eggs_day4                : num  0 0 0 0 0 0 NA NA NA NA ...
##  $ Larvae_day4              : num  0 0 0 0 0 0 0 0 NA NA ...
##  $ Surv_to_eggs(y/n)        : Factor w/ 3 levels "N","NA","Y": 3 3 3 3 3 3 1 1 3 3 ...
##  $ Surv_to_larvae(y/n)      : Factor w/ 3 levels "N","NA","Y": 3 3 3 3 3 3 2 2 3 3 ...
##  $ Date_of_death            : POSIXct, format: "2024-03-01" "2024-03-07" ...
##  $ Censor                   : num  1 1 1 1 1 1 1 1 1 1 ...
##  $ Notes                    : chr  NA NA NA NA ...
##  $ Oviposition_positive     : Factor w/ 2 levels "N","Y": 2 2 2 2 2 2 2 2 1 1 ...
##  $ total_eggs               : num  0 0 0 0 0 14 NA NA NA NA ...
##  $ Percent_eggs_day3        : num  NaN NaN NaN NaN NaN 1 NA NA NA NA ...
##  $ Percent_eggs_day4        : num  NaN NaN NaN NaN NaN 0 NA NA NA NA ...
##  $ egg_binary               : num  0 0 0 0 0 1 NA NA NA NA ...
##  $ egg_binary_day3          : num  0 0 0 0 0 1 NA NA NA NA ...
##  $ egg_binary_day4          : num  0 0 0 0 0 0 NA NA NA NA ...
##  $ larvae_binary            : num  0 0 0 0 0 0 0 0 NA NA ...
##  $ Percent_eggshatchedtolarv: num  NaN NaN NaN NaN NaN 0 NA NA NA NA ...
##  $ Days_to_death_post_BM    : 'difftime' num  17 23 18 13 ...
##   ..- attr(*, "units")= chr "days"
##  $ Age_of_death             : 'difftime' num  20 26 21 16 ...
##   ..- attr(*, "units")= chr "days"
##  $ Age_of_BM_days           : 'difftime' num  3 3 3 3 ...
##   ..- attr(*, "units")= chr "days"
##  $ Date_of_eclosion         : POSIXct, format: "2024-02-10" "2024-02-10" ...
##  $ days_alive_post_BM       : num  17 23 18 13 15 18 3 3 12 11 ...
##  $ days_alive_post_eclosion : num  20 26 21 16 18 21 6 6 15 14 ...
```

```
Fecundity_data_firstBM <- subset(Fecundity_data, Bloodmeal_number == 1)
#subset by oviposition positive and negative:
Fecundity_data_ovipos <- subset(Fecundity_data, Oviposition_positive== "Y")
Fecundity_data_ovineg <- subset(Fecundity_data, Oviposition_positive== "N")

####
#Here we filter to only look at mosquitoes that laid eggs day 3 to hatch larvae day 4. This will come after
#the analysis for likelihood of egg laying.
Fecundity_data_ovipos_eggsgreaterthanzero <- subset(Fecundity_data_ovipos,Eggs_day3 > 0)
Fecundity_data_ovipos <- Fecundity_data_ovipos_eggsgreaterthanzero

################################################
```

Calculate summary stats:

```
library(dplyr)
#calculate summary stats:
Summary_Fecundity_data_ovipos <- Fecundity_data_ovipos %>%
  group_by(Temperature,Age,Oviposition_positive) %>%
  dplyr::summarise(mean_eggsday3 = mean(Eggs_day3, na.rm = TRUE),
                   mean_eggsday4 = mean(Eggs_day4, na.rm = TRUE),
                   mean_totaleggs= mean(total_eggs, na.rm = TRUE),
                   mean_percenteggs_day3 = mean(Percent_eggs_day3, na.rm = TRUE), #% of total eggs laid on
day 3
                   mean_percenteggs_day4 = mean(Percent_eggs_day4, na.rm = TRUE),#% of total eggs laid on d
ay 4
                   mean_larvae_day4 = mean(Larvae_day4, na.rm = TRUE),
                   mean_Percent_eggshatchedtolarv = mean(Percent_eggshatchedtolarv, na.rm = TRUE), #of the
day3 eggs, what percent hatched?
                   #binary proportions:
                   sum_egg_binary = sum(egg_binary, na.rm = TRUE), #gives total number of mosquitoes per gr
oup that laid eggs
                   sum_surv_to_day3 = sum(`Surv_to_eggs(y/n)`=="Y"), #gives total number of mosquitoes per
group that survived to day 3 post BM
                   sum_surv_to_day4 = sum(`Surv_to_larvae(y/n)`=="Y"), #gives total number of mosquitoes pe
r group that survived to day 4 post BM
                   sum_egg_binary_day3 = sum(egg_binary_day3, na.rm = TRUE), #number of eggs laid on day 3
                   sum_egg_binary_day4 = sum(egg_binary_day4, na.rm = TRUE), #number of eggs laid on day 4
                   sum_larvae_binary = sum(larvae_binary, na.rm = TRUE), #number of larvae counted on day 4
                   proportion_binaryegg = (sum_egg_binary / n()), #what proportion laid any number of eggs?
#remove NAs, but keep all mosquitoes bc some laid eggs even if dead on day 3 count
                   proportion_binaryegg_day3 = (sum_egg_binary_day3 / sum_egg_binary), #what proportion lai
d eggs on day 3, out of ones that laid eggs?
                   proportion_binaryegg_day4 = (sum_egg_binary_day4 / sum_egg_binary), #what proportion lai
d eggs on day 4, out of ones that laid eggs?
                   proportion_binarylarvae = (sum_larvae_binary / sum_egg_binary_day3), #of ones that had e
ggs on day 3, what proportion had any # of larvae hatch?
                   #sample sizes
                   n_mosquitoes = n(), #total n
                   #standard errors
                   SE_eggsday3 = sd(Eggs_day3,na.rm = TRUE)/sqrt(n()),
                   SE_eggsday4 = sd(Eggs_day4,na.rm = TRUE)/sqrt(n()),
                   SE_totaleggs = sd(total_eggs,na.rm = TRUE)/sqrt(n()),
                   SE_percenteggs_day3 = sd(Percent_eggs_day3,na.rm = TRUE)/sqrt(n()),
                   SE_percenteggs_day4 = sd(Percent_eggs_day4,na.rm = TRUE)/sqrt(n()),
                   SE_larvae_day4 = sd(Larvae_day4,na.rm = TRUE)/sqrt(n()),
                   SE_percenteggshatched = sd(Percent_eggshatchedtolarv,na.rm = TRUE)/sqrt(n()))
```

```
## `summarise()` has grouped output by 'Temperature', 'Age'. You can override
## using the `.groups` argument.
```

```
Summary_Fecundity_data_ovipos <- as.data.frame(Summary_Fecundity_data_ovipos)

str(Summary_Fecundity_data_ovipos)
```

```
## 'data.frame':	12 obs. of  28 variables:
##  $ Temperature                   : Factor w/ 3 levels "27","30","32": 1 1 1 1 2 2 2 2 3 3 ...
##  $ Age                           : Factor w/ 4 levels "3","5","10","15": 1 2 3 4 1 2 3 4 1 2 ...
##  $ Oviposition_positive          : Factor w/ 2 levels "N","Y": 2 2 2 2 2 2 2 2 2 2 ...
##  $ mean_eggsday3                 : num  54.2 53.6 36.6 15.5 46.6 ...
##  $ mean_eggsday4                 : num  2.4 0.452 1.353 1.538 3 ...
##  $ mean_totaleggs                : num  56.6 54.1 38.9 17 49.6 ...
##  $ mean_percenteggs_day3         : num  0.965 0.991 0.972 0.898 0.945 ...
##  $ mean_percenteggs_day4         : num  0.0352 0.0089 0.0277 0.1019 0.0554 ...
##  $ mean_larvae_day4              : num  0.85 22.5 17.275 0.385 22 ...
##  $ mean_Percent_eggshatchedtolarv: num  0.0123 0.3888 0.4327 0.0142 0.3193 ...
##  $ sum_egg_binary                : num  20 44 40 13 30 31 22 2 15 4 ...
##  $ sum_surv_to_day3              : int  19 40 37 9 26 29 18 1 15 4 ...
##  $ sum_surv_to_day4              : int  16 36 29 6 21 28 17 1 13 4 ...
##  $ sum_egg_binary_day3           : num  20 44 40 13 30 31 22 2 15 4 ...
##  $ sum_egg_binary_day4           : num  8 4 6 3 11 17 2 1 7 1 ...
##  $ sum_larvae_binary             : num  3 29 28 1 16 22 15 0 2 0 ...
##  $ proportion_binaryegg          : num  1 1 1 1 1 1 1 1 1 1 ...
##  $ proportion_binaryegg_day3     : num  1 1 1 1 1 1 1 1 1 1 ...
##  $ proportion_binaryegg_day4     : num  0.4 0.0909 0.15 0.2308 0.3667 ...
##  $ proportion_binarylarvae       : num  0.15 0.6591 0.7 0.0769 0.5333 ...
##  $ n_mosquitoes                  : int  20 44 40 13 30 31 22 2 15 4 ...
##  $ SE_eggsday3                   : num  6.09 2.46 2.26 3.22 5.25 ...
##  $ SE_eggsday4                   : num  0.958 0.273 0.524 1.084 0.992 ...
##  $ SE_totaleggs                  : num  6.27 2.48 2.37 2.98 5.34 ...
##  $ SE_percenteggs_day3           : num  0.01373 0.00548 0.0108 0.06902 0.02108 ...
##  $ SE_percenteggs_day4           : num  0.01373 0.00548 0.0108 0.06902 0.02108 ...
##  $ SE_larvae_day4                : num  0.549 3.311 2.538 0.385 5.064 ...
##  $ SE_percenteggshatched         : num  0.0072 0.0513 0.057 0.0142 0.0675 ...
```

```
write_xlsx(Summary_Fecundity_data_ovipos, "Oviposition_eggsandlarvae/Summary_Fecundity_data_ovipos.xlsx")



Summary_Fecundity_data_ovipos_proportions <- Fecundity_data_ovipos %>% #Fecundity_data_firstBM_ovipos %>%
  group_by(Temperature,Age,Oviposition_positive,Trial_number) %>%
  dplyr::summarise(#binary proportions:
    sum_egg_binary = sum(egg_binary, na.rm = TRUE), #gives total number of mosquitoes per group that laid e
ggs
    sum_surv_to_day3 = sum(`Surv_to_eggs(y/n)`=="Y"), #gives total number of mosquitoes per group that surv
ived to day 3 post BM
    sum_surv_to_day4 = sum(`Surv_to_larvae(y/n)`=="Y"), #gives total number of mosquitoes per group that su
rvived to day 4 post BM
    sum_egg_binary_day3 = sum(egg_binary_day3, na.rm = TRUE), #number of eggs laid on day 3
    sum_egg_binary_day4 = sum(egg_binary_day4, na.rm = TRUE), #number of eggs laid on day 4
    sum_larvae_binary = sum(larvae_binary, na.rm = TRUE), #number of larvae counted on day 4
    proportion_binaryegg = (sum_egg_binary / n()), #what proportion laid any number of eggs? #remove NAs, b
ut keep all mosquitoes bc some laid eggs even if dead on day 3 count
    proportion_binaryegg_day3 = (sum_egg_binary_day3 / sum_egg_binary), #what proportion laid eggs on day
3, out of ones that laid eggs?
    proportion_binaryegg_day4 = (sum_egg_binary_day4 / sum_egg_binary), #what proportion laid eggs on day
4, out of ones that laid eggs?
    proportion_binarylarvae = (sum_larvae_binary / sum_egg_binary_day3), #of ones that had eggs on day 3, w
hat proportion had any # of larvae hatch?
    proportion_surv_to_day3 = (sum_surv_to_day3 /n()),
    proportion_surv_to_day4 = (sum_surv_to_day4 /n()),
    #sample sizes
    n_mosquitoes = n()) #total n
```

```
## `summarise()` has grouped output by 'Temperature', 'Age',
## 'Oviposition_positive'. You can override using the `.groups` argument.
```

```
#standard errors

Summary_Fecundity_data_ovipos_proportions <- as.data.frame(Summary_Fecundity_data_ovipos_proportions)
write_xlsx(Summary_Fecundity_data_ovipos_proportions, "Oviposition_eggsandlarvae/Summary_Fecundity_data_ovi
pos_proportions.xlsx")

Summary_Fecundity_data_ovipos_proportions_means <- Summary_Fecundity_data_ovipos_proportions %>% #Fecundity
_data_firstBM_ovipos %>%
  group_by(Temperature,Age,Oviposition_positive) %>%
  dplyr::summarise(#binary proportions:
    mean_proportion_binaryegg = mean(proportion_binaryegg,na.rm = TRUE), #what proportion laid any number o
f eggs? #remove NAs, but keep all mosquitoes bc some laid eggs even if dead on day 3 count
    mean_proportion_binaryegg_day3 = mean(proportion_binaryegg_day3,na.rm = TRUE), #what proportion laid eg
gs on day 3, out of ones that laid eggs?
    mean_proportion_binaryegg_day4 = mean(proportion_binaryegg_day4,na.rm = TRUE), #what proportion laid eg
gs on day 4, out of ones that laid eggs?
    mean_proportion_binarylarvae = mean(proportion_binarylarvae,na.rm = TRUE), #of ones that had eggs on da
y 3, what proportion had any # of larvae hatch?
    mean_proportion_surv_to_day3 = mean(proportion_surv_to_day3,na.rm = TRUE),
    mean_proportion_surv_to_day4 = mean(proportion_surv_to_day4,na.rm = TRUE),
    #sample sizes
    n_mosquitoes = sum(n_mosquitoes),
    n_trials = n(),
    SE_proportion_binaryegg = sd(proportion_binaryegg,na.rm = TRUE)/sqrt(n()),
    SE_proportion_binaryegg_day3 = sd(proportion_binaryegg_day3,na.rm = TRUE)/sqrt(n()),
    SE_proportion_binaryegg_day4 = sd(proportion_binaryegg_day4,na.rm = TRUE)/sqrt(n()),
    SE_proportion_binarylarvae = sd(proportion_binarylarvae,na.rm = TRUE)/sqrt(n()),
    SE_proportion_surv_to_day3 = sd(proportion_surv_to_day3,na.rm = TRUE)/sqrt(n()),
    SE_proportion_surv_to_day4 = sd(proportion_surv_to_day4,na.rm = TRUE)/sqrt(n()))
```

```
## `summarise()` has grouped output by 'Temperature', 'Age'. You can override
## using the `.groups` argument.
```

```
Summary_Fecundity_data_ovipos_proportions_means <- as.data.frame(Summary_Fecundity_data_ovipos_proportions_
means)
write_xlsx(Summary_Fecundity_data_ovipos_proportions_means, "Oviposition_eggsandlarvae/Summary_Fecundity_da
ta_ovipos_proportions_means.xlsx")


#TEMP only:
Summary_Fecundity_data_ovipos_TEMP <- Fecundity_data_ovipos %>%
  group_by(Temperature) %>%
  dplyr::summarise(mean_eggsday3 = mean(Eggs_day3, na.rm = TRUE),
                   mean_eggsday4 = mean(Eggs_day4, na.rm = TRUE),
                   mean_totaleggs= mean(total_eggs, na.rm = TRUE),
                   mean_percenteggs_day3 = mean(Percent_eggs_day3, na.rm = TRUE), #% of total eggs laid on 
day 3
                   mean_percenteggs_day4 = mean(Percent_eggs_day4, na.rm = TRUE),#% of total eggs laid on d
ay 4
                   mean_larvae_day4 = mean(Larvae_day4, na.rm = TRUE),
                   mean_Percent_eggshatchedtolarv = mean(Percent_eggshatchedtolarv, na.rm = TRUE), #of the 
day3 eggs, what percent hatched?
                   #binary proportions:
                   sum_egg_binary = sum(egg_binary, na.rm = TRUE), #gives total number of mosquitoes per gr
oup that laid eggs
                   sum_surv_to_day3 = sum(`Surv_to_eggs(y/n)`=="Y"), #gives total number of mosquitoes per 
group that survived to day 3 post BM
                   sum_surv_to_day4 = sum(`Surv_to_larvae(y/n)`=="Y"), #gives total number of mosquitoes pe
r group that survived to day 4 post BM
                   sum_egg_binary_day3 = sum(egg_binary_day3, na.rm = TRUE), #number of eggs laid on day 3
                   sum_egg_binary_day4 = sum(egg_binary_day4, na.rm = TRUE), #number of eggs laid on day 4
                   sum_larvae_binary = sum(larvae_binary, na.rm = TRUE), #number of larvae counted on day 4
                   proportion_binaryegg = (sum_egg_binary / n()), #what proportion laid any number of eggs?
#remove NAs, but keep all mosquitoes bc some laid eggs even if dead on day 3 count
                   proportion_binaryegg_day3 = (sum_egg_binary_day3 / sum_egg_binary), #what proportion lai
d eggs on day 3, out of ones that laid eggs?
                   proportion_binaryegg_day4 = (sum_egg_binary_day4 / sum_egg_binary), #what proportion lai
d eggs on day 4, out of ones that laid eggs?
                   proportion_binarylarvae = (sum_larvae_binary / sum_egg_binary_day3), #of ones that had e
ggs on day 3, what proportion had any # of larvae hatch?
                   #sample sizes
                   n_mosquitoes = n(), #total n
                   #standard errors
                   SE_eggsday3 = sd(Eggs_day3,na.rm = TRUE)/sqrt(n()),
                   SE_eggsday4 = sd(Eggs_day4,na.rm = TRUE)/sqrt(n()),
                   SE_totaleggs = sd(total_eggs,na.rm = TRUE)/sqrt(n()),
                   SE_percenteggs_day3 = sd(Percent_eggs_day3,na.rm = TRUE)/sqrt(n()),
                   SE_percenteggs_day4 = sd(Percent_eggs_day4,na.rm = TRUE)/sqrt(n()),
                   SE_larvae_day4 = sd(Larvae_day4,na.rm = TRUE)/sqrt(n()),
                   SE_percenteggshatched = sd(Percent_eggshatchedtolarv,na.rm = TRUE)/sqrt(n()))

Summary_Fecundity_data_ovipos_TEMP <- as.data.frame(Summary_Fecundity_data_ovipos_TEMP)
write_xlsx(Summary_Fecundity_data_ovipos_TEMP, "Oviposition_eggsandlarvae/Summary_Fecundity_data_ovipos_TEM
P.xlsx")

Summary_Fecundity_data_ovipos_proportions_means_TEMP <- Summary_Fecundity_data_ovipos_proportions %>% #Fecu
ndity_data_firstBM_ovipos %>%
  group_by(Temperature) %>%
  dplyr::summarise(#binary proportions:
```

```
    mean_proportion_binaryegg = mean(proportion_binaryegg,na.rm = TRUE), #what proportion laid any number o
f eggs? #remove NAs, but keep all mosquitoes bc some laid eggs even if dead on day 3 count
    mean_proportion_binaryegg_day3 = mean(proportion_binaryegg_day3,na.rm = TRUE), #what proportion laid eg
gs on day 3, out of ones that laid eggs?
    mean_proportion_binaryegg_day4 = mean(proportion_binaryegg_day4,na.rm = TRUE), #what proportion laid eg
gs on day 4, out of ones that laid eggs?
    mean_proportion_binarylarvae = mean(proportion_binarylarvae,na.rm = TRUE), #of ones that had eggs on da
y 3, what proportion had any # of larvae hatch?
    mean_proportion_surv_to_day3 = mean(proportion_surv_to_day3,na.rm = TRUE),
    mean_proportion_surv_to_day4 = mean(proportion_surv_to_day4,na.rm = TRUE),
    #sample sizes
    n_mosquitoes = sum(n_mosquitoes),
    n_trials = n(),
    SE_proportion_binaryegg = sd(proportion_binaryegg,na.rm = TRUE)/sqrt(n()),
    SE_proportion_binaryegg_day3 = sd(proportion_binaryegg_day3,na.rm = TRUE)/sqrt(n()),
    SE_proportion_binaryegg_day4 = sd(proportion_binaryegg_day4,na.rm = TRUE)/sqrt(n()),
    SE_proportion_binarylarvae = sd(proportion_binarylarvae,na.rm = TRUE)/sqrt(n()),
    SE_proportion_surv_to_day3 = sd(proportion_surv_to_day3,na.rm = TRUE)/sqrt(n()),
    SE_proportion_surv_to_day4 = sd(proportion_surv_to_day4,na.rm = TRUE)/sqrt(n()))


Summary_Fecundity_data_ovipos_proportions_means_TEMP <- as.data.frame(Summary_Fecundity_data_ovipos_proport
ions_means_TEMP)
write_xlsx(Summary_Fecundity_data_ovipos_proportions_means_TEMP, "Oviposition_eggsandlarvae/Summary_Fecundi
ty_data_ovipos_proportions_means_TEMP.xlsx")


#AGE only:
Summary_Fecundity_data_ovipos_AGE <- Fecundity_data_ovipos %>%
  group_by(Age) %>%
  dplyr::summarise(mean_eggsday3 = mean(Eggs_day3, na.rm = TRUE),
                   mean_eggsday4 = mean(Eggs_day4, na.rm = TRUE),
                   mean_totaleggs= mean(total_eggs, na.rm = TRUE),
                   mean_percenteggs_day3 = mean(Percent_eggs_day3, na.rm = TRUE), #% of total eggs laid on
day 3
                   mean_percenteggs_day4 = mean(Percent_eggs_day4, na.rm = TRUE),#% of total eggs laid on d
ay 4
                   mean_larvae_day4 = mean(Larvae_day4, na.rm = TRUE),
                   mean_Percent_eggshatchedtolarv = mean(Percent_eggshatchedtolarv, na.rm = TRUE), #of the
day3 eggs, what percent hatched?
                   #binary proportions:
                   sum_egg_binary = sum(egg_binary, na.rm = TRUE), #gives total number of mosquitoes per gr
oup that laid eggs
                   sum_surv_to_day3 = sum(`Surv_to_eggs(y/n)`=="Y"), #gives total number of mosquitoes per
group that survived to day 3 post BM
                   sum_surv_to_day4 = sum(`Surv_to_larvae(y/n)`=="Y"), #gives total number of mosquitoes pe
r group that survived to day 4 post BM
                   sum_egg_binary_day3 = sum(egg_binary_day3, na.rm = TRUE), #number of eggs laid on day 3
                   sum_egg_binary_day4 = sum(egg_binary_day4, na.rm = TRUE), #number of eggs laid on day 4
                   sum_larvae_binary = sum(larvae_binary, na.rm = TRUE), #number of larvae counted on day 4
                   proportion_binaryegg = (sum_egg_binary / n()), #what proportion laid any number of eggs?
#remove NAs, but keep all mosquitoes bc some laid eggs even if dead on day 3 count
                   proportion_binaryegg_day3 = (sum_egg_binary_day3 / sum_egg_binary), #what proportion lai
d eggs on day 3, out of ones that laid eggs?
                   proportion_binaryegg_day4 = (sum_egg_binary_day4 / sum_egg_binary), #what proportion lai
d eggs on day 4, out of ones that laid eggs?
```

```
                   proportion_binarylarvae = (sum_larvae_binary / sum_egg_binary_day3), #of ones that had e
ggs on day 3, what proportion had any # of larvae hatch?
                   #sample sizes
                   n_mosquitoes = n(), #total n
                   #standard errors
                   SE_eggsday3 = sd(Eggs_day3,na.rm = TRUE)/sqrt(n()),
                   SE_eggsday4 = sd(Eggs_day4,na.rm = TRUE)/sqrt(n()),
                   SE_totaleggs = sd(total_eggs,na.rm = TRUE)/sqrt(n()),
                   SE_percenteggs_day3 = sd(Percent_eggs_day3,na.rm = TRUE)/sqrt(n()),
                   SE_percenteggs_day4 = sd(Percent_eggs_day4,na.rm = TRUE)/sqrt(n()),
                   SE_larvae_day4 = sd(Larvae_day4,na.rm = TRUE)/sqrt(n()),
                   SE_percenteggshatched = sd(Percent_eggshatchedtolarv,na.rm = TRUE)/sqrt(n()))

Summary_Fecundity_data_ovipos_AGE <- as.data.frame(Summary_Fecundity_data_ovipos_AGE)
write_xlsx(Summary_Fecundity_data_ovipos_AGE, "Oviposition_eggsandlarvae/Summary_Fecundity_data_ovipos_AGE.
xlsx")


Summary_Fecundity_data_ovipos_proportions_means_AGE <- Summary_Fecundity_data_ovipos_proportions %>% #Fecun
dity_data_firstBM_ovipos %>%
  group_by(Age) %>%
  dplyr::summarise(#binary proportions:
    mean_proportion_binaryegg = mean(proportion_binaryegg,na.rm = TRUE), #what proportion laid any number o
f eggs? #remove NAs, but keep all mosquitoes bc some laid eggs even if dead on day 3 count
    mean_proportion_binaryegg_day3 = mean(proportion_binaryegg_day3,na.rm = TRUE), #what proportion laid eg
gs on day 3, out of ones that laid eggs?
    mean_proportion_binaryegg_day4 = mean(proportion_binaryegg_day4,na.rm = TRUE), #what proportion laid eg
gs on day 4, out of ones that laid eggs?
    mean_proportion_binarylarvae = mean(proportion_binarylarvae,na.rm = TRUE), #of ones that had eggs on da
y 3, what proportion had any # of larvae hatch?
    mean_proportion_surv_to_day3 = mean(proportion_surv_to_day3,na.rm = TRUE),
    mean_proportion_surv_to_day4 = mean(proportion_surv_to_day4,na.rm = TRUE),
    #sample sizes
    n_mosquitoes = sum(n_mosquitoes),
    n_trials = n(),
    SE_proportion_binaryegg = sd(proportion_binaryegg,na.rm = TRUE)/sqrt(n()),
    SE_proportion_binaryegg_day3 = sd(proportion_binaryegg_day3,na.rm = TRUE)/sqrt(n()),
    SE_proportion_binaryegg_day4 = sd(proportion_binaryegg_day4,na.rm = TRUE)/sqrt(n()),
    SE_proportion_binarylarvae = sd(proportion_binarylarvae,na.rm = TRUE)/sqrt(n()),
    SE_proportion_surv_to_day3 = sd(proportion_surv_to_day3,na.rm = TRUE)/sqrt(n()),
    SE_proportion_surv_to_day4 = sd(proportion_surv_to_day4,na.rm = TRUE)/sqrt(n()))


Summary_Fecundity_data_ovipos_proportions_means_AGE <- as.data.frame(Summary_Fecundity_data_ovipos_proporti
ons_means_AGE)
write_xlsx(Summary_Fecundity_data_ovipos_proportions_means_AGE, "Oviposition_eggsandlarvae/Summary_Fecundit
y_data_ovipos_proportions_means_AGE.xlsx")
```

Plot number of larvae on day 4

```
#basic plots:
#separate for plotting:
Fecundity_data_firstBM_ovipos <- Fecundity_data_ovipos
Summary_Fecundity_data_firstBM_ovipos <- Summary_Fecundity_data_ovipos
Summary_Fecundity_data_firstBM_ovipos_proportions <- Summary_Fecundity_data_ovipos_proportions
Summary_Fecundity_data_firstBM_ovipos_proportions_means <- Summary_Fecundity_data_ovipos_proportions_means
Summary_Fecundity_data_firstBM_ovipos_AGE <- Summary_Fecundity_data_ovipos_AGE
Summary_Fecundity_data_firstBM_ovipos_proportions_means_AGE <- Summary_Fecundity_data_ovipos_proportions_me
ans_AGE
Summary_Fecundity_data_firstBM_ovipos_TEMP <- Summary_Fecundity_data_ovipos_TEMP
Summary_Fecundity_data_firstBM_ovipos_proportions_means_TEMP <-Summary_Fecundity_data_ovipos_proportions_me
ans_TEMP
#######

#number of larvae day 4 (if eggs laid on day 3)

Summary_Fecundity_data_firstBM_ovipos$Age <- factor(Summary_Fecundity_data_firstBM_ovipos$Age,
                                                    labels = c("3 days","5 days","10 days","15 days"))

Summary_Fecundity_data_firstBM_ovipos$Temperature <- factor(Summary_Fecundity_data_firstBM_ovipos$Temperatu
re,
                                                            labels = c("27","30","32"))

Fecundity_data_firstBM_ovipos$Age <- factor(Fecundity_data_firstBM_ovipos$Age,
                                            labels = c("3 days","5 days","10 days","15 days"))

Fecundity_data_firstBM_ovipos$Temperature <- factor(Fecundity_data_firstBM_ovipos$Temperature,
                                                    labels = c("27","30","32"))

BM1_ovipos_meanlarvae_day4 <- Summary_Fecundity_data_firstBM_ovipos %>%
  ggplot(aes(x=Temperature,y=mean_larvae_day4,group=Temperature))+
  geom_bar(aes(fill=Temperature),
           stat = "identity",
           position = position_dodge(1),
           width = 0.8) +
  scale_shape_identity(guide="legend")+
  facet_grid(~Age)+
  geom_errorbar(aes(ymin=(mean_larvae_day4 - SE_larvae_day4),
                  ymax=(mean_larvae_day4 + SE_larvae_day4)),
                width=0.8,position=position_dodge(0.9),
                color="black")+
  ylab(expression("Number of larvae from day 3 eggs"))+
  xlab("Temperature (˚C)") +
  theme_pubr()+
  theme(legend.position = "none")+
  geom_jitter(data=Fecundity_data_firstBM_ovipos, aes(x=Temperature,y=Larvae_day4),
              position = "jitter", na.rm = TRUE,size=0.5)+
  scale_fill_manual(values= c("#4D6FAE","#6F9F51", "#CC763B"))+
  theme(panel.background = element_rect(fill = NA, color = "black"))+
  theme(panel.spacing = unit(0.5, "lines"))
BM1_ovipos_meanlarvae_day4
```

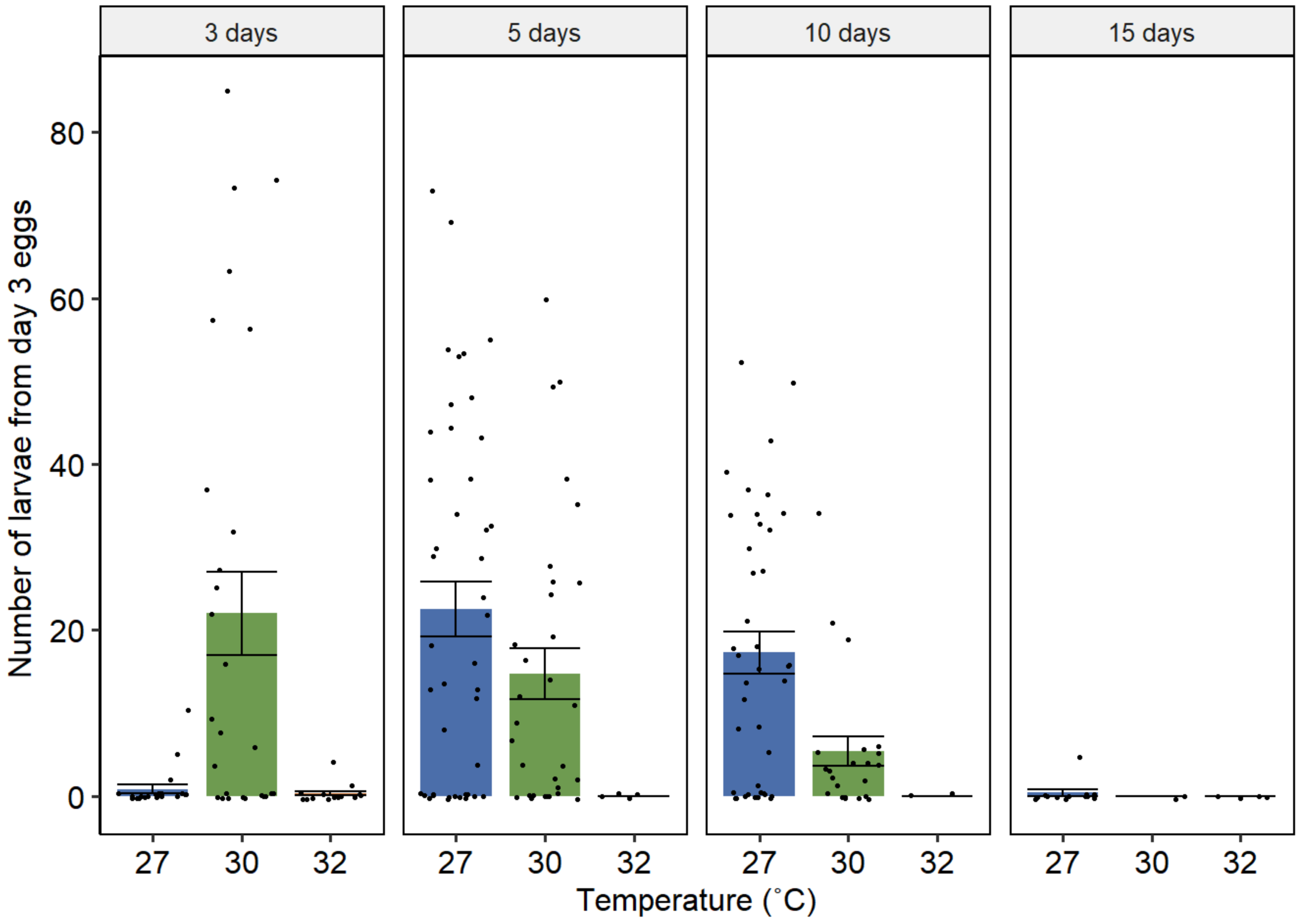

3 days
5 days
10 days
15 days
Number of larvae from day 3 eggs
0
20
40
60
80
27
30
32
Temperature (˚C)

```
ggsave("Oviposition_eggsandlarvae/BM1_ovipos_meanlarvae_day4.pdf", plot=BM1_ovipos_meanlarvae_day4, width =
6, height = 4,
       units = "in",dpi=600)
ggsave("Oviposition_eggsandlarvae/BM1_ovipos_meanlarvae_day4.png", plot=BM1_ovipos_meanlarvae_day4, width =
6, height = 4,
       units = "in",dpi=600)

Summary_Fecundity_data_firstBM_ovipos$Age <- factor(Summary_Fecundity_data_firstBM_ovipos$Age,
                                                    labels = c("3","5","10","15"))
Summary_Fecundity_data_firstBM_ovipos$Temperature <- factor(Summary_Fecundity_data_firstBM_ovipos$Temperatu
re,
                                                            labels = c("27˚C","30˚C","32˚C"))

Fecundity_data_firstBM_ovipos$Age <- factor(Fecundity_data_firstBM_ovipos$Age,
                                            labels = c("3","5","10","15"))
Fecundity_data_firstBM_ovipos$Temperature <- factor(Fecundity_data_firstBM_ovipos$Temperature,
                                                    labels = c("27˚C","30˚C","32˚C"))

BM1_ovipos_meanlarvae_day4_agewithintemp <- Summary_Fecundity_data_firstBM_ovipos %>%
  ggplot(aes(x=Age,y=mean_larvae_day4,group=Age))+
  geom_bar(aes(fill=Age),
           stat = "identity",
           position = position_dodge(1),
           width = 0.8) +
  scale_shape_identity(guide="legend")+
  facet_grid(~Temperature)+
  geom_errorbar(aes(ymin=(mean_larvae_day4 - SE_larvae_day4),
                    ymax=(mean_larvae_day4 + SE_larvae_day4)),
                width=0.8,position=position_dodge(0.9),
                color="black")+
  ylab(expression("Number of larvae from day 3 eggs"))+
  xlab("Age of first BM (days old)") +
  theme_pubr()+
  theme(legend.position = "none")+
  geom_jitter(data=Fecundity_data_firstBM_ovipos, aes(x=Age,y=Larvae_day4),
              position = "jitter", na.rm = TRUE,size=0.5)+
  scale_fill_manual(values= c("#DCD1E9","#BAA4D3","#9776BE","#7549A8"))+
  theme(panel.background = element_rect(fill = NA, color = "black"))+
  theme(panel.spacing = unit(0.5, "lines"))
BM1_ovipos_meanlarvae_day4_agewithintemp
```

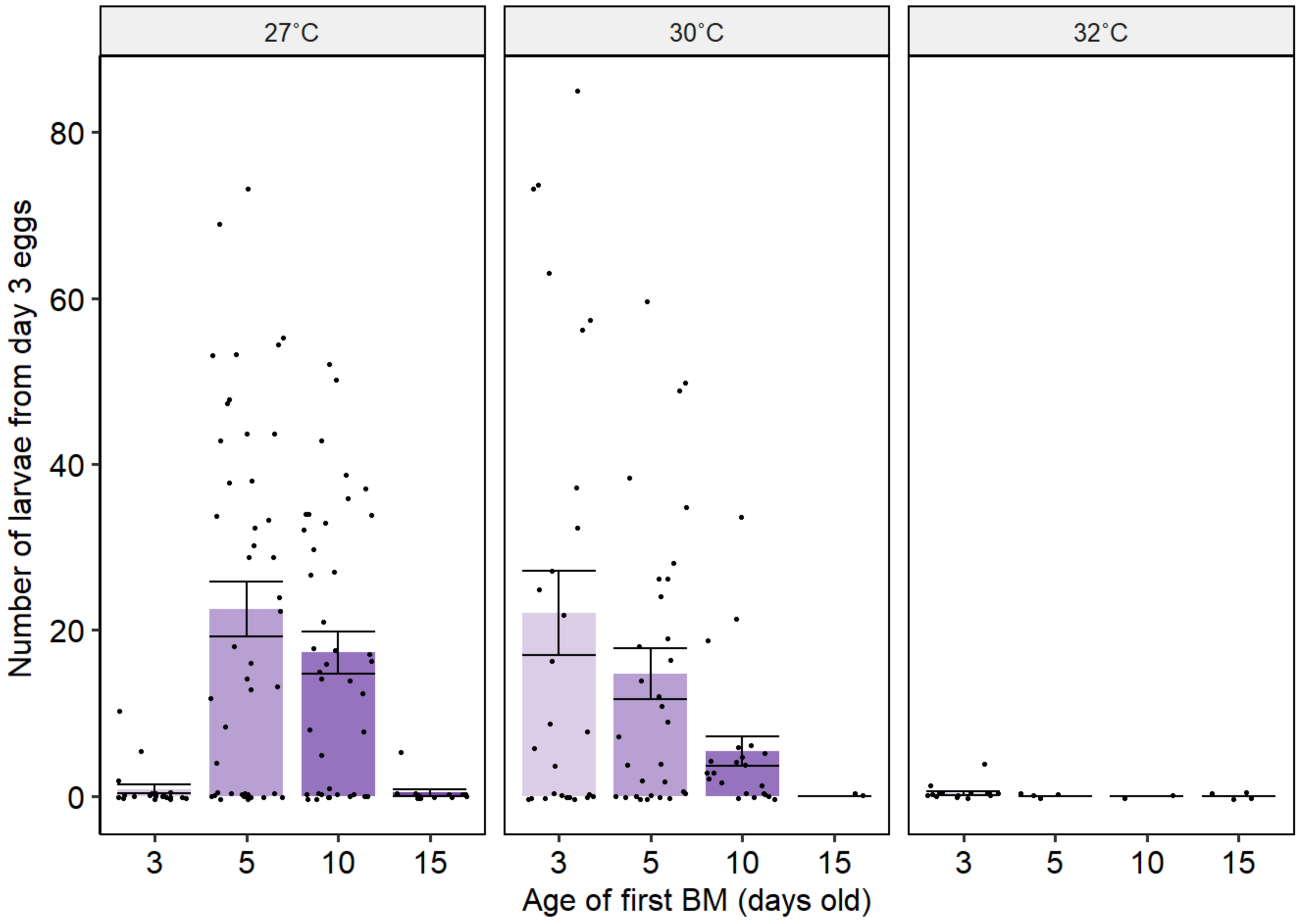


```
ggsave("Oviposition_eggsandlarvae/BM1_ovipos_meanlarvae_day4_agewithintemp.pdf", plot=BM1_ovipos_meanlarvae
_day4_agewithintemp, width = 6, height = 4,
       units = "in",dpi=600)
ggsave("Oviposition_eggsandlarvae/BM1_ovipos_meanlarvae_day4_agewithintemp.png", plot=BM1_ovipos_meanlarvae
_day4_agewithintemp, width = 6, height = 4,
       units = "in",dpi=600)

#raw data interaction plot:
BM1_ovipos_numberlarvae_rawdata_interaction <- Summary_Fecundity_data_firstBM_ovipos%>%
  ggplot()+
  aes(x=Age,y=mean_larvae_day4,group=Temperature)+
  geom_point(aes(shape=Temperature),size=3)+
  geom_line(aes(linetype = Temperature),linewidth=0.6)+
  theme_pubr()+
  scale_shape(labels=c(27,30,32))+
  scale_linetype(labels=c(27,30,32))+
  guides(shape = guide_legend(title = "Temperature (˚C)"),
         linetype = guide_legend(title = "Temperature (˚C)"))+
  xlab("Adult Age (days)") +
  theme(legend.position = "right")+
  scale_x_discrete(labels=c(3,5,10,15))+
  ylab(expression("Number of larvae from day 3 eggs"))+
  theme(panel.background = element_rect(fill = NA, color = "black"))+
  theme(panel.spacing = unit(0.6, "lines"))
BM1_ovipos_numberlarvae_rawdata_interaction
```

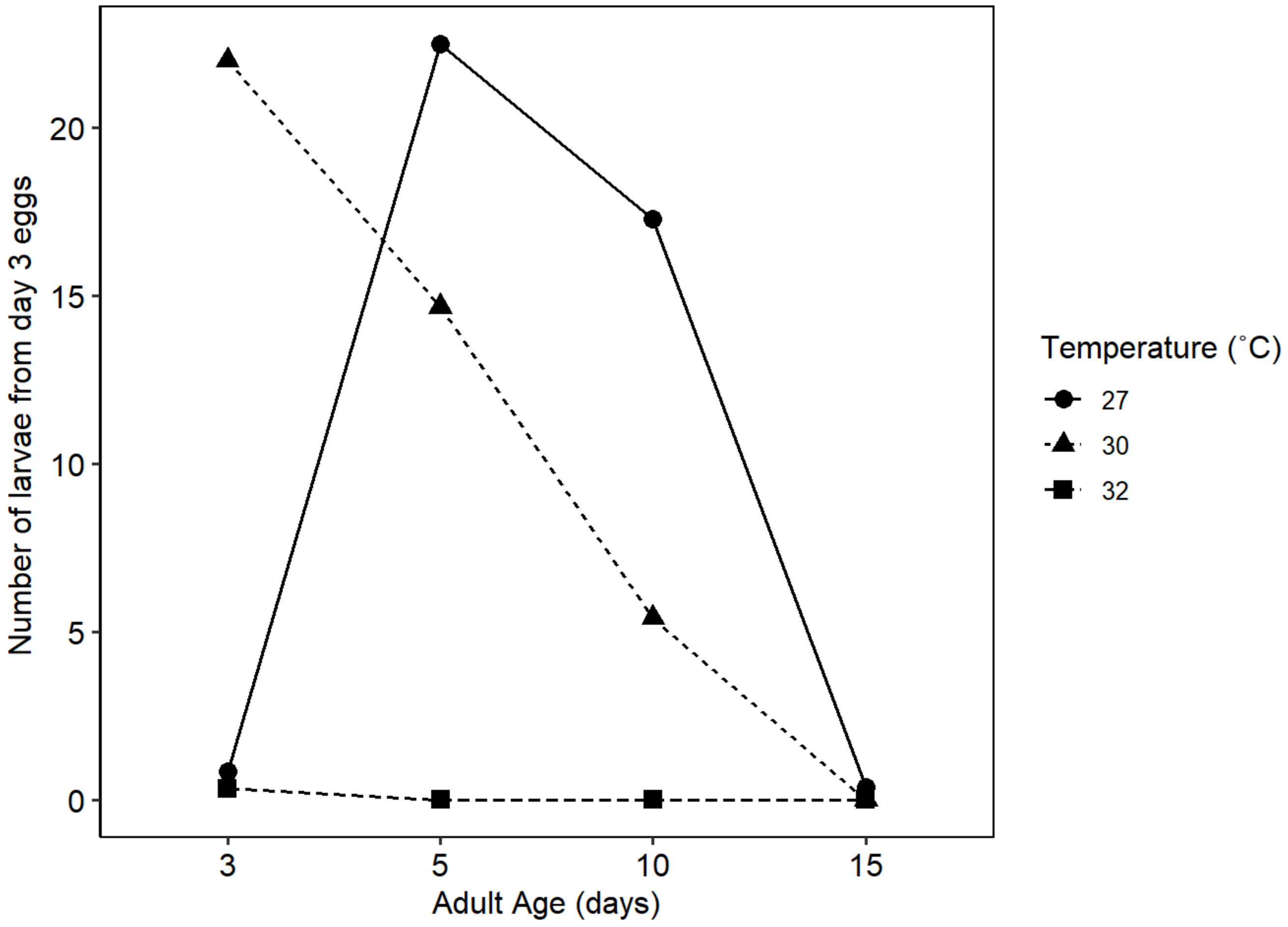
Number of larvae from day 3 eggs
20
15
10
5
0
3
5
10
15
Adult Age (days)
Temperature (˚C)
27
30
32

```r
ggsave("Oviposition_eggsandlarvae/BM1_ovipos_numberlarvae_rawdata_interaction.png",plot=BM1_ovipos_numberla
rvae_rawdata_interaction,width = 5.5, height = 4, units = "in", dpi = 600)
ggsave("Oviposition_eggsandlarvae/BM1_ovipos_numberlarvae_rawdata_interaction.pdf",plot=BM1_ovipos_numberla
rvae_rawdata_interaction,width = 5.5, height = 4, units = "in", dpi = 600)




#TEMP ONLY
Summary_Fecundity_data_firstBM_ovipos_TEMP$Temperature <- factor(Summary_Fecundity_data_firstBM_ovipos_TEMP
$Temperature,
                                                                 labels = c("27","30","32"))
Fecundity_data_firstBM_ovipos$Temperature <- factor(Fecundity_data_firstBM_ovipos$Temperature,
                                                    labels = c("27","30","32"))

BM1_ovipos_meanlarvae_day4_temponly <- Summary_Fecundity_data_firstBM_ovipos_TEMP %>%
  ggplot(aes(x=Temperature,y=mean_larvae_day4,group=Temperature))+
  geom_bar(aes(fill=Temperature),
           stat = "identity",
           position = position_dodge(1),
           width = 0.8) +
  scale_shape_identity(guide="legend")+
  #facet_grid(~Age)+
  geom_errorbar(aes(ymin=(mean_larvae_day4 - SE_larvae_day4),
                    ymax=(mean_larvae_day4 + SE_larvae_day4)),
                width=0.8,position=position_dodge(0.9),
                color="black")+
  ylab(expression("Number of larvae from day 3 eggs"))+
  xlab("Temperature (˚C)") +
  theme_pubr()+
  theme(legend.position = "none")+
  geom_jitter(data=Fecundity_data_firstBM_ovipos, aes(x=Temperature,y=Larvae_day4),#color=ZOI_italic$Techni
cal_Rep,
              position = "jitter", na.rm = TRUE,size=0.5)+
  scale_fill_manual(values= c("#4D6FAE","#6F9F51", "#CC763B"))+
  theme(panel.background = element_rect(fill = NA, color = "black"))+
  theme(panel.spacing = unit(0.5, "lines"))
BM1_ovipos_meanlarvae_day4_temponly
```

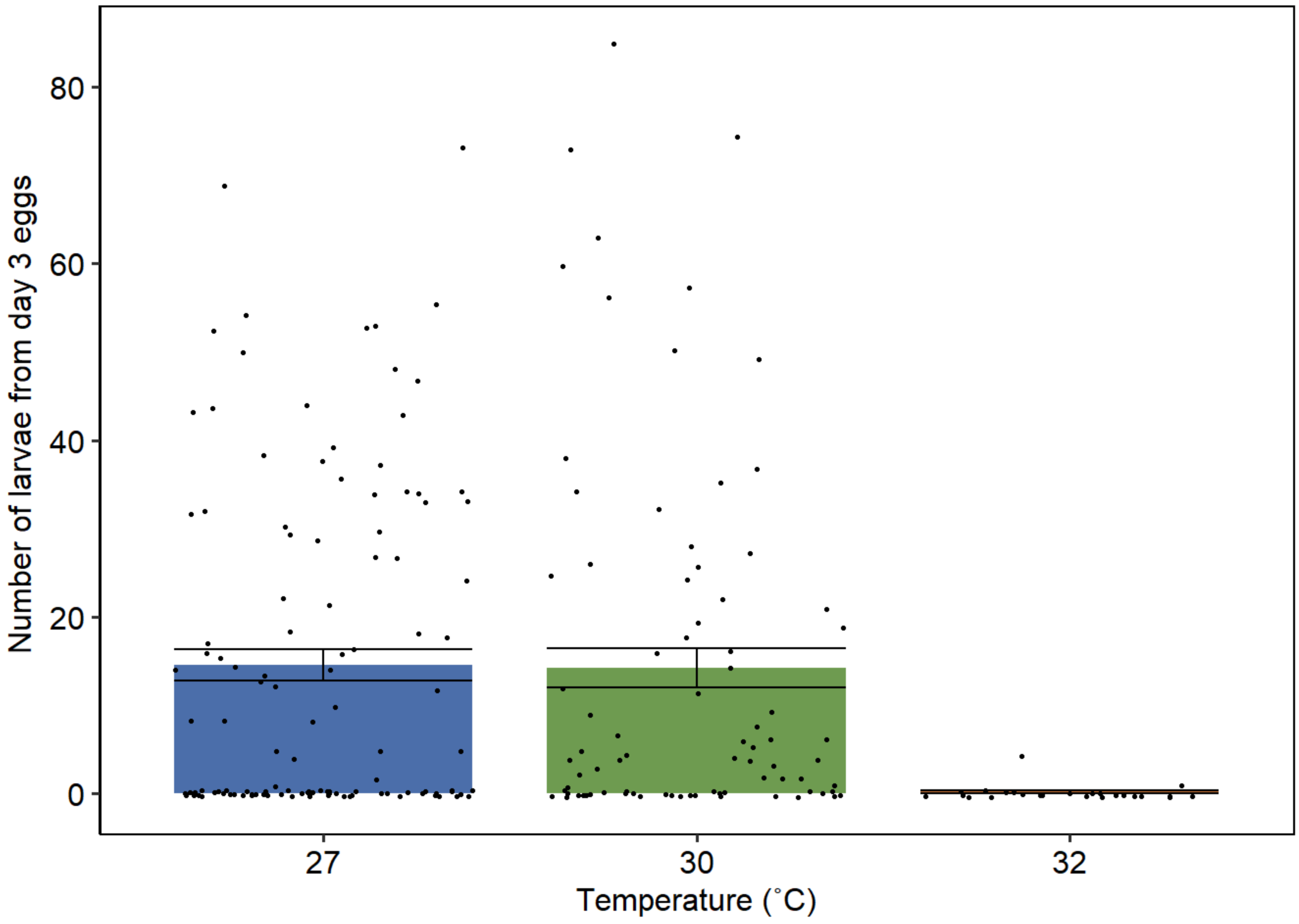

80
60
40
20
0
Number of larvae from day 3 eggs
27
30
32
Temperature (˚C)

```
ggsave("Oviposition_eggsandlarvae/BM1_ovipos_meanlarvae_day4_temponly.pdf", plot=BM1_ovipos_meanlarvae_day4
_temponly, width = 4, height = 4,
       units = "in",dpi=600)
ggsave("Oviposition_eggsandlarvae/BM1_ovipos_meanlarvae_day4_temponly.png", plot=BM1_ovipos_meanlarvae_day4
_temponly, width = 4, height = 4,
       units = "in",dpi=600)

#AGE ONLY
Summary_Fecundity_data_firstBM_ovipos_AGE$Age <- factor(Summary_Fecundity_data_firstBM_ovipos_AGE$Age,
                                                        labels = c("3","5","10","15"))
Fecundity_data_firstBM_ovipos$Age <- factor(Fecundity_data_firstBM_ovipos$Age,
                                            labels = c("3","5","10","15"))

BM1_ovipos_meanlarvae_day4_ageonly <- Summary_Fecundity_data_firstBM_ovipos_AGE %>%
  ggplot(aes(x=Age,y=mean_larvae_day4,group=Age))+
  geom_bar(aes(fill=Age),
           stat = "identity",
           position = position_dodge(1),
           width = 0.8) +
  scale_shape_identity(guide="legend")+
  #facet_grid(~Temperature)+
  geom_errorbar(aes(ymin=(mean_larvae_day4 - SE_larvae_day4),
                    ymax=(mean_larvae_day4 + SE_larvae_day4)),
                width=0.8,position=position_dodge(0.9),
                color="black")+
  ylab(expression("Number of larvae from day 3 eggs"))+
  xlab("Age of first BM (days old)") +
  theme_pubr()+
  theme(legend.position = "none")+
  geom_jitter(data=Fecundity_data_firstBM_ovipos, aes(x=Age,y=Larvae_day4),#color=ZOI_italic$Technical_Rep,
              position = "jitter", na.rm = TRUE,size=0.5)+
  scale_fill_manual(values= c("#DCD1E9","#BAA4D3","#9776BE","#7549A8"))+
  theme(panel.background = element_rect(fill = NA, color = "black"))+
  theme(panel.spacing = unit(0.5, "lines"))
BM1_ovipos_meanlarvae_day4_ageonly
```

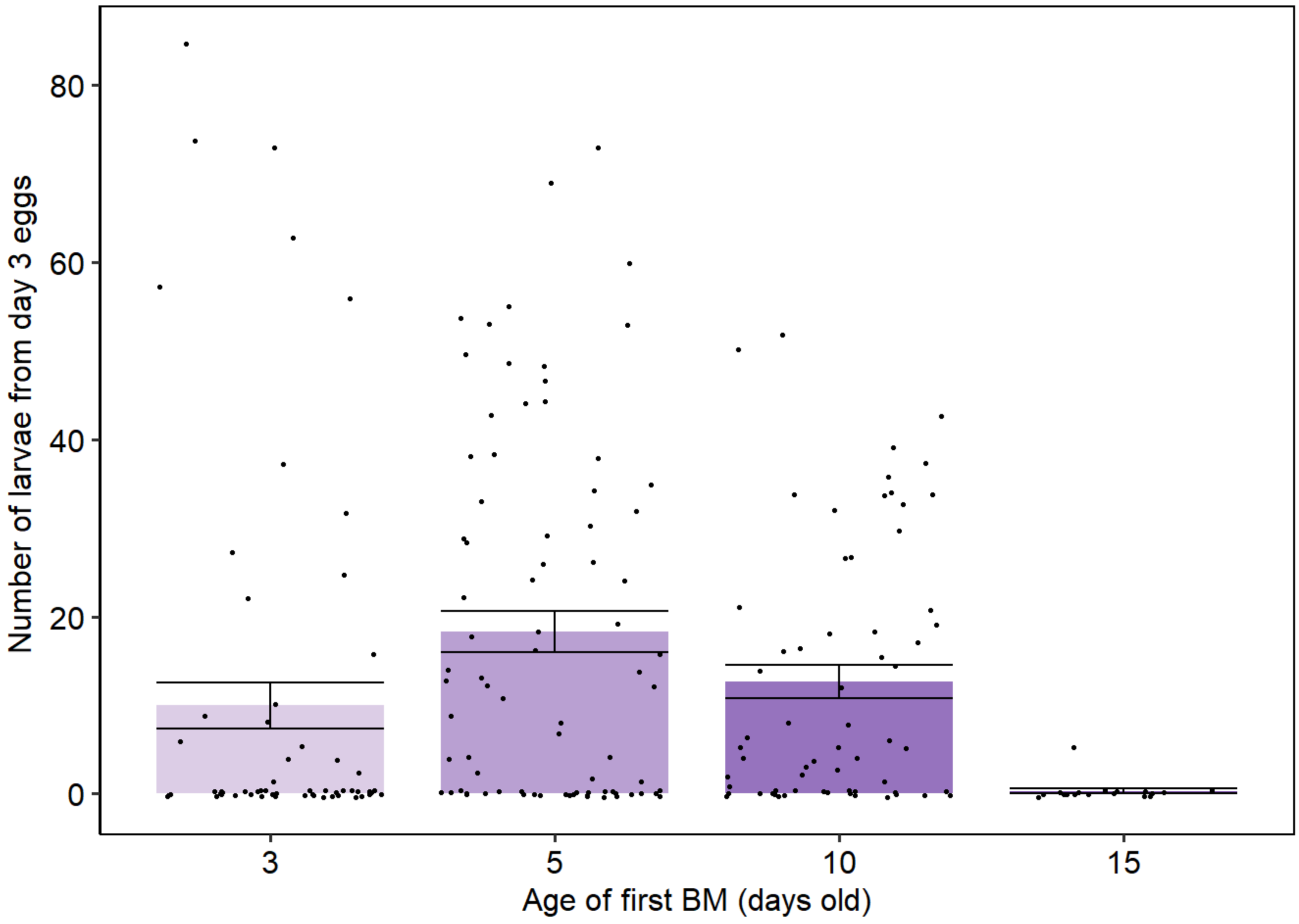


```
ggsave("Oviposition_eggsandlarvae/BM1_ovipos_meanlarvae_day4_ageonly.pdf", plot=BM1_ovipos_meanlarvae_day4_ageonly, width = 4, height = 4,
       units = "in",dpi=600)
ggsave("Oviposition_eggsandlarvae/BM1_ovipos_meanlarvae_day4_ageonly.png", plot=BM1_ovipos_meanlarvae_day4_ageonly, width = 4, height = 4,
       units = "in",dpi=600)
```

Plot percent eggs hatching into larvae

```
#Percent eggs hatched into larvae
Summary_Fecundity_data_firstBM_ovipos$Age <- factor(Summary_Fecundity_data_firstBM_ovipos$Age,
                                                    labels = c("3 days","5 days","10 days","15 days"))

Summary_Fecundity_data_firstBM_ovipos$Temperature <- factor(Summary_Fecundity_data_firstBM_ovipos$Temperatu
re,
                                                            labels = c("27","30","32"))

Fecundity_data_firstBM_ovipos$Age <- factor(Fecundity_data_firstBM_ovipos$Age,
                                            labels = c("3 days","5 days","10 days","15 days"))

Fecundity_data_firstBM_ovipos$Temperature <- factor(Fecundity_data_firstBM_ovipos$Temperature,
                                                    labels = c("27","30","32"))



BM1_ovipos_percenteggshatchedlarvae_tempwithinage <- Summary_Fecundity_data_firstBM_ovipos %>%
  ggplot(aes(x=Temperature,y=mean_Percent_eggshatchedtolarv,group=Temperature))+
  geom_bar(aes(fill=Temperature),
           stat = "identity",
           position = position_dodge(1),
           width = 0.8) +
  scale_shape_identity(guide="legend")+
  facet_grid(~Age)+
  geom_errorbar(aes(ymin=(mean_Percent_eggshatchedtolarv - SE_percenteggshatched),
                    ymax=(mean_Percent_eggshatchedtolarv + SE_percenteggshatched)),
                width=0.8,position=position_dodge(0.9),
                color="black")+
  ylab(expression("Mean % of eggs hatched into larvae"))+
  xlab("Temperature (˚C)") +
  theme_pubr()+
  theme(legend.position = "none")+
  scale_y_continuous(labels = function(x) paste0(x*100),limits=c(0,1)) +
  geom_jitter(data=Fecundity_data_firstBM_ovipos, aes(x=Temperature,y=Percent_eggshatchedtolarv),#color=ZOI
_italic$Technical_Rep,
              position = "jitter", na.rm = TRUE,size=0.5)+
  scale_fill_manual(values= c("#4D6FAE","#6F9F51", "#CC763B"))+
  theme(panel.background = element_rect(fill = NA, color = "black"))+
  theme(panel.spacing = unit(0.5, "lines"))
BM1_ovipos_percenteggshatchedlarvae_tempwithinage
```

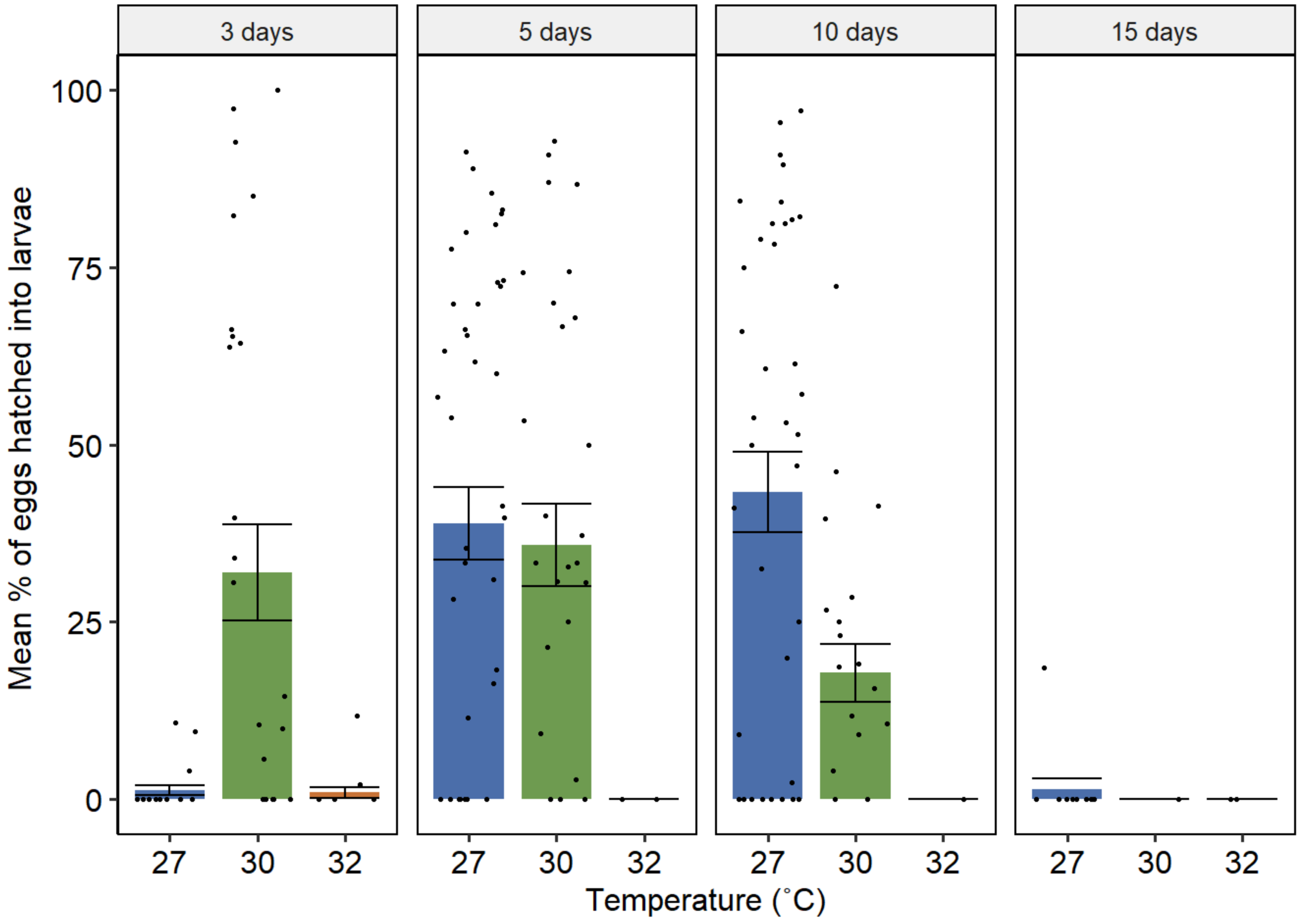
3 days
5 days
10 days
15 days
Mean % of eggs hatched into larvae
100
75
50
25
0
27
30
32
Temperature (˚C)

```
ggsave("Oviposition_eggsandlarvae/BM1_ovipos_percenteggshatchedlarvae_tempwithinage.pdf", plot=BM1_ovipos_p
ercenteggshatchedlarvae_tempwithinage, width = 6, height = 4,
       units = "in",dpi=600)
ggsave("Oviposition_eggsandlarvae/BM1_ovipos_percenteggshatchedlarvae_tempwithinage.png", plot=BM1_ovipos_p
ercenteggshatchedlarvae_tempwithinage, width = 6, height = 4,
       units = "in",dpi=600)



Summary_Fecundity_data_firstBM_ovipos$Age <- factor(Summary_Fecundity_data_firstBM_ovipos$Age,
                                                    labels = c("3","5","10","15"))
Summary_Fecundity_data_firstBM_ovipos$Temperature <- factor(Summary_Fecundity_data_firstBM_ovipos$Temperatu
re,
                                                            labels = c("27˚C","30˚C","32˚C"))

Fecundity_data_firstBM_ovipos$Age <- factor(Fecundity_data_firstBM_ovipos$Age,
                                            labels = c("3","5","10","15"))
Fecundity_data_firstBM_ovipos$Temperature <- factor(Fecundity_data_firstBM_ovipos$Temperature,
                                                    labels = c("27˚C","30˚C","32˚C"))

BM1_ovipos_percenteggshatchedlarvae_agewithintemp <- Summary_Fecundity_data_firstBM_ovipos %>%
  ggplot(aes(x=Age,y=mean_Percent_eggshatchedtolarv,group=Age))+
  geom_bar(aes(fill=Age),
           stat = "identity",
           position = position_dodge(1),
           width = 0.8) +
  scale_shape_identity(guide="legend")+
  facet_grid(~Temperature)+
  geom_errorbar(aes(ymin=(mean_Percent_eggshatchedtolarv - SE_percenteggshatched),
                    ymax=(mean_Percent_eggshatchedtolarv + SE_percenteggshatched)),
                width=0.8,position=position_dodge(0.9),
                color="black")+
  ylab(expression("Mean % of eggs hatched into larvae"))+
  scale_y_continuous(labels = function(x) paste0(x*100),limits=c(0,1)) +
  xlab("Age of first BM (days old)") +
  theme_pubr()+
  theme(legend.position = "none")+
  geom_jitter(data=Fecundity_data_firstBM_ovipos, aes(x=Age,y=Percent_eggshatchedtolarv),#color=ZOI_italic
$Technical_Rep,
              position = "jitter", na.rm = TRUE,size=0.5)+
  scale_fill_manual(values= c("#DCD1E9","#BAA4D3","#9776BE","#7549A8"))+
  theme(panel.background = element_rect(fill = NA, color = "black"))+
  theme(panel.spacing = unit(0.5, "lines"))
BM1_ovipos_percenteggshatchedlarvae_agewithintemp
```

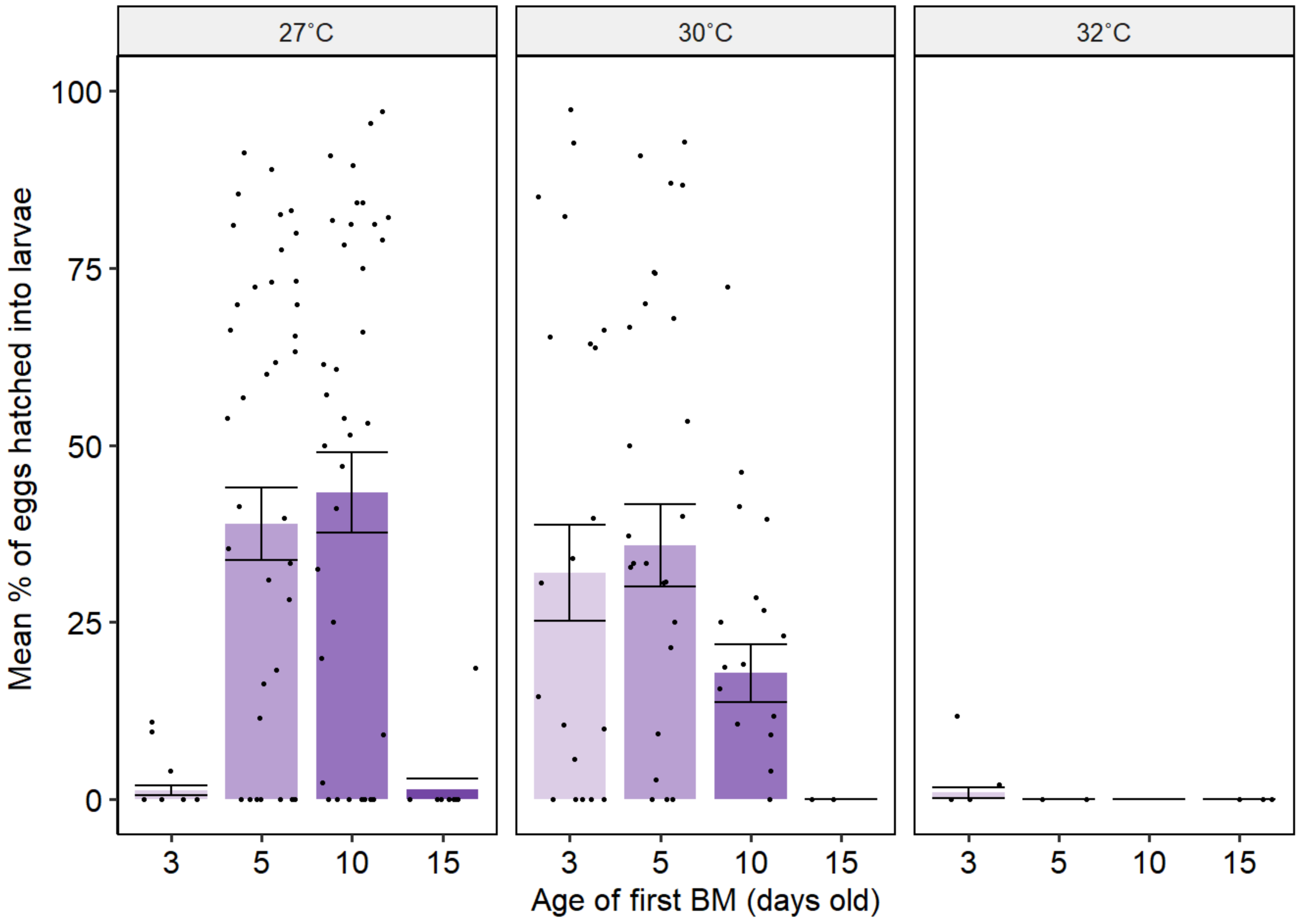


```
ggsave("Oviposition_eggsandlarvae/BM1_ovipos_percenteggshatchedlarvae_agewithintemp.pdf", plot=BM1_ovipos_percenteggshatchedlarvae_agewithintemp, width = 6, height = 4,
       units = "in",dpi=600)
ggsave("Oviposition_eggsandlarvae/BM1_ovipos_percenteggshatchedlarvae_agewithintemp.png", plot=BM1_ovipos_percenteggshatchedlarvae_agewithintemp, width = 6, height = 4,
       units = "in",dpi=600)

#raw data interaction plot:
BM1_ovipos_percenteggshatched_rawdata_interaction <- Summary_Fecundity_data_firstBM_ovipos%>%
  ggplot()+
  aes(x=Age,y=mean_Percent_eggshatchedtolarv,group=Temperature)+
  geom_point(aes(shape=Temperature),size=3)+
  geom_line(aes(linetype = Temperature),linewidth=0.6)+
  theme_pubr()+
  scale_shape(labels=c(27,30,32))+
  scale_linetype(labels=c(27,30,32))+
  guides(shape = guide_legend(title = "Temperature (˚C)"),
         linetype = guide_legend(title = "Temperature (˚C)"))+
  xlab("Adult Age (days)") +
  theme(legend.position = "right")+
  scale_x_discrete(labels=c(3,5,10,15))+
  ylab(expression("% of eggs hatched into larvae"))+
  theme(panel.background = element_rect(fill = NA, color = "black"))+
  theme(panel.spacing = unit(0.6, "lines"))
BM1_ovipos_percenteggshatched_rawdata_interaction
```

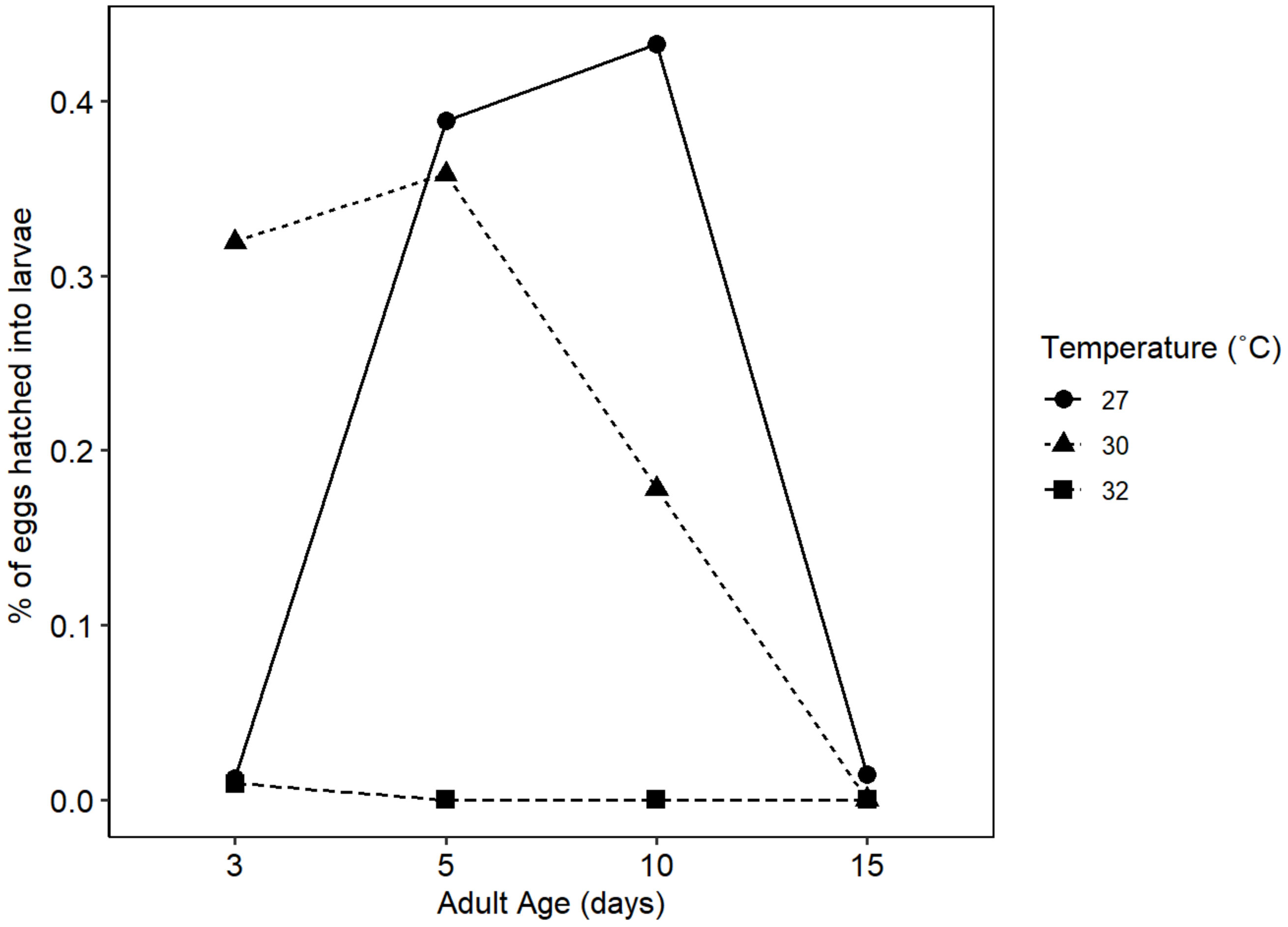

% of eggs hatched into larvae
0.0
0.1
0.2
0.3
0.4
3
5
10
15
Adult Age (days)
Temperature (˚C)
27
30
32

```
ggsave("Oviposition_eggsandlarvae/BM1_ovipos_percenteggshatched_rawdata_interaction.png",plot=BM1_ovipos_pe
rcenteggshatched_rawdata_interaction,width = 5.5, height = 4, units = "in", dpi = 600)
ggsave("Oviposition_eggsandlarvae/BM1_ovipos_percenteggshatched_rawdata_interaction.pdf",plot=BM1_ovipos_pe
rcenteggshatched_rawdata_interaction,width = 5.5, height = 4, units = "in", dpi = 600)




#TEMP ONLY
Summary_Fecundity_data_firstBM_ovipos_TEMP$Temperature <- factor(Summary_Fecundity_data_firstBM_ovipos_TEMP
$Temperature,
                                                                 labels = c("27","30","32"))
Fecundity_data_firstBM_ovipos$Temperature <- factor(Fecundity_data_firstBM_ovipos$Temperature,
                                                    labels = c("27","30","32"))

BM1_ovipos_percenteggshatchedlarvae_TEMPONLY <- Summary_Fecundity_data_firstBM_ovipos_TEMP %>%
  ggplot(aes(x=Temperature,y=mean_Percent_eggshatchedtolarv,group=Temperature))+
  geom_bar(aes(fill=Temperature),
           stat = "identity",
           position = position_dodge(1),
           width = 0.8) +
  scale_shape_identity(guide="legend")+
  #facet_grid(~Age)+
  geom_errorbar(aes(ymin=(mean_Percent_eggshatchedtolarv - SE_percenteggshatched),
                    ymax=(mean_Percent_eggshatchedtolarv + SE_percenteggshatched)),
                width=0.8,position=position_dodge(0.9),
                color="black")+
  ylab(expression("Mean % of eggs hatched into larvae"))+
  scale_y_continuous(labels = function(x) paste0(x*100),limits=c(0,1)) +
  xlab("Temperature (˚C)") +
  theme_pubr()+
  theme(legend.position = "none")+
  geom_jitter(data=Fecundity_data_firstBM_ovipos, aes(x=Temperature,y=Percent_eggshatchedtolarv),#color=ZOI
_italic$Technical_Rep,
              position = "jitter", na.rm = TRUE,size=0.5)+
  scale_fill_manual(values= c("#4D6FAE","#6F9F51", "#CC763B"))+
  theme(panel.background = element_rect(fill = NA, color = "black"))+
  theme(panel.spacing = unit(0.5, "lines"))
BM1_ovipos_percenteggshatchedlarvae_TEMPONLY
```

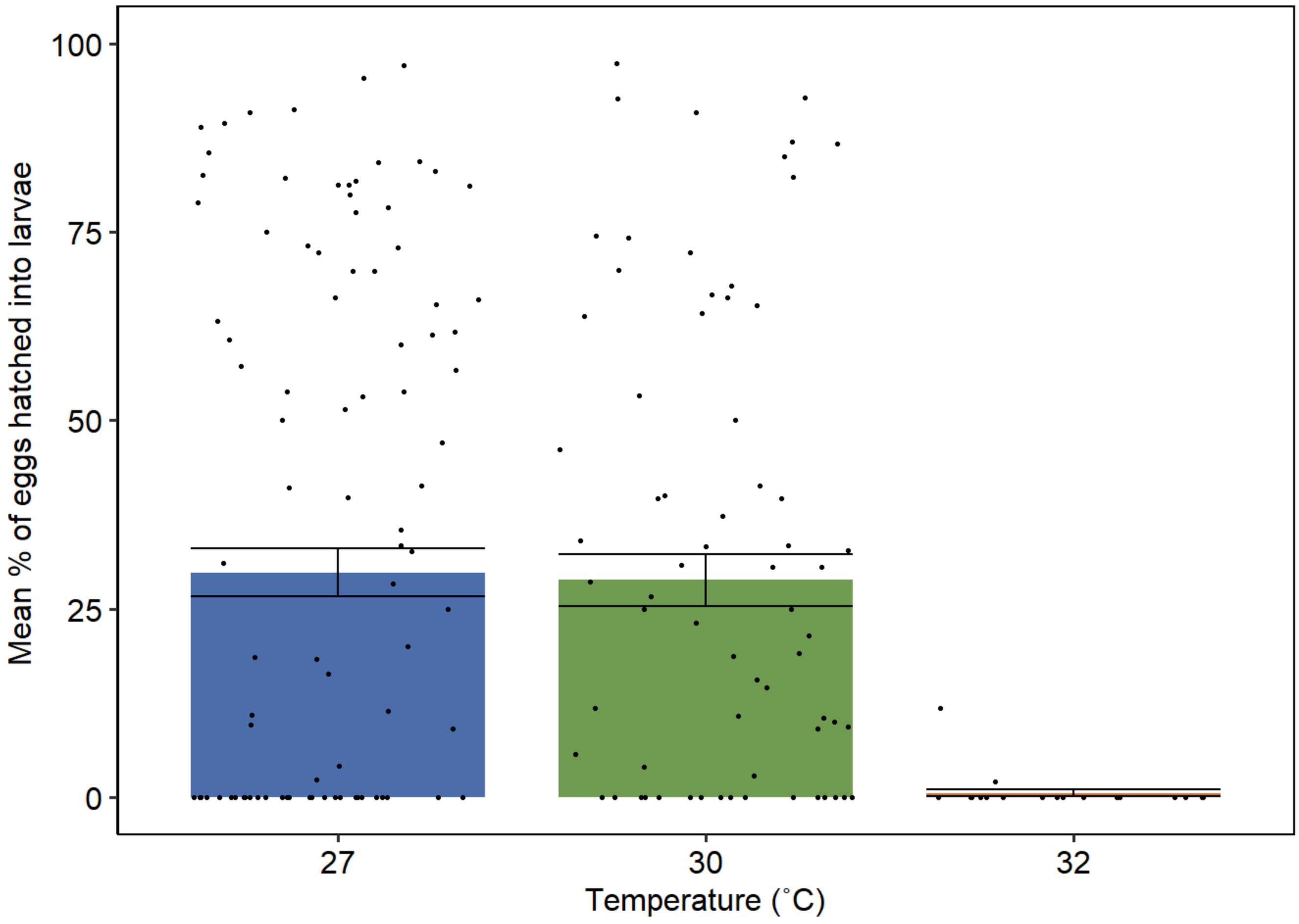

Mean % of eggs hatched into larvae
100
75
50
25
0
27
30
32
Temperature (˚C)

```
ggsave("Oviposition_eggsandlarvae/BM1_ovipos_percenteggshatchedlarvae_TEMPONLY.pdf", plot=BM1_ovipos_percen
teggshatchedlarvae_TEMPONLY, width = 4, height = 4,
       units = "in",dpi=600)
ggsave("Oviposition_eggsandlarvae/BM1_ovipos_percenteggshatchedlarvae_TEMPONLY.png", plot=BM1_ovipos_percen
teggshatchedlarvae_TEMPONLY, width =4, height = 4,
       units = "in",dpi=600)

#AGE ONLY
Summary_Fecundity_data_firstBM_ovipos_AGE$Age <- factor(Summary_Fecundity_data_firstBM_ovipos_AGE$Age,
                                                        labels = c("3","5","10","15"))
Fecundity_data_firstBM_ovipos$Age <- factor(Fecundity_data_firstBM_ovipos$Age,
                                            labels = c("3","5","10","15"))

BM1_ovipos_percenteggshatchedlarvae_AGEONLY <- Summary_Fecundity_data_firstBM_ovipos_AGE %>%
  ggplot(aes(x=Age,y=mean_Percent_eggshatchedtolarv,group=Age))+
  geom_bar(aes(fill=Age),
           stat = "identity",
           position = position_dodge(1),
           width = 0.8) +
  scale_shape_identity(guide="legend")+
  #facet_grid(~Temperature)+
  geom_errorbar(aes(ymin=(mean_Percent_eggshatchedtolarv - SE_percenteggshatched),
                    ymax=(mean_Percent_eggshatchedtolarv + SE_percenteggshatched)),
                width=0.8,position=position_dodge(0.9),
                color="black")+
  ylab(expression("Mean % of eggs hatched into larvae"))+
  scale_y_continuous(labels = function(x) paste0(x*100),limits=c(0,1)) +
  xlab("Age of first BM (days old)") +
  theme_pubr()+
  theme(legend.position = "none")+
  geom_jitter(data=Fecundity_data_firstBM_ovipos, aes(x=Age,y=Percent_eggshatchedtolarv),#color=ZOI_italic
$Technical_Rep,
              position = "jitter", na.rm = TRUE,size=0.5)+
  scale_fill_manual(values= c("#DCD1E9","#BAA4D3","#9776BE","#7549A8"))+
  theme(panel.background = element_rect(fill = NA, color = "black"))+
  theme(panel.spacing = unit(0.5, "lines"))
BM1_ovipos_percenteggshatchedlarvae_AGEONLY
```

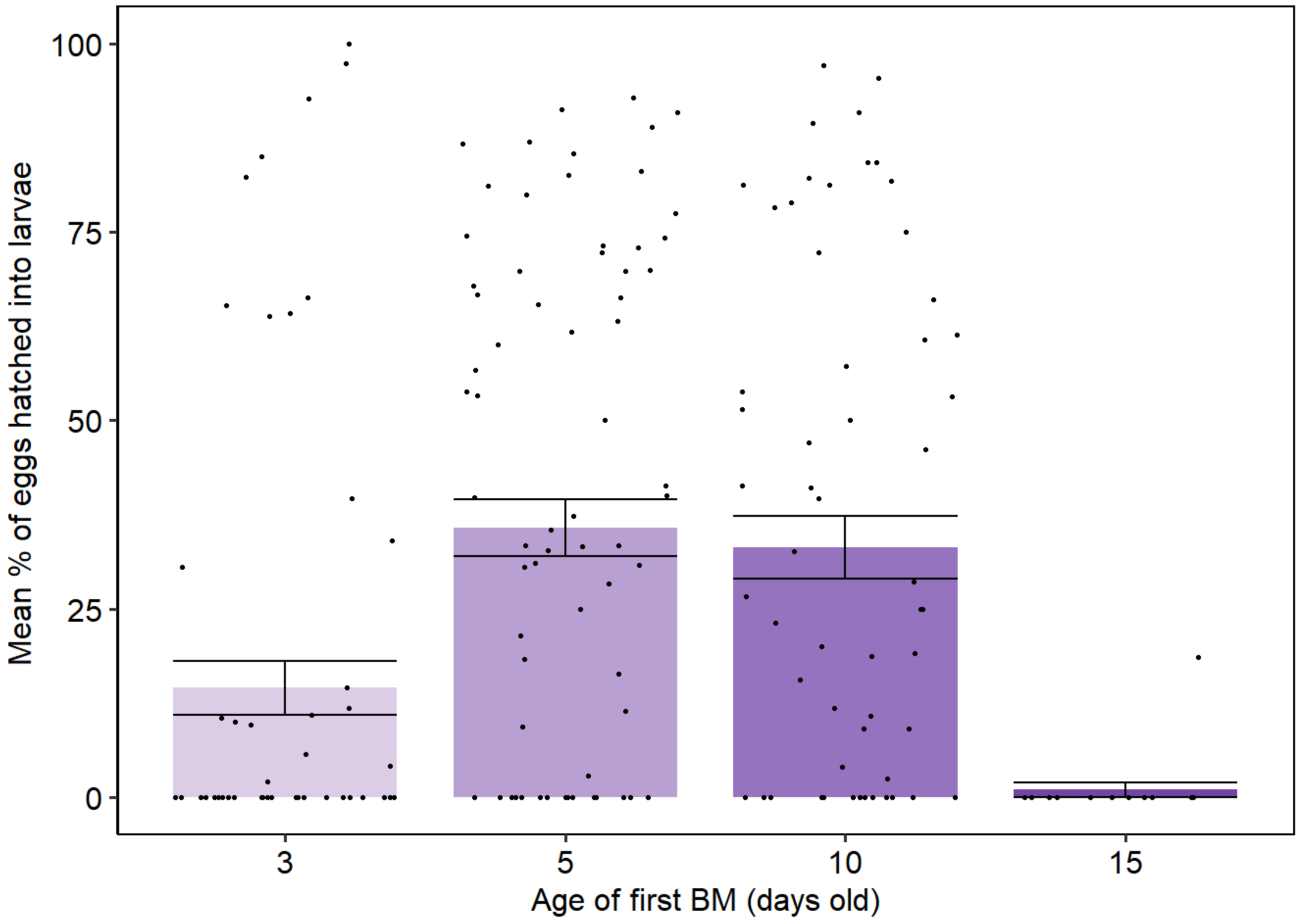


```
ggsave("Oviposition_eggsandlarvae/BM1_ovipos_percenteggshatchedlarvae_AGEONLY.pdf", plot=BM1_ovipos_percent
eggshatchedlarvae_AGEONLY, width = 4, height = 4,
       units = "in",dpi=600)
ggsave("Oviposition_eggsandlarvae/BM1_ovipos_percenteggshatchedlarvae_AGEONLY.png", plot=BM1_ovipos_percent
eggshatchedlarvae_AGEONLY, width =4, height = 4,
       units = "in",dpi=600)
```

Analysis for total number of larvae on day 4 (if laid eggs)

```
#ANALYSIS FOR NUMBER OF LARVAE ON DAY 4 IF LAID EGGS DAY 3

#data: Fecundity_data_firstBM_ovipos
#WITH FILTER FOR REMOVING EGGS < 0 ON DAY 3 (see above)

hist(Fecundity_data_firstBM_ovipos$Larvae_day4)
```

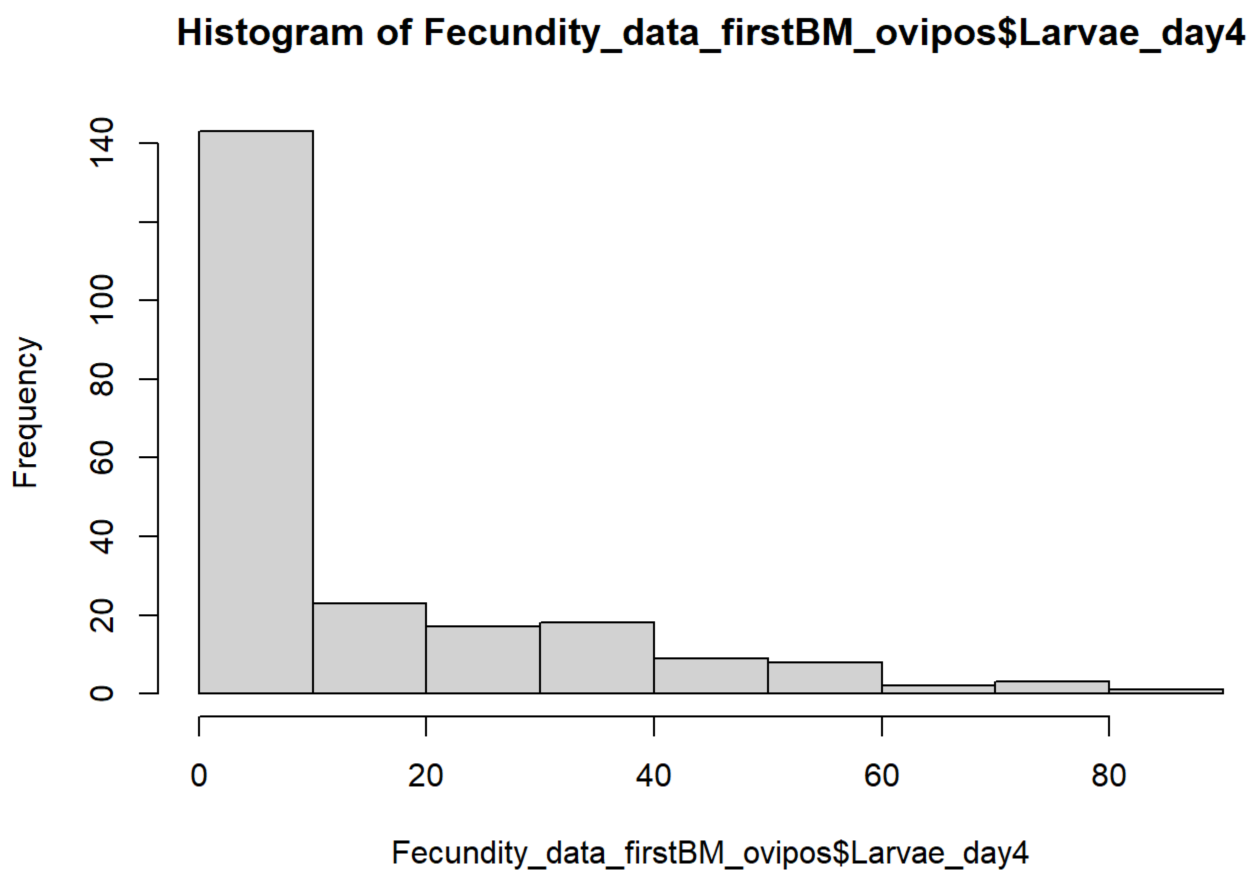


```
shapiro.test(Fecundity_data_firstBM_ovipos$Larvae_day4)
```

```
##
##  Shapiro-Wilk normality test
##
## data:  Fecundity_data_firstBM_ovipos$Larvae_day4
## W = 0.73157, p-value < 2.2e-16
```

```
#non-normal. count data.

library(glmmTMB)
model.larvaeday4.0 = glmmTMB(Larvae_day4 ~ Temperature*Age+(1|Trial_number), data= Fecundity_data_firstBM_o
vipos,family=poisson(link="log")) #equals model 1
#overdispersion?
pr <- residuals(model.larvaeday4.0,"pearson")
phi <- sum(pr^2)/df.residual(model.larvaeday4.0)
dis <-round(c(phi,sqrt(phi)),4) #variance is higher
dis #overdispersed
```

```
## [1] 16.6956  4.0860
```

```
library(DHARMa)
#try neg bin
model.larvaeday4.1 = glmmTMB(Larvae_day4 ~ Temperature*Age+(1|Trial_number), data= Fecundity_data_firstBM_o
vipos,family=nbinom2(link="log")) #equals model 1
plot(simulateResiduals(model.larvaeday4.1))
```

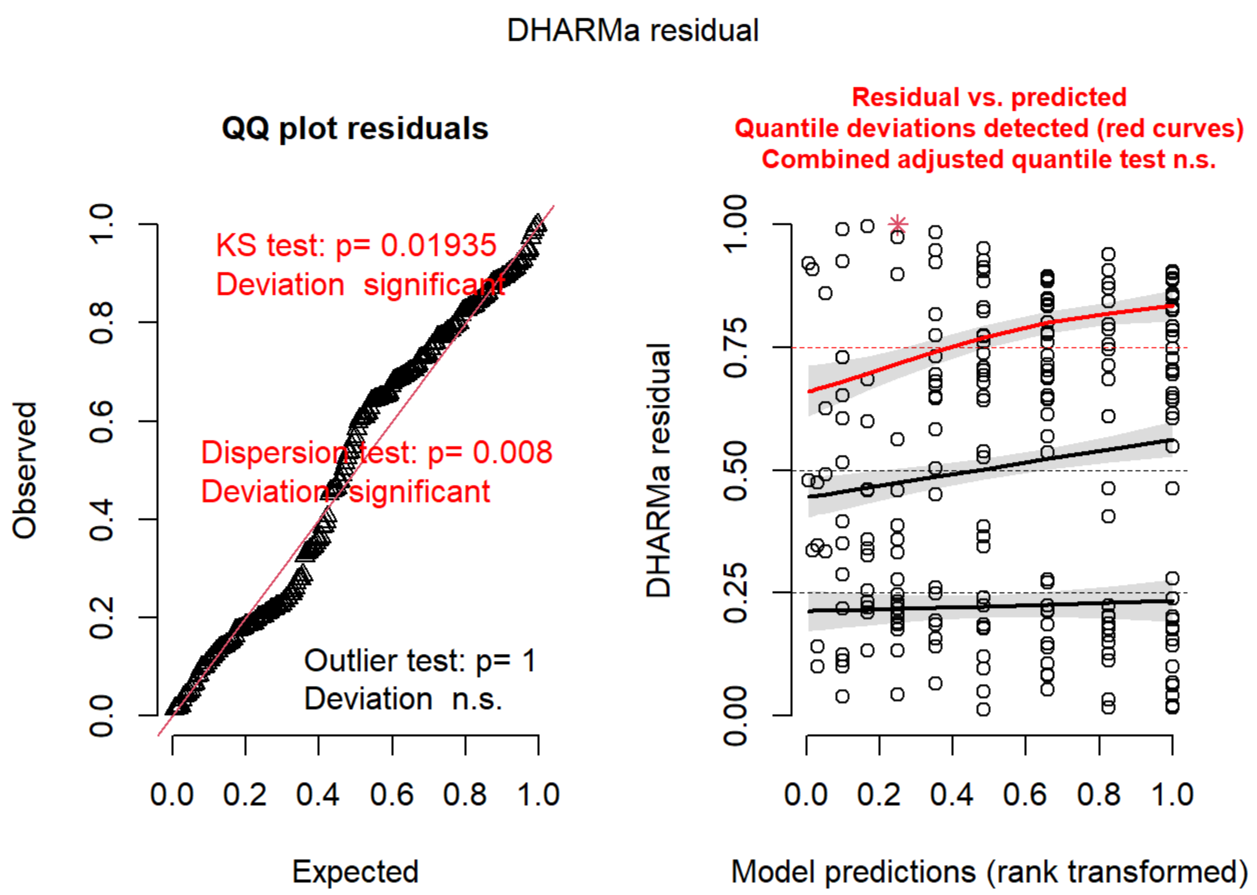


```
summary(model.larvaeday4.1)
```

```
##  Family: nbinom2  ( log )
## Formula:          Larvae_day4 ~ Temperature * Age + (1 | Trial_number)
## Data: Fecundity_data_firstBM_ovipos
##
##      AIC      BIC   logLik deviance df.resid
##   1276.6   1324.4   -624.3   1248.6      210
##
## Random effects:
##
## Conditional model:
##  Groups       Name        Variance  Std.Dev.
##  Trial_number (Intercept) 4.443e-11 6.666e-06
## Number of obs: 224, groups:  Trial_number, 5
##
## Dispersion parameter for nbinom2 family (): 0.329
##
## Conditional model:
##                        Estimate Std. Error z value Pr(>|z|)
## (Intercept)          -1.625e-01  4.591e-01  -0.354    0.723
## Temperature30         3.254e+00  5.701e-01   5.707 1.15e-08 ***
## Temperature32        -9.361e-01  7.832e-01  -1.195    0.232
## Age5                  3.276e+00  5.300e-01   6.181 6.35e-10 ***
## Age10                 3.012e+00  5.369e-01   5.610 2.02e-08 ***
## Age15                -7.930e-01  8.029e-01  -0.988    0.323
## Temperature30:Age5  -3.681e+00  7.038e-01  -5.230 1.70e-07 ***
## Temperature32:Age5  -2.672e+01  1.070e+05   0.000    1.000
## Temperature30:Age10 -4.415e+00  7.410e-01  -5.958 2.55e-09 ***
## Temperature32:Age10 -2.760e+01  2.680e+05   0.000    1.000
## Temperature30:Age15 -2.862e+01  3.670e+05   0.000    1.000
## Temperature32:Age15 -1.576e+01  3.409e+03  -0.005    0.996
## ---
## Signif. codes:  0 '***' 0.001 '**' 0.01 '*' 0.05 '.' 0.1 ' ' 1
```

```
plot(fitted(model.larvaeday4.1),
     residuals(model.larvaeday4.1))
```

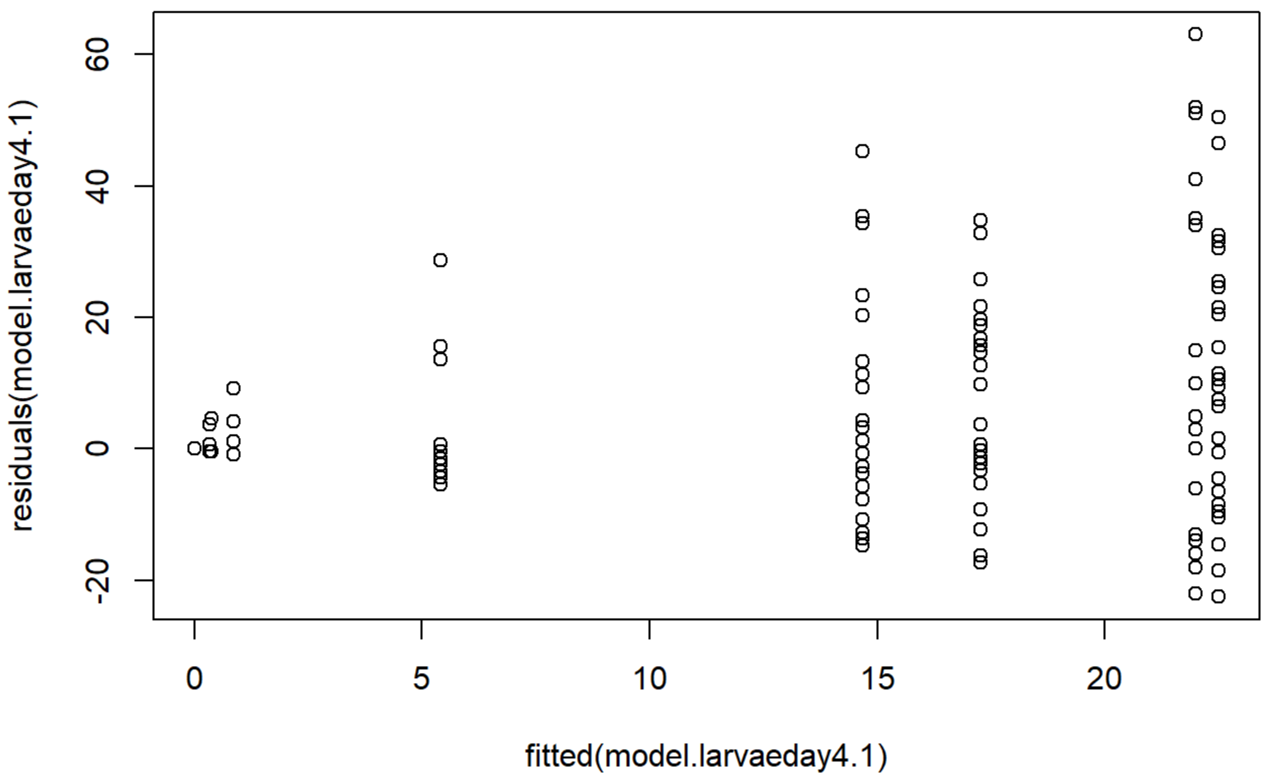


```
AIC(model.larvaeday4.0,model.larvaeday4.1) #big improvement.
```

```
##                     df      AIC
## model.larvaeday4.0 13 4188.879
## model.larvaeday4.1 14 1276.640
```

```
#still not quite right, try zero inflated neg bin.

library(pscl)
```

```
## Classes and Methods for R originally developed in the
## Political Science Computational Laboratory
## Department of Political Science
## Stanford University (2002-2015),
## by and under the direction of Simon Jackman.
## hurdle and zeroinfl functions by Achim Zeileis.
```

```
model.larvaeday4.2 <- zeroinfl(Larvae_day4 ~ Temperature*Age | Temperature, data = Fecundity_data_firstBM_o
vipos, dist = "negbin")
model.larvaeday4.3 <- zeroinfl(Larvae_day4 ~ Temperature*Age | Age, data = Fecundity_data_firstBM_ovipos, d
ist = "negbin")
model.larvaeday4.4 <- zeroinfl(Larvae_day4 ~ Temperature*Age | Temperature+Age, data = Fecundity_data_first
BM_ovipos, dist = "negbin")

AIC(model.larvaeday4.0,model.larvaeday4.1,model.larvaeday4.2,model.larvaeday4.3,model.larvaeday4.4)
```

```
##                     df      AIC
## model.larvaeday4.0 13 4188.879
## model.larvaeday4.1 14 1276.640
## model.larvaeday4.2 16 1220.487
## model.larvaeday4.3 17 1209.283
## model.larvaeday4.4 19 1208.347
```

```
summary(model.larvaeday4.2)
```

```
##
## Call:
## zeroinfl(formula = Larvae_day4 ~ Temperature * Age | Temperature, data = Fecundity_data_firstBM_ovipos,
##     dist = "negbin")
##
## Pearson residuals:
##     Min      1Q  Median      3Q     Max
## -0.8568 -0.7252 -0.3117  0.3168  4.9483
##
## Count model coefficients (negbin with log link):
##                       Estimate Std. Error z value Pr(>|z|)
## (Intercept)             0.8145     0.5300   1.537 0.124369
## Temperature30           2.7937     0.5635   4.958 7.13e-07 ***
## Temperature32          -0.2472     0.9534  -0.259 0.795423
## Age5                    2.7120     0.5484   4.945 7.61e-07 ***
## Age10                   2.3859     0.5493   4.344 1.40e-05 ***
## Age15                  -1.0579     0.7933  -1.334 0.182363
## Temperature30:Age5     -3.3004     0.6053  -5.453 4.96e-08 ***
## Temperature32:Age5    -16.4800   845.7098  -0.019 0.984453
## Temperature30:Age10    -3.9681     0.6226  -6.373 1.85e-10 ***
## Temperature32:Age10   -16.2158  1233.5791  -0.013 0.989512
## Temperature30:Age15   -16.6006   967.8970  -0.017 0.986316
## Temperature32:Age15   -12.4110   728.2239  -0.017 0.986402
## Log(theta)              0.5665     0.1653   3.427 0.000609 ***
##
## Zero-inflation model coefficients (binomial with logit link):
##                Estimate Std. Error z value Pr(>|z|)
## (Intercept)     -0.4405     0.2404  -1.832   0.0670 .
## Temperature30   -0.2904     0.3421  -0.849   0.3959
## Temperature32    1.8968     0.9197   2.062   0.0392 *
## ---
## Signif. codes:  0 '***' 0.001 '**' 0.01 '*' 0.05 '.' 0.1 ' ' 1
##
## Theta = 1.7622
## Number of iterations in BFGS optimization: 18
## Log-likelihood: -594.2 on 16 Df
```

```
#model 2 makes most sense with data. has low AIC

Anova(model.larvaeday4.2,type=2,test="Chisq")
```

```
## Analysis of Deviance Table (Type II tests)
##
## Response: Larvae_day4
##                  Df  Chisq Pr(>Chisq)
## Temperature      2 19.777  5.075e-05 ***
## Age              3 39.415  1.418e-08 ***
## Temperature:Age  6 40.637  3.413e-07 ***
## ---
## Signif. codes:  0 '***' 0.001 '**' 0.01 '*' 0.05 '.' 0.1 ' ' 1
```

```
BM1_larvaeday4_anova <- Anova(model.larvaeday4.2,type=2)
BM1_larvaeday4_anova <- as.data.frame(BM1_larvaeday4_anova)
write_xlsx(BM1_larvaeday4_anova, "Oviposition_eggsandlarvae/BM1_larvaeday4_anova.xlsx")

sink("Oviposition_eggsandlarvae/BM1_larvaeday4_anova.txt")
Anova(model.larvaeday4.2,type=2)
sink()

sink("Oviposition_eggsandlarvae/BM1_larvaeday4_modelsummary.txt")
summary(model.larvaeday4.2)
sink()

BM1_larvaeday4_emmeans <- emmeans(model.larvaeday4.2,~Temperature*Age,
                                  type="response")
BM1_larvaeday4_emmeans_PAIRS <- pairs(BM1_larvaeday4_emmeans, adjust="sidak")
BM1_larvaeday4_emmeans_PAIRS
```

```
##  contrast                                    estimate       SE  df z.ratio
##  Temperature27 Age3 - Temperature30 Age3      -23.533 5.238784 Inf  -4.492
##  Temperature27 Age3 - Temperature32 Age3        1.040 0.743463 Inf   1.399
##  Temperature27 Age3 - Temperature27 Age5      -19.313 3.679398 Inf  -5.249
##  Temperature27 Age3 - Temperature30 Age5      -12.455 2.649189 Inf  -4.701
##  Temperature27 Age3 - Temperature32 Age5        1.374 0.690287 Inf   1.990
##  Temperature27 Age3 - Temperature27 Age10     -13.556 2.761415 Inf  -4.909
##  Temperature27 Age3 - Temperature30 Age10      -3.745 1.355115 Inf  -2.764
##  Temperature27 Age3 - Temperature32 Age10       1.374 0.690287 Inf   1.990
##  Temperature27 Age3 - Temperature27 Age15       0.897 0.746047 Inf   1.202
##  Temperature27 Age3 - Temperature30 Age15       1.374 0.690287 Inf   1.990
##  Temperature27 Age3 - Temperature32 Age15       1.374 0.690287 Inf   1.990
##  Temperature30 Age3 - Temperature32 Age3       24.573 5.199242 Inf   4.726
##  Temperature30 Age3 - Temperature27 Age5        4.220 6.280710 Inf   0.672
##  Temperature30 Age3 - Temperature30 Age5       11.078 5.413685 Inf   2.046
##  Temperature30 Age3 - Temperature32 Age5       24.907 5.191905 Inf   4.797
##  Temperature30 Age3 - Temperature27 Age10       9.977 5.804963 Inf   1.719
##  Temperature30 Age3 - Temperature30 Age10      19.788 5.190108 Inf   3.813
##  Temperature30 Age3 - Temperature32 Age10      24.907 5.191905 Inf   4.797
##  Temperature30 Age3 - Temperature27 Age15      24.430 5.200549 Inf   4.698
##  Temperature30 Age3 - Temperature30 Age15      24.907 5.191905 Inf   4.797
##  Temperature30 Age3 - Temperature32 Age15      24.907 5.191905 Inf   4.797
##  Temperature32 Age3 - Temperature27 Age5      -20.353 3.549448 Inf  -5.734
##  Temperature32 Age3 - Temperature30 Age5      -13.495 2.571560 Inf  -5.248
##  Temperature32 Age3 - Temperature32 Age5        0.333 0.276119 Inf   1.207
##  Temperature32 Age3 - Temperature27 Age10     -14.597 2.615194 Inf  -5.581
##  Temperature32 Age3 - Temperature30 Age10      -4.785 1.200561 Inf  -3.986
##  Temperature32 Age3 - Temperature32 Age10       0.333 0.276119 Inf   1.207
##  Temperature32 Age3 - Temperature27 Age15      -0.144 0.406126 Inf  -0.354
##  Temperature32 Age3 - Temperature30 Age15       0.333 0.276119 Inf   1.207
##  Temperature32 Age3 - Temperature32 Age15       0.333 0.276119 Inf   1.207
##  Temperature27 Age5 - Temperature30 Age5        6.858 4.364238 Inf   1.571
##  Temperature27 Age5 - Temperature32 Age5       20.686 3.538692 Inf   5.846
##  Temperature27 Age5 - Temperature27 Age10       5.757 3.739416 Inf   1.539
##  Temperature27 Age5 - Temperature30 Age10      15.568 3.728335 Inf   4.176
##  Temperature27 Age5 - Temperature32 Age10      20.686 3.538692 Inf   5.846
##  Temperature27 Age5 - Temperature27 Age15      20.210 3.565860 Inf   5.668
##  Temperature27 Age5 - Temperature30 Age15      20.686 3.538692 Inf   5.846
##  Temperature27 Age5 - Temperature32 Age15      20.686 3.538692 Inf   5.846
##  Temperature30 Age5 - Temperature32 Age5       13.829 2.556693 Inf   5.409
##  Temperature30 Age5 - Temperature27 Age10      -1.101 3.645697 Inf  -0.302
##  Temperature30 Age5 - Temperature30 Age10       8.710 2.672239 Inf   3.259
##  Temperature30 Age5 - Temperature32 Age10      13.829 2.556693 Inf   5.409
##  Temperature30 Age5 - Temperature27 Age15      13.352 2.574069 Inf   5.187
##  Temperature30 Age5 - Temperature30 Age15      13.829 2.556693 Inf   5.409
##  Temperature30 Age5 - Temperature32 Age15      13.829 2.556693 Inf   5.409
##  Temperature32 Age5 - Temperature27 Age10     -14.930 2.600576 Inf  -5.741
##  Temperature32 Age5 - Temperature30 Age10      -5.119 1.168377 Inf  -4.381
##  Temperature32 Age5 - Temperature32 Age10       0.000 0.000502 Inf   0.000
##  Temperature32 Age5 - Temperature27 Age15      -0.477 0.297821 Inf  -1.601
##  Temperature32 Age5 - Temperature30 Age15       0.000 0.000595 Inf   0.000
##  Temperature32 Age5 - Temperature32 Age15       0.000 0.000453 Inf   0.000
##  Temperature27 Age10 - Temperature30 Age10      9.811 2.852557 Inf   3.439
##  Temperature27 Age10 - Temperature32 Age10     14.930 2.600576 Inf   5.741
##  Temperature27 Age10 - Temperature27 Age15     14.453 2.631830 Inf   5.492
##  Temperature27 Age10 - Temperature30 Age15     14.930 2.600577 Inf   5.741
```

```
##  Temperature27 Age10 - Temperature32 Age15   14.930 2.600576 Inf   5.741
##  Temperature30 Age10 - Temperature32 Age10    5.119 1.168377 Inf   4.381
##  Temperature30 Age10 - Temperature27 Age15    4.642 1.205539 Inf   3.850
##  Temperature30 Age10 - Temperature30 Age15    5.119 1.168377 Inf   4.381
##  Temperature30 Age10 - Temperature32 Age15    5.119 1.168377 Inf   4.381
##  Temperature32 Age10 - Temperature27 Age15   -0.477 0.297821 Inf  -1.601
##  Temperature32 Age10 - Temperature30 Age15    0.000 0.000657 Inf   0.000
##  Temperature32 Age10 - Temperature32 Age15    0.000 0.000531 Inf   0.000
##  Temperature27 Age15 - Temperature30 Age15    0.477 0.297821 Inf   1.601
##  Temperature27 Age15 - Temperature32 Age15    0.477 0.297821 Inf   1.601
##  Temperature30 Age15 - Temperature32 Age15    0.000 0.000620 Inf   0.000
##  p.value
##   0.0005
##   1.0000
##   <.0001
##   0.0002
##   0.9571
##   0.0001
##   0.3150
##   0.9571
##   1.0000
##   0.9571
##   0.9571
##   0.0002
##   1.0000
##   0.9357
##   0.0001
##   0.9973
##   0.0090
##   0.0001
##   0.0002
##   0.0001
##   0.0001
##   <.0001
##   <.0001
##   1.0000
##   <.0001
##   0.0044
##   1.0000
##   1.0000
##   1.0000
##   1.0000
##   0.9997
##   <.0001
##   0.9998
##   0.0020
##   <.0001
##   <.0001
##   <.0001
##   <.0001
##   <.0001
##   1.0000
##   0.0711
##   <.0001
##   <.0001
##   <.0001
```

```
##   <.0001
##   <.0001
##   0.0008
##   1.0000
##   0.9995
##   1.0000
##   1.0000
##   0.0378
##   <.0001
##   <.0001
##   <.0001
##   <.0001
##   0.0008
##   0.0078
##   0.0008
##   0.0008
##   0.9995
##   1.0000
##   1.0000
##   0.9995
##   0.9995
##   1.0000
##
## P value adjustment: sidak method for 66 tests
```

```
BM1_larvaeday4_emmeans_PAIRS <- as.data.frame(BM1_larvaeday4_emmeans_PAIRS)
write_xlsx(BM1_larvaeday4_emmeans_PAIRS, "Oviposition_eggsandlarvae/BM1_larvaeday4_emmeans_PAIRS.xlsx")

BM1_larvaeday4_emmeans_TEMP <- emmeans(model.larvaeday4.2,~Temperature,
                                       type="response")
```

```
## NOTE: Results may be misleading due to involvement in interactions
```

```
BM1_larvaeday4_emmeans_TEMP_PAIRS <- pairs(BM1_larvaeday4_emmeans_TEMP, adjust="sidak")
BM1_larvaeday4_emmeans_TEMP_PAIRS
```

```
##  contrast                      estimate   SE  df z.ratio p.value
##  Temperature27 - Temperature30    -1.60 2.02 Inf  -0.791  0.8138
##  Temperature27 - Temperature32     9.28 1.23 Inf   7.559  <.0001
##  Temperature30 - Temperature32    10.88 1.61 Inf   6.775  <.0001
##
## Results are averaged over the levels of: Age
## P value adjustment: sidak method for 3 tests
```

```
BM1_larvaeday4_emmeans_TEMP_PAIRS <- as.data.frame(BM1_larvaeday4_emmeans_TEMP_PAIRS)
write_xlsx(BM1_larvaeday4_emmeans_TEMP_PAIRS, "Oviposition_eggsandlarvae/BM1_oviposday3_emmeans_TEMP_PAIRS.
xlsx")

BM1_larvaeday4_emmeans_AGE <- emmeans(model.larvaeday4.2,~Age,
                                      type="response")
```

```
## NOTE: Results may be misleading due to involvement in interactions
```

```
BM1_larvaeday4_emmeans_AGE_PAIRS <- pairs(BM1_larvaeday4_emmeans_AGE, adjust="sidak")
BM1_larvaeday4_emmeans_AGE_PAIRS
```

```
##  contrast      estimate    SE  df z.ratio p.value
##  Age3 - Age5      -2.63 2.183 Inf  -1.206  0.7878
##  Age3 - Age10      2.19 1.960 Inf   1.116  0.8414
##  Age3 - Age15      8.71 1.750 Inf   4.977  <.0001
##  Age5 - Age10      4.82 1.532 Inf   3.147  0.0099
##  Age5 - Age15     11.35 1.463 Inf   7.755  <.0001
##  Age10 - Age15     6.52 0.959 Inf   6.801  <.0001
##
## Results are averaged over the levels of: Temperature
## P value adjustment: sidak method for 6 tests
```

```
BM1_larvaeday4_emmeans_AGE_PAIRS <- as.data.frame(BM1_larvaeday4_emmeans_AGE_PAIRS)
write_xlsx(BM1_larvaeday4_emmeans_AGE_PAIRS, "Oviposition_eggsandlarvae/BM1_larvaeday4_emmeans_AGE_PAIRS.xl
sx")

sink("Oviposition_eggsandlarvae/BM1_larvaeday4_modelparam.txt")
parameters::model_parameters(
  model.larvaeday4.2, exponentiate = TRUE, ci_method = "wald",
  effects = "all",
  component = "conditional",
  group_level = TRUE,
  verbose = FALSE
)
sink()
```

Analysis for percent eggs hatching into larvae

```
#################
#percent eggs that hatch to larvae:
hist(Fecundity_data_firstBM_ovipos$Percent_eggshatchedtolarv)
```

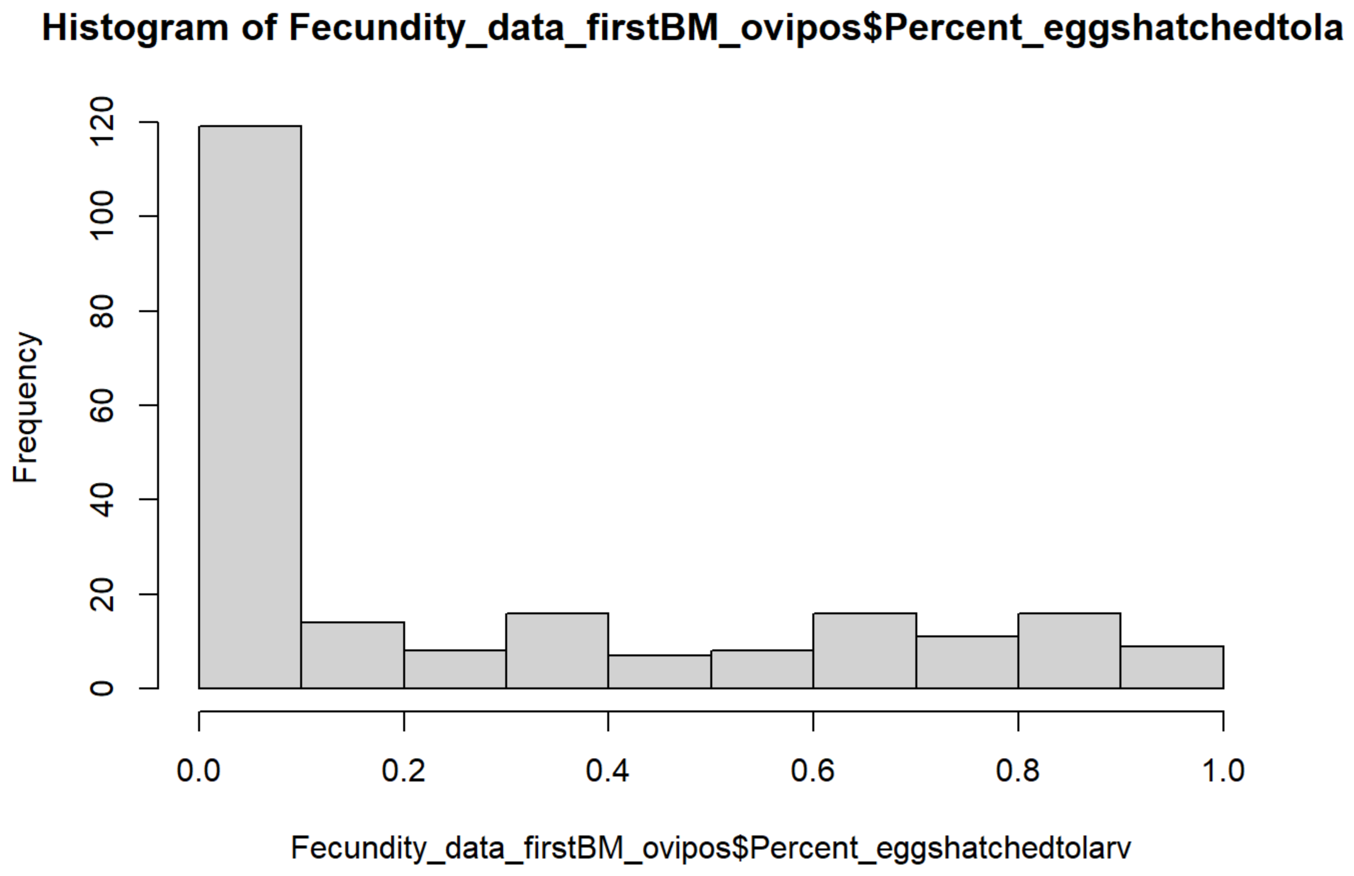


```
#non-normal
qqPlot(Fecundity_data_firstBM_ovipos$Percent_eggshatchedtolarv)
```

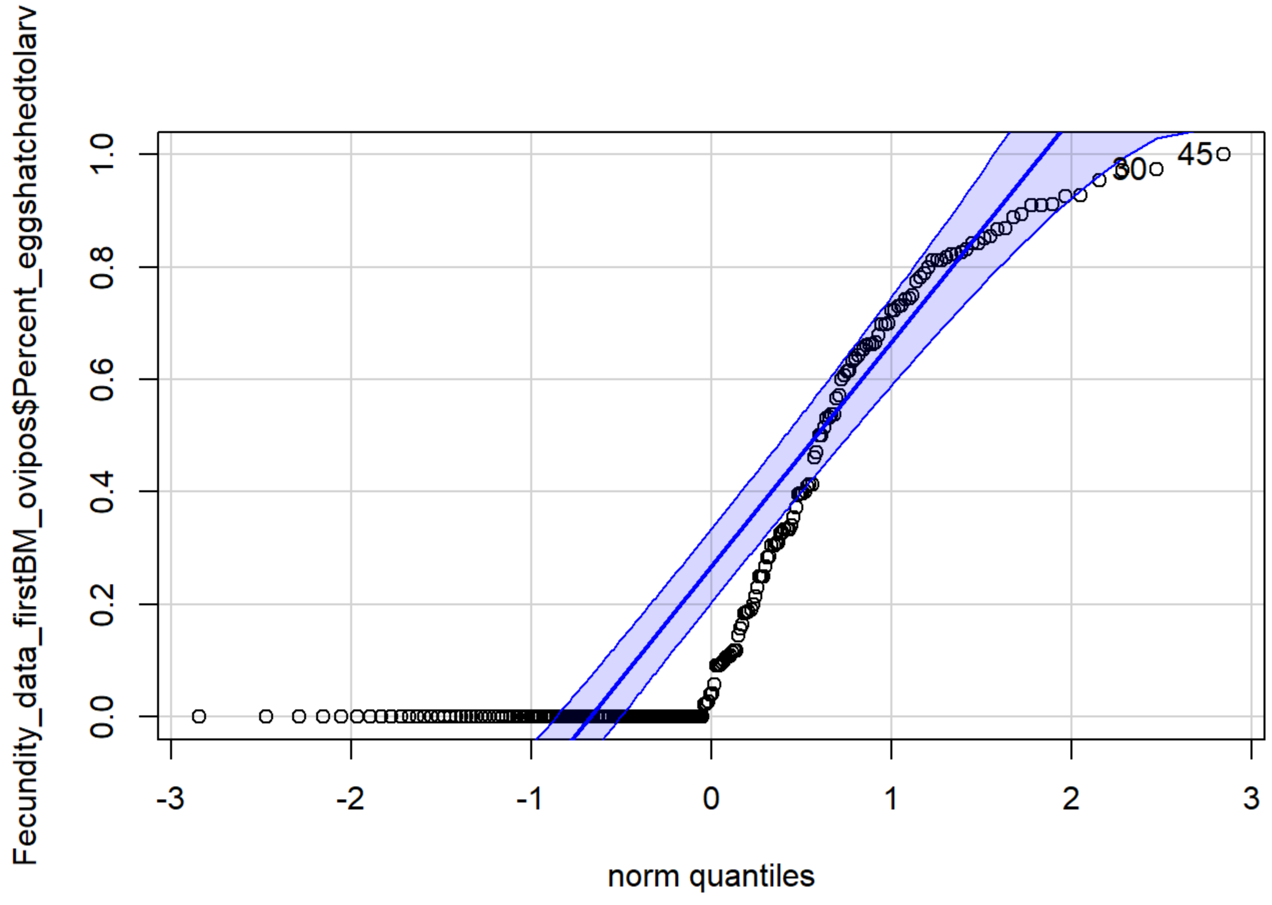

```
## [1] 45 30
```

```
#make temp and age a group and do non-parametric test
Fecundity_data_firstBM_ovipos$group <- paste(Fecundity_data_firstBM_ovipos$Temperature, Fecundity_data_firs
tBM_ovipos$Age, sep="_")
#ZEROS ARE INCLUDED.

#PERCENT EGGS DAY 3 THAT HATCH TO LARVAE DAY 4
#check to see if there are differences among groups using rank-based kruskal wallis test:
#kruskal chi squared test
sink("Oviposition_eggsandlarvae/Kruskaltest_BM1_percenteggshatched.txt")
kruskal.test(Fecundity_data_firstBM_ovipos$Percent_eggshatchedtolarv,
             Fecundity_data_firstBM_ovipos$group)
sink()

#Dunn's post hoc tests for groups:
dunn_tempwithinage <- Fecundity_data_firstBM_ovipos %>%
  group_by(Age)%>%
  dunn_test(Percent_eggshatchedtolarv ~ Temperature)
dunn_tempwithinage
```

```
## # A tibble: 12 × 10
##    Age   .y.    group1 group2    n1    n2 statistic       p   p.adj p.adj.signif
##  * <fct> <chr>  <chr>  <chr>  <int> <int>     <dbl>   <dbl>   <dbl> <chr>
##  1 3     Perce… 27     30        20    27    3.55   3.82e-4 0.00115 **
##  2 3     Perce… 27     32        20    15   -0.0738 9.41e-1 0.941   ns
##  3 3     Perce… 30     32        27    15   -3.33   8.60e-4 0.00172 **
##  4 5     Perce… 27     30        44    31   -0.174  8.62e-1 0.862   ns
##  5 5     Perce… 27     32        44     4   -2.33   2.01e-2 0.0602  ns
##  6 5     Perce… 30     32        31     4   -2.21   2.72e-2 0.0602  ns
##  7 10    Perce… 27     30        40    22   -2.31   2.08e-2 0.0624  ns
##  8 10    Perce… 27     32        40     2   -1.98   4.82e-2 0.0964  ns
##  9 10    Perce… 30     32        22     2   -1.11   2.68e-1 0.268   ns
## 10 15    Perce… 27     30        13     2   -0.441  6.59e-1 1       ns
## 11 15    Perce… 27     32        13     4   -0.586  5.58e-1 1       ns
## 12 15    Perce… 30     32         2     4    0      1   e+0 1       ns
```

```
write_xlsx(dunn_tempwithinage, "Oviposition_eggsandlarvae/Dunnposthoc_BM1_percenteggshatched_allpairwise_te
mpwithinage.xlsx")

dunn_agewithintemp <-Fecundity_data_firstBM_ovipos %>%
  group_by(Temperature)%>%
  dunn_test(Percent_eggshatchedtolarv ~ Age)
dunn_agewithintemp
```

```
## # A tibble: 18 × 10
##    Temperature .y.           group1 group2    n1    n2 statistic       p   p.adj
##  * <fct>       <chr>         <chr>  <chr>  <int> <int>     <dbl>   <dbl>   <dbl>
##  1 27          Percent_eggs… 3      5         20    44     3.98  6.98e-5 3.49e-4
##  2 27          Percent_eggs… 3      10        20    40     4.38  1.19e-5 7.13e-5
##  3 27          Percent_eggs… 3      15        20    13    -0.164 8.70e-1 1   e+0
##  4 27          Percent_eggs… 5      10        44    40     0.581 5.61e-1 1   e+0
##  5 27          Percent_eggs… 5      15        44    13    -3.58  3.41e-4 1.02e-3
##  6 27          Percent_eggs… 10     15        40    13    -3.94  8.16e-5 3.49e-4
##  7 30          Percent_eggs… 3      5         27    31     0.848 3.96e-1 7.92e-1
##  8 30          Percent_eggs… 3      10        27    22    -0.776 4.38e-1 7.92e-1
##  9 30          Percent_eggs… 3      15        27     2    -1.56  1.19e-1 5.47e-1
## 10 30          Percent_eggs… 5      10        31    22    -1.60  1.09e-1 5.47e-1
## 11 30          Percent_eggs… 5      15        31     2    -1.87  6.14e-2 3.68e-1
## 12 30          Percent_eggs… 10     15        22     2    -1.24  2.14e-1 6.41e-1
## 13 32          Percent_eggs… 3      5         15     4    -0.855 3.93e-1 1   e+0
## 14 32          Percent_eggs… 3      10        15     2    -0.639 5.23e-1 1   e+0
## 15 32          Percent_eggs… 3      15        15     4    -0.855 3.93e-1 1   e+0
## 16 32          Percent_eggs… 5      10         4     2     0     1   e+0 1   e+0
## 17 32          Percent_eggs… 5      15         4     4     0     1   e+0 1   e+0
## 18 32          Percent_eggs… 10     15         2     4     0     1   e+0 1   e+0
## # ℹ 1 more variable: p.adj.signif <chr>
```

```
write_xlsx(dunn_agewithintemp, "Oviposition_eggsandlarvae/Dunnposthoc_BM1_percenteggshatched_allpairwise_ag
ewithintemp.xlsx")

#main effects
dunn_hatched_temp <- Fecundity_data_firstBM_ovipos %>%
  dunn_test(Percent_eggshatchedtolarv ~ Temperature)
write_xlsx(dunn_hatched_temp, "Oviposition_eggsandlarvae/Dunnposthoc_BM1_percenteggshatched_pairwise_TEMP.x
lsx")

dunn_hatched_age <- Fecundity_data_firstBM_ovipos %>%
  dunn_test(Percent_eggshatchedtolarv ~ Age)

dunn_hatched_age
```

```
## # A tibble: 6 × 9
##   .y.           group1 group2    n1    n2 statistic       p   p.adj p.adj.signif
## * <chr>         <chr>  <chr>  <int> <int>     <dbl>   <dbl>   <dbl> <chr>
## 1 Percent_eggs… 3      5         62    79     3.95  7.68e-5 3.07e-4 ***
## 2 Percent_eggs… 3      10        62    64     3.61  3.08e-4 9.24e-4 ***
## 3 Percent_eggs… 3      15        62    19    -1.94  5.30e-2 1.06e-1 ns
## 4 Percent_eggs… 5      10        79    64    -0.166 8.68e-1 8.68e-1 ns
## 5 Percent_eggs… 5      15        79    19    -4.61  4.00e-6 2.40e-5 ****
## 6 Percent_eggs… 10     15        64    19    -4.40  1.07e-5 5.33e-5 ****
```

```
write_xlsx(dunn_hatched_age, "Oviposition_eggsandlarvae/Dunnposthoc_BM1_percenteggshatched_pairwise_AGE.xls
x")
```

Code for Fig 7: survival w/ or w/o access to oviposition site

Import data and clean it up.

```
# clear existing workspace
rm(list = ls(all = TRUE))
graphics.off()
shell("cls")

#set wd to your project folder
getwd() #check working directory
```

```
## [1] "C:/Users/linzm/OneDrive - Vanderbilt/Hillyer_Lab/Blood_feeding_project/Bloodfeeding"
```

```
#####################
#load libraries needed:
library(readxl)
library(writexl)
library(ggplot2)
library(dplyr)
library(tidyverse)
library(rstatix)
library(car)
library(ggpubr)
library(emmeans)
library(survival)
library(ggsurvfit)
library(survminer)

#######################################################
#import the data and clean it up:


#import the data:
Fecundity_data <- read_xlsx("SupplementaryData1_RawData.xlsx",
                             sheet = "Figs4-9")
Fecundity_data <- as.data.frame(Fecundity_data)
str(Fecundity_data)
```

```
## 'data.frame':    849 obs. of  18 variables:
##  $ ID_overall               : num  1 2 3 4 5 6 7 8 9 10 ...
##  $ Temperature              : num  32 32 32 32 32 32 32 32 32 32 ...
##  $ Age                      : num  3 3 3 3 3 3 3 3 3 3 ...
##  $ ID_per_group             : num  1 2 3 4 5 6 7 8 9 10 ...
##  $ Trial_start_date         : POSIXct, format: "2024-02-13" "2024-02-13" ...
##  $ Trial_number             : num  1 1 1 1 1 1 1 1 1 1 ...
##  $ BM1_Date                 : POSIXct, format: "2024-02-13" "2024-02-13" ...
##  $ Age_of_BM                : num  3 3 3 3 3 3 3 3 3 3 ...
##  $ Bloodmeal_number         : num  1 1 1 1 1 1 1 1 1 1 ...
##  $ Oviposition_positive(y/n): chr  "Y" "Y" "Y" "Y" ...
##  $ Eggs_day3                : chr  "0" "0" "0" "0" ...
##  $ Eggs_day4                : chr  "0" "0" "0" "0" ...
##  $ Larvae_day4              : chr  "0" "0" "0" "0" ...
##  $ Surv_to_eggs(y/n)        : chr  "Y" "Y" "Y" "Y" ...
##  $ Surv_to_larvae(y/n)      : chr  "Y" "Y" "Y" "Y" ...
##  $ Date_of_death            : POSIXct, format: "2024-03-01" "2024-03-07" ...
##  $ Censor                   : num  1 1 1 1 1 1 1 1 1 1 ...
##  $ Notes                    : chr  NA NA NA NA ...
```

```
head(Fecundity_data)
```

```
##   ID_overall Temperature Age ID_per_group Trial_start_date Trial_number
## 1          1          32   3            1       2024-02-13            1
## 2          2          32   3            2       2024-02-13            1
## 3          3          32   3            3       2024-02-13            1
## 4          4          32   3            4       2024-02-13            1
## 5          5          32   3            5       2024-02-13            1
## 6          6          32   3            6       2024-02-13            1
##     BM1_Date Age_of_BM Bloodmeal_number Oviposition_positive(y/n) Eggs_day3
## 1 2024-02-13         3                1                         Y         0
## 2 2024-02-13         3                1                         Y         0
## 3 2024-02-13         3                1                         Y         0
## 4 2024-02-13         3                1                         Y         0
## 5 2024-02-13         3                1                         Y         0
## 6 2024-02-13         3                1                         Y        14
##   Eggs_day4 Larvae_day4 Surv_to_eggs(y/n) Surv_to_larvae(y/n) Date_of_death
## 1         0           0                 Y                   Y    2024-03-01
## 2         0           0                 Y                   Y    2024-03-07
## 3         0           0                 Y                   Y    2024-03-02
## 4         0           0                 Y                   Y    2024-02-26
## 5         0           0                 Y                   Y    2024-02-28
## 6        14           0                 Y                   Y    2024-03-02
##   Censor Notes
## 1      1  <NA>
## 2      1  <NA>
## 3      1  <NA>
## 4      1  <NA>
## 5      1  <NA>
## 6      1  <NA>
```

```
Fecundity_data_numeric <- Fecundity_data

#variables of interest:

Fecundity_data$Temperature <- as.factor(Fecundity_data$Temperature)
Fecundity_data$Age <- as.factor(Fecundity_data$Age)
Fecundity_data$Age_of_BM <- as.numeric(Fecundity_data$Age_of_BM)
Fecundity_data$Bloodmeal_number <- as.factor(Fecundity_data$Bloodmeal_number)

Fecundity_data$Eggs_day3 <- as.numeric(Fecundity_data$Eggs_day3)
```

```
## Warning: NAs introduced by coercion
```

```
Fecundity_data$Eggs_day4 <- as.numeric(Fecundity_data$Eggs_day4)
```

```
## Warning: NAs introduced by coercion
```

```
Fecundity_data$Larvae_day4 <- as.numeric(Fecundity_data$Larvae_day4)
```

```
## Warning: NAs introduced by coercion
```

```
Fecundity_data$Oviposition_positive <- as.factor(Fecundity_data$Oviposition_positive)
Fecundity_data$`Surv_to_eggs(y/n)` <- as.factor(Fecundity_data$`Surv_to_eggs(y/n)`)
Fecundity_data$`Surv_to_larvae(y/n)` <- as.factor(Fecundity_data$`Surv_to_larvae(y/n)`)

str(Fecundity_data)
```

```
## 'data.frame':    849 obs. of  19 variables:
##  $ ID_overall              : num  1 2 3 4 5 6 7 8 9 10 ...
##  $ Temperature             : Factor w/ 3 levels "27","30","32": 3 3 3 3 3 3 3 3 3 3 ...
##  $ Age                     : Factor w/ 4 levels "3","5","10","15": 1 1 1 1 1 1 1 1 1 1 ...
##  $ ID_per_group            : num  1 2 3 4 5 6 7 8 9 10 ...
##  $ Trial_start_date        : POSIXct, format: "2024-02-13" "2024-02-13" ...
##  $ Trial_number            : num  1 1 1 1 1 1 1 1 1 1 ...
##  $ BM1_Date                : POSIXct, format: "2024-02-13" "2024-02-13" ...
##  $ Age_of_BM               : num  3 3 3 3 3 3 3 3 3 3 ...
##  $ Bloodmeal_number        : Factor w/ 1 level "1": 1 1 1 1 1 1 1 1 1 1 ...
##  $ Oviposition_positive(y/n): chr  "Y" "Y" "Y" "Y" ...
##  $ Eggs_day3               : num  0 0 0 0 0 14 NA NA NA NA ...
##  $ Eggs_day4               : num  0 0 0 0 0 14 NA NA NA NA ...
##  $ Larvae_day4             : num  0 0 0 0 0 0 0 0 NA NA ...
##  $ Surv_to_eggs(y/n)       : Factor w/ 3 levels "N","NA","Y": 3 3 3 3 3 3 1 1 3 3 ...
##  $ Surv_to_larvae(y/n)     : Factor w/ 3 levels "N","NA","Y": 3 3 3 3 3 3 2 2 3 3 ...
##  $ Date_of_death           : POSIXct, format: "2024-03-01" "2024-03-07" ...
##  $ Censor                  : num  1 1 1 1 1 1 1 1 1 1 ...
##  $ Notes                   : chr  NA NA NA NA ...
##  $ Oviposition_positive    : Factor w/ 2 levels "N","Y": 2 2 2 2 2 2 2 2 1 1 ...
```

```
Fecundity_data <- subset(Fecundity_data, Censor == 1) #get rid of mosquitoes censored out by experimental e
rror (get rid of 0 values; 1 = died naturally)

#calculate the total eggs laid per mosquito:

#need to subtract to find ones only laid on day 4 (exclude day 3 eggs)
Fecundity_data$Eggs_day4 <- (Fecundity_data$Eggs_day4)-(Fecundity_data$Eggs_day3)

#replace negative eggs day 4 values with zero (assume miscounted/eggs degraded and no new eggs laid)
for (row in 1:nrow(Fecundity_data)){
  if (is.na(Fecundity_data$Eggs_day4[row])){
    Fecundity_data$Eggs_day4[row] <- NA #keep NA values
  } else if ((Fecundity_data$Eggs_day4[row] <= 0)){
    Fecundity_data$Eggs_day4[row] <- 0
  }
}

#total eggs addition:
Fecundity_data$total_eggs <- (Fecundity_data$Eggs_day3)+(Fecundity_data$Eggs_day4)

#percents:
Fecundity_data$Percent_eggs_day3 <- (Fecundity_data$Eggs_day3) / (Fecundity_data$total_eggs)
Fecundity_data$Percent_eggs_day4 <- (Fecundity_data$Eggs_day4) / (Fecundity_data$total_eggs)

# decide if each mosquito laid eggs and on what day
#day 3
for (row in 1:nrow(Fecundity_data)){
  if(!is.na(Fecundity_data$Eggs_day3[row])){
    if (Fecundity_data$Eggs_day3[row] > 0){
      Fecundity_data$egg_binary[row] = 1
      Fecundity_data$egg_binary_day3[row] = 1
    }
    else {
      Fecundity_data$egg_binary[row] = 0
      Fecundity_data$egg_binary_day3[row] = 0
    }
  }
  else if (is.na(Fecundity_data$Eggs_day3[row])){
    Fecundity_data$egg_binary[row] = NA
    Fecundity_data$egg_binary_day3[row] = NA
  }
}

#day 4
for (row in 1:nrow(Fecundity_data)){
  if(!is.na(Fecundity_data$Eggs_day4[row])){
    if (Fecundity_data$Eggs_day4[row] >0){
      Fecundity_data$egg_binary[row] = 1
      Fecundity_data$egg_binary_day4[row] = 1
    }
    else{
      Fecundity_data$egg_binary_day4[row] = 0
    }
  }
  else if (is.na(Fecundity_data$Eggs_day4[row])){
    Fecundity_data$egg_binary_day4[row] = NA
```

```
  }
}

# decide if each mosquito had larvae
for (row in 1:nrow(Fecundity_data)){
  if(!is.na(Fecundity_data$Larvae_day4[row])){
    if (Fecundity_data$Larvae_day4[row] >0){
      Fecundity_data$larvae_binary[row] = 1
    }
    else{
      Fecundity_data$larvae_binary[row] = 0
    }
  }
  else{
    Fecundity_data$larvae_binary[row] = NA
  }
}

#percents:
Fecundity_data$Percent_eggshatchedtolarv <- (Fecundity_data$Larvae_day4) / (Fecundity_data$Eggs_day3)

#survival:
#calculate:
Fecundity_data$Days_to_death_post_BM <- Fecundity_data$Date_of_death - Fecundity_data$Trial_start_date
Fecundity_data$Age_of_death <- Fecundity_data$Age_of_BM + Fecundity_data$Days_to_death_post_BM

Fecundity_data <-
  Fecundity_data %>%
  mutate(
    Age_of_BM_days = as.difftime(Age_of_BM, unit="days")
  )
Fecundity_data$Date_of_eclosion <- Fecundity_data$Trial_start_date - (Fecundity_data$Age_of_BM_days)

library(lubridate)
Fecundity_data <-
  Fecundity_data %>%
  mutate(
    days_alive_post_BM = as.duration(Trial_start_date %--% Date_of_death) / ddays(1),
    days_alive_post_eclosion = as.duration(Date_of_eclosion %--% Date_of_death) / ddays(1),
  )

str(Fecundity_data)
```

```
## 'data.frame':    842 obs. of  33 variables:
##  $ ID_overall               : num  1 2 3 4 5 6 7 8 9 10 ...
##  $ Temperature              : Factor w/ 3 levels "27","30","32": 3 3 3 3 3 3 3 3 3 3 ...
##  $ Age                      : Factor w/ 4 levels "3","5","10","15": 1 1 1 1 1 1 1 1 1 1 ...
##  $ ID_per_group             : num  1 2 3 4 5 6 7 8 9 10 ...
##  $ Trial_start_date         : POSIXct, format: "2024-02-13" "2024-02-13" ...
##  $ Trial_number             : num  1 1 1 1 1 1 1 1 1 1 ...
##  $ BM1_Date                 : POSIXct, format: "2024-02-13" "2024-02-13" ...
##  $ Age_of_BM                : num  3 3 3 3 3 3 3 3 3 3 ...
##  $ Bloodmeal_number         : Factor w/ 1 level "1": 1 1 1 1 1 1 1 1 1 1 ...
##  $ Oviposition_positive(y/n): chr  "Y" "Y" "Y" "Y" ...
##  $ Eggs_day3                : num  0 0 0 0 0 14 NA NA NA NA ...
##  $ Eggs_day4                : num  0 0 0 0 0 0 NA NA NA NA ...
##  $ Larvae_day4              : num  0 0 0 0 0 0 0 0 NA NA ...
##  $ Surv_to_eggs(y/n)        : Factor w/ 3 levels "N","NA","Y": 3 3 3 3 3 3 1 1 3 3 ...
##  $ Surv_to_larvae(y/n)      : Factor w/ 3 levels "N","NA","Y": 3 3 3 3 3 3 2 2 3 3 ...
##  $ Date_of_death            : POSIXct, format: "2024-03-01" "2024-03-07" ...
##  $ Censor                   : num  1 1 1 1 1 1 1 1 1 1 ...
##  $ Notes                    : chr  NA NA NA NA ...
##  $ Oviposition_positive     : Factor w/ 2 levels "N","Y": 2 2 2 2 2 2 2 2 1 1 ...
##  $ total_eggs               : num  0 0 0 0 0 14 NA NA NA NA ...
##  $ Percent_eggs_day3        : num  NaN NaN NaN NaN NaN 1 NA NA NA NA ...
##  $ Percent_eggs_day4        : num  NaN NaN NaN NaN NaN 0 NA NA NA NA ...
##  $ egg_binary               : num  0 0 0 0 0 1 NA NA NA NA ...
##  $ egg_binary_day3          : num  0 0 0 0 0 1 NA NA NA NA ...
##  $ egg_binary_day4          : num  0 0 0 0 0 0 NA NA NA NA ...
##  $ larvae_binary            : num  0 0 0 0 0 0 0 0 NA NA ...
##  $ Percent_eggshatchedtolarv: num  NaN NaN NaN NaN NaN 0 NA NA NA NA ...
##  $ Days_to_death_post_BM    : 'difftime' num  17 23 18 13 ...
##   ..- attr(*, "units")= chr "days"
##  $ Age_of_death             : 'difftime' num  20 26 21 16 ...
##   ..- attr(*, "units")= chr "days"
##  $ Age_of_BM_days           : 'difftime' num  3 3 3 3 ...
##   ..- attr(*, "units")= chr "days"
##  $ Date_of_eclosion         : POSIXct, format: "2024-02-10" "2024-02-10" ...
##  $ days_alive_post_BM       : num  17 23 18 13 15 18 3 3 12 11 ...
##  $ days_alive_post_eclosion : num  20 26 21 16 18 21 6 6 15 14 ...
```

```
Fecundity_data_firstBM <- Fecundity_data
#subset by oviposition positive and negative:
Fecundity_data_ovipos <- subset(Fecundity_data, Oviposition_positive== "Y")
Fecundity_data_ovineg <- subset(Fecundity_data, Oviposition_positive== "N")
```

Plot survival curves

```
library(survival)
# Plot survival curves
#labels first
Fecundity_data_firstBM$group <- paste(Fecundity_data_firstBM$Age,Fecundity_data_firstBM$Temperature,sep
="_")

Fecundity_data_firstBM$Age <- factor(Fecundity_data_firstBM$Age,
                                     labels = c("3 days","5 days","10 days","15 days"))
Fecundity_data_firstBM$Temperature <- factor(Fecundity_data_firstBM$Temperature,
                                             labels = c("27˚C","30˚C","32˚C"))



str(Fecundity_data_firstBM) #make sure dates are dates
```

```
## 'data.frame':    842 obs. of  34 variables:
##  $ ID_overall              : num  1 2 3 4 5 6 7 8 9 10 ...
##  $ Temperature             : Factor w/ 3 levels "27˚C","30˚C",..: 3 3 3 3 3 3 3 3 3 3 ...
##  $ Age                     : Factor w/ 4 levels "3 days","5 days",..: 1 1 1 1 1 1 1 1 1 1 ...
##  $ ID_per_group            : num  1 2 3 4 5 6 7 8 9 10 ...
##  $ Trial_start_date        : POSIXct, format: "2024-02-13" "2024-02-13" ...
##  $ Trial_number            : num  1 1 1 1 1 1 1 1 1 1 ...
##  $ BM1_Date                : POSIXct, format: "2024-02-13" "2024-02-13" ...
##  $ Age_of_BM               : num  3 3 3 3 3 3 3 3 3 3 ...
##  $ Bloodmeal_number        : Factor w/ 1 level "1": 1 1 1 1 1 1 1 1 1 1 ...
##  $ Oviposition_positive(y/n): chr  "Y" "Y" "Y" "Y" ...
##  $ Eggs_day3               : num  0 0 0 0 0 14 NA NA NA NA ...
##  $ Eggs_day4               : num  0 0 0 0 0 0 NA NA NA NA ...
##  $ Larvae_day4             : num  0 0 0 0 0 0 0 0 NA NA ...
##  $ Surv_to_eggs(y/n)       : Factor w/ 3 levels "N","NA","Y": 3 3 3 3 3 3 1 1 3 3 ...
##  $ Surv_to_larvae(y/n)     : Factor w/ 3 levels "N","NA","Y": 3 3 3 3 3 3 2 2 3 3 ...
##  $ Date_of_death           : POSIXct, format: "2024-03-01" "2024-03-07" ...
##  $ Censor                  : num  1 1 1 1 1 1 1 1 1 1 ...
##  $ Notes                   : chr  NA NA NA NA ...
##  $ Oviposition_positive    : Factor w/ 2 levels "N","Y": 2 2 2 2 2 2 2 2 1 1 ...
##  $ total_eggs              : num  0 0 0 0 0 14 NA NA NA NA ...
##  $ Percent_eggs_day3       : num  NaN NaN NaN NaN NaN 1 NA NA NA NA ...
##  $ Percent_eggs_day4       : num  NaN NaN NaN NaN NaN 0 NA NA NA NA ...
##  $ egg_binary              : num  0 0 0 0 0 1 NA NA NA NA ...
##  $ egg_binary_day3         : num  0 0 0 0 0 1 NA NA NA NA ...
##  $ egg_binary_day4         : num  0 0 0 0 0 0 NA NA NA NA ...
##  $ larvae_binary           : num  0 0 0 0 0 0 0 0 NA NA ...
##  $ Percent_eggshatchedtolarv: num  NaN NaN NaN NaN NaN 0 NA NA NA NA ...
##  $ Days_to_death_post_BM   : 'difftime' num  17 23 18 13 ...
##   ..- attr(*, "units")= chr "days"
##  $ Age_of_death            : 'difftime' num  20 26 21 16 ...
##   ..- attr(*, "units")= chr "days"
##  $ Age_of_BM_days          : 'difftime' num  3 3 3 3 ...
##   ..- attr(*, "units")= chr "days"
##  $ Date_of_eclosion        : POSIXct, format: "2024-02-10" "2024-02-10" ...
##  $ days_alive_post_BM      : num  17 23 18 13 15 18 3 3 12 11 ...
##  $ days_alive_post_eclosion : num  20 26 21 16 18 21 6 6 15 14 ...
##  $ group                   : chr  "3_32" "3_32" "3_32" "3_32" ...
```

```
Fecundity_data_firstBM$start_time <- (Fecundity_data_firstBM$Age_of_BM +1) #start monitoring the day after
BM
Fecundity_data_firstBM$stop_time <- (Fecundity_data_firstBM$days_alive_post_eclosion+1) #age of death + 1 #
aod can't be sooner than start
Fecundity_data_firstBM$stopminusstart <- Fecundity_data_firstBM$stop_time - Fecundity_data_firstBM$start_ti
me

str(Fecundity_data_firstBM$Oviposition_positive)
```

```
##  Factor w/ 2 levels "N","Y": 2 2 2 2 2 2 2 2 1 1 ...
```

```
Fecundity_data_firstBM_ovipossurvival <- subset(Fecundity_data_firstBM, Oviposition_positive=="Y")

s1 <- survfit2(Surv(stopminusstart, Censor) ~ Temperature+Age+Oviposition_positive,
               data = Fecundity_data_firstBM)

ggsurv <- ggsurvplot(s1, conf.int = TRUE, color="Age",palette = c("#DCD1E9","#BAA4D3","#9776BE","#7549A8"),
                     ggtheme = theme_pubr(),surv.median.line = "v",confint=TRUE)

s1table <- as.data.frame(ggsurv$data.survplot)

firstBM_survival_oviposition_positive_vs_negative_stopminusstart <- ggsurv$plot +
  theme_pubr() +
  theme(legend.position = "bottom")+
  facet_grid(Temperature~Oviposition_positive)+
  ylab(expression("BM1 Survival probability"))+
  xlab("Time (Days post blood feeding)") +
  theme(panel.background = element_rect(fill = NA, color = "black"))+
  theme(panel.spacing = unit(0.6, "lines"))
firstBM_survival_oviposition_positive_vs_negative_stopminusstart
```

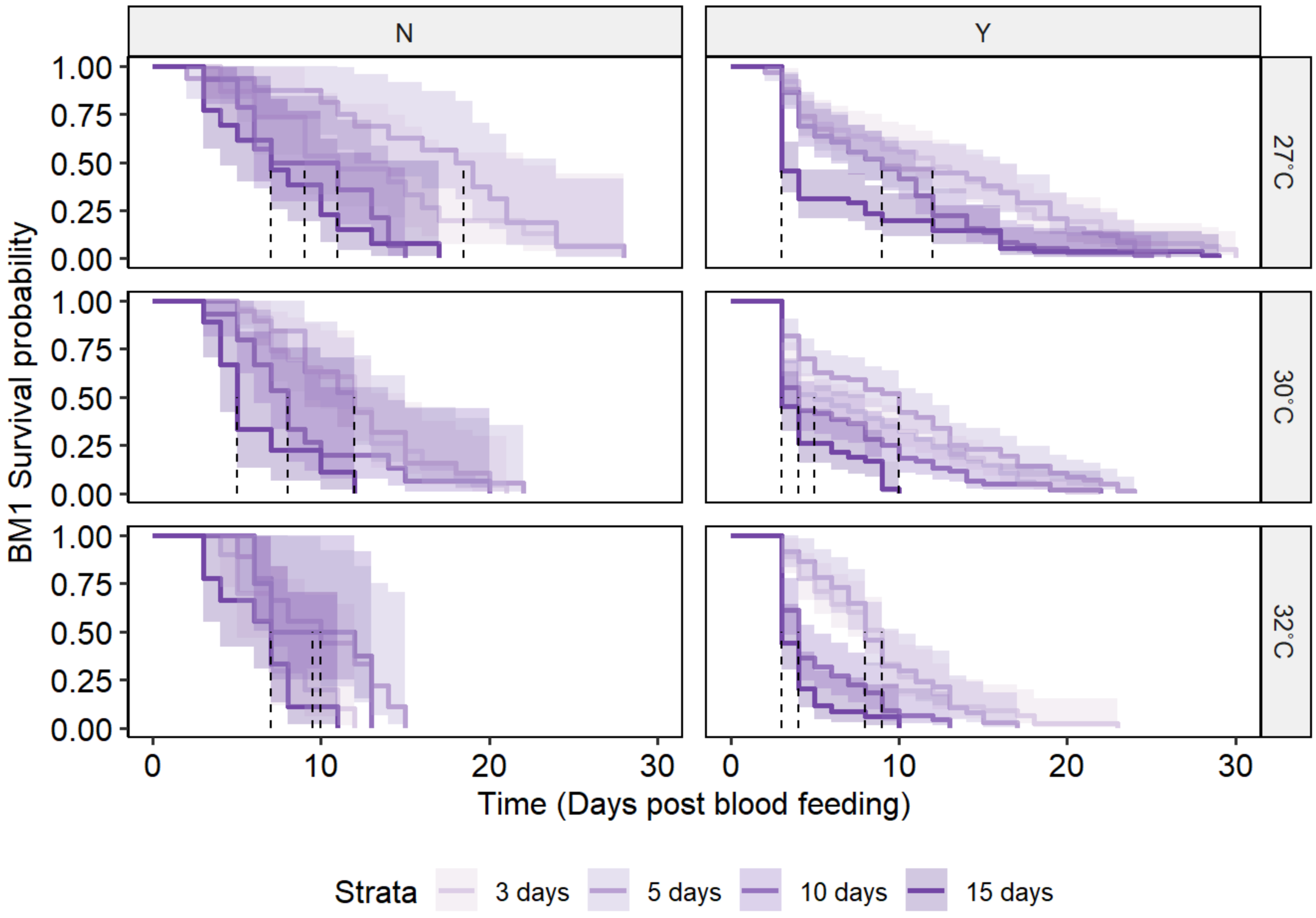


```
ggsave(filename = "Oviposition_survival/firstBM_survival_oviposition_positive_vs_negative_stopminusstart.pn
g",plot=firstBM_survival_oviposition_positive_vs_negative_stopminusstart,  width = 6, height = 5,units="i
n",dpi = 600)
ggsave(filename = "Oviposition_survival/firstBM_survival_oviposition_positive_vs_negative_stopminusstart.pd
f",plot=firstBM_survival_oviposition_positive_vs_negative_stopminusstart,  width = 6, height = 5,units="i
n",dpi = 600)




ggsurv <- ggsurvplot(s1, conf.int = TRUE, color="Temperature",palette = c("#4D6FAE","#6F9F51", "#CC763B"),
                     ggtheme = theme_pubr(),surv.median.line = "v",confint=TRUE)

firstBM_survival_oviposition_positive_vs_negative_flipped_stopminusstart <- ggsurv$plot +
  theme_pubr() +
  theme(legend.position = "bottom")+
  facet_grid(Age ~ Oviposition_positive)+
  ylab(expression("BM1 Survival probability"))+
  xlab("Time (Days post blood feeding)") +
  theme(panel.background = element_rect(fill = NA, color = "black"))+
  theme(panel.spacing = unit(0.6, "lines"))
firstBM_survival_oviposition_positive_vs_negative_flipped_stopminusstart
```

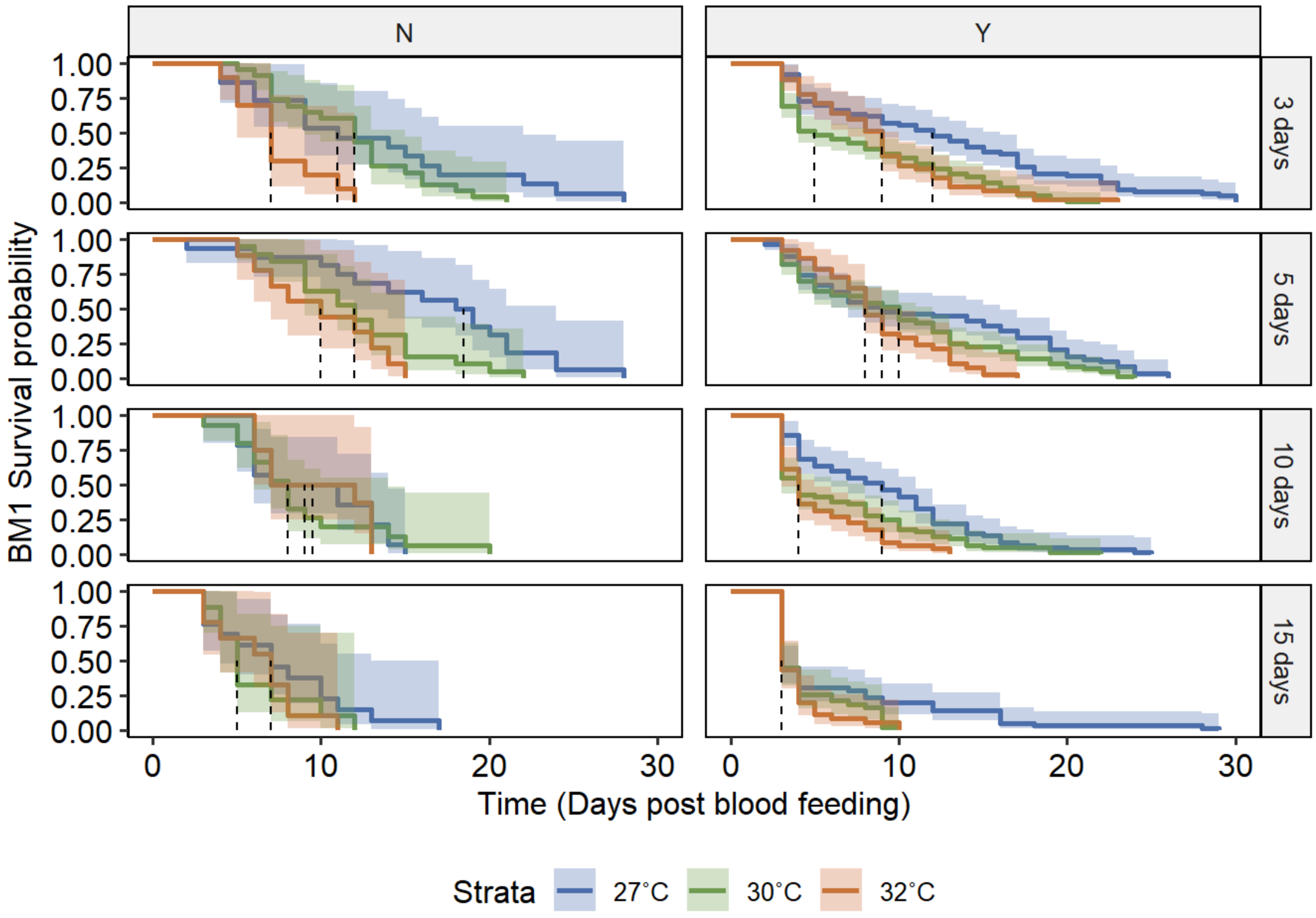

N
Y
3 days
5 days
10 days
15 days
BM1 Survival probability
1.00
0.75
0.50
0.25
0.00
0
10
20
30
Time (Days post blood feeding)
Strata
27°C
30°C
32°C

```
ggsave(filename = "Oviposition_survival/firstBM_survival_oviposition_positive_vs_negative_flipped_stopminus
start.png",plot=firstBM_survival_oviposition_positive_vs_negative_flipped_stopminusstart,  width = 6, heigh
t = 5,units="in",dpi = 600)
ggsave(filename = "Oviposition_survival/firstBM_survival_oviposition_positive_vs_negative_flipped_stopminus
start.pdf",plot=firstBM_survival_oviposition_positive_vs_negative_flipped_stopminusstart,  width = 6, heigh
t = 5,units="in",dpi = 600)

ggsurv <- ggsurvplot(s1, conf.int = TRUE,color="Temperature",palette = c("#4D6FAE","#6F9F51", "#CC763B"),
                     ggtheme = theme_pubr(),surv.median.line = "v",confint=TRUE)

png(filename = "firstBM_survival_oviposition_positive_vs_negative2_flipped.png", width = 6, height = 5, uni
ts = "in", res = 600)
firstBM_survival_oviposition_positive_vs_negative2_flipped <- ggsurv$plot +
  theme_pubr() +
  theme(legend.position = "bottom")+
  facet_grid(Age ~ Oviposition_positive)+
  ylab(expression("BM1 Survival probability"))+
  xlab("Time (Days post blood feeding)") +
  theme(panel.background = element_rect(fill = NA, color = "black"))+
  theme(panel.spacing = unit(0.6, "lines"))
firstBM_survival_oviposition_positive_vs_negative2_flipped
ggsave(filename = "Oviposition_survival/firstBM_survival_oviposition_positive_vs_negative2_flipped.png",plo
t=firstBM_survival_oviposition_positive_vs_negative2_flipped,  width = 6, height = 5,units="in",dpi = 600)
ggsave(filename = "Oviposition_survival/firstBM_survival_oviposition_positive_vs_negative2_flipped.pdf",plo
t=firstBM_survival_oviposition_positive_vs_negative2_flipped,  width = 6, height = 5,units="in",dpi = 600)

#this one compares ovipos vs ovineg within each temp and age:
ggsurv <- ggsurvplot(s1, conf.int = TRUE,color="Oviposition_positive",palette="Paired",
                     ggtheme = theme_pubr(),surv.median.line = "v",confint=TRUE)

firstBM_survival_oviposition_positive_vs_negative3 <- ggsurv$plot +
  theme_pubr() +
  theme(legend.position = "none")+
  facet_grid(Temperature~Age)+
  ylab(expression("BM1 Survival probability"))+
  xlab("Time (Days post blood feeding)") +
  theme(panel.background = element_rect(fill = NA, color = "black"))+
  theme(panel.spacing = unit(0.6, "lines"))
firstBM_survival_oviposition_positive_vs_negative3
ggsave("Oviposition_survival/firstBM_survival_oviposition_positive_vs_negative3_stopminusstart.pdf", plot =
firstBM_survival_oviposition_positive_vs_negative3, width = 6, height = 4, units = "in",dpi=600)
ggsave("Oviposition_survival/firstBM_survival_oviposition_positive_vs_negative3_stopminusstart.png", plot =
firstBM_survival_oviposition_positive_vs_negative3, width = 6, height = 4, units = "in",dpi=600)

firstBM_survival_oviposition_positive_vs_negative3flip <- ggsurv$plot +
  theme_pubr() +
  theme(legend.position = "bottom")+
  facet_grid(Age~Temperature)+
  ylab(expression("BM1 Survival probability"))+
  xlab("Time (Days post blood feeding)") +
  theme(panel.background = element_rect(fill = NA, color = "black"))+
  theme(panel.spacing = unit(0.6, "lines"))
firstBM_survival_oviposition_positive_vs_negative3flip
ggsave("Oviposition_survival/firstBM_survival_oviposition_positive_vs_negative3flip_stopminusstart.pdf", pl
ot = firstBM_survival_oviposition_positive_vs_negative3flip, width = 6.5, height = 5, units = "in",dpi=600)
ggsave("Oviposition_survival/firstBM_survival_oviposition_positive_vs_negative3flip_stopminusstart.png", pl
```

```
ot = firstBM_survival_oviposition_positive_vs_negative3flip, width = 6.5, height = 5, units = "in",dpi=600)

#now check main effect of oviposition positive:
s2 <- survfit2(Surv(stopminusstart, Censor) ~ Oviposition_positive,
               data = Fecundity_data_firstBM)

ggsurv <- ggsurvplot(s2, conf.int = TRUE, color="Oviposition_positive",palette = "Paired",
                     ggtheme = theme_pubr(),surv.median.line = "v",confint=TRUE)

firstBM_survival_oviposition_positive_vs_negative_onlyposneg <- ggsurv$plot +
  theme_pubr() +
  theme(legend.position = "bottom")+
  ylab(expression("BM1 Survival probability"))+
  xlab("Time (Days post blood feeding)") +
  theme(panel.background = element_rect(fill = NA, color = "black"))+
  theme(panel.spacing = unit(0.6, "lines"))
firstBM_survival_oviposition_positive_vs_negative_onlyposneg

ggsave("Oviposition_survival/firstBM_survival_oviposition_positive_vs_negative_onlyposneg_stopminusstart.pd
f", plot = firstBM_survival_oviposition_positive_vs_negative_onlyposneg, width = 5, height =4, units = "i
n",dpi=600)
ggsave("Oviposition_survival/firstBM_survival_oviposition_positive_vs_negative_onlyposneg_stopminusstart.pn
g", plot = firstBM_survival_oviposition_positive_vs_negative_onlyposneg, width = 5, height = 4, units = "i
n",dpi=600)


s3 <- survfit2(Surv(stopminusstart, Censor) ~ Oviposition_positive+Temperature,
               data = Fecundity_data_firstBM)

ggsurv <- ggsurvplot(s3, conf.int = TRUE, color="Temperature",palette = c("#4D6FAE","#6F9F51", "#CC763B"),
                     ggtheme = theme_pubr(),surv.median.line = "v",confint=TRUE)

firstBM_survival_oviposition_positive_vs_negative_TEMP <- ggsurv$plot +
  theme_pubr() +
  theme(legend.position = "bottom")+
  facet_grid(~ Oviposition_positive)+
  ylab(expression("BM1 Survival probability"))+
  xlab("Time (Days post blood feeding)") +
  theme(panel.background = element_rect(fill = NA, color = "black"))+
  theme(panel.spacing = unit(0.6, "lines"))
firstBM_survival_oviposition_positive_vs_negative_TEMP
ggsave("Oviposition_survival/firstBM_survival_oviposition_positive_vs_negative_TEMP_stopminusstart.png", pl
ot = firstBM_survival_oviposition_positive_vs_negative_TEMP, width = 6, height = 5, units = "in",dpi=600)
ggsave("Oviposition_survival/firstBM_survival_oviposition_positive_vs_negative_TEMP_stopminusstart.pdf", pl
ot = firstBM_survival_oviposition_positive_vs_negative_TEMP, width = 6, height = 5, units = "in",dpi=600)

ggsurv <- ggsurvplot(s3, conf.int = TRUE, color="Oviposition_positive",palette = "Paired",
                     ggtheme = theme_pubr(),surv.median.line = "v",confint=TRUE)

firstBM_survival_oviposition_positive_vs_negative_TEMP2 <- ggsurv$plot +
  theme_pubr() +
  theme(legend.position = "bottom")+
  facet_grid(~ Temperature)+
  ylab(expression("BM1 Survival probability"))+
  xlab("Time (Days post blood feeding)") +
  theme(panel.background = element_rect(fill = NA, color = "black"))+
```

```
  theme(panel.spacing = unit(0.6, "lines"))

firstBM_survival_oviposition_positive_vs_negative_TEMP2
ggsave("Oviposition_survival/firstBM_survival_oviposition_positive_vs_negative_TEMP2_stopminusstart.pdf", p
lot = firstBM_survival_oviposition_positive_vs_negative_TEMP2, width = 6, height = 5, units = "in",dpi=600)
ggsave("Oviposition_survival/firstBM_survival_oviposition_positive_vs_negative_TEMP2_stopminusstart.png", p
lot = firstBM_survival_oviposition_positive_vs_negative_TEMP2, width = 6, height = 5, units = "in",dpi=600)

s4 <- survfit2(Surv(stopminusstart, Censor) ~ Oviposition_positive+Age,
               data = Fecundity_data_firstBM)

ggsurv <- ggsurvplot(s4, conf.int = TRUE, color="Age",palette = c("#DCD1E9","#BAA4D3","#9776BE","#7549A
8"),,
                     ggtheme = theme_pubr(),surv.median.line = "v",confint=TRUE)

firstBM_survival_oviposition_positive_vs_negative_AGE <- ggsurv$plot +
  theme_pubr() +
  theme(legend.position = "bottom")+
  facet_grid(~ Oviposition_positive)+
  ylab(expression("BM1 Survival probability"))+
  xlab("Time (Days post blood feeding)") +
  theme(panel.background = element_rect(fill = NA, color = "black"))+
  theme(panel.spacing = unit(0.6, "lines"))
firstBM_survival_oviposition_positive_vs_negative_AGE

ggsave("Oviposition_survival/firstBM_survival_oviposition_positive_vs_negative_AGE_stopminusstart.pdf", plo
t = firstBM_survival_oviposition_positive_vs_negative_AGE, width = 6, height = 5, units = "in",dpi=600)
ggsave("Oviposition_survival/firstBM_survival_oviposition_positive_vs_negative_AGE_stopminusstart.png", plo
t = firstBM_survival_oviposition_positive_vs_negative_AGE, width = 6, height = 5, units = "in",dpi=600)

ggsurv <- ggsurvplot(s4, conf.int = TRUE,color="Oviposition_positive",palette = "Paired",
                     ggtheme = theme_pubr(),surv.median.line = "v",confint=TRUE)

firstBM_survival_oviposition_positive_vs_negative_AGE2 <- ggsurv$plot +
  theme_pubr() +
  theme(legend.position = "bottom")+
  facet_grid(~ Age)+
  ylab(expression("BM1 Survival probability"))+
  xlab("Time (Days post blood feeding)") +
  theme(panel.background = element_rect(fill = NA, color = "black"))+
  theme(panel.spacing = unit(0.6, "lines"))

ggsave("Oviposition_survival/firstBM_survival_oviposition_positive_vs_negative_AGE2_stopminusstart.pdf", pl
ot = firstBM_survival_oviposition_positive_vs_negative_AGE2, width = 6.5, height = 5, units = "in",dpi=600)
ggsave("Oviposition_survival/firstBM_survival_oviposition_positive_vs_negative_AGE2_stopminusstart.png", pl
ot = firstBM_survival_oviposition_positive_vs_negative_AGE2, width = 6.5, height = 5, units = "in",dpi=600)


s1
```

```
## Call: survfit(formula = Surv(stopminusstart, Censor) ~ Temperature +
##     Age + Oviposition_positive, data = Fecundity_data_firstBM)
##
##                                                          n events median 0.95LCL
## Temperature=27˚C, Age=3 days , Oviposition_positive=N  15     15   11.0       9
## Temperature=27˚C, Age=3 days , Oviposition_positive=Y  63     63   12.0       9
## Temperature=27˚C, Age=5 days , Oviposition_positive=N  16     16   18.5      12
## Temperature=27˚C, Age=5 days , Oviposition_positive=Y  58     58    9.0       7
## Temperature=27˚C, Age=10 days, Oviposition_positive=N  14     14    9.0       6
## Temperature=27˚C, Age=10 days, Oviposition_positive=Y  58     58    9.0       6
## Temperature=27˚C, Age=15 days, Oviposition_positive=N  13     13    7.0       4
## Temperature=27˚C, Age=15 days, Oviposition_positive=Y  55     55    3.0       3
## Temperature=30˚C, Age=3 days , Oviposition_positive=N  23     23   12.0       9
## Temperature=30˚C, Age=3 days , Oviposition_positive=Y 103    103    5.0       4
## Temperature=30˚C, Age=5 days , Oviposition_positive=N  19     19   12.0       9
## Temperature=30˚C, Age=5 days , Oviposition_positive=Y  83     83   10.0       7
## Temperature=30˚C, Age=10 days, Oviposition_positive=N  15     15    8.0       6
## Temperature=30˚C, Age=10 days, Oviposition_positive=Y  60     60    4.0       3
## Temperature=30˚C, Age=15 days, Oviposition_positive=N   9      9    5.0       4
## Temperature=30˚C, Age=15 days, Oviposition_positive=Y  42     42    3.0       3
## Temperature=32˚C, Age=3 days , Oviposition_positive=N  10     10    7.0       5
## Temperature=32˚C, Age=3 days , Oviposition_positive=Y  45     45    9.0       7
## Temperature=32˚C, Age=5 days , Oviposition_positive=N   9      9   10.0       7
## Temperature=32˚C, Age=5 days , Oviposition_positive=Y  37     37    8.0       8
## Temperature=32˚C, Age=10 days, Oviposition_positive=N   8      8    9.5       7
## Temperature=32˚C, Age=10 days, Oviposition_positive=Y  44     44    4.0       3
## Temperature=32˚C, Age=15 days, Oviposition_positive=N   9      9    7.0       4
## Temperature=32˚C, Age=15 days, Oviposition_positive=Y  34     34    3.0       3
##                                                       0.95UCL
## Temperature=27˚C, Age=3 days , Oviposition_positive=N      22
## Temperature=27˚C, Age=3 days , Oviposition_positive=Y      16
## Temperature=27˚C, Age=5 days , Oviposition_positive=N      24
## Temperature=27˚C, Age=5 days , Oviposition_positive=Y      16
## Temperature=27˚C, Age=10 days, Oviposition_positive=N      14
## Temperature=27˚C, Age=10 days, Oviposition_positive=Y      11
## Temperature=27˚C, Age=15 days, Oviposition_positive=N      NA
## Temperature=27˚C, Age=15 days, Oviposition_positive=Y       4
## Temperature=30˚C, Age=3 days , Oviposition_positive=N      15
## Temperature=30˚C, Age=3 days , Oviposition_positive=Y       8
## Temperature=30˚C, Age=5 days , Oviposition_positive=N      15
## Temperature=30˚C, Age=5 days , Oviposition_positive=Y      12
## Temperature=30˚C, Age=10 days, Oviposition_positive=N      14
## Temperature=30˚C, Age=10 days, Oviposition_positive=Y       8
## Temperature=30˚C, Age=15 days, Oviposition_positive=N      NA
## Temperature=30˚C, Age=15 days, Oviposition_positive=Y       4
## Temperature=32˚C, Age=3 days , Oviposition_positive=N      NA
## Temperature=32˚C, Age=3 days , Oviposition_positive=Y      10
## Temperature=32˚C, Age=5 days , Oviposition_positive=N      NA
## Temperature=32˚C, Age=5 days , Oviposition_positive=Y      10
## Temperature=32˚C, Age=10 days, Oviposition_positive=N      NA
## Temperature=32˚C, Age=10 days, Oviposition_positive=Y       5
## Temperature=32˚C, Age=15 days, Oviposition_positive=N      NA
## Temperature=32˚C, Age=15 days, Oviposition_positive=Y       4
```

```
sink("Oviposition_survival/firstBM_oviposvsneg_survival_mediansurvivaltime_stopminusstart.txt")
print(s1,print.rmean = TRUE)
sink()

s1data <- print(s1,print.rmean = TRUE)
```

```
## Call: survfit(formula = Surv(stopminusstart, Censor) ~ Temperature +
##     Age + Oviposition_positive, data = Fecundity_data_firstBM)
##
##                                                          n events rmean*
## Temperature=27˚C, Age=3 days , Oviposition_positive=N  15     15  12.93
## Temperature=27˚C, Age=3 days , Oviposition_positive=Y  63     63  12.67
## Temperature=27˚C, Age=5 days , Oviposition_positive=N  16     16  16.56
## Temperature=27˚C, Age=5 days , Oviposition_positive=Y  58     58  11.76
## Temperature=27˚C, Age=10 days, Oviposition_positive=N  14     14   9.21
## Temperature=27˚C, Age=10 days, Oviposition_positive=Y  58     58   9.22
## Temperature=27˚C, Age=15 days, Oviposition_positive=N  13     13   7.77
## Temperature=27˚C, Age=15 days, Oviposition_positive=Y  55     55   6.58
## Temperature=30˚C, Age=3 days , Oviposition_positive=N  23     23  11.78
## Temperature=30˚C, Age=3 days , Oviposition_positive=Y 103    103   7.94
## Temperature=30˚C, Age=5 days , Oviposition_positive=N  19     19  12.16
## Temperature=30˚C, Age=5 days , Oviposition_positive=Y  83     83  10.07
## Temperature=30˚C, Age=10 days, Oviposition_positive=N  15     15   8.73
## Temperature=30˚C, Age=10 days, Oviposition_positive=Y  60     60   6.60
## Temperature=30˚C, Age=15 days, Oviposition_positive=N   9      9   6.11
## Temperature=30˚C, Age=15 days, Oviposition_positive=Y  42     42   4.57
## Temperature=32˚C, Age=3 days , Oviposition_positive=N  10     10   7.40
## Temperature=32˚C, Age=3 days , Oviposition_positive=Y  45     45   8.73
## Temperature=32˚C, Age=5 days , Oviposition_positive=N   9      9  10.00
## Temperature=32˚C, Age=5 days , Oviposition_positive=Y  37     37   8.73
## Temperature=32˚C, Age=10 days, Oviposition_positive=N   8      8   9.62
## Temperature=32˚C, Age=10 days, Oviposition_positive=Y  44     44   5.25
## Temperature=32˚C, Age=15 days, Oviposition_positive=N   9      9   6.33
## Temperature=32˚C, Age=15 days, Oviposition_positive=Y  34     34   4.06
##                                                        se(rmean) median 0.95LCL
## Temperature=27˚C, Age=3 days , Oviposition_positive=N     1.849   11.0       9
## Temperature=27˚C, Age=3 days , Oviposition_positive=Y     1.017   12.0       9
## Temperature=27˚C, Age=5 days , Oviposition_positive=N     1.691   18.5      12
## Temperature=27˚C, Age=5 days , Oviposition_positive=Y     1.004    9.0       7
## Temperature=27˚C, Age=10 days, Oviposition_positive=N     1.070    9.0       6
## Temperature=27˚C, Age=10 days, Oviposition_positive=Y     0.715    9.0       6
## Temperature=27˚C, Age=15 days, Oviposition_positive=N     1.152    7.0       4
## Temperature=27˚C, Age=15 days, Oviposition_positive=Y     0.829    3.0       3
## Temperature=30˚C, Age=3 days , Oviposition_positive=N     0.895   12.0       9
## Temperature=30˚C, Age=3 days , Oviposition_positive=Y     0.539    5.0       4
## Temperature=30˚C, Age=5 days , Oviposition_positive=N     1.021   12.0       9
## Temperature=30˚C, Age=5 days , Oviposition_positive=Y     0.690   10.0       7
## Temperature=30˚C, Age=10 days, Oviposition_positive=N     1.113    8.0       6
## Temperature=30˚C, Age=10 days, Oviposition_positive=Y     0.616    4.0       3
## Temperature=30˚C, Age=15 days, Oviposition_positive=N     0.949    5.0       4
## Temperature=30˚C, Age=15 days, Oviposition_positive=Y     0.360    3.0       3
## Temperature=32˚C, Age=3 days , Oviposition_positive=N     0.777    7.0       5
## Temperature=32˚C, Age=3 days , Oviposition_positive=Y     0.677    9.0       7
## Temperature=32˚C, Age=5 days , Oviposition_positive=N     1.155   10.0       7
## Temperature=32˚C, Age=5 days , Oviposition_positive=Y     0.594    8.0       8
## Temperature=32˚C, Age=10 days, Oviposition_positive=N     1.117    9.5       7
## Temperature=32˚C, Age=10 days, Oviposition_positive=Y     0.437    4.0       3
## Temperature=32˚C, Age=15 days, Oviposition_positive=N     0.831    7.0       4
## Temperature=32˚C, Age=15 days, Oviposition_positive=Y     0.314    3.0       3
##                                                        0.95UCL
## Temperature=27˚C, Age=3 days , Oviposition_positive=N       22
## Temperature=27˚C, Age=3 days , Oviposition_positive=Y       16
```

```
## Temperature=27°C, Age=5 days , Oviposition_positive=N      24
## Temperature=27°C, Age=5 days , Oviposition_positive=Y      16
## Temperature=27°C, Age=10 days, Oviposition_positive=N      14
## Temperature=27°C, Age=10 days, Oviposition_positive=Y      11
## Temperature=27°C, Age=15 days, Oviposition_positive=N      NA
## Temperature=27°C, Age=15 days, Oviposition_positive=Y       4
## Temperature=30°C, Age=3 days , Oviposition_positive=N      15
## Temperature=30°C, Age=3 days , Oviposition_positive=Y       8
## Temperature=30°C, Age=5 days , Oviposition_positive=N      15
## Temperature=30°C, Age=5 days , Oviposition_positive=Y      12
## Temperature=30°C, Age=10 days, Oviposition_positive=N      14
## Temperature=30°C, Age=10 days, Oviposition_positive=Y       8
## Temperature=30°C, Age=15 days, Oviposition_positive=N      NA
## Temperature=30°C, Age=15 days, Oviposition_positive=Y       4
## Temperature=32°C, Age=3 days , Oviposition_positive=N      NA
## Temperature=32°C, Age=3 days , Oviposition_positive=Y      10
## Temperature=32°C, Age=5 days , Oviposition_positive=N      NA
## Temperature=32°C, Age=5 days , Oviposition_positive=Y      10
## Temperature=32°C, Age=10 days, Oviposition_positive=N      NA
## Temperature=32°C, Age=10 days, Oviposition_positive=Y       5
## Temperature=32°C, Age=15 days, Oviposition_positive=N      NA
## Temperature=32°C, Age=15 days, Oviposition_positive=Y       4
##     * restricted mean with upper limit =  30
```

```
sink("Oviposition_survival/firstBM_oviposvsneg_survival_mediansurvivaltime_maineffectovipos_stopminusstart.txt")
print(s2,print.rmean = TRUE)
sink()

sink("Oviposition_survival/firstBM_oviposvsneg_survival_mediansurvivaltime_maineffectoviposTEMP_stopminusstart.txt")
print(s3,print.rmean = TRUE)
sink()

sink("Oviposition_survival/firstBM_oviposvsneg_survival_mediansurvivaltime_maineffectoviposAGE_stopminusstart.txt")
print(s4,print.rmean = TRUE)
sink()
```

Analysis for hazard ratio and making forest plot

```
#HAZARD RATIO ANALYSIS:
#switch back to numbers:
Fecundity_data_firstBM$Temperature <- factor(Fecundity_data_firstBM$Temperature,
                                             labels = c("27","30","32"))
Fecundity_data_firstBM$Age <- factor(Fecundity_data_firstBM$Age,
                                     labels = c("3","5","10","15"))

Fecundity_data_firstBM_numeric <- Fecundity_data_firstBM

str(Fecundity_data_firstBM_numeric)
```

```
## 'data.frame':    842 obs. of  37 variables:
##  $ ID_overall               : num  1 2 3 4 5 6 7 8 9 10 ...
##  $ Temperature              : Factor w/ 3 levels "27","30","32": 3 3 3 3 3 3 3 3 3 3 ...
##  $ Age                      : Factor w/ 4 levels "3","5","10","15": 1 1 1 1 1 1 1 1 1 1 ...
##  $ ID_per_group             : num  1 2 3 4 5 6 7 8 9 10 ...
##  $ Trial_start_date         : POSIXct, format: "2024-02-13" "2024-02-13" ...
##  $ Trial_number             : num  1 1 1 1 1 1 1 1 1 1 ...
##  $ BM1_Date                 : POSIXct, format: "2024-02-13" "2024-02-13" ...
##  $ Age_of_BM                : num  3 3 3 3 3 3 3 3 3 3 ...
##  $ Bloodmeal_number         : Factor w/ 1 level "1": 1 1 1 1 1 1 1 1 1 1 ...
##  $ Oviposition_positive(y/n): chr  "Y" "Y" "Y" "Y" ...
##  $ Eggs_day3                : num  0 0 0 0 0 14 NA NA NA NA ...
##  $ Eggs_day4                : num  0 0 0 0 0 0 NA NA NA NA ...
##  $ Larvae_day4              : num  0 0 0 0 0 0 0 0 NA NA ...
##  $ Surv_to_eggs(y/n)        : Factor w/ 3 levels "N","NA","Y": 3 3 3 3 3 3 1 1 3 3 ...
##  $ Surv_to_larvae(y/n)      : Factor w/ 3 levels "N","NA","Y": 3 3 3 3 3 3 2 2 3 3 ...
##  $ Date_of_death            : POSIXct, format: "2024-03-01" "2024-03-07" ...
##  $ Censor                   : num  1 1 1 1 1 1 1 1 1 1 ...
##  $ Notes                    : chr  NA NA NA NA ...
##  $ Oviposition_positive     : Factor w/ 2 levels "N","Y": 2 2 2 2 2 2 2 2 1 1 ...
##  $ total_eggs               : num  0 0 0 0 0 14 NA NA NA NA ...
##  $ Percent_eggs_day3        : num  NaN NaN NaN NaN NaN 1 NA NA NA NA ...
##  $ Percent_eggs_day4        : num  NaN NaN NaN NaN NaN 0 NA NA NA NA ...
##  $ egg_binary               : num  0 0 0 0 0 1 NA NA NA NA ...
##  $ egg_binary_day3          : num  0 0 0 0 0 1 NA NA NA NA ...
##  $ egg_binary_day4          : num  0 0 0 0 0 0 NA NA NA NA ...
##  $ larvae_binary            : num  0 0 0 0 0 0 0 0 NA NA ...
##  $ Percent_eggshatchedtolarv: num  NaN NaN NaN NaN NaN 0 NA NA NA NA ...
##  $ Days_to_death_post_BM    : 'difftime' num  17 23 18 13 ...
##   ..- attr(*, "units")= chr "days"
##  $ Age_of_death             : 'difftime' num  20 26 21 16 ...
##   ..- attr(*, "units")= chr "days"
##  $ Age_of_BM_days           : 'difftime' num  3 3 3 3 ...
##   ..- attr(*, "units")= chr "days"
##  $ Date_of_eclosion         : POSIXct, format: "2024-02-10" "2024-02-10" ...
##  $ days_alive_post_BM       : num  17 23 18 13 15 18 3 3 12 11 ...
##  $ days_alive_post_eclosion : num  20 26 21 16 18 21 6 6 15 14 ...
##  $ group                    : chr  "3_32" "3_32" "3_32" "3_32" ...
##  $ start_time               : num  4 4 4 4 4 4 4 4 4 4 ...
##  $ stop_time                : num  21 27 22 17 19 22 7 7 16 15 ...
##  $ stopminusstart           : num  17 23 18 13 15 18 3 3 12 11 ...
```

```
#Compare hazard ratios:
#treat variables as numeric so hazard shows with every increase in age(days) or temp (degrees)
Fecundity_data_firstBM_numeric$Temperature <- as.character(Fecundity_data_firstBM_numeric$Temperature)
str(Fecundity_data_firstBM_numeric$Temperature)
```

```
##  chr [1:842] "32" "32" "32" "32" "32" "32" "32" "32" "32" "32" "27" "27" ...
```

```
Fecundity_data_firstBM_numeric$Temperature <- as.numeric(Fecundity_data_firstBM_numeric$Temperature)
Fecundity_data_firstBM_numeric$Age <- as.character(Fecundity_data_firstBM_numeric$Age)
str(Fecundity_data_firstBM_numeric$Age)
```

```
##  chr [1:842] "3" "3" "3" "3" "3" "3" "3" "3" "3" "3" "3" "3" "3" "3" "3" ...
```

```
Fecundity_data_firstBM_numeric$Age <- as.numeric(Fecundity_data_firstBM_numeric$Age)



Fecundity_data_firstBM_numeric$Trial_number <- as.factor(Fecundity_data_firstBM_numeric$Trial_number)

Fecundity_data_firstBM_numeric$Trial_start_date_factor <- as.factor(Fecundity_data_firstBM_numeric$Trial_st
art_date)
str(Fecundity_data_firstBM_numeric$Trial_start_date_factor)
```

```
##  Factor w/ 32 levels "2024-02-13","2024-02-19",..: 1 1 1 1 1 1 1 1 1 1 ...
```

```
str(Fecundity_data_firstBM_numeric)
```

```
## 'data.frame':    842 obs. of  38 variables:
##  $ ID_overall                : num  1 2 3 4 5 6 7 8 9 10 ...
##  $ Temperature               : num  32 32 32 32 32 32 32 32 32 32 ...
##  $ Age                       : num  3 3 3 3 3 3 3 3 3 3 ...
##  $ ID_per_group              : num  1 2 3 4 5 6 7 8 9 10 ...
##  $ Trial_start_date          : POSIXct, format: "2024-02-13" "2024-02-13" ...
##  $ Trial_number              : Factor w/ 5 levels "1","2","3","4",..: 1 1 1 1 1 1 1 1 1 1 ...
##  $ BM1_Date                  : POSIXct, format: "2024-02-13" "2024-02-13" ...
##  $ Age_of_BM                 : num  3 3 3 3 3 3 3 3 3 3 ...
##  $ Bloodmeal_number          : Factor w/ 1 level "1": 1 1 1 1 1 1 1 1 1 1 ...
##  $ Oviposition_positive(y/n): chr  "Y" "Y" "Y" "Y" ...
##  $ Eggs_day3                 : num  0 0 0 0 0 14 NA NA NA NA ...
##  $ Eggs_day4                 : num  0 0 0 0 0 0 NA NA NA NA ...
##  $ Larvae_day4               : num  0 0 0 0 0 0 0 0 NA NA ...
##  $ Surv_to_eggs(y/n)         : Factor w/ 3 levels "N","NA","Y": 3 3 3 3 3 3 1 1 3 3 ...
##  $ Surv_to_larvae(y/n)       : Factor w/ 3 levels "N","NA","Y": 3 3 3 3 3 3 2 2 3 3 ...
##  $ Date_of_death             : POSIXct, format: "2024-03-01" "2024-03-07" ...
##  $ Censor                    : num  1 1 1 1 1 1 1 1 1 1 ...
##  $ Notes                     : chr  NA NA NA NA ...
##  $ Oviposition_positive      : Factor w/ 2 levels "N","Y": 2 2 2 2 2 2 2 2 1 1 ...
##  $ total_eggs                : num  0 0 0 0 0 14 NA NA NA NA ...
##  $ Percent_eggs_day3         : num  NaN NaN NaN NaN NaN 1 NA NA NA NA ...
##  $ Percent_eggs_day4         : num  NaN NaN NaN NaN NaN 0 NA NA NA NA ...
##  $ egg_binary                : num  0 0 0 0 0 1 NA NA NA NA ...
##  $ egg_binary_day3           : num  0 0 0 0 0 1 NA NA NA NA ...
##  $ egg_binary_day4           : num  0 0 0 0 0 0 NA NA NA NA ...
##  $ larvae_binary             : num  0 0 0 0 0 0 0 0 NA NA ...
##  $ Percent_eggshatchedtolarv: num  NaN NaN NaN NaN NaN 0 NA NA NA NA ...
##  $ Days_to_death_post_BM    : 'difftime' num  17 23 18 13 ...
##   ..- attr(*, "units")= chr "days"
##  $ Age_of_death              : 'difftime' num  20 26 21 16 ...
##   ..- attr(*, "units")= chr "days"
##  $ Age_of_BM_days            : 'difftime' num  3 3 3 3 ...
##   ..- attr(*, "units")= chr "days"
##  $ Date_of_eclosion          : POSIXct, format: "2024-02-10" "2024-02-10" ...
##  $ days_alive_post_BM        : num  17 23 18 13 15 18 3 3 12 11 ...
##  $ days_alive_post_eclosion : num  20 26 21 16 18 21 6 6 15 14 ...
##  $ group                     : chr  "3_32" "3_32" "3_32" "3_32" ...
##  $ start_time                : num  4 4 4 4 4 4 4 4 4 4 ...
##  $ stop_time                 : num  21 27 22 17 19 22 7 7 16 15 ...
##  $ stopminusstart            : num  17 23 18 13 15 18 3 3 12 11 ...
##  $ Trial_start_date_factor  : Factor w/ 32 levels "2024-02-13","2024-02-19",..: 1 1 1 1 1 1 1 1 1 1 ...
```

```
Fecundity_data_firstBM_numeric$Trial_start_date_number <- as.numeric(Fecundity_data_firstBM_numeric$Trial_s
tart_date_factor)
str(Fecundity_data_firstBM_numeric)
```

```
## 'data.frame':    842 obs. of  39 variables:
##  $ ID_overall                : num  1 2 3 4 5 6 7 8 9 10 ...
##  $ Temperature               : num  32 32 32 32 32 32 32 32 32 32 ...
##  $ Age                       : num  3 3 3 3 3 3 3 3 3 3 ...
##  $ ID_per_group              : num  1 2 3 4 5 6 7 8 9 10 ...
##  $ Trial_start_date          : POSIXct, format: "2024-02-13" "2024-02-13" ...
##  $ Trial_number              : Factor w/ 5 levels "1","2","3","4",..: 1 1 1 1 1 1 1 1 1 1 ...
##  $ BM1_Date                  : POSIXct, format: "2024-02-13" "2024-02-13" ...
##  $ Age_of_BM                 : num  3 3 3 3 3 3 3 3 3 3 ...
##  $ Bloodmeal_number          : Factor w/ 1 level "1": 1 1 1 1 1 1 1 1 1 1 ...
##  $ Oviposition_positive(y/n): chr  "Y" "Y" "Y" "Y" ...
##  $ Eggs_day3                 : num  0 0 0 0 0 14 NA NA NA NA ...
##  $ Eggs_day4                 : num  0 0 0 0 0 0 NA NA NA NA ...
##  $ Larvae_day4               : num  0 0 0 0 0 0 0 0 NA NA ...
##  $ Surv_to_eggs(y/n)         : Factor w/ 3 levels "N","NA","Y": 3 3 3 3 3 3 1 1 3 3 ...
##  $ Surv_to_larvae(y/n)       : Factor w/ 3 levels "N","NA","Y": 3 3 3 3 3 3 2 2 3 3 ...
##  $ Date_of_death             : POSIXct, format: "2024-03-01" "2024-03-07" ...
##  $ Censor                    : num  1 1 1 1 1 1 1 1 1 1 ...
##  $ Notes                     : chr  NA NA NA NA ...
##  $ Oviposition_positive      : Factor w/ 2 levels "N","Y": 2 2 2 2 2 2 2 2 1 1 ...
##  $ total_eggs                : num  0 0 0 0 0 14 NA NA NA NA ...
##  $ Percent_eggs_day3         : num  NaN NaN NaN NaN NaN 1 NA NA NA NA ...
##  $ Percent_eggs_day4         : num  NaN NaN NaN NaN NaN 0 NA NA NA NA ...
##  $ egg_binary                : num  0 0 0 0 0 1 NA NA NA NA ...
##  $ egg_binary_day3           : num  0 0 0 0 0 1 NA NA NA NA ...
##  $ egg_binary_day4           : num  0 0 0 0 0 0 NA NA NA NA ...
##  $ larvae_binary             : num  0 0 0 0 0 0 0 0 NA NA ...
##  $ Percent_eggshatchedtolarv: num  NaN NaN NaN NaN NaN 0 NA NA NA NA ...
##  $ Days_to_death_post_BM     : 'difftime' num  17 23 18 13 ...
##   ..- attr(*, "units")= chr "days"
##  $ Age_of_death              : 'difftime' num  20 26 21 16 ...
##   ..- attr(*, "units")= chr "days"
##  $ Age_of_BM_days            : 'difftime' num  3 3 3 3 ...
##   ..- attr(*, "units")= chr "days"
##  $ Date_of_eclosion          : POSIXct, format: "2024-02-10" "2024-02-10" ...
##  $ days_alive_post_BM        : num  17 23 18 13 15 18 3 3 12 11 ...
##  $ days_alive_post_eclosion : num  20 26 21 16 18 21 6 6 15 14 ...
##  $ group                     : chr  "3_32" "3_32" "3_32" "3_32" ...
##  $ start_time                : num  4 4 4 4 4 4 4 4 4 4 ...
##  $ stop_time                 : num  21 27 22 17 19 22 7 7 16 15 ...
##  $ stopminusstart            : num  17 23 18 13 15 18 3 3 12 11 ...
##  $ Trial_start_date_factor   : Factor w/ 32 levels "2024-02-13","2024-02-19",..: 1 1 1 1 1 1 1 1 1 1 ...
##  $ Trial_start_date_number   : num  1 1 1 1 1 1 1 1 1 1 ...
```

```
Fecundity_data_firstBM_numeric$Tempscaled <- scale(Fecundity_data_firstBM_numeric$Temperature, center=TRUE,
scale=TRUE)
Fecundity_data_firstBM_numeric$Agescaled <- scale(Fecundity_data_firstBM_numeric$Age, center=TRUE, scale=TR
UE)

#check proportional hazards model
mv_fit <- coxph(Surv(stop_time, Censor) ~ Temperature*Age, data = Fecundity_data_firstBM_numeric)
mv_fit <- coxph(Surv(stop_time, Censor) ~ Tempscaled*Agescaled, data = Fecundity_data_firstBM_numeric)

cz <- cox.zph(mv_fit)
print(cz) # significant p-value indicates that the proportional hazards assumption is violated
```

```
##                          chisq df       p
## Tempscaled            11.5572  1 0.00067
## Agescaled            117.4058  1 < 2e-16
## Tempscaled:Agescaled   0.0314  1 0.85927
## GLOBAL               136.5232  3 < 2e-16
```

```
plot(cz) #violated for age, temp*age, global - non-proportional
```

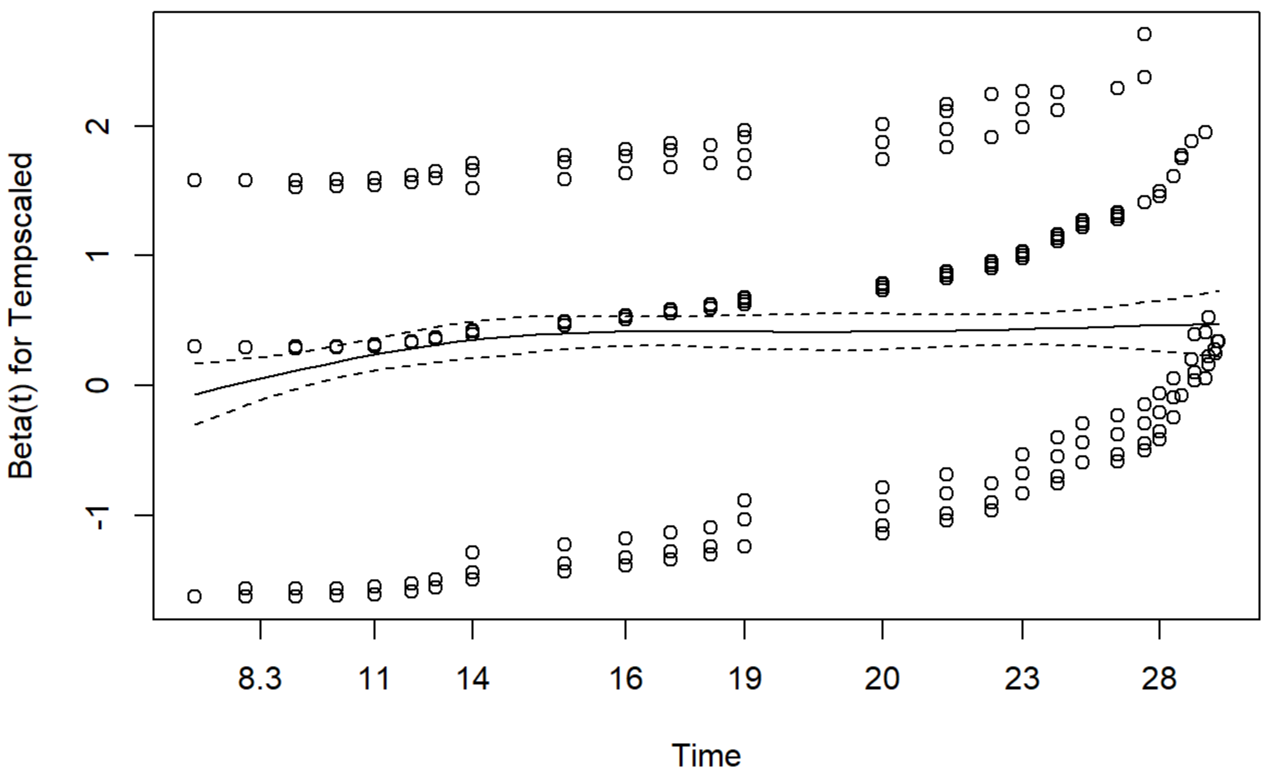
Beta(t) for Tempscaled
2
1
0
-1
8.3
11
14
16
19
20
23
28
Time

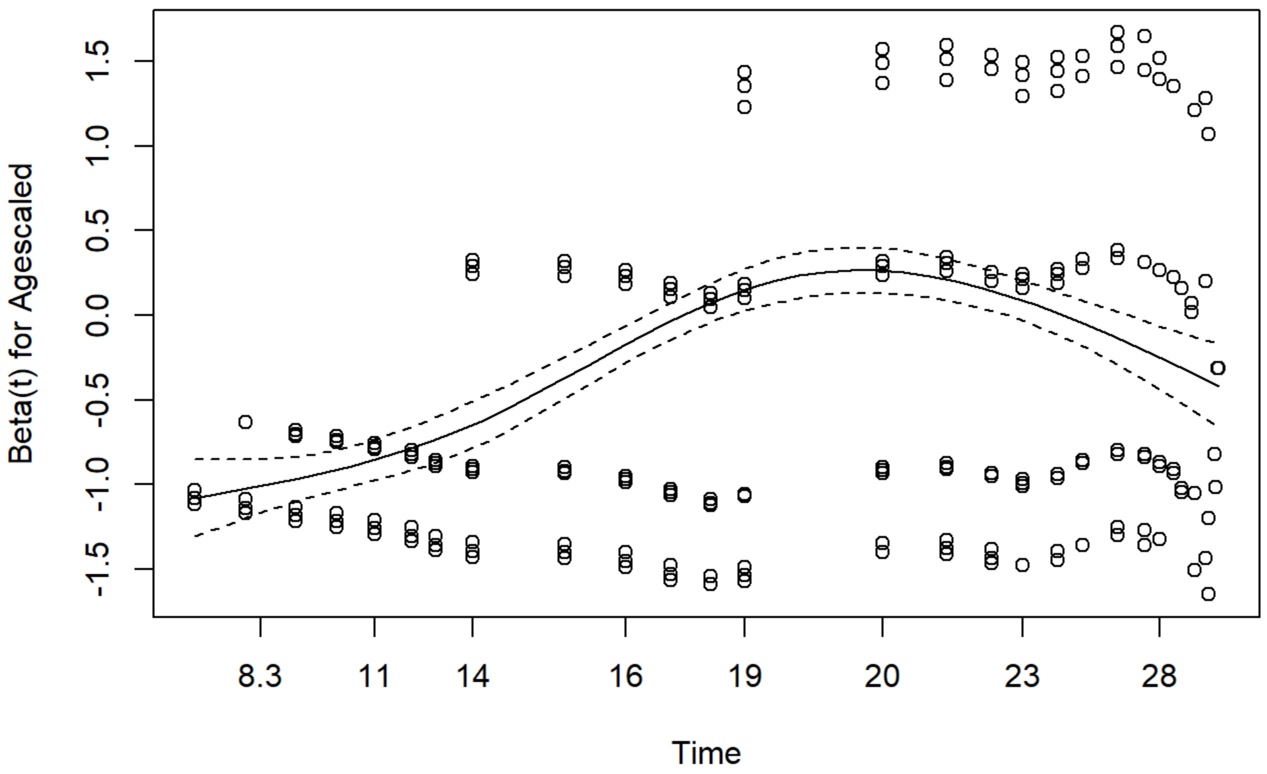
Beta(t) for Agescaled
1.5
1.0
0.5
0.0
-0.5
-1.0
-1.5
8.3
11
14
16
19
20
23
28
Time

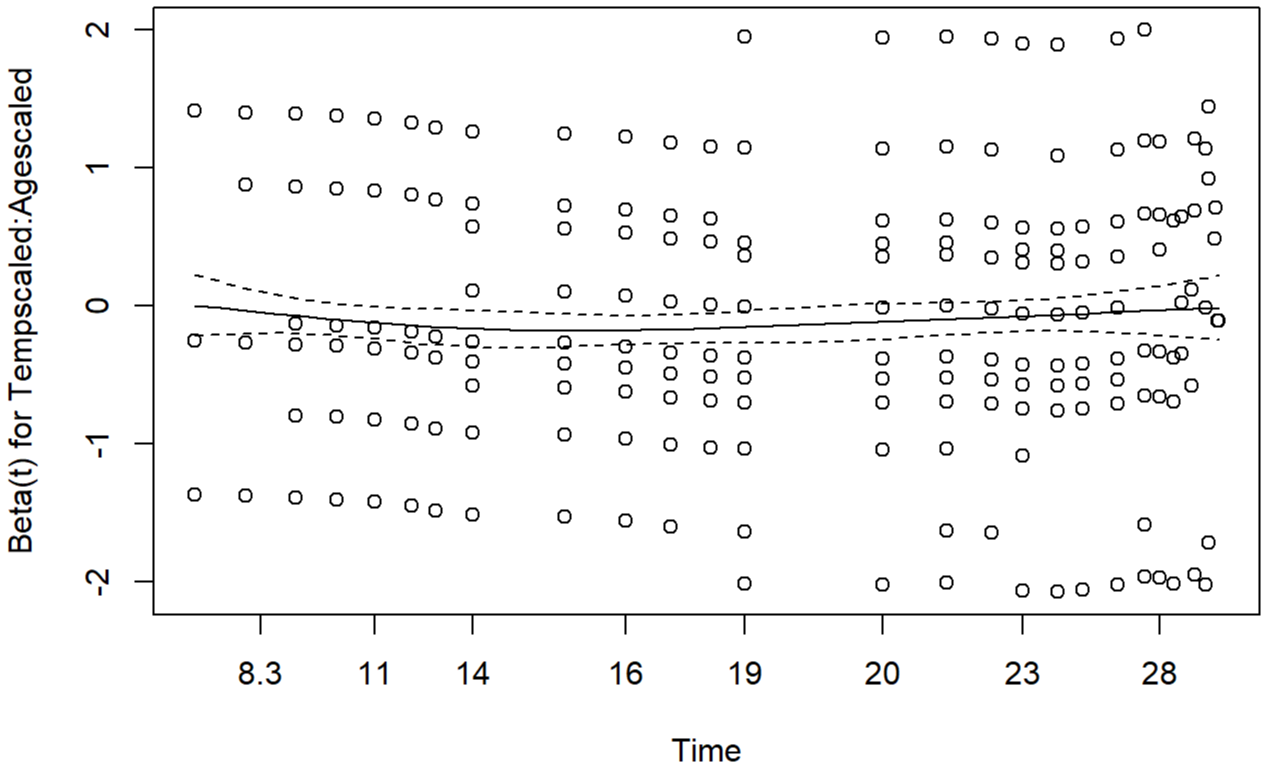


```
str(Fecundity_data_firstBM_numeric)
```

```
## 'data.frame':    842 obs. of  41 variables:
##  $ ID_overall                 : num  1 2 3 4 5 6 7 8 9 10 ...
##  $ Temperature                : num  32 32 32 32 32 32 32 32 32 32 ...
##  $ Age                        : num  3 3 3 3 3 3 3 3 3 3 ...
##  $ ID_per_group               : num  1 2 3 4 5 6 7 8 9 10 ...
##  $ Trial_start_date           : POSIXct, format: "2024-02-13" "2024-02-13" ...
##  $ Trial_number               : Factor w/ 5 levels "1","2","3","4",..: 1 1 1 1 1 1 1 1 1 1 ...
##  $ BM1_Date                   : POSIXct, format: "2024-02-13" "2024-02-13" ...
##  $ Age_of_BM                  : num  3 3 3 3 3 3 3 3 3 3 ...
##  $ Bloodmeal_number           : Factor w/ 1 level "1": 1 1 1 1 1 1 1 1 1 1 ...
##  $ Oviposition_positive(y/n): chr  "Y" "Y" "Y" "Y" ...
##  $ Eggs_day3                  : num  0 0 0 0 0 14 NA NA NA NA ...
##  $ Eggs_day4                  : num  0 0 0 0 0 0 NA NA NA NA ...
##  $ Larvae_day4                : num  0 0 0 0 0 0 0 0 NA NA ...
##  $ Surv_to_eggs(y/n)          : Factor w/ 3 levels "N","NA","Y": 3 3 3 3 3 3 1 1 3 3 ...
##  $ Surv_to_larvae(y/n)        : Factor w/ 3 levels "N","NA","Y": 3 3 3 3 3 3 2 2 3 3 ...
##  $ Date_of_death              : POSIXct, format: "2024-03-01" "2024-03-07" ...
##  $ Censor                     : num  1 1 1 1 1 1 1 1 1 1 ...
##  $ Notes                      : chr  NA NA NA NA ...
##  $ Oviposition_positive       : Factor w/ 2 levels "N","Y": 2 2 2 2 2 2 2 2 1 1 ...
##  $ total_eggs                 : num  0 0 0 0 0 14 NA NA NA NA ...
##  $ Percent_eggs_day3          : num  NaN NaN NaN NaN NaN 1 NA NA NA NA ...
##  $ Percent_eggs_day4          : num  NaN NaN NaN NaN NaN 0 NA NA NA NA ...
##  $ egg_binary                 : num  0 0 0 0 0 1 NA NA NA NA ...
##  $ egg_binary_day3            : num  0 0 0 0 0 1 NA NA NA NA ...
##  $ egg_binary_day4            : num  0 0 0 0 0 0 NA NA NA NA ...
##  $ larvae_binary              : num  0 0 0 0 0 0 0 0 NA NA ...
##  $ Percent_eggshatchedtolarv: num  NaN NaN NaN NaN NaN 0 NA NA NA NA ...
##  $ Days_to_death_post_BM      : 'difftime' num  17 23 18 13 ...
##   ..- attr(*, "units")= chr "days"
##  $ Age_of_death               : 'difftime' num  20 26 21 16 ...
##   ..- attr(*, "units")= chr "days"
##  $ Age_of_BM_days             : 'difftime' num  3 3 3 3 ...
##   ..- attr(*, "units")= chr "days"
##  $ Date_of_eclosion           : POSIXct, format: "2024-02-10" "2024-02-10" ...
##  $ days_alive_post_BM         : num  17 23 18 13 15 18 3 3 12 11 ...
##  $ days_alive_post_eclosion : num  20 26 21 16 18 21 6 6 15 14 ...
##  $ group                      : chr  "3_32" "3_32" "3_32" "3_32" ...
##  $ start_time                 : num  4 4 4 4 4 4 4 4 4 4 ...
##  $ stop_time                  : num  21 27 22 17 19 22 7 7 16 15 ...
##  $ stopminusstart             : num  17 23 18 13 15 18 3 3 12 11 ...
##  $ Trial_start_date_factor  : Factor w/ 32 levels "2024-02-13","2024-02-19",..: 1 1 1 1 1 1 1 1 1 1 ...
##  $ Trial_start_date_number  : num  1 1 1 1 1 1 1 1 1 1 ...
##  $ Tempscaled                 : num [1:842, 1] 1.33 1.33 1.33 1.33 1.33 ...
##   ..- attr(*, "scaled:center")= num 29.4
##   ..- attr(*, "scaled:scale")= num 1.93
##  $ Agescaled                  : num [1:842, 1] -0.999 -0.999 -0.999 -0.999 -0.999 ...
##   ..- attr(*, "scaled:center")= num 7.49
##   ..- attr(*, "scaled:scale")= num 4.49
```

```
mv_fit <- coxph(Surv(start_time,stop_time, Censor) ~ Oviposition_positive*Temperature*Age+cluster(Trial_sta
rt_date_factor), robust=TRUE,
                data = Fecundity_data_firstBM_numeric,method="breslow")

#non-proportional hazards:
#coxph regression with weighted estimation, accounting for experimental block
library(condsurv)
library(coxphw)
fit1 <- coxphw(Surv(start_time,stop_time, Censor) ~ Oviposition_positive*Temperature*Age +
                 frailty(Trial_start_date_factor,distribution = "gaussian"),
               data = Fecundity_data_firstBM_numeric,
               template = "AHR")

summary(fit1)
```

```
## coxphw(formula = Surv(start_time, stop_time, Censor) ~ Oviposition_positive *
##     Temperature * Age + frailty(Trial_start_date_factor, distribution = "gaussian"),
##     data = Fecundity_data_firstBM_numeric, template = "AHR")
##
## Model fitted by weighted estimation (AHR template)
##
##                                                                      coef
## Oviposition_positiveY                                          2.78406328
## Temperature                                                    0.25413204
## Age                                                            0.72312345
## frailty(Trial_start_date_factor, distribution = "gaussian")  0.03519474
## Oviposition_positiveY:Temperature                             -0.08014274
## Oviposition_positiveY:Age                                     -0.36877869
## Temperature:Age                                               -0.02431985
## Oviposition_positiveY:Temperature:Age                          0.01304136
##                                                                  se(coef)
## Oviposition_positiveY                                         3.184707724
## Temperature                                                   0.096648708
## Age                                                           0.296372944
## frailty(Trial_start_date_factor, distribution = "gaussian") 0.005372499
## Oviposition_positiveY:Temperature                             0.105807429
## Oviposition_positiveY:Age                                     0.325164106
## Temperature:Age                                               0.009892392
## Oviposition_positiveY:Temperature:Age                         0.010844832
##                                                                exp(coef)
## Oviposition_positiveY                                         16.1846502
## Temperature                                                    1.2893420
## Age                                                            2.0608602
## frailty(Trial_start_date_factor, distribution = "gaussian")  1.0358214
## Oviposition_positiveY:Temperature                              0.9229846
## Oviposition_positiveY:Age                                      0.6915784
## Temperature:Age                                                0.9759735
## Oviposition_positiveY:Temperature:Age                          1.0131268
##                                                               lower 0.95
## Oviposition_positiveY                                         0.03149743
## Temperature                                                   1.06684325
## Age                                                           1.15285693
## frailty(Trial_start_date_factor, distribution = "gaussian") 1.02497153
## Oviposition_positiveY:Temperature                             0.75012044
## Oviposition_positiveY:Age                                     0.36564637
## Temperature:Age                                               0.95723287
## Oviposition_positiveY:Temperature:Age                         0.99181953
##                                                                upper 0.95
## Oviposition_positiveY                                         8316.324892
## Temperature                                                      1.558245
## Age                                                              3.684017
## frailty(Trial_start_date_factor, distribution = "gaussian")    1.046786
## Oviposition_positiveY:Temperature                                1.135685
## Oviposition_positiveY:Age                                        1.308042
## Temperature:Age                                                  0.995081
## Oviposition_positiveY:Temperature:Age                            1.034892
##                                                                        z
## Oviposition_positiveY                                          0.8741974
## Temperature                                                    2.6294407
## Age                                                            2.4399105
## frailty(Trial_start_date_factor, distribution = "gaussian")  6.5509070
```

```
## Oviposition_positiveY:Temperature                           -0.7574397
## Oviposition_positiveY:Age                                   -1.1341310
## Temperature:Age                                             -2.4584396
## Oviposition_positiveY:Temperature:Age                        1.2025418
##                                                                        p
## Oviposition_positiveY                                       3.820108e-01
## Temperature                                                 8.552546e-03
## Age                                                         1.469090e-02
## frailty(Trial_start_date_factor, distribution = "gaussian") 5.718870e-11
## Oviposition_positiveY:Temperature                           4.487865e-01
## Oviposition_positiveY:Age                                   2.567396e-01
## Temperature:Age                                             1.395422e-02
## Oviposition_positiveY:Temperature:Age                       2.291537e-01
##
## Wald Chi-square = 118.947 on 8  df  p = 0  n = 842
##
## Covariance-Matrix:
##                                                             Oviposition_positiveY
## Oviposition_positiveY                                               10.1423632891
## Temperature                                                          0.2770624293
## Age                                                                  0.7648253499
## frailty(Trial_start_date_factor, distribution = "gaussian")         -0.0005519233
## Oviposition_positiveY:Temperature                                   -0.3365284922
## Oviposition_positiveY:Age                                           -0.9345171641
## Temperature:Age                                                     -0.0254559140
## Oviposition_positiveY:Temperature:Age                                0.0310543178
##                                                               Temperature
## Oviposition_positiveY                                        0.2770624293
## Temperature                                                  0.0093409728
## Age                                                          0.0258582021
## frailty(Trial_start_date_factor, distribution = "gaussian")  0.0000247557
## Oviposition_positiveY:Temperature                           -0.0092164391
## Oviposition_positiveY:Age                                   -0.0255493730
## Temperature:Age                                             -0.0008637139
## Oviposition_positiveY:Temperature:Age                        0.0008516393
##                                                                     Age
## Oviposition_positiveY                                        0.764825350
## Temperature                                                  0.025858202
## Age                                                          0.087836922
## frailty(Trial_start_date_factor, distribution = "gaussian")  0.000200051
## Oviposition_positiveY:Temperature                           -0.025400486
## Oviposition_positiveY:Age                                   -0.085930267
## Temperature:Age                                             -0.002926655
## Oviposition_positiveY:Temperature:Age                        0.002861394
##                                                             frailty(Trial_start_date_factor, distributio
n = "gaussian")
## Oviposition_positiveY
-5.519233e-04
## Temperature
2.475570e-05
## Age
2.000510e-04
## frailty(Trial_start_date_factor, distribution = "gaussian")
2.886374e-05
## Oviposition_positiveY:Temperature
2.101966e-05
```

```
## Oviposition_positiveY:Age
5.454101e-05
## Temperature:Age
-6.006206e-06
## Oviposition_positiveY:Temperature:Age
-2.081204e-06
##                                                               Oviposition_positiveY:Temperature
## Oviposition_positiveY                                                             -3.365285e-01
## Temperature                                                                       -9.216439e-03
## Age                                                                               -2.540049e-02
## frailty(Trial_start_date_factor, distribution = "gaussian")                        2.101966e-05
## Oviposition_positiveY:Temperature                                                  1.119521e-02
## Oviposition_positiveY:Age                                                          3.104526e-02
## Temperature:Age                                                                    8.477527e-04
## Oviposition_positiveY:Temperature:Age                                             -1.034595e-03
##                                                               Oviposition_positiveY:Age
## Oviposition_positiveY                                                     -9.345172e-01
## Temperature                                                               -2.554937e-02
## Age                                                                       -8.593027e-02
## frailty(Trial_start_date_factor, distribution = "gaussian")                5.454101e-05
## Oviposition_positiveY:Temperature                                          3.104526e-02
## Oviposition_positiveY:Age                                                  1.057317e-01
## Temperature:Age                                                            2.866061e-03
## Oviposition_positiveY:Temperature:Age                                     -3.520425e-03
##                                                               Temperature:Age
## Oviposition_positiveY                                           -2.545591e-02
## Temperature                                                     -8.637139e-04
## Age                                                             -2.926655e-03
## frailty(Trial_start_date_factor, distribution = "gaussian")     -6.006206e-06
## Oviposition_positiveY:Temperature                                8.477527e-04
## Oviposition_positiveY:Age                                        2.866061e-03
## Temperature:Age                                                  9.785942e-05
## Oviposition_positiveY:Temperature:Age                           -9.574761e-05
##                                                               Oviposition_positiveY:Temperature:Age
## Oviposition_positiveY                                                                  3.105432e-02
## Temperature                                                                            8.516393e-04
## Age                                                                                    2.861394e-03
## frailty(Trial_start_date_factor, distribution = "gaussian")                           -2.081204e-06
## Oviposition_positiveY:Temperature                                                     -1.034595e-03
## Oviposition_positiveY:Age                                                             -3.520425e-03
## Temperature:Age                                                                       -9.574761e-05
## Oviposition_positiveY:Temperature:Age                                                  1.176104e-04
##
## Generalized concordance probability:
##                                                               concordance prob.
## Oviposition_positiveY                                                    0.9418
## Temperature                                                              0.5632
## Age                                                                      0.6733
## frailty(Trial_start_date_factor, distribution = "gaussian")              0.5088
## Oviposition_positiveY:Temperature                                        0.4800
## Oviposition_positiveY:Age                                                0.4088
## Temperature:Age                                                          0.4939
## Oviposition_positiveY:Temperature:Age                                    0.5033
##                                                               lower 0.95
## Oviposition_positiveY                                             0.0305
## Temperature                                                       0.5162
```

```
## Age                                                             0.5355
## frailty(Trial_start_date_factor, distribution = "gaussian")     0.5062
## Oviposition_positiveY:Temperature                               0.4286
## Oviposition_positiveY:Age                                       0.2677
## Temperature:Age                                                 0.4891
## Oviposition_positiveY:Temperature:Age                           0.4979
##                                                             upper 0.95
## Oviposition_positiveY                                           0.9999
## Temperature                                                     0.6091
## Age                                                             0.7865
## frailty(Trial_start_date_factor, distribution = "gaussian")     0.5114
## Oviposition_positiveY:Temperature                               0.5318
## Oviposition_positiveY:Age                                       0.5667
## Temperature:Age                                                 0.4988
## Oviposition_positiveY:Temperature:Age                           0.5086
```

```
plot(fit1$dfbeta.resid) # residuals are bad
```

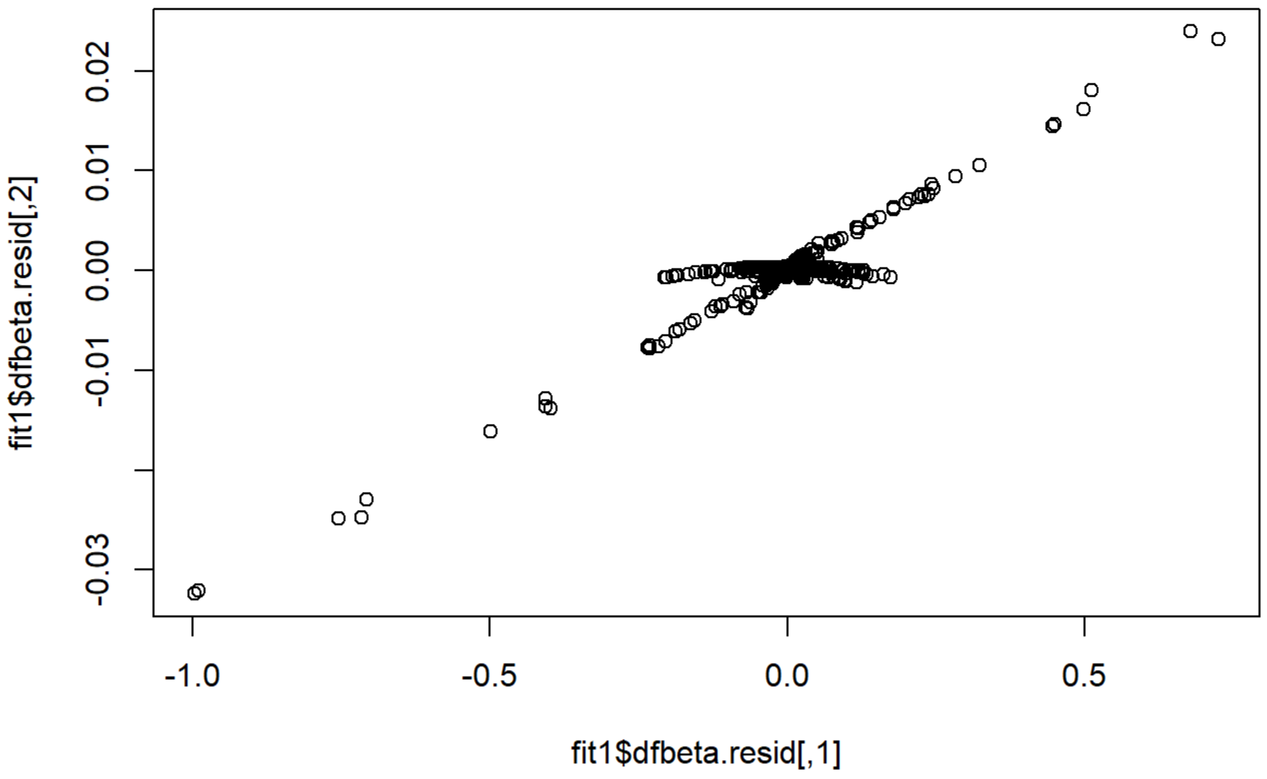


```
#residuals are bad - try centering and scaling temperature and age numbers

#scale and center independent numeric variables:
fit1 <- coxphw(Surv(start_time,stop_time, Censor) ~ Oviposition_positive*Tempscaled*Agescaled +
                 frailty(Trial_start_date_factor,distribution = "gaussian"),
               data =Fecundity_data_firstBM_numeric,
               template = "AHR")
summary(fit1)
```

```
## coxphw(formula = Surv(start_time, stop_time, Censor) ~ Oviposition_positive *
##     Tempscaled * Agescaled + frailty(Trial_start_date_factor,
##     distribution = "gaussian"), data = Fecundity_data_firstBM_numeric,
##     template = "AHR")
##
## Model fitted by weighted estimation (AHR template)
##
##                                                                coef
## Oviposition_positiveY                                    0.53794958
## Tempscaled                                               0.13891638
## Agescaled                                                0.03374258
## frailty(Trial_start_date_factor, distribution = "gaussian")  0.03519474
## Oviposition_positiveY:Tempscaled                         0.03386528
## Oviposition_positiveY:Agescaled                          0.06726305
## Tempscaled:Agescaled                                    -0.21099691
## Oviposition_positiveY:Tempscaled:Agescaled               0.11314574
##                                                             se(coef)
## Oviposition_positiveY                                   0.084783356
## Tempscaled                                              0.083972147
## Agescaled                                               0.082010914
## frailty(Trial_start_date_factor, distribution = "gaussian") 0.005372499
## Oviposition_positiveY:Tempscaled                        0.092471481
## Oviposition_positiveY:Agescaled                         0.088227545
## Tempscaled:Agescaled                                    0.085825541
## Oviposition_positiveY:Tempscaled:Agescaled              0.094088824
##                                                         exp(coef)
## Oviposition_positiveY                                   1.7124919
## Tempscaled                                              1.1490280
## Agescaled                                               1.0343183
## frailty(Trial_start_date_factor, distribution = "gaussian") 1.0358214
## Oviposition_positiveY:Tempscaled                        1.0344452
## Oviposition_positiveY:Agescaled                         1.0695768
## Tempscaled:Agescaled                                    0.8097766
## Oviposition_positiveY:Tempscaled:Agescaled              1.1197951
##                                                         lower 0.95
## Oviposition_positiveY                                    1.4503099
## Tempscaled                                               0.9746606
## Agescaled                                                0.8807373
## frailty(Trial_start_date_factor, distribution = "gaussian")  1.0249715
## Oviposition_positiveY:Tempscaled                         0.8629699
## Oviposition_positiveY:Agescaled                          0.8997307
## Tempscaled:Agescaled                                     0.6844006
## Oviposition_positiveY:Tempscaled:Agescaled               0.9312151
##                                                         upper 0.95
## Oviposition_positiveY                                    2.0220703
## Tempscaled                                               1.3545899
## Agescaled                                                1.2146804
## frailty(Trial_start_date_factor, distribution = "gaussian")  1.0467861
## Oviposition_positiveY:Tempscaled                         1.2399934
## Oviposition_positiveY:Agescaled                          1.2714855
## Tempscaled:Agescaled                                     0.9581203
## Oviposition_positiveY:Tempscaled:Agescaled               1.3465644
##                                                                 z
## Oviposition_positiveY                                    6.3449904
## Tempscaled                                               1.6543150
## Agescaled                                                0.4114401
```

```
## frailty(Trial_start_date_factor, distribution = "gaussian")  6.5509070
## Oviposition_positiveY:Tempscaled                              0.3662240
## Oviposition_positiveY:Agescaled                               0.7623816
## Tempscaled:Agescaled                                         -2.4584396
## Oviposition_positiveY:Tempscaled:Agescaled                    1.2025417
##                                                                      p
## Oviposition_positiveY                                        2.224393e-10
## Tempscaled                                                   9.806353e-02
## Agescaled                                                    6.807498e-01
## frailty(Trial_start_date_factor, distribution = "gaussian") 5.718870e-11
## Oviposition_positiveY:Tempscaled                             7.141979e-01
## Oviposition_positiveY:Agescaled                              4.458323e-01
## Tempscaled:Agescaled                                         1.395422e-02
## Oviposition_positiveY:Tempscaled:Agescaled                   2.291537e-01
##
## Wald Chi-square = 118.947 on 8  df  p = 0  n = 842
##
## Covariance-Matrix:
##                                                              Oviposition_positiveY
## Oviposition_positiveY                                                 0.0071882174
## Tempscaled                                                            0.0021321663
## Agescaled                                                             0.0005864351
## frailty(Trial_start_date_factor, distribution = "gaussian")          0.0000164067
## Oviposition_positiveY:Tempscaled                                     -0.0026438973
## Oviposition_positiveY:Agescaled                                      -0.0006022265
## Tempscaled:Agescaled                                                 -0.0012673321
## Oviposition_positiveY:Tempscaled:Agescaled                            0.0014217070
##                                                                Tempscaled
## Oviposition_positiveY                                         2.132166e-03
## Tempscaled                                                    7.051321e-03
## Agescaled                                                     7.833036e-04
## frailty(Trial_start_date_factor, distribution = "gaussian") -3.905833e-05
## Oviposition_positiveY:Tempscaled                             -6.928279e-03
## Oviposition_positiveY:Agescaled                              -1.085951e-03
## Tempscaled:Agescaled                                         -2.188875e-03
## Oviposition_positiveY:Tempscaled:Agescaled                    2.251577e-03
##                                                                 Agescaled
## Oviposition_positiveY                                         0.0005864351
## Tempscaled                                                    0.0007833036
## Agescaled                                                     0.0067257900
## frailty(Trial_start_date_factor, distribution = "gaussian")  0.0001047990
## Oviposition_positiveY:Tempscaled                             -0.0010897126
## Oviposition_positiveY:Agescaled                              -0.0060822723
## Tempscaled:Agescaled                                         -0.0018375328
## Oviposition_positiveY:Tempscaled:Agescaled                    0.0017158646
##                                                              frailty(Trial_start_date_factor, distributio
n = "gaussian")
## Oviposition_positiveY
1.640670e-05
## Tempscaled
-3.905833e-05
## Agescaled
1.047990e-04
## frailty(Trial_start_date_factor, distribution = "gaussian")
2.886374e-05
## Oviposition_positiveY:Tempscaled
```

```
1.048259e-05
## Oviposition_positiveY:Agescaled
-3.010744e-05
## Tempscaled:Agescaled
-5.210932e-05
## Oviposition_positiveY:Tempscaled:Agescaled
-1.805634e-05
##                                                               Oviposition_positiveY:Tempscaled
## Oviposition_positiveY                                                            -2.643897e-03
## Tempscaled                                                                       -6.928279e-03
## Agescaled                                                                        -1.089713e-03
## frailty(Trial_start_date_factor, distribution = "gaussian")                       1.048259e-05
## Oviposition_positiveY:Tempscaled                                                  8.550975e-03
## Oviposition_positiveY:Agescaled                                                   1.343141e-03
## Tempscaled:Agescaled                                                              2.186485e-03
## Oviposition_positiveY:Tempscaled:Agescaled                                       -2.573000e-03
##                                                               Oviposition_positiveY:Agescaled
## Oviposition_positiveY                                                          -6.022265e-04
## Tempscaled                                                                     -1.085951e-03
## Agescaled                                                                      -6.082272e-03
## frailty(Trial_start_date_factor, distribution = "gaussian")                    -3.010744e-05
## Oviposition_positiveY:Tempscaled                                                1.343141e-03
## Oviposition_positiveY:Agescaled                                                 7.784100e-03
## Tempscaled:Agescaled                                                            1.897833e-03
## Oviposition_positiveY:Tempscaled:Agescaled                                     -2.328642e-03
##                                                               Tempscaled:Agescaled
## Oviposition_positiveY                                                -1.267332e-03
## Tempscaled                                                           -2.188875e-03
## Agescaled                                                            -1.837533e-03
## frailty(Trial_start_date_factor, distribution = "gaussian")          -5.210932e-05
## Oviposition_positiveY:Tempscaled                                      2.186485e-03
## Oviposition_positiveY:Agescaled                                       1.897833e-03
## Tempscaled:Agescaled                                                  7.366023e-03
## Oviposition_positiveY:Tempscaled:Agescaled                           -7.207065e-03
##                                                               Oviposition_positiveY:Tempscaled:Agescaled
## Oviposition_positiveY                                                                      1.421707e-03
## Tempscaled                                                                                 2.251577e-03
## Agescaled                                                                                  1.715865e-03
## frailty(Trial_start_date_factor, distribution = "gaussian")                               -1.805634e-05
## Oviposition_positiveY:Tempscaled                                                          -2.573000e-03
## Oviposition_positiveY:Agescaled                                                           -2.328642e-03
## Tempscaled:Agescaled                                                                      -7.207065e-03
## Oviposition_positiveY:Tempscaled:Agescaled                                                 8.852707e-03
##
## Generalized concordance probability:
##                                                               concordance prob.
## Oviposition_positiveY                                                    0.6313
## Tempscaled                                                               0.5347
## Agescaled                                                                0.5084
## frailty(Trial_start_date_factor, distribution = "gaussian")              0.5088
## Oviposition_positiveY:Tempscaled                                         0.5085
## Oviposition_positiveY:Agescaled                                          0.5168
## Tempscaled:Agescaled                                                     0.4474
## Oviposition_positiveY:Tempscaled:Agescaled                               0.5283
##                                                               lower 0.95
## Oviposition_positiveY                                             0.5919
```

```
## Tempscaled                                                    0.4936
## Agescaled                                                     0.4683
## frailty(Trial_start_date_factor, distribution = "gaussian")   0.5062
## Oviposition_positiveY:Tempscaled                              0.4632
## Oviposition_positiveY:Agescaled                               0.4736
## Tempscaled:Agescaled                                          0.4063
## Oviposition_positiveY:Tempscaled:Agescaled                    0.4822
##                                                           upper 0.95
## Oviposition_positiveY                                         0.6691
## Tempscaled                                                    0.5753
## Agescaled                                                     0.5485
## frailty(Trial_start_date_factor, distribution = "gaussian")   0.5114
## Oviposition_positiveY:Tempscaled                              0.5536
## Oviposition_positiveY:Agescaled                               0.5598
## Tempscaled:Agescaled                                          0.4893
## Oviposition_positiveY:Tempscaled:Agescaled                    0.5738
```

```
plot(fit1$dfbeta.resid)
```

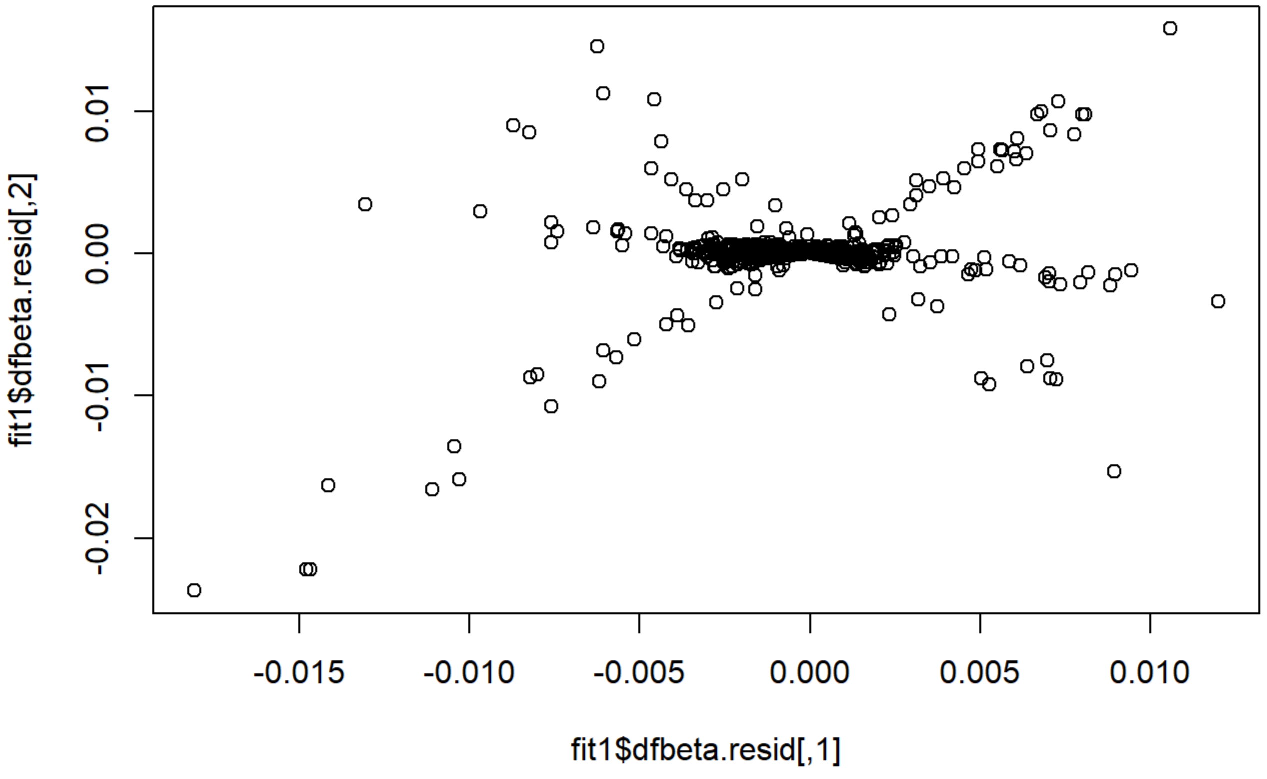


```
fit1
```

```
## coxphw(formula = Surv(start_time, stop_time, Censor) ~ Oviposition_positive *
##     Tempscaled * Agescaled + frailty(Trial_start_date_factor,
##     distribution = "gaussian"), data = Fecundity_data_firstBM_numeric,
##     template = "AHR")
##
## Model fitted by weighted estimation (AHR template)
##
##                                                                   coef
## Oviposition_positiveY                                       0.53794958
## Tempscaled                                                  0.13891638
## Agescaled                                                   0.03374258
## frailty(Trial_start_date_factor, distribution = "gaussian")  0.03519474
## Oviposition_positiveY:Tempscaled                            0.03386528
## Oviposition_positiveY:Agescaled                             0.06726305
## Tempscaled:Agescaled                                       -0.21099691
## Oviposition_positiveY:Tempscaled:Agescaled                  0.11314574
##                                                               se(coef)
## Oviposition_positiveY                                      0.084783356
## Tempscaled                                                 0.083972147
## Agescaled                                                  0.082010914
## frailty(Trial_start_date_factor, distribution = "gaussian") 0.005372499
## Oviposition_positiveY:Tempscaled                           0.092471481
## Oviposition_positiveY:Agescaled                            0.088227545
## Tempscaled:Agescaled                                       0.085825541
## Oviposition_positiveY:Tempscaled:Agescaled                 0.094088824
##                                                            exp(coef)
## Oviposition_positiveY                                      1.7124919
## Tempscaled                                                 1.1490280
## Agescaled                                                  1.0343183
## frailty(Trial_start_date_factor, distribution = "gaussian") 1.0358214
## Oviposition_positiveY:Tempscaled                           1.0344452
## Oviposition_positiveY:Agescaled                            1.0695768
## Tempscaled:Agescaled                                       0.8097766
## Oviposition_positiveY:Tempscaled:Agescaled                 1.1197951
##                                                            lower 0.95
## Oviposition_positiveY                                       1.4503099
## Tempscaled                                                  0.9746606
## Agescaled                                                   0.8807373
## frailty(Trial_start_date_factor, distribution = "gaussian")  1.0249715
## Oviposition_positiveY:Tempscaled                            0.8629699
## Oviposition_positiveY:Agescaled                             0.8997307
## Tempscaled:Agescaled                                        0.6844006
## Oviposition_positiveY:Tempscaled:Agescaled                  0.9312151
##                                                            upper 0.95
## Oviposition_positiveY                                       2.0220703
## Tempscaled                                                  1.3545899
## Agescaled                                                   1.2146804
## frailty(Trial_start_date_factor, distribution = "gaussian")  1.0467861
## Oviposition_positiveY:Tempscaled                            1.2399934
## Oviposition_positiveY:Agescaled                             1.2714855
## Tempscaled:Agescaled                                        0.9581203
## Oviposition_positiveY:Tempscaled:Agescaled                  1.3465644
##                                                                    z
## Oviposition_positiveY                                       6.3449904
## Tempscaled                                                  1.6543150
## Agescaled                                                   0.4114401
```

```
## frailty(Trial_start_date_factor, distribution = "gaussian")  6.5509070
## Oviposition_positiveY:Tempscaled                              0.3662240
## Oviposition_positiveY:Agescaled                               0.7623816
## Tempscaled:Agescaled                                         -2.4584396
## Oviposition_positiveY:Tempscaled:Agescaled                    1.2025417
##                                                                        p
## Oviposition_positiveY                                        2.224393e-10
## Tempscaled                                                   9.806353e-02
## Agescaled                                                    6.807498e-01
## frailty(Trial_start_date_factor, distribution = "gaussian") 5.718870e-11
## Oviposition_positiveY:Tempscaled                             7.141979e-01
## Oviposition_positiveY:Agescaled                              4.458323e-01
## Tempscaled:Agescaled                                         1.395422e-02
## Oviposition_positiveY:Tempscaled:Agescaled                   2.291537e-01
##
## Wald Chi-square=118.947 on 8df, p=0, n=842
```

```
fit2 <- coxphw(Surv(start_time,stop_time, Censor) ~ Oviposition_positive+
                 Tempscaled*Agescaled +
                 frailty(Trial_start_date_factor,distribution = "gaussian"),
               data =Fecundity_data_firstBM_numeric,
               template = "AHR")
summary(fit2)
```

```
## coxphw(formula = Surv(start_time, stop_time, Censor) ~ Oviposition_positive +
##     Tempscaled * Agescaled + frailty(Trial_start_date_factor,
##     distribution = "gaussian"), data = Fecundity_data_firstBM_numeric,
##     template = "AHR")
##
## Model fitted by weighted estimation (AHR template)
##
##                                                                  coef
## Oviposition_positiveY                                      0.51266720
## Tempscaled                                                 0.16697046
## Agescaled                                                  0.09197763
## frailty(Trial_start_date_factor, distribution = "gaussian")  0.03528969
## Tempscaled:Agescaled                                      -0.11391603
##                                                              se(coef)
## Oviposition_positiveY                                     0.087030789
## Tempscaled                                                0.038223302
## Agescaled                                                 0.044824378
## frailty(Trial_start_date_factor, distribution = "gaussian") 0.005375521
## Tempscaled:Agescaled                                      0.038964943
##                                                           exp(coef)
## Oviposition_positiveY                                     1.6697388
## Tempscaled                                                1.1817194
## Agescaled                                                 1.0963403
## frailty(Trial_start_date_factor, distribution = "gaussian") 1.0359198
## Tempscaled:Agescaled                                      0.8923329
##                                                           lower 0.95
## Oviposition_positiveY                                      1.4078870
## Tempscaled                                                 1.0964242
## Agescaled                                                  1.0041320
## frailty(Trial_start_date_factor, distribution = "gaussian")  1.0250628
## Tempscaled:Agescaled                                       0.8267227
##                                                           upper 0.95
## Oviposition_positiveY                                       1.980292
## Tempscaled                                                  1.273650
## Agescaled                                                   1.197016
## frailty(Trial_start_date_factor, distribution = "gaussian")   1.046892
## Tempscaled:Agescaled                                        0.963150
##                                                                   z
## Oviposition_positiveY                                      5.890642
## Tempscaled                                                 4.368290
## Agescaled                                                  2.051955
## frailty(Trial_start_date_factor, distribution = "gaussian")  6.564887
## Tempscaled:Agescaled                                      -2.923552
##                                                                     p
## Oviposition_positiveY                                     3.846987e-09
## Tempscaled                                                1.252232e-05
## Agescaled                                                 4.017399e-02
## frailty(Trial_start_date_factor, distribution = "gaussian") 5.207224e-11
## Tempscaled:Agescaled                                      3.460626e-03
##
## Wald Chi-square = 104.7091 on 5  df  p = 0  n = 842
##
## Covariance-Matrix:
##                                                           Oviposition_positiveY
## Oviposition_positiveY                                              7.574358e-03
## Tempscaled                                                        -1.377205e-04
```

```
## Agescaled                                                               -1.815178e-05
## frailty(Trial_start_date_factor, distribution = "gaussian")             2.955267e-05
## Tempscaled:Agescaled                                                     9.268221e-05
##                                                                Tempscaled
## Oviposition_positiveY                                          -1.377205e-04
## Tempscaled                                                      1.461021e-03
## Agescaled                                                      -1.009677e-04
## frailty(Trial_start_date_factor, distribution = "gaussian") -3.101525e-05
## Tempscaled:Agescaled                                           -2.833154e-04
##                                                                 Agescaled
## Oviposition_positiveY                                          -1.815178e-05
## Tempscaled                                                     -1.009677e-04
## Agescaled                                                       2.009225e-03
## frailty(Trial_start_date_factor, distribution = "gaussian")  7.887617e-05
## Tempscaled:Agescaled                                           -4.457715e-04
##                                                                frailty(Trial_start_date_factor, distributio
n = "gaussian")
## Oviposition_positiveY
2.955267e-05
## Tempscaled
-3.101525e-05
## Agescaled
7.887617e-05
## frailty(Trial_start_date_factor, distribution = "gaussian")
2.889623e-05
## Tempscaled:Agescaled
-6.795179e-05
##                                                                Tempscaled:Agescaled
## Oviposition_positiveY                                                  9.268221e-05
## Tempscaled                                                            -2.833154e-04
## Agescaled                                                             -4.457715e-04
## frailty(Trial_start_date_factor, distribution = "gaussian")        -6.795179e-05
## Tempscaled:Agescaled                                                   1.518267e-03
##
## Generalized concordance probability:
##                                                                concordance prob.
## Oviposition_positiveY                                                     0.6254
## Tempscaled                                                                0.5416
## Agescaled                                                                 0.5230
## frailty(Trial_start_date_factor, distribution = "gaussian")            0.5088
## Tempscaled:Agescaled                                                      0.4716
##                                                                lower 0.95
## Oviposition_positiveY                                              0.5847
## Tempscaled                                                         0.5230
## Agescaled                                                          0.5010
## frailty(Trial_start_date_factor, distribution = "gaussian")     0.5062
## Tempscaled:Agescaled                                               0.4526
##                                                                upper 0.95
## Oviposition_positiveY                                              0.6645
## Tempscaled                                                         0.5602
## Agescaled                                                          0.5448
## frailty(Trial_start_date_factor, distribution = "gaussian")     0.5115
## Tempscaled:Agescaled                                               0.4906
```

```
plot(fit2$dfbeta.resid) # resdiuals look better
```

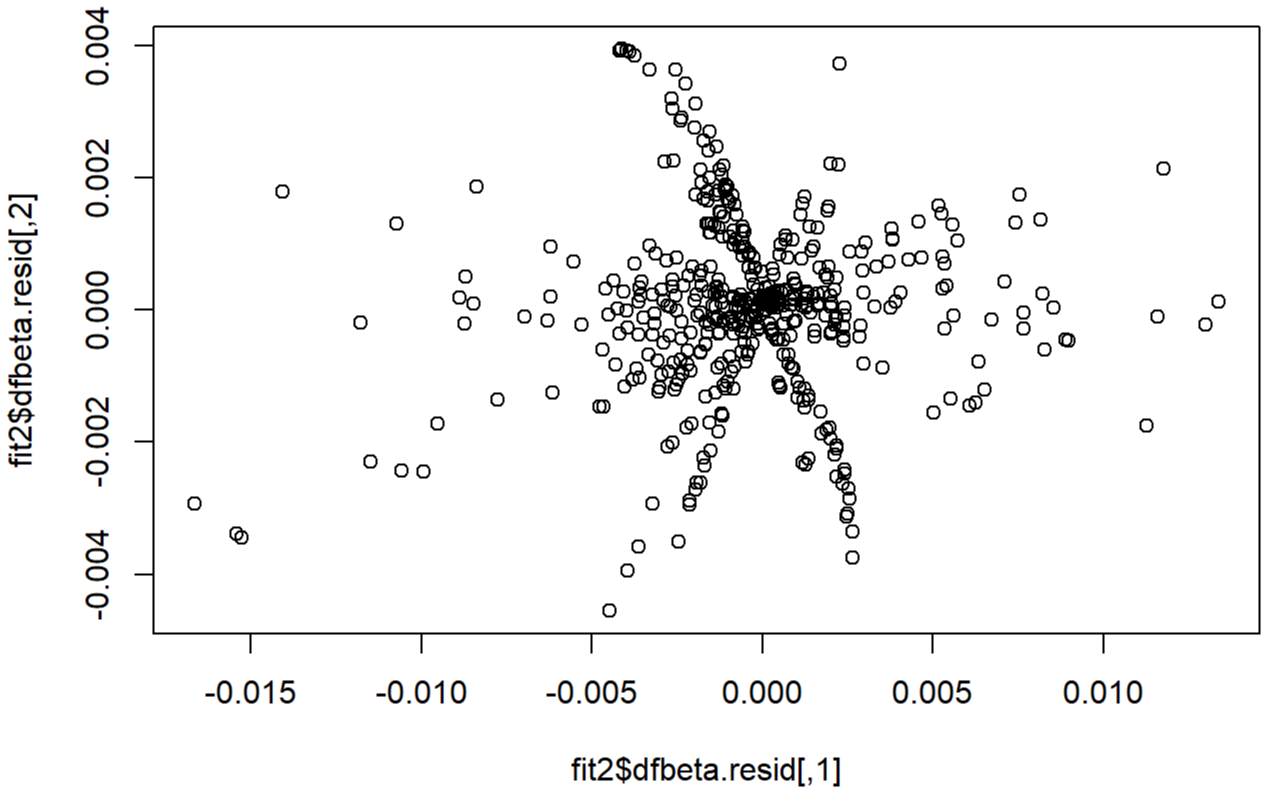

0.004
0.002
0.000
-0.002
-0.004
fit2$dfbeta.resid[,2]
-0.015
-0.010
-0.005
0.000
0.005
0.010
fit2$dfbeta.resid[,1]


```
fit2
```

```
## coxphw(formula = Surv(start_time, stop_time, Censor) ~ Oviposition_positive +
##     Tempscaled * Agescaled + frailty(Trial_start_date_factor,
##     distribution = "gaussian"), data = Fecundity_data_firstBM_numeric,
##     template = "AHR")
##
## Model fitted by weighted estimation (AHR template)
##
##                                                                 coef
## Oviposition_positiveY                                       0.51266720
## Tempscaled                                                  0.16697046
## Agescaled                                                   0.09197763
## frailty(Trial_start_date_factor, distribution = "gaussian")  0.03528969
## Tempscaled:Agescaled                                       -0.11391603
##                                                               se(coef)
## Oviposition_positiveY                                      0.087030789
## Tempscaled                                                 0.038223302
## Agescaled                                                  0.044824378
## frailty(Trial_start_date_factor, distribution = "gaussian") 0.005375521
## Tempscaled:Agescaled                                       0.038964943
##                                                            exp(coef)
## Oviposition_positiveY                                      1.6697388
## Tempscaled                                                 1.1817194
## Agescaled                                                  1.0963403
## frailty(Trial_start_date_factor, distribution = "gaussian") 1.0359198
## Tempscaled:Agescaled                                       0.8923329
##                                                            lower 0.95
## Oviposition_positiveY                                       1.4078870
## Tempscaled                                                  1.0964242
## Agescaled                                                   1.0041320
## frailty(Trial_start_date_factor, distribution = "gaussian")  1.0250628
## Tempscaled:Agescaled                                        0.8267227
##                                                            upper 0.95
## Oviposition_positiveY                                        1.980292
## Tempscaled                                                   1.273650
## Agescaled                                                    1.197016
## frailty(Trial_start_date_factor, distribution = "gaussian")   1.046892
## Tempscaled:Agescaled                                         0.963150
##                                                                    z
## Oviposition_positiveY                                       5.890642
## Tempscaled                                                  4.368290
## Agescaled                                                   2.051955
## frailty(Trial_start_date_factor, distribution = "gaussian")  6.564887
## Tempscaled:Agescaled                                       -2.923552
##                                                                       p
## Oviposition_positiveY                                      3.846987e-09
## Tempscaled                                                 1.252232e-05
## Agescaled                                                  4.017399e-02
## frailty(Trial_start_date_factor, distribution = "gaussian") 5.207224e-11
## Tempscaled:Agescaled                                       3.460626e-03
##
## Wald Chi-square=104.7091 on 5df, p=0, n=842
```

```
fit3 <- coxphw(Surv(start_time,stop_time, Censor) ~ Oviposition_positive+
                 Tempscaled*Agescaled,
               data =Fecundity_data_firstBM_numeric,
               template = "AHR")
summary(fit3)
```

```
## coxphw(formula = Surv(start_time, stop_time, Censor) ~ Oviposition_positive +
##     Tempscaled * Agescaled, data = Fecundity_data_firstBM_numeric,
##     template = "AHR")
##
## Model fitted by weighted estimation (AHR template)
##
##                               coef   se(coef) exp(coef) lower 0.95 upper 0.95
## Oviposition_positiveY  0.50922416 0.08985551 1.6639997  1.3953016   1.984442
## Tempscaled             0.21851932 0.03639554 1.2442330  1.1585687   1.336231
## Agescaled             -0.05272956 0.04365452 0.9486365  0.8708454   1.033377
## Tempscaled:Agescaled  -0.02914481 0.03761270 0.9712758  0.9022494   1.045583
##                                z            p
## Oviposition_positiveY  5.6671446 1.451968e-08
## Tempscaled             6.0040140 1.924981e-09
## Agescaled             -1.2078832 2.270922e-01
## Tempscaled:Agescaled  -0.7748662 4.384187e-01
##
## Wald Chi-square = 69.23859 on 4  df  p = 3.28626e-14  n = 842
##
## Covariance-Matrix:
##                       Oviposition_positiveY    Tempscaled     Agescaled
## Oviposition_positiveY          8.074012e-03 -1.287634e-05 -1.750521e-04
## Tempscaled                    -1.287634e-05  1.324635e-03 -3.284888e-05
## Agescaled                     -1.750521e-04 -3.284888e-05  1.905717e-03
## Tempscaled:Agescaled           9.301994e-05 -3.111169e-04 -2.124675e-04
##                       Tempscaled:Agescaled
## Oviposition_positiveY         9.301994e-05
## Tempscaled                   -3.111169e-04
## Agescaled                    -2.124675e-04
## Tempscaled:Agescaled          1.414715e-03
##
## Generalized concordance probability:
##                       concordance prob. lower 0.95 upper 0.95
## Oviposition_positiveY            0.6246     0.5825     0.6649
## Tempscaled                       0.5544     0.5367     0.5720
## Agescaled                        0.4868     0.4655     0.5082
## Tempscaled:Agescaled             0.4927     0.4743     0.5111
```

```
plot(fit3$dfbeta.resid) # resdiuals look better
```

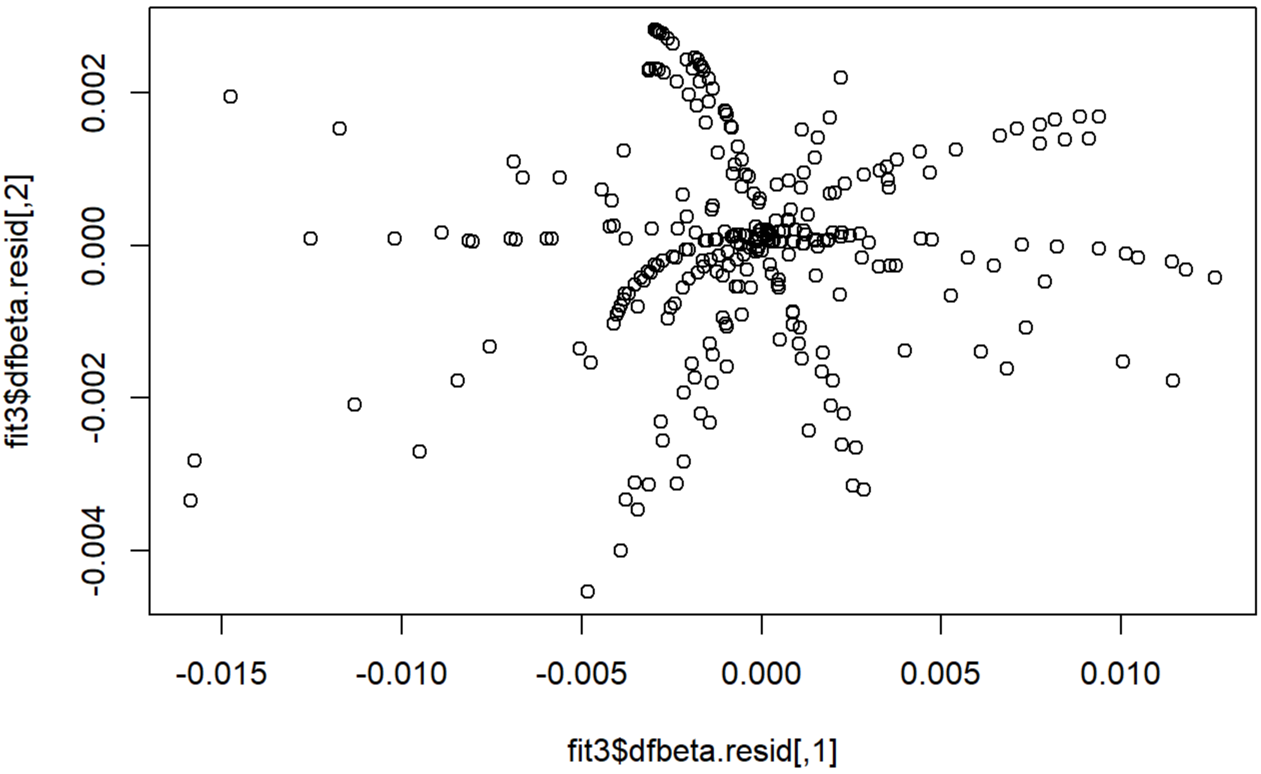


```
fit4 <- coxphw(Surv(start_time,stop_time, Censor) ~ Tempscaled*Agescaled +
                 strata(Oviposition_positive)+
                 frailty(Trial_start_date_factor,distribution = "gaussian"),
               data =Fecundity_data_firstBM_numeric,
               template = "AHR")
summary(fit4)
```

```
## coxphw(formula = Surv(start_time, stop_time, Censor) ~ Tempscaled *
##     Agescaled + strata(Oviposition_positive) + frailty(Trial_start_date_factor,
##     distribution = "gaussian"), data = Fecundity_data_firstBM_numeric,
##     template = "AHR")
##
## Model fitted by weighted estimation (AHR template)
##
##                                                                       coef
## Tempscaled                                                      0.16697046
## Agescaled                                                       0.09197763
## strata(Oviposition_positive)Y                                   0.51266720
## frailty(Trial_start_date_factor, distribution = "gaussian")  0.03528969
## Tempscaled:Agescaled                                           -0.11391603
##                                                                   se(coef)
## Tempscaled                                                     0.038223302
## Agescaled                                                      0.044824378
## strata(Oviposition_positive)Y                                  0.087030789
## frailty(Trial_start_date_factor, distribution = "gaussian") 0.005375521
## Tempscaled:Agescaled                                           0.038964943
##                                                                exp(coef)
## Tempscaled                                                     1.1817194
## Agescaled                                                      1.0963403
## strata(Oviposition_positive)Y                                  1.6697388
## frailty(Trial_start_date_factor, distribution = "gaussian") 1.0359198
## Tempscaled:Agescaled                                           0.8923329
##                                                                lower 0.95
## Tempscaled                                                      1.0964242
## Agescaled                                                       1.0041320
## strata(Oviposition_positive)Y                                   1.4078870
## frailty(Trial_start_date_factor, distribution = "gaussian")  1.0250628
## Tempscaled:Agescaled                                            0.8267227
##                                                                upper 0.95
## Tempscaled                                                       1.273650
## Agescaled                                                        1.197016
## strata(Oviposition_positive)Y                                    1.980292
## frailty(Trial_start_date_factor, distribution = "gaussian")   1.046892
## Tempscaled:Agescaled                                             0.963150
##                                                                        z
## Tempscaled                                                      4.368290
## Agescaled                                                       2.051955
## strata(Oviposition_positive)Y                                   5.890642
## frailty(Trial_start_date_factor, distribution = "gaussian")  6.564887
## Tempscaled:Agescaled                                           -2.923552
##                                                                           p
## Tempscaled                                                     1.252232e-05
## Agescaled                                                      4.017399e-02
## strata(Oviposition_positive)Y                                  3.846987e-09
## frailty(Trial_start_date_factor, distribution = "gaussian") 5.207224e-11
## Tempscaled:Agescaled                                           3.460626e-03
##
## Wald Chi-square = 104.7091 on 5  df  p = 0  n = 842
##
## Covariance-Matrix:
##                                                                   Tempscaled
## Tempscaled                                                      1.461021e-03
## Agescaled                                                      -1.009677e-04
```

```
## strata(Oviposition_positive)Y                                -1.377205e-04
## frailty(Trial_start_date_factor, distribution = "gaussian") -3.101525e-05
## Tempscaled:Agescaled                                        -2.833154e-04
##                                                                 Agescaled
## Tempscaled                                                  -1.009677e-04
## Agescaled                                                    2.009225e-03
## strata(Oviposition_positive)Y                               -1.815178e-05
## frailty(Trial_start_date_factor, distribution = "gaussian")  7.887617e-05
## Tempscaled:Agescaled                                        -4.457715e-04
##                                                             strata(Oviposition_positive)Y
## Tempscaled                                                                 -1.377205e-04
## Agescaled                                                                  -1.815178e-05
## strata(Oviposition_positive)Y                                               7.574358e-03
## frailty(Trial_start_date_factor, distribution = "gaussian")                 2.955267e-05
## Tempscaled:Agescaled                                                        9.268221e-05
##                                                             frailty(Trial_start_date_factor, distributio
n = "gaussian")
## Tempscaled
-3.101525e-05
## Agescaled
7.887617e-05
## strata(Oviposition_positive)Y
2.955267e-05
## frailty(Trial_start_date_factor, distribution = "gaussian")
2.889623e-05
## Tempscaled:Agescaled
-6.795179e-05
##                                                             Tempscaled:Agescaled
## Tempscaled                                                         -2.833154e-04
## Agescaled                                                          -4.457715e-04
## strata(Oviposition_positive)Y                                       9.268221e-05
## frailty(Trial_start_date_factor, distribution = "gaussian")        -6.795179e-05
## Tempscaled:Agescaled                                                1.518267e-03
##
## Generalized concordance probability:
##                                                             concordance prob.
## Tempscaled                                                             0.5416
## Agescaled                                                              0.5230
## strata(Oviposition_positive)Y                                          0.6254
## frailty(Trial_start_date_factor, distribution = "gaussian")            0.5088
## Tempscaled:Agescaled                                                   0.4716
##                                                             lower 0.95
## Tempscaled                                                      0.5230
## Agescaled                                                       0.5010
## strata(Oviposition_positive)Y                                   0.5847
## frailty(Trial_start_date_factor, distribution = "gaussian")     0.5062
## Tempscaled:Agescaled                                            0.4526
##                                                             upper 0.95
## Tempscaled                                                      0.5602
## Agescaled                                                       0.5448
## strata(Oviposition_positive)Y                                   0.6645
## frailty(Trial_start_date_factor, distribution = "gaussian")     0.5115
## Tempscaled:Agescaled                                            0.4906
```

```
plot(fit4$dfbeta.resid)
```

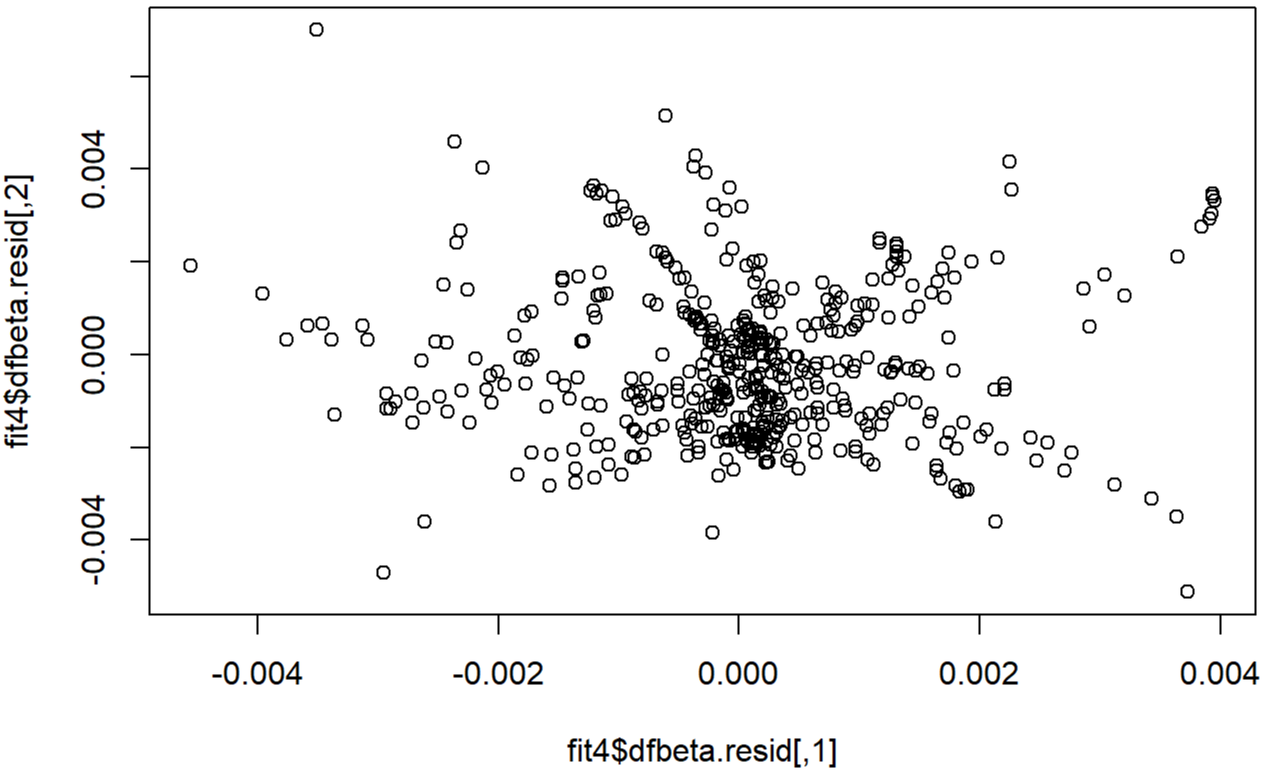
fit4$dfbeta.resid[,2]
-0.004
0.000
0.004
-0.004
-0.002
0.000
0.002
0.004
fit4$dfbeta.resid[,1]

```
fit4
```

```
## coxphw(formula = Surv(start_time, stop_time, Censor) ~ Tempscaled *
##     Agescaled + strata(Oviposition_positive) + frailty(Trial_start_date_factor,
##     distribution = "gaussian"), data = Fecundity_data_firstBM_numeric,
##     template = "AHR")
##
## Model fitted by weighted estimation (AHR template)
##
##                                                                      coef
## Tempscaled                                                     0.16697046
## Agescaled                                                      0.09197763
## strata(Oviposition_positive)Y                                  0.51266720
## frailty(Trial_start_date_factor, distribution = "gaussian")  0.03528969
## Tempscaled:Agescaled                                          -0.11391603
##                                                                  se(coef)
## Tempscaled                                                    0.038223302
## Agescaled                                                     0.044824378
## strata(Oviposition_positive)Y                                 0.087030789
## frailty(Trial_start_date_factor, distribution = "gaussian") 0.005375521
## Tempscaled:Agescaled                                          0.038964943
##                                                               exp(coef)
## Tempscaled                                                    1.1817194
## Agescaled                                                     1.0963403
## strata(Oviposition_positive)Y                                 1.6697388
## frailty(Trial_start_date_factor, distribution = "gaussian") 1.0359198
## Tempscaled:Agescaled                                          0.8923329
##                                                               lower 0.95
## Tempscaled                                                     1.0964242
## Agescaled                                                      1.0041320
## strata(Oviposition_positive)Y                                  1.4078870
## frailty(Trial_start_date_factor, distribution = "gaussian")  1.0250628
## Tempscaled:Agescaled                                           0.8267227
##                                                               upper 0.95
## Tempscaled                                                      1.273650
## Agescaled                                                       1.197016
## strata(Oviposition_positive)Y                                   1.980292
## frailty(Trial_start_date_factor, distribution = "gaussian")   1.046892
## Tempscaled:Agescaled                                            0.963150
##                                                                       z
## Tempscaled                                                     4.368290
## Agescaled                                                      2.051955
## strata(Oviposition_positive)Y                                  5.890642
## frailty(Trial_start_date_factor, distribution = "gaussian")  6.564887
## Tempscaled:Agescaled                                          -2.923552
##                                                                        p
## Tempscaled                                                    1.252232e-05
## Agescaled                                                     4.017399e-02
## strata(Oviposition_positive)Y                                 3.846987e-09
## frailty(Trial_start_date_factor, distribution = "gaussian") 5.207224e-11
## Tempscaled:Agescaled                                          3.460626e-03
##
## Wald Chi-square=104.7091 on 5df, p=0, n=842
```

```
sink("Oviposition_survival/firstBM_coxphwsurvival_oviposneg.txt")
fit4
summary(fit4)
sink()


fit4$coefficients
```

```
##             [,1]
## [1,]  0.16697046
## [2,]  0.09197763
## [3,]  0.51266720
## [4,]  0.03528969
## [5,] -0.11391603
## attr(,"names")
## [1] "Tempscaled"
## [2] "Agescaled"
## [3] "strata(Oviposition_positive)Y"
## [4] "frailty(Trial_start_date_factor, distribution = \"gaussian\")"
## [5] "Tempscaled:Agescaled"
```

```
#extract coefficients to plot hazard ratios:
coef <- as.numeric(fit4$coefficients[1:5])
expcoef <- as.numeric(exp(fit4$coefficients[1:5]))
names <- names(fit4$coefficients[1:5])
lowerCI <- as.numeric(fit4$ci.lower[1:5])
upperCI <- as.numeric(fit4$ci.upper[1:5])
pval <- as.numeric(fit4$prob[1:5])
Index <- c(1:4)
Label <- names(fit4$coefficients[1:5])

hazardratiotable <- as.data.frame(cbind(Label,coef,expcoef,lowerCI,upperCI,pval))
hazardratiotable
```

```
##                                                         Label
## 1                                                  Tempscaled
## 2                                                   Agescaled
## 3                               strata(Oviposition_positive)Y
## 4 frailty(Trial_start_date_factor, distribution = "gaussian")
## 5                                        Tempscaled:Agescaled
##                coef          expcoef           lowerCI           upperCI
## 1  0.166970461888475 1.18171935903461  1.09642421027079  1.27364995266958
## 2 0.0919776277452209 1.09634029420206  1.00413197401126  1.19701600168105
## 3  0.512667199126956 1.66973878683712   1.4078869846177  1.98029220152592
## 4 0.0352896904271831 1.03591976137455  1.02506278292635  1.04689173178517
## 5 -0.113916029439603 0.89233288242875 0.826722728700754 0.963149972077059
##                  pval
## 1 1.25223228130134e-05
## 2   0.0401739908613542
## 3 3.84698661815008e-09
## 4 5.20722354124814e-11
## 5  0.00346062610387232
```

```
hazardratiotable <- hazardratiotable[-4,]

hazardratiotable <- as.data.frame(cbind(Index,hazardratiotable))

print(hazardratiotable)
```

```
##   Index                          Label              coef          expcoef
## 1     1                     Tempscaled  0.166970461888475 1.18171935903461
## 2     2                      Agescaled 0.0919776277452209 1.09634029420206
## 3     3 strata(Oviposition_positive)Y  0.512667199126956 1.66973878683712
## 5     4          Tempscaled:Agescaled -0.113916029439603 0.89233288242875
##             lowerCI           upperCI                 pval
## 1  1.09642421027079  1.27364995266958 1.25223228130134e-05
## 2  1.00413197401126  1.19701600168105   0.0401739908613542
## 3   1.4078869846177  1.98029220152592 3.84698661815008e-09
## 5 0.826722728700754 0.963149972077059  0.00346062610387232
```

```
str(hazardratiotable)
```

```
## 'data.frame':    4 obs. of  7 variables:
##  $ Index  : int  1 2 3 4
##  $ Label  : chr  "Tempscaled" "Agescaled" "strata(Oviposition_positive)Y" "Tempscaled:Agescaled"
##  $ coef   : chr  "0.166970461888475" "0.0919776277452209" "0.512667199126956" "-0.113916029439603"
##  $ expcoef: chr  "1.18171935903461" "1.09634029420206" "1.66973878683712" "0.89233288242875"
##  $ lowerCI: chr  "1.09642421027079" "1.00413197401126" "1.4078869846177" "0.826722728700754"
##  $ upperCI: chr  "1.27364995266958" "1.19701600168105" "1.98029220152592" "0.963149972077059"
##  $ pval   : chr  "1.25223228130134e-05" "0.0401739908613542" "3.84698661815008e-09" "0.0034606261038723
2"
```

```
hazardratiotable$coef <- round(as.numeric(hazardratiotable$coef),digits = 3)
hazardratiotable$expcoef <- round(as.numeric(hazardratiotable$expcoef),digits = 3)
hazardratiotable$lowerCI <- round(as.numeric(hazardratiotable$lowerCI),digits = 3)
hazardratiotable$upperCI <- round(as.numeric(hazardratiotable$upperCI),digits = 3)
hazardratiotable$pval <- round(as.numeric(hazardratiotable$pval),digits = 3)
print(hazardratiotable)
```

```
##   Index                          Label   coef expcoef lowerCI upperCI  pval
## 1     1                     Tempscaled  0.167   1.182   1.096   1.274 0.000
## 2     2                      Agescaled  0.092   1.096   1.004   1.197 0.040
## 3     3 strata(Oviposition_positive)Y  0.513   1.670   1.408   1.980 0.000
## 5     4          Tempscaled:Agescaled -0.114   0.892   0.827   0.963 0.003
```

```
hazardratiotable$Label <- c("Temperature","Age",
                            "Has Opportunity to Oviposit","Temperature:Age")
print(hazardratiotable)
```

```
##   Index                       Label   coef expcoef lowerCI upperCI  pval
## 1     1                 Temperature  0.167   1.182   1.096   1.274 0.000
## 2     2                         Age  0.092   1.096   1.004   1.197 0.040
## 3     3 Has Opportunity to Oviposit  0.513   1.670   1.408   1.980 0.000
## 5     4             Temperature:Age -0.114   0.892   0.827   0.963 0.003
```

```
write_xlsx(hazardratiotable,"Oviposition_survival/firstBM_survival_hazardratiotable_oviposneg.xlsx")

plot1 <- ggplot(hazardratiotable, aes(y = Index, x = expcoef)) +
  geom_point(shape = 18, size =4) +
  geom_errorbarh(aes(xmin = lowerCI, xmax = upperCI), height = 0.25) +
  geom_vline(xintercept = 1, color = "gray", linetype = "dashed", cex = 1, alpha = 0.5) +
  scale_y_continuous(name = "",breaks = 1:4,labels = hazardratiotable$Label,  trans = "reverse") +
  xlab("Hazard Ratio (95% CI)") +
  ylab(" ") +
  theme_pubr() +
  theme(panel.border = element_blank(),
        panel.background = element_blank(),
        panel.grid.major = element_blank(),
        panel.grid.minor = element_blank(),
        axis.line = element_line(colour = "black"),
        axis.text.y = element_text(size = 12, colour = "black"),
        axis.text.x.bottom = element_text(size = 12, colour = "black"),
        axis.title.x = element_text(size = 12, colour = "black"))
plot1
```

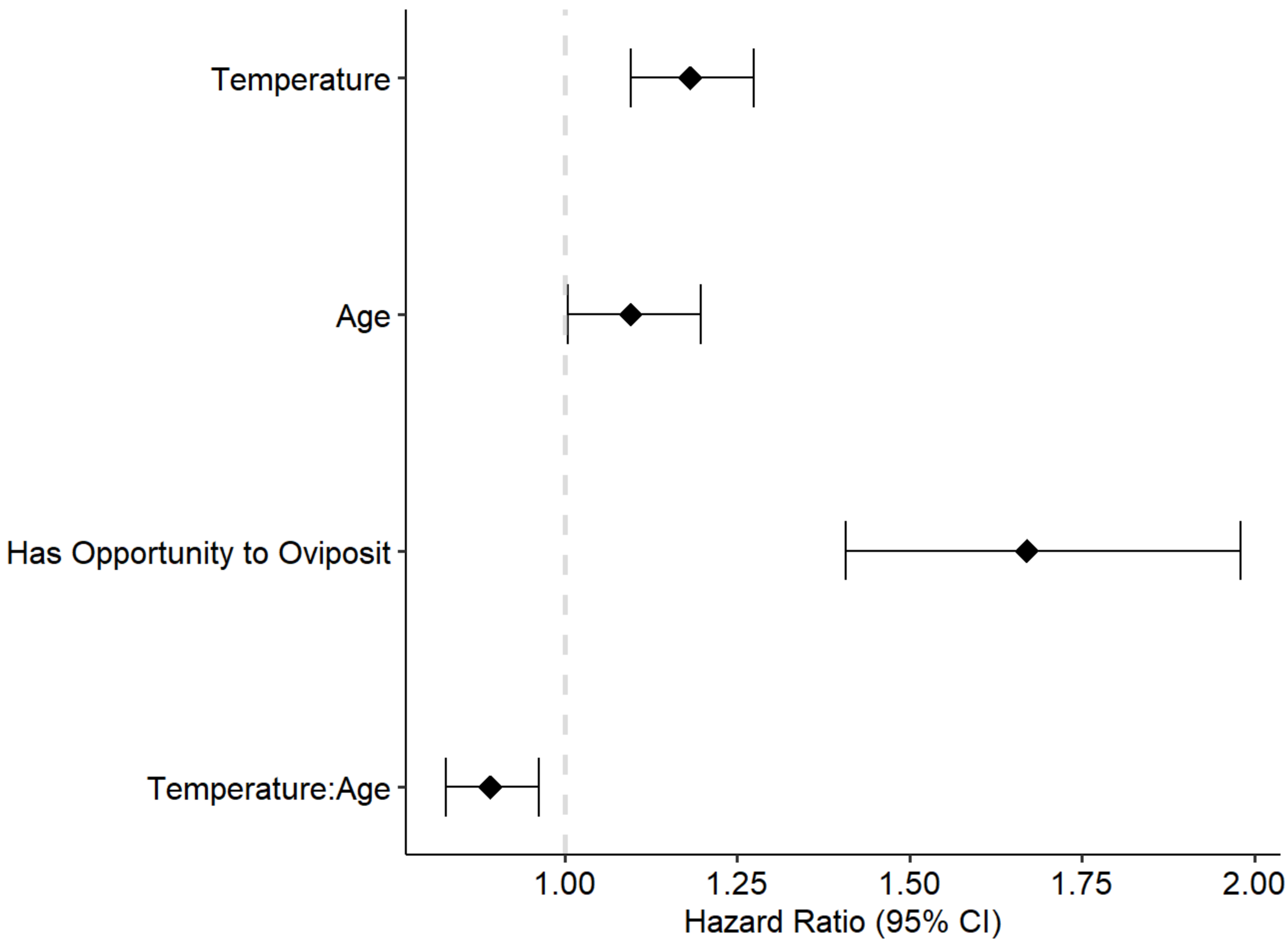

```
#add CIs to plot:
## Create the table-base pallete
table_base <- ggplot(hazardratiotable, aes(y=Label)) +
  ylab(NULL) + xlab("  ") +
  theme(plot.title = element_text(hjust = 0.5, size=12),
        axis.text.x = element_text(color="white", hjust = -3, size = 25), ## This is used to help with alignment
        axis.line = element_blank(),
        axis.text.y = element_blank(),
        axis.ticks = element_blank(),
        axis.title.y = element_blank(),
        legend.position = "none",
        panel.background = element_blank(),
        panel.border = element_blank(),
        panel.grid.major = element_blank(),
        panel.grid.minor = element_blank(),
        plot.background = element_blank())

## HR point estimate table
tab1 <- table_base +
  labs(title = "space") +
  geom_text(aes(y = rev(Index), x = 1, label = sprintf("%0.1f", round(expcoef, digits = 1))), size = 4) + ## decimal places
  ggtitle("HR")
tab1
```

HR

1.2

1.1

1.7

0.9

```r
#pval
tab3 <- table_base +
  geom_text(aes(y = rev(Index), x = 1, label = pval), size = 4) +
  ggtitle("P value")
tab3
```

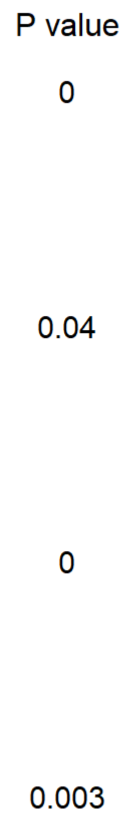


```r
library(gridExtra)
lay <-  matrix(c(1,1,1,1,1,1,1,1,1,1,1,1,1,1,1,1,1,1,1,1,2,2,2), nrow = 1)
bucketsurvival_forest <- grid.arrange(plot1, tab3,layout_matrix = lay)
```

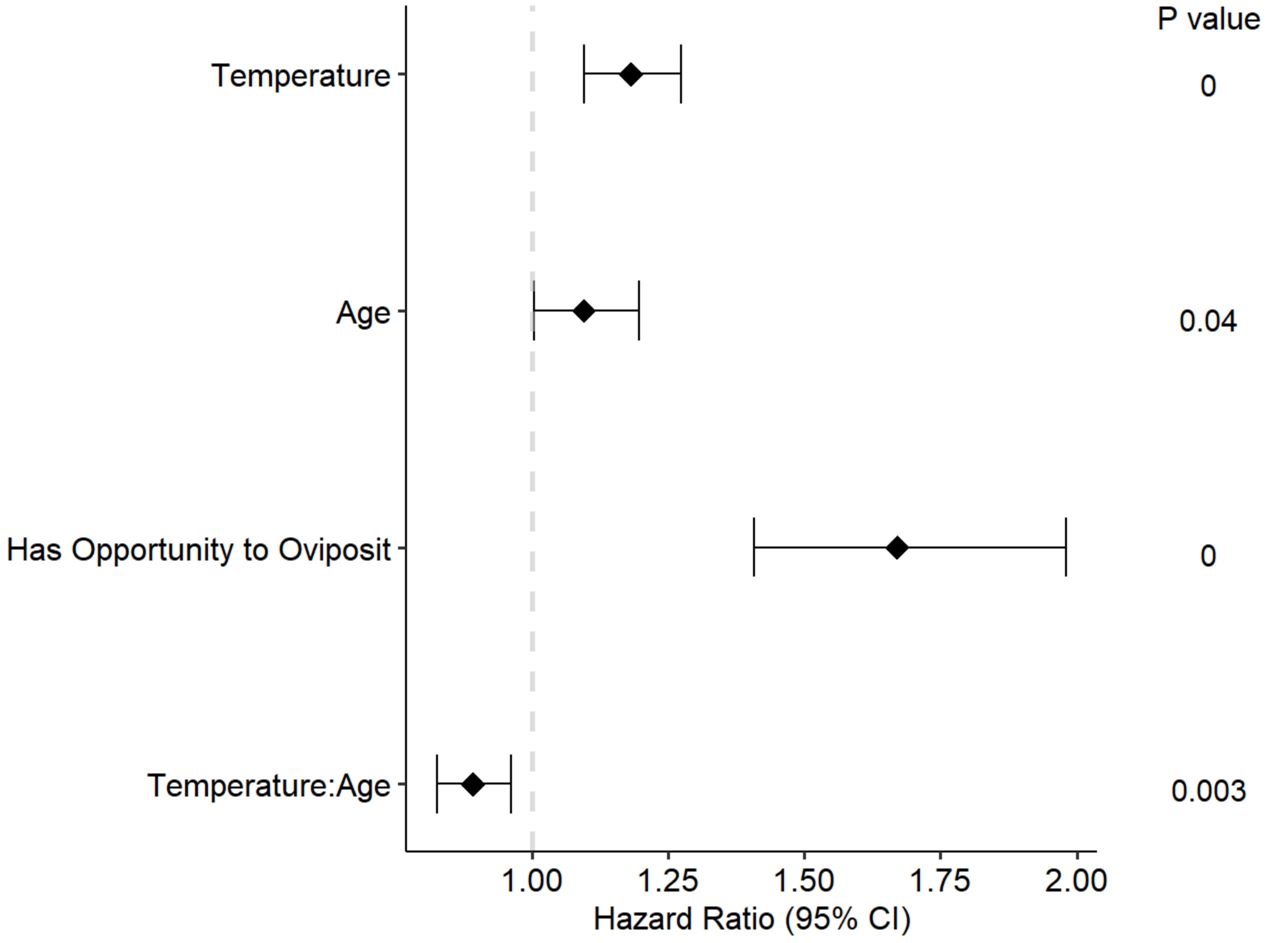


```
ggsave("Oviposition_survival/firstBM_hazardplot_oviposneg.pdf",bucketsurvival_forest,dpi=600,width = 7,heig
ht=5,units="in")
ggsave("Oviposition_survival/firstBM_hazardplot_oviposneg.png",bucketsurvival_forest,dpi=600,width = 7,heig
ht=5,units="in")
```

Code for Figure 8 - survival w/ access to oviposition site, then either laying eggs or not laying eggs

Import data and clean it up.

```
# clear existing workspace
rm(list = ls(all = TRUE))
graphics.off()
shell("cls")

#set wd to your project folder
getwd() #check working directory
```

```
## [1] "C:/Users/linzm/OneDrive - Vanderbilt/Hillyer_Lab/Blood_feeding_project/Bloodfeeding"
```

```
####################
#load libraries needed:
library(readxl)
library(writexl)
library(ggplot2)
library(dplyr)
library(tidyverse)
library(rstatix)
library(car)
library(ggpubr)
library(emmeans)
library(survival)
library(ggsurvfit)
library(survminer)

#######################################################
#import the data and clean it up:



#import the data:
Fecundity_data <- read_xlsx("SupplementaryData1_RawData.xlsx",
                            sheet = "Figs4-9")
Fecundity_data <- as.data.frame(Fecundity_data)
str(Fecundity_data)
```

```
## 'data.frame':    849 obs. of  18 variables:
##  $ ID_overall               : num  1 2 3 4 5 6 7 8 9 10 ...
##  $ Temperature              : num  32 32 32 32 32 32 32 32 32 32 ...
##  $ Age                      : num  3 3 3 3 3 3 3 3 3 3 ...
##  $ ID_per_group             : num  1 2 3 4 5 6 7 8 9 10 ...
##  $ Trial_start_date         : POSIXct, format: "2024-02-13" "2024-02-13" ...
##  $ Trial_number             : num  1 1 1 1 1 1 1 1 1 1 ...
##  $ BM1_Date                 : POSIXct, format: "2024-02-13" "2024-02-13" ...
##  $ Age_of_BM                : num  3 3 3 3 3 3 3 3 3 3 ...
##  $ Bloodmeal_number         : num  1 1 1 1 1 1 1 1 1 1 ...
##  $ Oviposition_positive(y/n): chr  "Y" "Y" "Y" "Y" ...
##  $ Eggs_day3                : chr  "0" "0" "0" "0" ...
##  $ Eggs_day4                : chr  "0" "0" "0" "0" ...
##  $ Larvae_day4              : chr  "0" "0" "0" "0" ...
##  $ Surv_to_eggs(y/n)        : chr  "Y" "Y" "Y" "Y" ...
##  $ Surv_to_larvae(y/n)      : chr  "Y" "Y" "Y" "Y" ...
##  $ Date_of_death            : POSIXct, format: "2024-03-01" "2024-03-07" ...
##  $ Censor                   : num  1 1 1 1 1 1 1 1 1 1 ...
##  $ Notes                    : chr  NA NA NA NA ...
```

```
head(Fecundity_data)
```

```
##   ID_overall Temperature Age ID_per_group Trial_start_date Trial_number
## 1          1          32   3            1       2024-02-13            1
## 2          2          32   3            2       2024-02-13            1
## 3          3          32   3            3       2024-02-13            1
## 4          4          32   3            4       2024-02-13            1
## 5          5          32   3            5       2024-02-13            1
## 6          6          32   3            6       2024-02-13            1
##     BM1_Date Age_of_BM Bloodmeal_number Oviposition_positive(y/n) Eggs_day3
## 1 2024-02-13         3                1                         Y         0
## 2 2024-02-13         3                1                         Y         0
## 3 2024-02-13         3                1                         Y         0
## 4 2024-02-13         3                1                         Y         0
## 5 2024-02-13         3                1                         Y         0
## 6 2024-02-13         3                1                         Y        14
##   Eggs_day4 Larvae_day4 Surv_to_eggs(y/n) Surv_to_larvae(y/n) Date_of_death
## 1         0           0                 Y                   Y    2024-03-01
## 2         0           0                 Y                   Y    2024-03-07
## 3         0           0                 Y                   Y    2024-03-02
## 4         0           0                 Y                   Y    2024-02-26
## 5         0           0                 Y                   Y    2024-02-28
## 6        14           0                 Y                   Y    2024-03-02
##   Censor Notes
## 1      1  <NA>
## 2      1  <NA>
## 3      1  <NA>
## 4      1  <NA>
## 5      1  <NA>
## 6      1  <NA>
```

```
Fecundity_data_numeric <- Fecundity_data

#variables of interest:

Fecundity_data$Temperature <- as.factor(Fecundity_data$Temperature)
Fecundity_data$Age <- as.factor(Fecundity_data$Age)
Fecundity_data$Age_of_BM <- as.numeric(Fecundity_data$Age_of_BM)
Fecundity_data$Bloodmeal_number <- as.factor(Fecundity_data$Bloodmeal_number)

Fecundity_data$Eggs_day3 <- as.numeric(Fecundity_data$Eggs_day3)
```

```
## Warning: NAs introduced by coercion
```

```
Fecundity_data$Eggs_day4 <- as.numeric(Fecundity_data$Eggs_day4)
```

```
## Warning: NAs introduced by coercion
```

```
Fecundity_data$Larvae_day4 <- as.numeric(Fecundity_data$Larvae_day4)
```

```
## Warning: NAs introduced by coercion
```

```
Fecundity_data$Oviposition_positive <- as.factor(Fecundity_data$Oviposition_positive)
Fecundity_data$`Surv_to_eggs(y/n)` <- as.factor(Fecundity_data$`Surv_to_eggs(y/n)`)
Fecundity_data$`Surv_to_larvae(y/n)` <- as.factor(Fecundity_data$`Surv_to_larvae(y/n)`)

str(Fecundity_data)
```

```
## 'data.frame':    849 obs. of  19 variables:
##  $ ID_overall                : num  1 2 3 4 5 6 7 8 9 10 ...
##  $ Temperature               : Factor w/ 3 levels "27","30","32": 3 3 3 3 3 3 3 3 3 3 ...
##  $ Age                       : Factor w/ 4 levels "3","5","10","15": 1 1 1 1 1 1 1 1 1 1 ...
##  $ ID_per_group              : num  1 2 3 4 5 6 7 8 9 10 ...
##  $ Trial_start_date          : POSIXct, format: "2024-02-13" "2024-02-13" ...
##  $ Trial_number              : num  1 1 1 1 1 1 1 1 1 1 ...
##  $ BM1_Date                  : POSIXct, format: "2024-02-13" "2024-02-13" ...
##  $ Age_of_BM                 : num  3 3 3 3 3 3 3 3 3 3 ...
##  $ Bloodmeal_number          : Factor w/ 1 level "1": 1 1 1 1 1 1 1 1 1 1 ...
##  $ Oviposition_positive(y/n): chr  "Y" "Y" "Y" "Y" ...
##  $ Eggs_day3                 : num  0 0 0 0 0 14 NA NA NA NA ...
##  $ Eggs_day4                 : num  0 0 0 0 0 14 NA NA NA NA ...
##  $ Larvae_day4               : num  0 0 0 0 0 0 0 0 NA NA ...
##  $ Surv_to_eggs(y/n)         : Factor w/ 3 levels "N","NA","Y": 3 3 3 3 3 3 1 1 3 3 ...
##  $ Surv_to_larvae(y/n)       : Factor w/ 3 levels "N","NA","Y": 3 3 3 3 3 3 2 2 3 3 ...
##  $ Date_of_death             : POSIXct, format: "2024-03-01" "2024-03-07" ...
##  $ Censor                    : num  1 1 1 1 1 1 1 1 1 1 ...
##  $ Notes                     : chr  NA NA NA NA ...
##  $ Oviposition_positive     : Factor w/ 2 levels "N","Y": 2 2 2 2 2 2 2 2 1 1 ...
```

```
Fecundity_data <- subset(Fecundity_data, Censor == 1) #get rid of mosquitoes censored out by experimental e
rror (get rid of 0 values; 1 = died naturally)

#calculate the total eggs laid per mosquito:

#need to subtract to find ones only laid on day 4 (exclude day 3 eggs)
Fecundity_data$Eggs_day4 <- (Fecundity_data$Eggs_day4)-(Fecundity_data$Eggs_day3)

#replace negative eggs day 4 values with zero (assume miscounted/eggs degraded and no new eggs laid)
for (row in 1:nrow(Fecundity_data)){
  if (is.na(Fecundity_data$Eggs_day4[row])){
    Fecundity_data$Eggs_day4[row] <- NA #keep NA values
  } else if ((Fecundity_data$Eggs_day4[row] <= 0)){
    Fecundity_data$Eggs_day4[row] <- 0
  }
}

#total eggs addition:
Fecundity_data$total_eggs <- (Fecundity_data$Eggs_day3)+(Fecundity_data$Eggs_day4)

#percents:
Fecundity_data$Percent_eggs_day3 <- (Fecundity_data$Eggs_day3) / (Fecundity_data$total_eggs)
Fecundity_data$Percent_eggs_day4 <- (Fecundity_data$Eggs_day4) / (Fecundity_data$total_eggs)

# decide if each mosquito laid eggs and on what day
#day 3
for (row in 1:nrow(Fecundity_data)){
  if(!is.na(Fecundity_data$Eggs_day3[row])){
    if (Fecundity_data$Eggs_day3[row] > 0){
      Fecundity_data$egg_binary[row] = 1
      Fecundity_data$egg_binary_day3[row] = 1
    }
    else {
      Fecundity_data$egg_binary[row] = 0
      Fecundity_data$egg_binary_day3[row] = 0
    }
  }
  else if (is.na(Fecundity_data$Eggs_day3[row])){
    Fecundity_data$egg_binary[row] = NA
    Fecundity_data$egg_binary_day3[row] = NA
  }
}

#day 4
for (row in 1:nrow(Fecundity_data)){
  if(!is.na(Fecundity_data$Eggs_day4[row])){
    if (Fecundity_data$Eggs_day4[row] >0){
      Fecundity_data$egg_binary[row] = 1
      Fecundity_data$egg_binary_day4[row] = 1
    }
    else{
      Fecundity_data$egg_binary_day4[row] = 0
    }
  }
  else if (is.na(Fecundity_data$Eggs_day4[row])){
    Fecundity_data$egg_binary_day4[row] = NA
```

```
  }
}

# decide if each mosquito had larvae
for (row in 1:nrow(Fecundity_data)){
  if(!is.na(Fecundity_data$Larvae_day4[row])){
    if (Fecundity_data$Larvae_day4[row] >0){
      Fecundity_data$larvae_binary[row] = 1
    }
    else{
      Fecundity_data$larvae_binary[row] = 0
    }
  }
  else{
    Fecundity_data$larvae_binary[row] = NA
  }
}

#percents:
Fecundity_data$Percent_eggshatchedtolarv <- (Fecundity_data$Larvae_day4) / (Fecundity_data$Eggs_day3)

#survival:
#calculate:
Fecundity_data$Days_to_death_post_BM <- Fecundity_data$Date_of_death - Fecundity_data$Trial_start_date
Fecundity_data$Age_of_death <- Fecundity_data$Age_of_BM + Fecundity_data$Days_to_death_post_BM

Fecundity_data <-
  Fecundity_data %>%
  mutate(
    Age_of_BM_days = as.difftime(Age_of_BM, unit="days")
  )
Fecundity_data$Date_of_eclosion <- Fecundity_data$Trial_start_date - (Fecundity_data$Age_of_BM_days)

library(lubridate)
Fecundity_data <-
  Fecundity_data %>%
  mutate(
    days_alive_post_BM = as.duration(Trial_start_date %--% Date_of_death) / ddays(1),
    days_alive_post_eclosion = as.duration(Date_of_eclosion %--% Date_of_death) / ddays(1),
  )

str(Fecundity_data)
```

```
## 'data.frame':    842 obs. of  33 variables:
##  $ ID_overall              : num  1 2 3 4 5 6 7 8 9 10 ...
##  $ Temperature             : Factor w/ 3 levels "27","30","32": 3 3 3 3 3 3 3 3 3 3 ...
##  $ Age                     : Factor w/ 4 levels "3","5","10","15": 1 1 1 1 1 1 1 1 1 1 ...
##  $ ID_per_group            : num  1 2 3 4 5 6 7 8 9 10 ...
##  $ Trial_start_date        : POSIXct, format: "2024-02-13" "2024-02-13" ...
##  $ Trial_number            : num  1 1 1 1 1 1 1 1 1 1 ...
##  $ BM1_Date                : POSIXct, format: "2024-02-13" "2024-02-13" ...
##  $ Age_of_BM               : num  3 3 3 3 3 3 3 3 3 3 ...
##  $ Bloodmeal_number        : Factor w/ 1 level "1": 1 1 1 1 1 1 1 1 1 1 ...
##  $ Oviposition_positive(y/n): chr  "Y" "Y" "Y" "Y" ...
##  $ Eggs_day3               : num  0 0 0 0 0 14 NA NA NA NA ...
##  $ Eggs_day4               : num  0 0 0 0 0 0 NA NA NA NA ...
##  $ Larvae_day4             : num  0 0 0 0 0 0 0 0 NA NA ...
##  $ Surv_to_eggs(y/n)       : Factor w/ 3 levels "N","NA","Y": 3 3 3 3 3 3 1 1 3 3 ...
##  $ Surv_to_larvae(y/n)     : Factor w/ 3 levels "N","NA","Y": 3 3 3 3 3 3 2 2 3 3 ...
##  $ Date_of_death           : POSIXct, format: "2024-03-01" "2024-03-07" ...
##  $ Censor                  : num  1 1 1 1 1 1 1 1 1 1 ...
##  $ Notes                   : chr  NA NA NA NA ...
##  $ Oviposition_positive    : Factor w/ 2 levels "N","Y": 2 2 2 2 2 2 2 2 1 1 ...
##  $ total_eggs              : num  0 0 0 0 0 14 NA NA NA NA ...
##  $ Percent_eggs_day3       : num  NaN NaN NaN NaN NaN 1 NA NA NA NA ...
##  $ Percent_eggs_day4       : num  NaN NaN NaN NaN NaN 0 NA NA NA NA ...
##  $ egg_binary              : num  0 0 0 0 0 1 NA NA NA NA ...
##  $ egg_binary_day3         : num  0 0 0 0 0 1 NA NA NA NA ...
##  $ egg_binary_day4         : num  0 0 0 0 0 0 NA NA NA NA ...
##  $ larvae_binary           : num  0 0 0 0 0 0 0 0 NA NA ...
##  $ Percent_eggshatchedtolarv: num  NaN NaN NaN NaN NaN 0 NA NA NA NA ...
##  $ Days_to_death_post_BM   : 'difftime' num  17 23 18 13 ...
##   ..- attr(*, "units")= chr "days"
##  $ Age_of_death            : 'difftime' num  20 26 21 16 ...
##   ..- attr(*, "units")= chr "days"
##  $ Age_of_BM_days          : 'difftime' num  3 3 3 3 ...
##   ..- attr(*, "units")= chr "days"
##  $ Date_of_eclosion        : POSIXct, format: "2024-02-10" "2024-02-10" ...
##  $ days_alive_post_BM      : num  17 23 18 13 15 18 3 3 12 11 ...
##  $ days_alive_post_eclosion : num  20 26 21 16 18 21 6 6 15 14 ...
```

```
Fecundity_data_firstBM <- Fecundity_data
#subset by oviposition positive and negative:
Fecundity_data_ovipos <- subset(Fecundity_data, Oviposition_positive== "Y")
Fecundity_data_ovineg <- subset(Fecundity_data, Oviposition_positive== "N")
```

Plot survival curves

```
library(survival)
# Plot survival curves
#labels first
Fecundity_data_firstBM$group <- paste(Fecundity_data_firstBM$Age,Fecundity_data_firstBM$Temperature,sep
="_")

Fecundity_data_firstBM$Age <- factor(Fecundity_data_firstBM$Age,
                                     labels = c("3 days","5 days","10 days","15 days"))
Fecundity_data_firstBM$Temperature <- factor(Fecundity_data_firstBM$Temperature,
                                             labels = c("27˚C","30˚C","32˚C"))



str(Fecundity_data_firstBM) #make sure dates are dates
```

```
## 'data.frame':    842 obs. of  34 variables:
##  $ ID_overall               : num  1 2 3 4 5 6 7 8 9 10 ...
##  $ Temperature              : Factor w/ 3 levels "27˚C","30˚C",..: 3 3 3 3 3 3 3 3 3 3 ...
##  $ Age                      : Factor w/ 4 levels "3 days","5 days",..: 1 1 1 1 1 1 1 1 1 1 ...
##  $ ID_per_group             : num  1 2 3 4 5 6 7 8 9 10 ...
##  $ Trial_start_date         : POSIXct, format: "2024-02-13" "2024-02-13" ...
##  $ Trial_number             : num  1 1 1 1 1 1 1 1 1 1 ...
##  $ BM1_Date                 : POSIXct, format: "2024-02-13" "2024-02-13" ...
##  $ Age_of_BM                : num  3 3 3 3 3 3 3 3 3 3 ...
##  $ Bloodmeal_number         : Factor w/ 1 level "1": 1 1 1 1 1 1 1 1 1 1 ...
##  $ Oviposition_positive(y/n): chr  "Y" "Y" "Y" "Y" ...
##  $ Eggs_day3                : num  0 0 0 0 0 14 NA NA NA NA ...
##  $ Eggs_day4                : num  0 0 0 0 0 0 NA NA NA NA ...
##  $ Larvae_day4              : num  0 0 0 0 0 0 0 0 NA NA ...
##  $ Surv_to_eggs(y/n)        : Factor w/ 3 levels "N","NA","Y": 3 3 3 3 3 3 1 1 3 3 ...
##  $ Surv_to_larvae(y/n)      : Factor w/ 3 levels "N","NA","Y": 3 3 3 3 3 3 2 2 3 3 ...
##  $ Date_of_death            : POSIXct, format: "2024-03-01" "2024-03-07" ...
##  $ Censor                   : num  1 1 1 1 1 1 1 1 1 1 ...
##  $ Notes                    : chr  NA NA NA NA ...
##  $ Oviposition_positive     : Factor w/ 2 levels "N","Y": 2 2 2 2 2 2 2 2 1 1 ...
##  $ total_eggs               : num  0 0 0 0 0 14 NA NA NA NA ...
##  $ Percent_eggs_day3        : num  NaN NaN NaN NaN NaN 1 NA NA NA NA ...
##  $ Percent_eggs_day4        : num  NaN NaN NaN NaN NaN 0 NA NA NA NA ...
##  $ egg_binary               : num  0 0 0 0 0 1 NA NA NA NA ...
##  $ egg_binary_day3          : num  0 0 0 0 0 1 NA NA NA NA ...
##  $ egg_binary_day4          : num  0 0 0 0 0 0 NA NA NA NA ...
##  $ larvae_binary            : num  0 0 0 0 0 0 0 0 NA NA ...
##  $ Percent_eggshatchedtolarv: num  NaN NaN NaN NaN NaN 0 NA NA NA NA ...
##  $ Days_to_death_post_BM    : 'difftime' num  17 23 18 13 ...
##   ..- attr(*, "units")= chr "days"
##  $ Age_of_death             : 'difftime' num  20 26 21 16 ...
##   ..- attr(*, "units")= chr "days"
##  $ Age_of_BM_days           : 'difftime' num  3 3 3 3 ...
##   ..- attr(*, "units")= chr "days"
##  $ Date_of_eclosion         : POSIXct, format: "2024-02-10" "2024-02-10" ...
##  $ days_alive_post_BM       : num  17 23 18 13 15 18 3 3 12 11 ...
##  $ days_alive_post_eclosion : num  20 26 21 16 18 21 6 6 15 14 ...
##  $ group                    : chr  "3_32" "3_32" "3_32" "3_32" ...
```

```
Fecundity_data_firstBM$start_time <- (Fecundity_data_firstBM$Age_of_BM +1) #start monitoring the day after BM
Fecundity_data_firstBM$stop_time <- (Fecundity_data_firstBM$days_alive_post_eclosion+1) #age of death + 1 # aod can't be sooner than start
Fecundity_data_firstBM$stopminusstart <- Fecundity_data_firstBM$stop_time - Fecundity_data_firstBM$start_time

str(Fecundity_data_firstBM$Oviposition_positive)
```

```
##  Factor w/ 2 levels "N","Y": 2 2 2 2 2 2 2 2 1 1 ...
```

```
Fecundity_data_firstBM_ovipossurvival <- subset(Fecundity_data_firstBM, Oviposition_positive=="Y")

################################################################
str(Fecundity_data_firstBM_ovipossurvival$egg_binary)
```

```
##  num [1:682] 0 0 0 0 0 1 NA NA 0 1 ...
```

```
Fecundity_data_firstBM_ovipossurvival$egg_binary <- factor(Fecundity_data_firstBM_ovipossurvival$egg_binary,
                                                           labels = c("Did not lay eggs","Laid eggs"))

s1 <- survfit2(Surv(stopminusstart, Censor) ~ Temperature+Age+egg_binary,
               data = Fecundity_data_firstBM_ovipossurvival)

ggsurv <- ggsurvplot(s1, conf.int = TRUE, color="Age",palette = c("#DCD1E9","#BAA4D3","#9776BE","#7549A8"),
                     ggtheme = theme_pubr(),surv.median.line = "v",confint=TRUE)

firstBM_survival_ovipos_eggsvsnoeggs <- ggsurv$plot +
  theme_pubr() +
  theme(legend.position = "bottom")+
  facet_grid(Temperature ~ egg_binary)+
  ylab(expression("BM1 Survival probability"))+
  xlab("Time (Days post blood feeding)") +
  theme(panel.background = element_rect(fill = NA, color = "black"))+
  theme(panel.spacing = unit(0.6, "lines"))

firstBM_survival_ovipos_eggsvsnoeggs
```

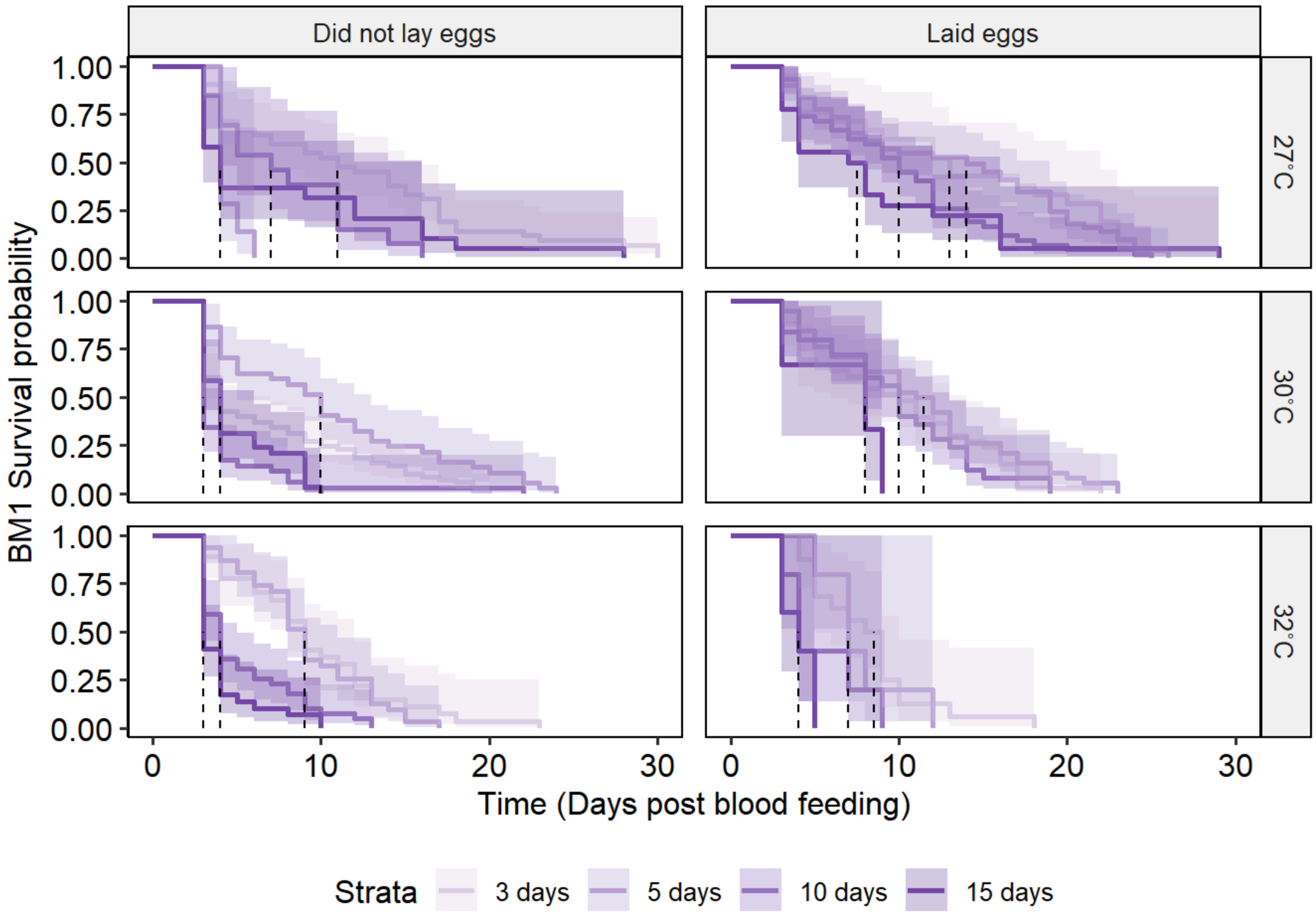


```r
ggsave("Oviposition_survival/firstBM_survival_ovipos_eggsvsnoeggs_stopminusstart.pdf",firstBM_survival_ovipos_eggsvsnoeggs, width = 6, height = 5, units = "in", dpi = 600)
ggsave("Oviposition_survival/firstBM_survival_ovipos_eggsvsnoeggs_stopminusstart.png",firstBM_survival_ovipos_eggsvsnoeggs, width = 6, height = 5, units = "in", dpi = 600)



ggsurv <- ggsurvplot(s1, conf.int = TRUE, color="Temperature",palette = c("#4D6FAE","#6F9F51", "#CC763B"),
                     ggtheme = theme_pubr(),surv.median.line = "v",confint=TRUE)

firstBM_oviposeggsvsnoeggs_flipped <- ggsurv$plot +
  theme_pubr() +
  theme(legend.position = "bottom")+
  facet_grid(Age ~ egg_binary)+
  ylab(expression("BM1 Survival probability"))+
  xlab("Time (Days post blood feeding)") +
  theme(panel.background = element_rect(fill = NA, color = "black"))+
  theme(panel.spacing = unit(0.6, "lines"))

firstBM_oviposeggsvsnoeggs_flipped
```

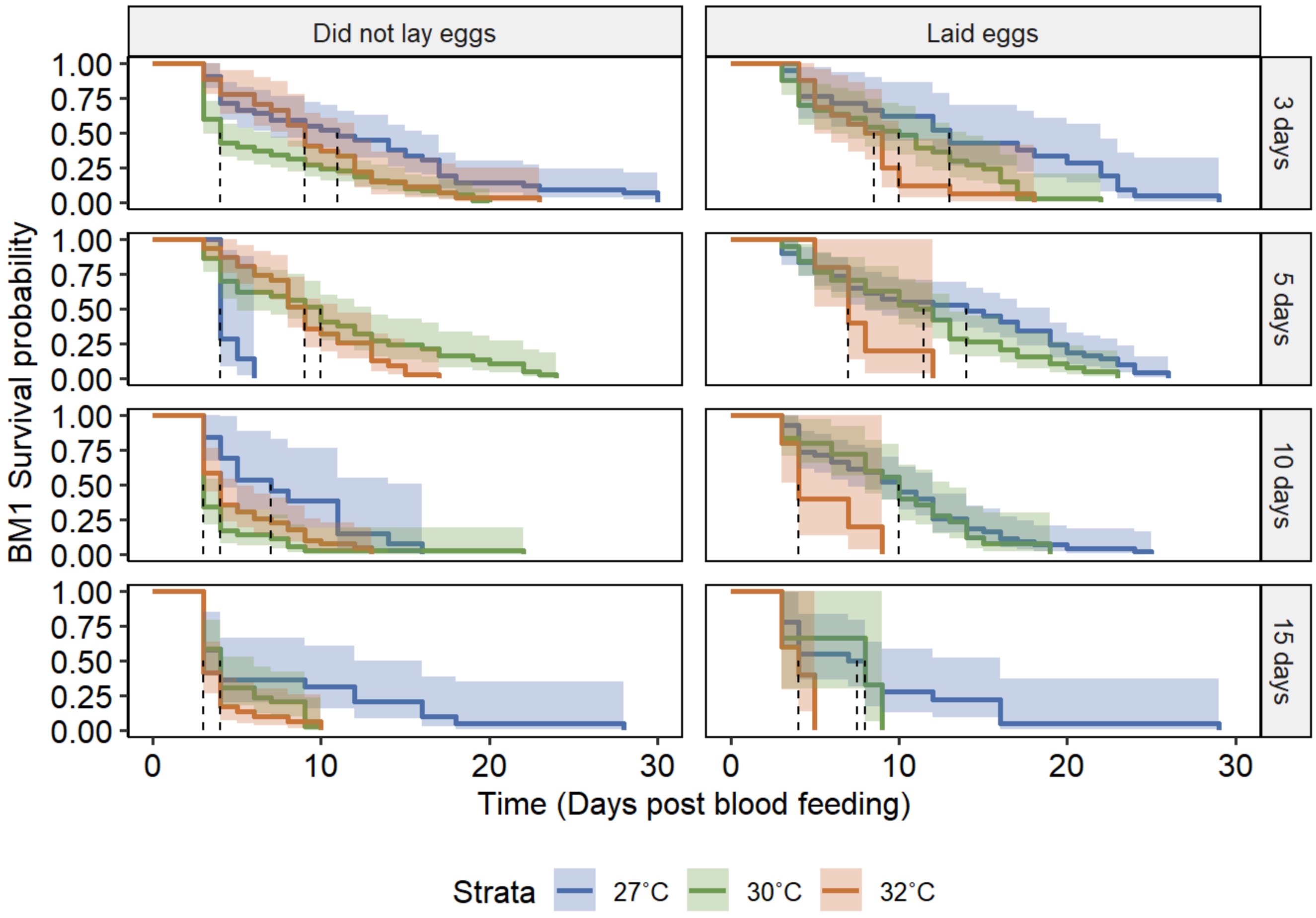


```
ggsave("Oviposition_survival/firstBM_survival_ovipos_eggsvsnoeggs_flipped_stopminusstart.pdf",firstBM_ovipo
seggsvsnoeggs_flipped, width = 6, height = 5, units = "in", dpi = 600)
ggsave("Oviposition_survival/firstBM_survival_ovipos_eggsvsnoeggs_flipped_stopminusstart.png",firstBM_ovipo
seggsvsnoeggs_flipped, width = 6, height = 5, units = "in", dpi = 600)


ggsurv <- ggsurvplot(s1, conf.int = TRUE,color="egg_binary",palette = c("#39B54A","#006838"),
                     ggtheme = theme_pubr(),surv.median.line = "v",confint=TRUE)

firstBM_oviposeggsvsnoeggs_3 <- ggsurv$plot +
  theme_pubr() +
  theme(legend.position = "none")+
  facet_grid(Temperature~Age)+
  ylab(expression("BM1 Survival probability"))+
  xlab("Time (Days post blood feeding)") +
  theme(panel.background = element_rect(fill = NA, color = "black"))+
  theme(panel.spacing = unit(0.6, "lines"))
firstBM_oviposeggsvsnoeggs_3
```

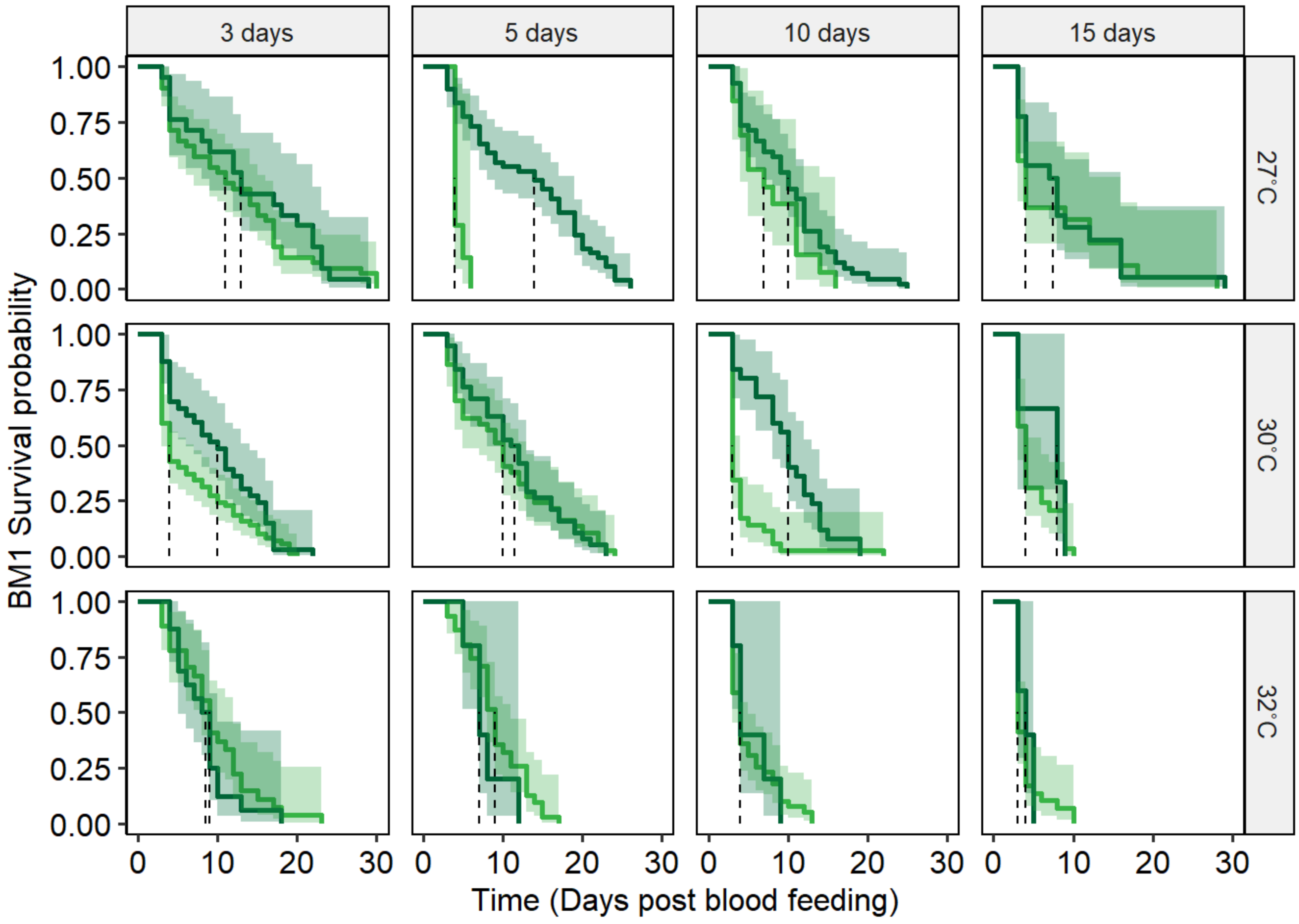


```
ggsave("Oviposition_survival/firstBM_oviposeggsvsnoeggs_3_stopminusstart.pdf",firstBM_oviposeggsvsnoeggs_3,
width = 6, height = 4, units = "in", dpi = 600)
ggsave("Oviposition_survival/firstBM_oviposeggsvsnoeggs_3_stopminusstart.png",firstBM_oviposeggsvsnoeggs_3,
width = 6, height =4, units = "in", dpi = 600)


firstBM_oviposeggsvsnoeggs_3_flip <- ggsurv$plot +
  theme_pubr() +
  theme(legend.position = "bottom")+
  facet_grid(Age~Temperature)+
  ylab(expression("BM1 Survival probability"))+
  xlab("Time (Days post blood feeding)") +
  theme(panel.background = element_rect(fill = NA, color = "black"))+
  theme(panel.spacing = unit(0.6, "lines"))
firstBM_oviposeggsvsnoeggs_3_flip
```

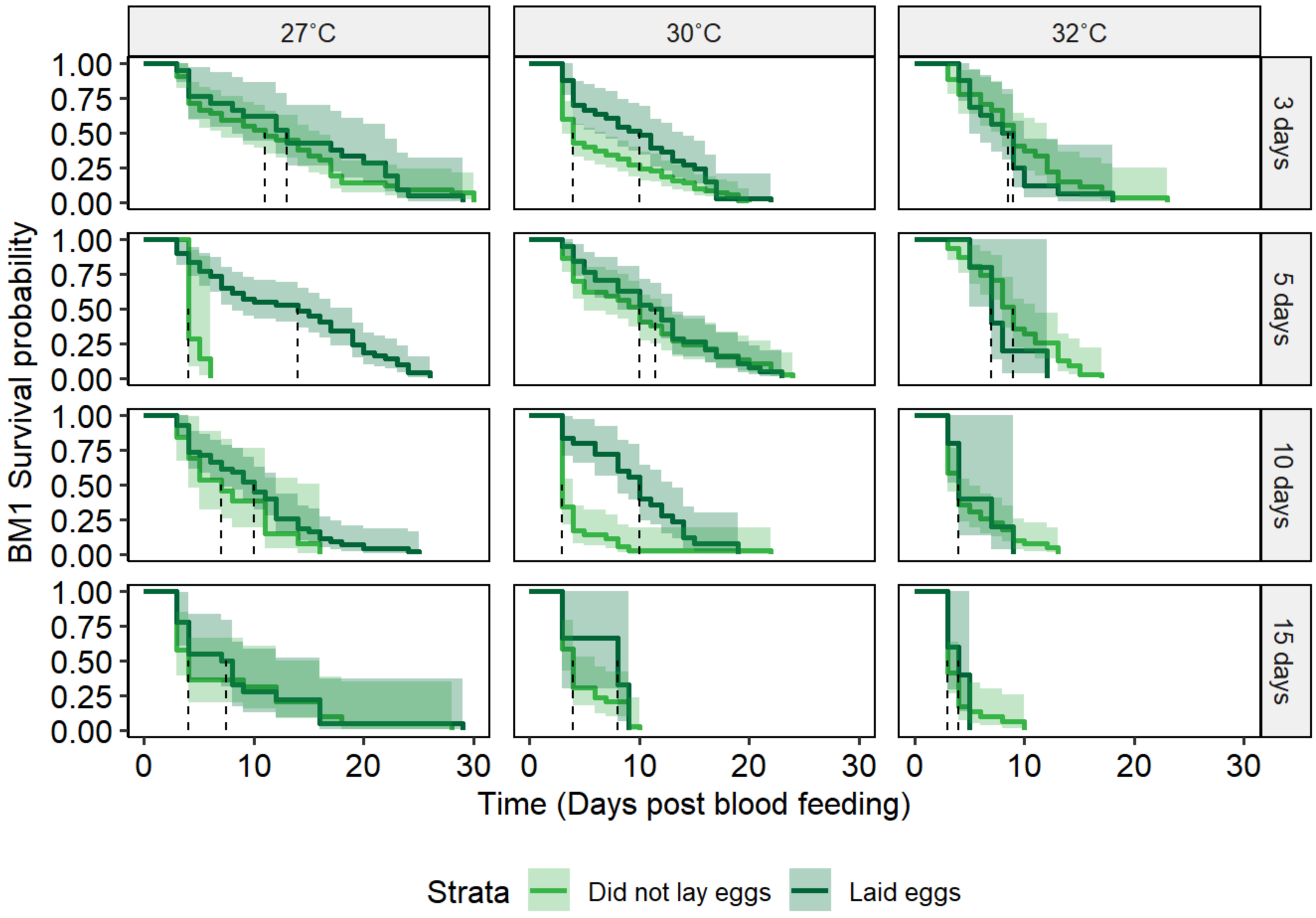


```
ggsave("Oviposition_survival/firstBM_oviposeggsvsnoeggs_3_flip_stopminusstart.pdf",firstBM_oviposeggsvsnoeg
gs_3_flip, width = 6.5, height = 5, units = "in", dpi = 600)
ggsave("Oviposition_survival/firstBM_oviposeggsvsnoeggs_3_flip_stopminusstart.png",firstBM_oviposeggsvsnoeg
gs_3_flip, width = 6.5, height = 5, units = "in", dpi = 600)


s2 <- survfit2(Surv(stopminusstart, Censor) ~ Temperature+egg_binary,
               data = Fecundity_data_firstBM_ovipossurvival)

ggsurv <- ggsurvplot(s2, conf.int = TRUE,color="Temperature",palette = c("#4D6FAE","#6F9F51", "#CC763B"),
                     ggtheme = theme_pubr(),surv.median.line = "v",confint=TRUE)
firstBM_oviposeggsvsnoeggs_temp <- ggsurv$plot +
  theme_pubr() +
  theme(legend.position = "bottom")+
  facet_grid(~egg_binary)+
  ylab(expression("BM1 Survival probability"))+
  xlab("Time (Days post blood feeding)") +
  theme(panel.background = element_rect(fill = NA, color = "black"))+
  theme(panel.spacing = unit(0.6, "lines"))
firstBM_oviposeggsvsnoeggs_temp
```

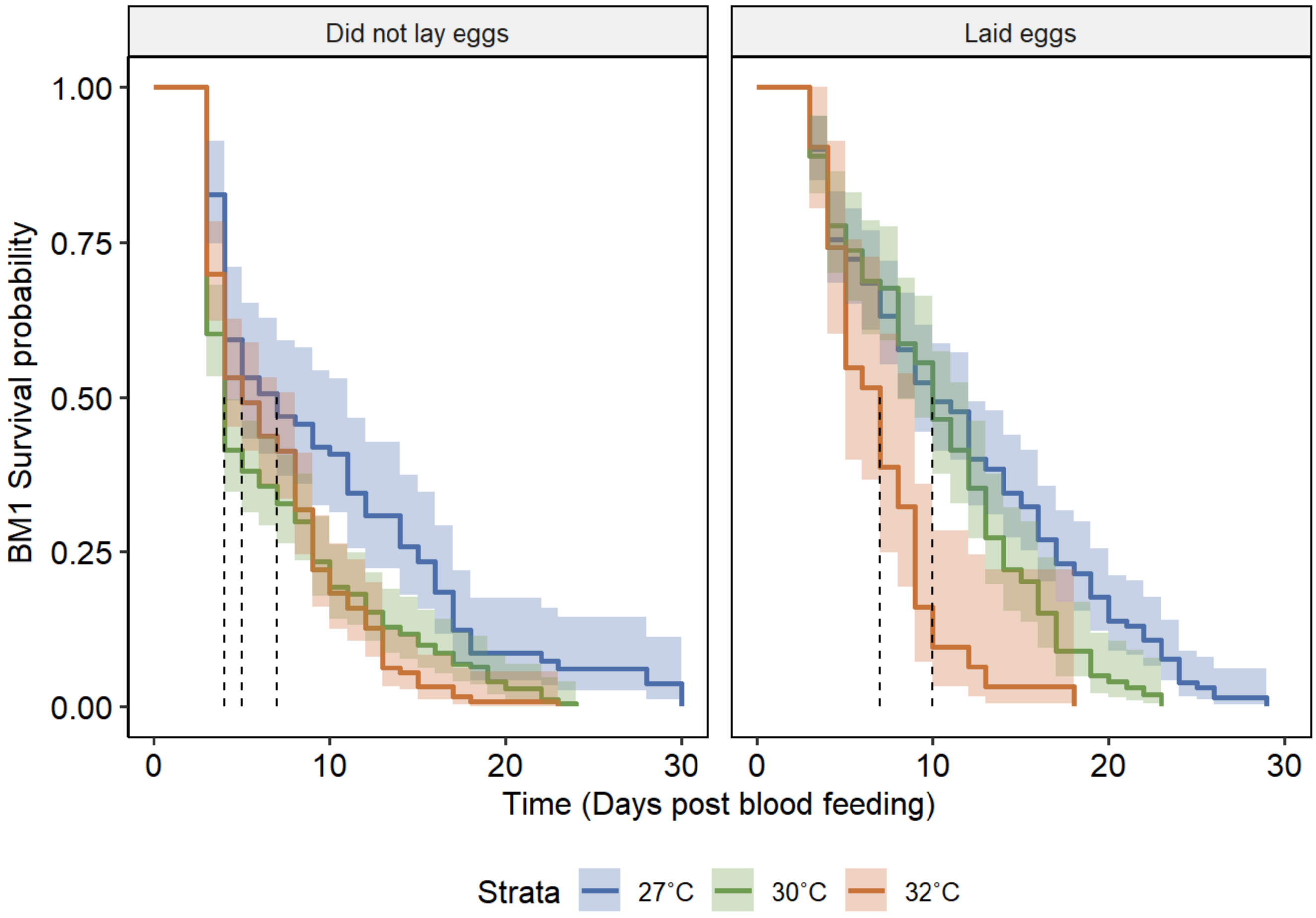


```
ggsave("Oviposition_survival/firstBM_oviposeggsvsnoeggs_temp_stopminusstart.pdf",firstBM_oviposeggsvsnoeggs_temp,width=6,height=5,units="in",dpi=600)
ggsave("Oviposition_survival/firstBM_oviposeggsvsnoeggs_temp_stopminusstart.png",firstBM_oviposeggsvsnoeggs_temp,width=6,height=5,units="in",dpi=600)

ggsurv <- ggsurvplot(s2, conf.int = TRUE,color="egg_binary",palette = "Paired",
                     ggtheme = theme_pubr(),surv.median.line = "v",confint=TRUE)

firstBM_oviposeggsvsnoeggs_temp_flip <- ggsurv$plot +
  theme_pubr() +
  theme(legend.position = "bottom")+
  facet_grid(~Temperature)+
  ylab(expression("BM1 Survival probability"))+
  xlab("Time (Days post blood feeding)") +
  theme(panel.background = element_rect(fill = NA, color = "black"))+
  theme(panel.spacing = unit(0.6, "lines"))
firstBM_oviposeggsvsnoeggs_temp_flip
```

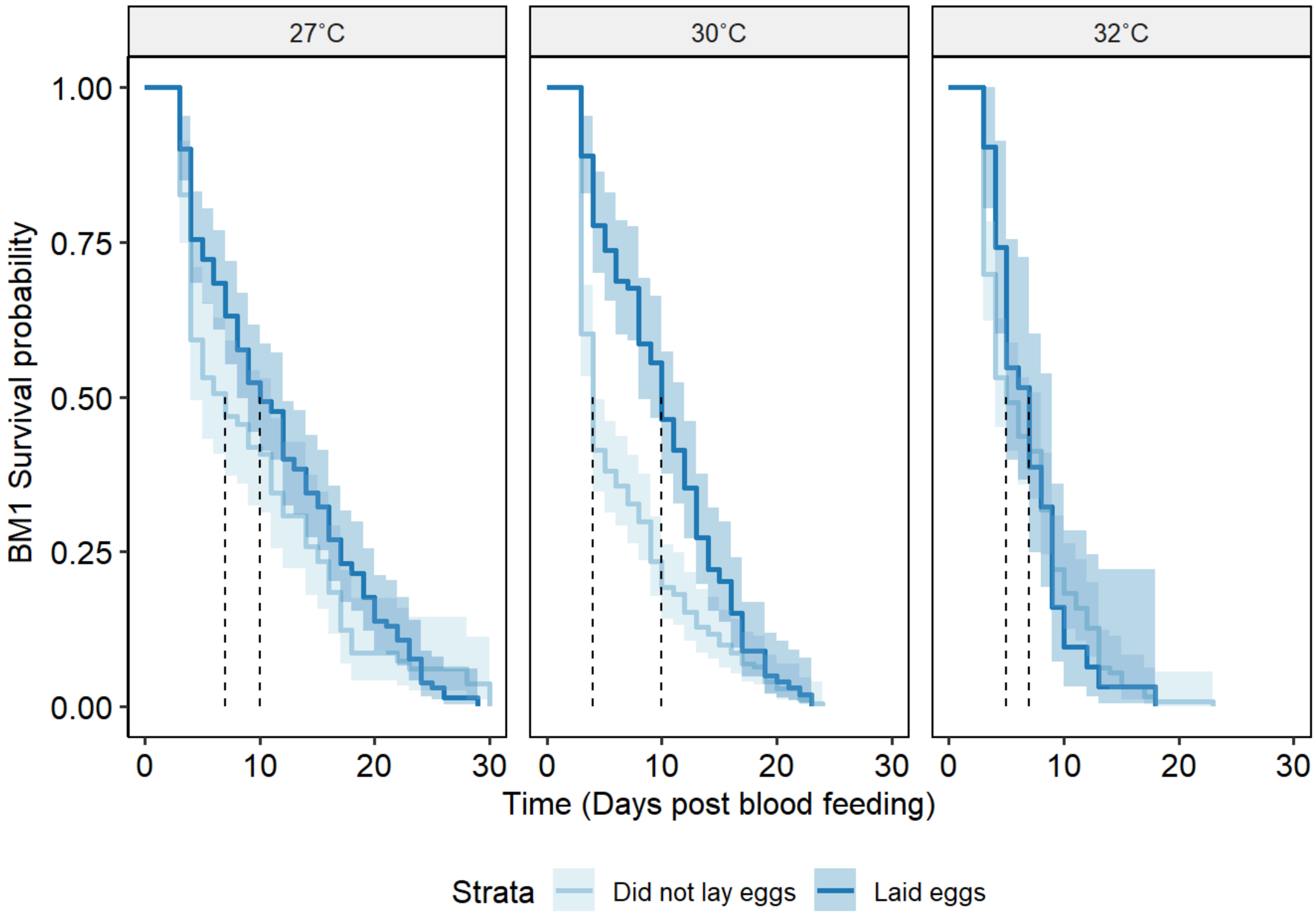


```
ggsave("Oviposition_survival/firstBM_oviposeggsvsnoeggs_temp_flip_stopminusstart.pdf",firstBM_oviposeggsvsn
oeggs_temp_flip,width=6,height=5,units="in",dpi=600)
ggsave("Oviposition_survival/firstBM_oviposeggsvsnoeggs_temp_flip_stopminusstart.png",firstBM_oviposeggsvsn
oeggs_temp_flip,width=6,height=5,units="in",dpi=600)

s3 <- survfit2(Surv(stopminusstart, Censor) ~ Age+egg_binary,
               data = Fecundity_data_firstBM_ovipossurvival)

ggsurv <- ggsurvplot(s3, conf.int = TRUE,color="Age",palette = c("#DCD1E9","#BAA4D3","#9776BE","#7549A8"),
                     ggtheme = theme_pubr(),surv.median.line = "v",confint=TRUE)

firstBM_oviposeggsvsnoeggs_age <- ggsurv$plot +
  theme_pubr() +
  theme(legend.position = "bottom")+
  facet_grid(~egg_binary)+
  ylab(expression("BM1 Survival probability"))+
  xlab("Time (Days post blood feeding)") +
  theme(panel.background = element_rect(fill = NA, color = "black"))+
  theme(panel.spacing = unit(0.6, "lines"))

firstBM_oviposeggsvsnoeggs_age
```

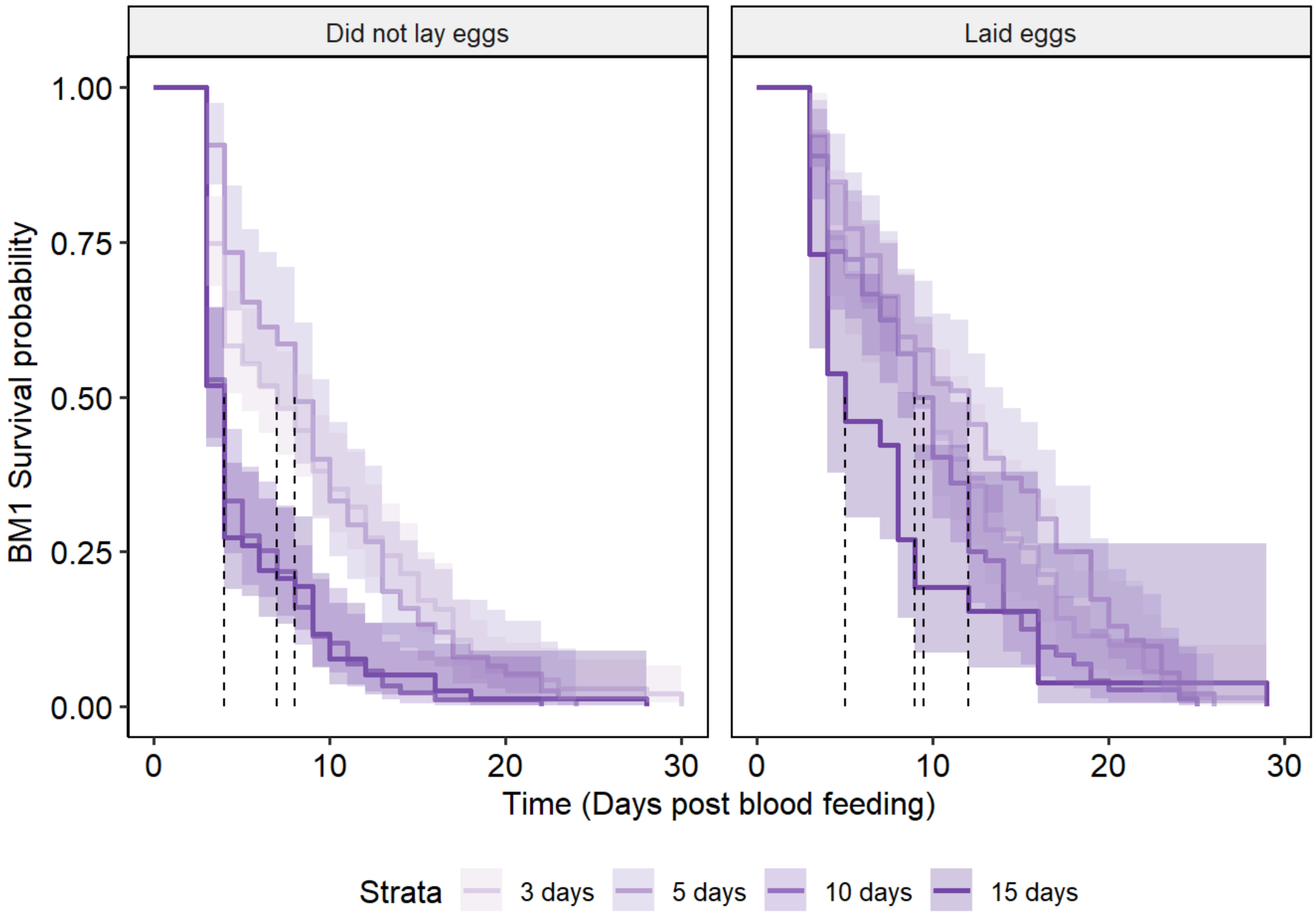


```
ggsave("Oviposition_survival/firstBM_oviposeggsvsnoeggs_age_stopminusstart.pdf",firstBM_oviposeggsvsnoeggs_
age,width=6,height=5,units="in",dpi=600)
ggsave("Oviposition_survival/firstBM_oviposeggsvsnoeggs_age_stopminusstart.png",firstBM_oviposeggsvsnoeggs_
age,width=6,height=5,units="in",dpi=600)

ggsurv <- ggsurvplot(s3, conf.int = TRUE,color="egg_binary",palette ="Paired",
                     ggtheme = theme_pubr(),surv.median.line = "v",confint=TRUE)
firstBM_oviposeggsvsnoeggs_age_flip <- ggsurv$plot +
  theme_pubr() +
  theme(legend.position = "bottom")+
  facet_grid(~Age)+
  ylab(expression("BM1 Survival probability"))+
  xlab("Time (Days post blood feeding)") +
  theme(panel.background = element_rect(fill = NA, color = "black"))+
  theme(panel.spacing = unit(0.6, "lines"))

firstBM_oviposeggsvsnoeggs_age_flip
```

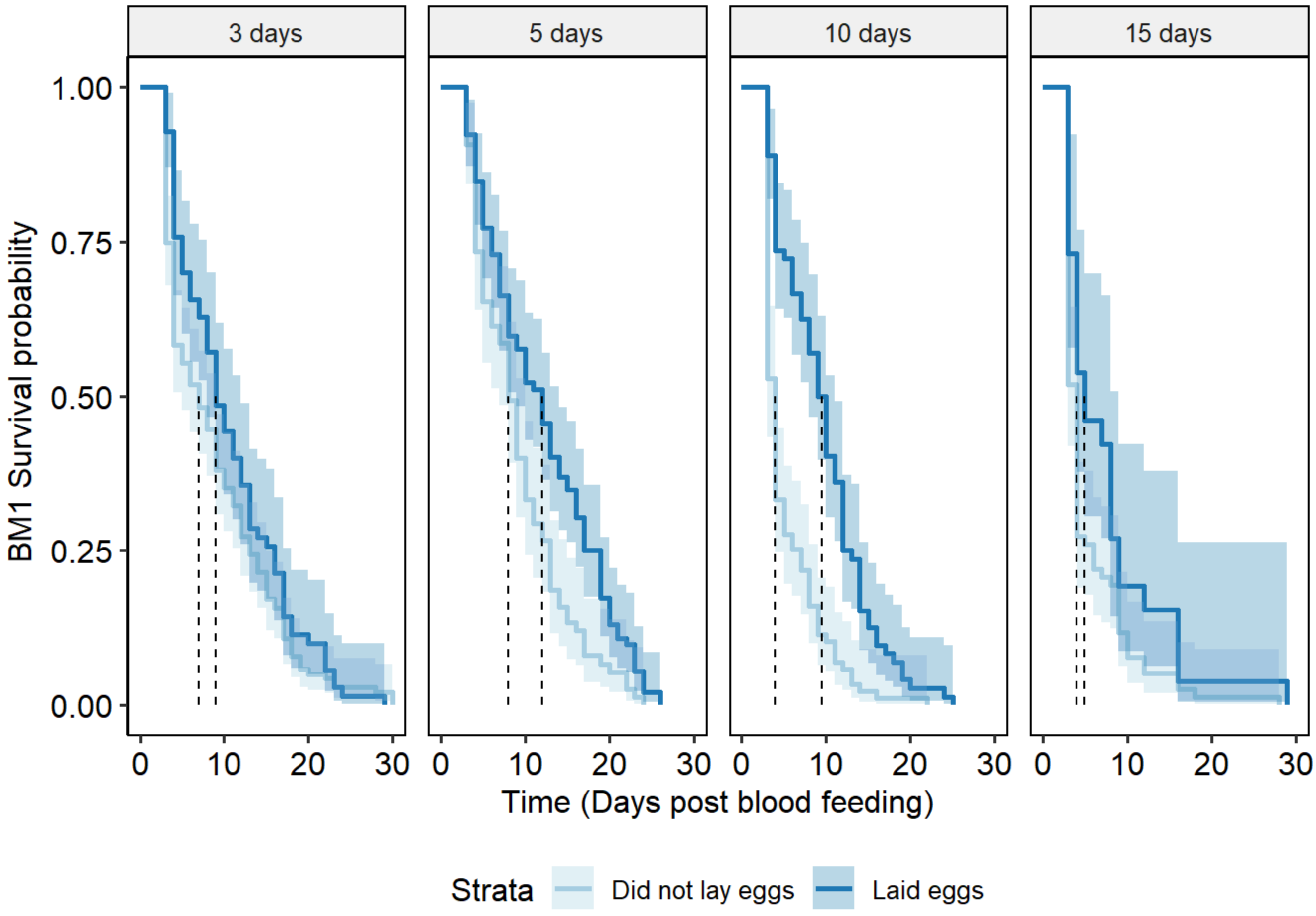


```
ggsave("Oviposition_survival/firstBM_oviposeggsvsnoeggs_age_flip_stopminusstart.pdf",firstBM_oviposeggsvsnoeggs_age_flip,width=6,height=5,units="in",dpi=600)
ggsave("Oviposition_survival/firstBM_oviposeggsvsnoeggs_age_flip_stopminusstart.png",firstBM_oviposeggsvsnoeggs_age_flip,width=6,height=5,units="in",dpi=600)

s4 <- survfit2(Surv(stopminusstart, Censor) ~ egg_binary,
               data = Fecundity_data_firstBM_ovipossurvival)

ggsurv <- ggsurvplot(s4, conf.int = TRUE,color="egg_binary",palette = c("#39B54A","#006838"),
                     ggtheme = theme_pubr(),surv.median.line = "v",confint=TRUE,
                     risk.table = TRUE)

firstBM_oviposeggsvsnoeggs_eggsonly <- ggsurv$plot +
  theme_pubr() +
  theme(legend.position = "bottom")+
  # facet_grid(~egg_binary)+
  ylab(expression("BM1 Survival probability"))+
  xlab("Time (Days post blood feeding)") +
  theme(panel.background = element_rect(fill = NA, color = "black"))+
  theme(panel.spacing = unit(0.6, "lines"))
firstBM_oviposeggsvsnoeggs_eggsonly
```

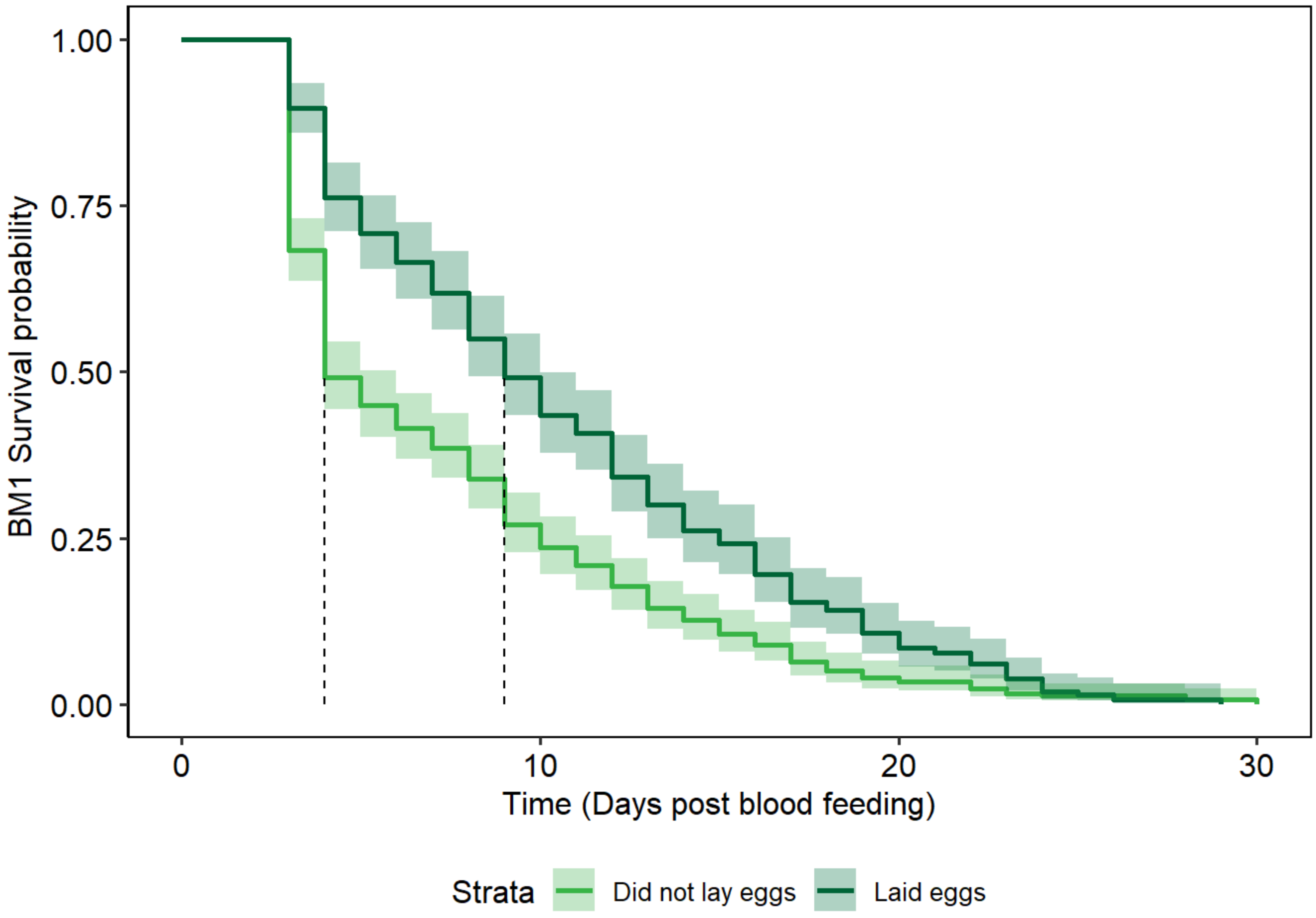


```
ggsave("Oviposition_survival/firstBM_oviposeggsvsnoeggs_eggsonly_stopminusstart.pdf",firstBM_oviposeggsvsno
eggs_eggsonly,width=5,height=5,units="in",dpi=600)
ggsave("Oviposition_survival/firstBM_oviposeggsvsnoeggs_eggsonly_stopminusstart.png",firstBM_oviposeggsvsno
eggs_eggsonly,width=5,height=5,units="in",dpi=600)


#save median survival times:
sink("Oviposition_survival/firstBM_eggbinary1_survival_mediansurvivaltime_stopminusstart.txt")
print(s1,print.rmean = TRUE)
sink()

sink("Oviposition_survival/firstBM_eggbinary2_survival_mediansurvivaltime_stopminusstart.txt")
print(s2,print.rmean = TRUE)
sink()

sink("Oviposition_survival/firstBM_eggbinary3_survival_mediansurvivaltime_stopminusstart.txt")
print(s3,print.rmean = TRUE)
sink()

sink("Oviposition_survival/firstBM_eggbinary4_survival_mediansurvivaltime_stopminusstart.txt")
print(s4,print.rmean = TRUE)
sink()
```

Analysis for hazard ratios and making forest plot

```
#ANALYSIS - HAZARD RATIOS
#switch back to numbers:
str(Fecundity_data_firstBM_ovipossurvival)
```

```
## 'data.frame':	682 obs. of  37 variables:
##  $ ID_overall               : num  1 2 3 4 5 6 7 8 11 12 ...
##  $ Temperature              : Factor w/ 3 levels "27˚C","30˚C",..: 3 3 3 3 3 3 3 3 1 1 ...
##  $ Age                      : Factor w/ 4 levels "3 days","5 days",..: 1 1 1 1 1 1 1 1 1 1 ...
##  $ ID_per_group             : num  1 2 3 4 5 6 7 8 1 2 ...
##  $ Trial_start_date         : POSIXct, format: "2024-02-13" "2024-02-13" ...
##  $ Trial_number             : num  1 1 1 1 1 1 1 1 1 1 ...
##  $ BM1_Date                 : POSIXct, format: "2024-02-13" "2024-02-13" ...
##  $ Age_of_BM                : num  3 3 3 3 3 3 3 3 3 3 ...
##  $ Bloodmeal_number         : Factor w/ 1 level "1": 1 1 1 1 1 1 1 1 1 1 ...
##  $ Oviposition_positive(y/n): chr  "Y" "Y" "Y" "Y" ...
##  $ Eggs_day3                : num  0 0 0 0 0 14 NA NA 0 72 ...
##  $ Eggs_day4                : num  0 0 0 0 0 0 NA NA 0 10 ...
##  $ Larvae_day4              : num  0 0 0 0 0 0 0 0 NA 0 ...
##  $ Surv_to_eggs(y/n)        : Factor w/ 3 levels "N","NA","Y": 3 3 3 3 3 3 1 1 3 3 ...
##  $ Surv_to_larvae(y/n)      : Factor w/ 3 levels "N","NA","Y": 3 3 3 3 3 3 2 2 3 3 ...
##  $ Date_of_death            : POSIXct, format: "2024-03-01" "2024-03-07" ...
##  $ Censor                   : num  1 1 1 1 1 1 1 1 1 1 ...
##  $ Notes                    : chr  NA NA NA NA ...
##  $ Oviposition_positive     : Factor w/ 2 levels "N","Y": 2 2 2 2 2 2 2 2 2 2 ...
##  $ total_eggs               : num  0 0 0 0 0 14 NA NA 0 82 ...
##  $ Percent_eggs_day3        : num  NaN NaN NaN NaN NaN ...
##  $ Percent_eggs_day4        : num  NaN NaN NaN NaN NaN ...
##  $ egg_binary               : Factor w/ 2 levels "Did not lay eggs",..: 1 1 1 1 1 2 NA NA 1 2 ...
##  $ egg_binary_day3          : num  0 0 0 0 0 1 NA NA 0 1 ...
##  $ egg_binary_day4          : num  0 0 0 0 0 0 NA NA 0 1 ...
##  $ larvae_binary            : num  0 0 0 0 0 0 0 0 NA 0 ...
##  $ Percent_eggshatchedtolarv: num  NaN NaN NaN NaN NaN 0 NA NA NA 0 ...
##  $ Days_to_death_post_BM    : 'difftime' num  17 23 18 13 ...
##   ..- attr(*, "units")= chr "days"
##  $ Age_of_death             : 'difftime' num  20 26 21 16 ...
##   ..- attr(*, "units")= chr "days"
##  $ Age_of_BM_days           : 'difftime' num  3 3 3 3 ...
##   ..- attr(*, "units")= chr "days"
##  $ Date_of_eclosion         : POSIXct, format: "2024-02-10" "2024-02-10" ...
##  $ days_alive_post_BM       : num  17 23 18 13 15 18 3 3 12 12 ...
##  $ days_alive_post_eclosion : num  20 26 21 16 18 21 6 6 15 15 ...
##  $ group                    : chr  "3_32" "3_32" "3_32" "3_32" ...
##  $ start_time               : num  4 4 4 4 4 4 4 4 4 4 ...
##  $ stop_time                : num  21 27 22 17 19 22 7 7 16 16 ...
##  $ stopminusstart           : num  17 23 18 13 15 18 3 3 12 12 ...
```

```
Fecundity_data_firstBM_ovipossurvival$Temperature <- factor(Fecundity_data_firstBM_ovipossurvival$Temperature,
                                                            labels = c("27","30","32"))
Fecundity_data_firstBM_ovipossurvival$Age <- factor(Fecundity_data_firstBM_ovipossurvival$Age,
                                                    labels = c("3","5","10","15"))

Fecundity_data_firstBM_ovipossurvival_numeric <- Fecundity_data_firstBM_ovipossurvival

str(Fecundity_data_firstBM_ovipossurvival_numeric)
```

```
## 'data.frame':    682 obs. of  37 variables:
##  $ ID_overall               : num  1 2 3 4 5 6 7 8 11 12 ...
##  $ Temperature              : Factor w/ 3 levels "27","30","32": 3 3 3 3 3 3 3 3 1 1 ...
##  $ Age                      : Factor w/ 4 levels "3","5","10","15": 1 1 1 1 1 1 1 1 1 1 ...
##  $ ID_per_group             : num  1 2 3 4 5 6 7 8 1 2 ...
##  $ Trial_start_date         : POSIXct, format: "2024-02-13" "2024-02-13" ...
##  $ Trial_number             : num  1 1 1 1 1 1 1 1 1 1 ...
##  $ BM1_Date                 : POSIXct, format: "2024-02-13" "2024-02-13" ...
##  $ Age_of_BM                : num  3 3 3 3 3 3 3 3 3 3 ...
##  $ Bloodmeal_number         : Factor w/ 1 level "1": 1 1 1 1 1 1 1 1 1 1 ...
##  $ Oviposition_positive(y/n): chr  "Y" "Y" "Y" "Y" ...
##  $ Eggs_day3                : num  0 0 0 0 0 14 NA NA 0 72 ...
##  $ Eggs_day4                : num  0 0 0 0 0 0 NA NA 0 10 ...
##  $ Larvae_day4              : num  0 0 0 0 0 0 0 0 NA 0 ...
##  $ Surv_to_eggs(y/n)        : Factor w/ 3 levels "N","NA","Y": 3 3 3 3 3 3 1 1 3 3 ...
##  $ Surv_to_larvae(y/n)      : Factor w/ 3 levels "N","NA","Y": 3 3 3 3 3 3 2 2 3 3 ...
##  $ Date_of_death            : POSIXct, format: "2024-03-01" "2024-03-07" ...
##  $ Censor                   : num  1 1 1 1 1 1 1 1 1 1 ...
##  $ Notes                    : chr  NA NA NA NA ...
##  $ Oviposition_positive     : Factor w/ 2 levels "N","Y": 2 2 2 2 2 2 2 2 2 2 ...
##  $ total_eggs               : num  0 0 0 0 0 14 NA NA 0 82 ...
##  $ Percent_eggs_day3        : num  NaN NaN NaN NaN NaN ...
##  $ Percent_eggs_day4        : num  NaN NaN NaN NaN NaN ...
##  $ egg_binary               : Factor w/ 2 levels "Did not lay eggs",..: 1 1 1 1 1 2 NA NA 1 2 ...
##  $ egg_binary_day3          : num  0 0 0 0 0 1 NA NA 0 1 ...
##  $ egg_binary_day4          : num  0 0 0 0 0 0 NA NA 0 1 ...
##  $ larvae_binary            : num  0 0 0 0 0 0 0 0 NA 0 ...
##  $ Percent_eggshatchedtolarv: num  NaN NaN NaN NaN NaN 0 NA NA NA 0 ...
##  $ Days_to_death_post_BM    : 'difftime' num  17 23 18 13 ...
##   ..- attr(*, "units")= chr "days"
##  $ Age_of_death             : 'difftime' num  20 26 21 16 ...
##   ..- attr(*, "units")= chr "days"
##  $ Age_of_BM_days           : 'difftime' num  3 3 3 3 ...
##   ..- attr(*, "units")= chr "days"
##  $ Date_of_eclosion         : POSIXct, format: "2024-02-10" "2024-02-10" ...
##  $ days_alive_post_BM       : num  17 23 18 13 15 18 3 3 12 12 ...
##  $ days_alive_post_eclosion : num  20 26 21 16 18 21 6 6 15 15 ...
##  $ group                    : chr  "3_32" "3_32" "3_32" "3_32" ...
##  $ start_time               : num  4 4 4 4 4 4 4 4 4 4 ...
##  $ stop_time                : num  21 27 22 17 19 22 7 7 16 16 ...
##  $ stopminusstart           : num  17 23 18 13 15 18 3 3 12 12 ...
```

```
#Compare hazard ratios:
#treat variables as numeric so hazard shows with every increase in age(days) or temp (degrees)
Fecundity_data_firstBM_ovipossurvival_numeric$Temperature <- as.character(Fecundity_data_firstBM_ovipossurv
ival_numeric$Temperature)
str(Fecundity_data_firstBM_ovipossurvival_numeric$Temperature)
```

```
##  chr [1:682] "32" "32" "32" "32" "32" "32" "32" "32" "27" "27" "27" "27" ...
```

```
Fecundity_data_firstBM_ovipossurvival_numeric$Temperature <- as.numeric(Fecundity_data_firstBM_ovipossurviv
al_numeric$Temperature)
Fecundity_data_firstBM_ovipossurvival_numeric$Age <- as.character(Fecundity_data_firstBM_ovipossurvival_num
eric$Age)
str(Fecundity_data_firstBM_ovipossurvival_numeric$Age)
```

```
##  chr [1:682] "3" "3" "3" "3" "3" "3" "3" "3" "3" "3" "3" "3" "3" "3" "3" ...
```

```
Fecundity_data_firstBM_ovipossurvival_numeric$Age <- as.numeric(Fecundity_data_firstBM_ovipossurvival_numer
ic$Age)



Fecundity_data_firstBM_ovipossurvival_numeric$Trial_number <- as.factor(Fecundity_data_firstBM_ovipossurviv
al_numeric$Trial_number)

Fecundity_data_firstBM_ovipossurvival_numeric$Trial_start_date_factor <- as.factor(Fecundity_data_firstBM_o
vipossurvival_numeric$Trial_start_date)
str(Fecundity_data_firstBM_ovipossurvival_numeric$Trial_start_date_factor)
```

```
##  Factor w/ 32 levels "2024-02-13","2024-02-19",..: 1 1 1 1 1 1 1 1 20 20 ...
```

```
str(Fecundity_data_firstBM_ovipossurvival_numeric)
```

```
## 'data.frame':    682 obs. of  38 variables:
##  $ ID_overall               : num  1 2 3 4 5 6 7 8 11 12 ...
##  $ Temperature              : num  32 32 32 32 32 32 32 32 27 27 ...
##  $ Age                      : num  3 3 3 3 3 3 3 3 3 3 ...
##  $ ID_per_group             : num  1 2 3 4 5 6 7 8 1 2 ...
##  $ Trial_start_date         : POSIXct, format: "2024-02-13" "2024-02-13" ...
##  $ Trial_number             : Factor w/ 5 levels "1","2","3","4",..: 1 1 1 1 1 1 1 1 1 1 ...
##  $ BM1_Date                 : POSIXct, format: "2024-02-13" "2024-02-13" ...
##  $ Age_of_BM                : num  3 3 3 3 3 3 3 3 3 3 ...
##  $ Bloodmeal_number         : Factor w/ 1 level "1": 1 1 1 1 1 1 1 1 1 1 ...
##  $ Oviposition_positive(y/n): chr  "Y" "Y" "Y" "Y" ...
##  $ Eggs_day3                : num  0 0 0 0 0 14 NA NA 0 72 ...
##  $ Eggs_day4                : num  0 0 0 0 0 0 NA NA 0 10 ...
##  $ Larvae_day4              : num  0 0 0 0 0 0 0 0 NA 0 ...
##  $ Surv_to_eggs(y/n)        : Factor w/ 3 levels "N","NA","Y": 3 3 3 3 3 3 1 1 3 3 ...
##  $ Surv_to_larvae(y/n)      : Factor w/ 3 levels "N","NA","Y": 3 3 3 3 3 3 2 2 3 3 ...
##  $ Date_of_death            : POSIXct, format: "2024-03-01" "2024-03-07" ...
##  $ Censor                   : num  1 1 1 1 1 1 1 1 1 1 ...
##  $ Notes                    : chr  NA NA NA NA ...
##  $ Oviposition_positive     : Factor w/ 2 levels "N","Y": 2 2 2 2 2 2 2 2 2 2 ...
##  $ total_eggs               : num  0 0 0 0 0 14 NA NA 0 82 ...
##  $ Percent_eggs_day3        : num  NaN NaN NaN NaN NaN ...
##  $ Percent_eggs_day4        : num  NaN NaN NaN NaN NaN ...
##  $ egg_binary               : Factor w/ 2 levels "Did not lay eggs",..: 1 1 1 1 1 2 NA NA 1 2 ...
##  $ egg_binary_day3          : num  0 0 0 0 0 1 NA NA 0 1 ...
##  $ egg_binary_day4          : num  0 0 0 0 0 0 NA NA 0 1 ...
##  $ larvae_binary            : num  0 0 0 0 0 0 0 0 NA 0 ...
##  $ Percent_eggshatchedtolarv: num  NaN NaN NaN NaN NaN 0 NA NA NA 0 ...
##  $ Days_to_death_post_BM    : 'difftime' num  17 23 18 13 ...
##   ..- attr(*, "units")= chr "days"
##  $ Age_of_death             : 'difftime' num  20 26 21 16 ...
##   ..- attr(*, "units")= chr "days"
##  $ Age_of_BM_days           : 'difftime' num  3 3 3 3 ...
##   ..- attr(*, "units")= chr "days"
##  $ Date_of_eclosion         : POSIXct, format: "2024-02-10" "2024-02-10" ...
##  $ days_alive_post_BM       : num  17 23 18 13 15 18 3 3 12 12 ...
##  $ days_alive_post_eclosion : num  20 26 21 16 18 21 6 6 15 15 ...
##  $ group                    : chr  "3_32" "3_32" "3_32" "3_32" ...
##  $ start_time               : num  4 4 4 4 4 4 4 4 4 4 ...
##  $ stop_time                : num  21 27 22 17 19 22 7 7 16 16 ...
##  $ stopminusstart           : num  17 23 18 13 15 18 3 3 12 12 ...
##  $ Trial_start_date_factor  : Factor w/ 32 levels "2024-02-13","2024-02-19",..: 1 1 1 1 1 1 1 1 20 20
...
```

```
Fecundity_data_firstBM_ovipossurvival_numeric$Trial_start_date_number <- as.numeric(Fecundity_data_firstBM_
ovipossurvival_numeric$Trial_start_date_factor)
str(Fecundity_data_firstBM_ovipossurvival_numeric)
```

```
## 'data.frame':	682 obs. of  39 variables:
##  $ ID_overall               : num  1 2 3 4 5 6 7 8 11 12 ...
##  $ Temperature              : num  32 32 32 32 32 32 32 32 27 27 ...
##  $ Age                      : num  3 3 3 3 3 3 3 3 3 3 ...
##  $ ID_per_group             : num  1 2 3 4 5 6 7 8 1 2 ...
##  $ Trial_start_date         : POSIXct, format: "2024-02-13" "2024-02-13" ...
##  $ Trial_number             : Factor w/ 5 levels "1","2","3","4",..: 1 1 1 1 1 1 1 1 1 1 ...
##  $ BM1_Date                 : POSIXct, format: "2024-02-13" "2024-02-13" ...
##  $ Age_of_BM                : num  3 3 3 3 3 3 3 3 3 3 ...
##  $ Bloodmeal_number         : Factor w/ 1 level "1": 1 1 1 1 1 1 1 1 1 1 ...
##  $ Oviposition_positive(y/n): chr  "Y" "Y" "Y" "Y" ...
##  $ Eggs_day3                : num  0 0 0 0 0 14 NA NA 0 72 ...
##  $ Eggs_day4                : num  0 0 0 0 0 0 NA NA 0 10 ...
##  $ Larvae_day4              : num  0 0 0 0 0 0 0 0 NA 0 ...
##  $ Surv_to_eggs(y/n)        : Factor w/ 3 levels "N","NA","Y": 3 3 3 3 3 3 1 1 3 3 ...
##  $ Surv_to_larvae(y/n)      : Factor w/ 3 levels "N","NA","Y": 3 3 3 3 3 3 2 2 3 3 ...
##  $ Date_of_death            : POSIXct, format: "2024-03-01" "2024-03-07" ...
##  $ Censor                   : num  1 1 1 1 1 1 1 1 1 1 ...
##  $ Notes                    : chr  NA NA NA NA ...
##  $ Oviposition_positive     : Factor w/ 2 levels "N","Y": 2 2 2 2 2 2 2 2 2 2 ...
##  $ total_eggs               : num  0 0 0 0 0 14 NA NA 0 82 ...
##  $ Percent_eggs_day3        : num  NaN NaN NaN NaN NaN ...
##  $ Percent_eggs_day4        : num  NaN NaN NaN NaN NaN ...
##  $ egg_binary               : Factor w/ 2 levels "Did not lay eggs",..: 1 1 1 1 1 2 NA NA 1 2 ...
##  $ egg_binary_day3          : num  0 0 0 0 0 1 NA NA 0 1 ...
##  $ egg_binary_day4          : num  0 0 0 0 0 0 NA NA 0 1 ...
##  $ larvae_binary            : num  0 0 0 0 0 0 0 0 NA 0 ...
##  $ Percent_eggshatchedtolarv: num  NaN NaN NaN NaN NaN 0 NA NA NA 0 ...
##  $ Days_to_death_post_BM    : 'difftime' num  17 23 18 13 ...
##   ..- attr(*, "units")= chr "days"
##  $ Age_of_death             : 'difftime' num  20 26 21 16 ...
##   ..- attr(*, "units")= chr "days"
##  $ Age_of_BM_days           : 'difftime' num  3 3 3 3 ...
##   ..- attr(*, "units")= chr "days"
##  $ Date_of_eclosion         : POSIXct, format: "2024-02-10" "2024-02-10" ...
##  $ days_alive_post_BM       : num  17 23 18 13 15 18 3 3 12 12 ...
##  $ days_alive_post_eclosion : num  20 26 21 16 18 21 6 6 15 15 ...
##  $ group                    : chr  "3_32" "3_32" "3_32" "3_32" ...
##  $ start_time               : num  4 4 4 4 4 4 4 4 4 4 ...
##  $ stop_time                : num  21 27 22 17 19 22 7 7 16 16 ...
##  $ stopminusstart           : num  17 23 18 13 15 18 3 3 12 12 ...
##  $ Trial_start_date_factor  : Factor w/ 32 levels "2024-02-13","2024-02-19",..: 1 1 1 1 1 1 1 1 20 20
...
##  $ Trial_start_date_number  : num  1 1 1 1 1 1 1 1 20 20 ...
```

```
Fecundity_data_firstBM_ovipossurvival_numeric$Tempscaled <- scale(Fecundity_data_firstBM_ovipossurvival_num
eric$Temperature, center=TRUE, scale=TRUE)
Fecundity_data_firstBM_ovipossurvival_numeric$Agescaled <- scale(Fecundity_data_firstBM_ovipossurvival_nume
ric$Age, center=TRUE, scale=TRUE)

#check proportional hazards model
mv_fit <- coxph(Surv(stop_time, Censor) ~ Temperature*Age, data = Fecundity_data_firstBM_ovipossurvival_num
eric)
mv_fit <- coxph(Surv(stop_time, Censor) ~ Tempscaled*Agescaled, data = Fecundity_data_firstBM_ovipossurviva
l_numeric)



cz <- cox.zph(mv_fit)
print(cz) # significant p-value indicates that the proportional hazards assumption is violated
```

```
##                          chisq df      p
## Tempscaled              9.3895  1 0.0022
## Agescaled             104.1958  1 <2e-16
## Tempscaled:Agescaled    0.0645  1 0.7994
## GLOBAL                123.4583  3 <2e-16
```

```
plot(cz) #violated for age, temp*age, global - non-proportional
```

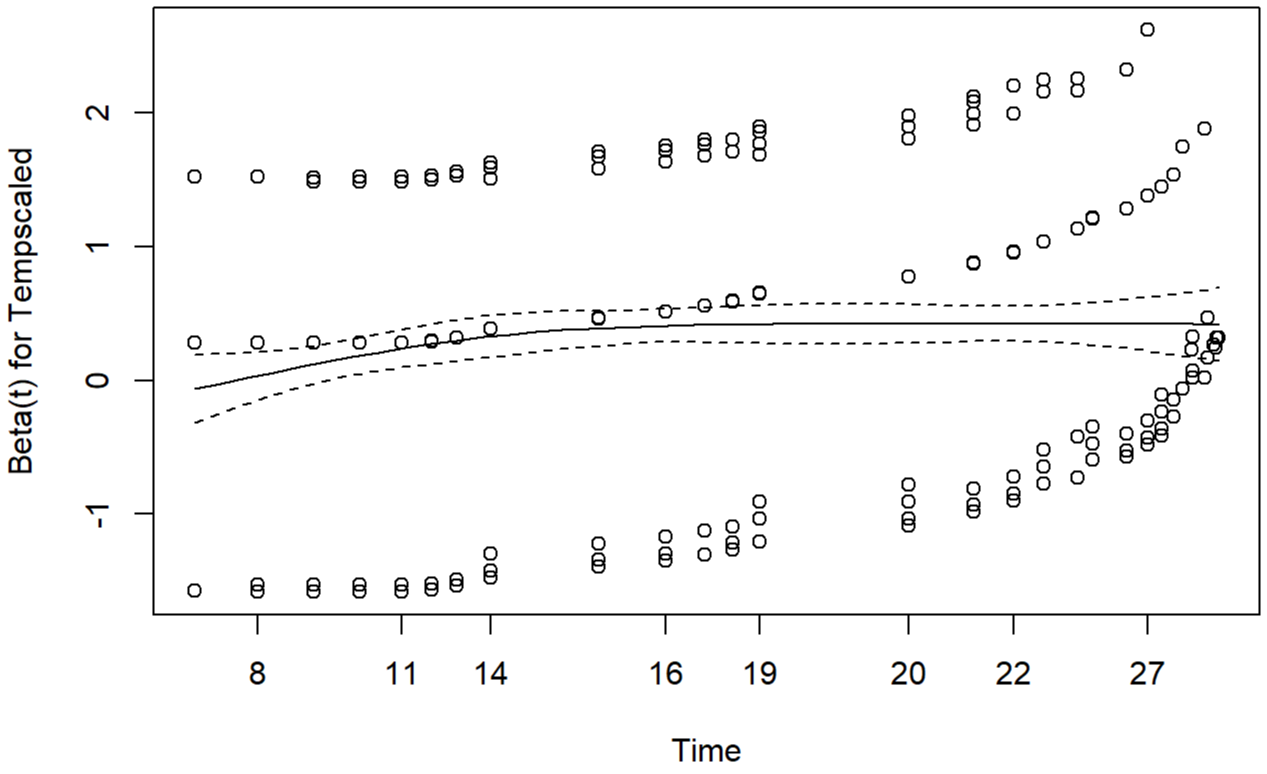
Beta(t) for Tempscaled
Time
8
11
14
16
19
20
22
27
-1
0
1
2

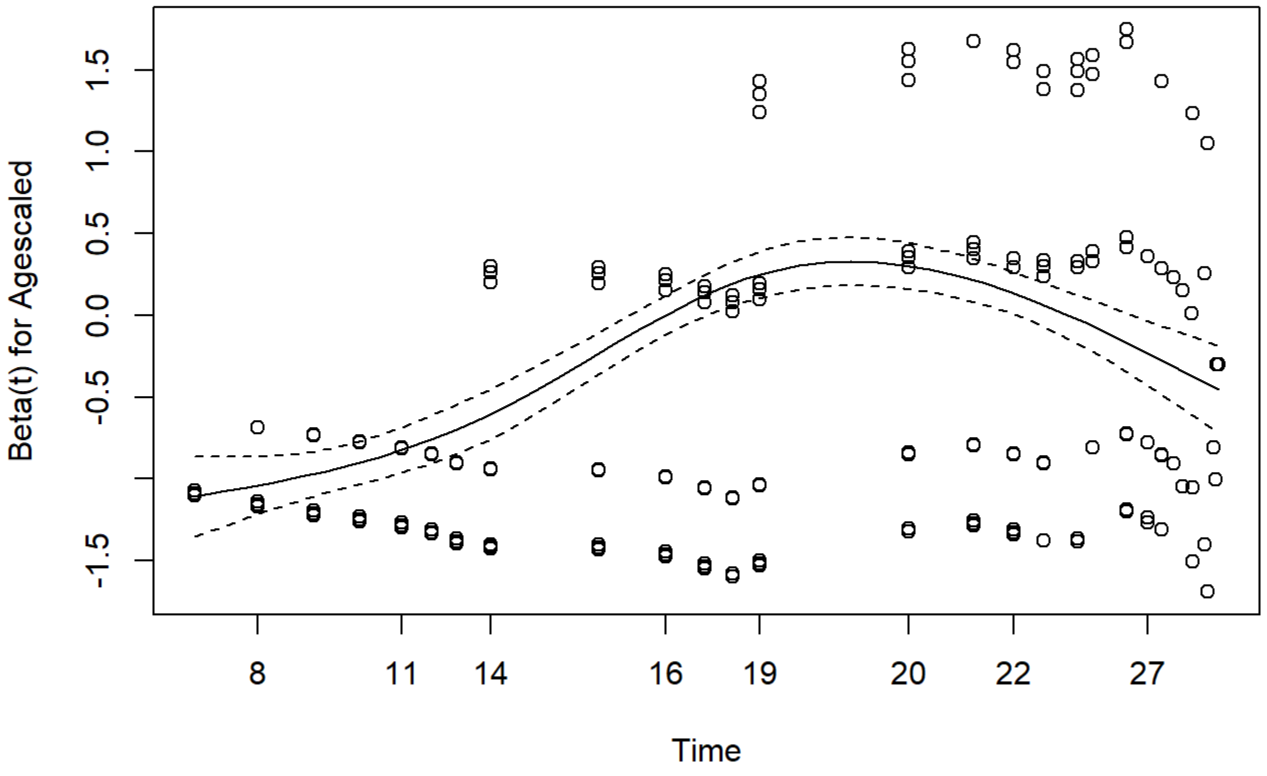
Beta(t) for Agescaled
Time
8
11
14
16
19
20
22
27
-1.5
-0.5
0.0
0.5
1.0
1.5

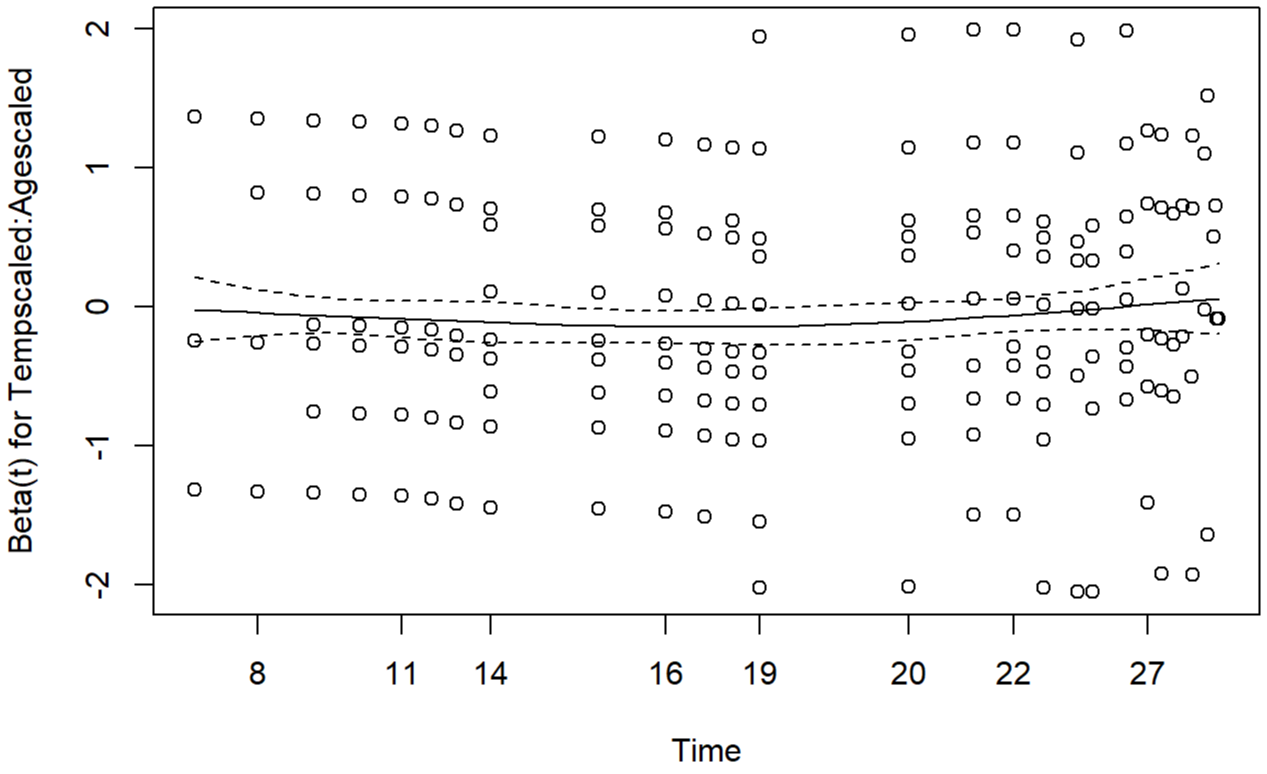


```
str(Fecundity_data_firstBM_ovipossurvival_numeric)
```

```
## 'data.frame':	682 obs. of  41 variables:
##  $ ID_overall               : num  1 2 3 4 5 6 7 8 11 12 ...
##  $ Temperature              : num  32 32 32 32 32 32 32 32 27 27 ...
##  $ Age                      : num  3 3 3 3 3 3 3 3 3 3 ...
##  $ ID_per_group             : num  1 2 3 4 5 6 7 8 1 2 ...
##  $ Trial_start_date         : POSIXct, format: "2024-02-13" "2024-02-13" ...
##  $ Trial_number             : Factor w/ 5 levels "1","2","3","4",..: 1 1 1 1 1 1 1 1 1 1 ...
##  $ BM1_Date                 : POSIXct, format: "2024-02-13" "2024-02-13" ...
##  $ Age_of_BM                : num  3 3 3 3 3 3 3 3 3 3 ...
##  $ Bloodmeal_number         : Factor w/ 1 level "1": 1 1 1 1 1 1 1 1 1 1 ...
##  $ Oviposition_positive(y/n): chr  "Y" "Y" "Y" "Y" ...
##  $ Eggs_day3                : num  0 0 0 0 0 14 NA NA 0 72 ...
##  $ Eggs_day4                : num  0 0 0 0 0 0 NA NA 0 10 ...
##  $ Larvae_day4              : num  0 0 0 0 0 0 0 0 NA 0 ...
##  $ Surv_to_eggs(y/n)        : Factor w/ 3 levels "N","NA","Y": 3 3 3 3 3 3 1 1 3 3 ...
##  $ Surv_to_larvae(y/n)      : Factor w/ 3 levels "N","NA","Y": 3 3 3 3 3 3 2 2 3 3 ...
##  $ Date_of_death            : POSIXct, format: "2024-03-01" "2024-03-07" ...
##  $ Censor                   : num  1 1 1 1 1 1 1 1 1 1 ...
##  $ Notes                    : chr  NA NA NA NA ...
##  $ Oviposition_positive     : Factor w/ 2 levels "N","Y": 2 2 2 2 2 2 2 2 2 2 ...
##  $ total_eggs               : num  0 0 0 0 0 14 NA NA 0 82 ...
##  $ Percent_eggs_day3        : num  NaN NaN NaN NaN NaN ...
##  $ Percent_eggs_day4        : num  NaN NaN NaN NaN NaN ...
##  $ egg_binary               : Factor w/ 2 levels "Did not lay eggs",..: 1 1 1 1 1 2 NA NA 1 2 ...
##  $ egg_binary_day3          : num  0 0 0 0 0 1 NA NA 0 1 ...
##  $ egg_binary_day4          : num  0 0 0 0 0 0 NA NA 0 1 ...
##  $ larvae_binary            : num  0 0 0 0 0 0 0 0 NA 0 ...
##  $ Percent_eggshatchedtolarv: num  NaN NaN NaN NaN NaN 0 NA NA NA 0 ...
##  $ Days_to_death_post_BM    : 'difftime' num  17 23 18 13 ...
##   ..- attr(*, "units")= chr "days"
##  $ Age_of_death             : 'difftime' num  20 26 21 16 ...
##   ..- attr(*, "units")= chr "days"
##  $ Age_of_BM_days           : 'difftime' num  3 3 3 3 ...
##   ..- attr(*, "units")= chr "days"
##  $ Date_of_eclosion         : POSIXct, format: "2024-02-10" "2024-02-10" ...
##  $ days_alive_post_BM       : num  17 23 18 13 15 18 3 3 12 12 ...
##  $ days_alive_post_eclosion : num  20 26 21 16 18 21 6 6 15 15 ...
##  $ group                    : chr  "3_32" "3_32" "3_32" "3_32" ...
##  $ start_time               : num  4 4 4 4 4 4 4 4 4 4 ...
##  $ stop_time                : num  21 27 22 17 19 22 7 7 16 16 ...
##  $ stopminusstart           : num  17 23 18 13 15 18 3 3 12 12 ...
##  $ Trial_start_date_factor  : Factor w/ 32 levels "2024-02-13","2024-02-19",..: 1 1 1 1 1 1 1 1 20 20
...
##  $ Trial_start_date_number  : num  1 1 1 1 1 1 1 1 20 20 ...
##  $ Tempscaled               : num [1:682, 1] 1.33 1.33 1.33 1.33 1.33 ...
##   ..- attr(*, "scaled:center")= num 29.4
##   ..- attr(*, "scaled:scale")= num 1.93
##  $ Agescaled                : num [1:682, 1] -0.999 -0.999 -0.999 -0.999 -0.999 ...
##   ..- attr(*, "scaled:center")= num 7.49
##   ..- attr(*, "scaled:scale")= num 4.5
```

```
mv_fit <- coxph(Surv(start_time,stop_time, Censor) ~ egg_binary*Temperature*Age+cluster(Trial_start_date_fa
ctor), robust=TRUE,
                data = Fecundity_data_firstBM_ovipossurvival_numeric,method="breslow")
cz <- cox.zph(mv_fit)
print(cz)
```

```
##                              chisq df       p
## egg_binary                   12.21  1 0.00048
## Temperature                   6.53  1 0.01060
## Age                           3.53  1 0.06011
## egg_binary:Temperature       12.69  1 0.00037
## egg_binary:Age               14.26  1 0.00016
## Temperature:Age               4.88  1 0.02712
## egg_binary:Temperature:Age 15.17  1 9.8e-05
## GLOBAL                       33.02  7 2.6e-05
```

```
#non-proportional hazards:
#coxph regression with weighted estimation, accounting for experimental block

library(condsurv)
library(coxphw)
fit1 <- coxphw(Surv(start_time,stop_time, Censor) ~ egg_binary*Temperature*Age +
                 frailty(Trial_start_date_factor,distribution = "gaussian"),
               data = Fecundity_data_firstBM_ovipossurvival_numeric,
               template = "AHR")

summary(fit1)
```

```
## coxphw(formula = Surv(start_time, stop_time, Censor) ~ egg_binary *
##     Temperature * Age + frailty(Trial_start_date_factor, distribution = "gaussian"),
##     data = Fecundity_data_firstBM_ovipossurvival_numeric, template = "AHR")
##
## Model fitted by weighted estimation (AHR template)
##
##                                                                 coef
## egg_binaryLaid eggs                                     -2.861929114
## Temperature                                              0.097470372
## Age                                                      0.232252645
## frailty(Trial_start_date_factor, distribution = "gaussian")  0.039226370
## egg_binaryLaid eggs:Temperature                          0.088320500
## egg_binaryLaid eggs:Age                                  0.122789243
## Temperature:Age                                         -0.006814610
## egg_binaryLaid eggs:Temperature:Age                     -0.004847259
##                                                            se(coef)
## egg_binaryLaid eggs                                      2.956897827
## Temperature                                              0.061405196
## Age                                                      0.202974631
## frailty(Trial_start_date_factor, distribution = "gaussian") 0.006548507
## egg_binaryLaid eggs:Temperature                          0.098625686
## egg_binaryLaid eggs:Age                                  0.344162066
## Temperature:Age                                          0.006671019
## egg_binaryLaid eggs:Temperature:Age                      0.011639842
##                                                            exp(coef)
## egg_binaryLaid eggs                                      0.05715839
## Temperature                                              1.10237878
## Age                                                      1.26143838
## frailty(Trial_start_date_factor, distribution = "gaussian") 1.04000588
## egg_binaryLaid eggs:Temperature                          1.09233816
## egg_binaryLaid eggs:Age                                  1.13064610
## Temperature:Age                                          0.99320856
## egg_binaryLaid eggs:Temperature:Age                      0.99516447
##                                                              lower 0.95
## egg_binaryLaid eggs                                      0.0001738457
## Temperature                                              0.9773781975
## Age                                                      0.8474102692
## frailty(Trial_start_date_factor, distribution = "gaussian") 1.0267428718
## egg_binaryLaid eggs:Temperature                          0.9003404648
## egg_binaryLaid eggs:Age                                  0.5759375690
## Temperature:Age                                          0.9803069259
## egg_binaryLaid eggs:Temperature:Age                      0.9727181297
##                                                          upper 0.95
## egg_binaryLaid eggs                                       18.792994
## Temperature                                                1.243366
## Age                                                        1.877753
## frailty(Trial_start_date_factor, distribution = "gaussian")   1.053440
## egg_binaryLaid eggs:Temperature                            1.325279
## egg_binaryLaid eggs:Age                                    2.219617
## Temperature:Age                                            1.006280
## egg_binaryLaid eggs:Temperature:Age                        1.018129
##                                                                   z
## egg_binaryLaid eggs                                      -0.9678823
## Temperature                                               1.5873310
## Age                                                       1.1442447
## frailty(Trial_start_date_factor, distribution = "gaussian")  5.9901239
```

```
## egg_binaryLaid eggs:Temperature                               0.8955121
## egg_binaryLaid eggs:Age                                       0.3567774
## Temperature:Age                                              -1.0215247
## egg_binaryLaid eggs:Temperature:Age                          -0.4164369
##                                                                      p
## egg_binaryLaid eggs                                          3.331031e-01
## Temperature                                                  1.124377e-01
## Age                                                          2.525222e-01
## frailty(Trial_start_date_factor, distribution = "gaussian") 2.096813e-09
## egg_binaryLaid eggs:Temperature                              3.705134e-01
## egg_binaryLaid eggs:Age                                      7.212585e-01
## Temperature:Age                                              3.070059e-01
## egg_binaryLaid eggs:Temperature:Age                          6.770904e-01
##
## Wald Chi-square = 102.7208 on 8  df  p = 0  n = 638
##
## Covariance-Matrix:
##                                                              egg_binaryLaid eggs
## egg_binaryLaid eggs                                                 8.743244757
## Temperature                                                         0.115068953
## Age                                                                 0.343753571
## frailty(Trial_start_date_factor, distribution = "gaussian")        0.005468501
## egg_binaryLaid eggs:Temperature                                    -0.291073412
## egg_binaryLaid eggs:Age                                            -0.890851239
## Temperature:Age                                                    -0.011245269
## egg_binaryLaid eggs:Temperature:Age                                 0.029841080
##                                                                Temperature
## egg_binaryLaid eggs                                           0.1150689526
## Temperature                                                   0.0037705981
## Age                                                           0.0109600190
## frailty(Trial_start_date_factor, distribution = "gaussian")  0.0001459497
## egg_binaryLaid eggs:Temperature                              -0.0038155455
## egg_binaryLaid eggs:Age                                      -0.0107549138
## Temperature:Age                                              -0.0003620725
## egg_binaryLaid eggs:Temperature:Age                           0.0003587143
##                                                                        Age
## egg_binaryLaid eggs                                           0.3437535709
## Temperature                                                   0.0109600190
## Age                                                           0.0411987009
## frailty(Trial_start_date_factor, distribution = "gaussian")  0.0005479018
## egg_binaryLaid eggs:Temperature                              -0.0113740895
## egg_binaryLaid eggs:Age                                      -0.0408820498
## Temperature:Age                                              -0.0013513313
## egg_binaryLaid eggs:Temperature:Age                           0.0013565115
##                                                              frailty(Trial_start_date_factor, distributio
n = "gaussian")
## egg_binaryLaid eggs
5.468501e-03
## Temperature
1.459497e-04
## Age
5.479018e-04
## frailty(Trial_start_date_factor, distribution = "gaussian")
4.288295e-05
## egg_binaryLaid eggs:Temperature
-1.837587e-04
```

```
## egg_binaryLaid eggs:Age
-5.544556e-04
## Temperature:Age
-1.763793e-05
## egg_binaryLaid eggs:Temperature:Age
1.915732e-05
##                                                               egg_binaryLaid eggs:Temperature
## egg_binaryLaid eggs                                                             -0.2910734119
## Temperature                                                                     -0.0038155455
## Age                                                                             -0.0113740895
## frailty(Trial_start_date_factor, distribution = "gaussian")                     -0.0001837587
## egg_binaryLaid eggs:Temperature                                                  0.0097270260
## egg_binaryLaid eggs:Age                                                          0.0298083650
## Temperature:Age                                                                  0.0003732376
## egg_binaryLaid eggs:Temperature:Age                                             -0.0010027380
##                                                               egg_binaryLaid eggs:Age
## egg_binaryLaid eggs                                                     -0.8908512388
## Temperature                                                             -0.0107549138
## Age                                                                     -0.0408820498
## frailty(Trial_start_date_factor, distribution = "gaussian")             -0.0005544556
## egg_binaryLaid eggs:Temperature                                          0.0298083650
## egg_binaryLaid eggs:Age                                                  0.1184475277
## Temperature:Age                                                          0.0013368801
## egg_binaryLaid eggs:Temperature:Age                                     -0.0039965911
##                                                               Temperature:Age
## egg_binaryLaid eggs                                             -1.124527e-02
## Temperature                                                     -3.620725e-04
## Age                                                             -1.351331e-03
## frailty(Trial_start_date_factor, distribution = "gaussian")     -1.763793e-05
## egg_binaryLaid eggs:Temperature                                  3.732376e-04
## egg_binaryLaid eggs:Age                                          1.336880e-03
## Temperature:Age                                                  4.450249e-05
## egg_binaryLaid eggs:Temperature:Age                             -4.449826e-05
##                                                               egg_binaryLaid eggs:Temperature:Age
## egg_binaryLaid eggs                                                                  2.984108e-02
## Temperature                                                                          3.587143e-04
## Age                                                                                  1.356511e-03
## frailty(Trial_start_date_factor, distribution = "gaussian")                          1.915732e-05
## egg_binaryLaid eggs:Temperature                                                     -1.002738e-03
## egg_binaryLaid eggs:Age                                                             -3.996591e-03
## Temperature:Age                                                                     -4.449826e-05
## egg_binaryLaid eggs:Temperature:Age                                                  1.354859e-04
##
## Generalized concordance probability:
##                                                               concordance prob.
## egg_binaryLaid eggs                                                      0.0541
## Temperature                                                              0.5243
## Age                                                                      0.5578
## frailty(Trial_start_date_factor, distribution = "gaussian")              0.5098
## egg_binaryLaid eggs:Temperature                                          0.5221
## egg_binaryLaid eggs:Age                                                  0.5307
## Temperature:Age                                                          0.4983
## egg_binaryLaid eggs:Temperature:Age                                      0.4988
##                                                               lower 0.95
## egg_binaryLaid eggs                                               0.0002
## Temperature                                                       0.4943
```

```
## Age                                                          0.4587
## frailty(Trial_start_date_factor, distribution = "gaussian")     0.5066
## egg_binaryLaid eggs:Temperature                              0.4738
## egg_binaryLaid eggs:Age                                      0.3655
## Temperature:Age                                              0.4950
## egg_binaryLaid eggs:Temperature:Age                          0.4931
##                                                          upper 0.95
## egg_binaryLaid eggs                                          0.9495
## Temperature                                                  0.5542
## Age                                                          0.6525
## frailty(Trial_start_date_factor, distribution = "gaussian")     0.5130
## egg_binaryLaid eggs:Temperature                              0.5699
## egg_binaryLaid eggs:Age                                      0.6894
## Temperature:Age                                              0.5016
## egg_binaryLaid eggs:Temperature:Age                          0.5045
```

```
plot(fit1$dfbeta.resid) # residuals are bad
```

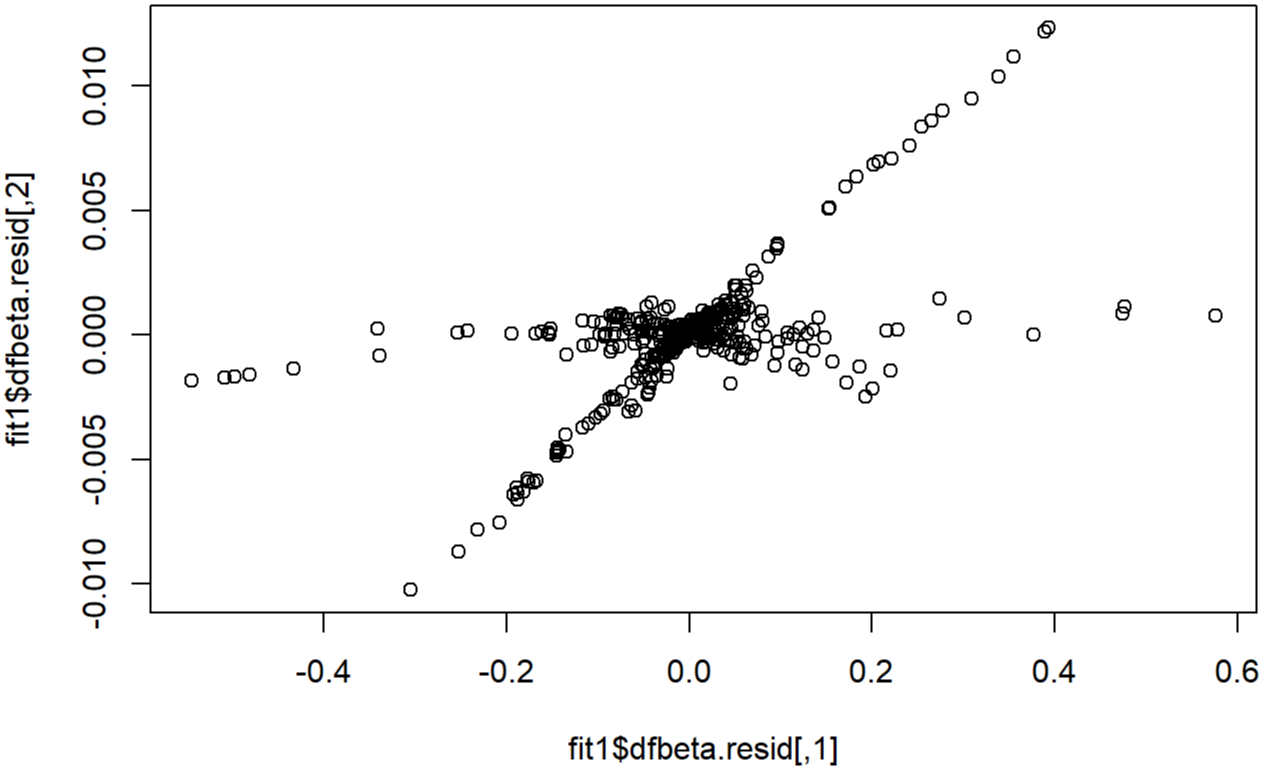


```
#residuals are bad


fit2 <- coxphw(Surv(start_time,stop_time, Censor) ~ egg_binary+
                 Tempscaled*Agescaled +
                 frailty(Trial_start_date_factor,distribution = "gaussian"),
               data =Fecundity_data_firstBM_ovipossurvival_numeric,
               template = "AHR")
summary(fit2)
```

```
## coxphw(formula = Surv(start_time, stop_time, Censor) ~ egg_binary +
##     Tempscaled * Agescaled + frailty(Trial_start_date_factor,
##     distribution = "gaussian"), data = Fecundity_data_firstBM_ovipossurvival_numeric,
##     template = "AHR")
##
## Model fitted by weighted estimation (AHR template)
##
##                                                                       coef
## egg_binaryLaid eggs                                            -0.36608189
## Tempscaled                                                      0.13311468
## Agescaled                                                       0.11854677
## frailty(Trial_start_date_factor, distribution = "gaussian")  0.04077020
## Tempscaled:Agescaled                                           -0.06349615
##                                                                   se(coef)
## egg_binaryLaid eggs                                            0.099432267
## Tempscaled                                                     0.045146153
## Agescaled                                                      0.054115542
## frailty(Trial_start_date_factor, distribution = "gaussian") 0.006284334
## Tempscaled:Agescaled                                           0.046495664
##                                                               exp(coef)
## egg_binaryLaid eggs                                            0.6934460
## Tempscaled                                                     1.1423810
## Agescaled                                                      1.1258595
## frailty(Trial_start_date_factor, distribution = "gaussian") 1.0416127
## Tempscaled:Agescaled                                           0.9384777
##                                                               lower 0.95
## egg_binaryLaid eggs                                             0.5706578
## Tempscaled                                                      1.0456407
## Agescaled                                                       1.0125605
## frailty(Trial_start_date_factor, distribution = "gaussian")  1.0288618
## Tempscaled:Agescaled                                            0.8567356
##                                                               upper 0.95
## egg_binaryLaid eggs                                             0.8426545
## Tempscaled                                                      1.2480714
## Agescaled                                                       1.2518360
## frailty(Trial_start_date_factor, distribution = "gaussian")  1.0545217
## Tempscaled:Agescaled                                            1.0280190
##                                                                       z
## egg_binaryLaid eggs                                            -3.681721
## Tempscaled                                                      2.948528
## Agescaled                                                       2.190623
## frailty(Trial_start_date_factor, distribution = "gaussian")  6.487592
## Tempscaled:Agescaled                                           -1.365636
##                                                                         p
## egg_binaryLaid eggs                                            2.316647e-04
## Tempscaled                                                     3.192915e-03
## Agescaled                                                      2.847906e-02
## frailty(Trial_start_date_factor, distribution = "gaussian") 8.721879e-11
## Tempscaled:Agescaled                                           1.720532e-01
##
## Wald Chi-square = 95.88259 on 5  df  p = 0  n = 638
##
## Covariance-Matrix:
##                                                               egg_binaryLaid eggs
## egg_binaryLaid eggs                                                  9.886776e-03
## Tempscaled                                                           1.524836e-03
```

```
## Agescaled                                                          7.820996e-04
## frailty(Trial_start_date_factor, distribution = "gaussian")        9.362575e-05
## Tempscaled:Agescaled                                               1.332321e-04
##                                                                Tempscaled
## egg_binaryLaid eggs                                          1.524836e-03
## Tempscaled                                                   2.038175e-03
## Agescaled                                                   -4.124589e-05
## frailty(Trial_start_date_factor, distribution = "gaussian") -8.350538e-06
## Tempscaled:Agescaled                                        -1.681021e-04
##                                                                 Agescaled
## egg_binaryLaid eggs                                          7.820996e-04
## Tempscaled                                                  -4.124589e-05
## Agescaled                                                    2.928492e-03
## frailty(Trial_start_date_factor, distribution = "gaussian")  1.134939e-04
## Tempscaled:Agescaled                                        -7.666555e-04
##                                                             frailty(Trial_start_date_factor, distributio
n = "gaussian")
## egg_binaryLaid eggs
9.362575e-05
## Tempscaled
-8.350538e-06
## Agescaled
1.134939e-04
## frailty(Trial_start_date_factor, distribution = "gaussian")
3.949285e-05
## Tempscaled:Agescaled
-9.964885e-05
##                                                             Tempscaled:Agescaled
## egg_binaryLaid eggs                                                 1.332321e-04
## Tempscaled                                                         -1.681021e-04
## Agescaled                                                          -7.666555e-04
## frailty(Trial_start_date_factor, distribution = "gaussian")        -9.964885e-05
## Tempscaled:Agescaled                                                2.161847e-03
##
## Generalized concordance probability:
##                                                             concordance prob.
## egg_binaryLaid eggs                                                    0.4095
## Tempscaled                                                             0.5332
## Agescaled                                                              0.5296
## frailty(Trial_start_date_factor, distribution = "gaussian")            0.5102
## Tempscaled:Agescaled                                                   0.4841
##                                                             lower 0.95
## egg_binaryLaid eggs                                             0.3633
## Tempscaled                                                      0.5112
## Agescaled                                                       0.5031
## frailty(Trial_start_date_factor, distribution = "gaussian")     0.5071
## Tempscaled:Agescaled                                            0.4614
##                                                             upper 0.95
## egg_binaryLaid eggs                                             0.4573
## Tempscaled                                                      0.5552
## Agescaled                                                       0.5559
## frailty(Trial_start_date_factor, distribution = "gaussian")     0.5133
## Tempscaled:Agescaled                                            0.5069
```

```
plot(fit2$dfbeta.resid) # resdiuals look better
```

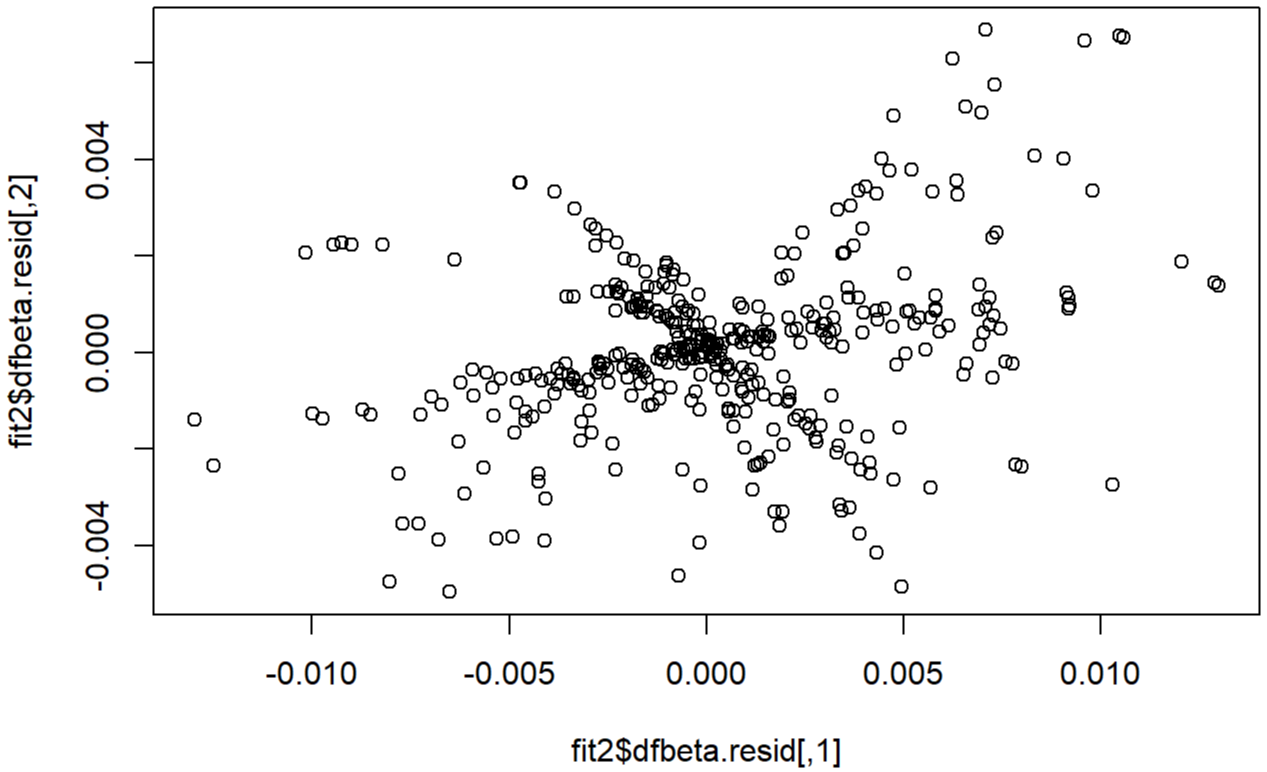

fit2$dfbeta.resid[,2]
-0.004
0.000
0.004
-0.010
-0.005
0.000
0.005
0.010
fit2$dfbeta.resid[,1]


```
fit2
```

```
## coxphw(formula = Surv(start_time, stop_time, Censor) ~ egg_binary +
##     Tempscaled * Agescaled + frailty(Trial_start_date_factor,
##     distribution = "gaussian"), data = Fecundity_data_firstBM_ovipossurvival_numeric,
##     template = "AHR")
##
## Model fitted by weighted estimation (AHR template)
##
##                                                                        coef
## egg_binaryLaid eggs                                             -0.36608189
## Tempscaled                                                       0.13311468
## Agescaled                                                        0.11854677
## frailty(Trial_start_date_factor, distribution = "gaussian")  0.04077020
## Tempscaled:Agescaled                                            -0.06349615
##                                                                     se(coef)
## egg_binaryLaid eggs                                             0.099432267
## Tempscaled                                                      0.045146153
## Agescaled                                                       0.054115542
## frailty(Trial_start_date_factor, distribution = "gaussian") 0.006284334
## Tempscaled:Agescaled                                            0.046495664
##                                                                 exp(coef)
## egg_binaryLaid eggs                                             0.6934460
## Tempscaled                                                      1.1423810
## Agescaled                                                       1.1258595
## frailty(Trial_start_date_factor, distribution = "gaussian") 1.0416127
## Tempscaled:Agescaled                                            0.9384777
##                                                                 lower 0.95
## egg_binaryLaid eggs                                              0.5706578
## Tempscaled                                                       1.0456407
## Agescaled                                                        1.0125605
## frailty(Trial_start_date_factor, distribution = "gaussian")  1.0288618
## Tempscaled:Agescaled                                             0.8567356
##                                                                 upper 0.95
## egg_binaryLaid eggs                                              0.8426545
## Tempscaled                                                       1.2480714
## Agescaled                                                        1.2518360
## frailty(Trial_start_date_factor, distribution = "gaussian")  1.0545217
## Tempscaled:Agescaled                                             1.0280190
##                                                                         z
## egg_binaryLaid eggs                                             -3.681721
## Tempscaled                                                       2.948528
## Agescaled                                                        2.190623
## frailty(Trial_start_date_factor, distribution = "gaussian")  6.487592
## Tempscaled:Agescaled                                            -1.365636
##                                                                            p
## egg_binaryLaid eggs                                             2.316647e-04
## Tempscaled                                                      3.192915e-03
## Agescaled                                                       2.847906e-02
## frailty(Trial_start_date_factor, distribution = "gaussian") 8.721879e-11
## Tempscaled:Agescaled                                            1.720532e-01
##
## Wald Chi-square=95.88259 on 5df, p=0, n=638
```

```
fit3 <- coxphw(Surv(start_time,stop_time, Censor) ~ egg_binary+
                 Tempscaled*Agescaled,
               data =Fecundity_data_firstBM_ovipossurvival_numeric,
               template = "AHR")
summary(fit3)
```

```
## coxphw(formula = Surv(start_time, stop_time, Censor) ~ egg_binary +
##     Tempscaled * Agescaled, data = Fecundity_data_firstBM_ovipossurvival_numeric,
##     template = "AHR")
##
## Model fitted by weighted estimation (AHR template)
##
##                             coef   se(coef) exp(coef) lower 0.95 upper 0.95
## egg_binaryLaid eggs  -0.49322271 0.09961969 0.6106553  0.5023422  0.7423224
## Tempscaled            0.16554268 0.04365033 1.1800333  1.0832758  1.2854332
## Agescaled            -0.05950823 0.05229831 0.9422278  0.8504318  1.0439322
## Tempscaled:Agescaled  0.03273208 0.04450217 1.0332737  0.9469674  1.1274458
##                               z            p
## egg_binaryLaid eggs  -4.9510562 7.381178e-07
## Tempscaled            3.7924726 1.491547e-04
## Agescaled            -1.1378614 2.551784e-01
## Tempscaled:Agescaled  0.7355165 4.620250e-01
##
## Wald Chi-square = 64.74428 on 4  df  p = 2.913225e-13  n = 638
##
## Covariance-Matrix:
##                      egg_binaryLaid eggs    Tempscaled     Agescaled
## egg_binaryLaid eggs         0.0099240835  1.647601e-03  5.669475e-04
## Tempscaled                  0.0016476006  1.905351e-03 -8.758065e-05
## Agescaled                   0.0005669475 -8.758065e-05  2.735114e-03
## Tempscaled:Agescaled        0.0002510083 -1.357451e-04 -3.908167e-04
##                      Tempscaled:Agescaled
## egg_binaryLaid eggs          0.0002510083
## Tempscaled                  -0.0001357451
## Agescaled                   -0.0003908167
## Tempscaled:Agescaled         0.0019804431
##
## Generalized concordance probability:
##                      concordance prob. lower 0.95 upper 0.95
## egg_binaryLaid eggs             0.3791     0.3344     0.4261
## Tempscaled                      0.5413     0.5200     0.5624
## Agescaled                       0.4851     0.4596     0.5107
## Tempscaled:Agescaled            0.5082     0.4864     0.5300
```

```
plot(fit3$dfbeta.resid) # resdiuals look better
```

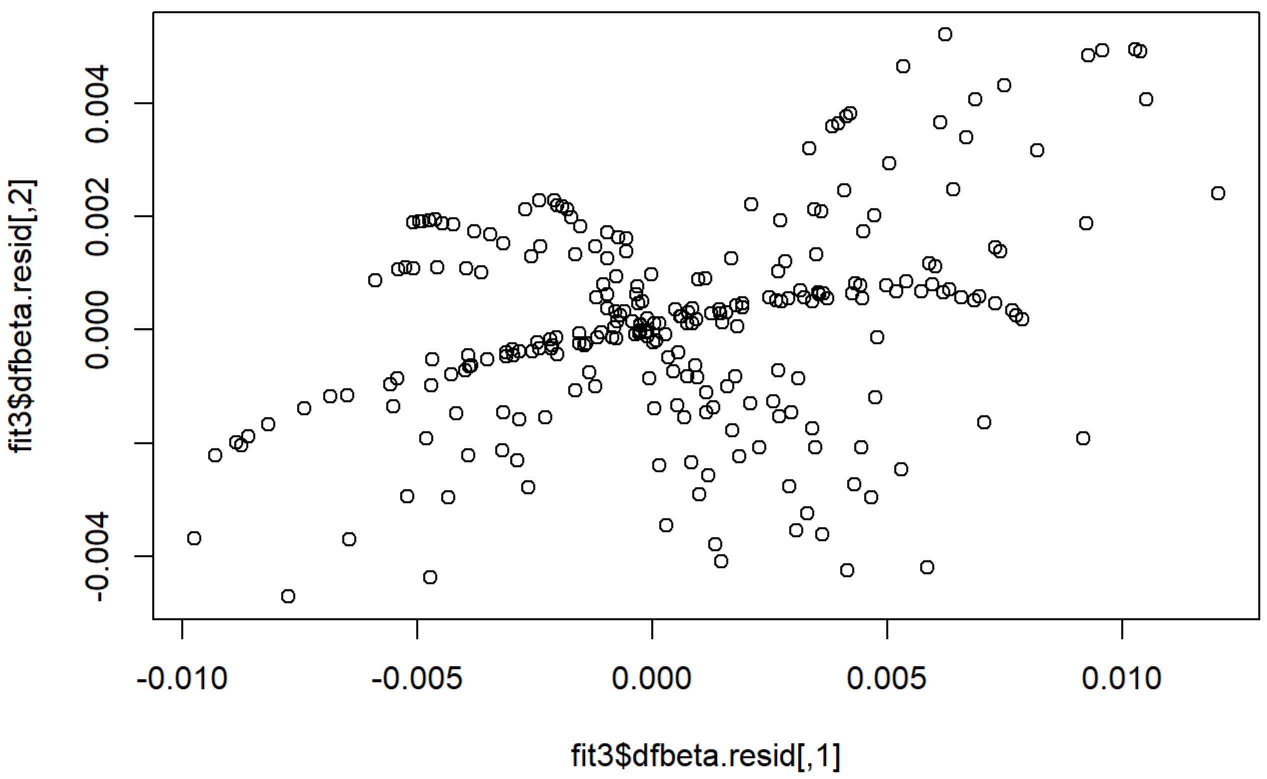


```
fit4 <- coxphw(Surv(start_time,stop_time, Censor) ~ Tempscaled*Agescaled +
                 strata(egg_binary)+
                 frailty(Trial_start_date_factor,distribution = "gaussian"),
               data =Fecundity_data_firstBM_ovipossurvival_numeric,
               template = "AHR")
summary(fit4)
```

```
## coxphw(formula = Surv(start_time, stop_time, Censor) ~ Tempscaled *
##     Agescaled + strata(egg_binary) + frailty(Trial_start_date_factor,
##     distribution = "gaussian"), data = Fecundity_data_firstBM_ovipossurvival_numeric,
##     template = "AHR")
##
## Model fitted by weighted estimation (AHR template)
##
##                                                                    coef
## Tempscaled                                                   0.13311468
## Agescaled                                                    0.11854677
## strata(egg_binary)Laid eggs                                 -0.36608189
## frailty(Trial_start_date_factor, distribution = "gaussian")  0.04077020
## Tempscaled:Agescaled                                        -0.06349615
##                                                                se(coef)
## Tempscaled                                                  0.045146153
## Agescaled                                                   0.054115542
## strata(egg_binary)Laid eggs                                 0.099432267
## frailty(Trial_start_date_factor, distribution = "gaussian") 0.006284334
## Tempscaled:Agescaled                                        0.046495664
##                                                             exp(coef)
## Tempscaled                                                  1.1423810
## Agescaled                                                   1.1258595
## strata(egg_binary)Laid eggs                                 0.6934460
## frailty(Trial_start_date_factor, distribution = "gaussian") 1.0416127
## Tempscaled:Agescaled                                        0.9384777
##                                                             lower 0.95
## Tempscaled                                                   1.0456407
## Agescaled                                                    1.0125605
## strata(egg_binary)Laid eggs                                  0.5706578
## frailty(Trial_start_date_factor, distribution = "gaussian")  1.0288618
## Tempscaled:Agescaled                                         0.8567356
##                                                             upper 0.95
## Tempscaled                                                   1.2480714
## Agescaled                                                    1.2518360
## strata(egg_binary)Laid eggs                                  0.8426545
## frailty(Trial_start_date_factor, distribution = "gaussian")  1.0545217
## Tempscaled:Agescaled                                         1.0280190
##                                                                     z
## Tempscaled                                                   2.948528
## Agescaled                                                    2.190623
## strata(egg_binary)Laid eggs                                 -3.681721
## frailty(Trial_start_date_factor, distribution = "gaussian")  6.487592
## Tempscaled:Agescaled                                        -1.365636
##                                                                        p
## Tempscaled                                                  3.192915e-03
## Agescaled                                                   2.847906e-02
## strata(egg_binary)Laid eggs                                 2.316647e-04
## frailty(Trial_start_date_factor, distribution = "gaussian") 8.721879e-11
## Tempscaled:Agescaled                                        1.720532e-01
##
## Wald Chi-square = 95.88259 on 5  df  p = 0  n = 638
##
## Covariance-Matrix:
##                                                                Tempscaled
## Tempscaled                                                   2.038175e-03
## Agescaled                                                   -4.124589e-05
```

```
## strata(egg_binary)Laid eggs                                     1.524836e-03
## frailty(Trial_start_date_factor, distribution = "gaussian") -8.350538e-06
## Tempscaled:Agescaled                                         -1.681021e-04
##                                                                  Agescaled
## Tempscaled                                                   -4.124589e-05
## Agescaled                                                     2.928492e-03
## strata(egg_binary)Laid eggs                                   7.820996e-04
## frailty(Trial_start_date_factor, distribution = "gaussian")  1.134939e-04
## Tempscaled:Agescaled                                         -7.666555e-04
##                                                              strata(egg_binary)Laid eggs
## Tempscaled                                                                  1.524836e-03
## Agescaled                                                                   7.820996e-04
## strata(egg_binary)Laid eggs                                                 9.886776e-03
## frailty(Trial_start_date_factor, distribution = "gaussian")                 9.362575e-05
## Tempscaled:Agescaled                                                        1.332321e-04
##                                                              frailty(Trial_start_date_factor, distributio
n = "gaussian")
## Tempscaled
-8.350538e-06
## Agescaled
1.134939e-04
## strata(egg_binary)Laid eggs
9.362575e-05
## frailty(Trial_start_date_factor, distribution = "gaussian")
3.949285e-05
## Tempscaled:Agescaled
-9.964885e-05
##                                                              Tempscaled:Agescaled
## Tempscaled                                                           -1.681021e-04
## Agescaled                                                            -7.666555e-04
## strata(egg_binary)Laid eggs                                           1.332321e-04
## frailty(Trial_start_date_factor, distribution = "gaussian")          -9.964885e-05
## Tempscaled:Agescaled                                                  2.161847e-03
##
## Generalized concordance probability:
##                                                              concordance prob.
## Tempscaled                                                              0.5332
## Agescaled                                                               0.5296
## strata(egg_binary)Laid eggs                                             0.4095
## frailty(Trial_start_date_factor, distribution = "gaussian")             0.5102
## Tempscaled:Agescaled                                                    0.4841
##                                                              lower 0.95
## Tempscaled                                                       0.5112
## Agescaled                                                        0.5031
## strata(egg_binary)Laid eggs                                      0.3633
## frailty(Trial_start_date_factor, distribution = "gaussian")      0.5071
## Tempscaled:Agescaled                                             0.4614
##                                                              upper 0.95
## Tempscaled                                                       0.5552
## Agescaled                                                        0.5559
## strata(egg_binary)Laid eggs                                      0.4573
## frailty(Trial_start_date_factor, distribution = "gaussian")      0.5133
## Tempscaled:Agescaled                                             0.5069
```

```
plot(fit4$dfbeta.resid) # resdiuals look best!
```

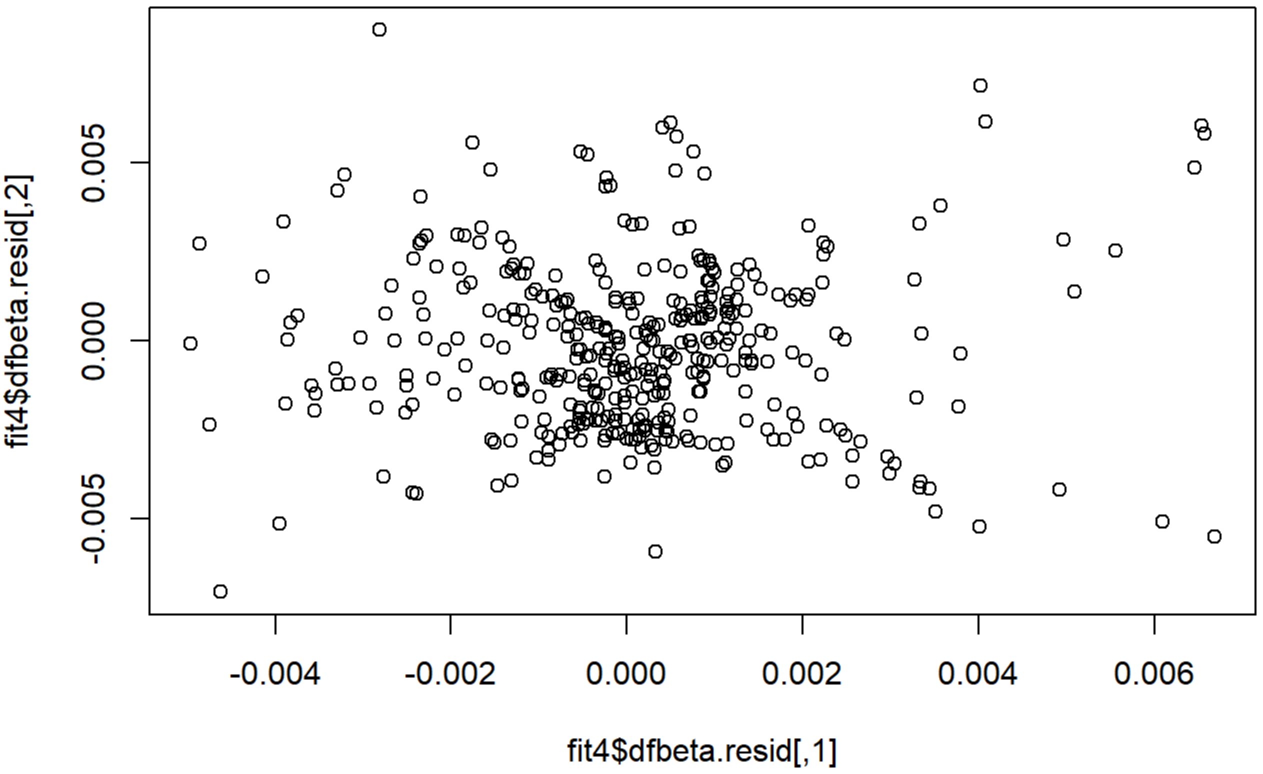
fit4$dfbeta.resid[,2]
0.005
0.000
-0.005
-0.004
-0.002
0.000
0.002
0.004
0.006
fit4$dfbeta.resid[,1]

```
fit4
```

```
## coxphw(formula = Surv(start_time, stop_time, Censor) ~ Tempscaled *
##     Agescaled + strata(egg_binary) + frailty(Trial_start_date_factor,
##     distribution = "gaussian"), data = Fecundity_data_firstBM_ovipossurvival_numeric,
##     template = "AHR")
##
## Model fitted by weighted estimation (AHR template)
##
##                                                                  coef
## Tempscaled                                                 0.13311468
## Agescaled                                                  0.11854677
## strata(egg_binary)Laid eggs                               -0.36608189
## frailty(Trial_start_date_factor, distribution = "gaussian")  0.04077020
## Tempscaled:Agescaled                                      -0.06349615
##                                                              se(coef)
## Tempscaled                                                0.045146153
## Agescaled                                                 0.054115542
## strata(egg_binary)Laid eggs                               0.099432267
## frailty(Trial_start_date_factor, distribution = "gaussian") 0.006284334
## Tempscaled:Agescaled                                      0.046495664
##                                                           exp(coef)
## Tempscaled                                                1.1423810
## Agescaled                                                 1.1258595
## strata(egg_binary)Laid eggs                               0.6934460
## frailty(Trial_start_date_factor, distribution = "gaussian") 1.0416127
## Tempscaled:Agescaled                                      0.9384777
##                                                           lower 0.95
## Tempscaled                                                 1.0456407
## Agescaled                                                  1.0125605
## strata(egg_binary)Laid eggs                                0.5706578
## frailty(Trial_start_date_factor, distribution = "gaussian")  1.0288618
## Tempscaled:Agescaled                                       0.8567356
##                                                           upper 0.95
## Tempscaled                                                 1.2480714
## Agescaled                                                  1.2518360
## strata(egg_binary)Laid eggs                                0.8426545
## frailty(Trial_start_date_factor, distribution = "gaussian")  1.0545217
## Tempscaled:Agescaled                                       1.0280190
##                                                                   z
## Tempscaled                                                 2.948528
## Agescaled                                                  2.190623
## strata(egg_binary)Laid eggs                               -3.681721
## frailty(Trial_start_date_factor, distribution = "gaussian")  6.487592
## Tempscaled:Agescaled                                      -1.365636
##                                                                      p
## Tempscaled                                                3.192915e-03
## Agescaled                                                 2.847906e-02
## strata(egg_binary)Laid eggs                               2.316647e-04
## frailty(Trial_start_date_factor, distribution = "gaussian") 8.721879e-11
## Tempscaled:Agescaled                                      1.720532e-01
##
## Wald Chi-square=95.88259 on 5df, p=0, n=638
```

```
sink("Oviposition_survival/firstBM_coxphwsurvival_eggbinary.txt")
fit4
summary(fit4)
sink()


fit4$coefficients
```

```
##             [,1]
## [1,]  0.13311468
## [2,]  0.11854677
## [3,] -0.36608189
## [4,]  0.04077020
## [5,] -0.06349615
## attr(,"names")
## [1] "Tempscaled"
## [2] "Agescaled"
## [3] "strata(egg_binary)Laid eggs"
## [4] "frailty(Trial_start_date_factor, distribution = \"gaussian\")"
## [5] "Tempscaled:Agescaled"
```

```
#extract coefficients to plot hazard ratios:
coef <- as.numeric(fit4$coefficients[1:5])
expcoef <- as.numeric(exp(fit4$coefficients[1:5]))
names <- names(fit4$coefficients[1:5])
lowerCI <- as.numeric(fit4$ci.lower[1:5])
upperCI <- as.numeric(fit4$ci.upper[1:5])
pval <- as.numeric(fit4$prob[1:5])
Index <- c(1:4)
Label <- names(fit4$coefficients[1:5])

hazardratiotable <- as.data.frame(cbind(Label,coef,expcoef,lowerCI,upperCI,pval))
hazardratiotable
```

```
##                                                         Label
## 1                                                  Tempscaled
## 2                                                   Agescaled
## 3                                 strata(egg_binary)Laid eggs
## 4 frailty(Trial_start_date_factor, distribution = "gaussian")
## 5                                        Tempscaled:Agescaled
##                coef           expcoef           lowerCI           upperCI
## 1  0.133114681294067  1.14238100055046  1.04564074182193  1.24807144387351
## 2  0.118546765847299  1.12585952464546  1.01256048192639  1.25183600571037
## 3 -0.366081885897163 0.693446015435702 0.570657799863072 0.842654523322094
## 4 0.0407701984551365  1.04161271382945  1.02886178698565  1.05452166591769
## 5 -0.063496152872135 0.938477729737544  0.85673555455673  1.02801902469079
##                  pval
## 1  0.00319291548407685
## 2   0.0284790640369923
## 3 0.000231664674185006
## 4 8.72187877476449e-11
## 5    0.172053236470185
```

```
hazardratiotable <- hazardratiotable[-4,]

hazardratiotable <- as.data.frame(cbind(Index,hazardratiotable))

print(hazardratiotable)
```

```
##   Index                       Label               coef           expcoef
## 1     1                  Tempscaled  0.133114681294067  1.14238100055046
## 2     2                   Agescaled  0.118546765847299  1.12585952464546
## 3     3 strata(egg_binary)Laid eggs -0.366081885897163 0.693446015435702
## 5     4        Tempscaled:Agescaled -0.063496152872135 0.938477729737544
##             lowerCI           upperCI                 pval
## 1  1.04564074182193  1.24807144387351  0.00319291548407685
## 2  1.01256048192639  1.25183600571037   0.0284790640369923
## 3 0.570657799863072 0.842654523322094 0.000231664674185006
## 5  0.85673555455673  1.02801902469079    0.172053236470185
```

```
str(hazardratiotable)
```

```
## 'data.frame':    4 obs. of  7 variables:
##  $ Index  : int  1 2 3 4
##  $ Label  : chr  "Tempscaled" "Agescaled" "strata(egg_binary)Laid eggs" "Tempscaled:Agescaled"
##  $ coef   : chr  "0.133114681294067" "0.118546765847299" "-0.366081885897163" "-0.063496152872135"
##  $ expcoef: chr  "1.14238100055046" "1.12585952464546" "0.693446015435702" "0.938477729737544"
##  $ lowerCI: chr  "1.04564074182193" "1.01256048192639" "0.570657799863072" "0.85673555455673"
##  $ upperCI: chr  "1.24807144387351" "1.25183600571037" "0.842654523322094" "1.02801902469079"
##  $ pval   : chr  "0.00319291548407685" "0.0284790640369923" "0.000231664674185006" "0.172053236470185"
```

```
hazardratiotable$coef <- round(as.numeric(hazardratiotable$coef),digits = 3)
hazardratiotable$expcoef <- round(as.numeric(hazardratiotable$expcoef),digits = 3)
hazardratiotable$lowerCI <- round(as.numeric(hazardratiotable$lowerCI),digits = 3)
hazardratiotable$upperCI <- round(as.numeric(hazardratiotable$upperCI),digits = 3)
hazardratiotable$pval <- round(as.numeric(hazardratiotable$pval),digits = 3)
print(hazardratiotable)
```

```
##   Index                       Label   coef expcoef lowerCI upperCI  pval
## 1     1                  Tempscaled  0.133   1.142   1.046   1.248 0.003
## 2     2                   Agescaled  0.119   1.126   1.013   1.252 0.028
## 3     3 strata(egg_binary)Laid eggs -0.366   0.693   0.571   0.843 0.000
## 5     4        Tempscaled:Agescaled -0.063   0.938   0.857   1.028 0.172
```

```
hazardratiotable$Label <- c("Temperature","Age","Laid eggs","Temperature:Age")

print(hazardratiotable)
```

```
##   Index           Label   coef expcoef lowerCI upperCI  pval
## 1     1     Temperature  0.133   1.142   1.046   1.248 0.003
## 2     2             Age  0.119   1.126   1.013   1.252 0.028
## 3     3       Laid eggs -0.366   0.693   0.571   0.843 0.000
## 5     4 Temperature:Age -0.063   0.938   0.857   1.028 0.172
```

```
plot1 <- ggplot(hazardratiotable, aes(y = Index, x = expcoef)) +
  geom_point(shape = 18, size =4) +
  geom_errorbarh(aes(xmin = lowerCI, xmax = upperCI), height = 0.25) +
  geom_vline(xintercept = 1, color = "gray", linetype = "dashed", cex = 1, alpha = 0.5) +
  scale_y_continuous(name = "",breaks = 1:4, labels = hazardratiotable$Label,  trans = "reverse") +
  scale_x_continuous(name = "Hazard Ratio (95% CI)", limits=c(0.5,1.3)) +
  xlab("Hazard Ratio (95% CI)") +
  ylab(" ") +
  theme_pubr() +
  theme(panel.border = element_blank(),
        panel.background = element_blank(),
        panel.grid.major = element_blank(),
        panel.grid.minor = element_blank(),
        axis.line = element_line(colour = "black"),
        axis.text.y = element_text(size = 12, colour = "black"),
        axis.text.x.bottom = element_text(size = 12, colour = "black"),
        axis.title.x = element_text(size = 12, colour = "black"))
plot1
```

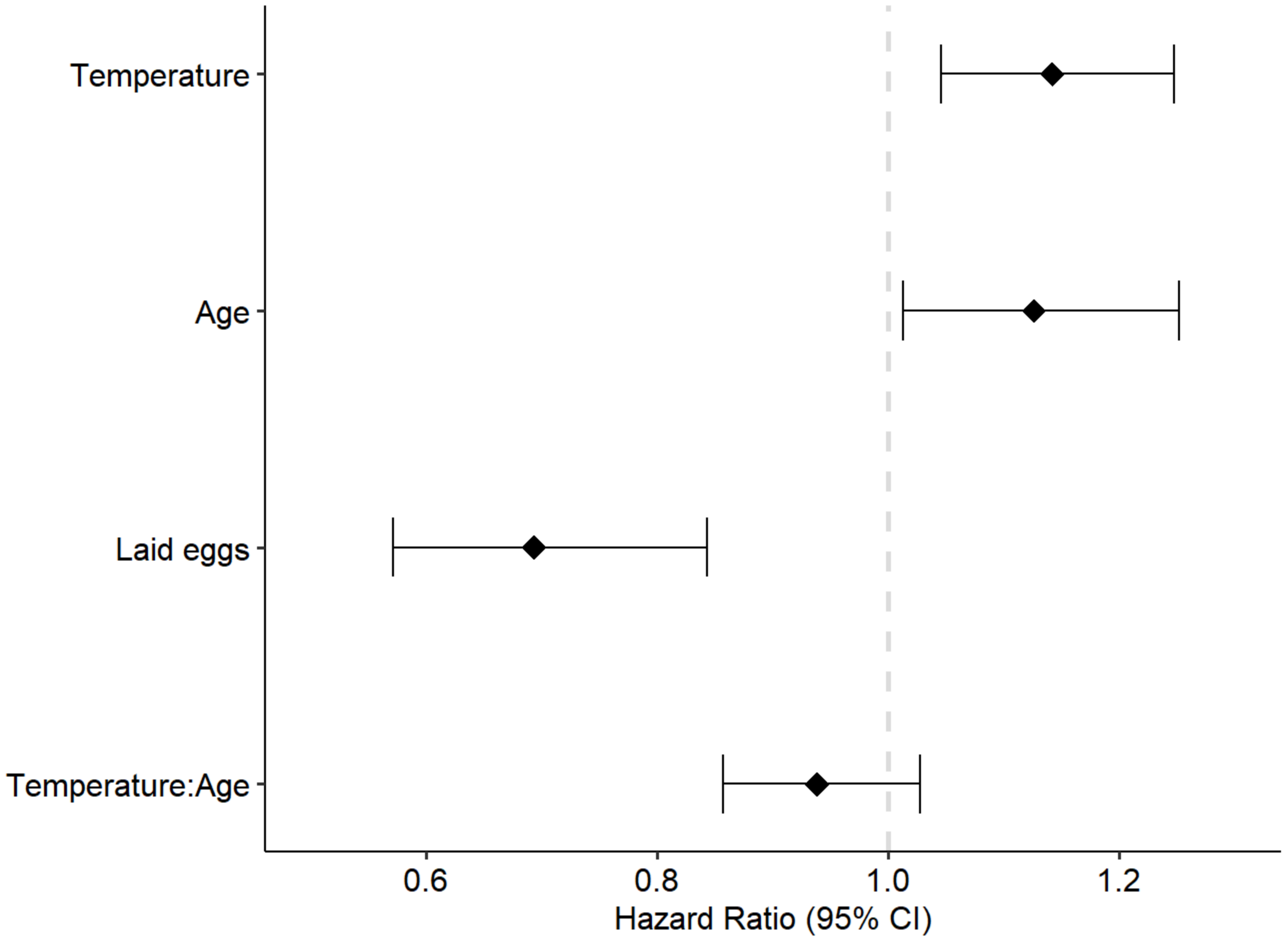

```r
## Create the table-base pallete
table_base <- ggplot(hazardratiotable, aes(y=Label)) +
  ylab(NULL) + xlab("  ") +
  theme(plot.title = element_text(hjust = 0.5, size=12),
        axis.text.x = element_text(color="white", hjust = -3, size = 25), ## This is used to help with alignment
        axis.line = element_blank(),
        axis.text.y = element_blank(),
        axis.ticks = element_blank(),
        axis.title.y = element_blank(),
        legend.position = "none",
        panel.background = element_blank(),
        panel.border = element_blank(),
        panel.grid.major = element_blank(),
        panel.grid.minor = element_blank(),
        plot.background = element_blank())

## HR point estimate table
tab1 <- table_base +
  labs(title = "space") +
  geom_text(aes(y = rev(Index), x = 1, label = sprintf("%0.1f", round(expcoef, digits = 1))), size = 4) + ## decimal places
  ggtitle("HR")
tab1
```

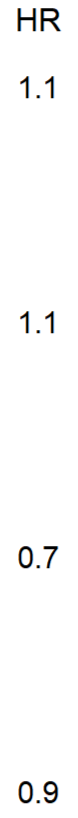

```
#pval
tab3 <- table_base +
  geom_text(aes(y = rev(Index), x = 1, label = pval), size = 4) +
  ggtitle("P value")
tab3
```

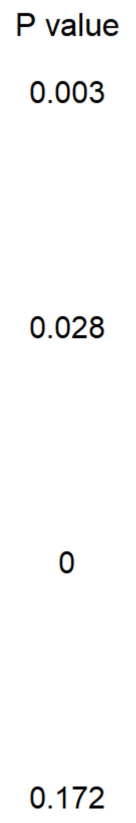


```
## Merge tables with plot
library(gridExtra)
lay <-  matrix(c(1,1,1,1,1,1,1,1,1,1,1,1,1,1,1,2,2,2), nrow = 1)
bucketsurvival_forest <- grid.arrange(plot1, tab3,layout_matrix = lay)
```

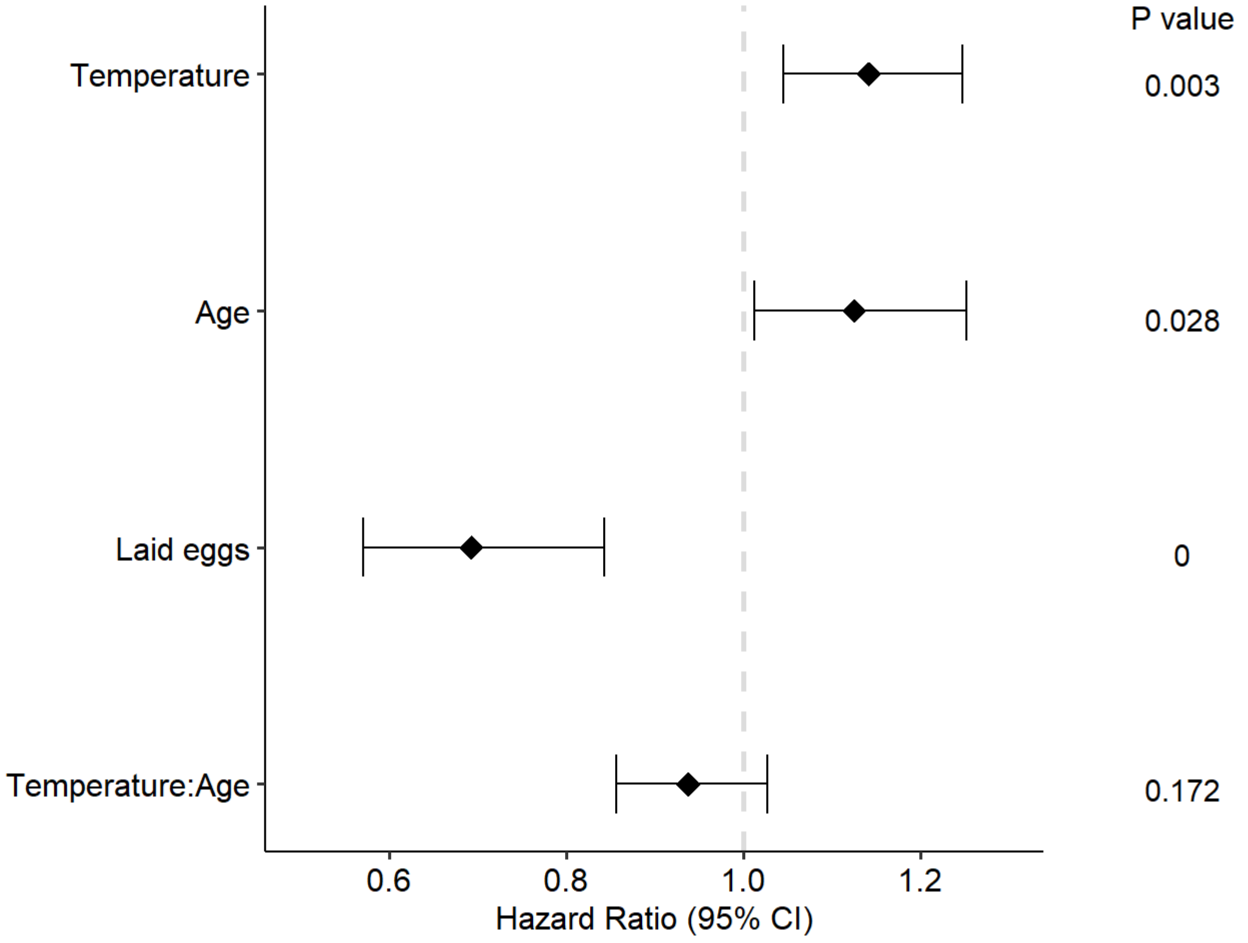


```
ggsave("Oviposition_survival/firstBM_hazardplot_eggbinary.pdf",bucketsurvival_forest,dpi=600,width = 7,heig
ht=5,units="in")
ggsave("Oviposition_survival/firstBM_hazardplot_eggbinary.png",bucketsurvival_forest,dpi=600,width = 7,heig
ht=5,units="in")
write_xlsx(hazardratiotable,"Oviposition_survival/firstBM_survival_hazardratiotable_eggbinary.xlsx")
```

Code for Figure 9 - combined Fig 7 and Fig 8 survival data

Comparing survival of oviposition site deprived mosquitoes, mosquitoes that have access to oviposition site and lay eggs, and mosquitoes that have access to oviposition site and do not lay eggs.

Import data and clean it up

```
# clear existing workspace
rm(list = ls(all = TRUE))
graphics.off()
shell("cls")

#set wd to your project folder
getwd() #check working directory
```

```
## [1] "C:/Users/linzm/OneDrive - Vanderbilt/Hillyer_Lab/Blood_feeding_project/Bloodfeeding"
```

```
#####################
#load libraries needed:
library(readxl)
library(writexl)
library(ggplot2)
library(dplyr)
library(tidyverse)
library(rstatix)
library(car)
library(ggpubr)
library(emmeans)
library(survival)
library(ggsurvfit)
library(survminer)

#######################################################
#import the data and clean it up:

Fecundity_data <- read_xlsx("SupplementaryData1_RawData.xlsx",
                             sheet = "Figs4-9")
Fecundity_data <- as.data.frame(Fecundity_data)
str(Fecundity_data)
```

```
## 'data.frame':    849 obs. of  18 variables:
##  $ ID_overall              : num  1 2 3 4 5 6 7 8 9 10 ...
##  $ Temperature             : num  32 32 32 32 32 32 32 32 32 32 ...
##  $ Age                     : num  3 3 3 3 3 3 3 3 3 3 ...
##  $ ID_per_group            : num  1 2 3 4 5 6 7 8 9 10 ...
##  $ Trial_start_date        : POSIXct, format: "2024-02-13" "2024-02-13" ...
##  $ Trial_number            : num  1 1 1 1 1 1 1 1 1 1 ...
##  $ BM1_Date                : POSIXct, format: "2024-02-13" "2024-02-13" ...
##  $ Age_of_BM               : num  3 3 3 3 3 3 3 3 3 3 ...
##  $ Bloodmeal_number        : num  1 1 1 1 1 1 1 1 1 1 ...
##  $ Oviposition_positive(y/n): chr  "Y" "Y" "Y" "Y" ...
##  $ Eggs_day3               : chr  "0" "0" "0" "0" ...
##  $ Eggs_day4               : chr  "0" "0" "0" "0" ...
##  $ Larvae_day4             : chr  "0" "0" "0" "0" ...
##  $ Surv_to_eggs(y/n)       : chr  "Y" "Y" "Y" "Y" ...
##  $ Surv_to_larvae(y/n)     : chr  "Y" "Y" "Y" "Y" ...
##  $ Date_of_death           : POSIXct, format: "2024-03-01" "2024-03-07" ...
##  $ Censor                  : num  1 1 1 1 1 1 1 1 1 1 ...
##  $ Notes                   : chr  NA NA NA NA ...
```

```
head(Fecundity_data)
```

```
##   ID_overall Temperature Age ID_per_group Trial_start_date Trial_number
## 1          1          32   3            1       2024-02-13            1
## 2          2          32   3            2       2024-02-13            1
## 3          3          32   3            3       2024-02-13            1
## 4          4          32   3            4       2024-02-13            1
## 5          5          32   3            5       2024-02-13            1
## 6          6          32   3            6       2024-02-13            1
##     BM1_Date Age_of_BM Bloodmeal_number Oviposition_positive(y/n) Eggs_day3
## 1 2024-02-13         3                1                         Y         0
## 2 2024-02-13         3                1                         Y         0
## 3 2024-02-13         3                1                         Y         0
## 4 2024-02-13         3                1                         Y         0
## 5 2024-02-13         3                1                         Y         0
## 6 2024-02-13         3                1                         Y        14
##   Eggs_day4 Larvae_day4 Surv_to_eggs(y/n) Surv_to_larvae(y/n) Date_of_death
## 1         0           0                 Y                   Y    2024-03-01
## 2         0           0                 Y                   Y    2024-03-07
## 3         0           0                 Y                   Y    2024-03-02
## 4         0           0                 Y                   Y    2024-02-26
## 5         0           0                 Y                   Y    2024-02-28
## 6        14           0                 Y                   Y    2024-03-02
##   Censor Notes
## 1      1  <NA>
## 2      1  <NA>
## 3      1  <NA>
## 4      1  <NA>
## 5      1  <NA>
## 6      1  <NA>
```

```
Fecundity_data_numeric <- Fecundity_data

#variables of interest:

Fecundity_data$Temperature <- as.factor(Fecundity_data$Temperature)
Fecundity_data$Age <- as.factor(Fecundity_data$Age)
Fecundity_data$Age_of_BM <- as.numeric(Fecundity_data$Age_of_BM)
Fecundity_data$Bloodmeal_number <- as.factor(Fecundity_data$Bloodmeal_number)

Fecundity_data$Eggs_day3 <- as.numeric(Fecundity_data$Eggs_day3)
```

```
## Warning: NAs introduced by coercion
```

```
Fecundity_data$Eggs_day4 <- as.numeric(Fecundity_data$Eggs_day4)
```

```
## Warning: NAs introduced by coercion
```

```
Fecundity_data$Larvae_day4 <- as.numeric(Fecundity_data$Larvae_day4)
```

```
## Warning: NAs introduced by coercion
```

```
Fecundity_data$Oviposition_positive <- as.factor(Fecundity_data$Oviposition_positive)
Fecundity_data$`Surv_to_eggs(y/n)` <- as.factor(Fecundity_data$`Surv_to_eggs(y/n)`)
Fecundity_data$`Surv_to_larvae(y/n)` <- as.factor(Fecundity_data$`Surv_to_larvae(y/n)`)

str(Fecundity_data)
```

```
## 'data.frame':    849 obs. of  19 variables:
##  $ ID_overall              : num  1 2 3 4 5 6 7 8 9 10 ...
##  $ Temperature             : Factor w/ 3 levels "27","30","32": 3 3 3 3 3 3 3 3 3 3 ...
##  $ Age                     : Factor w/ 4 levels "3","5","10","15": 1 1 1 1 1 1 1 1 1 1 ...
##  $ ID_per_group            : num  1 2 3 4 5 6 7 8 9 10 ...
##  $ Trial_start_date        : POSIXct, format: "2024-02-13" "2024-02-13" ...
##  $ Trial_number            : num  1 1 1 1 1 1 1 1 1 1 ...
##  $ BM1_Date                : POSIXct, format: "2024-02-13" "2024-02-13" ...
##  $ Age_of_BM               : num  3 3 3 3 3 3 3 3 3 3 ...
##  $ Bloodmeal_number        : Factor w/ 1 level "1": 1 1 1 1 1 1 1 1 1 1 ...
##  $ Oviposition_positive(y/n): chr  "Y" "Y" "Y" "Y" ...
##  $ Eggs_day3               : num  0 0 0 0 0 14 NA NA NA NA ...
##  $ Eggs_day4               : num  0 0 0 0 0 14 NA NA NA NA ...
##  $ Larvae_day4             : num  0 0 0 0 0 0 0 0 NA NA ...
##  $ Surv_to_eggs(y/n)       : Factor w/ 3 levels "N","NA","Y": 3 3 3 3 3 3 1 1 3 3 ...
##  $ Surv_to_larvae(y/n)     : Factor w/ 3 levels "N","NA","Y": 3 3 3 3 3 3 2 2 3 3 ...
##  $ Date_of_death           : POSIXct, format: "2024-03-01" "2024-03-07" ...
##  $ Censor                  : num  1 1 1 1 1 1 1 1 1 1 ...
##  $ Notes                   : chr  NA NA NA NA ...
##  $ Oviposition_positive    : Factor w/ 2 levels "N","Y": 2 2 2 2 2 2 2 2 1 1 ...
```

```
Fecundity_data <- subset(Fecundity_data, Censor == 1) #get rid of mosquitoes censored out by experimental e
rror (get rid of 0 values; 1 = died naturally)

#calculate the total eggs laid per mosquito:

#need to subtract to find ones only laid on day 4 (exclude day 3 eggs)
Fecundity_data$Eggs_day4 <- (Fecundity_data$Eggs_day4)-(Fecundity_data$Eggs_day3)

#replace negative eggs day 4 values with zero (assume miscounted/eggs degraded and no new eggs laid)
for (row in 1:nrow(Fecundity_data)){
  if (is.na(Fecundity_data$Eggs_day4[row])){
    Fecundity_data$Eggs_day4[row] <- NA #keep NA values
  } else if ((Fecundity_data$Eggs_day4[row] <= 0)){
    Fecundity_data$Eggs_day4[row] <- 0
  }
}

#total eggs addition:
Fecundity_data$total_eggs <- (Fecundity_data$Eggs_day3)+(Fecundity_data$Eggs_day4)

#percents:
Fecundity_data$Percent_eggs_day3 <- (Fecundity_data$Eggs_day3) / (Fecundity_data$total_eggs)
Fecundity_data$Percent_eggs_day4 <- (Fecundity_data$Eggs_day4) / (Fecundity_data$total_eggs)

# decide if each mosquito laid eggs and on what day
#day 3
for (row in 1:nrow(Fecundity_data)){
  if(!is.na(Fecundity_data$Eggs_day3[row])){
    if (Fecundity_data$Eggs_day3[row] > 0){
      Fecundity_data$egg_binary[row] = 1
      Fecundity_data$egg_binary_day3[row] = 1
    }
    else {
      Fecundity_data$egg_binary[row] = 0
      Fecundity_data$egg_binary_day3[row] = 0
    }
  }
  else if (is.na(Fecundity_data$Eggs_day3[row])){
    Fecundity_data$egg_binary[row] = NA
    Fecundity_data$egg_binary_day3[row] = NA
  }
}

#day 4
for (row in 1:nrow(Fecundity_data)){
  if(!is.na(Fecundity_data$Eggs_day4[row])){
    if (Fecundity_data$Eggs_day4[row] >0){
      Fecundity_data$egg_binary[row] = 1
      Fecundity_data$egg_binary_day4[row] = 1
    }
    else{
      Fecundity_data$egg_binary_day4[row] = 0
    }
  }
  else if (is.na(Fecundity_data$Eggs_day4[row])){
    Fecundity_data$egg_binary_day4[row] = NA
```

```
  }
}

# decide if each mosquito had larvae
for (row in 1:nrow(Fecundity_data)){
  if(!is.na(Fecundity_data$Larvae_day4[row])){
    if (Fecundity_data$Larvae_day4[row] >0){
      Fecundity_data$larvae_binary[row] = 1
    }
    else{
      Fecundity_data$larvae_binary[row] = 0
    }
  }
  else{
    Fecundity_data$larvae_binary[row] = NA
  }
}

#percents:
Fecundity_data$Percent_eggshatchedtolarv <- (Fecundity_data$Larvae_day4) / (Fecundity_data$Eggs_day3)

#survival:
#calculate:
Fecundity_data$Days_to_death_post_BM <- Fecundity_data$Date_of_death - Fecundity_data$Trial_start_date
Fecundity_data$Age_of_death <- Fecundity_data$Age_of_BM + Fecundity_data$Days_to_death_post_BM

Fecundity_data <-
  Fecundity_data %>%
  mutate(
    Age_of_BM_days = as.difftime(Age_of_BM, unit="days")
  )
Fecundity_data$Date_of_eclosion <- Fecundity_data$Trial_start_date - (Fecundity_data$Age_of_BM_days)

library(lubridate)
Fecundity_data <-
  Fecundity_data %>%
  mutate(
    days_alive_post_BM = as.duration(Trial_start_date %--% Date_of_death) / ddays(1),
    days_alive_post_eclosion = as.duration(Date_of_eclosion %--% Date_of_death) / ddays(1),
  )

str(Fecundity_data)
```

```
## 'data.frame':    842 obs. of  33 variables:
##  $ ID_overall               : num  1 2 3 4 5 6 7 8 9 10 ...
##  $ Temperature              : Factor w/ 3 levels "27","30","32": 3 3 3 3 3 3 3 3 3 3 ...
##  $ Age                      : Factor w/ 4 levels "3","5","10","15": 1 1 1 1 1 1 1 1 1 1 ...
##  $ ID_per_group             : num  1 2 3 4 5 6 7 8 9 10 ...
##  $ Trial_start_date         : POSIXct, format: "2024-02-13" "2024-02-13" ...
##  $ Trial_number             : num  1 1 1 1 1 1 1 1 1 1 ...
##  $ BM1_Date                 : POSIXct, format: "2024-02-13" "2024-02-13" ...
##  $ Age_of_BM                : num  3 3 3 3 3 3 3 3 3 3 ...
##  $ Bloodmeal_number         : Factor w/ 1 level "1": 1 1 1 1 1 1 1 1 1 1 ...
##  $ Oviposition_positive(y/n): chr  "Y" "Y" "Y" "Y" ...
##  $ Eggs_day3                : num  0 0 0 0 0 14 NA NA NA NA ...
##  $ Eggs_day4                : num  0 0 0 0 0 0 NA NA NA NA ...
##  $ Larvae_day4              : num  0 0 0 0 0 0 0 0 NA NA ...
##  $ Surv_to_eggs(y/n)        : Factor w/ 3 levels "N","NA","Y": 3 3 3 3 3 3 1 1 3 3 ...
##  $ Surv_to_larvae(y/n)      : Factor w/ 3 levels "N","NA","Y": 3 3 3 3 3 3 2 2 3 3 ...
##  $ Date_of_death            : POSIXct, format: "2024-03-01" "2024-03-07" ...
##  $ Censor                   : num  1 1 1 1 1 1 1 1 1 1 ...
##  $ Notes                    : chr  NA NA NA NA ...
##  $ Oviposition_positive     : Factor w/ 2 levels "N","Y": 2 2 2 2 2 2 2 2 1 1 ...
##  $ total_eggs               : num  0 0 0 0 0 14 NA NA NA NA ...
##  $ Percent_eggs_day3        : num  NaN NaN NaN NaN NaN 1 NA NA NA NA ...
##  $ Percent_eggs_day4        : num  NaN NaN NaN NaN NaN 0 NA NA NA NA ...
##  $ egg_binary               : num  0 0 0 0 0 1 NA NA NA NA ...
##  $ egg_binary_day3          : num  0 0 0 0 0 1 NA NA NA NA ...
##  $ egg_binary_day4          : num  0 0 0 0 0 0 NA NA NA NA ...
##  $ larvae_binary            : num  0 0 0 0 0 0 0 0 NA NA ...
##  $ Percent_eggshatchedtolarv: num  NaN NaN NaN NaN NaN 0 NA NA NA NA ...
##  $ Days_to_death_post_BM    : 'difftime' num  17 23 18 13 ...
##   ..- attr(*, "units")= chr "days"
##  $ Age_of_death             : 'difftime' num  20 26 21 16 ...
##   ..- attr(*, "units")= chr "days"
##  $ Age_of_BM_days           : 'difftime' num  3 3 3 3 ...
##   ..- attr(*, "units")= chr "days"
##  $ Date_of_eclosion         : POSIXct, format: "2024-02-10" "2024-02-10" ...
##  $ days_alive_post_BM       : num  17 23 18 13 15 18 3 3 12 11 ...
##  $ days_alive_post_eclosion : num  20 26 21 16 18 21 6 6 15 14 ...
```

```
Fecundity_data_firstBM <- Fecundity_data
```

Plot survival curves

```
library(survival)
# Plot survival curves
#labels first
Fecundity_data_firstBM$group <- paste(Fecundity_data_firstBM$Age,Fecundity_data_firstBM$Temperature,sep
="_")
Fecundity_data_firstBM$ovigroup <- paste(Fecundity_data_firstBM$Oviposition_positive,Fecundity_data_firstBM
$egg_binary,sep="_")
Fecundity_data_firstBM <- subset(Fecundity_data_firstBM,Fecundity_data_firstBM$ovigroup != "Y_NA")

Fecundity_data_firstBM$Age <- factor(Fecundity_data_firstBM$Age,
                                     labels = c("3 days","5 days","10 days","15 days"))
Fecundity_data_firstBM$Temperature <- factor(Fecundity_data_firstBM$Temperature,
                                             labels = c("27˚C","30˚C","32˚C"))



str(Fecundity_data_firstBM) #make sure dates are dates
```

```
## 'data.frame':	798 obs. of  35 variables:
##  $ ID_overall               : num  1 2 3 4 5 6 9 10 11 12 ...
##  $ Temperature              : Factor w/ 3 levels "27˚C","30˚C",..: 3 3 3 3 3 3 3 3 1 1 ...
##  $ Age                      : Factor w/ 4 levels "3 days","5 days",..: 1 1 1 1 1 1 1 1 1 1 ...
##  $ ID_per_group             : num  1 2 3 4 5 6 9 10 1 2 ...
##  $ Trial_start_date         : POSIXct, format: "2024-02-13" "2024-02-13" ...
##  $ Trial_number             : num  1 1 1 1 1 1 1 1 1 1 ...
##  $ BM1_Date                 : POSIXct, format: "2024-02-13" "2024-02-13" ...
##  $ Age_of_BM                : num  3 3 3 3 3 3 3 3 3 3 ...
##  $ Bloodmeal_number         : Factor w/ 1 level "1": 1 1 1 1 1 1 1 1 1 1 ...
##  $ Oviposition_positive(y/n): chr  "Y" "Y" "Y" "Y" ...
##  $ Eggs_day3                : num  0 0 0 0 0 14 NA NA 0 72 ...
##  $ Eggs_day4                : num  0 0 0 0 0 0 NA NA 0 10 ...
##  $ Larvae_day4              : num  0 0 0 0 0 0 NA NA NA 0 ...
##  $ Surv_to_eggs(y/n)        : Factor w/ 3 levels "N","NA","Y": 3 3 3 3 3 3 3 3 3 3 ...
##  $ Surv_to_larvae(y/n)      : Factor w/ 3 levels "N","NA","Y": 3 3 3 3 3 3 3 3 3 3 ...
##  $ Date_of_death            : POSIXct, format: "2024-03-01" "2024-03-07" ...
##  $ Censor                   : num  1 1 1 1 1 1 1 1 1 1 ...
##  $ Notes                    : chr  NA NA NA NA ...
##  $ Oviposition_positive     : Factor w/ 2 levels "N","Y": 2 2 2 2 2 2 1 1 2 2 ...
##  $ total_eggs               : num  0 0 0 0 0 14 NA NA 0 82 ...
##  $ Percent_eggs_day3        : num  NaN NaN NaN NaN NaN ...
##  $ Percent_eggs_day4        : num  NaN NaN NaN NaN NaN ...
##  $ egg_binary               : num  0 0 0 0 0 1 NA NA 0 1 ...
##  $ egg_binary_day3          : num  0 0 0 0 0 1 NA NA 0 1 ...
##  $ egg_binary_day4          : num  0 0 0 0 0 0 NA NA 0 1 ...
##  $ larvae_binary            : num  0 0 0 0 0 0 NA NA NA 0 ...
##  $ Percent_eggshatchedtolarv: num  NaN NaN NaN NaN NaN 0 NA NA NA 0 ...
##  $ Days_to_death_post_BM    : 'difftime' num  17 23 18 13 ...
##   ..- attr(*, "units")= chr "days"
##  $ Age_of_death             : 'difftime' num  20 26 21 16 ...
##   ..- attr(*, "units")= chr "days"
##  $ Age_of_BM_days           : 'difftime' num  3 3 3 3 ...
##   ..- attr(*, "units")= chr "days"
##  $ Date_of_eclosion         : POSIXct, format: "2024-02-10" "2024-02-10" ...
##  $ days_alive_post_BM       : num  17 23 18 13 15 18 12 11 12 12 ...
##  $ days_alive_post_eclosion : num  20 26 21 16 18 21 15 14 15 15 ...
##  $ group                    : chr  "3_32" "3_32" "3_32" "3_32" ...
##  $ ovigroup                 : chr  "Y_0" "Y_0" "Y_0" "Y_0" ...
```

```
Fecundity_data_firstBM$start_time <- (Fecundity_data_firstBM$Age_of_BM +1) #start monitoring the day after
BM
Fecundity_data_firstBM$stop_time <- (Fecundity_data_firstBM$days_alive_post_eclosion+1) #age of death + 1 #
aod can't be sooner than start
Fecundity_data_firstBM$stopminusstart <- Fecundity_data_firstBM$stop_time - Fecundity_data_firstBM$start_ti
me

s1 <- survfit2(Surv(stopminusstart, Censor) ~ ovigroup,
               data = Fecundity_data_firstBM)

ggsurv <- ggsurvplot(s1, conf.int = TRUE, color="ovigroup",palette = c("lightblue","lightgreen","darkgree
n"),
                     ggtheme = theme_pubr(),surv.median.line = "v",confint=TRUE)

s1table <- as.data.frame(ggsurv$data.survplot)

combined <- ggsurv$plot +
  theme_pubr() +
  theme(legend.position = "bottom")+
  #facet_grid(~ ovigroup)+
  ylab(expression("BM1 Survival probability"))+
  xlab("Time (Days post bloodmeal)") +
  theme(panel.background = element_rect(fill = NA, color = "black"))+
  theme(panel.spacing = unit(0.6, "lines"))
combined
```

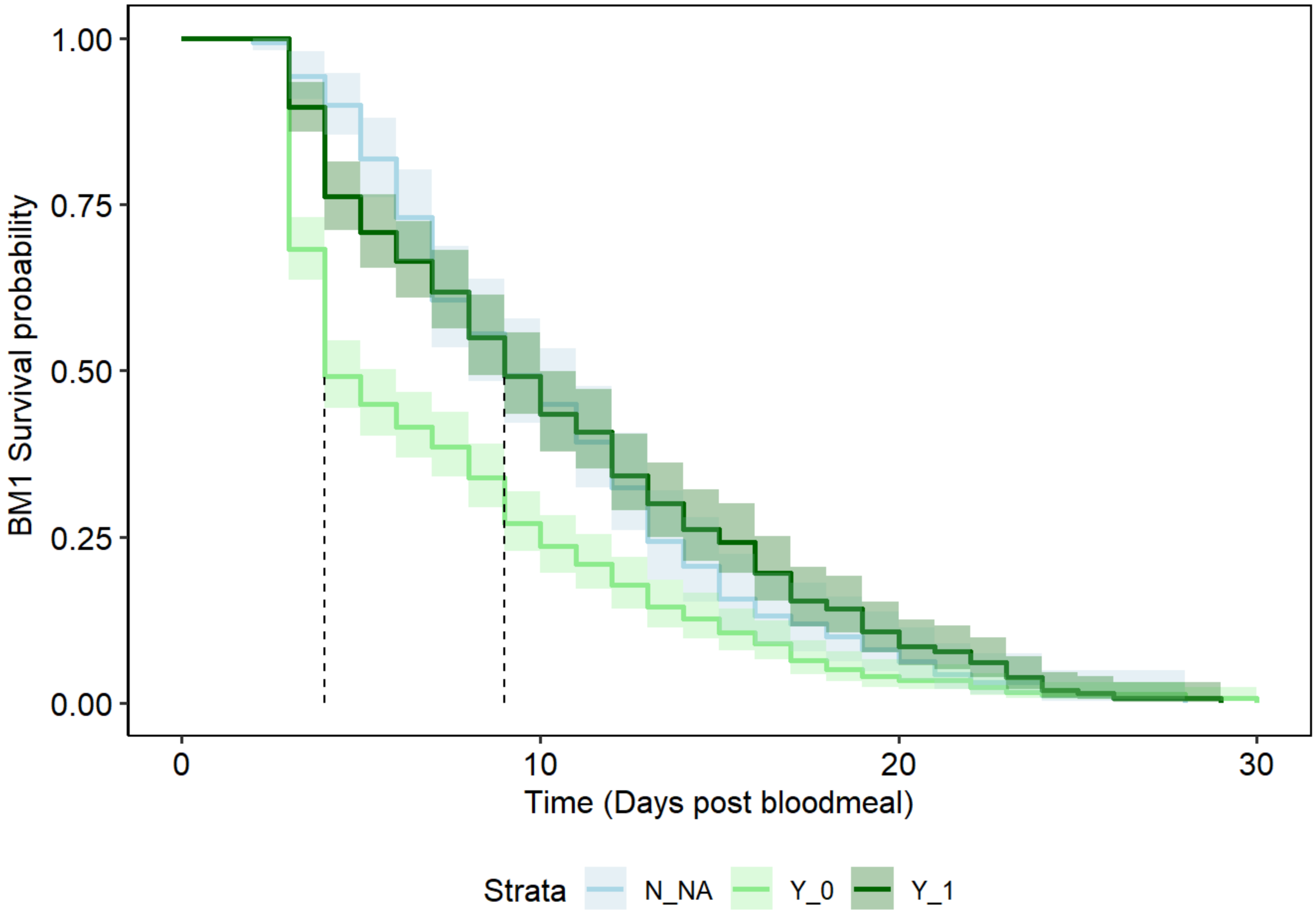

```
ggsave(combined,filename = "Oviposition_survival/firstBM_survival_oviposition_positive_vs_negative_eggsYoN_
stopminusstart.pdf",width = 5, height = 5, units = "in", dpi = 600)

sink("Oviposition_survival/firstBM_oviposvsnegandegglaying_survival_mediansurvivaltime_STOPMINUSSTART.txt")
print(s1,print.rmean = TRUE)
sink()
```

Analysis for hazard ratios and making forest plot

```
Fecundity_data_firstBM_numeric <- Fecundity_data_firstBM

str(Fecundity_data_firstBM_numeric)
```

```
## 'data.frame':	798 obs. of  38 variables:
##  $ ID_overall               : num  1 2 3 4 5 6 9 10 11 12 ...
##  $ Temperature              : Factor w/ 3 levels "27°C","30°C",..: 3 3 3 3 3 3 3 3 1 1 ...
##  $ Age                      : Factor w/ 4 levels "3 days","5 days",..: 1 1 1 1 1 1 1 1 1 1 ...
##  $ ID_per_group             : num  1 2 3 4 5 6 9 10 1 2 ...
##  $ Trial_start_date         : POSIXct, format: "2024-02-13" "2024-02-13" ...
##  $ Trial_number             : num  1 1 1 1 1 1 1 1 1 1 ...
##  $ BM1_Date                 : POSIXct, format: "2024-02-13" "2024-02-13" ...
##  $ Age_of_BM                : num  3 3 3 3 3 3 3 3 3 3 ...
##  $ Bloodmeal_number         : Factor w/ 1 level "1": 1 1 1 1 1 1 1 1 1 1 ...
##  $ Oviposition_positive(y/n): chr  "Y" "Y" "Y" "Y" ...
##  $ Eggs_day3                : num  0 0 0 0 0 14 NA NA 0 72 ...
##  $ Eggs_day4                : num  0 0 0 0 0 0 NA NA 0 10 ...
##  $ Larvae_day4              : num  0 0 0 0 0 0 NA NA NA 0 ...
##  $ Surv_to_eggs(y/n)        : Factor w/ 3 levels "N","NA","Y": 3 3 3 3 3 3 3 3 3 3 ...
##  $ Surv_to_larvae(y/n)      : Factor w/ 3 levels "N","NA","Y": 3 3 3 3 3 3 3 3 3 3 ...
##  $ Date_of_death            : POSIXct, format: "2024-03-01" "2024-03-07" ...
##  $ Censor                   : num  1 1 1 1 1 1 1 1 1 1 ...
##  $ Notes                    : chr  NA NA NA NA ...
##  $ Oviposition_positive     : Factor w/ 2 levels "N","Y": 2 2 2 2 2 2 1 1 2 2 ...
##  $ total_eggs               : num  0 0 0 0 0 14 NA NA 0 82 ...
##  $ Percent_eggs_day3        : num  NaN NaN NaN NaN NaN ...
##  $ Percent_eggs_day4        : num  NaN NaN NaN NaN NaN ...
##  $ egg_binary               : num  0 0 0 0 0 1 NA NA 0 1 ...
##  $ egg_binary_day3          : num  0 0 0 0 0 1 NA NA 0 1 ...
##  $ egg_binary_day4          : num  0 0 0 0 0 0 NA NA 0 1 ...
##  $ larvae_binary            : num  0 0 0 0 0 0 NA NA NA 0 ...
##  $ Percent_eggshatchedtolarv: num  NaN NaN NaN NaN NaN 0 NA NA NA 0 ...
##  $ Days_to_death_post_BM    : 'difftime' num  17 23 18 13 ...
##   ..- attr(*, "units")= chr "days"
##  $ Age_of_death             : 'difftime' num  20 26 21 16 ...
##   ..- attr(*, "units")= chr "days"
##  $ Age_of_BM_days           : 'difftime' num  3 3 3 3 ...
##   ..- attr(*, "units")= chr "days"
##  $ Date_of_eclosion         : POSIXct, format: "2024-02-10" "2024-02-10" ...
##  $ days_alive_post_BM       : num  17 23 18 13 15 18 12 11 12 12 ...
##  $ days_alive_post_eclosion : num  20 26 21 16 18 21 15 14 15 15 ...
##  $ group                    : chr  "3_32" "3_32" "3_32" "3_32" ...
##  $ ovigroup                 : chr  "Y_0" "Y_0" "Y_0" "Y_0" ...
##  $ start_time               : num  4 4 4 4 4 4 4 4 4 4 ...
##  $ stop_time                : num  21 27 22 17 19 22 16 15 16 16 ...
##  $ stopminusstart           : num  17 23 18 13 15 18 12 11 12 12 ...
```

```r
Fecundity_data_firstBM_numeric$Age <- factor(Fecundity_data_firstBM_numeric$Age,
                                             labels = c("3","5","10","15"))
Fecundity_data_firstBM_numeric$Temperature <- factor(Fecundity_data_firstBM_numeric$Temperature,
                                                     labels = c("27","30","32"))

#treat variables as numeric so hazard shows with every increase in age(days) or temp (degrees)
Fecundity_data_firstBM_numeric$Temperature <- as.character(Fecundity_data_firstBM_numeric$Temperature)
str(Fecundity_data_firstBM_numeric$Temperature)
```

```
##  chr [1:798] "32" "32" "32" "32" "32" "32" "32" "32" "27" "27" "27" "27" ...
```

```
Fecundity_data_firstBM_numeric$Temperature <- as.numeric(Fecundity_data_firstBM_numeric$Temperature)

Fecundity_data_firstBM_numeric$Age <- as.character(Fecundity_data_firstBM_numeric$Age)
str(Fecundity_data_firstBM_numeric$Age)
```

```
##  chr [1:798] "3" "3" "3" "3" "3" "3" "3" "3" "3" "3" "3" "3" "3" "3" "3" ...
```

```
Fecundity_data_firstBM_numeric$Age <- as.numeric(Fecundity_data_firstBM_numeric$Age)



Fecundity_data_firstBM_numeric$Trial_number <- as.factor(Fecundity_data_firstBM_numeric$Trial_number)

Fecundity_data_firstBM_numeric$Trial_start_date_factor <- as.factor(Fecundity_data_firstBM_numeric$Trial_st
art_date)
str(Fecundity_data_firstBM_numeric$Trial_start_date_factor)
```

```
##  Factor w/ 32 levels "2024-02-13","2024-02-19",..: 1 1 1 1 1 1 1 1 20 20 ...
```

```
str(Fecundity_data_firstBM_numeric)
```

```
## 'data.frame':	798 obs. of  39 variables:
##  $ ID_overall               : num  1 2 3 4 5 6 9 10 11 12 ...
##  $ Temperature              : num  32 32 32 32 32 32 32 32 27 27 ...
##  $ Age                      : num  3 3 3 3 3 3 3 3 3 3 ...
##  $ ID_per_group             : num  1 2 3 4 5 6 9 10 1 2 ...
##  $ Trial_start_date         : POSIXct, format: "2024-02-13" "2024-02-13" ...
##  $ Trial_number             : Factor w/ 5 levels "1","2","3","4",..: 1 1 1 1 1 1 1 1 1 1 ...
##  $ BM1_Date                 : POSIXct, format: "2024-02-13" "2024-02-13" ...
##  $ Age_of_BM                : num  3 3 3 3 3 3 3 3 3 3 ...
##  $ Bloodmeal_number         : Factor w/ 1 level "1": 1 1 1 1 1 1 1 1 1 1 ...
##  $ Oviposition_positive(y/n): chr  "Y" "Y" "Y" "Y" ...
##  $ Eggs_day3                : num  0 0 0 0 0 14 NA NA 0 72 ...
##  $ Eggs_day4                : num  0 0 0 0 0 0 NA NA 0 10 ...
##  $ Larvae_day4              : num  0 0 0 0 0 0 NA NA NA 0 ...
##  $ Surv_to_eggs(y/n)        : Factor w/ 3 levels "N","NA","Y": 3 3 3 3 3 3 3 3 3 3 ...
##  $ Surv_to_larvae(y/n)      : Factor w/ 3 levels "N","NA","Y": 3 3 3 3 3 3 3 3 3 3 ...
##  $ Date_of_death            : POSIXct, format: "2024-03-01" "2024-03-07" ...
##  $ Censor                   : num  1 1 1 1 1 1 1 1 1 1 ...
##  $ Notes                    : chr  NA NA NA NA ...
##  $ Oviposition_positive     : Factor w/ 2 levels "N","Y": 2 2 2 2 2 2 1 1 2 2 ...
##  $ total_eggs               : num  0 0 0 0 0 14 NA NA 0 82 ...
##  $ Percent_eggs_day3        : num  NaN NaN NaN NaN NaN ...
##  $ Percent_eggs_day4        : num  NaN NaN NaN NaN NaN ...
##  $ egg_binary               : num  0 0 0 0 0 1 NA NA 0 1 ...
##  $ egg_binary_day3          : num  0 0 0 0 0 1 NA NA 0 1 ...
##  $ egg_binary_day4          : num  0 0 0 0 0 0 NA NA 0 1 ...
##  $ larvae_binary            : num  0 0 0 0 0 0 NA NA NA 0 ...
##  $ Percent_eggshatchedtolarv: num  NaN NaN NaN NaN NaN 0 NA NA NA 0 ...
##  $ Days_to_death_post_BM    : 'difftime' num  17 23 18 13 ...
##   ..- attr(*, "units")= chr "days"
##  $ Age_of_death             : 'difftime' num  20 26 21 16 ...
##   ..- attr(*, "units")= chr "days"
##  $ Age_of_BM_days           : 'difftime' num  3 3 3 3 ...
##   ..- attr(*, "units")= chr "days"
##  $ Date_of_eclosion         : POSIXct, format: "2024-02-10" "2024-02-10" ...
##  $ days_alive_post_BM       : num  17 23 18 13 15 18 12 11 12 12 ...
##  $ days_alive_post_eclosion : num  20 26 21 16 18 21 15 14 15 15 ...
##  $ group                    : chr  "3_32" "3_32" "3_32" "3_32" ...
##  $ ovigroup                 : chr  "Y_0" "Y_0" "Y_0" "Y_0" ...
##  $ start_time               : num  4 4 4 4 4 4 4 4 4 4 ...
##  $ stop_time                : num  21 27 22 17 19 22 16 15 16 16 ...
##  $ stopminusstart           : num  17 23 18 13 15 18 12 11 12 12 ...
##  $ Trial_start_date_factor  : Factor w/ 32 levels "2024-02-13","2024-02-19",..: 1 1 1 1 1 1 1 1 20 20
...
```

```
Fecundity_data_firstBM_numeric$Trial_start_date_number <- as.numeric(Fecundity_data_firstBM_numeric$Trial_s
tart_date_factor)
str(Fecundity_data_firstBM_numeric)
```

```
## 'data.frame':    798 obs. of  40 variables:
##  $ ID_overall               : num  1 2 3 4 5 6 9 10 11 12 ...
##  $ Temperature              : num  32 32 32 32 32 32 32 32 27 27 ...
##  $ Age                      : num  3 3 3 3 3 3 3 3 3 3 ...
##  $ ID_per_group             : num  1 2 3 4 5 6 9 10 1 2 ...
##  $ Trial_start_date         : POSIXct, format: "2024-02-13" "2024-02-13" ...
##  $ Trial_number             : Factor w/ 5 levels "1","2","3","4",..: 1 1 1 1 1 1 1 1 1 1 ...
##  $ BM1_Date                 : POSIXct, format: "2024-02-13" "2024-02-13" ...
##  $ Age_of_BM                : num  3 3 3 3 3 3 3 3 3 3 ...
##  $ Bloodmeal_number         : Factor w/ 1 level "1": 1 1 1 1 1 1 1 1 1 1 ...
##  $ Oviposition_positive(y/n): chr  "Y" "Y" "Y" "Y" ...
##  $ Eggs_day3                : num  0 0 0 0 0 14 NA NA 0 72 ...
##  $ Eggs_day4                : num  0 0 0 0 0 0 NA NA 0 10 ...
##  $ Larvae_day4              : num  0 0 0 0 0 0 NA NA NA 0 ...
##  $ Surv_to_eggs(y/n)        : Factor w/ 3 levels "N","NA","Y": 3 3 3 3 3 3 3 3 3 3 ...
##  $ Surv_to_larvae(y/n)      : Factor w/ 3 levels "N","NA","Y": 3 3 3 3 3 3 3 3 3 3 ...
##  $ Date_of_death            : POSIXct, format: "2024-03-01" "2024-03-07" ...
##  $ Censor                   : num  1 1 1 1 1 1 1 1 1 1 ...
##  $ Notes                    : chr  NA NA NA NA ...
##  $ Oviposition_positive     : Factor w/ 2 levels "N","Y": 2 2 2 2 2 2 1 1 2 2 ...
##  $ total_eggs               : num  0 0 0 0 0 14 NA NA 0 82 ...
##  $ Percent_eggs_day3        : num  NaN NaN NaN NaN NaN ...
##  $ Percent_eggs_day4        : num  NaN NaN NaN NaN NaN ...
##  $ egg_binary               : num  0 0 0 0 0 1 NA NA 0 1 ...
##  $ egg_binary_day3          : num  0 0 0 0 0 1 NA NA 0 1 ...
##  $ egg_binary_day4          : num  0 0 0 0 0 0 NA NA 0 1 ...
##  $ larvae_binary            : num  0 0 0 0 0 0 NA NA NA 0 ...
##  $ Percent_eggshatchedtolarv: num  NaN NaN NaN NaN NaN 0 NA NA NA 0 ...
##  $ Days_to_death_post_BM    : 'difftime' num  17 23 18 13 ...
##   ..- attr(*, "units")= chr "days"
##  $ Age_of_death             : 'difftime' num  20 26 21 16 ...
##   ..- attr(*, "units")= chr "days"
##  $ Age_of_BM_days           : 'difftime' num  3 3 3 3 ...
##   ..- attr(*, "units")= chr "days"
##  $ Date_of_eclosion         : POSIXct, format: "2024-02-10" "2024-02-10" ...
##  $ days_alive_post_BM       : num  17 23 18 13 15 18 12 11 12 12 ...
##  $ days_alive_post_eclosion : num  20 26 21 16 18 21 15 14 15 15 ...
##  $ group                    : chr  "3_32" "3_32" "3_32" "3_32" ...
##  $ ovigroup                 : chr  "Y_0" "Y_0" "Y_0" "Y_0" ...
##  $ start_time               : num  4 4 4 4 4 4 4 4 4 4 ...
##  $ stop_time                : num  21 27 22 17 19 22 16 15 16 16 ...
##  $ stopminusstart           : num  17 23 18 13 15 18 12 11 12 12 ...
##  $ Trial_start_date_factor  : Factor w/ 32 levels "2024-02-13","2024-02-19",..: 1 1 1 1 1 1 1 1 20 20
...
##  $ Trial_start_date_number  : num  1 1 1 1 1 1 1 1 20 20 ...
```

```
Fecundity_data_firstBM_numeric$Tempscaled <- scale(Fecundity_data_firstBM_numeric$Temperature, center=TRUE,
scale=TRUE)
Fecundity_data_firstBM_numeric$Agescaled <- scale(Fecundity_data_firstBM_numeric$Age, center=TRUE, scale=TR
UE)
# HAZARD RATIOS FOR COMBINED OVIPOS AND EGG BINARY:

Fecundity_data_firstBM_numeric$ovigroup <- paste(Fecundity_data_firstBM_numeric$Oviposition_positive,Fecund
ity_data_firstBM_numeric$egg_binary,sep="_")
Fecundity_data_firstBM_numeric <- subset(Fecundity_data_firstBM_numeric,Fecundity_data_firstBM_numeric$ovig
roup != "Y_NA") #get rid of ones that did not survive to egg laying time

fit4 <- coxphw(Surv(start_time,stop_time, Censor) ~ Tempscaled*Agescaled +
                 strata(ovigroup)+
                 frailty(Trial_start_date_factor,distribution = "gaussian"),
               data =Fecundity_data_firstBM_numeric,
               template = "AHR")
summary(fit4)
```

```
## coxphw(formula = Surv(start_time, stop_time, Censor) ~ Tempscaled *
##     Agescaled + strata(ovigroup) + frailty(Trial_start_date_factor,
##     distribution = "gaussian"), data = Fecundity_data_firstBM_numeric,
##     template = "AHR")
##
## Model fitted by weighted estimation (AHR template)
##
##                                                                      coef
## Tempscaled                                                     0.15514794
## Agescaled                                                      0.04550146
## strata(ovigroup)Y_0                                            0.59855094
## strata(ovigroup)Y_1                                            0.22938890
## frailty(Trial_start_date_factor, distribution = "gaussian")  0.03839339
## Tempscaled:Agescaled                                          -0.09328129
##                                                                  se(coef)
## Tempscaled                                                    0.041646522
## Agescaled                                                     0.045426633
## strata(ovigroup)Y_0                                           0.097208648
## strata(ovigroup)Y_1                                           0.106828890
## frailty(Trial_start_date_factor, distribution = "gaussian") 0.005423386
## Tempscaled:Agescaled                                          0.040736419
##                                                               exp(coef)
## Tempscaled                                                    1.1678307
## Agescaled                                                     1.0465525
## strata(ovigroup)Y_0                                           1.8194804
## strata(ovigroup)Y_1                                           1.2578311
## frailty(Trial_start_date_factor, distribution = "gaussian") 1.0391399
## Tempscaled:Agescaled                                          0.9109372
##                                                               lower 0.95
## Tempscaled                                                     1.0762925
## Agescaled                                                      0.9574009
## strata(ovigroup)Y_0                                            1.5038455
## strata(ovigroup)Y_1                                            1.0202096
## frailty(Trial_start_date_factor, distribution = "gaussian")  1.0281528
## Tempscaled:Agescaled                                           0.8410340
##                                                               upper 0.95
## Tempscaled                                                     1.2671542
## Agescaled                                                      1.1440059
## strata(ovigroup)Y_0                                            2.2013623
## strata(ovigroup)Y_1                                            1.5507981
## frailty(Trial_start_date_factor, distribution = "gaussian")  1.0502445
## Tempscaled:Agescaled                                           0.9866505
##                                                                       z
## Tempscaled                                                     3.725352
## Agescaled                                                      1.001647
## strata(ovigroup)Y_0                                            6.157384
## strata(ovigroup)Y_1                                            2.147255
## frailty(Trial_start_date_factor, distribution = "gaussian")  7.079228
## Tempscaled:Agescaled                                          -2.289874
##                                                                         p
## Tempscaled                                                    1.950431e-04
## Agescaled                                                     3.165140e-01
## strata(ovigroup)Y_0                                           7.395654e-10
## strata(ovigroup)Y_1                                           3.177297e-02
## frailty(Trial_start_date_factor, distribution = "gaussian") 1.449618e-12
## Tempscaled:Agescaled                                          2.202859e-02
```

```
##
## Wald Chi-square = 129.2855 on 6  df  p = 0  n = 798
##
## Covariance-Matrix:
##                                                            Tempscaled
## Tempscaled                                               1.734433e-03
## Agescaled                                               -1.715416e-05
## strata(ovigroup)Y_0                                     -7.363566e-04
## strata(ovigroup)Y_1                                      5.089247e-04
## frailty(Trial_start_date_factor, distribution = "gaussian") -3.899091e-06
## Tempscaled:Agescaled                                    -3.381705e-04
##                                                             Agescaled
## Tempscaled                                              -1.715416e-05
## Agescaled                                                2.063579e-03
## strata(ovigroup)Y_0                                     -2.794056e-04
## strata(ovigroup)Y_1                                      3.940553e-04
## frailty(Trial_start_date_factor, distribution = "gaussian")  7.900875e-05
## Tempscaled:Agescaled                                    -4.926844e-04
##                                                         strata(ovigroup)Y_0
## Tempscaled                                                    -7.363566e-04
## Agescaled                                                     -2.794056e-04
## strata(ovigroup)Y_0                                            9.449521e-03
## strata(ovigroup)Y_1                                            5.456490e-03
## frailty(Trial_start_date_factor, distribution = "gaussian")        3.720318e-06
## Tempscaled:Agescaled                                           6.444622e-05
##                                                         strata(ovigroup)Y_1
## Tempscaled                                                     5.089247e-04
## Agescaled                                                      3.940553e-04
## strata(ovigroup)Y_0                                            5.456490e-03
## strata(ovigroup)Y_1                                            1.141241e-02
## frailty(Trial_start_date_factor, distribution = "gaussian")        6.922124e-05
## Tempscaled:Agescaled                                           1.436186e-04
##                                                         frailty(Trial_start_date_factor, distributio
n = "gaussian")
## Tempscaled
-3.899091e-06
## Agescaled
7.900875e-05
## strata(ovigroup)Y_0
3.720318e-06
## strata(ovigroup)Y_1
6.922124e-05
## frailty(Trial_start_date_factor, distribution = "gaussian")
2.941312e-05
## Tempscaled:Agescaled
-7.622946e-05
##                                                         Tempscaled:Agescaled
## Tempscaled                                                      -3.381705e-04
## Agescaled                                                       -4.926844e-04
## strata(ovigroup)Y_0                                              6.444622e-05
## strata(ovigroup)Y_1                                              1.436186e-04
## frailty(Trial_start_date_factor, distribution = "gaussian")         -7.622946e-05
## Tempscaled:Agescaled                                             1.659456e-03
##
## Generalized concordance probability:
##                                                         concordance prob.
```

```
## Tempscaled                                                              0.5387
## Agescaled                                                               0.5114
## strata(ovigroup)Y_0                                                     0.6453
## strata(ovigroup)Y_1                                                     0.5571
## frailty(Trial_start_date_factor, distribution = "gaussian")             0.5096
## Tempscaled:Agescaled                                                    0.4767
##                                                                 lower 0.95
## Tempscaled                                                          0.5184
## Agescaled                                                           0.4891
## strata(ovigroup)Y_0                                                 0.6006
## strata(ovigroup)Y_1                                                 0.5050
## frailty(Trial_start_date_factor, distribution = "gaussian")     0.5069
## Tempscaled:Agescaled                                                0.4568
##                                                                 upper 0.95
## Tempscaled                                                          0.5589
## Agescaled                                                           0.5336
## strata(ovigroup)Y_0                                                 0.6876
## strata(ovigroup)Y_1                                                 0.6080
## frailty(Trial_start_date_factor, distribution = "gaussian")     0.5123
## Tempscaled:Agescaled                                                0.4966
```

```
plot(fit4$dfbeta.resid) # resdiuals look best!
```

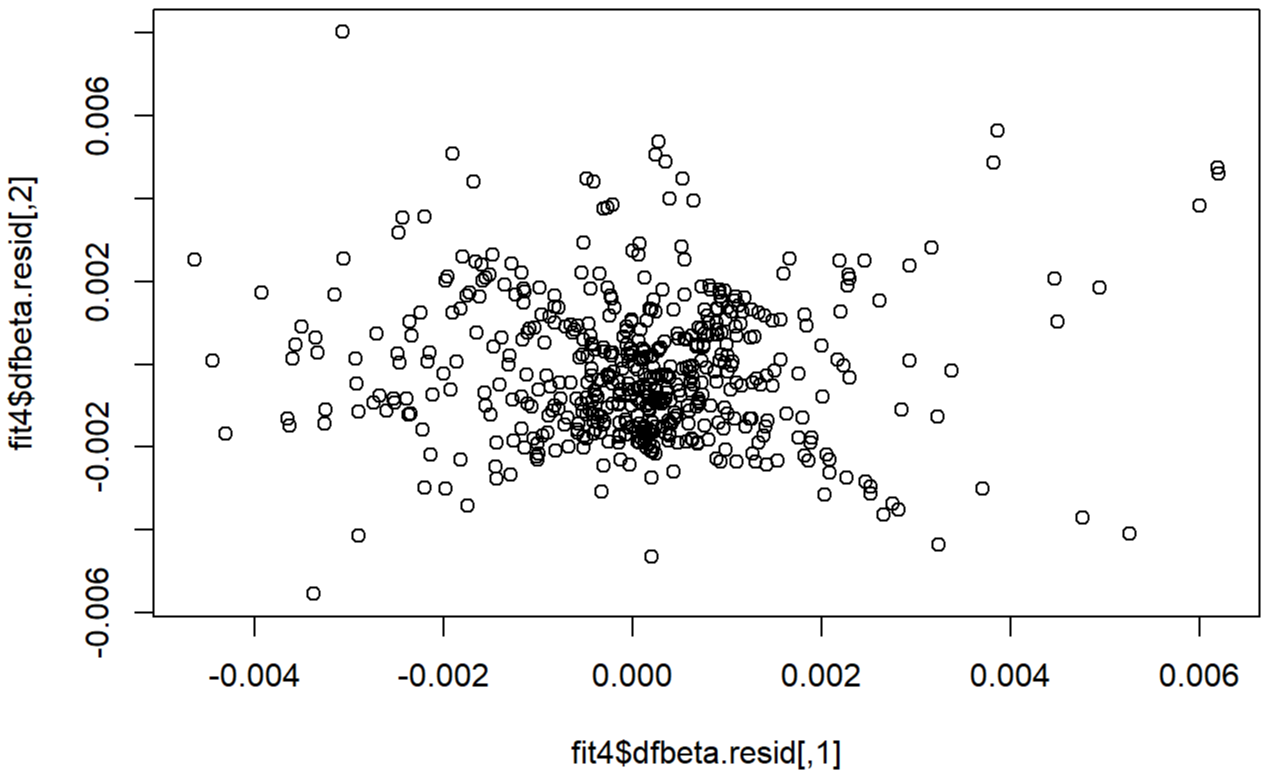


```
fit4
```

```
## coxphw(formula = Surv(start_time, stop_time, Censor) ~ Tempscaled *
##     Agescaled + strata(ovigroup) + frailty(Trial_start_date_factor,
##     distribution = "gaussian"), data = Fecundity_data_firstBM_numeric,
##     template = "AHR")
##
## Model fitted by weighted estimation (AHR template)
##
##                                                                    coef
## Tempscaled                                                   0.15514794
## Agescaled                                                    0.04550146
## strata(ovigroup)Y_0                                          0.59855094
## strata(ovigroup)Y_1                                          0.22938890
## frailty(Trial_start_date_factor, distribution = "gaussian")  0.03839339
## Tempscaled:Agescaled                                        -0.09328129
##                                                                se(coef)
## Tempscaled                                                  0.041646522
## Agescaled                                                   0.045426633
## strata(ovigroup)Y_0                                         0.097208648
## strata(ovigroup)Y_1                                         0.106828890
## frailty(Trial_start_date_factor, distribution = "gaussian") 0.005423386
## Tempscaled:Agescaled                                        0.040736419
##                                                             exp(coef)
## Tempscaled                                                  1.1678307
## Agescaled                                                   1.0465525
## strata(ovigroup)Y_0                                         1.8194804
## strata(ovigroup)Y_1                                         1.2578311
## frailty(Trial_start_date_factor, distribution = "gaussian") 1.0391399
## Tempscaled:Agescaled                                        0.9109372
##                                                             lower 0.95
## Tempscaled                                                   1.0762925
## Agescaled                                                    0.9574009
## strata(ovigroup)Y_0                                          1.5038455
## strata(ovigroup)Y_1                                          1.0202096
## frailty(Trial_start_date_factor, distribution = "gaussian")  1.0281528
## Tempscaled:Agescaled                                         0.8410340
##                                                             upper 0.95
## Tempscaled                                                   1.2671542
## Agescaled                                                    1.1440059
## strata(ovigroup)Y_0                                          2.2013623
## strata(ovigroup)Y_1                                          1.5507981
## frailty(Trial_start_date_factor, distribution = "gaussian")  1.0502445
## Tempscaled:Agescaled                                         0.9866505
##                                                                    z
## Tempscaled                                                   3.725352
## Agescaled                                                    1.001647
## strata(ovigroup)Y_0                                          6.157384
## strata(ovigroup)Y_1                                          2.147255
## frailty(Trial_start_date_factor, distribution = "gaussian")  7.079228
## Tempscaled:Agescaled                                        -2.289874
##                                                                        p
## Tempscaled                                                  1.950431e-04
## Agescaled                                                   3.165140e-01
## strata(ovigroup)Y_0                                         7.395654e-10
## strata(ovigroup)Y_1                                         3.177297e-02
## frailty(Trial_start_date_factor, distribution = "gaussian") 1.449618e-12
## Tempscaled:Agescaled                                        2.202859e-02
```

```
##
## Wald Chi-square=129.2855 on 6df, p=0, n=798
```

```
sink("Oviposition_survival/firstBM_coxphwsurvival_combinedoviposegg.txt")
fit4
summary(fit4)
sink()



fit4$coefficients
```

```
##              [,1]
## [1,]  0.15514794
## [2,]  0.04550146
## [3,]  0.59855094
## [4,]  0.22938890
## [5,]  0.03839339
## [6,] -0.09328129
## attr(,"names")
## [1] "Tempscaled"
## [2] "Agescaled"
## [3] "strata(ovigroup)Y_0"
## [4] "strata(ovigroup)Y_1"
## [5] "frailty(Trial_start_date_factor, distribution = \"gaussian\")"
## [6] "Tempscaled:Agescaled"
```

```
#extract coefficients to plot hazard ratios:
coef <- as.numeric(fit4$coefficients[1:6])
expcoef <- as.numeric(exp(fit4$coefficients[1:6]))
names <- names(fit4$coefficients[1:6])
lowerCI <- as.numeric(fit4$ci.lower[1:6])
upperCI <- as.numeric(fit4$ci.upper[1:6])
pval <- as.numeric(fit4$prob[1:6])
Index <- c(1:5)
Label <- names(fit4$coefficients[1:6])

hazardratiotable <- as.data.frame(cbind(Label,coef,expcoef,lowerCI,upperCI,pval))
hazardratiotable
```

```
##                                                          Label
## 1                                                   Tempscaled
## 2                                                    Agescaled
## 3                                         strata(ovigroup)Y_0
## 4                                         strata(ovigroup)Y_1
## 5 frailty(Trial_start_date_factor, distribution = "gaussian")
## 6                                        Tempscaled:Agescaled
##                 coef          expcoef          lowerCI          upperCI
## 1  0.155147940226498  1.16783071746686  1.07629249185332   1.2671542308269
## 2 0.0455014644849153  1.04655253727323 0.957400863417923  1.14400587582814
## 3  0.598550942296579  1.81948035719615  1.50384549714322  2.20136229187869
## 4  0.229388896733535   1.2578311103147  1.02020962304398  1.55079805790785
## 5 0.0383933882644104  1.03913993794001  1.02815275119004  1.05024453746987
## 6 -0.093281286817974 0.910937229620355 0.841033992028366 0.986650532765173
##                 pval
## 1  0.00019504310513152
## 2    0.316513965972134
## 3 7.39565408913734e-10
## 4   0.0317729656652621
## 5 1.44961820325307e-12
## 6   0.0220285937723677
```

```
hazardratiotable <- hazardratiotable[-5,]

hazardratiotable <- as.data.frame(cbind(Index,hazardratiotable))

print(hazardratiotable)
```

```
##   Index                Label               coef           expcoef
## 1     1           Tempscaled  0.155147940226498  1.16783071746686
## 2     2            Agescaled 0.0455014644849153  1.04655253727323
## 3     3  strata(ovigroup)Y_0  0.598550942296579  1.81948035719615
## 4     4  strata(ovigroup)Y_1  0.229388896733535   1.2578311103147
## 6     5 Tempscaled:Agescaled -0.093281286817974 0.910937229620355
##             lowerCI           upperCI                 pval
## 1  1.07629249185332   1.2671542308269  0.00019504310513152
## 2 0.957400863417923  1.14400587582814    0.316513965972134
## 3  1.50384549714322  2.20136229187869 7.39565408913734e-10
## 4  1.02020962304398  1.55079805790785   0.0317729656652621
## 6 0.841033992028366 0.986650532765173   0.0220285937723677
```

```
str(hazardratiotable)
```

```
## 'data.frame':    5 obs. of  7 variables:
##  $ Index  : int  1 2 3 4 5
##  $ Label  : chr  "Tempscaled" "Agescaled" "strata(ovigroup)Y_0" "strata(ovigroup)Y_1" ...
##  $ coef   : chr  "0.155147940226498" "0.0455014644849153" "0.598550942296579" "0.229388896733535" ...
##  $ expcoef: chr  "1.16783071746686" "1.04655253727323" "1.81948035719615" "1.2578311103147" ...
##  $ lowerCI: chr  "1.07629249185332" "0.957400863417923" "1.50384549714322" "1.02020962304398" ...
##  $ upperCI: chr  "1.2671542308269" "1.14400587582814" "2.20136229187869" "1.55079805790785" ...
##  $ pval   : chr  "0.00019504310513152" "0.316513965972134" "7.39565408913734e-10" "0.0317729656652621"
...
```

```
hazardratiotable$coef <- round(as.numeric(hazardratiotable$coef),digits = 3)
hazardratiotable$expcoef <- round(as.numeric(hazardratiotable$expcoef),digits = 3)
hazardratiotable$lowerCI <- round(as.numeric(hazardratiotable$lowerCI),digits = 3)
hazardratiotable$upperCI <- round(as.numeric(hazardratiotable$upperCI),digits = 3)
hazardratiotable$pval <- round(as.numeric(hazardratiotable$pval),digits = 3)
print(hazardratiotable)
```

```
##   Index                Label   coef expcoef lowerCI upperCI  pval
## 1     1           Tempscaled  0.155   1.168   1.076   1.267 0.000
## 2     2            Agescaled  0.046   1.047   0.957   1.144 0.317
## 3     3  strata(ovigroup)Y_0  0.599   1.819   1.504   2.201 0.000
## 4     4  strata(ovigroup)Y_1  0.229   1.258   1.020   1.551 0.032
## 6     5 Tempscaled:Agescaled -0.093   0.911   0.841   0.987 0.022
```

```
hazardratiotable$Label <- c("Temperature","Age","Has Opportunity: Did not lay eggs",
                            "Has opportunity: Laid eggs",
                            "Temperature:Age")

print(hazardratiotable)
```

```
##   Index                             Label   coef expcoef lowerCI upperCI  pval
## 1     1                       Temperature  0.155   1.168   1.076   1.267 0.000
## 2     2                               Age  0.046   1.047   0.957   1.144 0.317
## 3     3 Has Opportunity: Did not lay eggs  0.599   1.819   1.504   2.201 0.000
## 4     4        Has opportunity: Laid eggs  0.229   1.258   1.020   1.551 0.032
## 6     5                   Temperature:Age -0.093   0.911   0.841   0.987 0.022
```

```
plot1 <- ggplot(hazardratiotable, aes(y = Index, x = expcoef)) +
  geom_point(shape = 18, size =4) +
  geom_errorbarh(aes(xmin = lowerCI, xmax = upperCI), height = 0.25) +
  geom_vline(xintercept = 1, color = "gray", linetype = "dashed", cex = 1, alpha = 0.5) +
  scale_y_continuous(name = "",breaks = 1:5, labels = hazardratiotable$Label,  trans = "reverse") +
  scale_x_continuous(name = "Hazard Ratio (95% CI)") +
  xlab("Hazard Ratio (95% CI)") +
  ylab(" ") +
  theme_pubr() +
  theme(panel.border = element_blank(),
        panel.background = element_blank(),
        panel.grid.major = element_blank(),
        panel.grid.minor = element_blank(),
        axis.line = element_line(colour = "black"),
        axis.text.y = element_text(size = 12, colour = "black"),
        axis.text.x.bottom = element_text(size = 12, colour = "black"),
        axis.title.x = element_text(size = 12, colour = "black"))
plot1
```

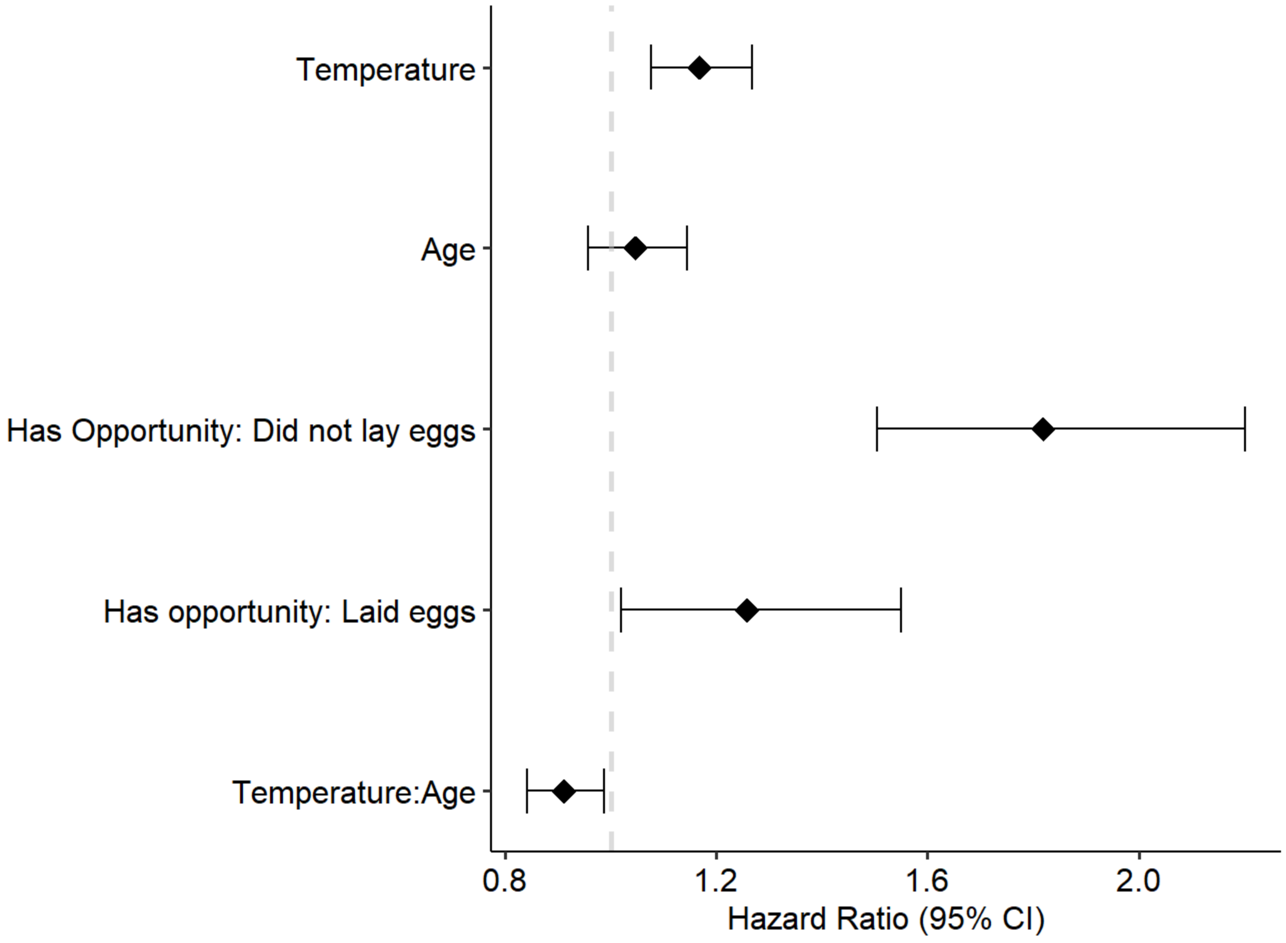


```
## Create the table-base pallete
table_base <- ggplot(hazardratiotable, aes(y=Label)) +
  ylab(NULL) + xlab("  ") +
  theme(plot.title = element_text(hjust = 0.5, size=12),
        axis.text.x = element_text(color="white", hjust = -3, size = 25), ## This is used to help with alig
nment
        axis.line = element_blank(),
        axis.text.y = element_blank(),
        axis.ticks = element_blank(),
        axis.title.y = element_blank(),
        legend.position = "none",
        panel.background = element_blank(),
        panel.border = element_blank(),
        panel.grid.major = element_blank(),
        panel.grid.minor = element_blank(),
        plot.background = element_blank())

## HR point estimate table
tab1 <- table_base +
  labs(title = "space") +
  geom_text(aes(y = rev(Index), x = 1, label = sprintf("%0.1f", round(expcoef, digits = 1))), size = 4) + #
# decimal places
  ggtitle("HR")
tab1
```

HR

1.2

1.0

1.8

1.3

0.9

```
#pval
tab3 <- table_base +
  geom_text(aes(y = rev(Index), x = 1, label = pval), size = 4) +
  ggtitle("P value")
tab3
```

P value
0
0.317
0
0.032
0.022

```
## Merge tables with plot
library(gridExtra)
lay <-  matrix(c(1,1,1,1,1,1,1,1,1,1,1,1,1,1,2,2,2), nrow = 1)
bucketsurvival_forest <- grid.arrange(plot1, tab3,layout_matrix = lay)
```

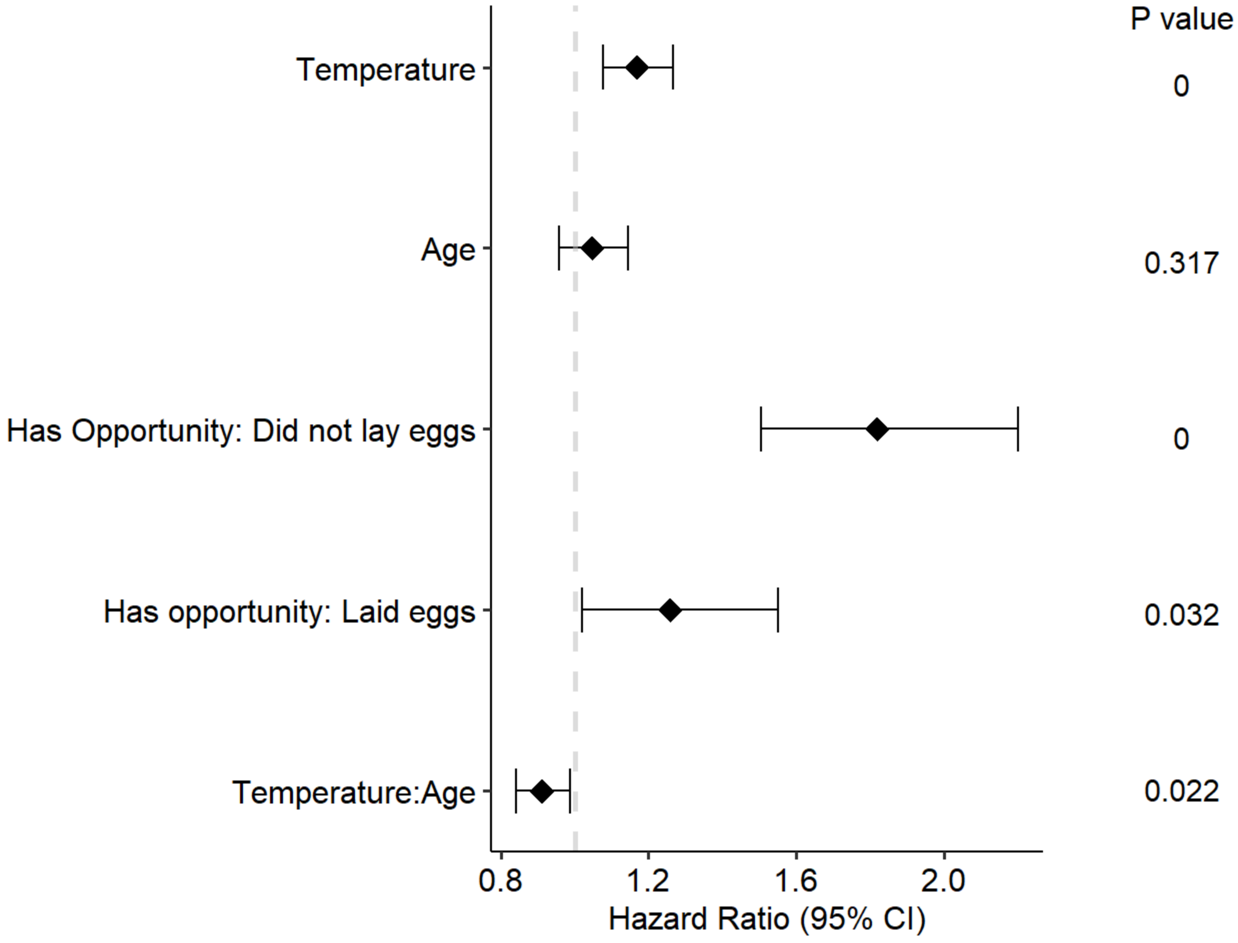


```
ggsave("Oviposition_survival/firstBM_hazardplot_combinedoviposegg.pdf",bucketsurvival_forest,dpi=600,width
= 7,height=5,units="in")
ggsave("Oviposition_survival/firstBM_hazardplot_combinedoviposegg.png",bucketsurvival_forest,dpi=600,width
= 7,height=5,units="in")
write_xlsx(hazardratiotable,"Oviposition_survival/firstBM_survival_hazardratiotable_combinedoviposegg.xls
x")
```

This completes the code for Figures 1-9 of the manuscript.
